# Ruthenium-Catalyzed Intermolecular [2 + 2] Cycloaddition of Unactivated Allenes and Alkynes with Unusual Regioselectivity

**DOI:** 10.1021/jacs.5c11285

**Published:** 2025-09-08

**Authors:** Chaoshen Zhang, Herman H. Y. Sung, Ian D. Williams, Yun-Dong Wu, Jianwei Sun

**Affiliations:** † Department of Chemistry and the Hong Kong Branch of Chinese National Engineering Research Centre for Tissue Restoration & Reconstruction, 58207 The Hong Kong University of Science and Technology, Clear Water Bay, Kowloon, Hong Kong SAR 999077, China; ‡ Shenzhen Bay Laboratory, Shenzhen 518132, China; § Lab of Computational Chemistry and Drug Design, State Key Laboratory of Chemical Oncogenomics, Peking University Shenzhen Graduate School, Shenzhen 518055, China

## Abstract

Described here is an efficient protocol for intermolecular [2 + 2] cycloaddition of unactivated and unsymmetrical allenes and alkynes with unusual regioselectivity, counterintuitively favoring the most hindered isomer. CpRu­(MeCN)_3_PF_6_ served as a uniquely effective catalyst, providing diverse 3-alkylidenecyclobutenes with a broad scope and good functional group compatibility. Both experiments and DFT studies provided important insights into the mechanism, particularly the unusual regioselectivity.

## Graphical abstract

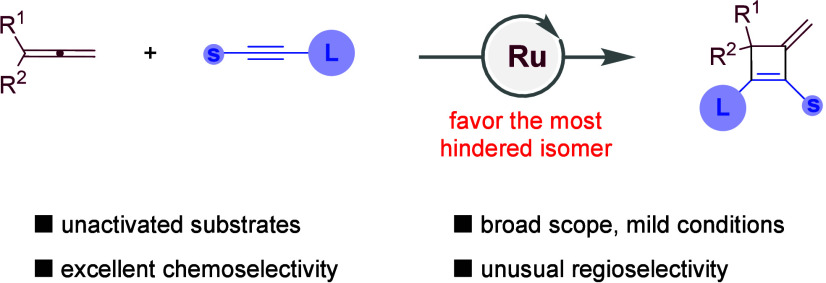

Alkylidenecyclobutene and related four-membered carbocycles are unique substructures present in diverse natural and bioactive molecules.^1^ Owing to the high ring strain and two reactive C = C bonds, they also have broad applications in organic synthesis.^2^ The [2 + 2] cycloaddition of allenes^3^ and alkynes^4^ represents arguably the most direct approach to access 3-alkylidenecyclobutenes (Scheme 1a).^5–9^ However, both allenes and alkynes have reactive π-systems that can undergo homo- and cross- [2 + 2] and [2 + 2 + 2] cycloadditions, with the latter leading to more stable 6-membered (often aromatic) products.^10,11^ Moreover, for terminal alkynes, hydroalkynylation of allenes is another well-known process, which may lead to multiple regio- and stereoisomers.^12,13^ Therefore, the chemoselective generation of a highly strained cross-[2 + 2] cycloadduct among so many thermodynamically favorable pathways represents a formidable challenge. Consequently, although this process has been known for over a half century, limited success has been achieved regarding substrate generality, efficiency, regioselectivity, and mild conditions. If fact, current success has been largely limited to activated substrates (e.g., electron-rich allenes and electron-poor alkynes)^6^ and/or intramolecular processes,^7^ where the electronic bias or geometric constraints help simplify the complications.

**Scheme 1:**
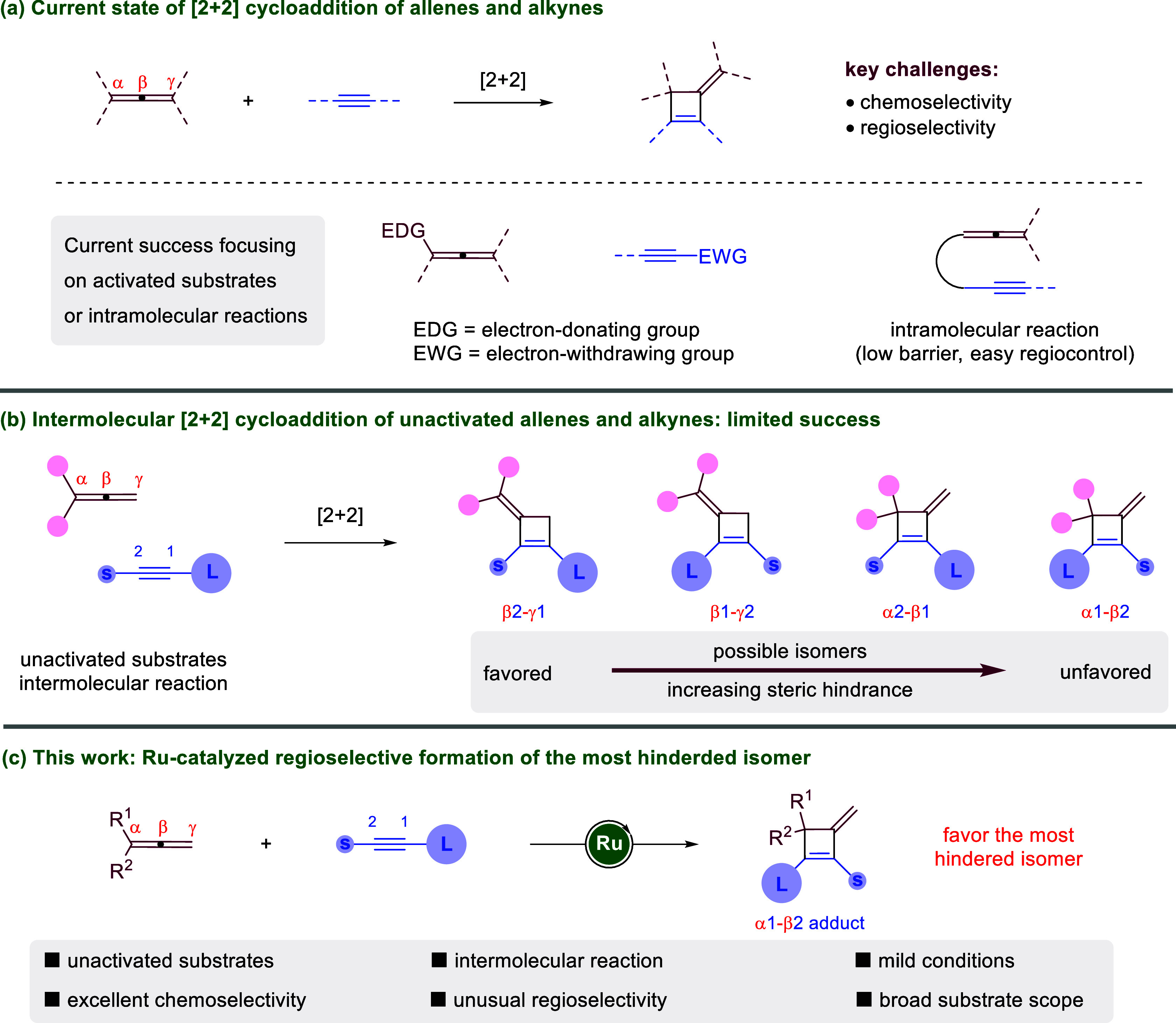
Introduction to [2 + 2] Cycloaddition of Allenes and Alkynes

Indeed, there has been almost no progress in developing efficient *intermolecular* [2 + 2] cycloadditions between *unactivated* allenes and alkynes.^8,9^ Moreover, when both allenes and alkynes are unsymmetrical but lack electronic bias, controls over regioselectivity are required, which constitutes another substantial challenge (Scheme 1b). Among the four possible isomers, the β2-γ1 adduct is least hindered, whereas the α1-β2 adduct contains all the bulky groups in proximity and is least favored (Scheme 1b). In 2019, the Yoshikai laboratory pioneered an efficient protocol favoring the least hindered β2-γ1 adduct using cobalt catalysis.^8b^ Though limited to internal alkynes and with moderate regioselectivity for unsymmetrical cases, this represents an important milestone for unactivated substrates in an intermolecular reaction. In contrast, selective formation of other isomers is more challenging. In continuation of our efforts in alkyne and allene functionalization,^13,14^ here we report our progress by developing such a mild efficient with excellent regioselectivity favoring the most hindered (α1−β2) isomer (Scheme 1c).

We employed allene **1a** and terminal alkyne **2a** as the model substrates. Ruthenium complexes were examined as catalysts, as they are known for alkyne/allene functionalization with unorthodox selectivity.^13–15^ [Cp*RuCl]_4_, previously used for α-selective hydrofucntionalization of allenes,^13^ was unable to catalyze the desired [2 + 2] cycloaddition (Table 1, entry 1). Other complexes, including Cp*Ru­(cod)­Cl, Ru­(PPh_3_)_3_Cl_2_, and CpRu­(cod)­Cl, did not provide the desired product either (entries 2–4). Next, we resorted to Cp*Ru­(MeCN)_3_PF_6_, a cationic complex known for alkyne *trans*-addition.^14,15^ Encouragingly, the desired [2 + 2] cycloadduct **3a** was formed, albeit in low yield (entry 5). More importantly, only the most hindered α1-β2 regioisomer was observed (>20:1 rr). We believe that the cationic nature of the metal center combined with weak ligands contributed significantly to the high activity by facilitating ligand exchange and substrate binding. We then evaluated the less hindered complex CpRu­(MeCN)_3_PF_6_, which significantly improved the chemoselectivity and yield (78% yield, entry 6). The same reaction provided much lower efficiency in other solvents (e.g., halogenated or polar solvents) than THF (entries 7–11). However, increasing the concentration to 0.1 M improved the yield (entry 12), but no improvement was observed by further increasing the concentration (entry 13). The sensitivity to concentration might be related to some competing side reactions that may have different kinetic dependence on substrates. For comparison, the reaction did not proceed in the absence of a catalyst, thus confirming the essential role of the ruthenium catalyst (entry 14). Notably, this catalytic process proceeded efficiently at room temperature, which is remarkable since almost all previous reported cases required an elevated temperature, including intramolecular ones or activated substrates.^6–8^ Other competing reactions, including hydroalkynylation and allene dimerization, were inhibited under the present conditions.

**Table 1:**
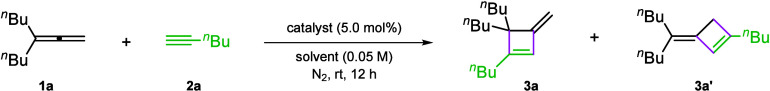
Condition Optimization^a^

entry	catalyst	solvent	conv (%)^b^	yield (%)^b^	**3a**/**3a′** ^b^
1	[Cp*RuCl]_4_	THF	67	0	–
2	Cp*Ru(cod)Cl	THF	68	0	–
3	Ru(PPh_3_)_3_Cl_2_	THF	24	0	–
4	CpRu(cod)Cl	THF	46	0	–
5	Cp*Ru(MeCN)_3_PF_6_	THF	>99	36	>20:1
6	CpRu(MeCN)_3_PF_6_	THF	>99	78	>20:1
7	CpRu(MeCN)_3_PF_6_	EtOAc	>99	28	>20:1
8	CpRu(MeCN)_3_PF_6_	DCM	38	0	–
9	CpRu(MeCN)_3_PF_6_	DMF	>99	46	7:4
10	CpRu(MeCN)_3_PF_6_	PhCl	>99	20	4:1
11	CpRu(MeCN)_3_PF_6_	MeCN	>99	68	>20:1
12^c^	CpRu(MeCN)_3_PF_6_	THF	>99	89	>20:1
13^d^	CpRu(MeCN)_3_PF_6_	THF	>99	78	>20:1
14	–	THF	0	–	–

a **1a** (0.05 mmol), **2a** (0.07 mmol), catalyst (5.0 mol % based on metal), solvent (1.0 mL).

b Determined by ^1^H NMR analysis of the crude product with CH_2_Br_2_ as an internal standard.

c *c* = 0.1 M, 6 h.

d *c* = 0.2 M, 6 h.

Next, we examined the alkyne scope (Scheme 2). Various terminal alkynes substituted with alkyl-, alkenyl-, and aryl- groups all efficiently reacted smoothly to furnish the corresponding cyclobutenes, favoring the hindered isomer with exceptional regioselectivity (**3a**–**3w**). The mild conditions tolerated diverse functional groups, including silyl, germanyl, halide, nitrile, (thio)­ether, mesyl, acetal, ketone, ester and even free alcohol. Electron-rich aryl acetylenes also showed good reactivity, although they are known to deactivate the catalyst by π-coordination. 1,4-Diethynylbenzene underwent double cycloaddition successfully (**3x**). This protocol was also applicable to internal alkynes, maintaining the unusual excellent regioselectivity (**3y**–**3ag**). Complex alkynes derived from active pharmaceutical molecules also reacted with equally high efficiency. Of note, some products were generated in low yield (e.g., **3l**, **3m**, **3ac**) due to oligomerization of the alkyne partner,^10a–10c,11d,11e^ but the regioselectivity remained excellent in these reactions. The low yield of **3t** might be related to the thioether motif that may deactivate the catalyst. The structure of **3ag** was unambiguously confirmed by X-ray crystallography. 2D NMR and NOE studies were also performed on other representative products.

**Scheme 2:**
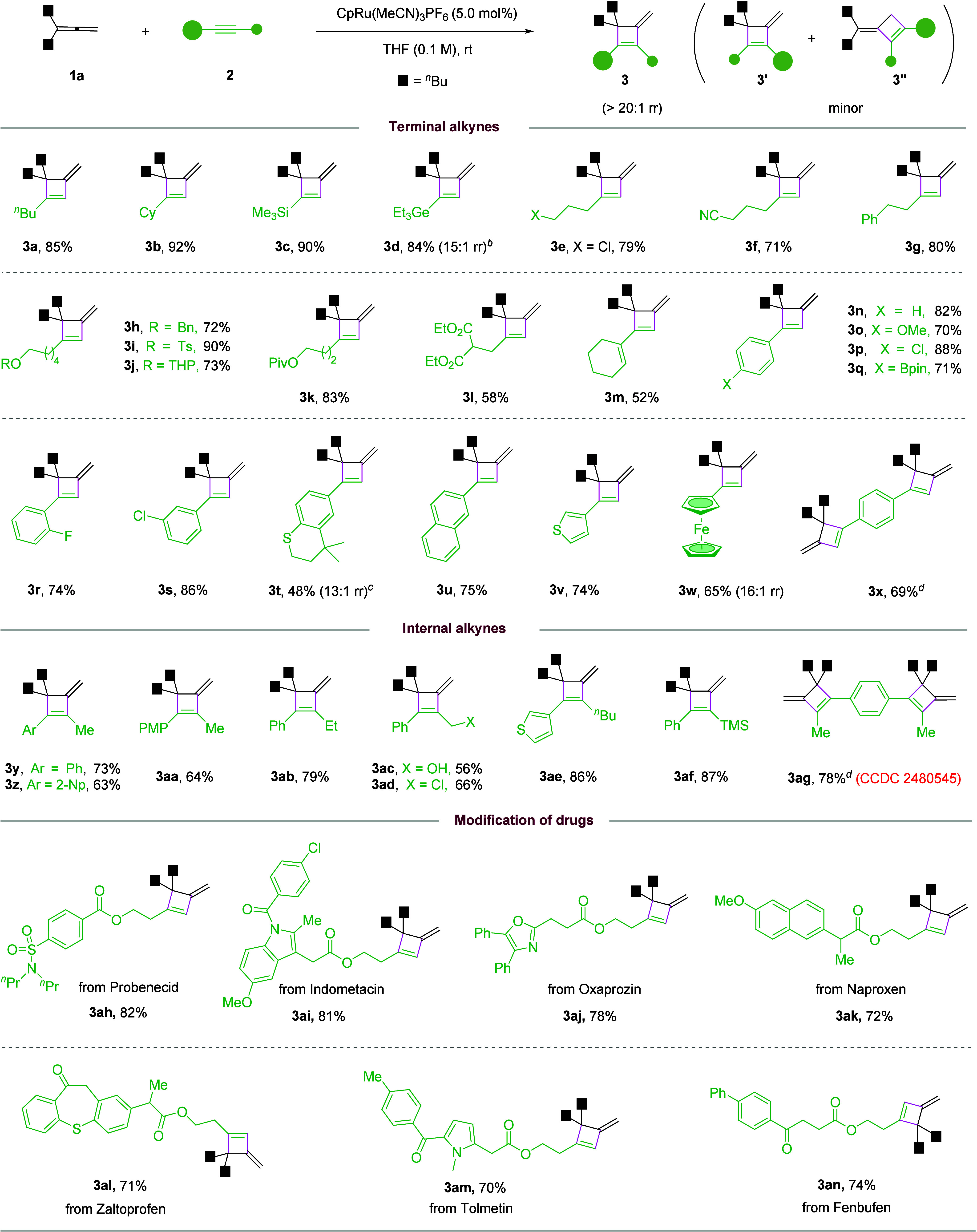
Scope of Alkynes[Fn s2fn1]

a
**1a** (0.30 mmol), **2** (0.42 mmol), CpRu­(MeCN)_3_PF_6_ (5.0 mol %), THF (3.0 mL), 6–12 h. Isolated yield, >20:1 rr.
b 0 °C, 8 h.
c 10 mol % of catalyst, 12 h.
d
**1a** (0.72 mmol), **2x** or **2ag** (0.30 mmol). PMP = (*p*-OMe)­C_6_H_4_.

Regarding the generality of allenes, both monosubstituted and 1,1-disubstituted allenes with aryl and alkyl groups all led to the desired products (**4a**–**4m**) with moderate to good efficiency and excellent regioselectivity (Scheme 3). Increasing the size of the substituents (**4k**–**4m**) had no detrimental effect. Such hindered products were not easily accessible by conventional methods. Moderate yields were observed for some monosubstituted allenes (e.g., **4a**–**4d**), presumably due to substrate dimerization/oligomerization.^10d,11^ We also tried di-, tri-, and tetrasubstituted internal allenes, but no desired [2 + 2] cycloaddition products were obtained (see the SI for details).

**Scheme 3:**
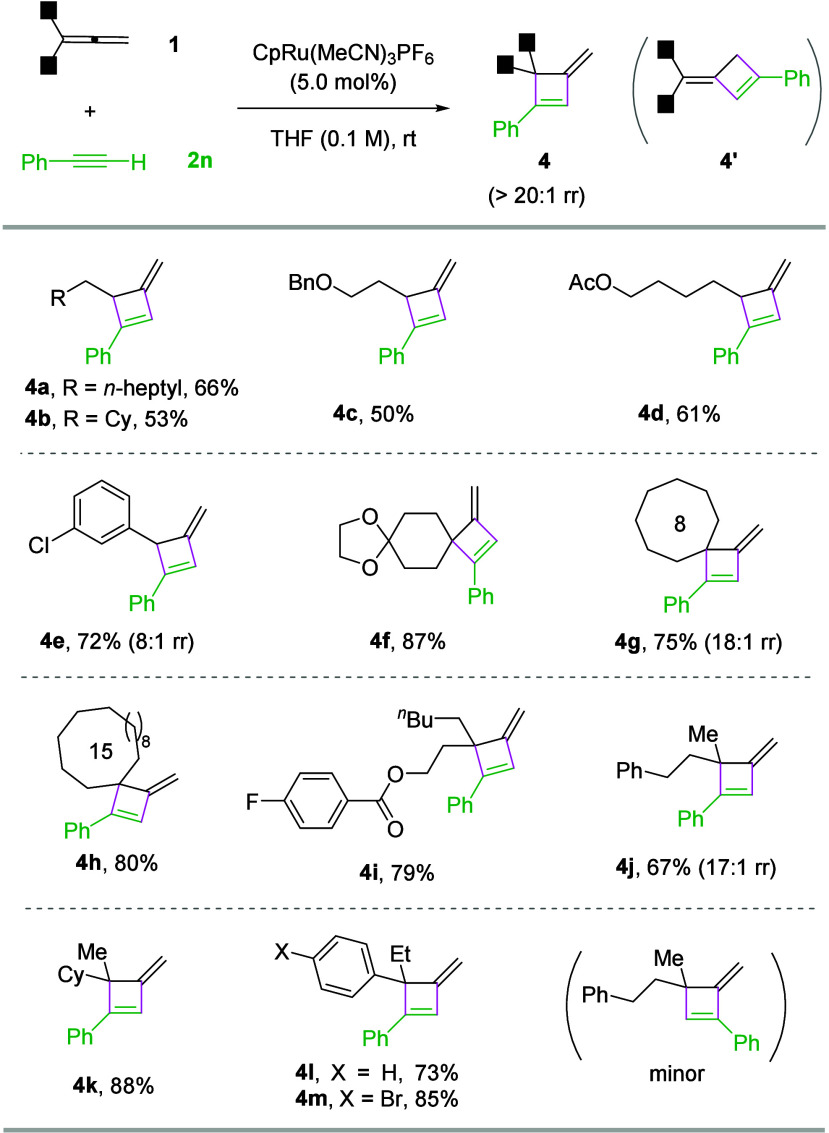
Scope of Allenes[Fn s3fn1]

a
**1** (0.3 mmol), **2n** (0.42 mmol), CpRu­(MeCN)_3_PF_6_ (5.0 mol %), THF (3.0 mL), 6 h. Isolated yield. >20:1 rr.

This protocol is robust for scale-up synthesis. With only 1 mol % of catalyst, a 2 mmol reaction of **1a** and **2n** afforded **3n** in 80% yield (Scheme 4). The product could be further functionalized. For example, the exocyclic olefin reacted with *in situ* generated Rh-carbene and benzyne to form spiro cyclopropane **6** (>20:1 dr) and double cyclobutene **7**, respectively. Interestingly, *m*-CPBA promoted an oxidative rearrangement to form cyclopentenone **8**, likely via epoxide **8a** followed by House–Meinwald Rearrangement to **8b** and a Rubottom oxidation.

**Scheme 4:**
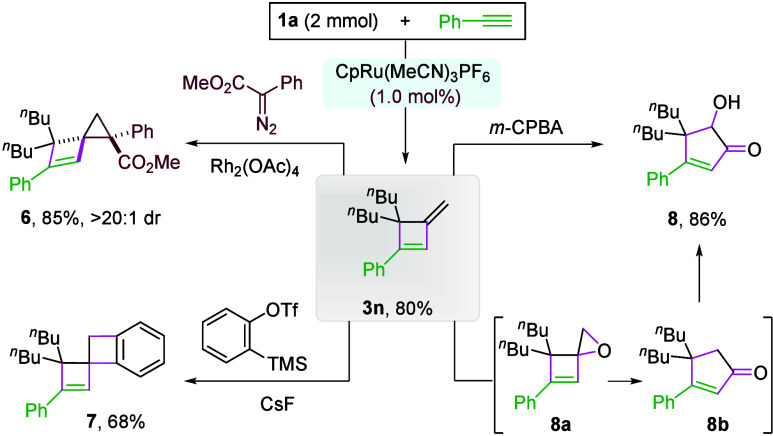
Scale-up Reaction and Product Transformation

Control experiments and kinetic studies were performed to help understand the mechanism (Figure 1). The reaction of **1e** with deuterated phenylacetylene afforded **4d**-*d*_1_ with complete D-incorporation at the same carbon without scrambling (Figure 1a). Moreover, a mixture of two different aryl alkynes, one of which was deuterated, resulted in a mixture without intermolecular deuterium crossover. These observations suggested that the cleavage of C­(*sp*)–H bond may not be involved, thus distinct from allene hydroalkynylation.^12,13^ To know more about the relationship between regioselectivity and the alkyne size, we made a plot between the observed regioisomeric ratio (rr) and the A-value of the alkyne substituent. In fact, a reverse linear relationship was observed (Figure 1b), suggesting that the bulkier alkyne tends to give a lower regioisomeric ratio (e.g., rr = 2 for ^*t*^Bu group, more details in the SI).

**Figure 1:**
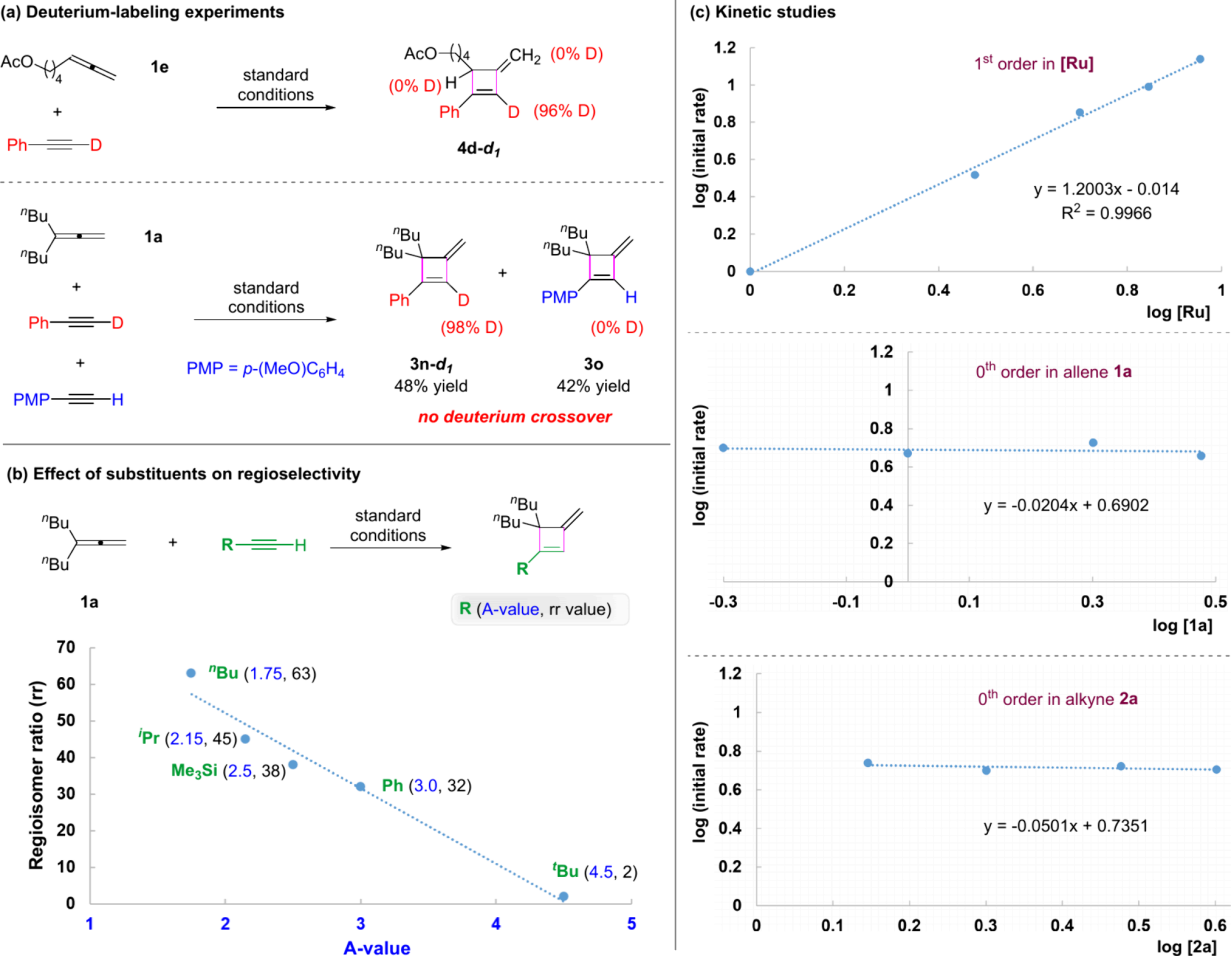
Control experiments and kinetic studies.

Kinetic studies were performed. This reaction exhibits zeroth order in allene **1a** and alkyne **2a**, and first order in catalyst (Figure 1c), implying that the catalyst might be saturated by the two substrates by forming a stable complex, serving as the catalyst resting state before the rate-determining step.

## DFT Studies

To shed more light on the mechanism, density functional theory (DFT) calculations were performed using the model reaction of **1a** and **2a**. All energies discussed are relative Gibbs free energies at room temperature in kcal/mol (see the SI for details). As shown in Figure 2, the reaction starts with ligand exchange to form complex **C-IM1a**, which is slightly endothermic. Next, oxidative cyclometalation occurs to form a ruthenacyclopentene. There are 8 permutations regarding which double bond in allene to participate and the orientations of each partner. Among them, **C-TS1a** is least hindered and has the lowest energy (15.5 kcal/mol, Figure 2a). In contrast, **A-TS1a**, seemingly leading to the observed product, has the highest energy (29.3 kcal/mol). While this step can be a pre-equilibrium and not regio-determining, it is unlikely for this process to proceed via **A-TS1a** considering the high efficiency at room temperature.

**Figure 2:**
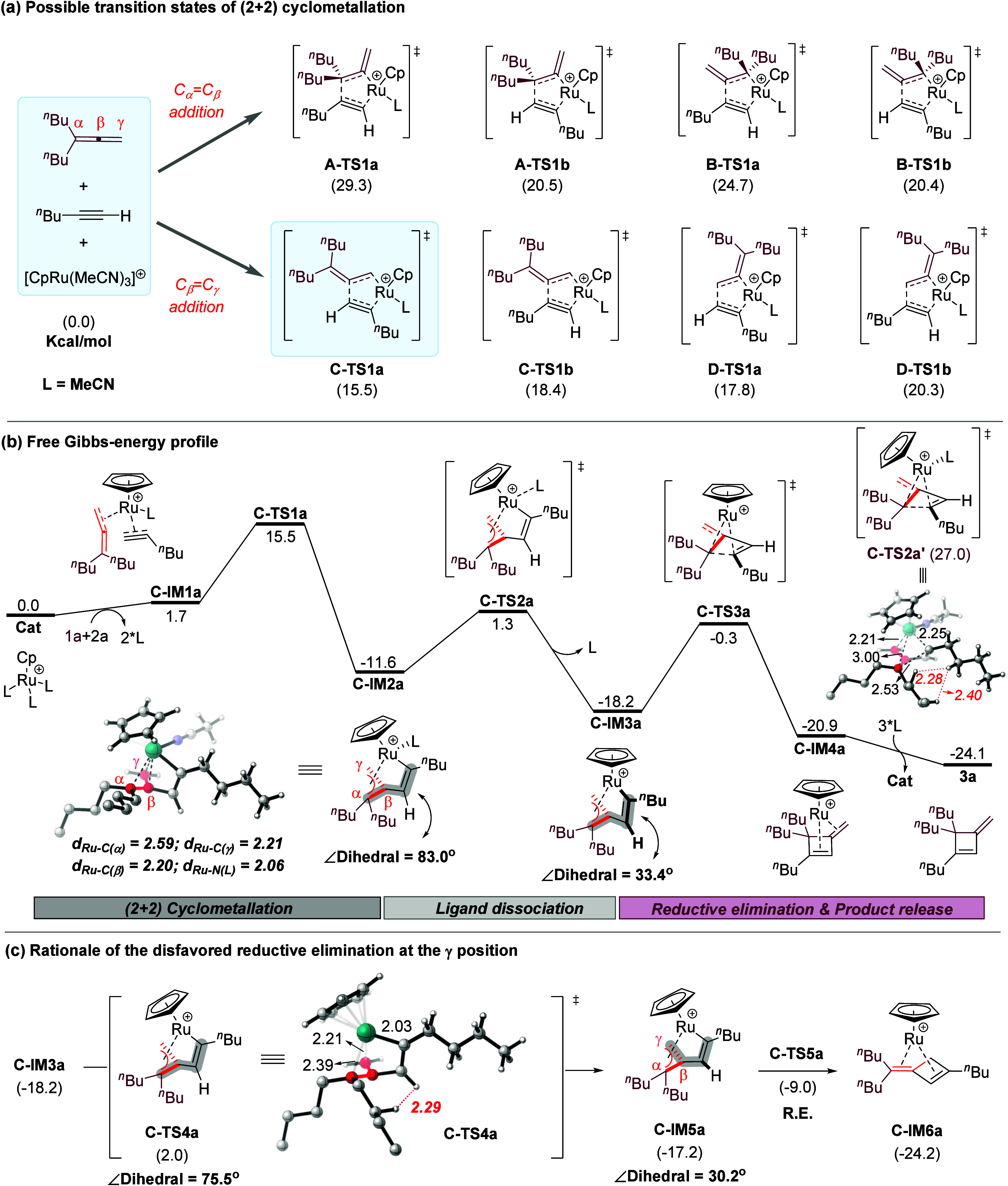
Gibbs-free energy profile (kcal/mol) for the [2 + 2] cycloaddition of allene **1a** and alkyne **2a**. The selected bond lengths in the key structures are labeled in Å. R.E., reductive elimination.

Next, we investigated the subsequent steps from **C-TS1a**. Notably, by adopting a π-allyl complexation form, the resulting **C-IM2a** gains additional stabilization. Structural analysis showed that the dihedral angle between C_α_–C_β_ bond and the Ru-attached C=C bond is almost perpendicular (83.0°) in **C-IM2a**. Unfortunately, direct reductive elimination is kinetically inaccessible, with a barrier as high as 38.6 kcal/mol (**C-TS2a′** relative to **C-IM2a**, see Figure S13 for details). Nevertheless, upon dissociation of a ligand, **C-IM2a** gains substantial stabilization by forming **C-IM3a** (Figure S14) after crossing a low barrier of 12.9 kcal/mol via **C-TS2a**. The detailed intrinsic reaction coordinate (IRC) calculations indicated that the above-mentioned dihedral angle gradually decreases to 33.6° in this step, with the vinyl ruthenium approaching toward C_α_ but the Cp ligand moving away (Figure S15). This sets the stage for a facile reductive elimination at C_α_ to form **C-IM4a**, which then releases the observed product **3a**. Notably, the rate-determining step is reductive elimination with a total barrier of 17.9 kcal/mol, in agreement with the mild conditions. Moreover, **C-IM3a** is a stable resting state, in which the catalyst is saturated by the two substrates, thus consistent with the kinetic results showing pseudo zeroth order in both allene and alkyne.

Although the above pathway well explains the experimental results, a key question remains unanswered, i.e., why the vinyl ruthenium does not approach the more accessible γ-position. Indeed, we examined the pathway toward reductive elimination at the γ-position. For this to take place, an additional rotation of the vinyl ruthenium motif toward the γ-position is required. Indeed, once this rotation is achieved, the reductive elimination via **C-TS5a** to form the least hindered isomer is both kinetically and thermody namically favored. However, the crucial rotation barrier is surprisingly high (20.2 kcal/mol, **C-TS4a**), likely due to severe steric clashes between the substituents (e.g., 2.29 Å). Overall, the energy barrier difference between the two pathways (2.3 kcal/mol, **C-TS4a** vs **C-TS3a**) is sufficient to ensure a high regioselectivity observed experimentally (see Figure S16 in the SI for details). We also examined the hydroalkynylation pathway, which requires a higher energy barrier (>25.5 kcal/mol, see Figure S17) and thus explains the preference for [2 + 2] cycloaddition.

Finally, based on the above mechanism, we were curious about whether a suitable alkyne can reverse the relative energies of the two key transition states (e.g., **C-TS4a** and **C-TS3a**). We first performed studies on a more hindered triisopropylsilyl (TIPS) alkyne (Figure 3a and more details in Figures S18–20 in the SI). DFT studies suggested that the direct reductive elimination at the α position suffered extremely severe steric repulsion between the bulky TIPS group and both the allene and Cp moieties (**C-TS7a**). In contrast, the rotation via **C-TS8a** and subsequent reductive elimination at the γ-position is relatively more facile (Figures S21–S22), thus implying reversal of regioselectivity. Indeed, under the standard conditions, both TIPS- and Ph_3_Si-substituted alkynes reacted exclusively on the less hindered γ-position, thereby consistent with the theoretical studies (Figure 3b), which also provided support to the overall reaction pathway.

**Figure 3:**
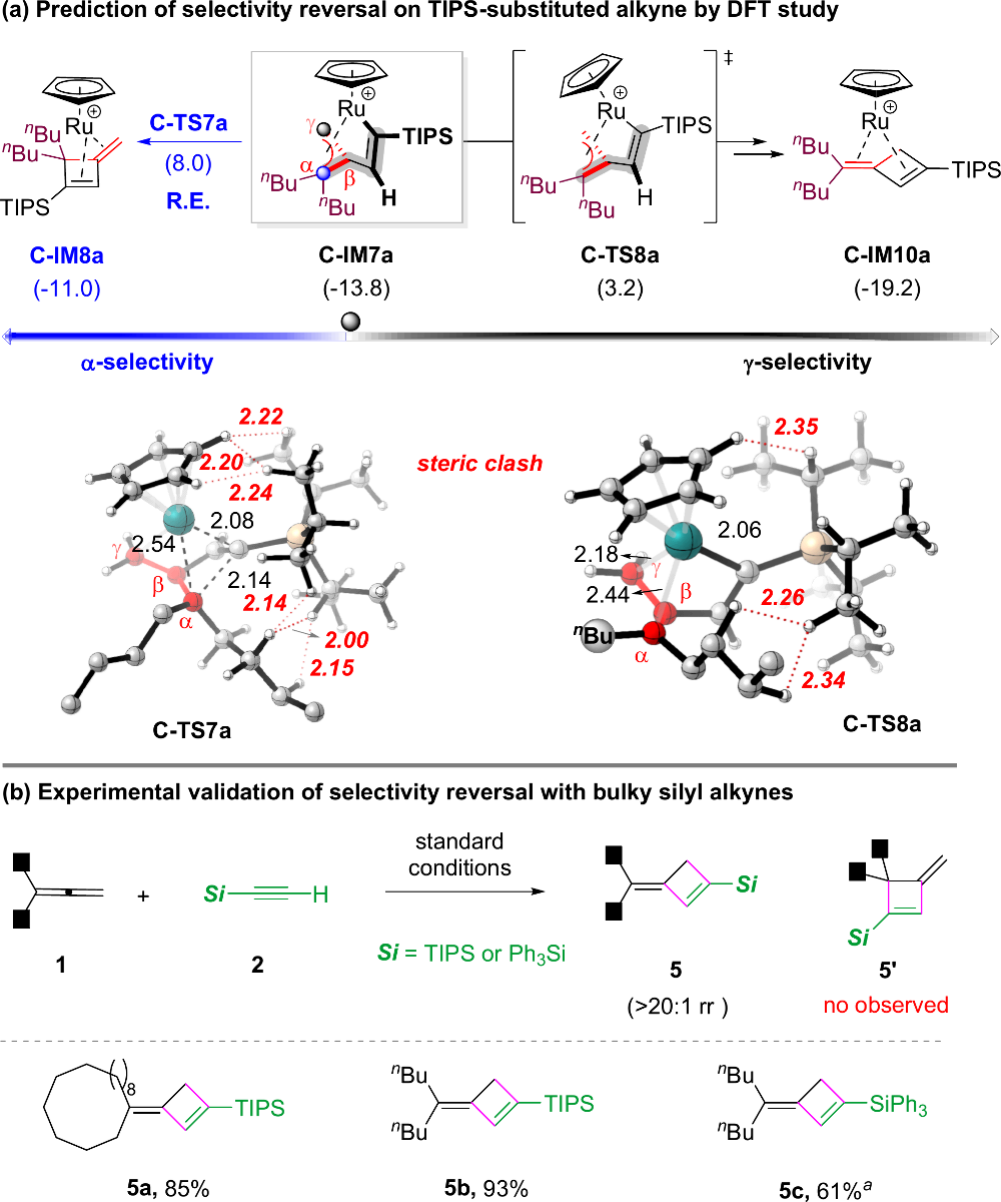
(a) DFT studies on the key transition states with TIPS-substituted alkyne. (b) Experimental validation of the regioselectivity reversal using bulky silyl alkynes. ^*a*^Run in MeCN.

In conclusion, we have developed a highly efficient and mild protocol for intermolecular [2 + 2] cycloaddition of unactivated allenes and alkynes, an important process with long history but with limited success in achieving high chemoselectivity and regioselectivity for unsymmetrical unactivated substrates. More importantly, our protocol not only achieved excellent regioselectivity but also counterintuitively favored the most hindered isomer. Cationic complex CpRu­(MeCN)_3_PF_6_ served as a uniquely effective catalyst in promoting the formation of the strained cyclobutene products with excellent chemoselectivity, overcoming many competing pathways, such as homo- and cross- [2 + 2 + 2] cycloadditions and hyroalkynylations. This protocol features mild conditions, a broad scope, and good functional group compatibility. The products can be easily converted to other functionalized strained molecules that are not straightforward to access. Both experiments and DFT studies provided important insights into the mechanism, which involves an initial oxidative cyclometalation, ligand dissociation, followed by reductive elimination. In contrast to the direct reductive elimination at the more hindered α-position, the key π-allyl ruthenium intermediate must undergo an energetically unfavored rotation to allow reductive limitation at the less hindered γ position, which explains the unusual regioselectivity. Finally, DFT studies predicted a possible reversal of this unusual regioselectivity for bulky silyl alkynes, which was confirmed experimentally.

## Supplementary Material



## Notes

Experimental procedures, spectroscopic data for substrates and products, and DFT calculations data (PDF)
