# Supplementary material for: Ruthenium-Catalyzed Intermolecular [2 + 2] Cycloaddition of Unactivated Allenes and Alkynes with Unusual Regioselectivity
Source: J Am Chem Soc. 2025 Sep 8;147(37):33350–8. doi: 10.1021/jacs.5c11285 (PMC12447507; doi:10.1021/jacs.5c11285)
Supplement: Supplementary file 1 [file ja5c11285_si_001.pdf]

Supporting Information

Ruthenium-Catalyzed Intermolecular [2+2] Cycloaddition of Unactivated Allenes and Alkynes with Unusual Regioselectivity

Chaoshen Zhang,[1] Herman H. Y. Sung,[1] Ian D. Williams,[1]
Yun-Dong Wu,[2,3*] and Jianwei Sun[1*]

[1]*Department of Chemistry and the Hong Kong Branch of Chinese National Engineering Research Centre for Tissue Restoration & Reconstruction, The Hong Kong University of Science and Technology, Clear Water Bay, Kowloon 999077, Hong Kong SAR, China*

[2]*Shenzhen Bay Laboratory, Shenzhen 518132, China*

[3]*Lab of Computational Chemistry and Drug Design, State Key Laboratory of Chemical Oncogenomics, Peking University Shenzhen Graduate School, Shenzhen 518055, China*

Table of Contents

I. General Information S-2

II. Substrate Preparation S-3

III. Ru-Catalyzed Regioselective [2+2] Cycloaddition S-10

IV. Scale-up Reaction and Product Transformations S-49

V. Mechanistic Experiments S-52

VI. Product Structure Determination S-63

VII. Computational Studies S-65

VIII. References S-219

NMR Spectra

I. General Information

All reagents were purchased and used without further purification unless otherwise specified. Flash column chromatography was performed over silica gel (200-300 mesh) purchased from Qindao Haiyang Co., China, or Silicycle P60 silica (230-400 mesh, 40-63 μm). All air or moisture sensitive reactions were conducted in oven-dried glassware under nitrogen atmosphere using anhydrous solvents. Anhydrous diethyl ether, dichloromethane and acetonitrile were purified by the Innovative® solvent purification system. Anhydrous tetrahydrofuran, 1,2-dichloroethane (DCE) and 1,4-dioxane were purchased from J&K Scientific® Chemicals and used as received. Anhydrous triethylamine was purchased from Scharlau® and used as received. All the ruthenium catalysts were purchased from Strem®. ^{1}H, ^{13}C, ^{19}F NMR spectra were collected on a Bruker AV 400 MHz NMR spectrometer using residue solvent peaks as an internal standard (^{1}H NMR: $CDCl_3$ at 7.26 ppm, ^{13}C NMR: $CDCl_3$ at 77.0 ppm). Data for ^{1}H NMR were recorded as follows: chemical shift (δ, ppm), multiplicity (s = singlet; d = doublet; t = triplet; q = quartet; p = pentet; m = multiplet), coupling constant (Hz), integration. Mass spectra were collected on an Agilent GC/MS 5975C system, a MALDI Micro MX mass spectrometer, or an API QSTAR XL System.

II. Substrate Preparation

Alkynes **2a–2c**, **2e–2g**, **2l–2y**, **2ab–2ad**, and **2af** are commercially available. Alkynes **2d**, **2h–2k**, **2z**, **2aa**, **2ae**, **2ag–2an** were prepared according to the literature procedures and purified by flash chromatography. Spectral data of the known alkynes **2d**,[1] **2h**,[2] **2i**,[3] **2j**,[4] **2k**,[5] **2z**,[6] **2aa**,[7] **2ae**,[8] **2ag**,[9] **2ah**,[10] **2ai**,[11] **2ak**[11], **2am**,[12] and **2an**[13] are consistent with the literature data. The preparation procedures and characterization data for new alkynes are shown below.

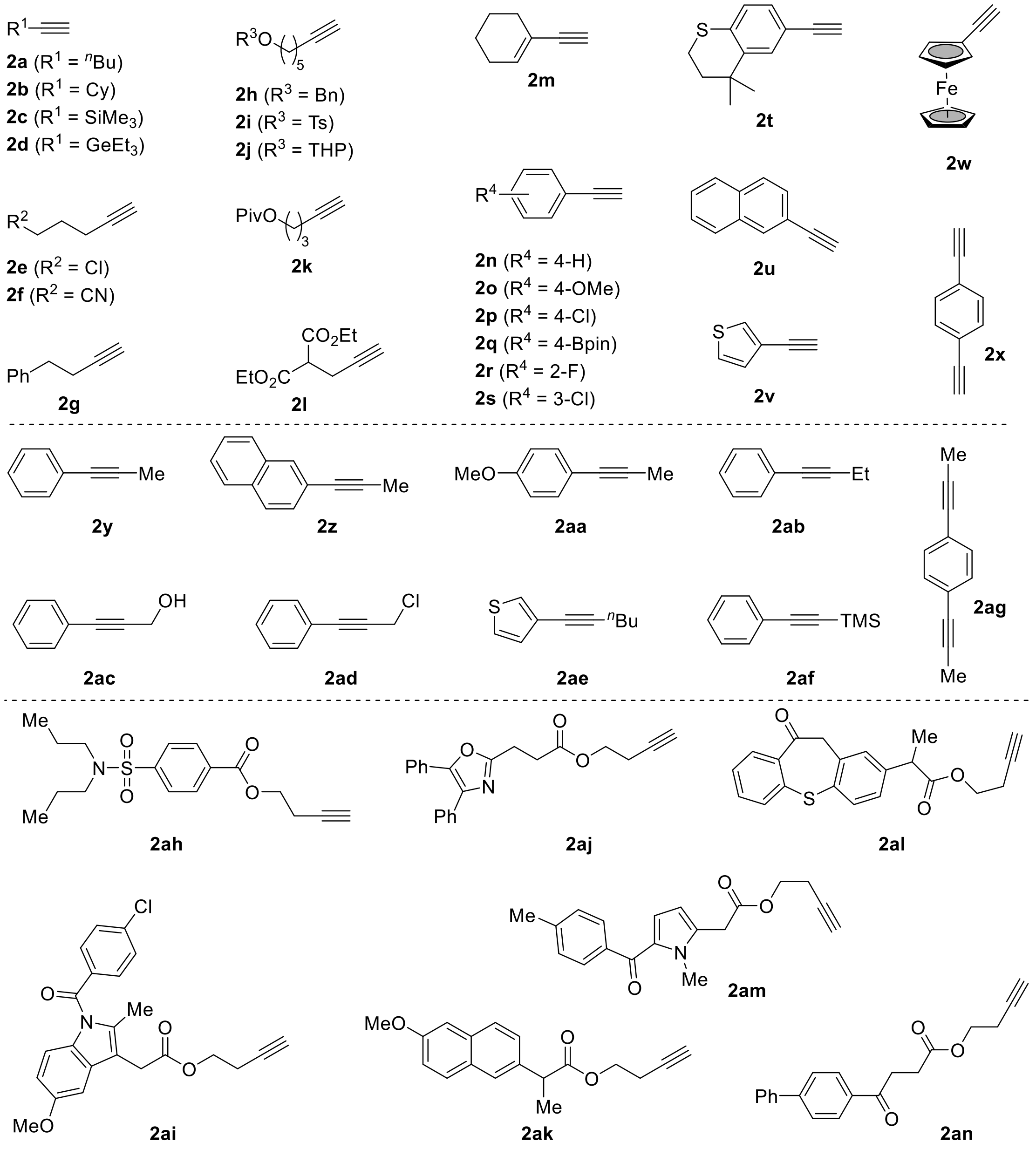

Figure S1. Alkynes used in this work.

General Procedure A.

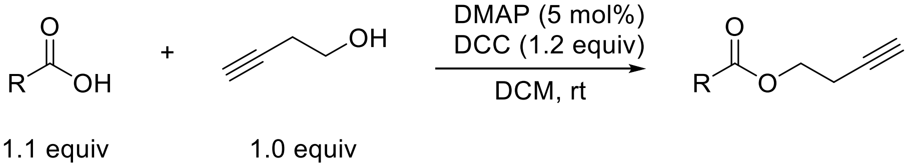

To a 50-mL oven-dried flask were added the carboxylic acid (5.5 mmol, 1.1 equiv), DCM (5.0 mL), 3-butyn-1-ol (5.0 mmol, 378 μL, 1.0 equiv), DMAP (0.25 mmol, 5 mol%, 30.5 mg) and a solution of DCC (6 mmol, 1.238 g, 1.2 equiv) in DCM (5.0 mL). The reaction mixture was stirred at room temperature for 12 h. The progress was monitored by thin-layer chromatography. Upon completion, the reaction mixture was filtered through a pad of silica gel, which was washed with DCM. The filtrate was concentrated under the reduced pressure, and the residue was purified by column chromatography on silica gel to afford the desired product.

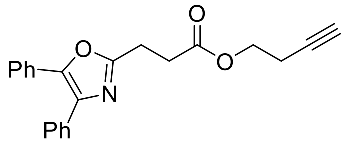

2aj

But-3-yn-1-yl 3-(4,5-diphenyloxazol-2-yl)propanoate (2aj) was prepared as white solid from 3-(4,5-diphenyloxazol-2-yl)propanoic acid (1.61 g, 5.5 mmol, 1.1 equiv) according to the General Procedure A (eluent: *n*-hexane/ethyl acetate = 10:1, 1.59 g, 92% yield).

^{1}H NMR (400 MHz, $CDCl_3$) δ 7.67 – 7.62 (m, 2H), 7.60 – 7.54 (m, 2H), 7.39 – 7.27 (m, 6H), 4.24 (t, *J* = 6.8 Hz, 2H), 3.19 (t, *J* = 7.5 Hz, 2H), 2.94 (t, *J* = 7.5 Hz, 2H), 2.53 (td, *J* = 6.8, 2.7 Hz, 2H), 1.98 (t, *J* = 2.7 Hz, 1H).

^{13}C NMR (100 MHz, $CDCl_3$) δ 171.6, 161.5, 145.3, 135.0, 132.3, 128.8, 128.5, 128.4, 128.3, 127.9, 127.7, 126.3, 79.8, 69.9, 62.3, 30.8, 23.3, 18.8.

HRMS (ES+) calcd for $C_{22}H_{19}NO_3Na^+$ $[M+Na]^+$: 368.1257, found: 368.1269.

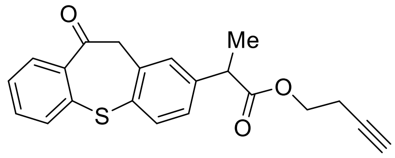

2al

But-3-yn-1-yl 2-(10-oxo-10,11-dihydrodibenzo[b,f]thiepin-2-yl)propanoate (2al) was prepared as a yellow oil from 2-(10-oxo-10,11-dihydrodibenzo[b,f]thiepin-2-yl)propanoic acid (1.64 g, 5.5 mmol, 1.1 equiv) according to the General Procedure A (eluent: *n*-hexane/ethyl acetate = 10:1, 1.57 g, 90% yield).

^{1}H NMR (400 MHz, $CDCl_3$) δ 8.18 (dd, *J* = 8.0, 1.6 Hz, 1H), 7.61 – 7.54 (m, 2H), 7.44 – 7.36 (m, 2H), 7.29 (ddd, *J* = 8.4, 7.2, 1.3 Hz, 1H), 7.15 (dd, *J* = 8.0, 2.0 Hz, 1H), 4.34 (s, 2H), 4.20 – 4.10 (m, 2H), 3.74 (q, *J* = 7.2 Hz, 1H), 2.47 (td, *J* = 6.8, 2.7 Hz, 2H), 1.95 (t, *J* = 2.7 Hz, 1H), 1.49 (d, *J* = 7.2 Hz, 3H).

^{13}C NMR (100 MHz, $CDCl_3$) δ 191.2, 173.5, 142.4, 140.1, 137.8, 136.1, 133.2, 132.4, 131.4, 131.4, 130.8, 128.6, 126.8, 126.3, 79.7, 70.0, 62.4, 51.0, 45.0, 18.8, 18.3.

HRMS (ES+) calcd for $C_{21}H_{18}O_3SNa^+$ $[M+Na]^+$: 373.0869, found: 373.0880.

All the allenes were synthesized according to the literature procedures and purified by flash chromatography. Spectral data for the known allenes **1a**,[14] **1d**,[15] **1e**,[16] **1f**,[17] **1g**,[18] **1h**,[19] **1j**,[20] **1k**,[18] **1l**,[21] and **1m–1n**[22] are in agreement with the literature data. The preparation procedures and characterization data for new allenes are shown below.

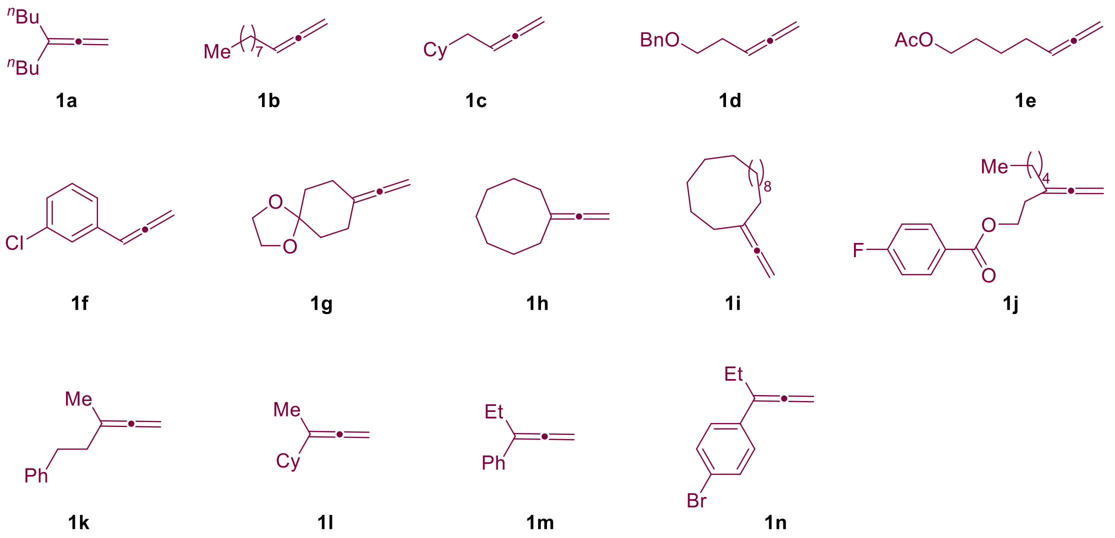

Figure S2. Allenes used in this work.

General Procedure B.

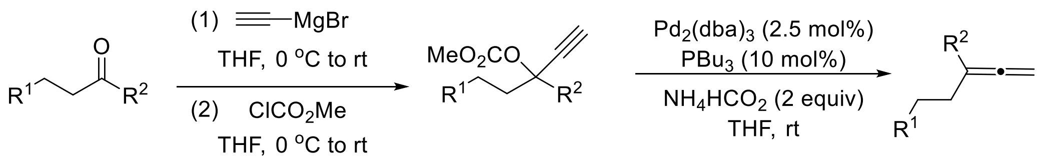

Step 1: At 0 °C under N_2, ethynylmagnesium bromide (0.5 M in THF, 1.1 equiv) was added dropwise to a solution of the corresponding ketone (1.0 equiv) in dry THF (0.2 M). The mixture was stirred at 0 °C for 2 h, at which point the disappearance of the ketone was confirmed by TLC. Then, methyl chloroformate (1.3 equiv) was added. The reaction mixture was allowed to warm to room temperature and stirred for 12 h before it was quenched by water. The aqueous phase was extracted with ethyl acetate. The combined organic layers were dried over $MgSO_4$, filtered, and concentrated. The crude reaction mixture was purified by column chromatography on silica gel (eluent: *n*-

hexane and ethyl acetate) to give the corresponding carbonate.

Step 2: At 0 °C, ^{n}Bu$_3$P (0.2 equiv) was added dropwise to a stirred mixture of carbonate (1.0 equiv), ammonium formate (2.0 equiv) and Pd(dba)$_2$ (0.05 equiv) in THF (0.2 M). The mixture was kept stirring for 12 h. The disappearance of carbonate was confirmed by TLC. Then, the reaction mixture was filtered through a short pad of celite. The filtrate was concentrated, and the residue was purified by column chromatography on silica gel (eluent: *n*-pentane) to give the pure allene.

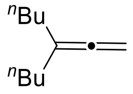

1a

5-Vinylidenenonane (1a) was prepared as a colorless oil from nonan-5-one (4.35 g, 30.0 mmol, 1.0 equiv) according to the General Procedure B (eluent: *n*-hexane, 2.74 g, 60% yield over two steps).

^{1}H NMR (400 MHz, CDCl$_3$) δ 4.63 (p, *J* = 3.2 Hz, 2H), 1.96 – 1.89 (m, 4H), 1.47 – 1.28 (m, 8H), 0.90 (t, *J* = 7.2 Hz, 6H).

^{13}C NMR (100 MHz, CDCl$_3$) δ 205.7, 103.3, 75.1, 31.8, 29.7, 22.5, 14.0.

This is a known compound and the spectral data are consistent with the literature.[14]

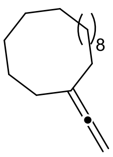

1i

Vinylidenecyclopentadecane (1i) was prepared as a colorless oil from cyclopentadecanone (2.24 g, 10.0 mmol, 1.0 equiv) according to the General Procedure B (eluent: *n*-hexane, 1.29 g, 55% yield over two steps).

^{1}H NMR (400 MHz, CDCl$_3$) δ 4.61 (p, *J* = 2.8 Hz, 2H), 1.98 (tt, *J* = 6.7, 2.9 Hz, 4H), 1.52 – 1.43 (m, 4H), 1.41 – 1.23 (m, 20H).

^{13}C NMR (100 MHz, $CDCl_3$) δ 206.6, 102.4, 74.4, 31.5, 27.1, 27.0, 26.8, 26.6, 26.54, 26.52.

HRMS (CI+) calcd for $C_{17}H_{30}^+$ $[M]^+$: 234.2348, found: 234.2349.

General Procedure C.

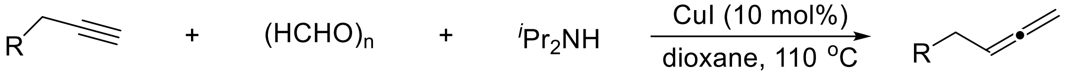

To a 50-mL three-neck round-bottom flask equipped with a magnetic stirring bar were added CuI (189.9 mg, 1.0 mmol, 10 mmol%), paraformaldehyde (481.2 mg, 16.0 mmol, 1.6 equiv), and dioxane (10 mL). The resulting mixture was stirred at room temperature before the addition of ^{i}Pr_2NH (1.98 mL, 1.42 g, 14.0 mmol, 1.4 equiv), the terminal alkyne (10.0 mmol, 1.0 equiv), and dioxane (5 mL). The mixture was stirred at 110 °C for 10 h (in air) before it was cooled to room temperature and filtered through a short column of silica gel with Et_2O as eluent. The filtrate was concentrated. The residue was diluted with Et_2O and diluted again to remove the precipitate. The filtrate was concentrated again and the crude product was purified by flash chromatography on silica gel.

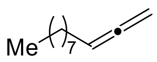

1b

Undeca-1,2-diene (1b) was prepared as a colorless oil from dec-1-yne (1.38 g, 10.0 mmol, 1.0 equiv) according to the General Procedure C (eluent: *n*-hexane, 1.03 g, 68% yield).

^{1}H NMR (400 MHz, $CDCl_3$) δ 5.09 (p, *J* = 6.8 Hz, 1H), 4.64 (dt, *J* = 6.5, 3.2 Hz, 2H), 2.04 – 1.95 (m, 2H), 1.41 (q, *J* = 7.3 Hz, 2H), 1.36 – 1.25 (m, 10H), 0.95 – 0.80 (m, 3H).

^{13}C NMR (100 MHz, $CDCl_3$) δ 208.5, 90.1, 74.4, 31.9, 29.4, 29.3, 29.2, 29.1, 28.3, 22.7, 14.1.

HRMS (CI+) calcd for $C_{11}H_{20}^+$ $[M]^+$: 152.1565, found: 152.1560.

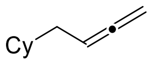

1c

Buta-2,3-dien-1-ylcyclohexane (1c) was prepared as a colorless oil from prop-2-yn-1-ylcyclohexane (1.22 g, 10.0 mmol, 1.0 equiv) according to the General Procedure C (eluent: *n*-hexane, 884.7 mg, 65% yield).

^{1}H NMR (400 MHz, $CDCl_3$) δ 5.05 (p, *J* = 7.1 Hz, 1H), 4.62 (dt, *J* = 6.3, 2.9 Hz, 2H), 1.90 (tt, *J* = 6.7, 2.9 Hz, 2H), 1.70 – 1.61 (m, 2H), 1.32 (td, *J* = 7.3, 3.6 Hz, 1H), 1.25 – 1.17 (m, 2H), 1.09 (d, *J* = 2.7 Hz, 4H), 0.92 (tt, *J* = 12.5, 6.4 Hz, 2H).

^{13}C NMR (100 MHz, $CDCl_3$) δ 208.9, 88.4, 73.8, 38.1, 36.4, 33.0, 26.6, 26.3, 18.4, 11.0.

HRMS (CI+) calcd for $C_{10}H_{15}^+$ [M-H]$^+$:135.1175, found: 135.1176.

III. Ru-Catalyzed Regioselective [2+2] Cycloaddition

Table S1. Evaluation of Catalysts and Solvents[a]

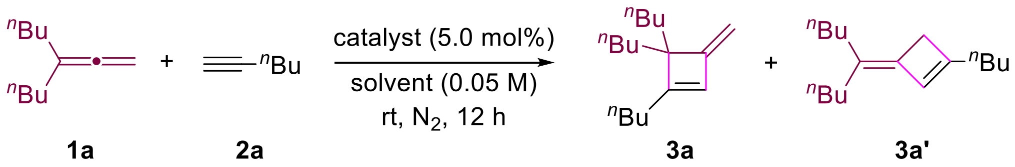

entry	catalyst	solvent	conv (%)	yield (%)	**3a/3a'**[b]
1	$Cp^*Ru(MeCN)_3PF_6$	THF	>99	36	>20:1
2	$CpRu(MeCN)_3PF_6$	THF	>99	78	>20:1
3	$[Cp^*RuCl]_4$	THF	67	0	–
4	$Ru(PPh_3)_3Cl_2$	THF	24	0	–
5	CpRu(COD)Cl	THF	46	0	–
6	Cp*Ru(COD)Cl	THF	68	0	–
7	$CpRu(MeCN)_3PF_6$	EtOAc	>99	28	>20:1
8	$CpRu(MeCN)_3PF_6$	DCM	38	0	–
9	$CpRu(MeCN)_3PF_6$	DMF	>99	46	7:4
10	$CpRu(MeCN)_3PF_6$	PhCl	>99	20	4:1
11	$CpRu(MeCN)_3PF_6$	MeCN	>99	68	>20:1
12	$CpRu(MeCN)_3PF_6$	DCE	>99	0	–
13	$CpRu(MeCN)_3PF_6$	$CHCl_3$	58	21	>20:1
14	$CpRu(MeCN)_3PF_6$	^{t}BuOMe	60	36	>20:1
15	$CpRu(MeCN)_3PF_6$	DMSO	>99	0	–
16[c]	$CpRu(MeCN)_3PF_6$	THF	>99	89	>20:1
17[d]	$CpRu(MeCN)_3PF_6$	THF	>99	78	>20:1
18[c,e]	$CpRu(MeCN)_3PF_6$	THF	74	24	>20:1
19	–	THF	0	–	–

[a]Reactions were carried out with **1a** (0.05 mmol), **2a** (0.07 mmol), catalyst (5.0 mol% based on metal), solvent (1.0 mL), 12 h at room temperature. [b]Determined by ^{1}H NMR analysis of unpurified mixtures with CH_2Br_2 as an internal standard. [c]0.1 M, 6 h. [d]0.2 M, 6 h. [e]0 °C.

General Procedure D.

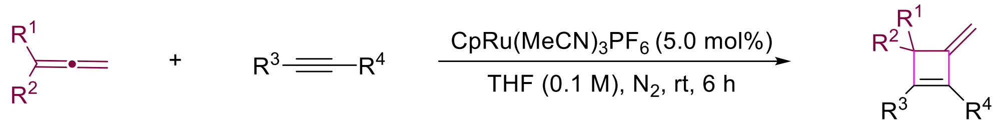

1 2 3 – 4

In a glove box, to an oven-dried 4-mL vial charged with the allene **1** (0.3 mmol, 1.0 equiv), the alkyne (0.42 mmol, 1.4 equiv), and THF (3.0 mL) was added [CpRu(MeCN)$_3$]PF$_6$ (6.5 mg, 15.0 µmol, 5.0 mol%). The vial was capped and removed from the glove box. The reaction mixture was stirred at room temperature for 6–12 h, and then Et$_3$N (~20 mg, 3 drops) was added. The mixture was filtered through a short pad of silica gel. The filtrate was concentrated *in vacuo*, and the residue was purified by silica gel flash column chromatography (Silicycle P60 silica (230-400 mesh, 40-63 µm)) to give the desired product.

General Procedure E.

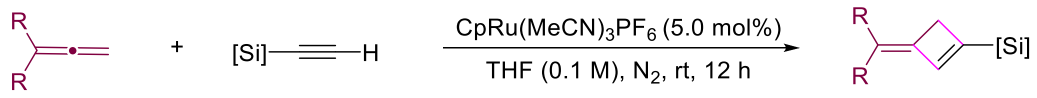

1 2 5

In a glove box, to an oven-dried 4-mL vial charged with allene **1** (0.30 mmol, 1.0 equiv), the alkyne (0.36 mmol, 1.2 equiv), and THF (3.0 mL) was added [CpRu(MeCN)$_3$]PF$_6$ (6.5 mg, 15.0 µmol, 5.0 mol%). The vial was capped and removed from the glove box. The reaction mixture was stirred at room temperature for 12 h, and then Et$_3$N (~20 mg, 3 drops) was added. The mixture was filtered through a short pad of silica gel. The filtrate was concentrated *in vacuo*, and the residue was purified by silica gel flash column chromatography to give the desired product.

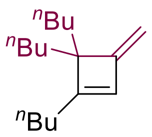

3a

1,4,4-Tributyl-3-methylenecyclobut-1-ene (3a) was prepared as a yellow oil from 5-vinylidenenonane **1a** (45.6 mg, 0.3 mmol, 1.0 equiv), hex-1-yne **2a** (34.5 mg, 0.42 mmol, 1.4 equiv), THF (3.0 mL) and $[CpRu(MeCN)_3]PF_6$ (6.5 mg, 15.0 μmol, 5.0 mol%) at room temperature for 6 h according to the General Procedure D (eluent: *n*-hexane, 59.7 mg, 85% yield).

^{1}H NMR (400 MHz, $CDCl_3$) δ 5.98 (s, 1H), 4.38 (s, 1H), 4.30 (s, 1H), 2.04 (t, *J* = 7.6 Hz, 2H), 1.54 – 1.27 (m, 16H), 0.94 – 0.86 (m, 9H).

^{13}C NMR (100 MHz, $CDCl_3$) δ 165.0, 154.5, 127.7, 91.5, 57.8, 34.7, 28.1, 27.5, 27.4, 23.5, 22.7, 14.1, 13.9.

HRMS (CI+) calcd for $C_{17}H_{30}^+$ $[M]^+$: 234.2348, found: 234.2349.

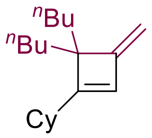

3b

(4,4-Dibutyl-3-methylenecyclobut-1-en-1-yl)cyclohexane (3b) was prepared as a yellow oil from 5-vinylidenenonane **1a** (45.6 mg, 0.3 mmol, 1.0 equiv), ethynylcyclohexane **2b** (45.4 mg, 0.42 mmol, 1.4 equiv), THF (3.0 mL) and $[CpRu(MeCN)_3]PF_6$ (6.5 mg, 15.0 μmol, 5.0 mol%) at room temperature for 6 h according to the General Procedure D (eluent: *n*-hexane, 71.8 mg, 92% yield).

^{1}H NMR (400 MHz, $CDCl_3$) δ 5.97 (s, 1H), 4.39 (s, 1H), 4.30 (s, 1H), 2.10 – 2.01 (m, 1H), 1.89 – 1.56 (m, 8H), 1.50 – 1.43 (m, 2H), 1.34 – 1.21 (m, 12H), 0.88 (t, *J* = 7.0 Hz, 6H).

^{13}C NMR (100 MHz, $CDCl_3$) δ 168.5, 154.3, 127.1, 91.8, 58.7, 38.0, 35.6, 30.8, 27.5, 26.21, 26.18, 23.5, 14.1.

HRMS (CI+) calcd for $C_{19}H_{32}^+$ $[M]^+$: 260.2504, found: 250.2509.

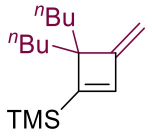

3c

(4,4-Dibutyl-3-methylenecyclobut-1-en-1-yl)trimethylsilane (3c) was prepared as a colorless oil from 5-vinylidenenonane **1a** (45.6 mg, 0.3 mmol, 1.0 equiv), ethynyltrimethylsilane **2c** (41.2 mg, 0.42 mmol, 1.4 equiv), THF (3.0 mL) and $[CpRu(MeCN)_3]PF_6$ (6.5 mg, 15.0 μmol, 5.0 mol%) at room temperature for 6 h according to the General Procedure D (eluent: *n*-hexane, 67.6 mg, 90% yield).

^{1}H NMR (400 MHz, $CDCl_3$) δ 6.68 (s, 1H), 4.45 (s, 1H), 4.35 (s, 1H), 1.62 – 1.55 (m, 2H), 1.52 – 1.44 (m, 2H), 1.35 – 1.23 (m, 8H), 0.88 (t, *J* = 7.0 Hz, 6H), 0.10 (s, 9H).

^{13}C NMR (100 MHz, $CDCl_3$) δ 167.8, 156.5, 145.3, 94.0, 61.2, 36.7, 27.8, 23.5, 14.1, -1.3.

HRMS (CI+) calcd for $C_{16}H_{30}Si^+$ $[M]^+$: 250.2117, found: 250.2121.

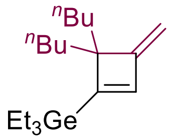

3d

(4,4-Dibutyl-3-methylenecyclobut-1-en-1-yl)triethylgermane (3d) was prepared as a colorless oil from 5-vinylidenenonane **1a** (45.6 mg, 0.3 mmol, 1.0 equiv), triethyl(ethynyl)germane **2d** (78.2 mg, 0.42 mmol, 1.4 equiv), THF (3.0 mL) and $[CpRu(MeCN)_3]PF_6$ (6.5 mg, 15.0 μmol, 5.0 mol%) at 0 °C for 18 h according to the General Procedure D (eluent: *n*-hexane, 85.2 mg, 84% yield, rr = 15:1).

¹H NMR (400 MHz, $CDCl_3$) δ 6.66 (s, 1H), 4.38 (s, 1H), 4.29 (d, *J* = 0.6 Hz, 1H), 1.65 – 1.53 (m, 2H), 1.50 – 1.41 (m, 2H), 1.36 – 1.24 (m, 8H), 1.07 – 1.04 (m, 6H), 0.94 – 0.80 (m, 15H).

¹³C NMR (100 MHz, $CDCl_3$) δ 168.0, 156.5, 145.6, 92.4, 61.8, 36.7, 27.9, 23.6, 14.1, 8.9, 4.4.

HRMS (CI+) calcd for $C_{19}H_{36}Ge^+$ $[M]^+$: 338.2029, found: 338.2027.

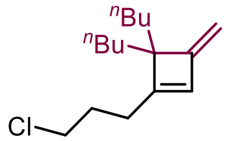

3e

4,4-Dibutyl-1-(3-chloropropyl)-3-methylenecyclobut-1-ene (3e) was prepared as a colorless oil from 5-vinylidenenonane **1a** (45.6 mg, 0.3 mmol, 1.0 equiv), 5-chloropent-1-yne **2e** (42.9 mg, 0.42 mmol, 1.4 equiv), THF (3.0 mL) and $[CpRu(MeCN)_3]PF_6$ (6.5 mg, 15.0 μmol, 5.0 mol%) at room temperature for 6 h according to the General Procedure D (eluent: *n*-hexane, 60.2 mg, 79% yield).

¹H NMR (400 MHz, $CDCl_3$) δ 6.02 (t, *J* = 1.8 Hz, 1H), 4.42 (s, 1H), 4.34 (s, 1H), 3.60 (t, *J* = 6.5 Hz, 2H), 2.25 – 2.20 (m, 2H), 2.03 – 1.96 (m, 2H), 1.59 – 1.52 (m, 2H), 1.47 – 1.38 (m, 2H), 1.32 – 1.17 (m, 8H), 0.88 (t, *J* = 7.1 Hz, 6H).

¹³C NMR (100 MHz, $CDCl_3$) δ 162.8, 154.0, 128.2, 92.4, 57.9, 44.6, 34.6, 29.0, 27.4, 25.0, 23.5, 14.1.

HRMS (CI+) calcd for $C_{16}H_{27}Cl^+$ $[M]^+$: 254.1801, found: 254.1803.

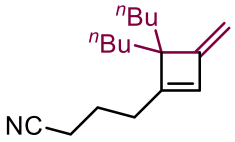

3f

4-(4,4-Dibutyl-3-methylenecyclobut-1-en-1-yl)butanenitrile (3f) was prepared as a colorless oil from 5-vinylidenenonane **1a** (45.6 mg, 0.3 mmol, 1.0

equiv), hex-5-ynenitrile **2f** (39.1 mg, 0.42 mmol, 1.4 equiv), THF (3.0 mL) and $[CpRu(MeCN)_3]PF_6$ (6.5 mg, 15.0 μmol, 5.0 mol%) at room temperature for 6 h according to the General Procedure D (eluent: *n*-hexane, 52.2 mg, 71% yield).

^{1}H NMR (400 MHz, $CDCl_3$) δ 6.02 (s, 1H), 4.44 (s, 1H), 4.36 (s, 1H), 2.43 (t, *J* = 7.1 Hz, 2H), 2.25 – 2.18 (m, 2H), 1.94 – 1.85 (m, 2H), 1.59 – 1.51 (m, 2H), 1.45 – 1.38 (m, 2H), 1.29 – 1.13 (m, 8H), 0.87 (t, *J* = 7.0 Hz, 6H).

^{13}C NMR (100 MHz, $CDCl_3$) δ 161.7, 153.6, 128.6, 119.3, 93.0, 58.0, 34.6, 27.4, 26.6, 23.4, 22.0, 16.9, 14.0.

HRMS (CI+) calcd for $C_{17}H_{27}N^+$ $[M]^+$: 245.2143, found:245.2138.

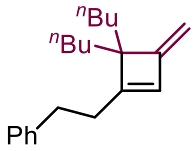

3g

(2-(4,4-Dibutyl-3-methylenecyclobut-1-en-1-yl)ethyl)benzene (3g) was prepared as a colorless oil from 5-vinylidenenonane **1a** (45.6 mg, 0.3 mmol, 1.0 equiv), but-3-yn-1-ylbenzene **2g** (53.6 mg, 0.42 mmol, 1.4 equiv), THF (3.0 mL) and $[CpRu(MeCN)_3]PF_6$ (6.5 mg, 15.0 μmol, 5.0 mol%) at room temperature for 6 h according to the General Procedure D (eluent: *n*-hexane, 67.7 mg, 80% yield).

^{1}H NMR (400 MHz, $CDCl_3$) δ 7.32 – 7.26 (m, 2H), 7.24 – 7.16 (m, 3H), 6.05 (s, 1H), 4.42 (s, 1H), 4.33 (s, 1H), 2.83 (t, *J* = 7.6 Hz, 2H), 2.41 – 2.34 (m, 2H), 1.59 – 1.51 (m, 2H), 1.47 – 1.38 (m, 2H), 1.33 – 1.14 (m, 8H), 0.87 (t, *J* = 7.1 Hz, 6H).

^{13}C NMR (100 MHz, $CDCl_3$) δ 163.8, 154.2, 141.8, 128.4, 128.2 (2C), 125.9, 92.2, 58.0, 34.7, 32.2, 29.6, 27.5, 23.5, 14.1.

HRMS (CI+) calcd for $C_{21}H_{30}^+$ $[M]^+$: 282.2348, found: 282.2349.

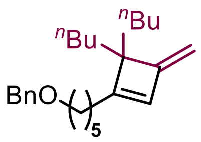

3h

(((6-(4,4-Dibutyl-3-methylenecyclobut-1-en-1-yl)hexyl)oxy)methyl)benzene (3h) was prepared as a colorless oil from 5-vinylidenenonane **1a** (45.6 mg, 0.3 mmol, 1.0 equiv), ((hept-6-yn-1-yloxy)methyl)benzene **2h** (84.6 mg, 0.42 mmol, 1.4 equiv), THF (3.0 mL) and [CpRu(MeCN)$_3$]PF$_6$ (6.5 mg, 15.0 µmol, 5.0 mol%) at room temperature for 6 h according to the General Procedure D (eluent: *n*-hexane/ethyl acetate = 20:1, 76.5 mg, 72% yield).

^{1}H NMR (400 MHz, CDCl$_3$) δ 7.35 (d, *J* = 4.4 Hz, 4H), 7.32 – 7.27 (m, 1H), 5.99 (s, 1H), 4.51 (s, 2H), 4.39 (s, 1H), 4.31 (s, 1H), 3.48 (t, *J* = 6.6 Hz, 2H), 2.08 – 2.02 (m, 2H), 1.67 – 1.43 (m, 10H), 1.31 – 1.17 (m, 8H), 0.89 (d, *J* = 6.9 Hz, 6H).

^{13}C NMR (100 MHz, CDCl$_3$) δ 164.7, 154.4, 138.6, 128.3, 127.7, 127.6, 127.5, 91.6, 72.9, 70.3, 57.8, 34.7, 29.6, 27.7, 27.4, 26.2, 25.7, 23.5, 14.1.

HRMS (CI+) calcd for $C_{25}H_{38}O^+$ [M]$^+$: 354.2923, found: 354.2921.

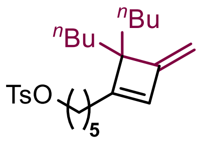

3i

5-(4,4-Dibutyl-3-methylenecyclobut-1-en-1-yl)pentyl 4-methylbenzene sulfonate (3i) was prepared as a colorless oil from 5-vinylidenenonane **1a** (45.6 mg, 0.3 mmol, 1.0 equiv), hept-6-yn-1-yl 4-methylbenzenesulfonate **2i** (111.7 mg, 0.42 mmol, 1.4 equiv), THF (3.0 mL) and [CpRu(MeCN)$_3$]PF$_6$ (6.5 mg, 15.0 µmol, 5.0 mol%) at room temperature for 6 h according to the General Procedure D (eluent: *n*-hexane/ethyl acetate = 10:1, 112.9 mg, 90% yield).

^{1}H NMR (400 MHz, CDCl$_3$) δ 7.79 (d, *J* = 8.3 Hz, 2H), 7.34 (d, *J* = 8.2 Hz, 2H), 5.93 (s, 1H), 4.38 (s, 1H), 4.30 (s, 1H), 4.03 (t, *J* = 6.5 Hz, 2H), 2.45 (s, 3H), 2.03 –

1.96 (m, 2H), 1.72 – 1.64 (m, 2H), 1.55 – 1.36 (m, 8H), 1.29 – 1.13 (m, 8H), 0.87 (t, *J* = 7.0 Hz, 6H).

^{13}C NMR (100 MHz, $CDCl_3$) δ 164.2, 154.2, 144.7, 133.1, 129.8, 127.9, 127.8, 91.9, 70.4, 57.8, 34.6, 28.7, 27.5, 27.4, 25.3, 25.2, 23.5, 21.6, 14.1.

HRMS (ES+) calcd for $C_{25}H_{39}O_3S^+$ $[M+H]^+$: 419.2614, found: 419.2622.

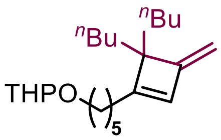

3j

2-((5-(4,4-Dibutyl-3-methylenecyclobut-1-en-1-yl)pentyl)oxy)tetrahydro-2H-pyran (3j) was prepared as a colorless oil from 5-vinylidenenonane **1a** (45.6 mg, 0.3 mmol, 1.0 equiv), 2-(hept-6-yn-1-yloxy)tetrahydro-2H-pyran **2j** (82.3 mg, 0.42 mmol, 1.4 equiv), THF (3.0 mL) and $[CpRu(MeCN)_3]PF_6$ (6.5 mg, 15.0 μmol, 5.0 mol%) at room temperature for 6 h according to the General Procedure D (eluent: *n*-hexane/ethyl acetate = 10:1, 76.2 mg, 73% yield).

^{1}H NMR (400 MHz, $CDCl_3$) δ 5.98 (t, *J* = 1.9 Hz, 1H), 4.57 (dd, *J* = 4.5, 2.7 Hz, 1H), 4.38 (s, 1H), 4.29 (s, 1H), 3.89 – 3.83 (m, 1H), 3.77 – 3.71 (m, 1H), 3.53 – 3.46 (m, 1H), 3.42 – 3.35 (m, 1H), 2.07 – 2.02 (m, 2H), 1.87 – 1.52 (m, 12H), 1.46 – 1.21 (m, 12H), 0.87 (t, *J* = 7.1 Hz, 6H).

^{13}C NMR (100 MHz, $CDCl_3$) δ 164.7, 154.4, 127.7, 98.8, 91.6, 67.5, 62.3, 57.8, 34.7, 30.8, 29.6, 27.7, 27.4, 26.3, 25.7, 25.5, 23.5, 19.7, 14.1.

HRMS (CI+) calcd for $C_{23}H_{40}O_2^+$ $[M]^+$: 348.3028, found: 348.3030.

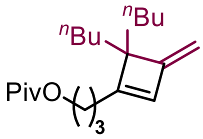

3k

3-(4,4-Dibutyl-3-methylenecyclobut-1-en-1-yl)propyl pivalate (3k) was prepared as a colorless oil from 5-vinylidenenonane **1a** (45.6 mg, 0.3 mmol, 1.0

equiv), pent-4-yn-1-yl pivalate **2k** (70.6 mg, 0.42 mmol, 1.4 equiv), THF (3.0 mL) and $[CpRu(MeCN)_3]PF_6$ (6.5 mg, 15.0 μmol, 5.0 mol%) at room temperature for 6 h according to the General Procedure D (eluent: *n*-hexane/ethyl acetate = 10:1, 79.8 mg, 83% yield).

^{1}H NMR (400 MHz, $CDCl_3$) δ 6.02 (s, 1H), 4.41 (s, 1H), 4.32 (s, 1H), 4.11 (t, *J* = 6.4 Hz, 2H), 2.16 – 2.09 (m, 2H), 1.89 – 1.80 (m, 2H), 1.58 – 1.50 (m, 2H), 1.45 – 1.37 (m, 2H), 1.32 – 1.24 (m, 6H), 1.19 (s, 9H), 1.18 – 1.10 (m, 2H), 0.87 (t, *J* = 7.1 Hz, 6H).

^{13}C NMR (100 MHz, $CDCl_3$) δ 178.5, 163.2, 154.0, 128.1, 92.2, 63.7, 57.8, 38.7, 34.6, 27.4, 27.2, 25.1, 24.1, 23.5, 14.1.

HRMS (CI+) calcd for $C_{21}H_{34}O_4^+$ $[M]^+$: 320.2715, found: 320.2714.

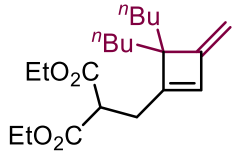

3l

Diethyl 2-((4,4-dibutyl-3-methylenecyclobut-1-en-1-yl)methyl)malonate (3l) was prepared as a colorless oil from 5-vinylidenenonane **1a** (45.6 mg, 0.3 mmol, 1.0 equiv), diethyl 2-(prop-2-yn-1-yl)malonate **2l** (83.2 mg, 0.42 mmol, 1.4 equiv), THF (3.0 mL) and $[CpRu(MeCN)_3]PF_6$ (6.5 mg, 15.0 μmol, 5.0 mol%) at room temperature for 12 h according to the General Procedure D (eluent: *n*-hexane/ethyl acetate = 10:1, 60.9 mg, 58% yield).

^{1}H NMR (400 MHz, $CDCl_3$) δ 5.99 (t, *J* = 1.5 Hz, 1H), 4.42 (s, 1H), 4.34 (s, 1H), 4.20 (q, *J* = 7.1 Hz, 4H), 3.61 (t, *J* = 7.6 Hz, 1H), 2.69 – 2.63 (m, 2H), 1.58 – 1.49 (m, 2H), 1.41 (ddd, *J* = 12.8, 11.0, 4.1 Hz, 2H), 1.28 – 1.22 (m, 12H), 1.20 – 1.10 (m, 2H), 0.86 (t, *J* = 7.1 Hz, 6H).

^{13}C NMR (100 MHz, $CDCl_3$) δ 168.9, 159.9, 153.5, 128.9, 93.1, 61.6, 58.1, 49.3, 34.5, 27.2, 27.0, 23.4, 14.0 (2C).

HRMS (ES+) calcd for $C_{21}H_{34}O_4Na^+$ $[M+Na]^+$: 373.2349, found: 373.2356.

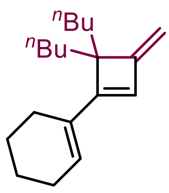

3m

1-(4,4-Dibutyl-3-methylenecyclobut-1-en-1-yl)cyclohex-1-ene (3m) was prepared as a colorless oil from 5-vinylidenenonane **1a** (45.6 mg, 0.3 mmol, 1.0 equiv), 1-ethynylcyclohex-1-ene **2m** (44.5 mg, 0.42 mmol, 1.4 equiv), THF (3.0 mL) and [CpRu(MeCN)$_3$]PF$_6$ (6.5 mg, 15.0 μmol, 5.0 mol%) at room temperature for 12 h according to the General Procedure D (eluent: *n*-hexane, 40.2 mg, 52% yield).

^{1}H NMR (400 MHz, CDCl$_3$) δ 6.02 (s, 1H), 5.76 (td, *J* = 4.0, 1.9 Hz, 1H), 4.49 (s, 1H), 4.40 (s, 1H), 2.22 – 2.05 (m, 4H), 1.70 – 1.58 (m, 6H), 1.55 – 1.47 (m, 2H), 1.30 – 1.22 (m, 6H), 1.18 – 1.09 (m, 2H), 0.86 (t, *J* = 7.1 Hz, 6H).

^{13}C NMR (100 MHz, CDCl$_3$) δ 158.8, 154.2, 132.4, 127.8, 125.1, 93.0, 58.0, 35.5, 27.2, 25.7, 24.5, 23.4, 22.3, 22.2, 14.1.

HRMS (CI+) calcd for $C_{19}H_{30}^+$ [M]$^+$: 258.2348, found: 258.2353.

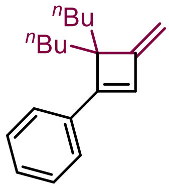

3n

(4,4-Dibutyl-3-methylenecyclobut-1-en-1-yl)benzene (3n) was prepared as a colorless oil from 5-vinylidenenonane **1a** (45.6 mg, 0.3 mmol, 1.0 equiv), ethynylbenzene **2n** (42.8 mg, 0.42 mmol, 1.4 equiv), THF (3.0 mL) and [CpRu(MeCN)$_3$]PF$_6$ (6.5 mg, 15.0 μmol, 5.0 mol%) at room temperature for 6 h according to the General Procedure D (eluent: *n*-hexane, 62.5 mg, 82% yield).

^{1}H NMR (400 MHz, $CDCl_3$) δ 7.39 – 7.31 (m, 4H), 7.29 – 7.23 (m, 1H), 6.56 (s, 1H), 4.64 (s, 1H), 4.55 (s, 1H), 1.83 – 1.66 (m, 4H), 1.37 – 1.14 (m, 8H), 0.82 (t, *J* = 7.2 Hz, 6H).

^{13}C NMR (100 MHz, $CDCl_3$) δ 156.6, 153.6, 134.0, 128.5, 128.2, 127.4, 125.8, 94.5, 58.3, 35.6, 27.3, 23.4, 14.0.

HRMS (CI+) calcd for $C_{19}H_{26}^+$ $[M]^+$: 254.2035, found: 254.2037.

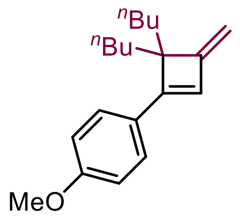

3o

1-(4,4-Dibutyl-3-methylenecyclobut-1-en-1-yl)-4-methoxybenzene (3o) was prepared as a colorless oil from 5-vinylidenenonane **1a** (45.6 mg, 0.3 mmol, 1.0 equiv), 1-ethynyl-4-methoxybenzene **2o** (55.4 mg, 0.42 mmol, 1.4 equiv), THF (3.0 mL) and $[CpRu(MeCN)_3]PF_6$ (6.5 mg, 15.0 μmol, 5.0 mol%) at room temperature for 6 h according to the General Procedure D (eluent: *n*-hexane/ethyl acetate = 50:1, 59.6 mg, 70% yield).

^{1}H NMR (400 MHz, $CDCl_3$) δ 7.35 – 7.30 (m, 2H), 6.91 – 6.86 (m, 2H), 6.43 (s, 1H), 4.57 (s, 1H), 4.49 (s, 1H), 3.83 (s, 3H), 1.81 – 1.63 (m, 4H), 1.37 – 1.12 (m, 8H), 0.82 (t, *J* = 7.1 Hz, 6H).

^{13}C NMR (100 MHz, $CDCl_3$) δ 159.7, 156.3, 153.7, 127.2, 126.9, 125.1, 114.0, 93.2, 58.1, 55.3, 35.6, 27.2, 23.4, 14.0.

HRMS (CI+) calcd for $C_{20}H_{28}O^+$ $[M]^+$: 284.2140, found: 284.2140.

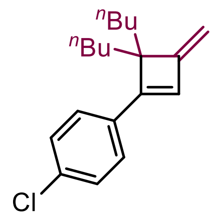

3p

1-Chloro-4-(4,4-dibutyl-3-methylenecyclobut-1-en-1-yl)benzene (3p) was prepared as a colorless oil from 5-vinylidenenonane **1a** (45.6 mg, 0.3 mmol, 1.0 equiv), 1-chloro-4-ethynylbenzene **2p** (57.1 mg, 0.42 mmol, 1.4 equiv), THF (3.0 mL) and [CpRu(MeCN)$_3$]PF$_6$ (6.5 mg, 15.0 μmol, 5.0 mol%) at room temperature for 6 h according to the General Procedure D (eluent: *n*-hexane, 76.0 mg, 88% yield).

^{1}H NMR (400 MHz, CDCl$_3$) δ 7.31 (d, *J* = 1.0 Hz, 4H), 6.56 (s, 1H), 4.67 (s, 1H), 4.58 (s, 1H), 1.83 – 1.75 (m, 2H), 1.71 – 1.63 (m, 2H), 1.38 – 1.23 (m, 6H), 1.18 – 1.06 (m, 2H), 0.83 (t, *J* = 7.2 Hz, 6H).

^{13}C NMR (100 MHz, CDCl$_3$) δ 155.3, 153.2, 133.9, 132.4, 128.8, 128.0, 127.0, 95.1, 58.3, 35.5, 27.2, 23.3, 14.0.

HRMS (CI+) calcd for $C_{19}H_{25}Cl^+$ [M]$^+$: 288.1645, found: 288.1644..

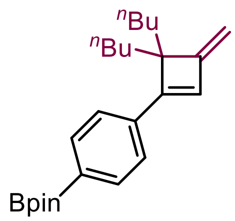

3q

2-(4-(4,4-Dibutyl-3-methylenecyclobut-1-en-1-yl)phenyl)-4,4,5,5-tetramethyl-1,3,2-dioxaborolane (3q) was prepared as a colorless oil from 5-vinylidenenonane **1a** (45.6 mg, 0.3 mmol, 1.0 equiv), 2-(4-ethynylphenyl)-4,4,5,5-tetramethyl-1,3,2-dioxaborolane **2q** (95.8 mg, 0.42 mmol, 1.4 equiv), THF (3.0 mL) and [CpRu(MeCN)$_3$]PF$_6$ (6.5 mg, 15.0 μmol, 5.0 mol%) at room temperature for 6 h according to the General Procedure D (eluent: *n*-

hexane/ethyl acetate = 50:1, 80.9 mg, 71% yield).

^{1}H NMR (400 MHz, $CDCl_3$) δ 7.80 (d, *J* = 7.9 Hz, 2H), 7.38 (d, *J* = 7.8 Hz, 2H), 6.63 (s, 1H), 4.67 (s, 1H), 4.58 (s, 1H), 1.84 – 1.69 (m, 4H), 1.35 (s, 12H), 1.32 – 1.10 (m, 8H), 0.82 (t, *J* = 7.2 Hz, 6H).

^{13}C NMR (100 MHz, $CDCl_3$) δ 156.4, 153.5, 136.4, 134.9, 128.6, 125.0, 95.0, 83.8, 58.3, 35.5, 27.2, 24.8, 23.3, 14.0. The boron-bound carbon was not detected due to quadrupolar relaxation.

HRMS (ES+) calcd for $C_{25}H_{38}BO_2^+$ [M+H]$^+$: 381.2959, found: 381.2956.

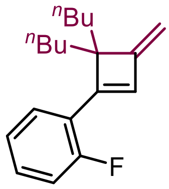

3r

1-(4,4-Dibutyl-3-methylenecyclobut-1-en-1-yl)-2-fluorobenzene (3r) was prepared as a colorless oil from 5-vinylidenenonane **1a** (45.6 mg, 0.3 mmol, 1.0 equiv), 1-ethynyl-2-fluorobenzene **2r** (50.4 mg, 0.42 mmol, 1.4 equiv), THF (3.0 mL) and $[CpRu(MeCN)_3]PF_6$ (6.5 mg, 15.0 μmol, 5.0 mol%) at room temperature for 6 h according to the General Procedure D (eluent: *n*-hexane, 60.4 mg, 74% yield).

^{1}H NMR (400 MHz, $CDCl_3$) δ 7.31 – 7.23 (m, 2H), 7.16 – 7.06 (m, 2H), 6.67 (d, *J* = 4.1 Hz, 1H), 4.71 (s, 1H), 4.61 (s, 1H), 1.86 – 1.69 (m, 4H), 1.42 – 1.15 (m, 8H), 0.86 (t, *J* = 7.2 Hz, 6H).

^{13}C NMR (100 MHz, $CDCl_3$) δ 161.3 (d, *J* = 253.4 Hz), 154.4, 150.4, 133.1 (d, *J* = 9.7 Hz), 129.3 (d, *J* = 8.5 Hz), 127.4 (d, *J* = 4.1 Hz), 124.0 (d, *J* = 3.6 Hz), 122.1 (d, *J* = 14.1 Hz), 115.8 (d, *J* = 21.4 Hz), 95.2, 59.2, 35.5, 27.2, 23.4, 14.0.

^{19}F NMR (376 MHz, $CDCl_3$) δ -111.32.

HRMS (CI+) calcd for $C_{19}H_{25}F^+$ [M]$^+$: 272.1940, found: 272.1935.

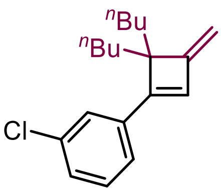

3s

1-Chloro-3-(4,4-dibutyl-3-methylenecyclobut-1-en-1-yl)benzene (3s) was prepared as a colorless oil from 5-vinylidenenonane **1a** (45.6 mg, 0.3 mmol, 1.0 equiv), 1-chloro-3-ethynylbenzene **2s** (57.1 mg, 0.42 mmol, 1.4 equiv), THF (3.0 mL) and [CpRu(MeCN)$_3$]PF$_6$ (6.5 mg, 15.0 μmol, 5.0 mol%) at room temperature for 6 h according to the General Procedure D (eluent: *n*-hexane, 74.3 mg, 86% yield).

^{1}H NMR (400 MHz, CDCl$_3$) δ 7.35 – 7.33 (m, 1H), 7.30 – 7.20 (m, 3H), 6.59 (s, 1H), 4.68 (s, 1H), 4.59 (s, 1H), 1.82 – 1.64 (m, 4H), 1.37 – 1.22 (m, 6H), 1.17 – 1.07 (m, 2H), 0.82 (t, *J* = 7.2 Hz, 6H).

^{13}C NMR (100 MHz, CDCl$_3$) δ 155.1, 153.1, 135.7, 134.5, 129.8, 128.9, 128.2, 125.6, 123.8, 95.7, 58.4, 35.4, 27.2, 23.3, 14.0.

HRMS (CI+) calcd for $C_{19}H_{25}Cl^+$ [M]$^+$: 288.1645, found: 288.1644.

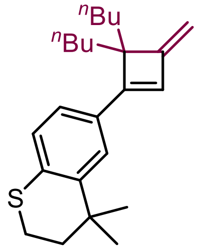

3t

6-(4,4-Dibutyl-3-methylenecyclobut-1-en-1-yl)-4,4-dimethylthiochromane (3t) was prepared as a colorless oil from 5-vinylidenenonane **1a** (45.6 mg, 0.3 mmol, 1.0 equiv), 6-ethynyl-4,4-dimethylthiochromane **2t** (84.9 mg, 0.42 mmol, 1.4 equiv), THF (3.0 mL) and [CpRu(MeCN)$_3$]PF$_6$ (13.0 mg, 30.0 μmol, 10.0 mol%) at room temperature for 12 h according to the General Procedure D (eluent: *n*-hexane, 51.0 mg, 48% yield, rr = 13:1).

^{1}**H NMR** (400 MHz, $CDCl_3$) δ 7.35 (d, *J* = 1.6 Hz, 1H), 7.09 – 7.03 (m, 2H), 6.47 (s, 1H), 4.59 (s, 1H), 4.51 (s, 1H), 3.06 – 3.01 (m, 2H), 1.99 – 1.95 (m, 2H), 1.82 – 1.64 (m, 4H), 1.34 (s, 6H), 1.31 – 1.09 (m, 8H), 0.83 (t, *J* = 7.2 Hz, 6H).

^{13}**C NMR** (100 MHz, $CDCl_3$) δ 156.6, 153.7, 141.9, 132.6, 129.9, 126.6, 125.9, 123.9, 123.5, 93.6, 58.1, 37.4, 35.7, 32.9, 30.2, 27.2, 23.4, 23.2, 14.0.

HRMS (CI+) calcd for $C_{24}H_{34}S^+$ $[M]^+$: 354.2381, found: 354.2381.

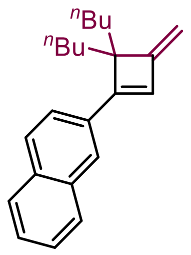

3u

2-(4,4-Dibutyl-3-methylenecyclobut-1-en-1-yl)naphthalene (3u) was prepared as a colorless oil from 5-vinylidenenonane **1a** (45.6 mg, 0.3 mmol, 1.0 equiv), 2-ethynylnaphthalene **2u** (63.8 mg, 0.42 mmol, 1.4 equiv), THF (3.0 mL) and $[CpRu(MeCN)_3]PF_6$ (6.5 mg, 15.0 μmol, 5.0 mol%) at room temperature for 12 h according to the General Procedure D (eluent: *n*-hexane, 68.4 mg, 75% yield).

^{1}**H NMR** (400 MHz, $CDCl_3$) δ 7.89 – 7.78 (m, 3H), 7.74 (s, 1H), 7.58 (dd, *J* = 8.6, 1.7 Hz, 1H), 7.54 – 7.43 (m, 2H), 6.70 (s, 1H), 4.71 (s, 1H), 4.62 (s, 1H), 1.94 – 1.78 (m, 4H), 1.43 – 1.19 (m, 8H), 0.84 (t, *J* = 7.1 Hz, 6H).

^{13}**C NMR** (100 MHz, $CDCl_3$) δ 156.5, 153.6, 133.4, 133.2, 131.5, 128.4, 128.14, 128.11, 127.8, 126.34, 126.30, 124.7, 123.8, 94.8, 58.4, 35.7, 27.3, 23.4, 14.0.

HRMS (CI+) calcd for $C_{23}H_{28}^+$ $[M]^+$: 304.2191, found: 304.2191.

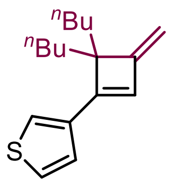

3v

3-(4,4-Dibutyl-3-methylenecyclobut-1-en-1-yl)thiophene (3v) was prepared as a colorless oil from 5-vinylidenenonane **1a** (45.6 mg, 0.3 mmol, 1.0 equiv), 3-ethynylthiophene **2v** (45.4 mg, 0.42 mmol, 1.4 equiv), THF (3.0 mL) and [CpRu(MeCN)$_3$]PF$_6$ (6.5 mg, 15.0 μmol, 5.0 mol%) at room temperature for 12 h according to the General Procedure D (eluent: *n*-hexane, 57.7 mg, 74% yield).

^{1}H NMR (400 MHz, CDCl$_3$) δ 7.30 (dd, *J* = 5.0, 2.9 Hz, 1H), 7.22 (dd, *J* = 2.9, 1.3 Hz, 1H), 7.19 (dd, *J* = 5.0, 1.2 Hz, 1H), 6.33 (s, 1H), 4.63 (s, 1H), 4.54 (s, 1H), 1.80 – 1.61 (m, 4H), 1.38 – 1.17 (m, 8H), 0.84 (t, *J* = 7.1 Hz, 6H).

^{13}C NMR (100 MHz, CDCl$_3$) δ 153.9, 152.3, 135.8, 126.1, 125.74, 125.71, 121.9, 94.6, 58.4, 35.4, 27.2, 23.4, 14.0.

HRMS (ES+) calcd for $C_{17}H_{25}S^+$ [M+H]$^+$: 261.1671, found: 261.1676.

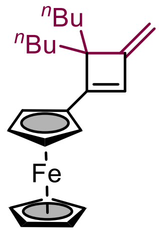

3w

2-(4,4-Dibutyl-3-methylenecyclobut-1-en-1-yl)ferrocene (3w) was prepared as a colorless oil from 5-vinylidenenonane **1a** (45.6 mg, 0.3 mmol, 1.0 equiv), ethynylferrocene **2w** (91.0 mg, 0.42 mmol, 1.4 equiv), THF (3.0 mL) and [CpRu(MeCN)$_3$]PF$_6$ (6.5 mg, 15.0 μmol, 5.0 mol%) at room temperature for 12 h according to the General Procedure D (eluent: *n*-hexane, 72.5 mg, 65% yield, rr = 16:1).

^{1}H NMR (400 MHz, $CDCl_3$) δ 6.21 (s, 1H), 4.49 (s, 1H), 4.43 (s, 1H), 4.36 – 4.33 (m, 2H), 4.31 – 4.29 (m, 2H), 4.13 (s, 5H), 1.80 – 1.72 (m, 2H), 1.63 – 1.56 (m, 2H), 1.43 – 1.28 (m, 8H), 0.89 (t, J = 7.0 Hz, 6H).

^{13}C NMR (100 MHz, $CDCl_3$) δ 159.3, 154.9, 125.5, 92.5, 69.5, 69.3, 69.0, 66.9, 58.6, 35.8, 27.7, 23.6, 14.1.

HRMS (ES+) calcd for $C_{19}H_{31}BNaO_2Si^+$ $[M]^+$: 362.1697, found: 362.1702.

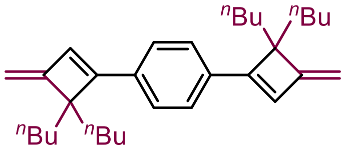

3x

1,4-Bis(4,4-dibutyl-3-methylenecyclobut-1-en-1-yl)benzene (3x) was prepared as a colorless oil from 5-vinylidenenonane **1a** (109.6 mg, 0.72 mmol, 2.4 equiv), 1,4-diethynylbenzene **2x** (37.8 mg, 0.3 mmol, 1.0 equiv), THF (3.0 mL) and $[CpRu(MeCN)_3]PF_6$ (6.5 mg, 15.0 μmol, 5.0 mol%) at room temperature for 12 h according to the General Procedure D (eluent: *n*-hexane, 89.0 mg, 69% yield).

^{1}H NMR (400 MHz, $CDCl_3$) δ 7.35 (s, 4H), 6.59 (s, 2H), 4.67 (s, 2H), 4.58 (d, J = 0.8 Hz, 2H), 1.85 – 1.67 (m, 8H), 1.37 – 1.18 (m, 16H), 0.84 (t, J = 7.1 Hz, 12H).

^{13}C NMR (100 MHz, $CDCl_3$) δ 156.2, 153.6, 133.7, 127.9, 126.0, 95.0, 58.3, 35.6, 27.3, 23.4, 14.0.

HRMS (ES+) calcd for $C_{32}H_{46}^+$ $[M]^+$: 430.3600, found: 430.3604.

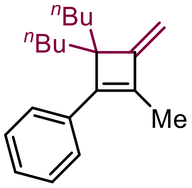

3y

(4,4-Dibutyl-2-methyl-3-methylenecyclobut-1-en-1-yl)benzene (3y) was prepared as a colorless oil from 5-vinylidenenonane **1a** (45.6 mg, 0.3 mmol, 1.0

equiv), prop-1-yn-1-ylbenzene **2y** (48.7 mg, 0.42 mmol, 1.4 equiv), THF (3.0 mL) and $[CpRu(MeCN)_3]PF_6$ (6.5 mg, 15.0 μmol, 5.0 mol%) at room temperature for 6 h according to the General Procedure D (eluent: *n*-hexane, 58.7 mg, 73% yield).

^{1}H NMR (400 MHz, $CDCl_3$) δ 7.44 – 7.35 (m, 4H), 7.29 – 7.23 (m, 1H), 4.64 (s, 1H), 4.55 (s, 1H), 2.03 (s, 3H), 1.85 – 1.67 (m, 4H), 1.37 – 1.16 (m, 8H), 0.85 (t, *J* = 7.1 Hz, 6H).

^{13}C NMR (100 MHz, $CDCl_3$) δ 156.4, 149.2, 138.4, 135.5, 128.5, 127.1, 126.5, 91.6, 57.0, 35.9, 27.3, 23.4, 14.0, 10.5.

HRMS (CI+) calcd for $C_{20}H_{28}^+$ $[M]^+$: 268.2191, found: 268.2193.

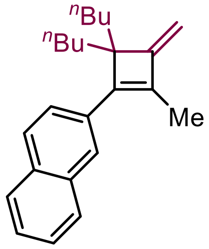

3z

2-(4,4-Dibutyl-2-methyl-3-methylenecyclobut-1-en-1-yl)naphthalene (3z) was prepared as a colorless oil from 5-vinylidenenonane **1a** (45.6 mg, 0.3 mmol, 1.0 equiv), 2-(prop-1-yn-1-yl)naphthalene **2z** (69.7 mg, 0.42 mmol, 1.4 equiv), THF (3.0 mL) and $[CpRu(MeCN)_3]PF_6$ (6.5 mg, 15.0 μmol, 5.0 mol%) at room temperature for 12 h according to the General Procedure D (eluent: *n*-hexane, 60.1 mg, 63% yield).

^{1}H NMR (400 MHz, $CDCl_3$) δ 7.87 – 7.75 (m, 4H), 7.61 (dd, *J* = 8.6, 1.7 Hz, 1H), 7.52 – 7.44 (m, 2H), 4.67 (s, 1H), 4.58 (s, 1H), 2.12 (s, 3H), 1.90 – 1.76 (m, 4H), 1.39 – 1.18 (m, 8H), 0.84 (t, *J* = 7.1 Hz, 6H).

^{13}C NMR (100 MHz, $CDCl_3$) δ 156.4, 149.2, 139.0, 133.5, 133.1, 132.4, 128.2, 128.0, 127.7, 126.2, 126.0, 125.1, 124.8, 91.8, 57.1, 36.0, 27.3, 23.4, 14.0, 10.7.

HRMS (CI+) calcd for $C_{24}H_{30}^+$ $[M]^+$: 318.2348, found: 318.2349.

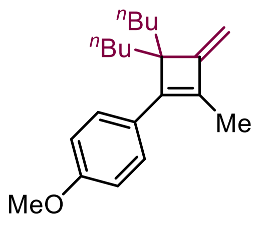

3aa

1-(4,4-Dibutyl-2-methyl-3-methylenecyclobut-1-en-1-yl)-4-methoxybenzene (3aa) was prepared as a colorless oil from 5-vinylidenenonane **1a** (45.6 mg, 0.3 mmol, 1.0 equiv), 1-methoxy-4-(prop-1-yn-1-yl)benzene **2aa** (61.3 mg, 0.42 mmol, 1.4 equiv), THF (3.0 mL) and [CpRu(MeCN)$_3$]PF$_6$ (6.5 mg, 15.0 μmol, 5.0 mol%) at room temperature for 6 h according to the General Procedure D (eluent: *n*-hexane, 57.2 mg, 64% yield).

^{1}H NMR (400 MHz, CDCl$_3$) δ 7.37 – 7.31 (m, 2H), 6.93 – 6.86 (m, 2H), 4.54 (s, 1H), 4.46 (s, 1H), 3.83 (s, 3H), 1.96 (s, 3H), 1.80 – 1.61 (m, 4H), 1.32 – 1.11 (m, 8H), 0.82 (t, *J* = 7.1 Hz, 6H).

^{13}C NMR (100 MHz, CDCl$_3$) δ 158.7, 156.5, 148.9, 136.0, 128.4, 127.9, 114.0, 90.4, 56.8, 55.2, 35.6, 27.3, 23.4, 14.0, 10.4.

HRMS (CI+) calcd for $C_{21}H_{30}O^+$ [M]$^+$: 298.2297, found: 298.2298.

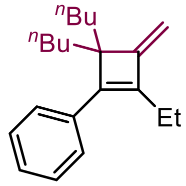

3ab

(4,4-Dibutyl-2-ethyl-3-methylenecyclobut-1-en-1-yl)benzene (3ab) was prepared as a colorless oil from 5-vinylidenenonane **1a** (45.6 mg, 0.3 mmol, 1.0 equiv), but-1-yn-1-ylbenzene **2ab** (54.6 mg, 0.42 mmol, 1.4 equiv), THF (3.0 mL) and [CpRu(MeCN)$_3$]PF$_6$ (6.5 mg, 15.0 μmol, 5.0 mol%) at room temperature for 6 h according to the General Procedure D (eluent: *n*-hexane, 66.8 mg, 79% yield).

^{1}H NMR (400 MHz, $CDCl_3$) δ 7.38 – 7.32 (m, 4H), 7.25 – 7.20 (m, 1H), 4.64 (s, 1H), 4.52 (s, 1H), 2.46 (q, *J* = 7.6 Hz, 2H), 1.79 – 1.65 (m, 4H), 1.26 – 1.11 (m, 11H), 0.81 (t, *J* = 7.1 Hz, 6H).

^{13}C NMR (100 MHz, $CDCl_3$) δ 155.1, 148.0, 144.6, 135.4, 128.5, 127.1, 126.6, 92.0, 56.6, 35.8, 27.1, 23.4, 19.2, 14.0, 12.3.

HRMS (CI+) calcd for $C_{21}H_{30}^+$ [M]$^+$: 282.2348, found: 282.2360.

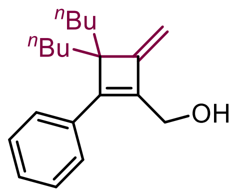

3ac

(3,3-Dibutyl-4-methylene-2-phenylcyclobut-1-en-1-yl)methanol (3ac) was prepared as a colorless oil from 5-vinylidenenonane **1a** (45.6 mg, 0.3 mmol, 1.0 equiv), 3-phenylprop-2-yn-1-ol **2ac** (55.5 mg, 0.42 mmol, 1.4 equiv), THF (3.0 mL) and $[CpRu(MeCN)_3]PF_6$ (6.5 mg, 15.0 μmol, 5.0 mol%) at room temperature for 6 h according to the General Procedure D (eluent: *n*-hexane/ethyl acetate = 20:1, 47.7 mg, 56% yield).

^{1}H NMR (400 MHz, $CDCl_3$) δ 7.44 – 7.33 (m, 4H), 7.31 – 7.25 (m, 1H), 4.80 (s, 1H), 4.60 (s, 1H), 4.53 (s, 2H), 1.85 – 1.66 (m, 4H), 1.62 (br, 1H), 1.36 – 1.13 (m, 8H), 0.82 (t, *J* = 7.1 Hz, 6H).

^{13}C NMR (100 MHz, $CDCl_3$) δ 153.5, 151.7, 140.1, 134.1, 128.6, 128.0, 127.3, 93.6, 57.2, 56.5, 35.6, 27.2, 23.3, 14.0.

HRMS (CI+) calcd for $C_{20}H_{28}O^+$ [M]$^+$: 284.2140, found: 284.2142.

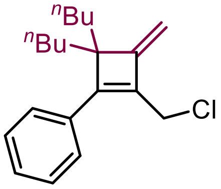

3ad

(4,4-Dibutyl-2-(chloromethyl)-3-methylenecyclobut-1-en-1-yl)benzene (3ad) was prepared as a colorless oil from 5-vinylidenenonane **1a** (45.6 mg, 0.3 mmol, 1.0 equiv), (3-chloroprop-1-yn-1-yl)benzene **2ad** (63.0 mg, 0.42 mmol, 1.4 equiv), THF (3.0 mL) and $[CpRu(MeCN)_3]PF_6$ (6.5 mg, 15.0 μmol, 5.0 mol%) at room temperature for 6 h according to the General Procedure D (eluent: *n*-hexane, 59.8 mg, 66% yield).

^{1}H NMR (400 MHz, $CDCl_3$) δ 7.44 – 7.37 (m, 4H), 7.35 – 7.29 (m, 1H), 4.80 (s, 1H), 4.65 (s, 1H), 4.34 (s, 2H), 1.84 – 1.65 (m, 4H), 1.36 – 1.14 (m, 8H), 0.82 (t, *J* = 7.1 Hz, 6H).

^{13}C NMR (100 MHz, $CDCl_3$) δ 153.8, 152.7, 136.1, 133.7, 128.8, 128.5, 127.3, 93.8, 57.8, 35.7, 35.1, 27.0, 23.3, 13.9.

HRMS (CI+) calcd for $C_{20}H_{27}Cl^+$ $[M]^+$: 302.1801, found: 302.1800.

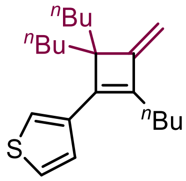

3ae

3-(2,4,4-Tributyl-3-methylenecyclobut-1-en-1-yl)thiophene (3ae) was prepared as a colorless oil from 5-vinylidenenonane **1a** (45.6 mg, 0.3 mmol, 1.0 equiv), (3-chloroprop-1-yn-1-yl)benzene **2ae** (68.9 mg, 0.42 mmol, 1.4 equiv), THF (3.0 mL) and $[CpRu(MeCN)_3]PF_6$ (6.5 mg, 15.0 μmol, 5.0 mol%) at room temperature for 12 h according to the General Procedure D (eluent: *n*-hexane, 81.6 mg, 86% yield).

^{1}H NMR (400 MHz, $CDCl_3$) δ 7.31 (dd, *J* = 5.1, 2.9 Hz, 1H), 7.22 (dd, *J* = 5.0, 1.2 Hz, 1H), 7.19 (dd, *J* = 2.9, 1.2 Hz, 1H), 4.61 (s, 1H), 4.49 (s, 1H), 2.36 (t, *J* = 7.5 Hz, 2H), 1.76 – 1.69 (m, 2H), 1.62 – 1.56 (m, 4H), 1.39 – 1.12 (m, 10H), 0.93 (s, 3H), 0.82 (t, *J* = 7.1 Hz, 6H).

^{13}C NMR (100 MHz, $CDCl_3$) δ 155.5, 144.8, 141.1, 136.2, 126.3, 125.5, 121.5, 92.3, 56.4, 35.7, 30.1, 27.2, 25.6, 23.4, 22.9, 14.02, 13.97.

HRMS (ES+) calcd for $C_{21}H_{32}S^+$ $[M]^+$: 316.2225, found: 316.2221.

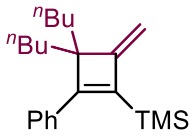

3af

(3,3-Dibutyl-4-methylene-2-phenylcyclobut-1-en-1-yl)trimethylsilane (3af) was prepared as a colorless oil from 5-vinylidenenonane **1a** (45.6 mg, 0.3 mmol, 1.0 equiv), trimethyl(phenylethynyl)silane **2af** (73.1 mg, 0.42 mmol, 1.4 equiv), THF (3.0 mL) and $[CpRu(MeCN)_3]PF_6$ (6.5 mg, 15.0 μmol, 5.0 mol%) at room temperature for 12 h according to the General Procedure D (eluent: *n*-hexane, 85.1 mg, 87% yield).

^{1}H NMR (400 MHz, $CDCl_3$) δ 7.35 (d, *J* = 4.4 Hz, 4H), 7.30 – 7.27 (m, 1H), 4.68 (s, 1H), 4.51 (s, 1H), 1.76 – 1.62 (m, 4H), 1.34 – 1.16 (m, 8H), 0.83 (t, *J* = 7.1 Hz, 6H), 0.26 (s, 9H).

^{13}C NMR (100 MHz, $CDCl_3$) δ 166.6, 157.4, 145.0, 136.0, 128.2, 127.9, 127.0, 94.2, 60.3, 35.6, 27.0, 23.4, 14.0.

HRMS (CI+) calcd for $C_{22}H_{34}Si^+$ $[M]^+$: 326.2430, found: 326.2437.

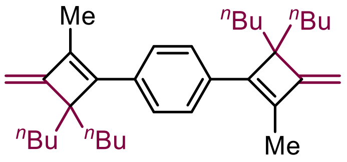

3ag

1,4-Bis(4,4-dibutyl-2-methyl-3-methylenecyclobut-1-en-1-yl)benzene (3ag) was prepared as light yellow solid from 5-vinylidenenonane **1a** (109.6 mg, 0.72 mmol, 2.4 equiv), 1,4-di(prop-1-yn-1-yl)benzene **2ag** (46.2 mg, 0.3 mmol, 1.0 equiv), THF (3.0 mL) and [CpRu(MeCN)$_3$]PF$_6$ (6.5 mg, 15.0 µmol, 5.0 mol%) at room temperature for 12 h according to the General Procedure D (eluent: *n*-hexane, 107.2 mg, 78% yield).

^{1}H NMR (400 MHz, CDCl$_3$) δ 7.38 (s, 4H), 4.63 (s, 2H), 4.55 (s, 2H), 2.03 (s, 6H), 1.84 – 1.66 (m, 8H), 1.28 (dp, *J* = 19.9, 6.4 Hz, 16H), 0.85 (t, *J* = 7.0 Hz, 12H).

^{13}C NMR (100 MHz, CDCl$_3$) δ 156.4, 149.0, 138.7, 134.1, 126.6, 91.9, 57.0, 35.9, 27.4, 23.4, 14.1, 10.7.

HRMS (CI+) calcd for $C_{34}H_{50}^+$ [M]$^+$: 458.3913, found: 458.3914.

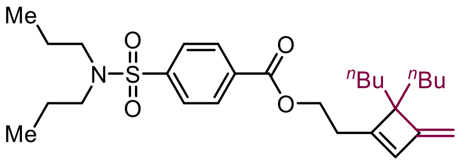

3ah

2-(4,4-Dibutyl-3-methylenecyclobut-1-en-1-yl)ethyl 4-(*N,N*-dipropyl sulfamoyl)benzoate (3ah) was prepared as a colorless oil from 5-vinylidenenonane **1a** (45.6 mg, 0.3 mmol, 1.0 equiv), but-3-yn-1-yl 4-(*N,N*-dipropylsulfamoyl)benzoate **2ah** (141.5 mg, 0.42 mmol, 1.4 equiv), THF (3.0 mL) and [CpRu(MeCN)$_3$]PF$_6$ (6.5 mg, 15.0 µmol, 5.0 mol%) at room temperature for 12 h according to the General Procedure D (eluent: *n*-hexane/ethyl acetate = 10:1, 120.4 mg, 82% yield).

^{1}**H NMR** (400 MHz, $CDCl_3$) δ 8.14 – 8.11 (m, 2H), 7.87 – 7.84 (m, 2H), 6.11 (s, 1H), 4.52 (t, *J* = 6.6 Hz, 2H), 4.44 (s, 1H), 4.36 (s, 1H), 3.08 (t, *J* = 7.7 Hz, 4H), 2.57 – 2.54 (m, 2H), 1.58 – 1.49 (m, 8H), 1.36 – 1.11 (m, 10H), 0.87 – 0.82 (m, 10H).

^{13}**C NMR** (100 MHz, $CDCl_3$) δ 165.1, 159.2, 153.7, 144.2, 133.4, 130.2, 129.5, 127.0, 93.0, 62.5, 58.2, 49.9, 34.6, 27.4, 27.2, 23.4, 21.9, 14.0, 11.1.

HRMS (ES+) calcd for $C_{28}H_{43}NO_4SNa^+$ $[M+Na]^+$: 512.2805, found: 512.2809.

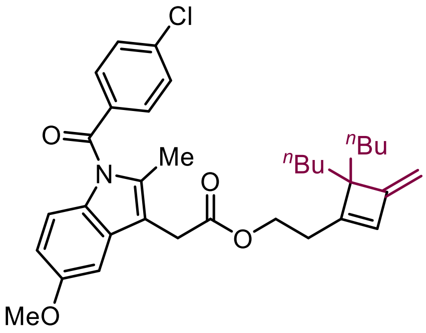

3ai

2-(4,4-Dibutyl-3-methylenecyclobut-1-en-1-yl)ethyl 2-(1-(4-chlorobenzoyl)-5-methoxy-2-methyl-*1H*-indol-3-yl)acetate (3ai) was prepared as a colorless oil from 5-vinylidenenonane **1a** (45.6 mg, 0.3 mmol, 1.0 equiv), but-3-yn-1-yl 2-(1-(4-chlorobenzoyl)-5-methoxy-2-methyl-*1H*-indol-3-yl)acetate **2ai** (171.8 mg, 0.42 mmol, 1.4 equiv), THF (3.0 mL) and $[CpRu(MeCN)_3]PF_6$ (6.5 mg, 15.0 μmol, 5.0 mol%) at room temperature for 12 h according to the General Procedure D (eluent: *n*-hexane/ethyl acetate = 10:1, 136.3 mg, 81% yield).

^{1}**H NMR** (400 MHz, $CDCl_3$) δ 7.66 (d, *J* = 8.1 Hz, 2H), 7.46 (d, *J* = 8.1 Hz, 2H), 6.96 (d, *J* = 2.6 Hz, 1H), 6.85 (d, *J* = 8.9 Hz, 1H), 6.67 (dd, *J* = 9.0, 2.6 Hz, 1H), 5.92 (s, 1H), 4.40 (s, 1H), 4.35 (s, 1H), 4.28 (t, *J* = 6.8 Hz, 2H), 3.83 (s, 3H), 3.66 (s, 2H), 2.39 (s, 3H), 1.59 – 1.48 (m, 2H), 1.48 – 1.06 (m, 12H), 0.86 (t, *J* = 6.9 Hz, 6H).

^{13}**C NMR** (100 MHz, $CDCl_3$) δ 170.7, 168.2, 159.3, 156.0, 153.8, 139.2, 135.9, 133.9, 131.1, 130.8, 130.6, 129.5, 129.1, 114.9, 112.5, 111.6, 101.2, 93.0, 62.1, 58.1, 55.6, 34.6, 30.3, 27.4, 27.2, 23.4, 14.1, 13.3.

HRMS (ES+) calcd for $C_{34}H_{40}ClNO_4Na^+$ $[M+Na]^+$: 584.2538, found: 584.2544.

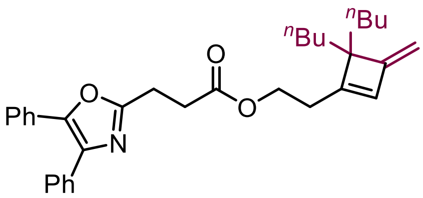

3aj

2-(4,4-Dibutyl-3-methylenecyclobut-1-en-1-yl)ethyl 3-(4,5-diphenyloxazol-2-yl)propanoate (3aj) was prepared as a colorless oil from 5-vinylidenenonane **1a** (45.6 mg, 0.3 mmol, 1.0 equiv), but-3-yn-1-yl 3-(4,5-diphenyloxazol-2-yl)propanoate **2aj** (144.9 mg, 0.42 mmol, 1.4 equiv), THF (3.0 mL) and [CpRu(MeCN)$_3$]PF$_6$ (6.5 mg, 15.0 μmol, 5.0 mol%) at room temperature for 12 h according to the General Procedure D (eluent: *n*-hexane/ethyl acetate = 10:1, 116.3 mg, 78% yield).

^{1}H NMR (400 MHz, CDCl$_3$) δ 7.64 – 7.62 (m, 2H), 7.57 (dd, *J* = 6.4, 1.8 Hz, 2H), 7.38 – 7.31 (m, 6H), 6.05 (s, 1H), 4.42 (s, 1H), 4.35 (s, 1H), 4.31 (t, *J* = 6.9 Hz, 2H), 3.20 (d, *J* = 7.1 Hz, 2H), 2.92 (t, *J* = 7.5 Hz, 2H), 2.46 – 2.40 (m, 2H), 1.59 – 1.51 (m, 2H), 1.44 – 1.38 (m, 2H), 1.32 – 1.22 (m, 8H), 0.88 (d, *J* = 7.0 Hz, 6H).

^{13}C NMR (100 MHz, CDCl$_3$) δ 171.9, 161.7, 159.3, 153.9, 145.4, 135.1, 132.3, 129.5, 128.9, 128.6, 128.5, 128.4, 128.0, 127.8, 126.4, 92.9, 61.8, 58.1, 34.6, 31.1, 27.4, 27.1, 23.44, 23.41, 14.0.

HRMS (ES+) calcd for $C_{33}H_{39}NO_3Na^+$ [M+Na]$^+$: 520.2822, found: 520.2827.

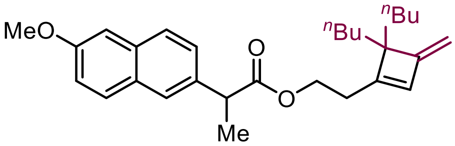

3ak

2-(4,4-Dibutyl-3-methylenecyclobut-1-en-1-yl)ethyl 2-(6-methoxynaphthalen-2-yl)propanoate (3ak) was prepared as a colorless oil from 5-vinylidenenonane **1a** (45.6 mg, 0.3 mmol, 1.0 equiv), but-3-yn-1-yl 2-(6-methoxynaphthalen-2-yl)propanoate **2ak** (118.4 mg, 0.42 mmol, 1.4 equiv),

THF (3.0 mL) and [CpRu(MeCN)$_3$]PF$_6$ (6.5 mg, 15.0 μmol, 5.0 mol%) at room temperature for 12 h according to the General Procedure D (eluent: *n*-hexane/ethyl acetate = 10:1, 93.7 mg, 72% yield).

^{1}H NMR (400 MHz, CDCl$_3$) δ 7.73 – 7.67 (m, 3H), 7.42 (dd, *J* = 8.4, 1.8 Hz, 1H), 7.18 – 7.11 (m, 2H), 5.89 (s, 1H), 4.37 (s, 1H), 4.32 (s, 1H), 4.31 – 4.20 (m, 2H), 3.92 (s, 3H), 3.86 (q, *J* = 7.1 Hz, 1H), 2.41 – 2.33 (m, 2H), 1.60 (d, *J* = 7.2 Hz, 3H), 1.56 – 1.47 (m, 2H), 1.41 – 1.14 (m, 10H), 0.88 (t, *J* = 7.0 Hz, 6H).

^{13}C NMR (100 MHz, CDCl$_3$) δ 174.5, 159.2, 157.6, 153.8, 135.5, 133.7, 129.5, 129.3, 128.9, 127.1, 126.2, 126.0, 118.9, 105.5, 92.7, 61.8, 58.0, 55.2, 45.4, 34.5, 27.3, 27.1, 23.4, 18.4, 14.0.

HRMS (ES+) calcd for $C_{29}H_{38}O_3Na^+$ $[M+Na]^+$: 457.2713, found: 457.2714.

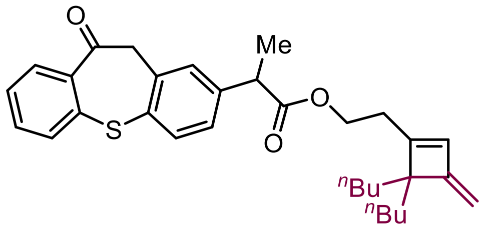

3al

2-(4,4-Dibutyl-3-methylenecyclobut-1-en-1-yl)ethyl 2-(10-oxo-10,11-dihydrodibenzo[b,f]thiepin-2-yl)propanoate (3al) was prepared as a colorless oil from 5-vinylidenenonane **1a** (45.6 mg, 0.3 mmol, 1.0 equiv), but-3-yn-1-yl 2-(1-but-3-yn-1-yl 2-(10-oxo-10,11-dihydrodibenzo[b,f]thiepin-2-yl)propanoatee **2al** (147.0 mg, 0.42 mmol, 1.4 equiv), THF (3.0 mL) and [CpRu(MeCN)$_3$]PF$_6$ (6.5 mg, 15.0 μmol, 5.0 mol%) at room temperature for 12 h according to the General Procedure D (eluent: *n*-hexane/ethyl acetate = 10:1, 106.9 mg, 71% yield).

^{1}H NMR (400 MHz, CDCl$_3$) δ 8.19 (dd, *J* = 8.0, 1.6 Hz, 1H), 7.59 (d, *J* = 7.9 Hz, 2H), 7.40 (ddd, *J* = 15.9, 7.8, 1.8 Hz, 2H), 7.34 – 7.28 (m, 1H), 7.14 (dd, *J* = 8.0, 2.0 Hz, 1H), 5.87 (d, *J* = 1.9 Hz, 1H), 4.38 (s, 1H), 4.35 (s, 2H), 4.32 (s, 1H), 4.29 – 4.13 (m, 2H), 3.70 (q, *J* = 7.1 Hz, 1H), 2.40 – 2.30 (m, 2H), 1.48 (d, *J* = 7.1 Hz, 3H), 1.46 – 1.03 (m, 12H), 0.86 (t, *J* = 6.9 Hz, 6H).

^{13}C NMR (100 MHz, $CDCl_3$) δ 191.3, 173.8, 159.2, 153.8, 142.5, 140.1, 137.9, 136.1, 133.2, 132.5, 131.5, 131.4, 130.8, 129.4, 128.6, 126.8, 126.3, 92.9, 62.0, 58.0, 51.0, 45.1, 34.5, 27.3, 27.0, 23.4, 18.3, 14.1.

HRMS (ES+) calcd for $C_{29}H_{38}O_3SNa^+$ $[M+Na]^+$: 525.2434, found: 525.2437.

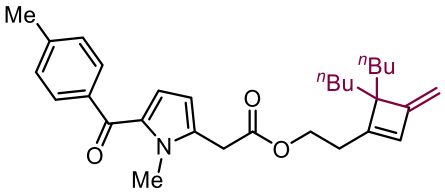

3am

2-(4,4-Dibutyl-3-methylenecyclobut-1-en-1-yl)ethyl 2-(1-methyl-5-(4-methylbenzoyl)-*1H*-pyrrol-2-yl)acetate (3am) was prepared as a colorless oil from 5-vinylidenenonane **1a** (45.6 mg, 0.3 mmol, 1.0 equiv), but-3-yn-1-yl 2-(1-methyl-5-(4-methylbenzoyl)-1H-pyrrol-2-yl)acetate **2am** (129.2 mg, 0.42 mmol, 1.4 equiv), THF (3.0 mL) and $[CpRu(MeCN)_3]PF_6$ (6.5 mg, 15.0 µmol, 5.0 mol%) at room temperature for 12 h according to the General Procedure D (eluent: *n*-hexane/ethyl acetate = 10:1, 96.8 mg, 70% yield).

^{1}H NMR (400 MHz, $CDCl_3$) δ 7.71 (d, *J* = 8.1 Hz, 2H), 7.24 (d, *J* = 7.8 Hz, 2H), 6.67 (d, *J* = 4.1 Hz, 1H), 6.10 (d, *J* = 4.0 Hz, 1H), 6.01 – 5.98 (m, 1H), 4.44 (s, 1H), 4.36 (s, 1H), 4.32 (t, *J* = 6.8 Hz, 2H), 3.94 (s, 3H), 3.71 (s, 2H), 2.46 – 2.43 (m, 2H), 2.42 (s, 3H), 1.55 (ddd, *J* = 13.5, 11.9, 3.6 Hz, 2H), 1.45 – 1.38 (m, 2H), 1.30 – 1.17 (m, 8H), 0.88 (d, *J* = 7.0 Hz, 6H).

^{13}C NMR (100 MHz, $CDCl_3$) δ 185.9, 169.3, 159.0, 153.8, 141.9, 137.3, 134.2, 131.4, 129.6, 129.4, 128.6, 122.2, 109.5, 93.1, 62.4, 58.2, 34.6, 33.2, 32.9, 27.4, 27.1, 23.4, 21.5, 14.1.

HRMS (ES+) calcd for $C_{30}H_{39}NO_3Na^+$ $[M+Na]^+$: 484.2822, found: 484.2829.

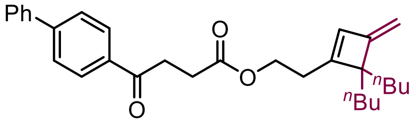

3an

2-(4,4-Dibutyl-3-methylenecyclobut-1-en-1-yl)ethyl 4-([1,1'-biphenyl]-4-yl)-4-oxobutanoate (3an) was prepared as a colorless oil from 5-vinylidenenonane **1a** (45.6 mg, 0.3 mmol, 1.0 equiv), 3-(but-3-yn-1-yloxy)-3-oxopropyl [1,1'-biphenyl]-4-carboxylate **2an** (135.2 mg, 0.42 mmol, 1.4 equiv), THF (3.0 mL) and $[CpRu(MeCN)_3]PF_6$ (6.5 mg, 15.0 μmol, 5.0 mol%) at room temperature for 12 h according to the General Procedure D (eluent: *n*-hexane/ethyl acetate = 10:1, 101.7 mg, 74% yield).

^{1}H NMR (400 MHz, $CDCl_3$) δ 8.07 – 8.05 (m, 2H), 7.71 – 7.68 (m, 2H), 7.65 – 7.61 (m, 2H), 7.47 (dd, *J* = 8.3, 6.6 Hz, 2H), 7.43 – 7.38 (m, 1H), 6.06 (s, 1H), 4.43 (s, 1H), 4.35 (s, 1H), 4.29 (t, *J* = 7.0 Hz, 2H), 3.35 (t, *J* = 6.6 Hz, 2H), 2.79 (t, *J* = 6.6 Hz, 2H), 2.44 (td, *J* = 6.9, 1.7 Hz, 2H), 1.59 – 1.52 (m, 2H), 1.46 – 1.39 (m, 2H), 1.31 – 1.20 (m, 8H), 0.89 (d, *J* = 7.0 Hz, 6H).

^{13}C NMR (100 MHz, $CDCl_3$) δ 197.6, 172.8, 159.5, 154.0, 145.9, 139.8, 135.2, 129.5, 128.9, 128.6, 128.2, 127.3 (2C), 92.8, 61.7, 58.2, 34.6, 33.4, 28.3, 27.4, 27.2, 23.5, 14.1.

HRMS (ES+) calcd for $C_{31}H_{38}O_3Na^+$ $[M+Na]^+$: 481.2713, found: 481.2720.

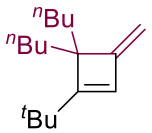

3ao

1-(*tert*-Butyl)-4,4-dibutyl-3-methylenecyclobut-1-ene (3ao) was prepared as a colorless oil from 5-vinylidenenonane **1a** (45.6 mg, 0.3 mmol, 1.0 equiv), 3,3-dimethylbut-1-yne (34.5 mg, 0.42 mmol, 1.4 equiv), THF (3.0 mL) and $[CpRu(MeCN)_3]PF_6$ (6.5 mg, 15.0 μmol, 5.0 mol%) at room temperature for 6 h according to the General Procedure D (eluent: *n*-hexane, 60.4 mg, 86% yield, rr = 2:1).

The two regioisomers could not be separated by silica gel chromatography. Shown below is the data of a mixture of the two isomers.

^{1}H NMR (400 MHz, $CDCl_3$) 6.08 (s, 0.37H), 6.06 (s, 0.22H), 6.02 (d, *J* = 0.7 Hz, 1H), 4.42 (s, 1H), 4.32 (d, *J* = 0.8 Hz, 1H), 2.77 (s, 0.78H), 2.63 (d, *J* = 1.1 Hz, 0.45H), 2.11 – 2.05 (m, 1H), 2.00 (td, *J* = 8.5, 6.3 Hz, 1H), 1.71 – 1.61 (m, 2H), 1.58 – 1.49 (m, 3H), 1.45 – 1.22 (m, 16H), 1.10 (s, 9H), 1.09 (s, 3H), 0.92 – 0.86 (m, 10H).

^{13}C NMR (100 MHz, $CDCl_3$) 171.1, 160.2, 159.4, 153.5, 133.4, 131.0, 127.3, 127.0, 124.7, 124.3, 122.3, 92.1, 59.9, 36.3, 34.4, 33.5, 32.8, 32.6, 32.0, 31.5, 31.0, 30.8, 30.7, 30.3, 30.2, 29.9, 29.0, 28.7, 27.9, 27.6, 23.6, 23.2, 14.1.

HRMS (CI+) calcd for $C_{17}H_{30}^{+}$ $[M]^{+}$: 234.2348, found: 234.2349.

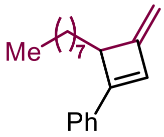

4a

(1-Ethyl-4-methylenecyclobut-2-ene-1,2-diyl)dibenzene (4a) was prepared as a colorless oil from undeca-1,2-diene **1b** (45.6 mg, 0.3 mmol, 1.0 equiv), ethynylbenzene **2n** (42.8 mg, 0.42 mmol, 1.4 equiv), THF (3.0 mL) and $[CpRu(MeCN)_3]PF_6$ (6.5 mg, 15.0 μmol, 5.0 mol%) at room temperature for 6 h according to the General Procedure D (eluent: *n*-hexane, 50.3 mg, 66% yield).

^{1}H NMR (400 MHz, $CDCl_3$) δ 7.44 – 7.39 (m, 2H), 7.38 – 7.33 (m, 2H), 7.31 – 7.24 (m, 1H), 6.55 (s, 1H), 4.72 (d, *J* = 1.3 Hz, 1H), 4.63 (s, 1H), 3.60 – 3.54 (m, 1H), 1.95 – 1.80 (m, 1H), 1.67 – 1.57 (m, 1H), 1.47 – 1.35 (m, 2H), 1.33 – 1.23 (m, 10H), 0.86 (t, *J* = 6.8 Hz, 3H).

^{13}C NMR (100 MHz, $CDCl_3$) δ 154.8, 150.4, 133.7, 128.5, 128.3, 127.8, 125.8, 97.1, 48.8, 31.9, 31.0, 30.1, 29.5, 29.3, 27.0, 22.7, 14.1.

HRMS (CI+) calcd for $C_{19}H_{26}^{+}$ $[M]^{+}$: 254.2035, found: 254.2037.

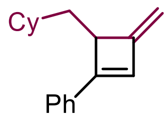

4b

(4-(Cyclohexylmethyl)-3-methylenecyclobut-1-en-1-yl)benzene (4b) was prepared as a colorless oil from buta-2,3-dien-1-ylcyclohexane **1c** (40.8 mg, 0.3 mmol, 1.0 equiv), ethynylbenzene **2n** (42.8 mg, 0.42 mmol, 1.4 equiv), THF (3.0 mL) and [CpRu(MeCN)$_3$]PF$_6$ (6.5 mg, 15.0 μmol, 5.0 mol%) at room temperature for 6 h according to the General Procedure D (eluent: *n*-hexane, 37.8 mg, 53% yield).

^{1}H NMR (400 MHz, CDCl$_3$) δ 7.43 – 7.33 (m, 4H), 7.28 (d, *J* = 7.1 Hz, 1H), 6.54 (s, 1H), 4.71 (s, 1H), 4.63 (s, 1H), 3.65 (dd, *J* = 9.8, 4.0 Hz, 1H), 2.02 - 1.92 (m, 1H), 1.80 – 1.62 (m, 6H), 1.53 – 1.43 (m, 2H), 1.28 – 1.20 (m, 2H), 1.00 – 0.88 (m, 2H).

^{13}C NMR (100 MHz, CDCl$_3$) δ 155.3, 151.1, 133.5, 128.5, 128.3, 127.6, 125.9, 97.3, 46.3, 39.8, 36.9, 34.6, 33.2, 26.7, 26.4, 26.3.

HRMS (CI+) calcd for $C_{18}H_{22}^+$ [M]$^+$: 238.1722, found: 238.1727.

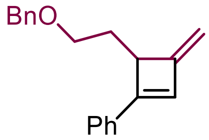

4c

(4-(2-(Benzyloxy)ethyl)-3-methylenecyclobut-1-en-1-yl)benzene (4c) was prepared as a colorless oil from ((penta-3,4-dien-1-yloxy)methyl)benzene **1d** (52.2 mg, 0.3 mmol, 1.0 equiv), ethynylbenzene **2n** (42.8 mg, 0.42 mmol, 1.4 equiv), THF (3.0 mL) and [CpRu(MeCN)$_3$]PF$_6$ (6.5 mg, 15.0 μmol, 5.0 mol%) at room temperature for 6 h according to the General Procedure D (eluent: *n*-hexane/ethyl acetate = 20:1, 41.4 mg, 50% yield).

^{1}H NMR (400 MHz, CDCl$_3$) δ 7.48 – 7.44 (m, 2H), 7.40 – 7.29 (m, 8H), 6.57 (s, 1H), 4.75 (d, *J* = 1.4 Hz, 1H), 4.62 (s, 1H), 4.52 (d, *J* = 4.0 Hz, 2H), 3.75 (dd, *J* = 8.4, 4.3 Hz, 1H), 3.71 – 3.62 (m, 2H), 2.31 – 2.23 (m, 1H), 2.02 - 1.93 (m, 1H).

^{13}C NMR (100 MHz, $CDCl_3$) δ 154.5, 149.7, 138.5, 133.4, 128.5, 128.4, 128.3, 127.9, 127.6, 127.5, 125.9, 97.6, 72.9, 68.8, 45.9, 31.3.

HRMS (CI+) calcd for $C_{20}H_{20}O^+$ $[M]^+$: 276.1514, found: 276.1516.

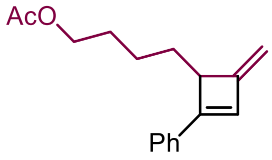

4d

4-(4-Methylene-2-phenylcyclobut-2-en-1-yl)butyl acetate (4d) was prepared as a colorless oil from hepta-5,6-dien-1-yl acetate **1e** (46.2 mg, 0.3 mmol, 1.0 equiv), ethynylbenzene **2n** (42.8 mg, 0.42 mmol, 1.4 equiv), THF (3.0 mL) and $[CpRu(MeCN)_3]PF_6$ (6.5 mg, 15.0 μmol, 5.0 mol%) at room temperature for 6 h according to the General Procedure D (eluent: *n*-hexane/ethyl acetate = 20:1, 46.1 mg, 61% yield).

^{1}H NMR (400 MHz, $CDCl_3$) δ 7.43 – 7.34 (m, 4H), 7.31 – 7.27 (m, 1H), 6.56 (s, 1H), 4.73 (d, *J* = 1.3 Hz, 1H), 4.62 (s, 1H), 4.03 (t, *J* = 6.7 Hz, 2H), 3.63 – 3.57 (m, 1H), 2.01 (s, 3H), 1.94 -1.86 (m, 1H), 1.72 – 1.60 (m, 3H), 1.52 – 1.41 (m, 2H).

^{13}C NMR (100 MHz, $CDCl_3$) δ 171.2, 154.4, 149.9, 133.5, 128.5, 128.4, 128.0, 125.8, 97.2, 64.4, 48.5, 30.3, 28.9, 23.1, 20.9.

HRMS (CI+) calcd for $C_{17}H_{20}O_2^+$ $[M+Na]^+$: 256.1463, found: 256.1460.

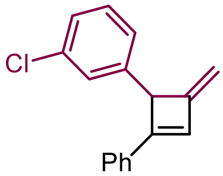

4e

1-Chloro-3-(4-methylene-2-phenylcyclobut-2-en-1-yl)benzene (4e) was prepared as a colorless oil from 1-chloro-3-(propa-1,2-dien-1-yl)benzene **1f** (45.0 mg, 0.3 mmol, 1.0 equiv), ethynylbenzene **2n** (42.8 mg, 0.42 mmol, 1.4 equiv), THF (3.0 mL) and $[CpRu(MeCN)_3]PF_6$ (6.5 mg, 15.0 μmol, 5.0 mol%) at

room temperature for 6 h according to the General Procedure D (eluent: *n*-hexane, 54.4 mg, 72% yield, rr = 8:1).

^{1}H NMR (400 MHz, $CDCl_3$) δ 7.34 – 7.25 (m, 6H), 7.22 – 7.13 (m, 3H), 6.84 (s, 1H), 4.80 (d, *J* = 1.0 Hz, 1H), 4.54 (s, 1H), 4.47 (d, *J* = 0.9 Hz, 1H).

^{13}C NMR (100 MHz, $CDCl_3$) (mixture) δ 152.8, 149.8, 141.9, 134.3, 132.5, 130.3, 129.8, 129.7, 129.0, 128.8, 128.60, 128.56, 127.5, 127.01, 126.95, 126.8, 126.1, 126.0, 125.8, 125.6, 125.4, 113.6, 98.5, 53.7, 36.9.

HRMS (ES+) calcd for $C_{17}H_{13}Cl^+$ $[M]^+$: 252.0706, found:252.0702.

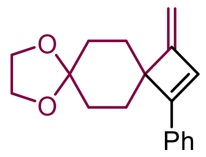

4f

3-Methylene-1-phenyl-8,11-dioxadispiro[3.2.47.24]tridec-1-ene (4f) was prepared as a colorless oil from 8-vinylidene-1,4-dioxaspiro[4.5]decane **1g** (49.8 mg, 0.3 mmol, 1.0 equiv), ethynylbenzene **2n** (42.8 mg, 0.42 mmol, 1.4 equiv), THF (3.0 mL) and $[CpRu(MeCN)_3]PF_6$ (6.5 mg, 15.0 μmol, 5.0 mol%) at room temperature for 6 h according to the General Procedure D (eluent: *n*-hexane/ethyl acetate = 20:1, 70.0 mg, 87% yield).

^{1}H NMR (400 MHz, $CDCl_3$) δ 7.50 – 7.45 (m, 2H), 7.38 – 7.32 (m, 2H), 7.29 – 7.26 (m, 1H), 6.53 (s, 1H), 4.85 (s, 1H), 4.68 (s, 1H), 4.04 – 3.99 (m, 4H), 2.42 – 2.34 (m, 2H), 2.01 – 1.93 (m, 2H), 1.90 – 1.80 (m, 4H).

^{13}C NMR (100 MHz, $CDCl_3$) δ 158.1, 156.3, 132.9, 128.6, 128.4, 126.7, 126.0, 108.7, 97.1, 64.4, 64.3, 53.8, 34.1, 31.3.

HRMS (CI+) calcd for $C_{18}H_{20}O_2^+$ $[M]^+$: 268.1463, found: 268.1468.

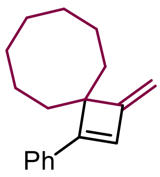

4g

3-Methylene-1-phenylspiro[3.7]undec-1-ene (4g) was prepared as a colorless oil from vinylidenecycloheptane **1h** (36.6 mg, 0.3 mmol, 1.0 equiv), ethynylbenzene **2n** (42.8 mg, 0.42 mmol, 1.4 equiv), THF (3.0 mL) and $[CpRu(MeCN)_3]PF_6$ (6.5 mg, 15.0 µmol, 5.0 mol%) at room temperature for 6 h according to the General Procedure D (eluent: *n*-hexane, 53.6 mg, 75% yield, rr = 18:1).

^{1}H NMR (400 MHz, $CDCl_3$) δ 7.52 – 7.47 (m, 2H), 7.39 – 7.33 (m, 2H), 7.31 – 7.25 (m, 1H), 6.52 (d, *J* = 0.8 Hz, 1H), 4.78 (d, *J* = 0.8 Hz, 1H), 4.64 (s, 1H), 2.18 – 2.11 (m, 2H), 2.07 – 2.00 (m, 2H), 1.87 – 1.62 (m, 10H).

^{13}C NMR (100 MHz, $CDCl_3$) δ 161.83, 157.78, 133.61, 128.5, 128.1, 126.5, 126.2, 95.8, 57.8, 33.0, 28.9, 25.5, 24.5.

HRMS (CI+) calcd for $C_{18}H_{22}$ $[M]^+$: 238.1722, found: 238.1736.

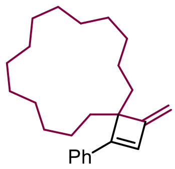

4h

3-Methylene-1-phenylspiro[3.14]octadec-1-ene (4h) was prepared as a colorless oil from vinylidenecyclopentadecane **1i** (70.2 mg, 0.3 mmol, 1.0 equiv), ethynylbenzene **2n** (42.8 mg, 0.42 mmol, 1.4 equiv), THF (3.0 mL) and $[CpRu(MeCN)_3]PF_6$ (6.5 mg, 15.0 µmol, 5.0 mol%) at room temperature for 6 h according to the General Procedure D (eluent: *n*-hexane, 80.7 mg, 80% yield).

^{1}H NMR (400 MHz, $CDCl_3$) δ 7.50 – 7.44 (m, 2H), 7.36 (t, *J* = 7.6 Hz, 2H), 7.31 – 7.26 (m, 1H), 6.55 (s, 1H), 4.67 (s, 2H), 2.0 – 1.93 (m, 2H), 1.87 – 1.80 (m, 2H), 1.55 – 1.48 (m, 4H), 1.45 – 1.35 (m, 20H).

^{13}C NMR (100 MHz, $CDCl_3$) δ 159.9, 155.5, 134.0, 128.4, 128.1, 127.2, 126.2, 95.9, 58.0, 35.2, 28.1, 27.0, 26.8, 26.7, 26.5, 24.6.

HRMS (CI+) calcd for $C_{25}H_{36}^{+}$ $[M]^{+}$: 336.2817, found: 336.2821.

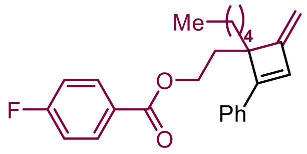

4i

2-(4-Methylene-1-pentyl-2-phenylcyclobut-2-en-1-yl)ethyl 4-fluorobenzoate (4i) was prepared as a colorless oil from 3-vinylideneoctyl 4-fluorobenzoate **1j** (82.8 mg, 0.3 mmol, 1.0 equiv), ethynylbenzene **2n** (42.8 mg, 0.42 mmol, 1.4 equiv), THF (3.0 mL) and $[CpRu(MeCN)_3]PF_6$ (6.5 mg, 15.0 μmol, 5.0 mol%) at room temperature for 6 h according to the General Procedure D (eluent: *n*-hexane/ethyl acetate = 10:1, 89.6 mg, 79% yield).

^{1}H NMR (400 MHz, $CDCl_3$) δ 7.70 – 7.61 (m, 2H), 7.41 – 7.35 (m, 2H), 7.34 – 7.25 (m, 3H), 6.97 – 6.88 (m, 2H), 6.59 (s, 1H), 4.72 (s, 1H), 4.62 (s, 1H), 4.41 – 4.35 (m, 1H), 4.28 – 4.21 (m, 1H), 2.29 – 2.25 (m, 2H), 1.90 – 1.74 (m, 2H), 1.43 – 1.21 (m, 6H), 0.85 – 0.79 (m, 3H).

^{13}C NMR (100 MHz, $CDCl_3$) δ 165.6, 165.4 (d, J = 253.2 Hz), 155.6, 152.0, 133.6, 131.9 (d, J = 9.2 Hz), 128.6, 128.5, 127.7, 126.5 (d, J = 3.0 Hz), 125.9, 115.1 (d, J = 21.9 Hz), 95.5, 62.8, 56.4, 35.9, 33.8, 32.4, 24.4, 22.4, 14.0.

^{19}F NMR (377 MHz, $CDCl_3$) δ -106.53.

HRMS (CI+) calcd for $C_{25}H_{27}FO_2^{+}$ $[M]^{+}$: 378.1995, found: 378.2020.

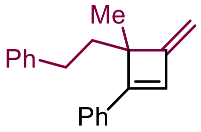

4j

(4-Methyl-3-methylene-4-phenethylcyclobut-1-en-1-yl)benzene (4j) was prepared as a colorless oil from (3-methylpenta-3,4-dien-1-yl)benzene **1k** (47.4

mg, 0.3 mmol, 1.0 equiv), ethynylbenzene **2n** (42.8 mg, 0.42 mmol, 1.4 equiv), THF (3.0 mL) and [CpRu(MeCN)$_3$]PF$_6$ (6.5 mg, 15.0 μmol, 5.0 mol%) at room temperature for 6 h according to the General Procedure D (eluent: *n*-hexane, 52.3 mg, 67% yield, rr = 17:1).

^{1}H NMR (400 MHz, CDCl$_3$) δ 7.46 – 7.41 (m, 2H), 7.38 – 7.33 (m, 2H), 7.32 – 7.19 (m, 3H), 7.16 – 7.07 (m, 3H), 6.60 (d, *J* = 0.8 Hz, 1H), 4.71 (s, 1H), 4.65 (s, 1H), 2.71 – 2.62 (m, 1H), 2.53 – 2.43 (m, 1H), 2.11 – 2.03 (m, 2H), 1.49 (s, 3H).

^{13}C NMR (100 MHz, CDCl$_3$) δ 157.7, 155.0, 143.0, 133.3, 128.6, 128.5, 128.2 (2C), 126.9, 125.9, 125.5, 94.6, 54.2, 38.2, 31.8, 23.1.

HRMS (CI+) calcd for $C_{20}H_{20}^+$ [M]$^+$: 260.1565, found: 260.1564.

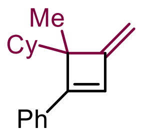

4k

(4-Cyclohexyl-4-methyl-3-methylenecyclobut-1-en-1-yl)benzene (4k) was prepared as a colorless oil from buta-2,3-dien-2-ylcyclohexane **1l** (40.8 mg, 0.3 mmol, 1.0 equiv), ethynylbenzene **2n** (42.8 mg, 0.42 mmol, 1.4 equiv), THF (3.0 mL) and [CpRu(MeCN)$_3$]PF$_6$ (6.5 mg, 15.0 μmol, 5.0 mol%) at room temperature for 6 h according to the General Procedure D (eluent: *n*-hexane, 62.8 mg, 88% yield).

^{1}H NMR (400 MHz, CDCl$_3$) δ 7.43 – 7.38 (m, 2H), 7.36 – 7.31 (m, 2H), 7.29 – 7.23 (m, 1H), 6.51 (s, 1H), 4.64 (s, 1H), 4.56 (s, 1H), 2.06 – 1.98 (m, 1H), 1.77 – 1.59 (m, 4H), 1.50 (s, 3H), 1.32 – 1.01 (m, 6H).

^{13}C NMR (100 MHz, CDCl$_3$) δ 158.5, 154.6, 133.7, 128.5, 128.2, 126.9, 125.9, 96.1, 57.9, 43.3, 29.4, 29.0, 27.2, 26.8, 26.5, 21.2.

HRMS (CI+) calcd for $C_{18}H_{22}^+$ [M]$^+$: 238.1722, found: 238.1722.

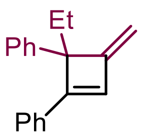

4l

(1-Ethyl-4-methylenecyclobut-2-ene-1,2-diyl)dibenzene (4l) was prepared as a colorless oil from (3-methylpenta-3,4-dien-1-yl)benzene **1m** (47.4 mg, 0.3 mmol, 1.0 equiv), ethynylbenzene **2n** (42.8 mg, 0.42 mmol, 1.4 equiv), THF (3.0 mL) and $[CpRu(MeCN)_3]PF_6$ (6.5 mg, 15.0 μmol, 5.0 mol%) at room temperature for 6 h according to the General Procedure D (eluent: *n*-hexane, 53.9 mg, 73% yield).

^{1}H NMR (400 MHz, $CDCl_3$) δ 7.47 – 7.42 (m, 2H), 7.34 – 7.17 (m, 8H), 6.83 (s, 1H), 4.69 (s, 1H), 4.50 (s, 1H), 2.48 – 2.38 (m, 1H), 2.23 – 2.14 (m, 1H), 0.92 (t, *J* = 7.4 Hz, 3H).

^{13}C NMR (100 MHz, $CDCl_3$) δ 155.1, 153.9, 143.6, 133.1, 129.4, 128.51, 128.49, 128.2, 126.3, 126.24, 126.21, 96.0, 61.4, 25.4, 9.6.

HRMS (CI+) calcd for $C_{19}H_{18}^+$ $[M]^+$: 246.1409, found: 246.1406.

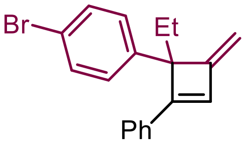

4m

1-Bromo-4-(1-ethyl-4-methylene-2-phenylcyclobut-2-en-1-yl)benzene (4m) was prepared as a colorless oil from (3-methylpenta-3,4-dien-1-yl)benzene **1n** (47.4 mg, 0.3 mmol, 1.0 equiv), ethynylbenzene **2n** (42.8 mg, 0.42 mmol, 1.4 equiv), THF (3.0 mL) and $[CpRu(MeCN)_3]PF_6$ (6.5 mg, 15.0 μmol, 5.0 mol%) at room temperature for 6 h according to the General Procedure D (eluent: *n*-hexane, 82.6 mg, 85% yield).

^{1}H NMR (400 MHz, $CDCl_3$) δ 7.41 – 7.36 (m, 2H), 7.33 – 7.24 (m, 7H), 6.83 (s, 1H), 4.70 (s, 1H), 4.46 (s, 1H), 2.37 (dq, *J* = 14.5, 7.3 Hz, 1H), 2.15 (dq, *J* = 14.5, 7.4 Hz, 1H), 0.91 (t, *J* = 7.3 Hz, 3H).

^{13}**C NMR** (100 MHz, $CDCl_3$) δ 154.7, 153.5, 142.7, 132.7, 131.3, 129.6, 128.7, 128.6, 128.1, 126.2, 120.1, 96.3, 60.9, 25.2, 9.5.

HRMS (CI+) calcd for $C_{19}H_{17}Br^+$ $[M]^+$: 324.0514, found: 324.0514.

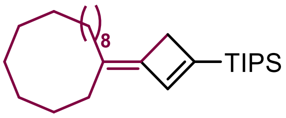

5a

(3-Cyclopentadecylidenecyclobut-1-en-1-yl)triisopropylsilane (5a) was prepared as a colorless oil from vinylidenecyclopentadecane **1i** (70.2 mg, 0.3 mmol, 1.0 equiv), ethynyltriisopropylsilane (65.5 mg, 0.36 mmol, 1.2 equiv), THF (3.0 mL) and $[CpRu(MeCN)_3]PF_6$ (6.5 mg, 15.0 µmol, 5.0 mol%) at room temperature for 12 h according to the General Procedure E (eluent: *n*-hexane, 106.1 mg, 85% yield).

^{1}**H NMR** (400 MHz, $CDCl_3$) δ 6.90 (s, 1H), 2.97 (s, 2H), 2.16 – 2.07 (m, 2H), 2.03 – 1.95 (m, 2H), 1.51 – 1.33 (m, 24H), 1.17 – 1.11 (m, 3H), 1.08 (d, *J* = 5.5 Hz, 18H).

^{13}**C NMR** (100 MHz, $CDCl_3$) δ 148.7, 147.4, 137.0, 124.2, 40.1, 31.0, 30.5, 27.9, 27.8, 27.6, 27.4, 26.8 (2C), 26.7, 26.6, 26.5, 26.44, 26.39, 26.3, 18.8, 11.1.

HRMS (CI+) calcd for $C_{28}H_{52}Si^+$ $[M]^+$: 416.3838, found: 416.3838.

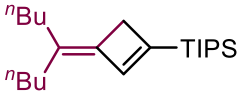

5b

Triisopropyl(3-(nonan-5-ylidene)cyclobut-1-en-1-yl)silane (5b) was prepared as a colorless oil from 5-vinylidenenonane **1a** (45.6 mg, 0.3 mmol, 1.0 equiv), ethynyltriisopropylsilane (65.5 mg, 0.36 mmol, 1.2 equiv), THF (3.0 mL) and $[CpRu(MeCN)_3]PF_6$ (6.5 mg, 15.0 µmol, 5.0 mol%) at room temperature for 12 h according to the General Procedure E (eluent: *n*-hexane, 93.2 mg, 93% yield).

^{1}H NMR (400 MHz, $CDCl_3$) δ 6.91 (s, 1H), 2.98 (s, 2H), 2.17 – 2.07 (m, 2H), 2.03 – 1.95 (m, 2H), 1.44 – 1.28 (m, 8H), 1.17 – 1.11 (m, 3H), 1.09 (d, *J* = 5.3 Hz, 18H), 0.95 – 0.90 (m, 6H).

^{13}C NMR (100 MHz, $CDCl_3$) δ 148.6, 147.4, 136.8, 123.9, 40.1, 30.8, 30.7, 30.1, 29.6, 22.81, 22.75, 18.8, 14.1, 14.0, 11.1.

HRMS (CI+) calcd for $C_{22}H_{42}Si^+$ $[M]^+$: 334.3056, found: 334.3057.

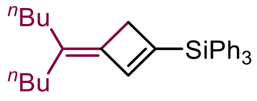

5c

(3-(Nonan-5-ylidene)cyclobut-1-en-1-yl)triphenylsilane (5c) was prepared as white solid from 5-vinylidenenonane **1a** (45.6 mg, 0.3 mmol, 1.0 equiv), ethynyltriphenylsilane (102.2 mg, 0.36 mmol, 1.2 equiv), MeCN (3.0 mL) and $[CpRu(MeCN)_3]PF_6$ (6.5 mg, 15.0 μmol, 5.0 mol%) at room temperature for 12 h according to the General Procedure E (eluent: *n*-hexane, 79.8 mg, 61% yield).

^{1}H NMR (400 MHz, $CDCl_3$) δ 7.60 – 7.56 (m, 6H), 7.47 – 7.37 (m, 9H), 7.07 (s, 1H), 3.18 (s, 2H), 2.17 – 2.12 (m, 2H), 2.05 – 2.00 (m, 2H), 1.48 – 1.27 (m, 8H), 0.95 – 0.91 (m, 6H).

^{13}C NMR (100 MHz, $CDCl_3$) δ 150.1, 145.5, 136.2, 135.8, 134.3, 129.5, 127.9, 126.6, 38.8, 30.7, 30.6, 30.2, 29.7, 22.8, 22.7, 14.04, 14.01.

HRMS (CI+) calcd for $C_{31}H_{36}Si^+$ $[M]^+$: 436.2586, found: 436.2584.

Unsuccessful examples:

We also evaluated other di-, tri-, and tetrasubstituted internal allenes, but these reaction failed to form the desired products. Shown below are some details. For those low conversion cases, oligomerization of the alkyne partner may account for the remainder of the mass balance. For other cases, both partners may decompose by oligomerization, but no desired allene-alkyne [2+2] cycloaddition product was formed.

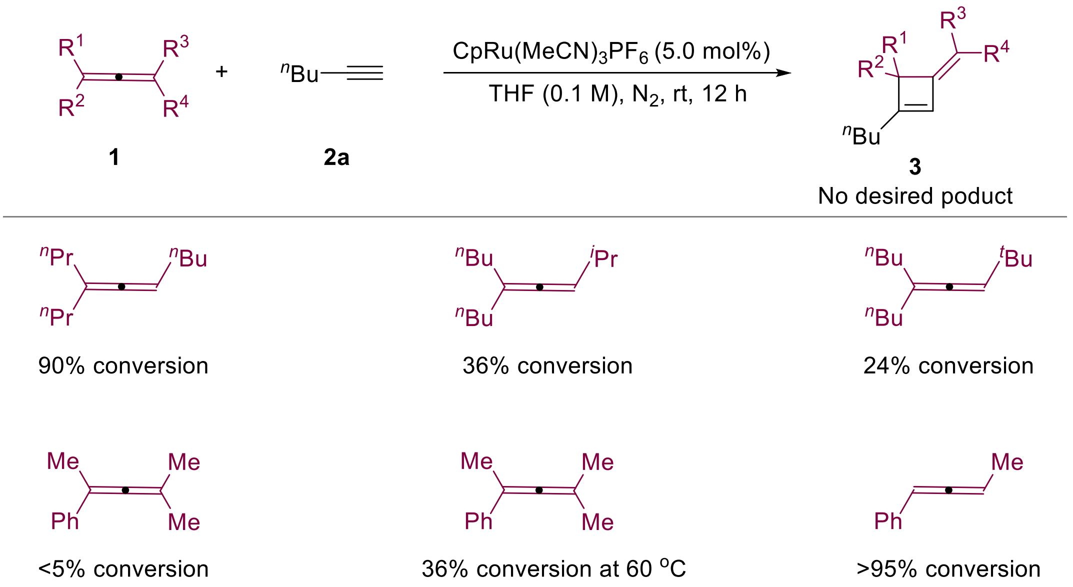

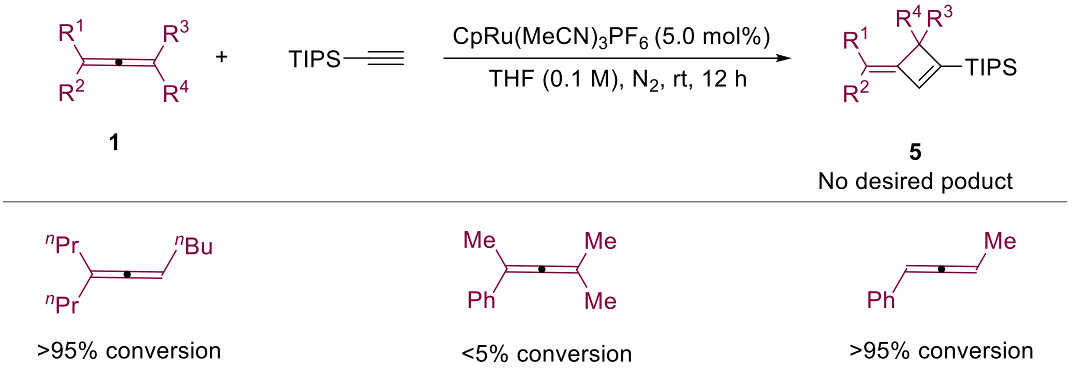

IV. Scale-up Reaction and Product Transformations

Scale-up Reaction

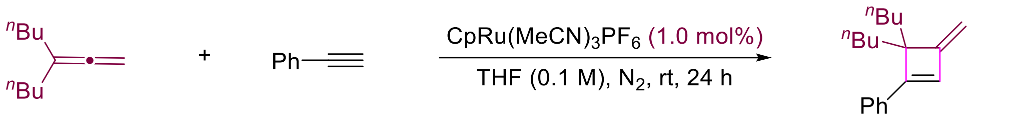

1a 2.0 mmol　　1.4 equiv　　**3n** 406.4 mg, 80% yield

A 50-mL oven-dried Schlenk flask charged with 5-vinylidenenonane **1a** (304 mg, 2.0 mmol, 1.0 equiv) and $CpRu(MeCN)_3PF_6$ (8.7 mg, 0.02 mmol, 1.0 mol%) was evacuated and backfilled with N_2 for three times. Then, ethynylbenzene (0.31 mL, 2.8 mmol, 1.4 equiv) and THF (20 mL) were added under N_2. The mixture was stirred at room temperature for 24 h before it was concentrated *in vacuo*. The residue was purified by silica gel flash column chromatography to afford the desired product **3n** as a coloreless oil (eluent: *n*-hexane, 406.4 mg, 80% yield).

Product Transformations

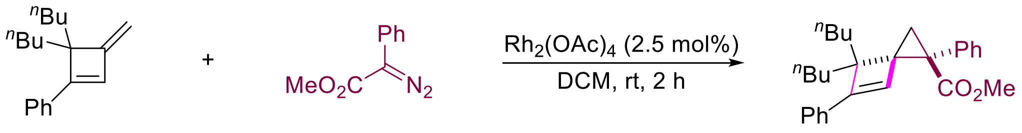

3n　　1.5 equiv　　**6**

Methyl 6,6-dibutyl-1,5-diphenylspiro[2.3]hex-4-ene-1-carboxylate (6). Under N_2, an oven-dried 4-mL flask was charged with (4,4-dibutyl-3-methylenecyclobut-1-en-1-yl)benzene **3n** (50.8 mg, 0.2 mmol, 1.0 equiv), $Rh_2(OAc)_4$ (2.2 mg, 5.0 μmol, 2.5 mol%), and DCM (2.0 mL). Then, methyl 2-diazo-2-phenylacetate (52.8, 0.3 mmol, 1.5 equiv) dissolved in DCM (0.5 mL) was added slowly over 10 min. The reaction mixture was stirred at room temperature for 2 h before it was concentrated *in vacuo*. The residue was purified by flash silica gel chromatography to afford the product **6** as a colorless oil (eluent: *n*-hexane/ethyl acetate = 20:1→10:1, 68.3 mg, 85% yield, >20:1 dr).

^{1}**H NMR** (400 MHz, $CDCl_3$) δ 7.51 – 7.46 (m, 2H), 7.35 – 7.30 (m, 6H), 7.28 – 7.22 (m, 2H), 6.51 (s, 1H), 3.77 (s, 3H), 2.03 (d, *J* = 5.5 Hz, 1H), 1.90 (d, *J* = 5.5 Hz, 1H), 1.61 – 1.54 (m, 2H), 1.40 – 1.15 (m, 8H), 1.11 – 0.92 (m, 2H), 0.87 (t, *J* = 7.0 Hz, 3H), 0.75 (t, *J* = 6.9 Hz, 3H).

^{13}**C NMR** (100 MHz, $CDCl_3$) δ 173.1, 150.7, 134.7, 134.1, 129.9, 128.6, 128.4, 127.6, 127.5, 126.7, 125.0, 56.6, 52.1, 48.1, 37.7, 36.3, 34.6, 27.7, 26.6, 23.2, 22.4, 19.6, 14.1, 14.0.

HRMS (ES+) calcd for $C_{28}H_{34}O_2Na^+$ $[M+Na]^+$: 425.2451, found: 425.2457.

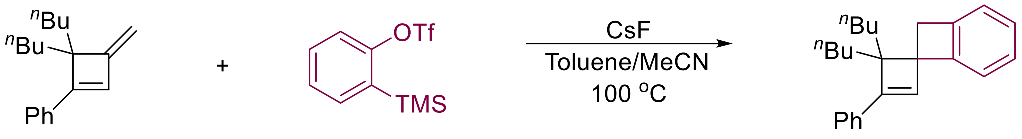

3n   3.0 equiv   7

4',4'-Dibutyl-3'-phenylspiro[bicyclo[4.2.0]octane-7,1'-cyclobutane]-1(6),2,2',4-tetraene (7). An oven-dried 5-mL flask was charged with (4,4-dibutyl-3-methylenecyclobut-1-en-1-yl)benzene **3n** (50.8 mg, 0.2 mmol, 1.0 equiv), 2-(trimethylsilyl)phenyl trifluoromethanesulfonate (180 mg, 0.6 mmol, 3.0 equiv), CsF (92 mg, 0.6 mmol, 3.0 equiv), toluene (1.0 mL), and MeCN (1.0 mL). The mixture was stirred at 100 °C for 12 h before it was cooled to room temeature and concentrated *in vacuo*. The residue was purified by flash silica gel chromatography to afford the product **7** as a colorless oil (eluent: *n*-hexane, 44.9 mg, 68% yield).

^{1}**H NMR** (400 MHz, $CDCl_3$) δ 7.43 (d, *J* = 7.1 Hz, 2H), 7.34 (t, *J* = 7.5 Hz, 2H), 7.26 – 7.04 (m, 5H), 6.55 (s, 1H), 3.42 (d, *J* = 14.3 Hz, 1H), 3.18 (d, *J* = 14.2 Hz, 1H), 2.05 – 1.98 (m, 1H), 1.86 – 1.80 (m, 2H), 1.79 – 1.73 (m, 1H), 1.33 – 1.13 (m, 8H), 0.88 (t, *J* = 7.1 Hz, 3H), 0.73 (t, *J* = 7.3 Hz, 3H).

^{13}**C NMR** (100 MHz, $CDCl_3$) δ 152.2, 150.2, 143.1, 134.5, 130.8, 128.4, 127.6, 127.5, 126.7, 125.6, 122.5, 122.2, 61.5, 55.6, 37.5, 37.3, 34.9, 27.7, 26.9, 23.4, 23.3, 14.1, 14.0.

HRMS (CI+) calcd for $C_{25}H_{30}^+$ $[M]^+$: 330.2348, found: 330.2349.

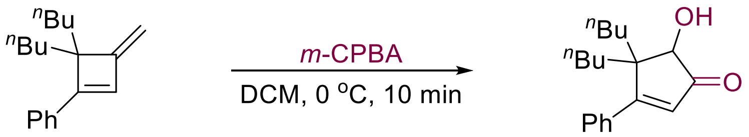

3n **8**

4,4-Dibutyl-5-hydroxy-3-phenylcyclopent-2-en-1-one (8). At 0 °C, an oven-dried 4-mL flask was charged with (4,4-dibutyl-3-methylenecyclobut-1-en-1-yl)benzene **3n** (50.8 mg, 0.2 mmol, 1.0 equiv) and DCM (2.0 mL). Then *m*-CPBA (128 mg, 0.6 mmol, 3.0 equiv) was added, and the reaction mixture was stirred at 0 °C for 10 min before it was diluted with a saturated aqueous solution of $NaHCO_3$ (4.0 mL). The mixture was extracted with ethyl acetate (5.0 mL × 3). The combined organic layers were washed with brine (10 mL), dried over Na_2SO_4, and concentrated *in vacuo*. The residue was purified by silica gel flash column chromatography to afford the product **8** as a colorless oil (eluent: *n*-hexane/ethyl acetate = 20:1→10:1, 49.2 mg, 86% yield).

^{1}H NMR (400 MHz, $CDCl_3$) δ 7.53 – 7.47 (m, 2H), 7.45 – 7.42 (m, 3H), 6.39 (s, 1H), 4.12 (s, 1H), 3.05 (s, 1H), 2.24 – 2.16 (m, 1H), 1.80 – 1.68 (m, 2H), 1.59 – 1.50 (m, 1H), 1.40 – 1.14 (m, 6H), 0.98 – 0.90 (m, 2H), 0.83 (t, *J* = 7.4 Hz, 3H), 0.77 (t, *J* = 7.1 Hz, 3H).

^{13}C NMR (100 MHz, $CDCl_3$) δ 207.3, 177.9, 135.2, 130.2, 128.8, 127.6, 127.3, 79.0, 54.7, 37.9, 34.3, 28.2, 26.5, 23.1, 23.0, 13.9, 13.8.

HRMS (ES+) calcd for $C_{19}H_{26}ONa^+$ $[M+Na]^+$: 309.1825, found: 309.1829.

V. Mechanistic Experiments

(1) Deuterium-labeling experiment

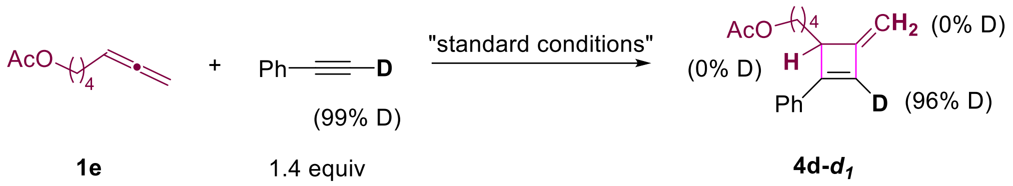

4-(4-Methylene-2-phenylcyclobut-2-en-1-yl-3-d)butyl acetate (4d-*d*1). In a glove box, to an oven-dried 4-mL vial charged with allene **1a** (15.4 mg, 0.1 mmol, 1.0 equiv), deuterated phenylacetylene (14.4 mg, 0.14 mmol, 1.4 equiv), and THF (1.0 mL) was added $[CpRu(MeCN)_3]PF_6$ (2.2 mg, 5.0 μmol, 5.0 mol%). The vial was capped and removed from the glove box. The reaction mixture was stirred at room temperature for 6 h before it was filtered through a short pad of silica gel. The filtrate was concentrated *in vacuo*, and the residue was purified by silica gel flash column chromatography to give the desired product **4d-*d*1** (eluent: *n*-hexane/ethyl acetate = 100:1→50:1, 13.1 mg, 51% yield, 96%D).

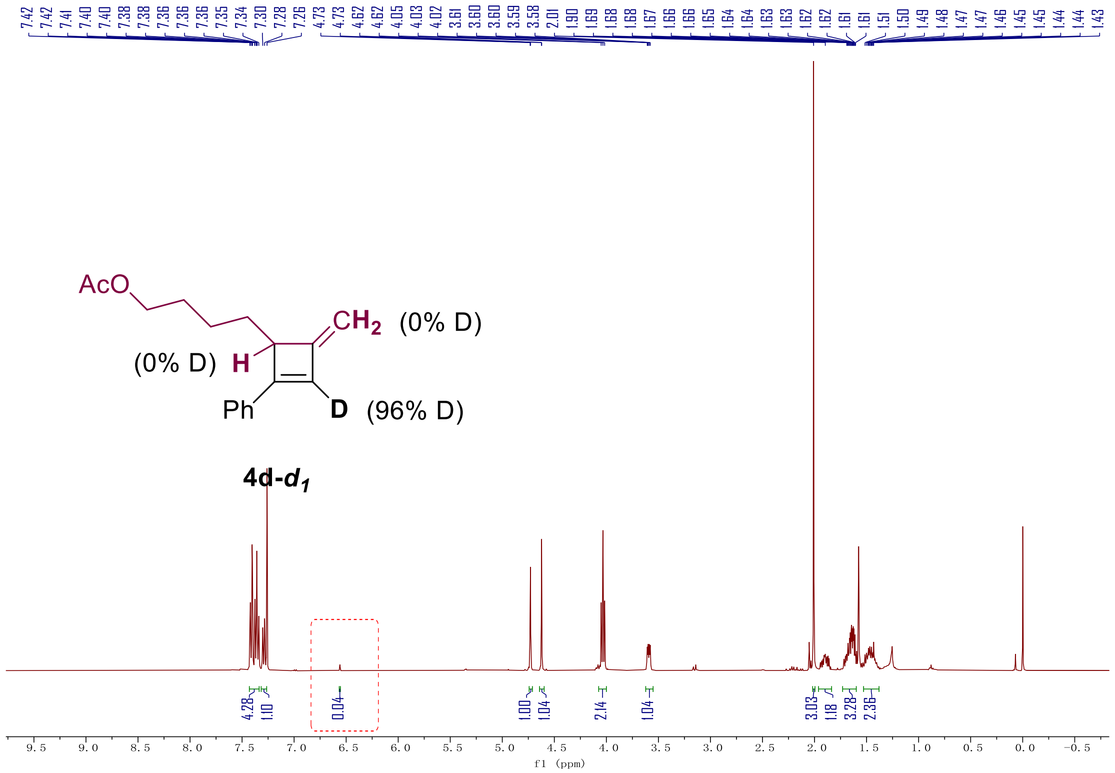

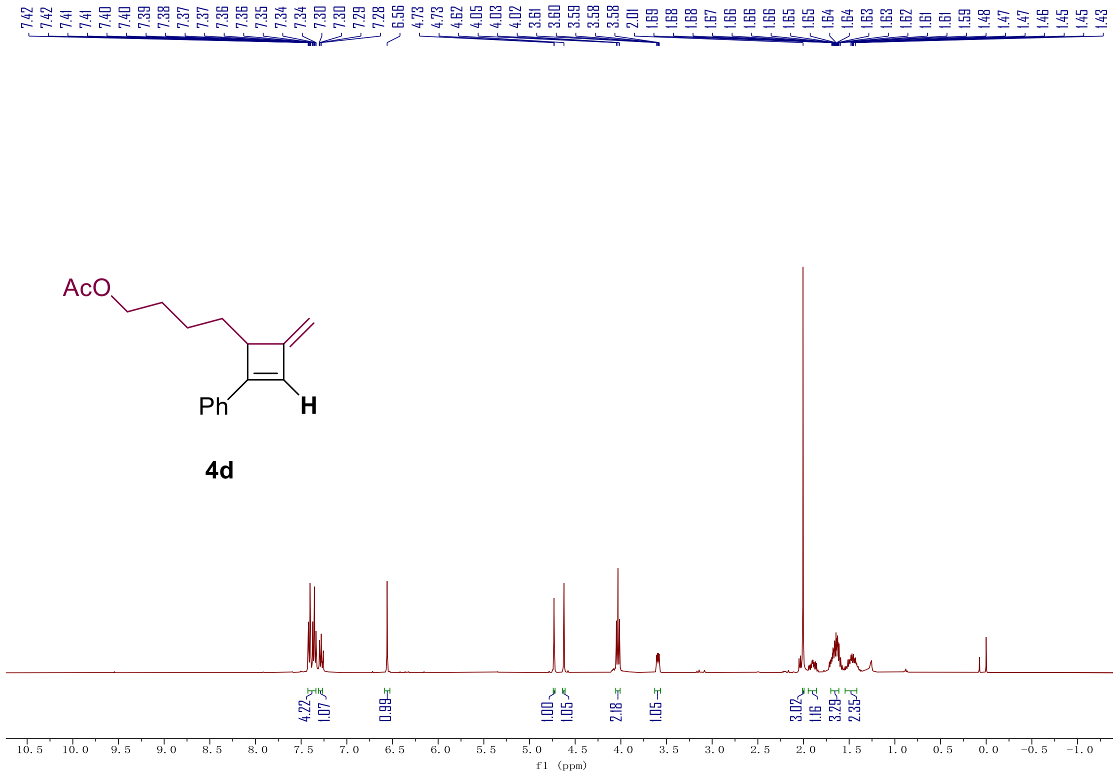

(2) Crossover experiment

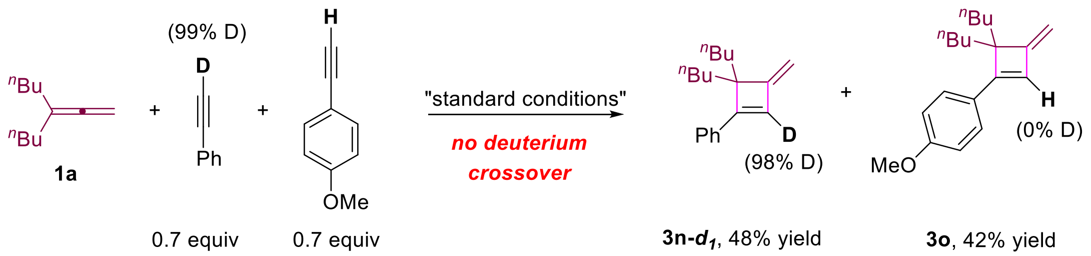

In a glove box, to an oven-dried 4-mL vial charged with allene **1a** (15.2 mg, 0.1 mmol, 1.0 equiv), deuterated phenylacetylene (7.2 mg, 0.07 mmol, 0.7 equiv), 1-ethynyl-4-methoxybenzene (9.3 mg, 0.07 mmol, 0.7 equiv) and THF (1.0 mL), was added $[CpRu(MeCN)_3]PF_6$ (2.2 mg, 5.0 μmol, 5.0 mol%). The vial was capped and removed from the glove box. The reaction mixture was stirred at room temperature for 6 h. Then, the mixture was filtered through a short pad of silica gel. The filtrate was concentrated *in vacuo*. Subsequently, CH_2Br_2 (0.1 mmol) was added as an internal standard, and the yields of **3n-*d*₁** and **3o** were determined to be 48% and 42%, respectively,by ^{1}H NMR sepectroscopy. The

crude product was purified by silica gel flash column chromatography to give the pure products **3n-*d*1** (eluent: *n*-hexane, 98%D) and **3o** (eluent: *n*-hexane/ethyl acetate = 50:1, 0%D) and their deuterium content was determined carefully by ^{1}H NMR sepectroscopy.

(4,4-Dibutyl-3-methylenecyclobut-1-en-1-yl-2-*d*)benzene (3n-*d*1).

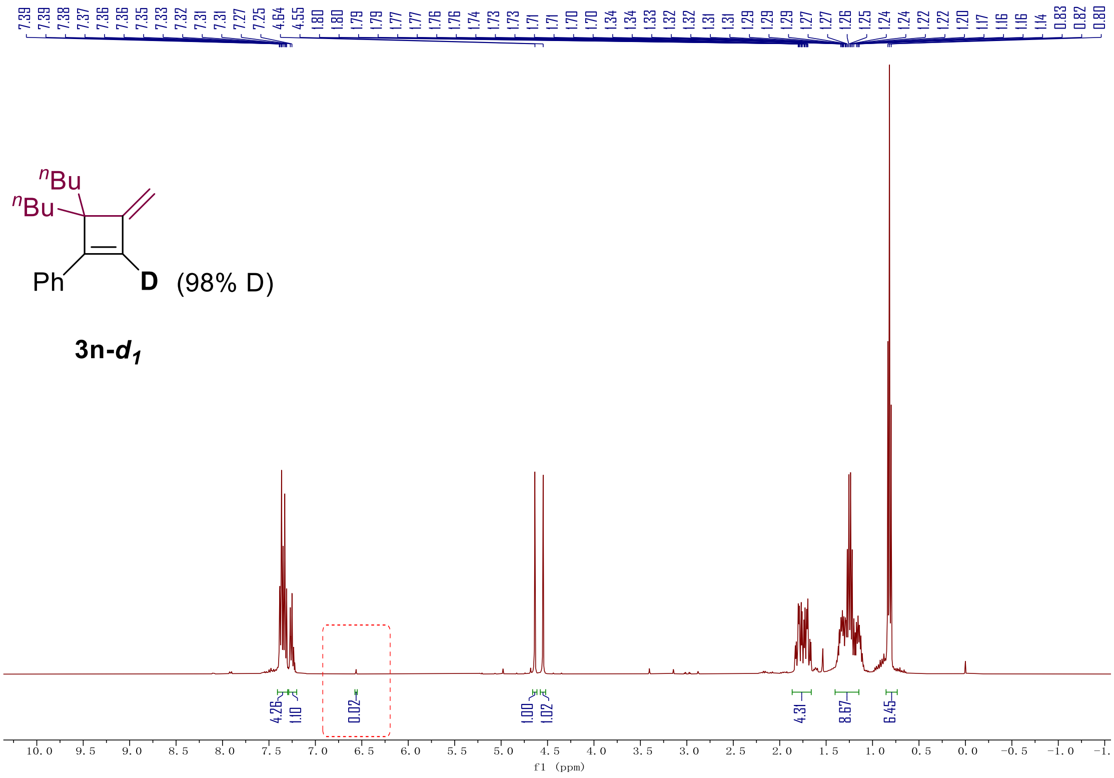

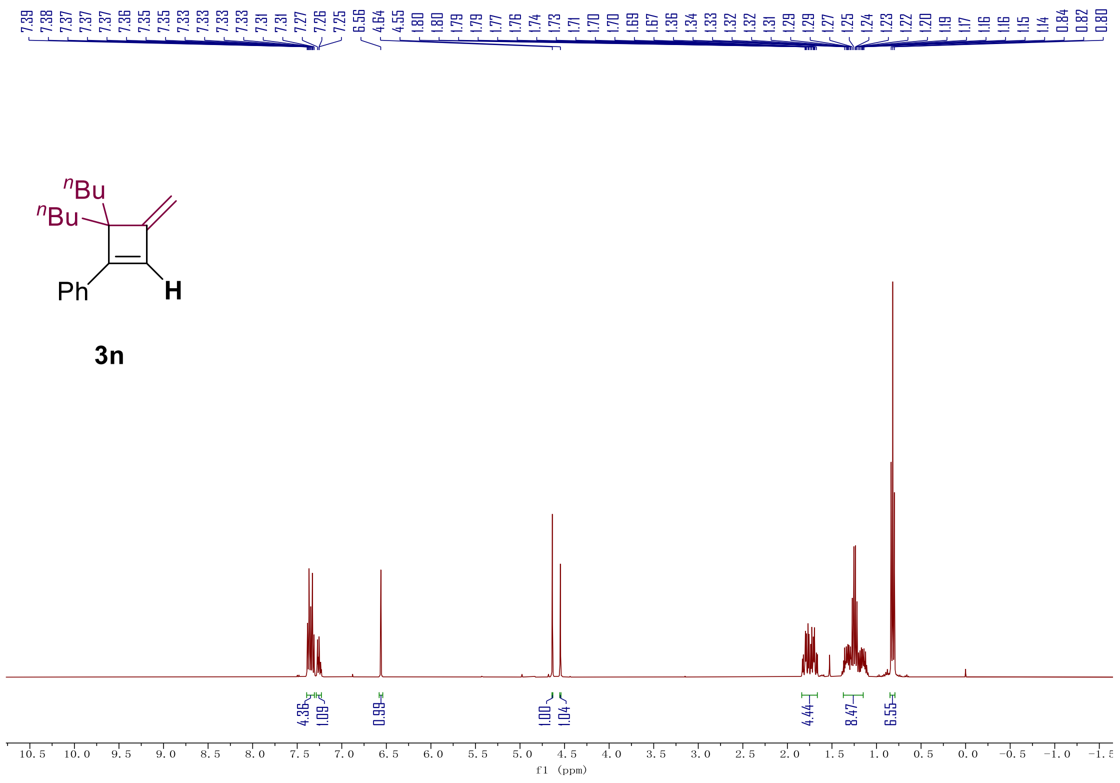

(3) Effect of substituents on regioselectivity

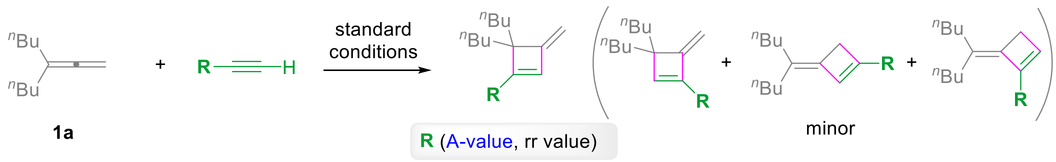

entry	R	A-value	rr value[a]
1	^{n}Bu	1.75	63
2	^{i}Pr	2.15	45
3	Me_3Si	2.5	38
4	Ph	3.0	32
5	^{t}Bu	4.5	2

[a]The rr value stands for the regioisomeric ratio.

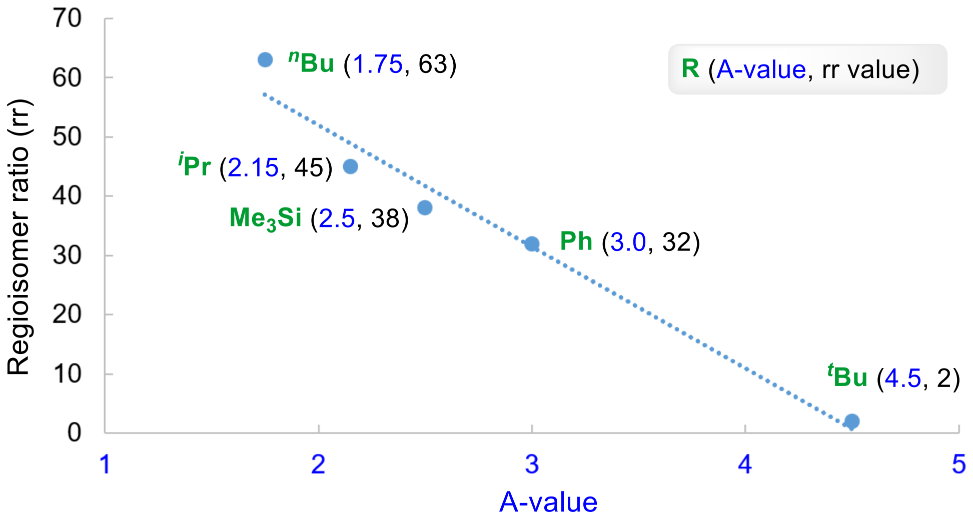

Figure S3. A plot of the relationship between A-value and regioisomer ratio.

(4) Kinetic experiments

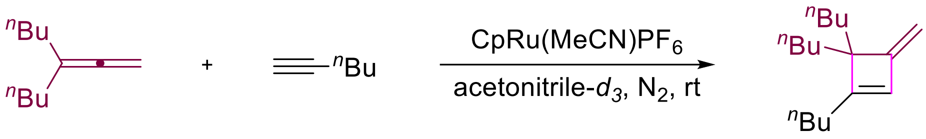

Procedures for the Kinetic Experiments

(a) Reaction order in catalyst.

In a glove-box, to each of the four NMR tubes were added an aliquot of the catalyst $CpRu(MeCN)_3PF_6$ (1.1 mg, 1.9 mg, 2.7 mg, and 3.4 mg, corresponding to 3.0 mol% to 9.0 mol% of the catalyst for each tube), allene **1a** (11.4 mg, 0.075 mmol, 1.0 equiv), alkyne **2a** (8.7 mg, 0.105 mmol, 1.4 equiv), CH_2Br_2 (0.10 mmol, 7.0 μL, internal standard), and MeCN-d_3 (0.75 mL). Then the NMR tubes were sealed with PTFE septa. The NMR tubes were taken out from the glove-box and immediately subjected to ^1H NMR analysis. The reaction progress was monitored by ^1H NMR over time.

Table S2. Reaction Progress at Different $CpRu(MeCN)_3PF_6$ Loadings

Entry	Time /min	[Ru] = 0.003 M 3 mol%	[Ru] = 0.005 M 5 mol%	[Ru] = 0.007 M 7 mol%	[Ru] = 0.009 M 9 mol%
		Concentration of product 3a/M			
1	10			0.0083	0.0085
2	15	0.0029	0.0091	0.0010	0.0114
3	20	0.0045	0.0110	0.0130	0.0176
4	25	0.0051	0.0130	0.0167	0.0205
5	30	0.0064	0.0145	0.0197	0.0248
6	35	0.0071	0.0160		
7	40	0.0080	0.0178		

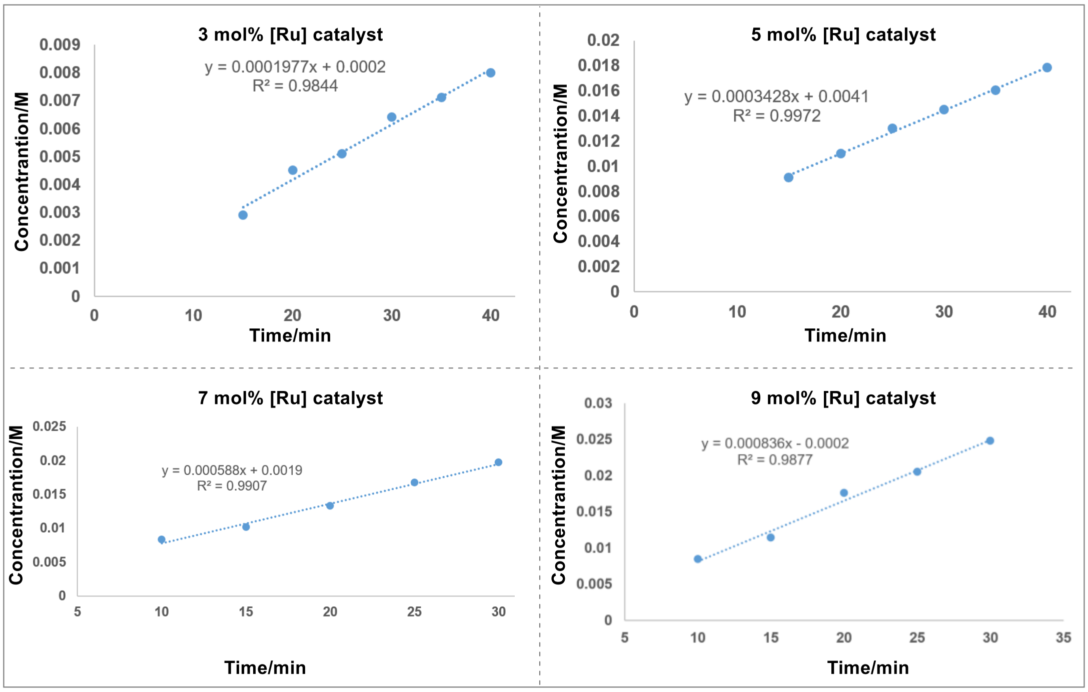

Figure S4. Plots of concentrations of **3a** *vs* time at different catalyst loadings.

Table S3. Initial Rate Dependence on Catalyst Loading

Entry	Catalyst loading	[Ru]/mM	Initial rate/M·min^{-1}	Initial rate/M·s^{-1}
1	3%	3	1.977E-04	3.295E-06
2	5%	5	3.428E-04	5.713E-06
3	7%	7	5.880E-04	9.800E-06
4	9%	9	8.360E-04	13.933E-06

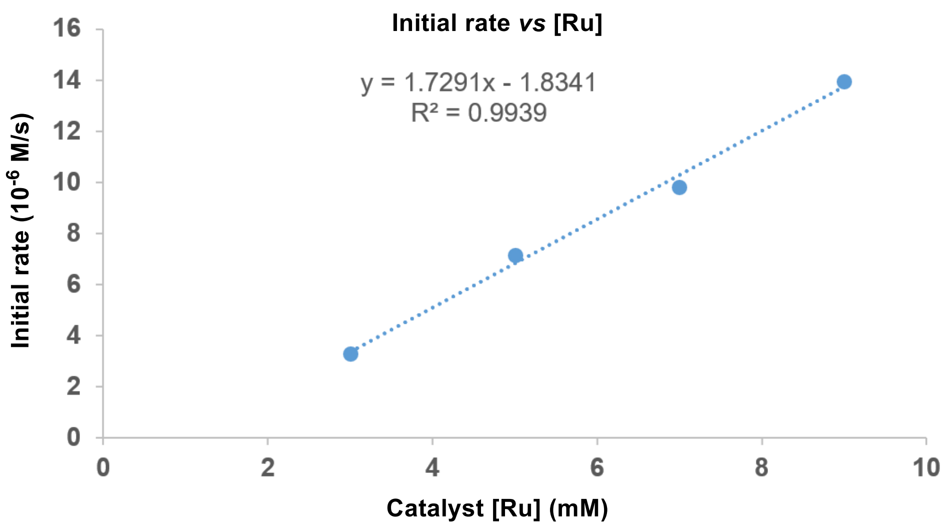

Figure S5. Plot of initial rates *vs* catalyst loading.

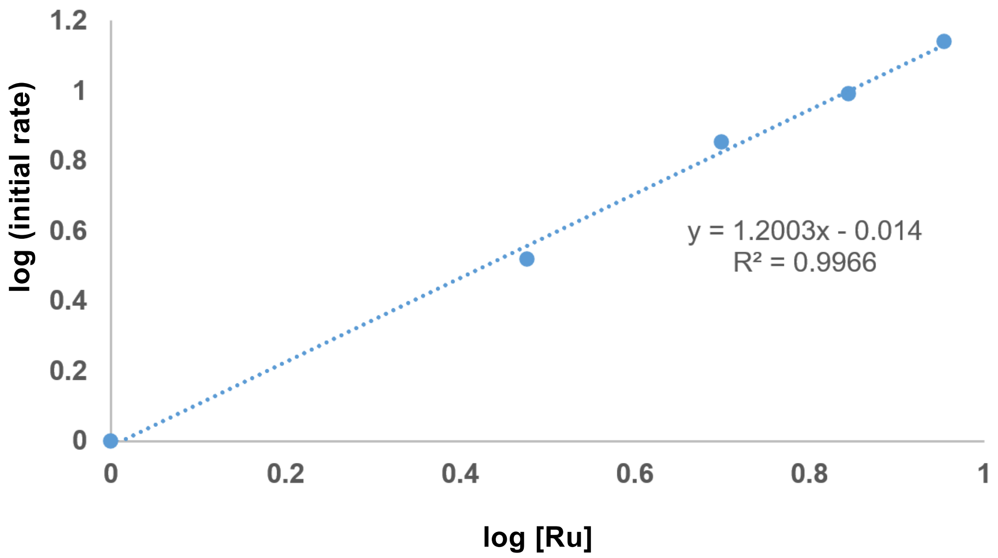

Figure S6. First-order kinetics in catalyst.

(b) Reaction order in allene 1a.

In a glove-box, to each of the four NMR tubes were added an aliquot of the allene **1a** (5.7 mg, 11.4 mg, 22.8 mg and 34.2 mg, corresponding to 0.5 equiv to 3.0 equiv of **1a** for each tube), alkyne **2a** (6.0 mg, 0.075 mmol, 1.0 equiv), CH_2Br_2 (0.10 mmol, 7.0 μL, internal standard), $CpRu(MeCN)_3PF_6$ (1.9 mg, 5.0 mol%) and MeCN-d_3 (0.75 mL). Then NMR tubes were sealed with PTFE septa. Then the NMR tubes were taken out from the glove-box and immediately subjected

to ^{1}H NMR analysis. The reaction progress was monitored by ^{1}H NMR over time.

Table S4. Reaction Progress at Different Equivalents of Allene [1a]

Entry	Time /min	[1a] = 0.05 M	[1a] = 0.10 M	[1a] = 0.20 M	[1a] = 0.30 M
		Concentration of product/M			
1	15	0.0100	0.0051	0.0058	0.0055
2	20	0.0113	0.0074	0.0078	0.0071
3	25	0.0133	0.0090	0.0093	0.0087
4	30	0.0152	0.0097	0.0111	0.0099
5	35	0.0161	0.0110	0.0124	0.0109
6	40	0.0177	0.0126	0.0138	0.0122

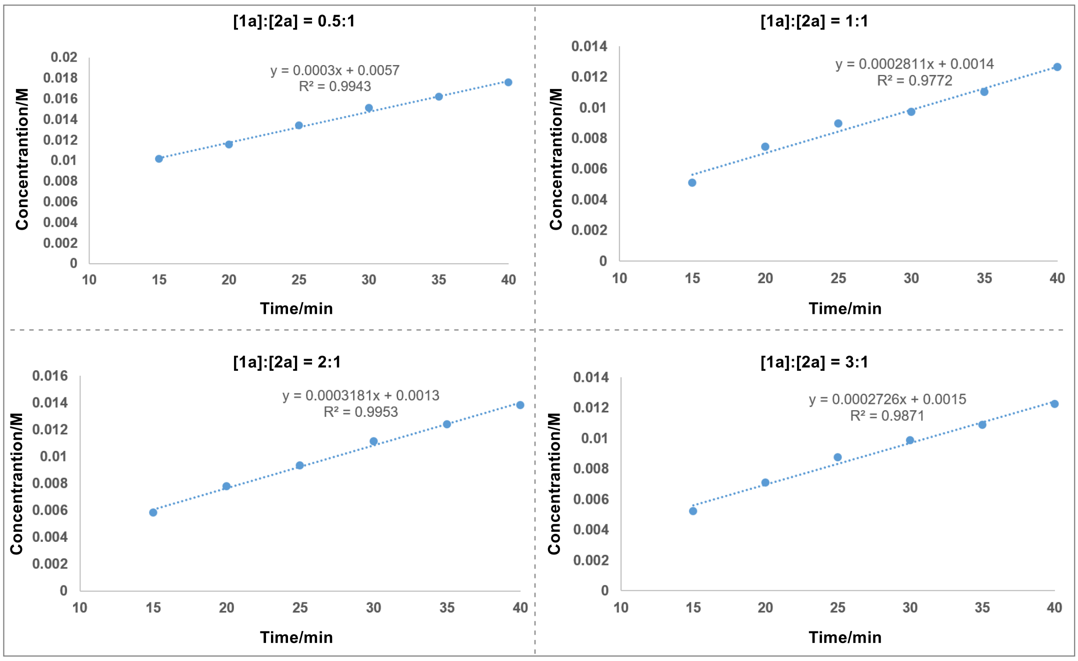

Figure S7. Plots of concentrations of **3a** *vs* time at different equivalents of allene **[1a]**.

Table S5. Initial Rate Dependence on Different Concentrations of Allene 1a

Entry	[1a]/M	Initial rate/M·min^{-1}	Initial rate/M·s^{-1}
1	0.05	3.000E-04	5.000E-06
2	0.10	2.811E-04	4.685E-06
3	0.20	3.181E-04	5.302E-06
4	0.30	2.726E-04	4.543E-06

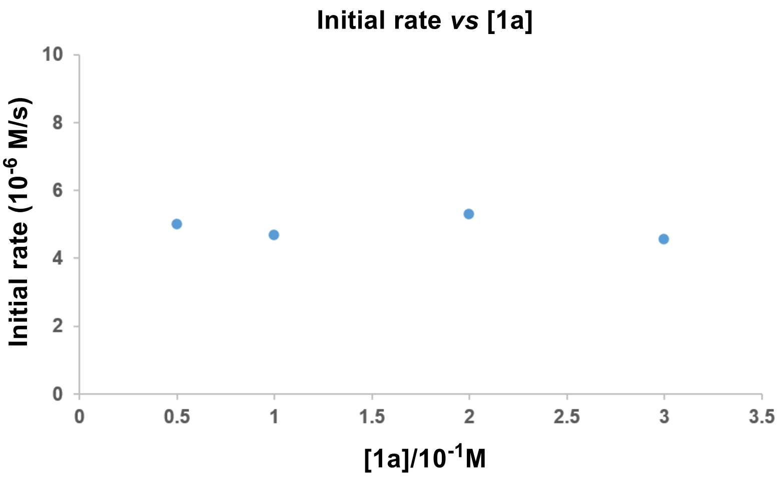

Figure S8. Plot of initial rates *vs* concentration of allene **[1a]**.

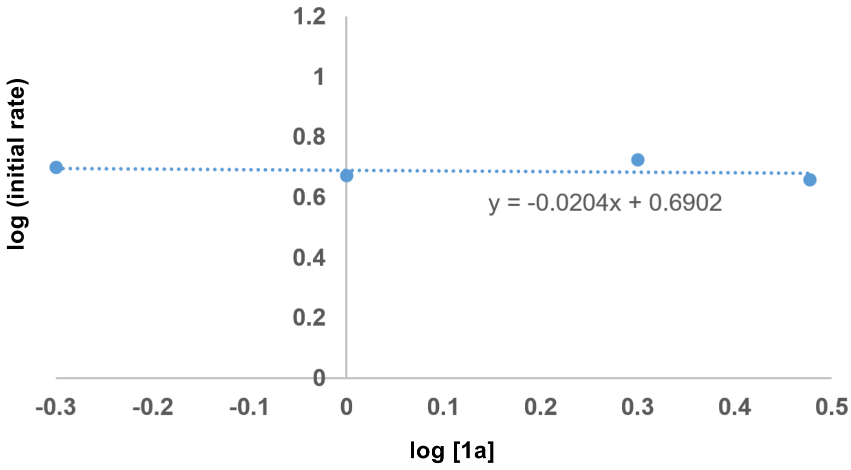

Figure S9. Zero-order kinetics in allene **1a**

(c) Reaction order in alkyne 2a.

In a glove-box, to each of the four NMR tubes were added an aliquot of the alkyne **2a** (8.7 mg, 12.0 mg, 18.0 mg and 24.0 mg, corresponding to 1.4 equiv to 3.0 equiv of **2a** for each tube), allene **1a** (11.4 mg, 0.075 mmol, 1.0 equiv), CH_2Br_2 (0.10 mmol, 7.0 μL, internal standard), $CpRu(MeCN)_3PF_6$ (1.9 mg, 5.0 mol%) and MeCN-d_3 (0.75 mL). Then NMR tubes were sealed with PTFE septa. Then the NMR tubes were taken out from the glove-box and immediately subjected to ^1H NMR analysis. The reaction progress was monitored by ^1H NMR over

time.

Table S6. Reaction Progress at Different Equivalents of Alkyne [2a]

Entry	Time /min	[2a] = 0.14 M	[2a] = 0.20 M	[2a] = 0.30 M	[2a] = 0.40 M
		Concentration of product/M			
1	15	0.0090	0.0102	0.0099	0.0095
2	20	0.0110	0.0115	0.0121	0.0112
3	25	0.0130	0.0134	0.0143	0.0132
4	30	0.0145	0.0151	0.0155	0.0142
5	35	0.0160	0.0162	0.0163	0.0160
6	40	0.0178	0.0176	0.0181	0.0175

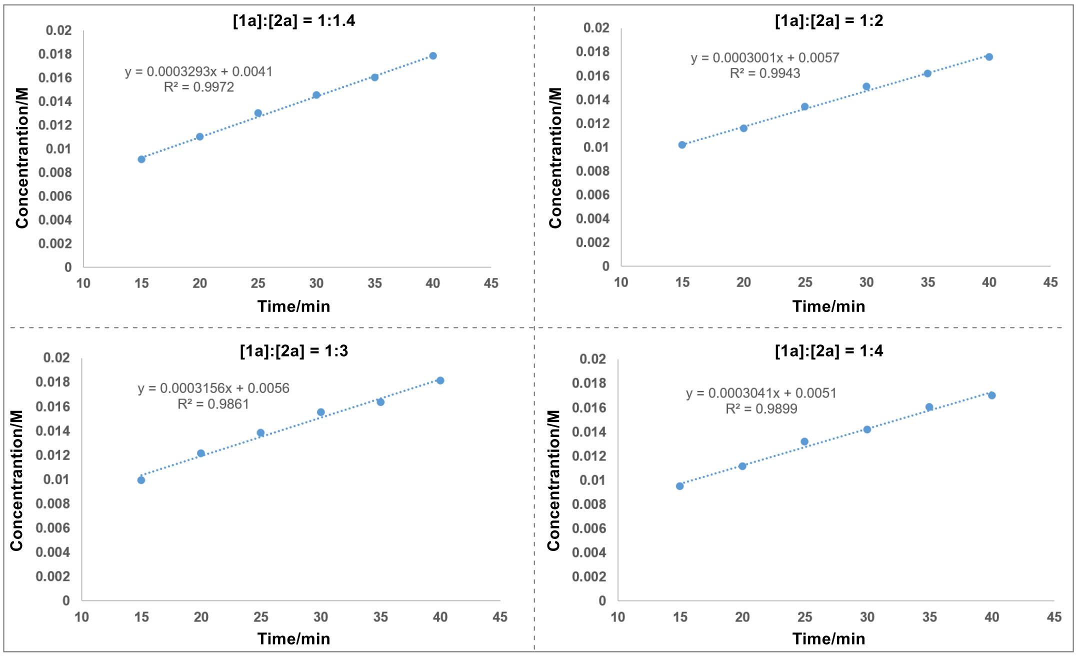

Figure S10. Plots of concentrations of **3a** *vs* time at different equivalents of alkyne **[2a]**.

Table S7. Initial Rate Dependence on Different Concentrations of Alkyne 2a

Entry	[2a]/M	Initial rate/M·min^{-1}	Initial rate/M·s^{-1}
1	0.14	3.293E-04	5.488E-06
2	0.20	3.001E-04	5.002E-06
3	0.30	3.156E-04	5.260E-06
4	0.40	3.041E-04	5.068E-06

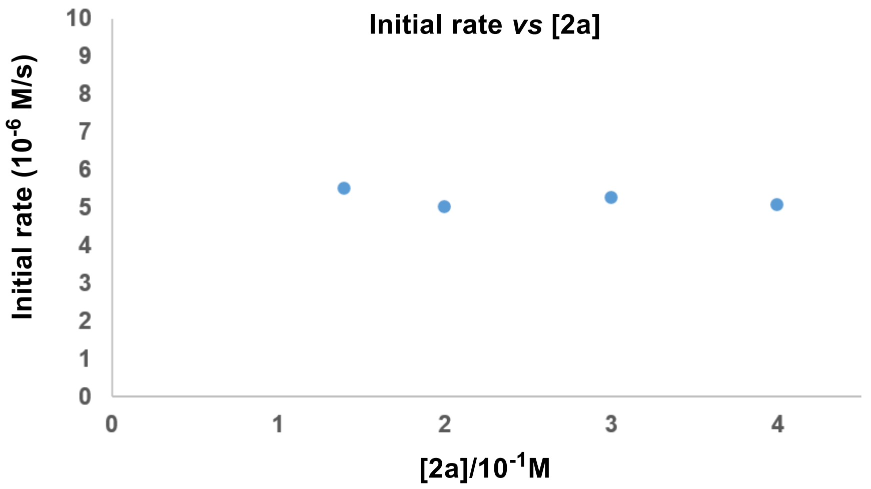

Figure S11. Plot of initial rates *vs* concentration of alkyne **[2a]**.

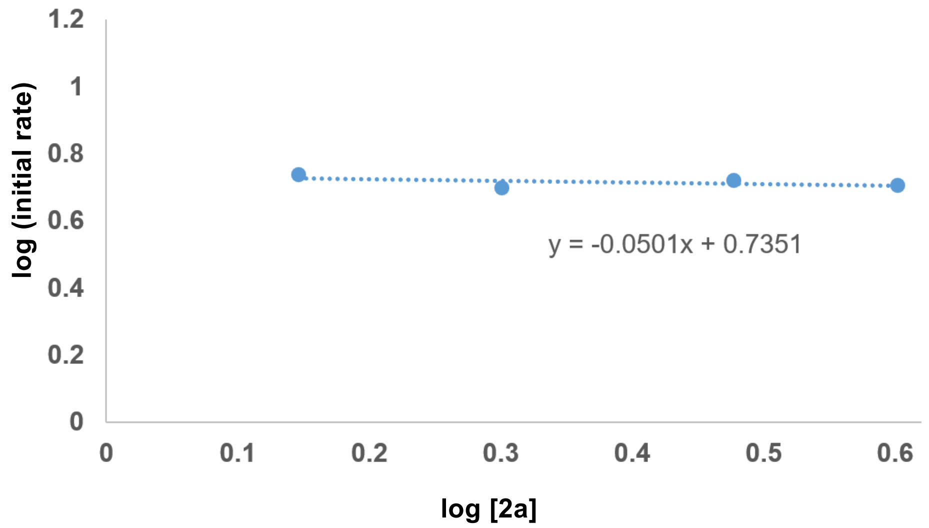

Figure S12. Zero-order kinetics in alkyne **2a.**

VI. Product Structure Determination

The structure of product **3ag** was determined by X-ray crystallography. The X-ray data have been deposited at the Cambridge Crystallographic Data Center (CCDC 2480545). The structures of other products were assumed by analogy.

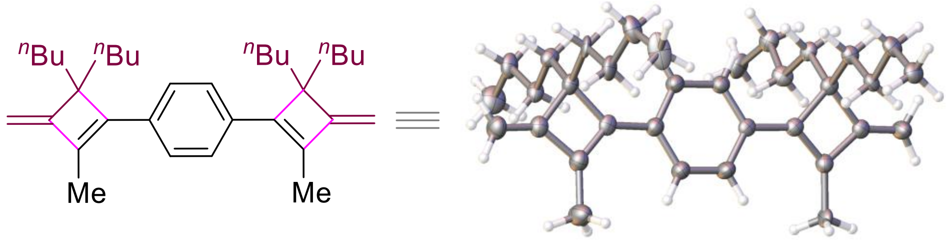

3ag CCDC 2480545

Table S8. Crystal Data and Structure Refinement for 3ag

Identification code	**3ag**
Empirical formula	$C_{34}H_{50}$
Formula weight	458.74
Temperature/K	99.98(10)
Crystal system	monoclinic
Space group	I2/m
a/Å	13.3882(10)
b/Å	15.9222(11)
c/Å	28.670(2)
α/°	90
β/°	101.089(7)
γ/°	90
Volume/Å^3	5997.5(8)
Z	8
ρ_{calc}g/cm^3	1.016

μ/mm^{-1}	0.056
F(000)	2032.0
Crystal size/mm^3	0.185 × 0.15 × 0.065
Radiation	MoKα (λ = 0.71073)
2Θ range for data collection/°	4.826 to 52.98
Index ranges	-16 ≤ h ≤ 16, -19 ≤ k ≤ 16, -34 ≤ l ≤ 35
Reflections collected	20464
Independent reflections	6283 [R_{int} = 0.0631, R_{sigma} = 0.0737]
Data/restraints/parameters	6283/832/625
Goodness-of-fit on F^2	1.045
Final R indexes [I>=2σ (I)]	R_1 = 0.0937, wR_2 = 0.2629
Final R indexes [all data]	R_1 = 0.1819, wR_2 = 0.3113
Largest diff. peak/hole / e Å^{-3}	0.33/-0.39

Experimental procedure for sample preparation and data collection:

Single crystals were grown in a solution of the sample **3ag** (~15 mg) in *n*-hexane/THF (0.5 mL/0.01 mL) in a 4.0-mL vial, which is open and placed under N_2 atmosphere at room temperature. A suitable crystal was selected and mounted with a Mitegen LoopTM on a SuperNova, Dual, Atlas diffractometer. The crystal was kept at 99.98(10) K during data collection. Using Olex2,[23] the structure was solved with the SHELXT structure solution program[24] using Intrinsic Phasing and refined with the SHELXL refinement package using Least Squares minimisation.[25]

VII. Computational Studies

Computational Details

All structures were optimized and characterized in experimental solvent (tetrahydrofuran) with the SMD[26] solvent model (SCRF = SMD) at M06[27]/BSI level, BSI represents a basis set with SDD[28] for Ru and 6-31G(d,p) for other atoms. Harmonic frequency analysis calculations at the same level were performed to verify the optimized geometries to be minima (no imaginary frequency) or transition states (TSs, having unique one imaginary frequency). The energies were further improved by M06/BSII//M06/BSI single-point calculations with solvent effects accounted by the SMD solvent model, using tetrahydrofuran, BSII denotes a basis set with SDD for Ru and 6-311++G(d,p) for other atoms. When necessary, intrinsic reaction coordinate (IRC) calculations[29] were carried out at the M06 /BSI level to verify a transition state that correctly connects with its nearby minima (reactant and product). All DFT calculations were carried out using the Gaussian 09 program on the HPC cluster at Hong Kong University of Science and Technology.[30] All energies discussed are Gibbs free relative energies at 298.15 K and 1 atm in kcal/mol. The 3D structures of selected key transition states and intermediates were generated by CYLview 2.0.[31]

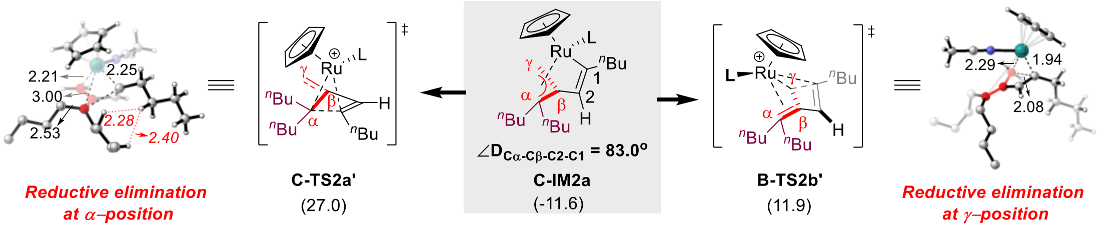

Figure S13. The detailed structural and free energy analysis of reductive elimination of **C-IM2a** at the α- and γ -positions. The selected bond lengths in the structures are labelled in Å. From **C-IM2a**, direct reductive elimination at the α-position is kinetically inaccessible, with a barrier as high as 38.6 kcal/mol (**C-TS2a'** relative to **C-IM2a**). Alternatively, reductive elimination at the γ -position via **B-TS2b′** is also disfavored, which is 9.9 kcal/mol higher than **C-TS4a**.

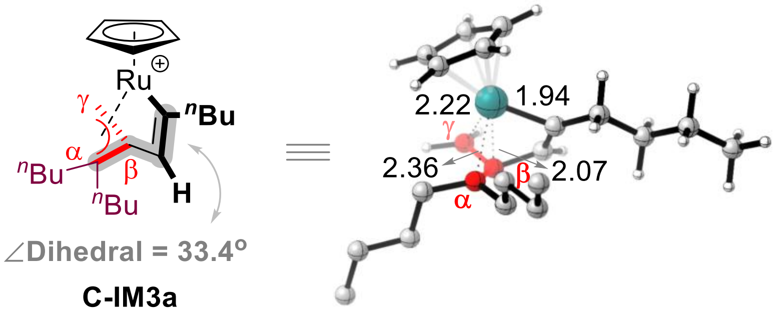

Figure S14. The detailed structural analysis of the key π-allyl intermediate **C-IM3a**. The selected bond lengths in the structures are labelled in Å.

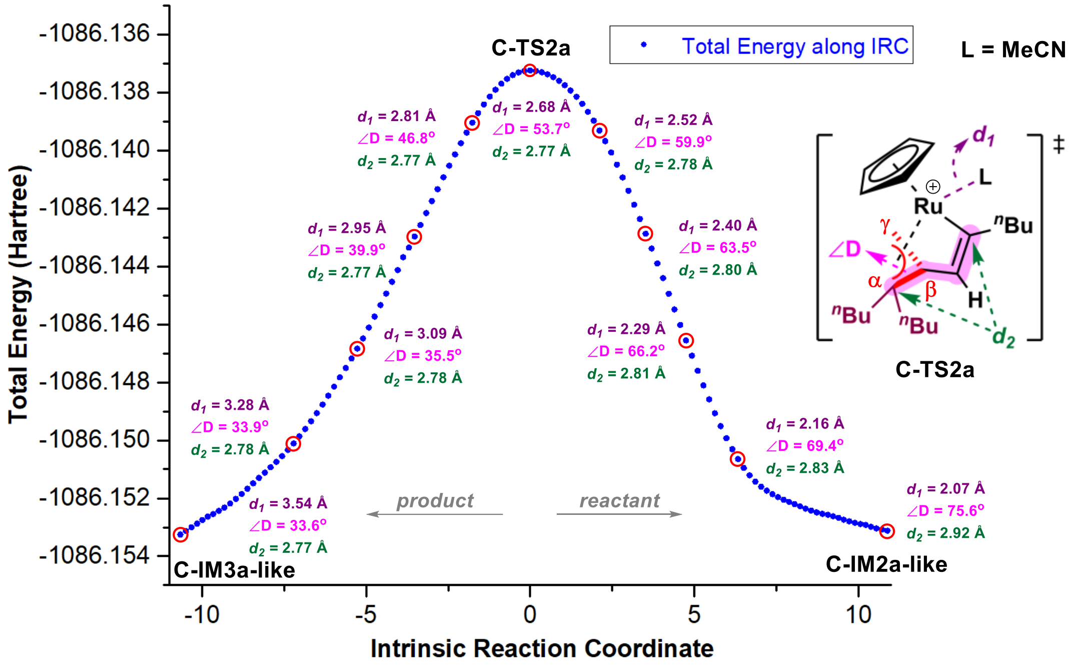

Figure S15. Intrinsic reaction coordinate (IRC) results for confirming the right connections of the ligand dissociation transition state **C-TS2a** with reactant and product (stepsize = 8). Remarkably, the detailed structural analysis demonstrates that the indicated dihedral angle (∠**D**) gradually decreases from the reactant (75.6° in **C-IM2a-like**) to the product (33.6° in **C-IM3a-like**) and the alkyne part gradually approaches the C_α of allene moiety (*d_2*), as the ligand (**L**) gradually moves away from the ruthenium center (*d_1*).

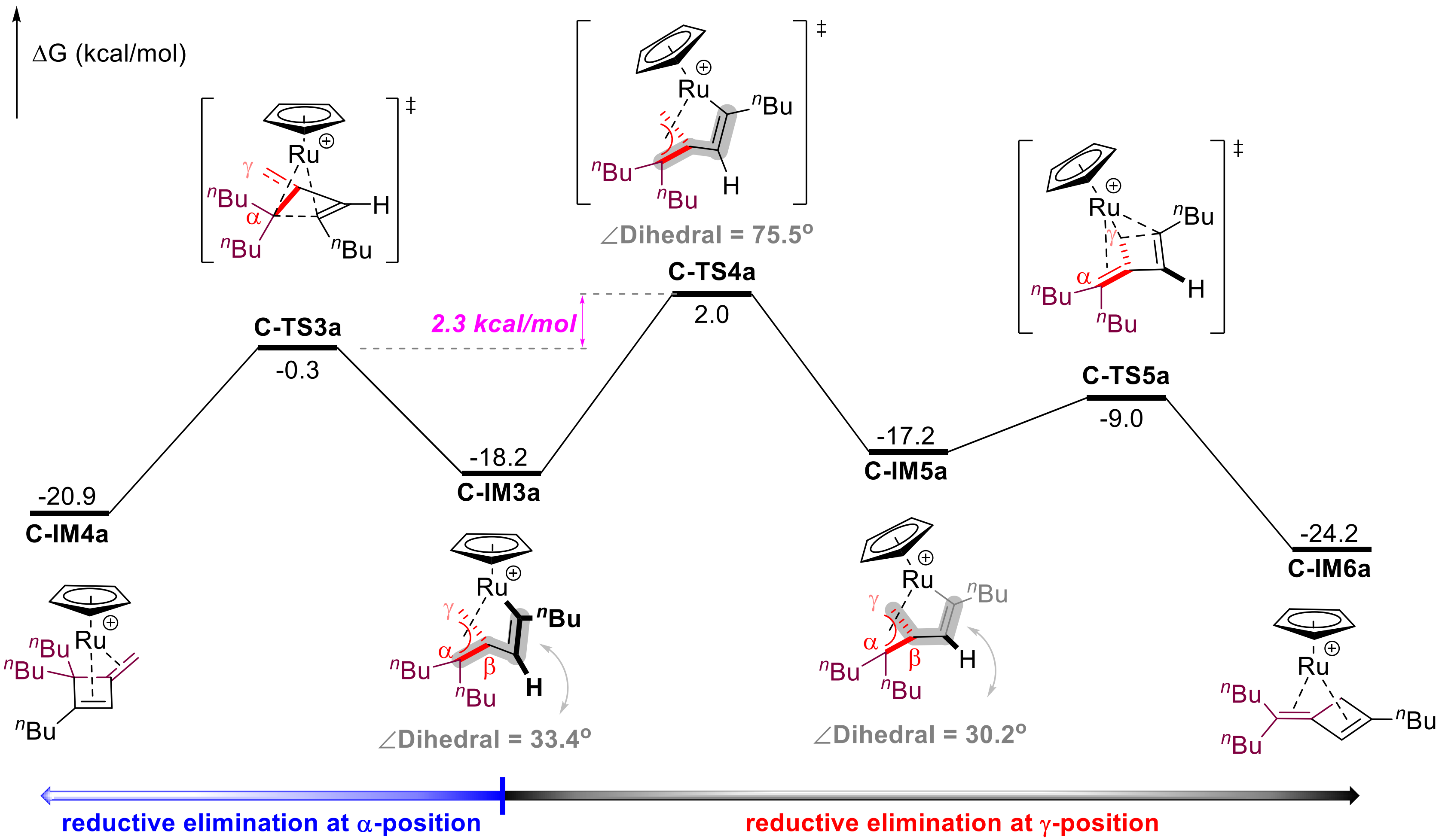

Figure S16. Gibbs-free energy (kcal/mol) for the energy barrier difference between the two regioselective pathways. The 2.3 kcal/mol barrier difference is sufficient to ensure a high regioselectivity at the α-position observed experimentally. Free energies are relative to allene **1a**, alkyne **2a**, MeCN and the catalyst $[CpRu(MeCN)_3]^+$, and are mass-balanced.

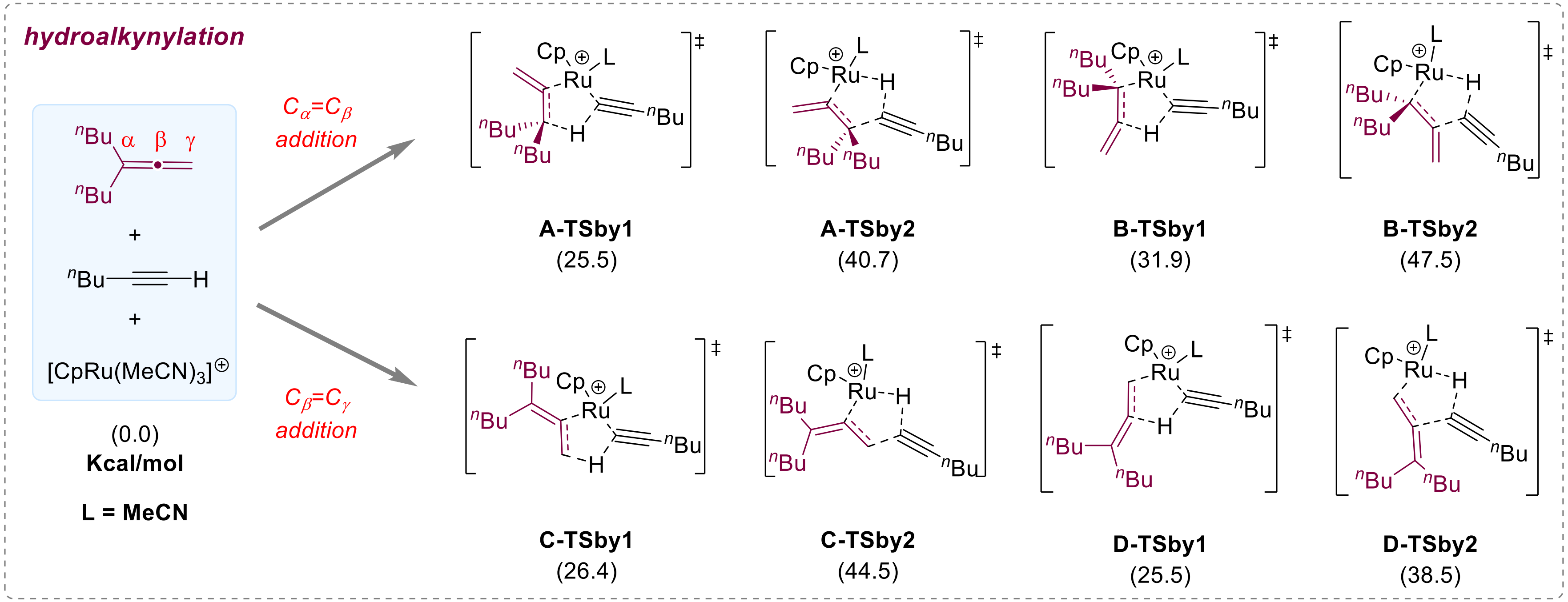

Figure S17. Gibbs-free energy (kcal/mol) for possible transition states for the hydroalkynylation of allene. The calculations indicate that the energy barrier of hydroalkynylation was too high to overcome (up to 25.5 kcal/mol – 47.5 kcal/mol), thus excluding this possibility. Free energies are relative to allene **1a**, alkyne **2a**, MeCN and the catalyst $[CpRu(MeCN)_3]^+$, and are mass-balanced.

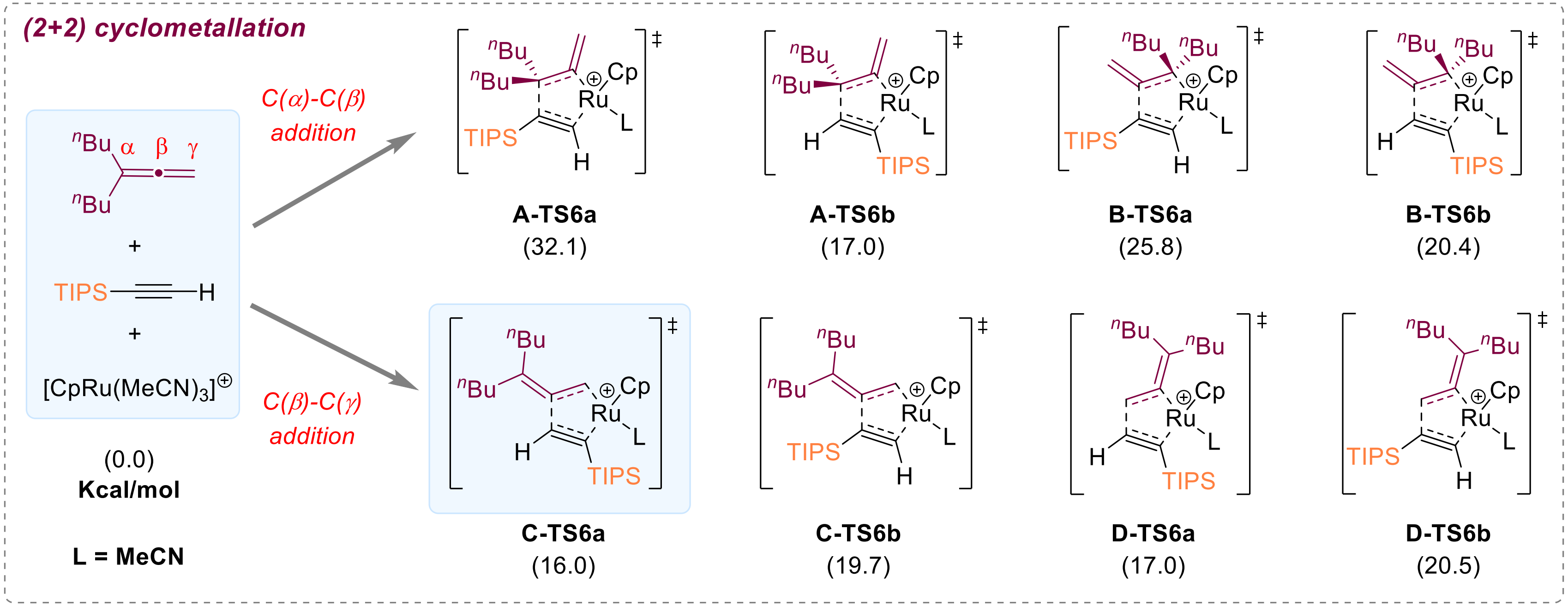

Figure S18. Gibbs-free energy (kcal/mol) for possible transition states for the oxidative cyclometallation of allene with TIPS-substituted acetylene. Free energies are relative to allene **1a**, TIPS-substituted acetylene, MeCN and the catalyst $[CpRu(MeCN)_3]^+$, and are mass-balanced.

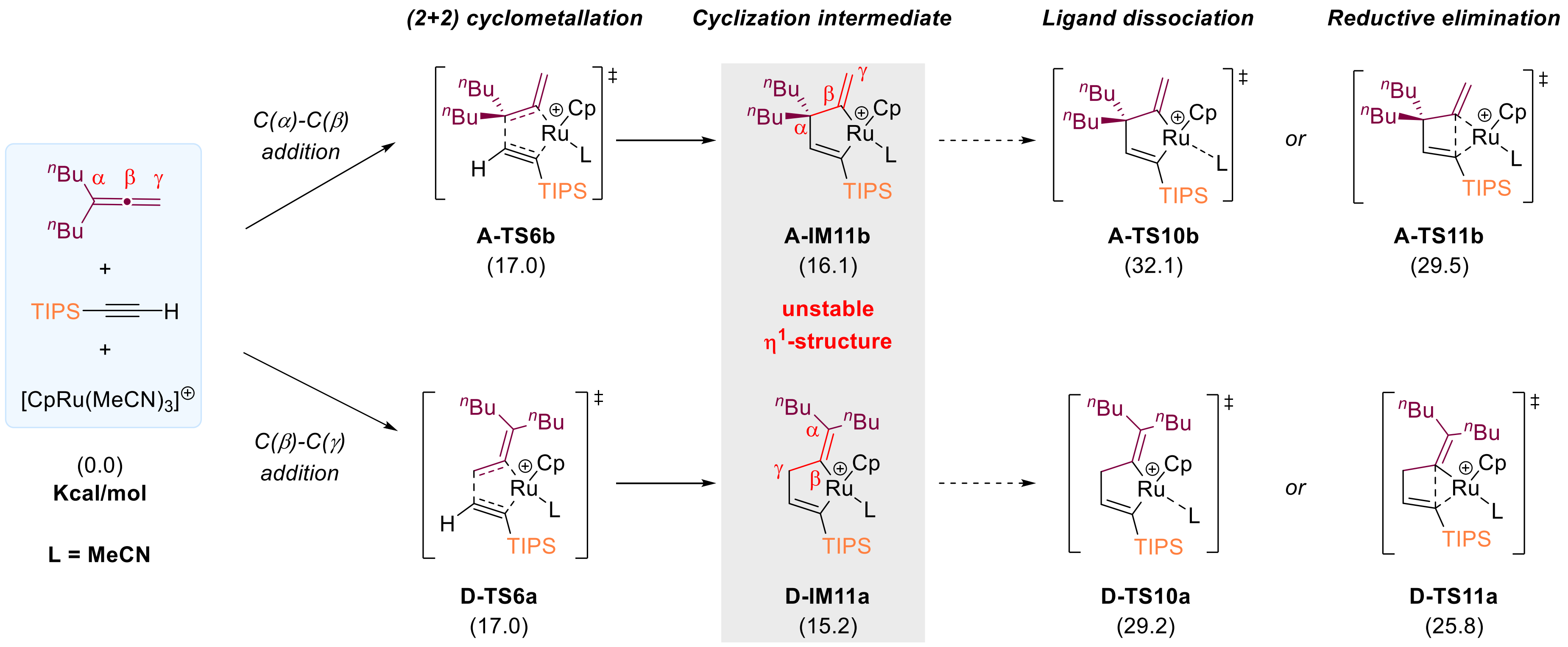

Figure S19. Gibbs-free energy (kcal/mol) for possible cycloaddition pathways from the oxidative cyclometallation transition states **A-TS6b** and **D-TS6a**. The cyclization intermediates **A-IM11b** and **D-IM11a** have high energy due to the unstable η^1-structures, resulting in inaccessible barriers (>25.8 kcal/mol) for subsequent ligand dissociation or reductive elimination. Free energies are relative to allene **1a**, TIPS-substituted acetylene, MeCN, and $[CpRu(MeCN)_3]^+$ are mass-balanced.

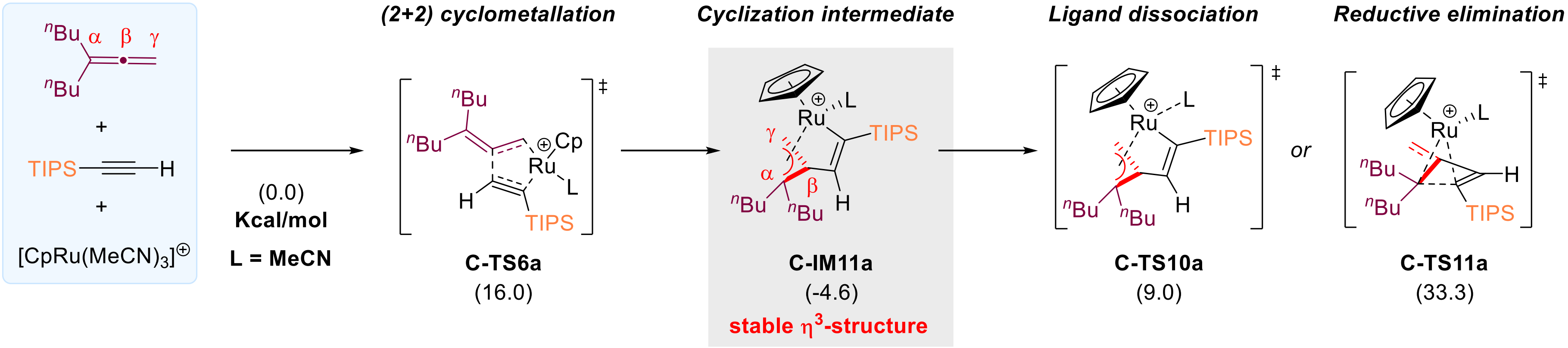

Figure S20. Gibbs-free energy (kcal/mol) for the cycloaddition pathway from the oxidative cyclometallation transition state **C-TS6a**. The pathway from **C-TS6a** leading to the formation of the stable η^3-struture **C-IM11a** has a relative energy of -4.6 kcal/mol and an accessible barrier for ligand dissociation (13.6 kcal/mol, **C-TS10a** relative to **C-IM11a**). Free energies are relative to allene **1a**, TIPS-substituted, MeCN, $[CpRu(MeCN)_3]^+$ are mass-balanced.

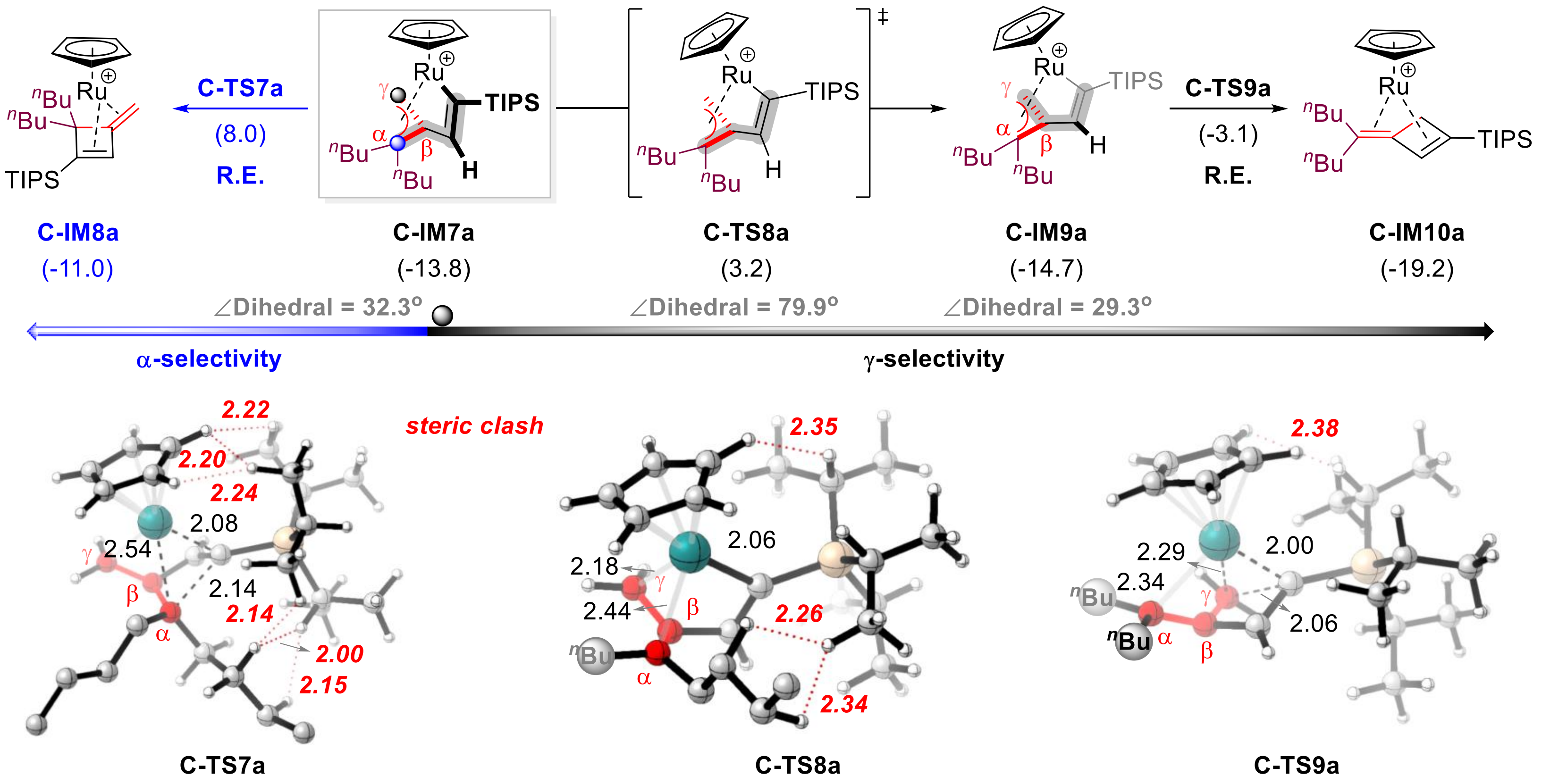

Figure S21. Gibbs-free energy (kcal/mol) for energetic comparison of the regioselectivity control of **C-IM7a** using TIPS-substituted terminal alkyne. R.E., reductive elimination. Free energies are relative to allene **1a**, TIPS-substituted acetylene, MeCN and the catalyst $[CpRu(MeCN)_3]^+$, and are mass-balanced. The selected bond lengths in the key structures are labelled in Å.

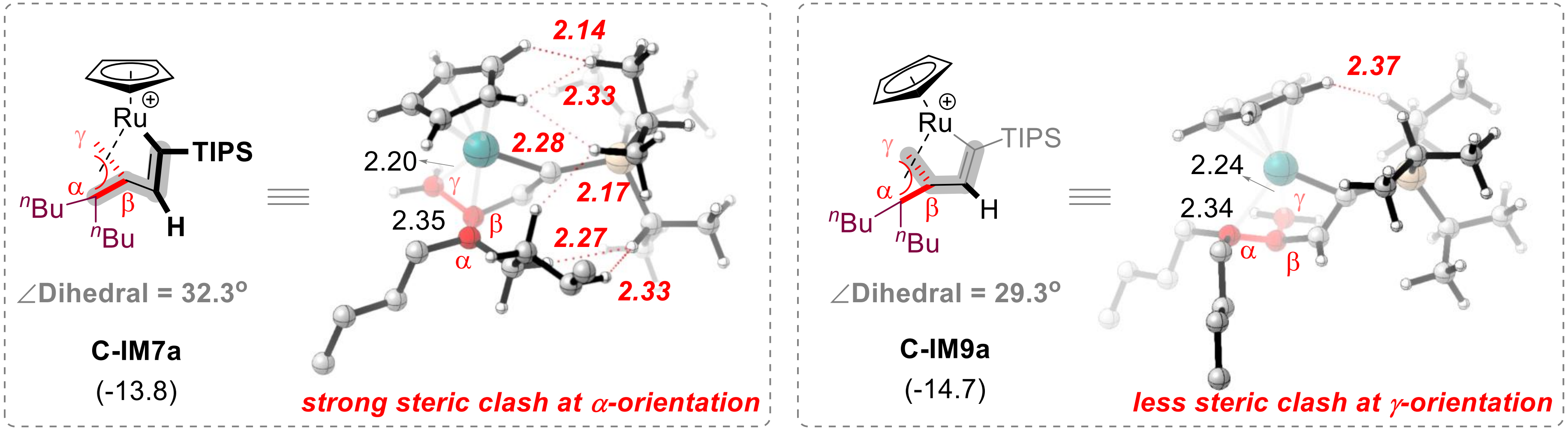

Figure S22. Structural comparision of the α-oriented **C-IM7a** and γ -oriented **C-IM9a**. Free energies (kcal/mol) are relative to allene **1a**, TIPS-substituted acetylene, MeCN and the catalyst $[CpRu(MeCN)_3]^+$, and are mass-balanced. The selected bond lengths in the key structures are labelled in Å.

Cartesian Coordinates in Å, SCF Energies and Free Energies (in a.u.) at 298.15 K and 1 atm for the Optimized Structures [BSI= 6-31G(d,p), BSII=6-311++G(d,p)]

$[CpRu(MeCN)_3]^+$

M06/BSI SCF energy in THF: -686.205869 a.u.

M06/BSII SCF energy in THF: -686.3463109 a.u.

M06/BSII free energy in THF: -686.1704309 a.u.

C	-2.34854600	0.90311200	-0.78282000
C	-2.32831100	1.05943600	0.64420800
C	-2.32838500	-0.25863000	1.20828900
C	-2.33325800	-1.21225800	0.15257500
C	-2.34683400	-0.48362500	-1.09220300
Ru	-0.54332500	0.00376000	0.00865500
C	1.42977700	2.29279900	-1.21600100
N	0.74990600	1.46705500	-0.77261100
C	2.27088700	3.32583600	-1.77229300
H	2.06852200	3.43774100	-2.84173700
H	3.32453600	3.06531700	-1.63488300
H	2.07281100	4.27845100	-1.27166300
H	-2.27847100	-0.49086800	2.26668600
H	-2.35552600	1.99410000	1.19061800
H	-2.32119700	1.71036800	-1.50678100
H	-2.35111200	-0.91585400	-2.08599700
H	-2.33526800	-2.28996700	0.26403600
C	1.48121300	-0.10725900	2.56276700
N	0.78257300	-0.06550400	1.64039600
C	2.34762800	-0.15904600	3.71612100

H	2.18131400	0.71924300	4.34732900
H	3.39401300	-0.17474900	3.39749400
H	2.13959400	-1.06060700	4.30035000
C	1.41645700	-2.19229400	-1.39814800
N	0.74264800	-1.39863100	-0.89150300
C	2.24817100	-3.18800700	-2.03143600
H	1.71967900	-4.14536100	-2.06808400
H	3.17625000	-3.31612800	-1.46666100
H	2.49246800	-2.87793500	-3.05187800

1a

M06/BSI SCF energy in THF: -430.82951419 a.u.

M06/BSII SCF energy in THF: -430.9316989 a.u.

M06/BSII free energy in THF: -430.690141 a.u.

C	-0.00013500	2.59724300	0.00002400
C	-0.00002800	1.28552700	0.00005100
C	-0.00031300	3.90420300	-0.00003000
H	-0.50050600	4.47724800	-0.78189700
H	0.49971000	4.47747700	0.78177300
C	1.09436400	0.51425000	-0.69971000
H	1.79691200	1.22202300	-1.16093600
H	0.65831600	-0.07026700	-1.52669900
C	-1.09431700	0.51409700	0.69980600
H	-0.65815600	-0.07052700	1.52665900
H	-1.79686500	1.22176400	1.16118800
C	1.85750000	-0.43210100	0.22254900
H	1.16927800	-1.18146500	0.64507000
H	2.25411300	0.13443200	1.08018100

C	2.99606200	-1.14602600	-0.48974200
H	3.68037200	-0.39861400	-0.91946000
H	2.59026700	-1.71007500	-1.34354200
C	3.76331700	-2.07939800	0.43032900
H	3.10257300	-2.84481600	0.85819500
H	4.57447500	-2.59798600	-0.09368000
H	4.20982900	-1.52865000	1.26858900
C	-1.85750500	-0.43213600	-0.22255100
H	-2.25435600	0.13459000	-1.07994900
H	-1.16926400	-1.18128800	-0.64541200
C	-2.99583200	-1.14640900	0.48975700
H	-3.68015200	-0.39919500	0.91981100
H	-2.58981200	-1.71063400	1.34333500
C	-3.76315700	-2.07960400	-0.43043200
H	-4.57415200	-2.59844100	0.09358100
H	-4.20989900	-1.52864200	-1.26842900
H	-3.10241300	-2.84480900	-0.85867800

2a

M06/BSI SCF energy in THF: -234.4083768 a.u.

M06/BSII SCF energy in THF: -234.4693509 a.u.

M06/BSII free energy in THF: -234.3607559 a.u.

C	-2.13268600	0.06556700	0.00033500
C	-0.83690200	0.73000600	-0.00032000
H	-0.76610700	1.38984800	0.87722100
H	-0.76647200	1.38923100	-0.87835100
C	-3.19832400	-0.50634800	-0.00002900
H	-4.14147900	-1.01074100	0.00047700

C	0.33812200	-0.24932800	-0.00022900
H	0.26536600	-0.90550500	-0.88011100
H	0.26487400	-0.90585800	0.87934400
C	1.67892700	0.46714400	0.00035200
H	1.73573000	1.12701100	0.87946900
H	1.73603500	1.12806800	-0.87795600
C	2.85108800	-0.49779700	-0.00004200
H	2.82977000	-1.14683900	-0.88517600
H	3.81236500	0.02842900	0.00129800
H	2.82856300	-1.14911000	0.88339000

L (MeCN)

M06/BSI SCF energy in THF: -132.6644433 a.u.

M06/BSII SCF energy in THF: -132.7025016 a.u.

M06/BSII free energy in THF: -132.6812196 a.u.

C	-0.27397000	0.00031400	0.00022700
N	-1.43513900	-0.00012800	-0.00009300
C	1.17334800	-0.00003800	-0.00003800
H	1.54938000	-0.76103900	-0.69019200
H	1.55009000	-0.21761500	1.00353000
H	1.55023500	0.97789100	-0.31381800

A-TSby1

M06/BSI SCF energy in THF: -1086.087578 a.u.

M06/BSII SCF energy in THF: -1086.311627 a.u.

M06/BSII free energy in THF: -1085.818205 a.u.

C	-3.14665300	0.68759400	-1.20153100

C	-3.66272900	-0.19618800	-0.20747600
C	-3.46315000	-1.53586300	-0.65003900
C	-2.85185500	-1.47348500	-1.95158400
C	-2.67830500	-0.10930400	-2.28787500
Ru	-1.48519000	-0.60975500	-0.42330400
C	0.19872000	0.10609600	-1.37289000
H	-3.74510000	-2.43834900	-0.11982500
H	-4.09356300	0.09962200	0.74256100
H	-3.14615300	1.77074400	-1.15994200
H	-2.19778900	0.26555900	-3.18426400
H	-2.55929500	-2.32213300	-2.55897500
C	0.16576300	-3.36093000	-0.12984100
N	-0.40711300	-2.36252200	-0.22931100
C	0.91405800	-4.58486100	0.01686400
H	1.78325500	-4.40167200	0.65768100
H	1.25852300	-4.93523100	-0.96042200
H	0.28913000	-5.35600900	0.47652400
C	1.24329000	0.48536300	-1.88718300
C	2.54501800	0.88008100	-2.40875400
H	2.80949900	0.24470800	-3.26748400
H	2.50919900	1.91012100	-2.79255200
C	3.62622900	0.76425900	-1.33050800
H	3.36216300	1.42290300	-0.48736900
H	3.62183800	-0.26191200	-0.92776200
C	5.01570200	1.11143500	-1.83655700
H	5.01272000	2.13690600	-2.23569600
H	5.26868300	0.45579600	-2.68346300
C	6.06409600	0.98050900	-0.74578600
H	6.09692900	-0.04402400	-0.35068100

H	7.06786300	1.22648500	-1.10980700
H	5.84448900	1.65003500	0.09651500
H	-0.25759000	0.55841600	-0.04898000
C	-0.59932800	0.53275600	1.42527500
C	-1.38939800	-0.62414200	1.65410000
C	-1.88612000	-1.40006600	2.59953300
H	-2.49198900	-2.27615300	2.37405500
H	-1.69611000	-1.17981600	3.65191300
C	0.88334400	0.53136500	1.80399200
H	1.40779900	1.29379800	1.20787300
H	0.89472600	0.90705400	2.84151400
C	-1.28954900	1.87546400	1.64321300
H	-2.36322700	1.76148100	1.44343700
H	-1.19778100	2.09111200	2.72101800
C	1.66272000	-0.76867900	1.73030300
H	1.17777000	-1.54294400	2.34489800
H	1.66530700	-1.14049800	0.69525200
C	-0.74405600	3.05123500	0.84577800
H	-0.73318800	2.80094600	-0.22872000
C	3.10244800	-0.57965900	2.18756900
H	3.57299400	0.20580300	1.57422000
H	3.11436500	-0.20585800	3.22238800
C	3.91230800	-1.86006900	2.08844200
H	4.95251100	-1.71091600	2.39928200
H	3.48769200	-2.64881500	2.72374800
H	3.92843500	-2.23996000	1.05717000
H	0.30148800	3.25246200	1.12360700
C	-1.56722000	4.31337700	1.06014600
H	-2.61317800	4.11492600	0.77754900

H	-1.58583100	4.55485400	2.13372100
C	-1.03468700	5.49597100	0.27114900
H	-1.03864300	5.28622900	-0.80653600
H	-1.63341300	6.39874100	0.43495800
H	-0.00026200	5.72888700	0.55509400

A-TSby2

M06/BSI SCF energy in THF: -1086.061098 a.u.

M06/BSII SCF energy in THF: -1086.284006 a.u.

M06/BSII free energy in THF: -1085.794031 a.u.

C	0.78095300	-2.67043300	-1.64515500
C	1.30606400	-2.97197500	-0.35282500
C	2.68489100	-2.59897000	-0.34230900
C	2.99892400	-2.09577600	-1.65332100
C	1.84264900	-2.17489400	-2.46385000
Ru	1.48397600	-0.79516800	-0.65953400
C	-0.32792900	0.30529800	-0.97841600
H	3.38260800	-2.71847400	0.47810600
H	0.74931600	-3.38517600	0.48128500
H	-0.24767200	-2.81349200	-1.95909300
H	1.76047200	-1.85736600	-3.49627500
H	3.96362900	-1.70871700	-1.96306400
C	3.59771100	1.59450200	-0.19525000
N	2.81698700	0.75902600	-0.36998600
C	4.54877600	2.65601700	0.02874000
H	4.01509900	3.57892900	0.27877400
H	5.14481700	2.82512800	-0.87290600
H	5.21494200	2.39587100	0.85651700

C	-1.33135200	0.75063600	-1.56089500
C	-2.52048000	1.29283500	-2.18078200
H	-2.55721200	2.38017500	-2.01220400
H	-2.43478900	1.15966000	-3.27207700
C	-3.82372500	0.64342000	-1.69847700
H	-3.76386100	-0.44510300	-1.84586100
H	-3.93219500	0.80738500	-0.61561400
C	-5.03551800	1.20337600	-2.42664000
H	-4.91063300	1.04707800	-3.50856800
H	-5.07481200	2.29293700	-2.27855800
C	-6.33114700	0.56748200	-1.95591300
H	-6.49029600	0.73817200	-0.88334400
H	-7.19899900	0.97357100	-2.48716600
H	-6.32114900	-0.51798700	-2.11818800
H	0.79851800	0.36529200	-1.67520800
C	-0.34242600	0.10284100	0.88816000
C	0.95317700	-0.39979400	1.27652700
C	1.57715600	-0.54060500	2.44026700
H	1.09427700	-0.28264300	3.38646700
H	2.59698300	-0.92070300	2.49884200
C	-0.66260800	1.52187300	1.32939500
H	-1.63498100	1.80154200	0.89485100
H	-0.80694000	1.44041800	2.42105800
C	-1.50583000	-0.87058900	1.05768400
H	-2.41852200	-0.37602100	0.69445000
H	-1.34532100	-1.75019400	0.42122000
C	0.36169300	2.60870800	1.05832800
H	1.29584300	2.37993100	1.59329600
H	0.60975100	2.63027100	-0.01466600

C	-1.70148200	-1.32778700	2.49932300
H	-0.82914600	-1.91504300	2.82296800
H	-1.75882000	-0.46122300	3.17630700
C	-2.96370500	-2.16453400	2.65616900
H	-2.92877400	-3.01142900	1.95397300
H	-3.83749800	-1.56343000	2.36288000
C	-3.13951400	-2.67631600	4.07473000
H	-4.05720600	-3.26475900	4.18479100
H	-2.29743800	-3.31458300	4.37200800
H	-3.18992300	-1.84632100	4.79155200
C	-0.14296100	3.98048500	1.48358800
H	-1.04883900	4.22708400	0.90904600
H	-0.45255600	3.94570200	2.53911600
C	0.90256400	5.06399200	1.29048200
H	0.52148200	6.05378900	1.56523700
H	1.78911600	4.86993200	1.90836600
H	1.23158700	5.11525200	0.24372700

B-TSby1

M06/BSI SCF energy in THF: -1086.073108 a.u.

M06/BSII SCF energy in THF: -1086.297155 a.u.

M06/BSII free energy in THF: -1085.808124 a.u.

C	0.66621900	-2.64728300	-1.47463300
C	-0.75701500	-2.57650000	-1.35548800
C	-1.12915500	-3.11218400	-0.08611600
C	0.06980800	-3.53022500	0.56900800
C	1.16684100	-3.26384600	-0.28300800
Ru	0.15189300	-1.29558900	0.17759100

C	2.02542200	-0.46919300	0.30931600
H	-2.13414300	-3.20381100	0.30946600
H	-1.42834600	-2.16910800	-2.10434600
H	1.25680100	-2.32414800	-2.32447800
H	2.21052700	-3.43104900	-0.04426600
H	0.13423000	-3.93854400	1.57144600
C	-0.44708000	0.46784000	-0.90892400
C	-0.09249700	1.04645800	-2.05624400
C	0.09621300	-0.77198600	3.36705800
N	0.06833600	-0.89873300	2.21854400
C	0.11393900	-0.59240800	4.79780700
H	1.12035900	-0.77681100	5.18483100
H	-0.58585300	-1.28777900	5.27019000
H	-0.17948600	0.43308400	5.04257700
C	-1.50408300	0.29196000	0.01536400
C	3.15459200	0.00236300	0.32226700
C	4.50550300	0.55143600	0.30055600
H	4.66657700	1.18684000	1.18406500
H	5.23446600	-0.26912600	0.38386600
C	4.79449500	1.35826000	-0.96731500
H	4.60729700	0.72668100	-1.84914200
H	4.08391800	2.19645500	-1.03294800
C	6.22033000	1.88428400	-1.00371300
H	6.91901200	1.03618000	-0.94181000
H	6.40497300	2.49435300	-0.10649200
C	6.50638600	2.69927100	-2.25227600
H	5.84435000	3.57249500	-2.31675700
H	7.53905200	3.06516400	-2.27256900
H	6.34910900	2.10162500	-3.15958200

H	0.75451000	0.25193900	-0.34684200
H	-0.74482100	1.76596300	-2.55011200
H	0.88937300	0.86890200	-2.49566700
C	-1.52817400	1.26738200	1.17541300
H	-2.02699000	0.80666100	2.04116600
H	-0.50205800	1.51574500	1.48188000
C	-2.87283100	-0.19590500	-0.44041600
H	-3.57845100	-0.00940600	0.38271100
H	-2.89452300	-1.27823700	-0.60407500
C	-3.38575000	0.47883700	-1.71191000
H	-2.74573000	0.19705200	-2.56305000
H	-3.31024200	1.57355500	-1.62396200
C	-4.82711100	0.09637200	-2.01415800
H	-4.90641100	-0.99990000	-2.07794000
H	-5.46415800	0.39778100	-1.16854900
C	-5.33698800	0.72812900	-3.29679100
H	-4.73394900	0.41760900	-4.16000500
H	-6.37675700	0.45054300	-3.50311000
H	-5.29219300	1.82365200	-3.24336600
C	-2.25571400	2.56298300	0.81405000
H	-3.30474700	2.35149400	0.55405400
H	-1.79405500	3.00562800	-0.08400400
C	-2.21773800	3.57425300	1.95136200
H	-2.65919600	3.12236000	2.85291400
H	-1.16943300	3.79577700	2.20286200
C	-2.95058500	4.85751200	1.60326600
H	-4.00785300	4.66129700	1.38273900
H	-2.91297500	5.58518300	2.42164500
H	-2.51397300	5.33408300	0.71600300

B-TSby2

M06/BSI SCF energy in THF: -1086.052288 a.u.

M06/BSII SCF energy in THF: -1086.277266 a.u.

M06/BSII free energy in THF: -1085.783142 a.u.

C	-2.26954200	-2.15995800	-1.91433000
C	-2.72265700	-0.80784500	-1.99584000
C	-3.60465500	-0.55327300	-0.91077800
C	-3.69028300	-1.75443000	-0.14645200
C	-2.90508500	-2.75852300	-0.78592100
Ru	-1.53962800	-1.10151900	-0.09864500
C	0.45628300	-1.56581400	-0.16562100
H	-4.11360300	0.37879000	-0.69540400
H	-2.40486000	-0.08951000	-2.74316500
H	-1.59649100	-2.65285800	-2.60622900
H	-2.79542400	-3.78504600	-0.45849600
H	-4.25622600	-1.88923400	0.76865300
C	-1.82652900	-0.42911500	3.03948600
N	-1.66546300	-0.63339000	1.91300100
C	-2.00907500	-0.16001600	4.44452100
H	-1.71363100	-1.03388800	5.03288700
H	-3.05921900	0.06975900	4.64812100
H	-1.39329900	0.69560000	4.73886700
C	1.56303600	-2.05689500	0.04836100
C	2.96790200	-2.41735000	0.16634400
H	3.28346300	-2.34315300	1.21904700
H	3.12533000	-3.46669100	-0.12311700
C	3.84916700	-1.50511700	-0.69401300

H	3.61900400	-1.67604300	-1.75676600
H	3.58599200	-0.45507200	-0.48670500
C	5.33104300	-1.72171300	-0.43547500
H	5.57255900	-2.78715800	-0.57091700
H	5.54639200	-1.49217800	0.62063200
C	6.20584700	-0.87193100	-1.33940600
H	6.00127300	0.19777500	-1.19718700
H	7.27197200	-1.03377100	-1.14466700
H	6.02216400	-1.10191500	-2.39687600
H	-1.10575100	-2.45942300	0.56234000
C	-0.37346300	0.93869600	-0.01865300
C	0.06296400	0.07216300	-1.03614700
C	0.48545900	0.12449000	-2.30054500
C	0.53030000	1.12078000	1.18570600
H	0.02611100	1.74566500	1.93736000
H	0.71381900	0.14607100	1.65150300
C	-1.31464700	2.07202900	-0.35063400
H	-1.90666800	1.81844100	-1.24019800
H	-2.02296800	2.20720600	0.48415000
C	-0.59017100	3.39544500	-0.60050700
H	-0.05340500	3.70777700	0.30758600
H	0.17603200	3.25453100	-1.38049000
C	-1.54807900	4.50098200	-1.01957100
H	-2.07097600	4.20002100	-1.93996900
H	-2.32762100	4.61339200	-0.25026100
C	-0.84044300	5.82712000	-1.23518800
H	-0.07478800	5.74572000	-2.01753200
H	-1.53774100	6.61702800	-1.53590700
H	-0.33706400	6.16077500	-0.31853400

C	1.90155300	1.71550400	0.82214800
H	2.18910500	1.39130100	-0.19135100
H	1.85128200	2.81322300	0.78475400
C	2.99074900	1.28630700	1.79589700
H	3.06919000	0.18611800	1.77410200
H	2.69421800	1.54882600	2.82326800
C	4.33963300	1.90160200	1.47035300
H	5.12313500	1.54926400	2.15109500
H	4.65172100	1.64949400	0.44698100
H	4.30638100	2.99658800	1.53848900
H	0.86106700	1.06949700	-2.69195100
H	0.54558000	-0.75521200	-2.93718500

C-TSby1

M06/BSI SCF energy in THF: -1086.086415 a.u.

M06/BSII SCF energy in THF: -1086.310225 a.u.

M06/BSII free energy in THF: -1085.816762 a.u.

C	1.70826600	-2.75204500	-1.42915700
C	0.68143900	-2.35717600	-2.33013700
C	-0.57612900	-2.67909100	-1.72886300
C	-0.32438200	-3.23941000	-0.45137000
C	1.09065800	-3.28354500	-0.24915800
Ru	0.52582700	-1.16824200	-0.46384600
C	2.28544700	-0.41983200	0.31376700
H	-1.55092900	-2.50018800	-2.16652600
H	0.82228500	-1.90272800	-3.30440100
H	2.77372700	-2.63289200	-1.58817500
H	1.60086500	-3.66852800	0.62529500

H	-1.07785500	-3.54662800	0.26599700
C	-0.71173100	-0.35489300	2.41040100
N	-0.25997100	-0.61261300	1.37811000
C	-1.28514000	-0.03963600	3.69631300
H	-0.50638800	-0.05348100	4.46502200
H	-2.05018800	-0.77986800	3.95095000
H	-1.74029700	0.95505900	3.66757000
C	3.33191000	-0.01816300	0.80683900
C	4.58107700	0.46023000	1.38394600
H	4.36995400	1.02643700	2.30336000
H	5.18988300	-0.40272700	1.69328800
C	5.39257500	1.33335900	0.42376700
H	5.61908700	0.75940700	-0.48709300
H	4.78080800	2.19108100	0.10617400
C	6.68271600	1.82874800	1.05712800
H	7.27992400	0.96391600	1.38351700
H	6.44258200	2.39394900	1.97039400
C	7.49691200	2.69339800	0.11145000
H	6.92813400	3.57687500	-0.20642300
H	8.42319200	3.04682800	0.57812700
H	7.77322800	2.13799300	-0.79414400
H	1.48741300	0.29160700	-0.66170300
C	0.35023000	0.86763500	-1.42104000
H	0.75140900	0.87157300	-2.43781300
H	0.57821000	1.77222000	-0.84411800
C	-0.88319300	0.23047100	-1.15105800
C	-2.20048600	0.38410000	-1.14762700
C	-3.14672600	-0.61465500	-0.54666400
H	-2.59429200	-1.51608400	-0.25053100

H	-3.87984500	-0.92753800	-1.30810600
C	-2.78863600	1.65219900	-1.73485600
H	-2.25086100	1.91705700	-2.65690000
H	-3.83278600	1.45793600	-2.02481500
C	-2.74158400	2.84582800	-0.78278200
H	-1.69389500	3.04899200	-0.50699700
H	-3.26173100	2.59932300	0.15551200
C	-3.35887100	4.09788900	-1.38806700
H	-2.82284800	4.35427700	-2.31455300
H	-4.39514900	3.87948600	-1.68796000
C	-3.33632000	5.27729000	-0.43224300
H	-2.30926300	5.52702500	-0.13515100
H	-3.77755800	6.17506200	-0.87977100
H	-3.89821900	5.05499600	0.48437200
C	-3.90454100	-0.08717100	0.67245600
H	-3.19411100	0.39187400	1.36748600
H	-4.61389900	0.69917600	0.37129400
C	-4.65002400	-1.19196400	1.40549500
H	-5.32042500	-1.70872100	0.70210600
H	-3.92255900	-1.94980300	1.74164100
C	-5.44258700	-0.67148500	2.59124200
H	-5.95898400	-1.47719300	3.12492300
H	-4.78928200	-0.16094700	3.31204000
H	-6.20117400	0.05361400	2.26988600

C-TSby2

M06/BSI SCF energy in THF: -1086.054752 a.u.

M06/BSII SCF energy in THF: -1086.277927 a.u.

M06/BSII free energy in THF: -1085.787972 a.u.

C	1.61784600	-3.10771600	-0.25139600
C	1.07569600	-2.42037700	-1.38603400
C	-0.34215600	-2.44636100	-1.27870400
C	-0.68000800	-3.07842800	-0.05208700
C	0.54404600	-3.50578100	0.58032500
Ru	0.49729300	-1.28953400	0.46033600
C	2.15780700	0.10761100	0.60483000
H	-1.04037400	-1.99769200	-1.97672600
H	1.64573900	-1.98265700	-2.19745200
H	2.67335000	-3.24702100	-0.04477100
H	0.61964800	-4.04238000	1.51848200
H	-1.68158500	-3.24870700	0.32784600
C	-0.91882200	-0.55382300	3.26139900
N	-0.38141800	-0.82989900	2.27443200
C	-1.59047800	-0.20808600	4.49050600
H	-2.50499300	0.35098800	4.26949100
H	-0.93584400	0.41017200	5.11202600
H	-1.85124900	-1.11581700	5.04294300
C	3.35712400	0.43239800	0.60664600
C	4.73575300	0.86541800	0.57327400
H	4.89114200	1.64657400	1.33268400
H	5.38871300	0.02642000	0.86572300
C	5.16450300	1.38176300	-0.80700900
H	5.00947500	0.59004400	-1.55443200
H	4.51522400	2.22008800	-1.09926000
C	6.61863300	1.82583400	-0.81453800
H	7.25503000	0.97953400	-0.51526600
H	6.76370000	2.60223900	-0.04838100

C	7.05282000	2.34879500	-2.17190400
H	6.44807900	3.21307700	-2.47552400
H	8.10189800	2.66463900	-2.16814800
H	6.94116100	1.58037400	-2.94781200
H	1.87227400	-0.87639000	1.39053500
C	0.80097900	1.08092300	0.04447200
H	1.25888700	1.44901800	-0.87589200
H	0.83664400	1.81435300	0.85599800
C	-0.47672600	0.42921400	-0.10245900
C	-1.71398300	0.82730500	-0.39213800
C	-2.88546100	-0.11623200	-0.39490200
H	-3.64058600	0.22189100	0.33431500
H	-2.54942300	-1.10295000	-0.04881300
C	-1.99258300	2.27150000	-0.74610900
H	-1.05351700	2.84420300	-0.73412400
H	-2.37209400	2.33239900	-1.78007700
C	-2.99945800	2.95052400	0.17952600
H	-2.67822800	2.82257300	1.22691300
H	-3.98010300	2.45598800	0.09949300
C	-3.16719900	4.43165100	-0.12528500
H	-2.19311500	4.93515400	-0.02809100
H	-3.46474600	4.55101300	-1.17834300
C	-4.18741200	5.10080900	0.77859100
H	-3.89902500	5.01388800	1.83447700
H	-4.29689500	6.16778900	0.55347100
H	-5.17661000	4.63704000	0.67017400
C	-3.56078300	-0.26338700	-1.75736300
H	-4.04924800	0.68205400	-2.04145500
H	-2.79504800	-0.44870400	-2.53001100

C	-4.58367800	-1.38882500	-1.78146500
H	-4.08129000	-2.33514900	-1.52122600
H	-5.33073400	-1.21787300	-0.99086200
C	-5.27518000	-1.52660300	-3.12592800
H	-6.00554900	-2.34360500	-3.12871300
H	-5.80920600	-0.60537800	-3.39280800
H	-4.55014000	-1.72713200	-3.92564600

D-TSby1

M06/BSI SCF energy in THF: -1086.086818 a.u.

M06/BSII SCF energy in THF: -1086.310779 a.u.

M06/BSII free energy in THF: -1085.818295 a.u.

C	2.07312300	0.20850100	-2.42925500
C	2.38229600	-1.16226500	-2.19210100
C	3.61113200	-1.21823400	-1.45821600
C	4.05279400	0.11514900	-1.24872100
C	3.11152300	1.00275800	-1.83287900
Ru	2.02004900	-0.15839000	-0.27363900
C	0.66855700	1.37706700	0.04666600
H	4.11534700	-2.11512900	-1.11915500
H	1.78984900	-2.01424200	-2.50796300
H	1.21029200	0.58666400	-2.96568900
H	3.16452500	2.08464100	-1.82587200
H	4.92724500	0.40564100	-0.67672200
C	0.41131800	-1.53431700	0.21268900
C	-0.75144900	-1.93915600	-0.31378800
C	3.11423100	0.81009600	2.61856500
N	2.70033900	0.43927500	1.60508900

C	3.63557100	1.27070600	3.88198800
H	2.88416900	1.87944200	4.39324100
H	4.53168400	1.87522500	3.71374200
H	3.89403200	0.41489600	4.51250900
C	1.59210600	-2.00505400	0.80565700
H	2.09837100	-2.86028500	0.35305900
H	1.77974200	-1.86733200	1.87009200
C	-1.22654000	-3.33494300	0.00471100
H	-0.50222200	-3.82029600	0.67317200
H	-1.20820600	-3.89961600	-0.94368200
C	-1.58630500	-1.08925400	-1.22803300
H	-0.93989900	-0.38162500	-1.76877200
H	-2.03794700	-1.74699400	-1.98705300
C	-2.62389700	-3.46172000	0.60457300
H	-3.37647100	-3.08761200	-0.10576600
H	-2.70256400	-2.82910900	1.50276400
C	-2.95787400	-4.90224500	0.96396300
H	-2.20744900	-5.28314800	1.67324100
H	-2.86925300	-5.52884600	0.06334600
C	-4.34849400	-5.04143700	1.55739400
H	-5.11489800	-4.68645000	0.85617300
H	-4.58438200	-6.08213000	1.80673500
H	-4.44604700	-4.44939600	2.47666000
C	-2.70120100	-0.29407500	-0.54364400
H	-3.40176700	-0.97333300	-0.03813400
H	-2.26722400	0.34237100	0.24451900
C	-3.46236300	0.56943100	-1.53832800
H	-3.76961400	-0.04994200	-2.39513100
H	-2.77874800	1.33190200	-1.94605400

C	-4.68177100	1.23127600	-0.92240300
H	-5.19039700	1.89298800	-1.63312900
H	-5.40969400	0.48072500	-0.58823800
H	-4.40765100	1.83252800	-0.04504300
C	0.05583000	2.42070400	0.22977100
C	-0.62323900	3.69054200	0.45346800
H	-0.38737400	4.05488500	1.46460600
H	-0.20940500	4.43866700	-0.23984600
C	-2.14170900	3.63567400	0.28148500
H	-2.38394100	3.28891000	-0.73525600
H	-2.56095900	2.88865200	0.97358200
C	-2.78257000	4.99155500	0.53381000
H	-2.34517900	5.72932400	-0.15584000
H	-2.51938600	5.33094000	1.54720200
C	-4.29188200	4.96625000	0.37269600
H	-4.75600300	4.26738800	1.08085300
H	-4.73533400	5.95317800	0.54705000
H	-4.57752600	4.65101400	-0.63945000
H	0.33460800	-0.00233300	0.20831800

D-TSby2

M06/BSI SCF energy in THF: -1086.067744 a.u.

M06/BSII SCF energy in THF: -1086.290977 a.u.

M06/BSII free energy in THF: -1085.797493 a.u.

C	-0.86664000	2.05232100	-1.70122100
C	-1.05205700	2.65940400	-0.42148100
C	-2.45179500	2.66821500	-0.13549800
C	-3.12148500	2.09286000	-1.27055900

C	-2.15801600	1.74151600	-2.23878400
Ru	-1.76012400	0.60243800	-0.28922800
C	-0.70873300	-1.33406800	-0.34324100
H	-2.92899400	3.07167000	0.74923700
H	-0.26293300	3.01542200	0.23383700
H	0.08240400	1.90034900	-2.20511400
H	-2.35516700	1.26922900	-3.19384600
H	-4.18972900	1.92262800	-1.34985500
C	-4.24800300	-1.11140000	0.86648100
N	-3.34365200	-0.51004000	0.46727400
C	-5.37996900	-1.85466300	1.36377200
H	-5.08511600	-2.88721400	1.57272300
H	-6.18085100	-1.85724200	0.61826400
H	-5.75074000	-1.39664900	2.28564100
C	-0.58562600	-2.57877800	-0.35974000
C	-0.13542200	-3.95096400	-0.27292300
H	-0.72155300	-4.48152800	0.49200600
H	-0.34432000	-4.47134100	-1.22208900
C	1.36553300	-4.04854300	0.03963500
H	1.92436400	-3.51761100	-0.74550000
H	1.57506900	-3.52275300	0.98382600
C	1.84108300	-5.48986600	0.13302300
H	1.60668700	-6.00981300	-0.80813700
H	1.27247700	-6.00985100	0.91849700
C	3.32902100	-5.58282300	0.42109200
H	3.58016200	-5.09376800	1.37112900
H	3.66665800	-6.62321800	0.48450300
H	3.91523400	-5.09145200	-0.36611900
H	-1.39245900	-0.86896600	-1.22854200

C	-0.88479900	0.26716800	1.65380700
H	-1.45506700	-0.47627400	2.21339000
H	-0.67194700	1.19073200	2.19397200
C	0.04717800	-0.16133000	0.66627200
C	1.35986800	0.09575800	0.43612100
C	2.22843500	0.47221700	1.60802500
H	3.25047100	0.09672800	1.44036700
H	1.85660200	-0.02788700	2.51438700
C	2.01311700	-0.11855800	-0.89188400
H	2.66691700	-1.00821600	-0.82243100
H	1.25476000	-0.35517900	-1.65132300
C	2.86069000	1.05624900	-1.38041100
H	3.68978400	1.23935600	-0.67980900
H	2.25308300	1.97702600	-1.37975900
C	3.42112600	0.82470000	-2.77545700
H	4.00333100	-0.10939200	-2.78197700
H	2.58812400	0.66660400	-3.47882200
C	4.28587000	1.97913600	-3.24975500
H	5.13974900	2.13418600	-2.57772500
H	4.68385600	1.80755700	-4.25620400
H	3.71488100	2.91671300	-3.27489300
C	2.29216500	1.97745400	1.88686200
H	2.68899200	2.50901500	1.00856500
H	1.27190600	2.36473400	2.04276900
C	3.14408200	2.30412500	3.10455000
H	2.73241400	1.78324300	3.98237800
H	4.15567300	1.89617700	2.95784200
C	3.22111300	3.79612100	3.37548900
H	2.22176900	4.22222100	3.53550000

H	3.82154500	4.02036800	4.26435000
H	3.67256900	4.32991200	2.52900700

C-IM1a

M06/BSI SCF energy in THF: -1086.122492 a.u.

M06/BSII SCF energy in THF: -1086.346576 a.u.

M06/BSII free energy in THF: -1085.856135 a.u.

C	1.06452400	0.68107400	-2.26584900
C	-0.11664400	-0.05705800	-2.51690100
C	0.25736500	-1.42522400	-2.74151200
C	1.66474000	-1.52336900	-2.63420900
C	2.17470200	-0.22143500	-2.30786200
Ru	0.85046500	-0.88008900	-0.67396700
C	0.69250300	0.19895500	1.27797500
H	-0.42743700	-2.24147700	-2.94222900
H	-1.12242000	0.34292600	-2.56299800
H	1.10768900	1.74151200	-2.03714700
H	3.21783800	0.03798300	-2.16694200
H	2.24894100	-2.43154700	-2.73002900
C	-1.21307600	-1.18004500	0.00968400
C	-2.27389400	-0.39724600	0.13650300
C	3.04565300	-2.71607300	0.85194000
N	2.23874400	-2.06967200	0.33264400
C	4.05064100	-3.52759700	1.49526900
H	3.76136700	-3.72521400	2.53166100
H	5.01208300	-3.00597800	1.48762500
H	4.15514100	-4.47983700	0.96688000
C	-0.55480500	-2.38290800	0.14538800

H	-0.66586600	-3.18232200	-0.58946900
H	-0.21732000	-2.70626200	1.13287100
C	-3.57000100	-1.00586500	0.62543700
H	-3.42959300	-2.08461600	0.78159400
H	-4.34007400	-0.90286500	-0.15693800
C	-2.26589500	1.07734400	-0.15247900
H	-2.45044400	1.63219900	0.78234600
H	-1.26375600	1.37794300	-0.48611200
C	-4.09077000	-0.37060100	1.91166800
H	-4.30989500	0.69571400	1.74441000
H	-3.30288200	-0.40310300	2.68256600
C	-5.34384800	-1.05870600	2.43276900
H	-5.12311600	-2.12012300	2.62252800
H	-6.11438700	-1.04542100	1.64670100
C	-5.88093400	-0.40714700	3.69441400
H	-6.13845100	0.64500700	3.51594200
H	-6.78151900	-0.91090600	4.06324500
H	-5.13436000	-0.42933100	4.49899200
C	-3.29886100	1.51421000	-1.18984200
H	-3.25952600	0.84243600	-2.06455100
H	-4.31541300	1.40988500	-0.77969600
C	-3.07633200	2.94897500	-1.64282900
H	-2.06966700	3.03058800	-2.08521500
H	-3.07119600	3.60860800	-0.76149400
C	-4.11947800	3.42185800	-2.63896500
H	-3.94053500	4.45459800	-2.95870600
H	-4.12278200	2.79259300	-3.53874100
H	-5.12757100	3.37837400	-2.20697100
C	1.68861700	0.69301100	0.72137600

C	2.92286900	1.45102600	0.49908400
H	3.74357300	0.74091600	0.30933400
H	2.83703400	2.06662500	-0.40823700
C	3.26200300	2.33573100	1.69900200
H	2.44267000	3.05015400	1.86650300
H	3.32062000	1.71356400	2.60433800
C	4.57026200	3.08593300	1.50247000
H	4.50799000	3.69129500	0.58548800
H	5.38035400	2.36025900	1.33389900
C	4.90708500	3.97208500	2.68824600
H	4.98962700	3.38313900	3.61096000
H	5.85694600	4.49926100	2.54576400
H	4.12896200	4.72872500	2.85152900
H	-0.05283400	0.01475900	2.03170400

A-TS1a

M06/BSI SCF energy in THF: -1086.088261 a.u.

M06/BSII SCF energy in THF: -1086.30942 a.u.

M06/BSII free energy in THF: -1085.812181 a.u.

C	3.24022800	0.62451500	-1.58865000
C	2.28133400	-0.08055800	-2.37050500
C	2.30051500	-1.44606200	-1.96259100
C	3.22986900	-1.58440000	-0.89243800
C	3.81283400	-0.28410900	-0.65998600
Ru	1.67507100	-0.22570900	-0.19447700
C	0.03778300	1.48909700	0.08760500
C	-1.05560000	2.43486000	-0.31191900
H	-2.02534900	2.17788600	0.13183400

H	-1.19726700	2.37461400	-1.40097600
H	1.67417000	-2.23330400	-2.36675400
H	1.65552700	0.34453700	-3.14701900
H	3.44178000	1.68825000	-1.64411600
H	4.56595100	-0.05250000	0.08421200
H	3.48647700	-2.49958300	-0.37116800
C	0.13445300	-1.50617000	0.01735800
C	-0.77229000	-0.37365300	-0.00792900
C	2.01993700	-0.73086300	2.96721100
N	1.87021300	-0.53740900	1.83680900
C	2.19575700	-0.97028100	4.37927200
H	2.57803800	-1.98227300	4.54190400
H	1.23441700	-0.86581400	4.89181000
H	2.90317000	-0.24773900	4.79674300
C	0.07611100	-2.82192300	0.17350600
H	-0.87019400	-3.35471300	0.27346200
H	0.98044000	-3.43024000	0.19036300
C	-1.52116900	-0.23173800	1.32682100
H	-1.94767000	0.77253900	1.43215800
H	-0.79656900	-0.35007300	2.14376100
C	-1.59715900	-0.25971900	-1.30200100
H	-2.45849300	0.39340000	-1.10978000
H	-0.99875100	0.23791400	-2.07684600
C	-2.65689400	-1.23279000	1.51610300
H	-2.28420200	-2.26508700	1.44462400
H	-3.40730000	-1.11077300	0.71997300
C	-3.33515400	-1.04207000	2.86672300
H	-3.70508700	-0.00837400	2.94378200
H	-2.58715700	-1.15736700	3.66610700

C	-4.47843500	-2.01758700	3.08242000
H	-4.12604200	-3.05634700	3.04024700
H	-4.96182600	-1.87165300	4.05506800
H	-5.24906300	-1.90200700	2.30916600
C	-2.10622700	-1.57345700	-1.88813300
H	-1.25724700	-2.19739000	-2.20483500
H	-2.64906300	-2.15782700	-1.13006200
C	-3.01991700	-1.32632800	-3.08036500
H	-2.48710100	-0.71456600	-3.82493300
H	-3.88448700	-0.72567400	-2.75906800
C	-3.49787300	-2.61747200	-3.72077400
H	-4.15381800	-2.42877200	-4.57795500
H	-2.65223600	-3.22007000	-4.07712400
H	-4.05828100	-3.23148300	-3.00387000
C	1.27670700	1.69571200	0.38351800
H	1.92015000	2.52905100	0.63792600
C	-0.70337100	3.87195800	0.05749400
H	0.23469300	4.16046000	-0.44049000
H	-0.51434800	3.94162100	1.13973400
C	-1.80730100	4.84284900	-0.33476200
H	-2.73864700	4.56114800	0.17904800
H	-2.01179400	4.74386300	-1.41170900
C	-1.45169000	6.28223800	-0.00742800
H	-0.54654300	6.59766200	-0.54232400
H	-2.25647200	6.97288200	-0.28275700
H	-1.25911500	6.40855700	1.06587600

A-TS1b

M06/BSI SCF energy in THF: -1086.101904 a.u.

M06/BSII SCF energy in THF: -1086.323535 a.u.

M06/BSII free energy in THF: -1085.826261 a.u.

C	1.80937300	-2.86606200	-0.17000700
C	0.44777000	-3.14280300	-0.47636600
C	-0.27916300	-3.16645800	0.74777800
C	0.61430000	-2.85516200	1.81225600
C	1.92166600	-2.66288900	1.23122200
Ru	0.55812200	-1.10672700	0.51207200
C	0.61549600	0.43624300	-1.20243600
H	-1.34458700	-3.34173600	0.84316500
H	0.03962100	-3.32154900	-1.46465200
H	2.60843100	-2.76981700	-0.89601200
H	2.82907300	-2.42025500	1.77352900
H	0.37115900	-2.80867400	2.86753400
C	-1.33796800	-0.44455600	0.84764100
C	-1.22315000	0.32905900	-0.35587800
C	1.32995000	1.08641400	2.73663800
N	1.04991600	0.32961900	1.90741400
C	1.66992900	2.03093400	3.77297200
H	2.74787400	2.21704400	3.77365500
H	1.37359100	1.63496400	4.74879800
H	1.14543100	2.97543300	3.59819300
C	-2.21134700	-0.66138100	1.82202700
H	-3.21056000	-0.22302000	1.80945200
H	-1.97290200	-1.30455700	2.66865300
C	-1.26923300	1.84173100	-0.12389900
H	-0.95156100	2.36334400	-1.03841400
H	-0.54533700	2.10433800	0.66014400

C	-1.97599400	-0.19705100	-1.58248100
H	-1.95839000	0.59051700	-2.35304100
H	-1.43377000	-1.05321400	-2.00544400
C	-2.64383100	2.37273000	0.27047500
H	-3.02420300	1.83627700	1.15204400
H	-3.36346400	2.19882600	-0.54345500
C	-2.58986900	3.86358400	0.57747600
H	-2.18716500	4.39997000	-0.29518000
H	-1.87492600	4.03641700	1.39656800
C	-3.94954200	4.42720700	0.94964100
H	-4.35752900	3.92462700	1.83626300
H	-3.89984200	5.49935700	1.17078500
H	-4.67206500	4.29136500	0.13460400
C	-3.41677100	-0.62649400	-1.32679600
H	-3.42766000	-1.49659000	-0.65263000
H	-3.97639900	0.16731500	-0.80932600
C	-4.13270100	-0.97942700	-2.62272300
H	-3.57054600	-1.76844700	-3.14572700
H	-4.12184500	-0.10575800	-3.29198300
C	-5.56322300	-1.43090600	-2.38874000
H	-6.07353700	-1.67263200	-3.32787000
H	-5.59924900	-2.32546800	-1.75346600
H	-6.14694000	-0.64830700	-1.88684900
C	1.75020000	0.06291700	-0.74085200
H	0.21664700	0.96107500	-2.06148600
C	3.21420600	0.21556200	-0.84631000
H	3.66096300	-0.73399000	-1.17881500
H	3.62079600	0.38924100	0.16368500
C	3.63097600	1.34571100	-1.78259300

H	3.19624800	1.17040000	-2.77887900
H	3.20116000	2.29262100	-1.42152100
C	5.14202900	1.47479400	-1.89433500
H	5.55934800	0.52684200	-2.26648700
H	5.56844300	1.62001600	-0.88988100
C	5.55929000	2.61718500	-2.80307600
H	5.18314100	3.57812700	-2.42855100
H	6.64907400	2.69693400	-2.88357200
H	5.16032000	2.48235200	-3.81671500

B-TS1a

M06/BSI SCF energy in THF: -1086.093066 a.u.

M06/BSII SCF energy in THF: -1086.315624 a.u.

M06/BSII free energy in THF: -1085.819465 a.u.

C	-1.28463200	-3.36899800	-0.36829400
C	-1.44891000	-2.62366900	-1.56790400
C	-2.50097000	-1.68380500	-1.35939300
C	-2.94434600	-1.80144000	-0.01858900
C	-2.18798400	-2.85730900	0.60573900
Ru	-0.82476900	-1.20932700	0.09149600
C	1.49782400	-1.06895200	0.42827500
H	-2.87099100	-0.97465900	-2.08958100
H	-0.89003100	-2.76237600	-2.48622500
H	-0.55459900	-4.15434700	-0.20852300
H	-2.30183900	-3.21096500	1.62386400
H	-3.72433600	-1.21453200	0.45383000
C	0.62488200	-0.26298100	-1.15117400
C	1.31067100	-0.65275800	-2.23039500

C	-1.31382900	0.31207700	2.89384000
N	-1.09390400	-0.17942900	1.86953500
C	-1.58839800	0.94761900	4.16009700
H	-1.29159700	0.29234700	4.98419100
H	-2.65771500	1.16406000	4.24339100
H	-1.02942600	1.88575100	4.23099600
C	-0.27491700	0.78563600	-0.81467700
C	0.86396200	-2.00876200	1.01700100
C	0.20054200	1.83904700	0.16804900
H	-0.65722700	2.34267200	0.63899000
H	0.76069600	1.37194700	0.98855300
C	-1.10878200	1.33085300	-1.96561400
H	-1.49273200	0.50268500	-2.57275200
H	-0.40504800	1.86898000	-2.62615700
C	-2.23005000	2.29905100	-1.59893100
H	-1.80300300	3.17864300	-1.09141300
H	-2.66197200	2.68614300	-2.53411300
C	-3.35255900	1.73469800	-0.74029200
H	-3.83879900	0.90042100	-1.26918700
H	-2.93905400	1.30688900	0.18638700
C	1.09721000	2.89216300	-0.48662800
H	1.87093200	2.39417900	-1.09650500
H	0.51371800	3.51174200	-1.18436400
C	1.76551300	3.78974100	0.54446800
H	0.99131900	4.25198900	1.17644600
H	2.37648600	3.16807800	1.21844000
C	2.62789600	4.86755600	-0.08793100
H	2.03121000	5.51989200	-0.73864600
H	3.10773700	5.50202900	0.66578800

H	3.42219500	4.42700800	-0.70469900
C	-4.38864900	2.79287900	-0.40015900
H	-4.83009600	3.22046900	-1.30994900
H	-5.20763000	2.38778500	0.20513200
H	-3.93693300	3.62095500	0.16209800
H	1.48119200	0.04851700	-3.04948200
H	1.76160800	-1.64048600	-2.31131800
H	0.93072500	-2.78618100	1.76447700
C	2.79796900	-0.33161600	0.51026600
H	2.76908400	0.59929200	-0.06797800
H	2.95885200	-0.05462400	1.56331200
C	3.95961100	-1.19733400	0.02801400
H	3.96530100	-2.14159900	0.59348100
H	3.80059200	-1.46539400	-1.02804500
C	5.29963800	-0.49388600	0.18326400
H	5.28597400	0.44470300	-0.39089600
H	5.43785900	-0.20499000	1.23602300
C	6.45964300	-1.36261500	-0.26927100
H	6.35996700	-1.63854500	-1.32694200
H	7.42145700	-0.85135600	-0.14988300
H	6.50527500	-2.29389900	0.31013600

B-TS1b

M06/BSI SCF energy in THF: -1086.099377 a.u.

M06/BSII SCF energy in THF: -1086.32205 a.u.

M06/BSII free energy in THF: -1085.826447 a.u.

C	1.28250600	-2.73323900	-0.98429100
C	-0.07647400	-2.74841000	-1.40893800

C	-0.90076200	-2.86331000	-0.25101100
C	-0.06158100	-2.86579500	0.88814700
C	1.30113900	-2.79439600	0.44526000
Ru	0.30707800	-0.92508300	-0.14901800
C	1.37434400	0.83541600	-1.15856400
H	-1.98387200	-2.89563000	-0.23681100
H	-0.42250700	-2.69169000	-2.43500700
H	2.14806700	-2.66070200	-1.63370700
H	2.18075300	-2.80706100	1.07846000
H	-0.39229800	-2.90090800	1.92053900
C	-0.45438200	0.35065100	-1.71341500
C	-0.47183900	0.32357200	-3.04557100
C	0.46892300	0.54324300	2.71872000
N	0.37852700	0.08759700	1.65867700
C	0.56473000	1.11474000	4.04047100
H	-0.38084600	0.97770300	4.57370800
H	0.78130300	2.18472600	3.96834000
H	1.36638900	0.62634400	4.60228500
C	-1.39607100	0.43054500	-0.65297700
C	2.12908500	0.05489600	-0.48643900
H	1.34545400	1.82078000	-1.59928400
C	3.46980300	-0.19501900	0.06812900
H	3.37731900	-0.28465200	1.16252100
H	3.83224100	-1.17577800	-0.27810200
C	4.47459100	0.89666700	-0.29003800
H	4.09580200	1.86674000	0.06693600
H	4.54879200	0.97836000	-1.38499200
C	5.84883900	0.62928800	0.30394200
H	5.75652900	0.52774900	1.39599500

H	6.21883900	-0.34087700	-0.06112600
C	6.84764100	1.72265000	-0.03105900
H	6.50738900	2.69723300	0.34235800
H	7.83129600	1.52487300	0.40937800
H	6.98339700	1.81655200	-1.11614700
C	-1.46416900	1.73971600	0.11241900
H	-1.85978500	1.57578000	1.12572300
H	-0.45344900	2.15182100	0.23958300
C	-2.73161900	-0.25182700	-0.87626600
H	-2.58691800	-1.16595700	-1.46780200
H	-3.34317900	0.41023500	-1.51640000
C	-3.52039600	-0.56189500	0.38863600
H	-2.87288800	-1.08483000	1.11481800
H	-3.82781800	0.37503000	0.87982800
C	-4.75354800	-1.40631000	0.10487900
H	-5.37154900	-0.89883100	-0.65155600
H	-4.44010800	-2.35806200	-0.35373100
C	-2.32154300	2.78909100	-0.59587000
H	-1.99799200	2.87823200	-1.64684300
H	-3.37459700	2.46951700	-0.62457000
C	-2.23445600	4.15023600	0.07894500
H	-2.53520500	4.05040800	1.13340600
H	-1.18432800	4.48032600	0.09311000
C	-3.09479800	5.19694100	-0.60633000
H	-4.15365900	4.90760300	-0.59816800
H	-3.01473200	6.17475800	-0.11789000
H	-2.79910900	5.32609200	-1.65567600
C	-5.57873200	-1.67710900	1.34982900
H	-5.93831900	-0.74106200	1.79644700

H	-6.45544400	-2.29772000	1.13336500
H	-4.98508100	-2.19717000	2.11342400
H	-1.37491000	0.61995000	-3.58162000
H	0.40653600	0.06851000	-3.63638800

C-TS1a

M06/BSI SCF energy in THF: -1086.106314 a.u.

M06/BSII SCF energy in THF: -1086.328449 a.u.

M06/BSII free energy in THF: -1085.834206 a.u.

C	-1.28591500	0.04870700	2.52857400
C	-0.00345500	-0.56612900	2.54574600
C	-0.19335500	-1.97769300	2.44171400
C	-1.58438100	-2.23514200	2.31609900
C	-2.26878300	-0.96661300	2.36475200
Ru	-0.98688900	-1.03485700	0.57533800
C	-0.48879100	0.52218200	-1.03812400
H	0.59791000	-2.71867000	2.41141000
H	0.94777600	-0.05456600	2.64459300
H	-1.47369400	1.11599600	2.57484800
H	-3.34144300	-0.81705900	2.31232500
H	-2.05169900	-3.20804700	2.21772800
C	0.89420500	-0.85503400	-0.50204700
C	2.06812900	-0.23544600	-0.31241600
C	-3.20107500	-2.25045000	-1.43576800
N	-2.39222000	-1.81495300	-0.73237200
C	-4.20481700	-2.80513800	-2.31149600
H	-3.88346800	-2.70718000	-3.35263600
H	-5.15289200	-2.27566200	-2.17902600

H	-4.35290900	-3.86493900	-2.08339800
C	0.38491200	-2.16416700	-0.64918000
H	0.87490000	-2.97290100	-0.10438400
H	-0.08820900	-2.46642100	-1.58498700
C	3.33337500	-0.92746500	-0.75020700
H	3.10890300	-1.96362800	-1.03811500
H	4.03130200	-0.98030100	0.10111700
C	2.19047400	1.16280800	0.21849900
H	2.45608700	1.84907800	-0.60466500
H	1.21472100	1.50102700	0.59200700
C	4.03267900	-0.22285100	-1.91207800
H	4.28410300	0.81165100	-1.63042400
H	3.33609100	-0.14689800	-2.76272300
C	5.30017500	-0.94189300	-2.35025300
H	5.05065700	-1.97432800	-2.63878100
H	5.98114600	-1.02477300	-1.48935700
C	6.00194700	-0.23778200	-3.49758800
H	6.28789500	0.78506500	-3.21969700
H	6.91306700	-0.76444000	-3.80319800
H	5.34999100	-0.16716700	-4.37795600
C	3.22649200	1.31212000	1.33206200
H	3.08427100	0.51522500	2.08211400
H	4.24218600	1.17014500	0.93228900
C	3.14033100	2.66714900	2.01784200
H	2.13480200	2.78640700	2.45269800
H	3.23947000	3.46213800	1.26297700
C	4.19356000	2.84121000	3.09713100
H	4.11213000	3.81452600	3.59396700
H	4.10093900	2.06641600	3.86965000

H	5.20539500	2.76539300	2.67847000
C	-1.54469700	0.76370200	-0.36729800
C	-2.75024400	1.59545100	-0.20896600
H	-3.63563500	0.94014200	-0.23871400
H	-2.75331300	2.04724500	0.79549700
C	-2.85629500	2.68341800	-1.27414000
H	-1.94119400	3.29515700	-1.25791100
H	-2.89433000	2.21327000	-2.26879100
C	-4.07355100	3.57320300	-1.07368300
H	-4.02171700	4.03494900	-0.07602300
H	-4.98210100	2.95164700	-1.07411700
C	-4.18388700	4.65170000	-2.13631400
H	-4.27869800	4.21364600	-3.13831400
H	-5.05569500	5.29476900	-1.97252000
H	-3.29390700	5.29429900	-2.14221500
H	0.05365200	0.80208100	-1.92914600

C-TS1b

M06/BSI SCF energy in THF: -1086.10371 a.u.

M06/BSII SCF energy in THF: -1086.325649 a.u.

M06/BSII free energy in THF: -1085.829577 a.u.

C	-2.38572500	-1.88856400	1.96588100
C	-1.49548900	-2.67579500	1.18152700
C	-2.14015100	-2.94716400	-0.06598200
C	-3.40105700	-2.29149100	-0.07198300
C	-3.54910200	-1.62451900	1.19836000
Ru	-1.84586800	-0.76067800	0.07073700
C	-0.29024000	0.91677000	0.38790600

H	-1.71383100	-3.52004100	-0.88202800
H	-0.52438300	-3.04203200	1.49210100
H	-2.17923100	-1.50256000	2.95784500
H	-4.40377600	-1.03689200	1.51285500
H	-4.13036300	-2.30326000	-0.87340900
C	0.16403100	-0.69457300	-0.67400100
C	1.35851900	-1.18106300	-0.28735500
C	-3.53145400	1.63798300	-1.32050200
N	-2.92015500	0.77919000	-0.84329700
C	-4.29793600	2.70504100	-1.91714600
H	-3.63109600	3.52003800	-2.21422800
H	-5.02833400	3.08850900	-1.19859900
H	-4.82519100	2.33622000	-2.80195500
C	-0.72478800	-0.79786400	-1.77140000
H	-0.79616100	-1.75227500	-2.29516900
H	-0.94106100	0.07708800	-2.38779500
C	2.27491500	-1.74850400	-1.34196400
H	1.80252100	-1.66383200	-2.33016000
H	2.38456600	-2.82804900	-1.13630600
C	1.85697200	-1.21787500	1.13352800
H	1.02308800	-1.39367800	1.82642800
H	2.52153400	-2.09214900	1.22390600
C	3.67362100	-1.13842200	-1.40161500
H	4.18244600	-1.26395300	-0.43279800
H	3.59593700	-0.05247700	-1.57307300
C	4.52489600	-1.76790400	-2.49436900
H	4.01637500	-1.65125000	-3.46352300
H	4.59392900	-2.85213600	-2.31713900
C	5.91695600	-1.16585500	-2.56505700

H	6.45095300	-1.29154500	-1.61406600
H	6.52371100	-1.63198100	-3.34951600
H	5.87291900	-0.08938000	-2.77575000
C	2.62503300	0.01707100	1.62228200
H	3.34924400	0.35357200	0.86629800
H	1.92213600	0.85145000	1.76777300
C	3.35486200	-0.25278400	2.92963800
H	4.09554800	-1.05198600	2.77048800
H	2.64008900	-0.64538800	3.66970400
C	4.03871700	0.98749000	3.47650500
H	4.57224600	0.78329900	4.41166700
H	4.76839200	1.38579500	2.75927600
H	3.31064600	1.78440900	3.67758700
C	-1.13573500	0.76957100	1.33385600
H	-1.57394800	1.20367600	2.22013400
C	0.46655500	1.95362100	-0.35909000
H	0.12259000	1.94693600	-1.40664000
H	1.53331500	1.68833800	-0.39509000
C	0.27463100	3.33814600	0.24662900
H	-0.79738400	3.59150900	0.24857500
H	0.58793000	3.31987800	1.30268700
C	1.05596000	4.40592000	-0.50362500
H	2.12334700	4.13905700	-0.50664300
H	0.74168600	4.41022100	-1.55860400
C	0.86904200	5.78675000	0.09959700
H	1.43182400	6.55115000	-0.44767900
H	-0.18795500	6.08315700	0.09015600
H	1.20680200	5.81014900	1.14364300

D-TS1a

M06/BSI SCF energy in THF: -1086.106718 a.u.

M06/BSII SCF energy in THF: -1086.328975 a.u.

M06/BSII free energy in THF: -1085.83055 a.u.

C	1.96185000	-1.52153500	2.23504700
C	0.67778800	-2.13187500	2.14805700
C	-0.29690200	-1.14190100	2.47376700
C	0.37382500	0.08485900	2.71181100
C	1.78456200	-0.14307200	2.56743200
Ru	0.83270200	-0.50644400	0.62559600
C	1.96105800	-1.31600700	-1.24535700
H	-1.36961100	-1.29299600	2.48598800
H	0.47654900	-3.16759400	1.89938500
H	2.90677200	-2.01605400	2.03793200
H	2.56607600	0.59288200	2.71628700
H	-0.09462900	1.03056400	2.96083900
C	-0.93908300	-0.81829800	-0.39275100
C	-0.06650800	-1.56399200	-1.22521100
C	0.49902600	2.43676100	-0.65400900
N	0.66478600	1.37646900	-0.22153800
C	0.25917100	3.75810000	-1.18286300
H	1.19537400	4.32107600	-1.23858300
H	-0.44001800	4.29266400	-0.53171400
H	-0.17222900	3.68289900	-2.18569000
C	-2.19100800	-0.36705400	-0.37231700
C	2.62721300	-0.56212000	-0.46422500
C	-3.18401900	-0.87545900	-1.39374400
H	-3.48192900	-0.04488700	-2.05577000

H	-2.69885100	-1.62295300	-2.03726500
C	-2.67443100	0.67125000	0.60231300
H	-1.85126200	0.94544100	1.27526600
H	-3.47525100	0.26190500	1.24006600
C	-3.19398300	1.93479800	-0.08316900
H	-2.44686300	2.28763400	-0.81580400
H	-4.10094300	1.70629400	-0.66463900
C	-3.49614500	3.05040900	0.90557200
H	-4.20897500	2.68181300	1.65906300
H	-2.57591100	3.29996700	1.45883900
C	-4.44538200	-1.48047200	-0.78357400
H	-4.16617400	-2.28144200	-0.07961700
H	-4.97582300	-0.72153500	-0.18699500
C	-5.39329900	-2.03400100	-1.83726600
H	-5.64094500	-1.23512100	-2.55315000
H	-4.87557100	-2.81169700	-2.41922900
C	-6.66663700	-2.59973500	-1.23429600
H	-7.21718100	-1.82901000	-0.67925000
H	-7.34084900	-2.99958800	-2.00026400
H	-6.44461200	-3.41376100	-0.53201900
C	-4.05069700	4.29448100	0.23454100
H	-4.99133800	4.07665600	-0.28755500
H	-4.25095400	5.09425700	0.95636600
H	-3.34900000	4.69090600	-0.51197400
H	-0.04060500	-2.65229500	-1.14071600
H	0.00246100	-1.20014100	-2.25249400
C	3.93268000	0.06938600	-0.19578200
H	3.77778100	1.15318500	-0.06859100
H	4.31188700	-0.28809600	0.77425300

C	4.95964800	-0.19587500	-1.29223900
H	5.09062100	-1.28270300	-1.40934200
H	4.56746700	0.17214900	-2.25273900
C	6.30121400	0.45712500	-0.99847700
H	6.15653200	1.54114000	-0.87326600
H	6.68026400	0.08765600	-0.03335100
C	7.32336500	0.19454100	-2.08979100
H	8.28475500	0.67319700	-1.87219300
H	7.50829700	-0.88087500	-2.20915400
H	6.97505000	0.57640500	-3.05826500
H	2.03742000	-1.98159600	-2.09090300

D-TS1b

M06/BSI SCF energy in THF: -1086.104083 a.u.

M06/BSII SCF energy in THF: -1086.325782 a.u.

M06/BSII free energy in THF: -1085.826539 a.u.

C	2.12879100	-1.88865800	-1.98929100
C	1.59542200	-0.63514800	-2.40719600
C	0.18221500	-0.78349300	-2.52863100
C	-0.16438600	-2.10464100	-2.14009700
C	1.04753700	-2.80145000	-1.80410800
Ru	0.78740800	-1.15384800	-0.38570000
C	2.43502100	-0.46854500	1.22158300
H	-0.51161700	-0.00323200	-2.81827800
H	2.16302200	0.26453300	-2.61710800
H	3.17596000	-2.09299600	-1.79302300
H	1.12285300	-3.83812900	-1.49852300
H	-1.16569900	-2.52052900	-2.10904100

C	-0.28929600	0.53199200	0.09422100
C	0.92107500	0.82526100	0.78641200
C	-1.18246700	-2.44360300	1.81790300
N	-0.44255800	-1.97739700	1.06071300
C	-2.12213500	-3.01796100	2.75096300
H	-1.61917500	-3.75017000	3.38966800
H	-2.92856200	-3.51871700	2.20577100
H	-2.54970500	-2.23112500	3.38015200
C	-1.53568600	0.98799400	-0.00396500
C	2.31027500	-1.70254700	0.91692900
C	-1.89700200	2.32378700	0.60420100
H	-2.65009800	2.17200400	1.39590700
H	-1.01342900	2.75440900	1.09683800
C	-2.64014500	0.20148100	-0.65264500
H	-2.22135000	-0.71996600	-1.07610000
H	-3.08171200	0.76054600	-1.49352400
C	-3.75179100	-0.17759200	0.32814400
H	-3.30162400	-0.55198700	1.26422600
H	-4.33557600	0.71242100	0.60998600
C	-4.68258700	-1.23927900	-0.23568300
H	-5.12207100	-0.87639400	-1.17722100
H	-4.08610800	-2.12749700	-0.50265600
C	-2.44712000	3.33196600	-0.40165300
H	-1.73174100	3.44582700	-1.23232000
H	-3.37779600	2.95007700	-0.85010500
C	-2.71870300	4.69122700	0.22574600
H	-3.42071700	4.56792500	1.06450300
H	-1.78705500	5.07968300	0.66488900
C	-3.27637600	5.69154700	-0.77115800

H	-4.22556000	5.34008300	-1.19627900
H	-3.46177600	6.66858800	-0.31055700
H	-2.58108000	5.84683700	-1.60648800
C	-5.78153800	-1.62735200	0.73724800
H	-6.40466600	-0.76172000	0.99647700
H	-6.44244600	-2.39829200	0.32538800
H	-5.36100400	-2.01655000	1.67490300
H	1.57516800	1.59180000	0.36611100
H	0.80587300	0.87671100	1.87279300
H	2.78920100	-2.66023000	1.07094300
C	3.42669500	0.50627800	1.73775400
H	2.94942100	1.31702800	2.30475800
H	4.05849800	-0.04820700	2.44663600
C	4.30450400	1.08389800	0.62680800
H	4.79890500	0.25508800	0.09546900
H	3.67545800	1.59559600	-0.11969700
C	5.34631800	2.05403800	1.16212700
H	4.83829300	2.86834200	1.70050300
H	5.97029300	1.53900100	1.90758600
C	6.21951900	2.62665400	0.05995300
H	5.61677400	3.16466800	-0.68324700
H	6.96333100	3.32871400	0.45270900
H	6.76065300	1.83182300	-0.46961000

B-TS2b′

M06/BSI SCF energy in THF: -1086.116363 a.u.

M06/BSII SCF energy in THF: -1086.336906 a.u.

M06/BSII free energy in THF: -1085.83995 a.u.

C	-3.99945800	-0.12182500	-0.72024300
C	-3.84701000	-1.53439700	-0.89777000
C	-3.83157800	-2.12781400	0.40017100
C	-3.93729600	-1.11328700	1.38010600
C	-4.03720200	0.13796400	0.68472900
Ru	-2.09649000	-0.68749900	0.14537600
C	-0.14731400	0.61324000	0.75248800
H	-3.67047200	-3.18120600	0.60384900
H	-3.78883600	-2.06039000	-1.84276500
H	-4.07409000	0.61507000	-1.51239800
H	-4.15562400	1.10933500	1.15151300
H	-3.94343000	-1.25489100	2.45348300
C	0.59786800	-0.06205100	-0.29667500
C	-0.46877800	-0.08949600	-1.35654100
C	-0.10666900	-3.25642600	0.46456100
N	-0.82363900	-2.35345800	0.36485800
C	0.79635600	-4.37376400	0.59582000
H	0.31022300	-5.18402400	1.14740900
H	1.08823800	-4.74052900	-0.39283500
H	1.69254700	-4.06143000	1.14213100
C	1.84225400	-0.57950800	-0.27034000
C	-1.31277200	1.08686300	0.14233100
C	-1.83875400	2.45912600	0.01158900
H	-2.08613600	2.76485900	1.04499300
H	-2.77980700	2.48307300	-0.55334100
C	-0.82798800	3.44963800	-0.56760000
H	-0.55845600	3.14383700	-1.59029400
H	0.10035600	3.40903500	0.02348600
C	-1.36877400	4.87064300	-0.58628900

H	-2.30487800	4.89405600	-1.16429400
H	-1.63670700	5.16711900	0.43916100
C	-0.37381800	5.85711100	-1.17051600
H	0.55937600	5.86687900	-0.59270700
H	-0.76963600	6.87863100	-1.17742400
H	-0.11673800	5.59430400	-2.20479900
H	0.22503800	0.99068100	1.70677400
H	-0.56487700	-0.96617100	-2.00600900
H	-0.51043100	0.81047100	-1.98338300
C	2.67725900	-0.46574200	0.97922500
H	3.50295400	-1.19269700	0.93514600
H	2.07560000	-0.74728300	1.86061400
C	3.25649700	0.93092700	1.21789000
H	2.43368400	1.64052000	1.40253200
H	3.75157700	1.28870700	0.30148000
C	4.24053600	0.97328300	2.37573200
H	3.75038300	0.59191500	3.28498500
H	5.06935100	0.27836000	2.16693800
C	4.78719600	2.36777100	2.62526100
H	5.50638700	2.38440200	3.45189200
H	3.98158300	3.07141100	2.87222400
H	5.29832400	2.75559900	1.73441300
C	2.40834800	-1.28211900	-1.47143400
H	1.60245800	-1.44306900	-2.20058100
H	2.74849900	-2.28739900	-1.16892300
C	3.56193400	-0.56574900	-2.19326700
H	3.62929600	-0.98089400	-3.21055900
H	3.29345200	0.49418500	-2.31558000
C	4.94595700	-0.67939900	-1.55717300

H	5.64100200	-0.07388300	-2.15561100
H	4.95427600	-0.23011300	-0.55385500
C	5.47028900	-2.10456300	-1.48341300
H	6.50479900	-2.13050700	-1.12252500
H	5.45166700	-2.58356000	-2.47170900
H	4.87755300	-2.73281700	-0.80583700

C-IM2a

M06/BSI SCF energy in THF: -1086.155568 a.u.

M06/BSII SCF energy in THF: -1086.375333 a.u.

M06/BSII free energy in THF: -1085.877392 a.u.

C	-0.26835400	-0.18166900	2.48517600
C	0.92797100	-0.91474800	2.18864800
C	0.61653600	-2.30148100	2.08716800
C	-0.77717300	-2.42218800	2.23332600
C	-1.33845400	-1.11050800	2.49213400
Ru	-0.53384900	-1.04585000	0.45580000
C	-0.70630100	0.68241200	-1.59393500
H	1.31731200	-3.10083800	1.87859900
H	1.92498400	-0.49390400	2.14370400
H	-0.33221600	0.88578400	2.66650000
H	-2.38174600	-0.89303300	2.69215200
H	-1.34750300	-3.34177600	2.16141700
C	0.42701600	-0.27400300	-1.36905700
C	1.51305000	0.16180800	-0.56230900
C	-3.19571700	-2.49871700	-0.61977000
N	-2.23321200	-1.97157300	-0.25521600
C	-4.39249300	-3.16109100	-1.07792200

H	-4.53878400	-2.96250200	-2.14390500
H	-5.26077400	-2.79090600	-0.52486600
H	-4.30349500	-4.24066500	-0.92458300
C	0.20365400	-1.66953800	-1.53727700
H	1.01785900	-2.38706400	-1.46650600
H	-0.61128300	-1.96767600	-2.19328700
C	2.75536900	-0.66777700	-0.42999500
H	2.53196200	-1.73859600	-0.37579300
H	3.28781200	-0.41310800	0.49474600
C	1.64566600	1.62714000	-0.24764400
H	1.95155100	2.10968800	-1.19469300
H	0.65346900	2.04850900	-0.04001200
C	3.69237900	-0.40946000	-1.61825600
H	3.85319000	0.67244000	-1.74285000
H	3.20659200	-0.75815500	-2.54294000
C	5.03584800	-1.10016100	-1.44067600
H	4.87357200	-2.17694900	-1.28107900
H	5.51132700	-0.72334400	-0.52219900
C	5.95653600	-0.88378200	-2.62819100
H	6.14941100	0.18452100	-2.79089100
H	6.92497900	-1.37644800	-2.48667800
H	5.51393400	-1.28085200	-3.55079000
C	2.62257900	2.04355000	0.84286200
H	2.37616700	1.53808800	1.79002600
H	3.65016800	1.74006000	0.58909100
C	2.59262700	3.54866900	1.06980200
H	1.56804600	3.85233200	1.33438300
H	2.82632700	4.06270600	0.12508100
C	3.55883400	3.99122500	2.15360900

H	3.52266800	5.07435200	2.31470200
H	3.32880300	3.50676400	3.11172900
H	4.59321100	3.73014800	1.89486200
C	-1.46936100	0.53998700	-0.50878400
C	-2.69238900	1.25724400	-0.06229100
H	-3.49009000	0.52253600	0.13896300
H	-2.48955600	1.72729600	0.91587700
C	-3.19459100	2.31071200	-1.04213300
H	-2.40185700	3.05588700	-1.21524400
H	-3.38699800	1.83882900	-2.01882000
C	-4.45542100	3.01155500	-0.55830800
H	-4.25754700	3.48304700	0.41656700
H	-5.23925000	2.25997100	-0.37702900
C	-4.95851800	4.05224300	-1.54264300
H	-5.18683100	3.59872200	-2.51595100
H	-5.86963600	4.54554600	-1.18571100
H	-4.20523900	4.83211000	-1.71427700
H	-0.75445100	1.40633700	-2.41018100

C-TS2a

M06/BSI SCF energy in THF: -1086.137242 a.u.

M06/BSII SCF energy in THF: -1086.356508 a.u.

M06/BSII free energy in THF: -1085.85689 a.u.

C	-0.15239200	-0.21933100	2.67603600
C	1.15484300	-0.72441700	2.37548500
C	1.05788100	-2.13503800	2.16718400
C	-0.30471800	-2.48358600	2.22547300
C	-1.06031300	-1.29023200	2.54031900

Ru	-0.09099100	-0.94265300	0.56199600
C	-0.73028500	0.09523500	-1.61515600
H	1.88158200	-2.79686100	1.92579500
H	2.07319100	-0.14852600	2.38617700
H	-0.40066400	0.80850700	2.91479500
H	-2.13677000	-1.23759800	2.65630800
H	-0.72511900	-3.46773900	2.05538900
C	0.62562800	-0.50332200	-1.36021200
C	1.53761000	0.29377500	-0.59274500
C	-3.24341000	-2.71248100	-0.64220500
N	-2.26706800	-2.34319800	-0.13472400
C	-4.45771700	-3.16539600	-1.28170600
H	-4.92966400	-2.33520800	-1.81581400
H	-5.15631900	-3.54890300	-0.53246400
H	-4.22908400	-3.96330400	-1.99401100
C	0.74937000	-1.91577700	-1.22123400
H	1.71127800	-2.38534500	-1.03124500
H	0.00631100	-2.55605200	-1.69382300
C	2.93921400	-0.21531900	-0.33214000
H	2.94904500	-1.23156700	0.07884700
H	3.41280600	0.42459900	0.42584500
C	1.45409300	1.80184100	-0.57364600
H	2.32716300	2.16112700	-1.14468200
H	0.56744900	2.15352100	-1.11344900
C	3.79540400	-0.18989100	-1.60245900
H	3.75449700	0.80851100	-2.06497400
H	3.37221100	-0.88676800	-2.34278600
C	5.24665200	-0.55714500	-1.33076300
H	5.28824800	-1.54878200	-0.85443200

H	5.66832200	0.14948000	-0.60005300
C	6.08651600	-0.55561000	-2.59578000
H	6.07276600	0.43021700	-3.07814600
H	7.13259100	-0.80897100	-2.39039200
H	5.70576400	-1.28225400	-3.32516400
C	1.48679600	2.46895300	0.79618900
H	0.61687700	2.13523600	1.38147700
H	2.37975700	2.15658100	1.36067500
C	1.46776500	3.98660100	0.68827900
H	0.57371900	4.29388200	0.12312900
H	2.33197600	4.32004000	0.09404000
C	1.47929800	4.66662500	2.04539100
H	1.46845900	5.75850700	1.95468300
H	0.60406100	4.37689800	2.64216600
H	2.37393700	4.39196500	2.61962900
C	-1.22491500	0.43496500	-0.40994300
C	-2.41667900	1.21031900	0.00584400
H	-3.00538000	0.62227200	0.72823500
H	-2.07901600	2.10141600	0.56370000
C	-3.29780700	1.64605500	-1.16063300
H	-2.69900500	2.25365000	-1.85761700
H	-3.61908400	0.75705200	-1.72809200
C	-4.51984400	2.43343600	-0.71348900
H	-4.19491300	3.29940100	-0.11704100
H	-5.12545100	1.80978900	-0.03831300
C	-5.36499300	2.90138800	-1.88483000
H	-5.71296400	2.05132700	-2.48627000
H	-6.24941600	3.45772500	-1.55481600
H	-4.78841900	3.55797300	-2.54912300

H	-1.19563500	0.16740600	-2.60251700

C-TS2a′

M06/BSI SCF energy in THF: -1086.097155 a.u.

M06/BSII SCF energy in THF: -1086.316015 a.u.

M06/BSII free energy in THF: -1085.815864 a.u.

C	1.28158200	-0.05870900	2.44490200
C	0.67340700	1.20721500	2.22109100
C	1.70223300	2.12145500	1.83139700
C	2.92839600	1.40922200	1.77850600
C	2.66424500	0.04020400	2.15235100
Ru	1.60170500	0.53855600	0.27464400
C	0.47854000	-0.58935000	-1.33375100
H	1.56649800	3.17028500	1.59166800
H	-0.37263400	1.43703200	2.37736600
H	0.75646600	-0.96130200	2.73576600
H	3.39626100	-0.75628100	2.21903500
H	3.89506200	1.81341200	1.49988200
C	0.02430100	0.78976100	-1.20272300
C	-1.26883300	0.82541400	-0.54849700
C	4.11747100	-0.34040500	-1.55198000
N	3.16372600	-0.04646700	-0.96383700
C	5.30323500	-0.70959300	-2.28807800
H	5.29454800	-1.78244100	-2.50264200
H	6.19563700	-0.47049700	-1.70176100
H	5.34229500	-0.15795100	-3.23220900
C	0.96077500	1.85424100	-1.37839500
H	0.67255700	2.87464600	-1.12625600

H	1.67771200	1.76471200	-2.19401400
C	-1.69636000	2.06220800	0.16955300
H	-0.82661600	2.65032800	0.48407200
H	-2.24839400	1.78936600	1.08144500
C	-2.37734700	-0.08006400	-0.98438500
H	-3.04959000	0.52183000	-1.62415700
H	-1.99075600	-0.88826400	-1.61336600
C	-2.60649400	2.95380300	-0.69389000
H	-3.57724100	2.46095300	-0.84834400
H	-2.15983900	3.08265700	-1.69284900
C	-2.82660800	4.31597100	-0.05444200
H	-1.85436000	4.81418100	0.07918900
H	-3.24120900	4.17869000	0.95563100
C	-3.74878200	5.19328500	-0.88236600
H	-4.73675900	4.73014700	-1.00072600
H	-3.89651700	6.17619300	-0.42124600
H	-3.34019400	5.35670800	-1.88799000
C	-3.21316900	-0.67078400	0.15107700
H	-2.52330300	-1.10168700	0.89599100
H	-3.77684500	0.12033700	0.66972300
C	-4.17309700	-1.74424700	-0.33704500
H	-3.60128300	-2.51967000	-0.87193300
H	-4.86615200	-1.31312000	-1.07509200
C	-4.94916800	-2.38015200	0.80261500
H	-5.64295700	-3.14917800	0.44494500
H	-4.26932900	-2.85553400	1.52303100
H	-5.53660500	-1.63252100	1.35126600
C	0.21343600	-1.18854200	-0.13366500
C	0.46270900	-2.61405500	0.21843000

H	1.37522900	-2.96421800	-0.30519900
H	0.65730200	-2.73717400	1.29325900
C	-0.72412700	-3.49257900	-0.17475000
H	-1.62617400	-3.12770800	0.34252500
H	-0.92248900	-3.37571700	-1.25311200
C	-0.52562000	-4.96414500	0.14940600
H	-0.31286200	-5.07332900	1.22379300
H	0.36414500	-5.34070700	-0.37754400
C	-1.74386400	-5.79037700	-0.22405200
H	-1.95256700	-5.72289000	-1.29986600
H	-1.61384200	-6.85024300	0.02186900
H	-2.63858900	-5.43344100	0.30486500
H	1.02109300	-0.99748400	-2.19233500

C-IM3a

M06/BSI SCF energy in THF: -953.4809788 a.u.

M06/BSII SCF energy in THF: -953.6657288 a.u.

M06/BSII free energy in THF: -953.2066338 a.u.

C	0.56165800	-1.93774400	-2.04444500
C	-0.86487100	-1.92881200	-2.00358700
C	-1.28463800	-2.92882000	-1.08894000
C	-0.12308400	-3.54653000	-0.53287200
C	1.01990800	-2.93625600	-1.13936600
Ru	-0.05507000	-1.38701200	0.03616500
C	0.68892100	-0.26233300	1.76975500
H	-2.31221300	-3.15464400	-0.82696500
H	-1.51280800	-1.26292300	-2.56313300
H	1.18475900	-1.28704600	-2.64801200

H	2.05536800	-3.18368200	-0.93426500
H	-0.10941500	-4.35337000	0.18968900
C	-0.76909800	-0.13197400	1.51503800
C	-1.16634900	0.66422000	0.39732600
C	-1.45609300	-1.36325700	1.75338000
H	-2.49823000	-1.48404000	1.46634700
H	-1.11551900	-2.00048800	2.56689000
C	-2.61170100	0.65455400	-0.04126000
H	-2.99648100	-0.36346300	-0.18107200
H	-2.68303600	1.14001400	-1.02515700
C	-0.41578800	1.90783200	-0.00896300
H	-1.07275400	2.76593100	0.21234100
H	0.47974200	2.04143000	0.61491400
C	-3.51849400	1.39764000	0.94506100
H	-3.12433100	2.40894300	1.13059200
H	-3.50139800	0.88011700	1.91695600
C	-4.95264900	1.49711600	0.44808000
H	-5.33265200	0.48639900	0.23356100
H	-4.96710800	2.03769800	-0.51019700
C	-5.86266900	2.18830100	1.44795100
H	-5.51171800	3.20523500	1.66597600
H	-6.89078000	2.26545200	1.07696900
H	-5.89330800	1.64065500	2.39887900
C	-0.01051500	1.98806600	-1.47758600
H	0.62192900	1.12042600	-1.72425700
H	-0.89781900	1.91375000	-2.12580100
C	0.74445500	3.27019900	-1.79558600
H	1.60889700	3.35446400	-1.11741900
H	0.10394000	4.13733300	-1.57613700

C	1.21562300	3.32044300	-3.23751900
H	1.75800000	4.24634600	-3.45878200
H	1.88912000	2.48205000	-3.46193400
H	0.37017200	3.25801700	-3.93481700
C	1.42095200	-0.25628600	0.59488100
C	2.78843600	0.09286700	0.19144700
H	3.25599500	-0.76433800	-0.31684300
H	2.68048500	0.85785800	-0.60112100
C	3.66823700	0.63600700	1.31229400
H	3.14712400	1.47437700	1.80162900
H	3.80378700	-0.13773800	2.08336100
C	5.02406300	1.09956600	0.80321400
H	4.87323100	1.87305200	0.03453400
H	5.52818800	0.26233900	0.29718900
C	5.90498800	1.63927500	1.91534900
H	6.09856600	0.87161900	2.67562200
H	6.87418800	1.98167100	1.53619200
H	5.42776100	2.48850600	2.42137000
H	1.07363000	-0.55786500	2.74975000

C-TS3a

M06/BSI SCF energy in THF: -953.4529109 a.u.

M06/BSII SCF energy in THF: -953.6378502 a.u.

M06/BSII free energy in THF: -953.17822129 a.u.

C	1.25049900	-1.93028800	-1.89101200
C	0.03652500	-2.68634500	-1.74861100
C	0.19452800	-3.57085400	-0.65906600
C	1.48045800	-3.34486800	-0.07663200

C	2.15007400	-2.35084400	-0.87322800
Ru	0.42665400	-1.44302500	0.08311700
C	0.81669100	-0.07138800	1.73907900
H	-0.56238900	-4.24514200	-0.27449900
H	-0.85433700	-2.57796100	-2.35670100
H	1.43937500	-1.17393100	-2.64380300
H	3.15449500	-1.97805100	-0.70771200
H	1.90014300	-3.86846000	0.77374900
C	-0.58762300	-0.46909000	1.65350300
C	-1.08546400	0.43870800	0.59511300
C	-0.97321000	-1.81308100	1.79774100
H	-1.97069300	-2.13417700	1.49961900
H	-0.47294500	-2.42544100	2.54565500
C	-2.22007100	-0.01511700	-0.31288400
H	-2.17193300	-1.09786200	-0.48075500
H	-2.09818900	0.44120400	-1.30529800
C	-1.15498500	1.93580600	0.89224300
H	-2.07563400	2.08865900	1.47848400
H	-0.33449700	2.25386300	1.54705900
C	-3.60744200	0.32549500	0.24429900
H	-3.79490300	1.40764400	0.19304200
H	-3.65540500	0.05443000	1.31196800
C	-4.70548100	-0.41026100	-0.50985500
H	-4.54155600	-1.49508900	-0.41596700
H	-4.62383900	-0.18140200	-1.58341400
C	-6.09202100	-0.05072900	-0.00713100
H	-6.29310800	1.02092600	-0.13320600
H	-6.87529200	-0.59894900	-0.54243100
H	-6.19772500	-0.28028300	1.06113000

C	-1.20679600	2.82086300	-0.34835800
H	-0.41274800	2.52828400	-1.05450700
H	-2.15544200	2.66706400	-0.88381600
C	-1.06718100	4.29892900	-0.01524700
H	-0.10529400	4.46963500	0.49310100
H	-1.84574600	4.58269800	0.70890800
C	-1.16207500	5.17504800	-1.25145800
H	-1.05523900	6.23815300	-1.00914700
H	-0.37792100	4.92024600	-1.97644500
H	-2.12844600	5.04549600	-1.75577300
C	0.97138900	0.45508900	0.44660600
C	1.92776900	1.40333500	-0.15974400
H	1.91704300	1.30974600	-1.25441200
H	1.62068500	2.43484000	0.07672800
C	3.34683800	1.18169100	0.37593300
H	3.35748100	1.33153300	1.46595100
H	3.64767900	0.13684900	0.19952700
C	4.34880800	2.12252900	-0.27702800
H	4.03649800	3.16229500	-0.09915200
H	4.32326700	1.97762400	-1.36731500
C	5.75905300	1.90674600	0.24158300
H	6.10025600	0.88174900	0.04690200
H	6.47393100	2.58867600	-0.23183000
H	5.81287100	2.07157100	1.32531600
H	1.52507200	-0.17159300	2.55681900

C-IM4a

M06/BSI SCF energy in THF: -953.4875631 a.u.

M06/BSII SCF energy in THF: -953.6721605 a.u.

M06/BSII free energy in THF: -953.2110495 a.u.

C	2.94564700	-0.47503100	-1.22745500
C	2.73945200	-1.88766000	-1.10620400
C	2.97468000	-2.27370000	0.23259600
C	3.29439100	-1.08818200	0.98031000
C	3.30303300	0.01577800	0.05856500
Ru	1.30856300	-0.79715900	0.25226800
C	0.52916000	0.65704200	1.69184500
H	2.88432000	-3.27674800	0.63241000
H	2.40667000	-2.54483800	-1.90265600
H	2.84999600	0.10493600	-2.13733300
H	3.52107100	1.04754800	0.31166100
H	3.55028900	-1.04335100	2.03293100
C	-0.23561100	-0.58251400	1.69789700
C	-1.19266600	-0.03862700	0.61260800
C	0.26823100	-1.85502500	1.95487800
H	-0.30197100	-2.74120200	1.68161900
H	1.01873900	-1.99019100	2.73281900
C	-1.25343100	-0.90598800	-0.64289700
H	-0.22635900	-1.27664500	-0.93844300
H	-1.53013100	-0.27717300	-1.50273200
C	-2.54572200	0.42012200	1.14875200
H	-3.09558200	-0.46303100	1.50949600
H	-2.36422900	1.04876400	2.03500900
C	-2.14366800	-2.13769700	-0.56941200
H	-3.19620900	-1.81503800	-0.57828200
H	-1.99835600	-2.66012700	0.38921800
C	-1.89521600	-3.10634500	-1.71531300

H	-0.84057500	-3.42820200	-1.68706000
H	-2.02802100	-2.57964000	-2.67270800
C	-2.80627600	-4.31885600	-1.65836800
H	-3.86156900	-4.02264300	-1.71336600
H	-2.61286100	-5.01264700	-2.48366100
H	-2.66978800	-4.87179400	-0.71994200
C	-3.41180100	1.18133000	0.15484600
H	-2.86212800	2.05047000	-0.23951300
H	-3.64088000	0.54550100	-0.71510000
C	-4.71490100	1.65722300	0.78076800
H	-4.48565700	2.26454400	1.66967700
H	-5.27854400	0.78583100	1.14700500
C	-5.56793600	2.45859200	-0.18584300
H	-6.50388500	2.79230100	0.27611700
H	-5.03474800	3.35231200	-0.53532400
H	-5.82945000	1.86553600	-1.07193000
C	-0.10985800	1.06544800	0.50548900
C	-0.07652700	2.30501300	-0.30878400
H	-0.44124800	2.08477500	-1.32412000
H	-0.81772300	2.99980800	0.12598700
C	1.27708200	2.99865500	-0.37499900
H	1.68813900	3.11655500	0.64085900
H	1.98603500	2.35815400	-0.92300600
C	1.19932400	4.35937500	-1.04991900
H	0.52947300	5.01244900	-0.47079500
H	0.73149100	4.24548000	-2.03977900
C	2.56206600	5.01279100	-1.19478100
H	3.23237600	4.39798600	-1.80973200
H	2.49283700	5.99900700	-1.66690100

H	3.04327800	5.14746600	-0.21706200
H	1.27172000	1.08837000	2.35739300

3a

M06/BSI SCF energy in THF: -665.3162251 a.u.

M06/BSII SCF energy in THF: -665.4688858 a.u.

M06/BSII free energy in THF: -665.0893058 a.u.

C	1.13823200	-1.54138500	1.51072200
C	-0.30378300	-1.47841600	1.74372200
C	-0.41892300	-0.41301500	0.63615800
C	-1.18221700	-2.03930300	2.57600700
H	-2.23709900	-1.76875500	2.55818000
H	-0.87458300	-2.77934800	3.31349700
C	-1.12978400	-0.89630400	-0.62937900
H	-0.72599600	-1.88914600	-0.88700000
H	-0.85732700	-0.23573900	-1.46959900
C	-0.90526500	0.94300900	1.14288500
H	-1.93684200	0.83776900	1.51724100
H	-0.29690900	1.21670600	2.02097300
C	-2.64609100	-0.98759400	-0.53588100
H	-3.07797700	0.00536900	-0.33226500
H	-2.92277600	-1.62185500	0.32151200
C	-3.27672900	-1.55133500	-1.80126500
H	-2.85649900	-2.54907800	-2.00021800
H	-2.98724100	-0.92588600	-2.66008500
C	-4.79030200	-1.63652100	-1.71206200
H	-5.23379000	-0.64537200	-1.54813600
H	-5.23333800	-2.04900300	-2.62597200

H	-5.10122300	-2.27546000	-0.87505100
C	-0.85714400	2.07633000	0.12690300
H	0.15439100	2.15374700	-0.30543600
H	-1.52782900	1.85644600	-0.71948700
C	-1.24768200	3.41654300	0.73382300
H	-0.57107300	3.64513900	1.57158800
H	-2.25302200	3.33171000	1.17488400
C	-1.22027400	4.55097300	-0.27495400
H	-1.50467300	5.50840500	0.17658100
H	-0.21790000	4.67348800	-0.70640500
H	-1.91137300	4.35958600	-1.10640700
C	1.10372000	-0.58742000	0.55367700
C	2.11485600	0.05778700	-0.31423500
H	1.74960200	0.05101700	-1.35578800
H	2.18659400	1.12768200	-0.05067900
C	3.49299400	-0.58243900	-0.23750300
H	3.84475400	-0.56566800	0.80629000
H	3.41593400	-1.64601300	-0.51363900
C	4.51421300	0.10444600	-1.13075400
H	4.59760600	1.16246800	-0.83851500
H	4.14488800	0.10619600	-2.16787100
C	5.87888900	-0.55834400	-1.07082100
H	5.82392800	-1.60578500	-1.39507100
H	6.60964700	-0.05200800	-1.71174400
H	6.27623100	-0.55392700	-0.04742200
H	1.93562300	-2.13475200	1.95377500

C-TS4a

M06/BSI SCF energy in THF: -953.4459819 a.u.

M06/BSII SCF energy in THF: -953.6307448 a.u.

M06/BSII free energy in THF: -953.1744408 a.u.

C	1.33723800	-2.83179600	1.22352900
C	1.13725100	-3.39849700	-0.03515300
C	-0.27217500	-3.64600200	-0.19490000
C	-0.93919700	-3.29178000	1.02513400
C	0.06069600	-2.73129600	1.87409100
Ru	-0.18986100	-1.48555200	0.05744600
C	-1.02875000	0.64372500	-1.10202800
H	-0.73416100	-4.08867800	-1.07038600
H	1.89193900	-3.56138200	-0.79563800
H	2.27490500	-2.46673700	1.62542900
H	-0.10082600	-2.31136600	2.86157200
H	-1.98725500	-3.43588100	1.25801400
C	0.42154700	0.21036000	-1.08227800
C	1.22161800	0.44742800	0.07305900
C	0.72360200	-0.93176800	-1.87325900
H	1.73711900	-1.31769300	-1.94101600
H	0.07495100	-1.17745200	-2.71153600
C	2.67450600	0.05439900	0.11090500
H	2.86436000	-0.88969400	-0.41227400
H	2.97323400	-0.10247700	1.15699800
C	0.80793000	1.48173400	1.09056600
H	-0.10163900	1.17219900	1.62336100
H	1.59617200	1.55684900	1.85188300
C	3.56806000	1.13644200	-0.50595600
H	3.46001700	2.08249200	0.04601800
H	3.23389400	1.33656500	-1.53705700

C	5.03154400	0.71952800	-0.51170000
H	5.13346100	-0.23047800	-1.05805100
H	5.35164700	0.51288100	0.52070200
C	5.92972000	1.77329000	-1.13408000
H	5.86225600	2.72351200	-0.58904100
H	6.98080200	1.46389500	-1.13180400
H	5.64511800	1.97154500	-2.17549100
C	0.56957100	2.86528200	0.47742700
H	1.43194800	3.14545400	-0.14656700
H	-0.29883700	2.84009900	-0.19906200
C	0.33618100	3.92962700	1.53974800
H	1.19662000	3.95113700	2.22495000
H	-0.53432700	3.64756000	2.15109000
C	0.12254900	5.30451500	0.93169800
H	-0.03966400	6.06880700	1.69988500
H	0.99218600	5.61271200	0.33692000
H	-0.74998800	5.31348200	0.26570900
C	-1.73351600	-0.23007900	-0.34701600
C	-3.19100400	-0.24266700	-0.04398400
H	-3.61666600	-1.19179200	-0.40889500
H	-3.31943300	-0.28387800	1.05015600
C	-3.97838800	0.93238600	-0.60956000
H	-3.53553600	1.87636200	-0.25111300
H	-3.88042700	0.94702400	-1.70677800
C	-5.45082000	0.88659700	-0.23142400
H	-5.54142700	0.87602500	0.86543500
H	-5.88328300	-0.06371800	-0.57995300
C	-6.23549700	2.05318800	-0.80469700
H	-6.18573500	2.06262900	-1.90127000

H	-7.29356200	2.01336300	-0.52246000
H	-5.83494900	3.01200300	-0.45032300
H	-1.38504900	1.49118400	-1.69775700

C-IM5a

M06/BSI SCF energy in THF: -953.4805186 a.u.

M06/BSII SCF energy in THF: -953.6650105 a.u.

M06/BSII free energy in THF: -953.2050695 a.u.

C	2.02209800	-2.08378200	-1.50487100
C	0.69856100	-2.61714600	-1.36658100
C	-0.15938600	-1.91465800	-2.25497200
C	0.60016300	-0.90856500	-2.90141500
C	1.95650200	-1.01510100	-2.44357700
Ru	0.74365200	-0.48520800	-0.67096600
C	0.62573400	1.41654900	0.46526800
H	-1.22666500	-2.07341100	-2.36494000
H	0.40959200	-3.43166000	-0.71229600
H	2.90616100	-2.42928800	-0.98160200
H	2.78636600	-0.39814400	-2.76831800
H	0.22275500	-0.18625300	-3.61634700
C	-0.30633800	0.35818800	0.90275300
C	0.36389300	-0.74730600	1.50934300
C	-1.41083900	0.05845700	0.03393600
C	1.85979400	0.88935200	0.10975100
C	3.23855200	1.38959900	0.07822100
H	3.23586400	2.49065800	0.06727500
H	3.76349200	1.03355000	-0.81800100
C	3.98496100	0.89709300	1.32945600

H	3.97825200	-0.20411500	1.33988300
H	3.44249600	1.22313300	2.23026400
C	5.41669600	1.40819500	1.37424300
H	5.94417900	1.08562200	0.46423300
H	5.40794100	2.50782600	1.34989600
C	6.15863800	0.92088200	2.60580900
H	5.66330700	1.25709100	3.52566600
H	7.18940800	1.29127400	2.62982700
H	6.19996000	-0.17557100	2.63567800
H	0.31237800	2.44025100	0.24472100
H	1.22203800	-0.56079500	2.14918500
H	-0.18508500	-1.66043900	1.72694000
C	-1.99524500	1.10024400	-0.88342200
H	-2.36032600	0.59035800	-1.78831700
H	-1.22681500	1.80917000	-1.21691800
C	-2.31632700	-1.11273200	0.34374900
H	-3.05308900	-1.20127800	-0.46809600
H	-1.76510600	-2.06200800	0.35363400
C	-3.05652800	-0.96977200	1.67876800
H	-3.52500500	0.02328900	1.75272400
H	-2.33348000	-1.02528200	2.50628100
C	-4.11842900	-2.04441300	1.85531300
H	-4.85852100	-1.95650300	1.04540600
H	-3.65472400	-3.03594100	1.73704200
C	-4.81240600	-1.95346800	3.20286600
H	-5.29257600	-0.97514900	3.33481900
H	-5.58715000	-2.72012800	3.31507600
H	-4.09759300	-2.08149300	4.02591700
C	-3.15215900	1.89296000	-0.26774900

H	-3.97948800	1.21733400	-0.00330000
H	-2.81628900	2.35908600	0.67323300
C	-3.66670700	2.96407100	-1.21766300
H	-2.83920200	3.63740600	-1.48792000
H	-3.99103500	2.48793900	-2.15534800
C	-4.81071800	3.76398500	-0.62018900
H	-5.18185700	4.52544000	-1.31527700
H	-5.65634700	3.11387900	-0.36076000
H	-4.49757500	4.27751300	0.29799500

C-TS5a

M06/BSI SCF energy in THF: -953.4631083 a.u.

M06/BSII SCF energy in THF: -953.6476652 a.u.

M06/BSII free energy in THF: -953.1920442 a.u.

C	1.52436600	-3.13494800	-0.09070100
C	0.10796300	-3.26088400	0.11479000
C	-0.54051400	-2.87273200	-1.09145100
C	0.44685100	-2.45126600	-2.01863100
C	1.73240700	-2.63132300	-1.40404500
Ru	0.63759500	-1.13235600	-0.23643500
C	0.89632500	1.04461100	-0.43482700
H	-1.61257000	-2.82988100	-1.25129600
H	-0.37811000	-3.61847900	1.01537300
H	2.29563700	-3.36893500	0.63382600
H	2.69007800	-2.41488100	-1.86384300
H	0.26527400	-2.07386300	-3.01822300
C	-0.16898300	0.66875700	0.49220400
C	0.62555300	0.18868400	1.62360100

C	-1.37070000	0.05781100	0.06318200
C	1.97065400	0.29512600	0.08217200
C	3.43036200	0.46650800	0.12178700
H	3.74752000	0.43459800	-0.93637700
H	3.90250200	-0.39960400	0.60500400
C	3.89371000	1.77953600	0.74967800
H	3.57947100	1.81314900	1.80429100
H	3.38565300	2.61660000	0.24667300
C	5.40062900	1.96035000	0.65875700
H	5.89880000	1.10977400	1.14759800
H	5.70357100	1.92455800	-0.39860000
C	5.86121700	3.26306800	1.28749400
H	5.39195300	4.12590400	0.79733200
H	6.94675800	3.38986100	1.21255100
H	5.59536000	3.30444700	2.35160000
H	0.87577400	1.70190400	-1.30027700
H	1.32665400	0.90293700	2.05971000
H	0.25862300	-0.53034500	2.35818400
C	-1.97515000	0.44698300	-1.26596700
H	-2.83529600	-0.21072300	-1.46033400
H	-1.25706900	0.26925500	-2.07854700
C	-2.28938300	-0.58288100	1.06897400
H	-2.77172000	-1.45558300	0.60416500
H	-1.72471400	-0.95528900	1.93394800
C	-3.37395300	0.37049100	1.57785900
H	-4.02903900	0.67524200	0.74780700
H	-2.90114000	1.29126400	1.95625400
C	-4.21870500	-0.25793500	2.67662100
H	-4.66981300	-1.18794800	2.29856800

H	-3.56574700	-0.55308800	3.51196700
C	-5.30380700	0.67951100	3.17529500
H	-5.98954200	0.95859400	2.36503100
H	-5.90118500	0.22164800	3.97150800
H	-4.87379100	1.60711700	3.57485900
C	-2.42795600	1.90887900	-1.34345700
H	-3.07643400	2.15628600	-0.49034100
H	-1.55051300	2.56904400	-1.25981200
C	-3.16538300	2.19982800	-2.64194600
H	-2.52648200	1.91864700	-3.49329300
H	-4.05284600	1.55187000	-2.70227400
C	-3.57909800	3.65538800	-2.76264500
H	-4.12072000	3.84752300	-3.69560800
H	-4.23381400	3.95076400	-1.93261000
H	-2.70567300	4.31995100	-2.74413100

C-IM6a

M06/BSI SCF energy in THF: -953.4895837 a.u.

M06/BSII SCF energy in THF: -953.6744935 a.u.

M06/BSII free energy in THF: -953.2162055 a.u.

C	1.49789100	-3.21144400	0.18535100
C	0.07387700	-3.33701600	0.37353900
C	-0.55956200	-2.94427800	-0.83125300
C	0.45078000	-2.53260600	-1.75657100
C	1.72694800	-2.73743900	-1.13220800
Ru	0.58578200	-1.23177400	-0.03514900
C	0.92812300	0.82434800	-0.81786600
H	-1.62959600	-2.88923400	-1.00100900

H	-0.42393900	-3.67224800	1.27611000
H	2.25826800	-3.43348200	0.92470500
H	2.69195900	-2.52083000	-1.57627900
H	0.28340700	-2.17143200	-2.76484800
C	-0.12599800	0.73982000	0.18892900
C	0.92788500	0.62034100	1.28975900
C	-1.36899700	0.10631800	0.08945400
C	1.92521700	0.56733200	0.14431000
C	3.40775500	0.58257600	0.12973000
H	3.75912400	0.26163100	-0.86201500
H	3.79126400	-0.14748500	0.85818600
C	3.97486000	1.96705400	0.45157900
H	3.61656000	2.28868200	1.44214100
H	3.58038500	2.69722400	-0.27204900
C	5.49526000	1.98800900	0.42588500
H	5.87909800	1.25360600	1.15015400
H	5.84339100	1.65050000	-0.56220700
C	6.06016000	3.36348800	0.73249900
H	5.71171200	4.10522600	0.00222700
H	7.15564400	3.36733700	0.71283200
H	5.74522500	3.70906100	1.72580600
H	0.93918100	1.01523200	-1.88626100
H	1.02156000	1.39448900	2.05970600
H	0.84878800	-0.34787800	1.88770800
C	-2.11731900	0.16503500	-1.22161500
H	-3.00275700	-0.48503600	-1.15369000
H	-1.49750700	-0.23443600	-2.03631600
C	-2.15491100	-0.23787200	1.32660100
H	-2.57603800	-1.24787800	1.19826100

H	-1.48686200	-0.28465500	2.19797600
C	-3.30322500	0.72470700	1.63663200
H	-4.01863100	0.74070600	0.80009700
H	-2.91058200	1.74972600	1.73068600
C	-4.03318000	0.33761800	2.91420800
H	-4.39994100	-0.69609700	2.82148100
H	-3.31963700	0.33398700	3.75209200
C	-5.19038500	1.27043500	3.22370500
H	-5.93100900	1.26397800	2.41366000
H	-5.70759400	0.98593100	4.14681500
H	-4.84344500	2.30500400	3.34308200
C	-2.55004500	1.58427100	-1.60179300
H	-3.06985400	2.06627600	-0.76024900
H	-1.65284500	2.19351300	-1.79325900
C	-3.45072000	1.60033900	-2.82703400
H	-2.94269400	1.08817800	-3.65874200
H	-4.35533500	1.01063000	-2.61416600
C	-3.83577200	3.00916200	-3.24248300
H	-4.49225200	3.01134500	-4.11977000
H	-4.36306100	3.53007300	-2.43276900
H	-2.94822100	3.60522200	-3.49170300

A-TS6a

M06/BSI SCF energy in THF: -1573.195372 a.u.

M06/BSII SCF energy in THF: -1573.486084 a.u.

M06/BSII free energy in THF: -1572.83557 a.u.

C	2.55888500	-2.49291400	1.46730500
C	2.51703200	-1.31353000	2.26765300

C	3.51384100	-0.42226600	1.78188800
C	4.14555300	-1.01753400	0.65604300
C	3.55089600	-2.31966400	0.46493700
Ru	2.05187300	-0.77352900	0.10563400
C	-0.33865100	-0.83439000	-0.27405100
H	3.71410600	0.56876200	2.17206400
H	1.86145400	-1.13166600	3.11182000
H	1.90672900	-3.35251500	1.57677400
H	3.82339500	-3.03523000	-0.30185800
H	4.95044200	-0.59153900	0.06794500
C	1.84955500	1.24214100	0.03934900
C	0.41635500	1.08241600	0.00485300
C	2.59773500	-0.51580000	-3.05804900
N	2.36529200	-0.58463800	-1.92698600
C	2.88410400	-0.42503500	-4.46928400
H	3.90861200	-0.07249300	-4.62042800
H	2.19215300	0.27935600	-4.94074100
H	2.76923000	-1.40649400	-4.93888000
C	2.73023300	2.23358800	-0.01840600
H	2.42334700	3.28086600	-0.03164000
H	3.80324900	2.04220100	-0.03384300
C	-0.17207300	1.61875500	-1.30049800
H	-1.13535600	1.14881200	-1.51013100
H	0.49381400	1.33833300	-2.12801400
C	-0.30898700	1.40684800	1.31347500
H	-1.37793700	1.54441900	1.09743400
H	-0.24407400	0.54652200	1.99078500
C	-0.37724000	3.13001800	-1.30779500
H	0.58123400	3.65510100	-1.17925600

H	-1.01567700	3.42898400	-0.46142400
C	-1.03472200	3.58515100	-2.60398200
H	-2.00348200	3.07155700	-2.71337300
H	-0.42047700	3.25998400	-3.45770400
C	-1.23570600	5.08890500	-2.65538000
H	-0.27701300	5.61848600	-2.58079100
H	-1.71706400	5.40479500	-3.58773100
H	-1.86551300	5.43254900	-1.82454000
C	0.19170400	2.62933300	2.07644300
H	1.22009500	2.45289500	2.42816500
H	0.24051200	3.51299900	1.42197200
C	-0.70887200	2.92943900	3.26663400
H	-0.77242600	2.03413200	3.90555200
H	-1.73264200	3.11575700	2.90665500
C	-0.22478500	4.11525700	4.08100200
H	-0.88371500	4.32137600	4.93199400
H	0.78385300	3.93996200	4.47724200
H	-0.18212500	5.02486300	3.46782200
C	0.42381200	-1.85783200	-0.47890300
H	0.35582600	-2.91049100	-0.72827400
Si	-2.23509800	-1.05574400	0.04262300
C	-2.40667800	-1.31736500	1.93709100
H	-2.41764400	-0.29994100	2.36539000
C	-2.63346000	-2.68028200	-0.89462900
H	-2.10371900	-3.45519100	-0.31209400
C	-3.69051400	-2.02821600	2.37357300
H	-3.66471600	-3.09038000	2.09409100
H	-3.78508900	-1.99455900	3.46810800
H	-4.60446000	-1.59429000	1.95439400

C	-1.22193900	-2.07229900	2.53777200
H	-1.37347200	-2.23074100	3.61503900
H	-1.11056100	-3.06812600	2.08189200
H	-0.26996500	-1.54647700	2.40778200
C	-3.33759800	0.39620900	-0.55090700
H	-2.80120300	1.32579600	-0.30549900
C	-4.67335600	0.45784900	0.19348200
H	-5.26320100	1.31514900	-0.16067600
H	-5.28061500	-0.44030200	0.01931600
H	-4.54706200	0.57846500	1.27628500
C	-3.59012200	0.39770400	-2.05939500
H	-4.21081100	-0.45626000	-2.35957200
H	-4.13261400	1.30623100	-2.35792700
H	-2.66884100	0.35551400	-2.65608900
C	-4.12556300	-3.02689000	-0.87295800
H	-4.71101200	-2.31870800	-1.47420900
H	-4.28770000	-4.02077100	-1.31439600
H	-4.55885300	-3.04451500	0.13164000
C	-2.14384600	-2.75443500	-2.34276700
H	-1.08806400	-2.48915700	-2.46503300
H	-2.27692800	-3.77129500	-2.73869300
H	-2.72565400	-2.08554200	-2.99004400

A-TS6b

M06/BSI SCF energy in THF: -1573.215048 a.u.

M06/BSII SCF energy in THF: -1573.505972 a.u.

M06/BSII free energy in THF: -1572.859674 a.u.

C	-1.24234700	-2.81628200	-0.84063500

C	-0.01649500	-3.16005600	-0.20102500
C	1.02669000	-3.05697900	-1.16797600
C	0.45762500	-2.59987500	-2.39597200
C	-0.95428100	-2.44544300	-2.17682900
Ru	0.18840900	-1.03615900	-0.90203500
C	0.14024800	0.19783600	1.04024900
H	2.07613800	-3.26441900	-0.98978500
H	0.10087400	-3.46462900	0.83319800
H	-2.21711000	-2.78117800	-0.36735200
H	-1.67047000	-2.10265400	-2.91536400
H	0.97789200	-2.43557500	-3.33233200
C	2.10955500	-0.40006500	-0.99230700
C	1.93356800	0.17340200	0.32156000
C	-0.39340500	1.52443900	-2.77292900
N	-0.17012000	0.63910400	-2.06244800
C	-0.68531500	2.62631200	-3.65742600
H	-0.12173300	3.51189600	-3.34828700
H	-1.75559100	2.85488400	-3.62668800
H	-0.40566300	2.36610400	-4.68272000
C	3.05443700	-0.49201200	-1.91841500
H	4.05999900	-0.10073900	-1.75897400
H	2.87042300	-0.98279800	-2.87375000
C	2.05543300	1.70481700	0.33332200
H	1.70020400	2.08288900	1.30403100
H	1.38048300	2.11687700	-0.43051700
C	2.62651800	-0.56859000	1.47297800
H	2.58373700	0.08290800	2.36140600
H	2.04759800	-1.46894000	1.72420200
C	3.45991400	2.25244000	0.10351800

H	3.87802600	1.86907900	-0.83885600
H	4.13257800	1.92119800	0.90882300
C	3.45155000	3.77545400	0.05849400
H	3.01323500	4.16468900	0.99001200
H	2.78560100	4.10799900	-0.75271800
C	4.84030500	4.35559000	-0.14091000
H	5.28853900	3.99689000	-1.07653300
H	4.82197300	5.45054800	-0.18054600
H	5.51169500	4.06525000	0.67738600
C	4.07058400	-0.98644300	1.22407800
H	4.11140300	-1.71367800	0.39925800
H	4.67717200	-0.12684100	0.90300700
C	4.69361300	-1.60115000	2.46973900
H	4.08505500	-2.45953900	2.79358700
H	4.65343200	-0.87268400	3.29368800
C	6.12855400	-2.04121400	2.24117700
H	6.56751600	-2.48303900	3.14283000
H	6.19121900	-2.78982900	1.44076600
H	6.76010300	-1.19261100	1.94771000
C	-1.01753100	-0.02751500	0.51392100
H	0.47329900	0.54084400	2.01634500
Si	-2.81446300	0.42456400	0.84411500
C	-2.81641300	1.76479200	2.19141100
H	-3.87716500	2.05303300	2.29846500
C	-3.75463000	-1.14584800	1.34832300
H	-3.87692800	-1.71725000	0.41181200
C	-3.49538500	1.18063600	-0.76538200
H	-2.66915200	1.81616500	-1.13095000
C	-5.15306600	-0.81451600	1.86926000

H	-5.71632300	-1.73073300	2.09605700
H	-5.10889500	-0.22879700	2.79788300
H	-5.74511600	-0.23849200	1.14653300
C	-3.00215800	-2.02318900	2.34617200
H	-1.97961200	-2.25486400	2.01902600
H	-2.92276600	-1.53580400	3.32684000
H	-3.52548200	-2.97697800	2.50411000
C	-2.03342700	3.00126300	1.74748000
H	-2.09047900	3.79395100	2.50677400
H	-0.96834800	2.76819600	1.60317500
H	-2.40383200	3.42732500	0.80564500
C	-2.32033500	1.27789500	3.55052200
H	-2.29551400	2.10305900	4.27668800
H	-2.96077200	0.49447000	3.97280800
H	-1.30015500	0.87096100	3.49288500
C	-4.71774000	2.07304400	-0.55436600
H	-5.03078800	2.53407400	-1.50222300
H	-5.57942500	1.49980300	-0.18477000
H	-4.53653200	2.88638100	0.15887700
C	-3.78925800	0.12985400	-1.83182900
H	-4.02542100	0.59964100	-2.79804700
H	-2.94373400	-0.55084500	-1.99490800
H	-4.65871400	-0.48437900	-1.55664400

B-TS6a

M06/BSI SCF energy in THF: -1573.206304 a.u.

M06/BSII SCF energy in THF: -1573.49787 a.u.

M06/BSII free energy in THF: -1572.845602 a.u.

C	-1.49051200	-3.43864900	-0.50266800
C	-1.82591900	-2.70309100	-1.67419800
C	-3.00964300	-1.95903000	-1.40071900
C	-3.36782200	-2.17492500	-0.04661800
C	-2.42316900	-3.10441200	0.51818100
Ru	-1.35773000	-1.24977800	-0.00775500
C	0.99270700	-0.86034300	0.16320600
H	-3.52414000	-1.31024600	-2.09902400
H	-1.28901700	-2.72774000	-2.61546900
H	-0.63381700	-4.09514100	-0.39475300
H	-2.43425000	-3.49253900	1.53015500
H	-4.21639800	-1.74045800	0.46967000
C	-0.17619000	-0.00719500	-1.31991200
C	0.41758500	-0.22596200	-2.49505600
C	-1.82030600	0.08020500	2.88924100
N	-1.65036300	-0.32897700	1.82014100
C	-2.02585500	0.60776200	4.21678900
H	-2.78614300	1.39442400	4.19349600
H	-1.08917700	1.02748300	4.59627300
H	-2.35743400	-0.18840600	4.88986900
C	-1.19327200	0.84792100	-0.82206400
C	0.44396400	-1.77762700	0.86569400
C	-0.79578300	1.91686600	0.18042500
H	-1.65411500	2.19729700	0.80949900
H	-0.03254400	1.51780700	0.86392700
C	-2.22520800	1.30949200	-1.84617100
H	-2.51803600	0.47346800	-2.49317800
H	-1.69503500	2.01189100	-2.51376800
C	-3.45966500	2.02836100	-1.30757700

H	-3.15406200	2.92699600	-0.74875800
H	-4.03266700	2.40330000	-2.16908200
C	-4.38408500	1.20000100	-0.42854800
H	-4.74657400	0.32934700	-0.99623400
H	-3.81707900	0.79526500	0.42333100
C	-0.24202600	3.17440100	-0.49385900
H	0.52042200	2.88750400	-1.23717200
H	-1.03171300	3.68853800	-1.06123600
C	0.36432700	4.15576600	0.50052300
H	-0.42187900	4.51876100	1.18013100
H	1.09628000	3.63261100	1.13840500
C	1.04336900	5.32378000	-0.19171900
H	0.34111900	5.86913400	-0.83550100
H	1.46187700	6.03990200	0.52452700
H	1.86651000	4.97206800	-0.82998300
C	-5.56762000	2.00867600	0.07490800
H	-6.15972800	2.40698600	-0.75929900
H	-6.23912700	1.40763200	0.69897900
H	-5.23526800	2.86575500	0.67564800
H	0.34692000	0.52698600	-3.28279700
H	1.00841400	-1.11630300	-2.70575300
H	0.59185600	-2.52031300	1.63845100
Si	2.78852600	-0.23807700	0.00945900
C	3.62075100	-1.27718800	-1.34661900
H	3.30066600	-0.81399100	-2.29443100
C	3.21295400	-2.74656100	-1.38631900
H	2.13212200	-2.87325200	-1.54245100
H	3.72682800	-3.26964900	-2.20542100
H	3.47311700	-3.27372300	-0.45783500

C	5.14014300	-1.12286500	-1.24485900
H	5.45462700	-0.07005400	-1.24160700
H	5.53243800	-1.58415200	-0.32830500
H	5.64383800	-1.61102400	-2.09086500
C	3.48407400	-0.54948300	1.75637500
H	4.46664700	-0.04734400	1.73592000
C	3.73100200	-2.01048300	2.11996600
H	4.14104700	-2.09176800	3.13700800
H	2.80666200	-2.60284000	2.10226600
H	4.44462700	-2.49436300	1.44300600
C	2.63466200	0.13363100	2.83037000
H	2.41877000	1.18755000	2.60444300
H	1.66747200	-0.37456900	2.95198300
H	3.14166500	0.10751200	3.80507400
C	2.94463300	1.62231500	-0.39049000
H	2.00919100	2.07291700	-0.02906300
C	4.09364400	2.30084600	0.35917900
H	5.07003200	1.89425300	0.05923500
H	4.11145000	3.37551200	0.12561900
H	4.01742100	2.20849700	1.44886000
C	3.07691200	1.93230100	-1.88214200
H	3.09519300	3.02016600	-2.04636500
H	4.01730100	1.53468800	-2.28992300
H	2.25510900	1.52271900	-2.48160600

B-TS6b

M06/BSI SCF energy in THF: -1573.211339 a.u.

M06/BSII SCF energy in THF: -1573.502693 a.u.

M06/BSII free energy in THF: -1572.854101 a.u.

C	0.10541800	-2.67322400	-1.60143200
C	-1.29596200	-2.74174200	-1.33068500
C	-1.47525400	-2.95879200	0.06464700
C	-0.17096000	-3.04314300	0.64989200
C	0.79710100	-2.89957300	-0.37201500
Ru	-0.45912000	-1.00057400	-0.26470900
C	0.46486200	0.85574400	-1.26533800
H	-2.41859700	-3.06193400	0.58909600
H	-2.07555300	-2.63010900	-2.07558200
H	0.55975400	-2.51719300	-2.57454800
H	1.87305000	-2.90519500	-0.23730900
H	0.03771600	-3.17664900	1.70636200
C	-1.26695000	0.30239700	-1.76630000
C	-1.35912000	0.24377200	-3.09868600
C	-0.34910500	0.38725200	2.64717500
N	-0.44356600	-0.07653800	1.59132800
C	-0.22011200	0.97480900	3.95916400
H	-1.20543000	1.08418500	4.42230800
H	0.24445800	1.96261100	3.87376300
H	0.40555500	0.33969000	4.59440300
C	-2.18479800	0.35197900	-0.67386100
C	1.30091700	0.16955100	-0.56702900
H	0.40562600	1.84533700	-1.70349100
C	-2.22454900	1.65455200	0.10755100
H	-2.61528200	1.49124000	1.12208200
H	-1.20800800	2.05374400	0.23073900
C	-3.53167500	-0.32262500	-0.83171100
H	-3.43238900	-1.24570700	-1.41482900

H	-4.17237100	0.33567200	-1.44641000
C	-4.24937800	-0.62090800	0.47867600
H	-3.54234100	-1.08880500	1.18540300
H	-4.58025300	0.31691800	0.95243400
C	-5.44974700	-1.53567400	0.28674500
H	-6.12802400	-1.09444600	-0.45979800
H	-5.10701600	-2.49035600	-0.14397000
C	-3.07051800	2.72064000	-0.59028000
H	-2.76426100	2.79755400	-1.64748800
H	-4.13090900	2.42491200	-0.59853600
C	-2.93863700	4.08292000	0.07450700
H	-3.23232600	3.99964000	1.13232000
H	-1.87875600	4.38186100	0.07632900
C	-3.77385700	5.14899000	-0.61202400
H	-4.84030700	4.88945200	-0.59560800
H	-3.66379200	6.12751100	-0.13092600
H	-3.48135800	5.26262100	-1.66405700
C	-6.20110000	-1.79386900	1.58009700
H	-6.58747500	-0.85914200	2.00662100
H	-7.05310900	-2.46657700	1.43139000
H	-5.54549700	-2.25133500	2.33313200
H	-2.30350300	0.48018600	-3.59101300
H	-0.49814900	0.03267900	-3.73145800
Si	3.06354200	0.33385700	0.08224200
C	3.55405000	2.14855900	-0.21069900
H	4.58719000	2.22747900	0.16922400
C	4.16098000	-0.89649600	-0.86706800
H	4.07161400	-1.85190200	-0.32068400
C	3.10489400	0.00746300	1.95691000

H	2.34627100	0.68504900	2.38383100
C	5.63171300	-0.47558700	-0.84046500
H	6.26352800	-1.23950000	-1.31491100
H	5.78905800	0.45907100	-1.39604000
H	6.01652600	-0.32096400	0.17537000
C	3.70703800	-1.13159400	-2.30651300
H	2.68020000	-1.51472200	-2.36641800
H	3.74187800	-0.20525000	-2.89672800
H	4.36196900	-1.85761200	-2.80870400
C	3.56632800	2.55557200	-1.68151700
H	3.86122000	3.60854500	-1.79441900
H	4.26683900	1.95623800	-2.27601400
H	2.57530500	2.45043700	-2.14472500
C	2.67392400	3.09261500	0.60857000
H	2.97671900	4.13921600	0.46381500
H	1.61739600	3.01922000	0.30777000
H	2.72109000	2.88695300	1.68626100
C	4.45987400	0.38221200	2.56035500
H	4.43183300	0.30396600	3.65637800
H	5.24749300	-0.30229000	2.21544900
H	4.77655300	1.40369600	2.31439500
C	2.74322900	-1.42032100	2.35249000
H	2.76554900	-1.54154500	3.44548100
H	1.74317800	-1.70622100	2.00778500
H	3.46213700	-2.14411300	1.94108600

C-TS6a

M06/BSI SCF energy in THF: -1573.218283 a.u.

M06/BSII SCF energy in THF: -1573.509631 a.u.

M06/BSII free energy in THF: -1572.861218 a.u.

C	1.29367000	-1.86476400	-2.21481200
C	-0.00933100	-1.32990500	-2.48265000
C	-0.96202600	-2.35560900	-2.20245500
C	-0.25573700	-3.51108400	-1.74751700
C	1.13159400	-3.19425300	-1.75045900
Ru	0.04361300	-1.80491500	-0.33475000
C	-0.29816100	0.05416000	0.90859700
H	-2.03937500	-2.26523800	-2.29784400
H	-0.22488500	-0.34085900	-2.87127100
H	2.23861900	-1.34967300	-2.33816700
H	1.92884400	-3.84605800	-1.40829500
H	-0.69183700	-4.45462800	-1.44242300
C	-1.83941500	-1.03723600	0.37504000
C	-2.81903100	-0.27502000	-0.14097700
C	1.83272400	-2.69392700	2.23038000
N	1.14355700	-2.42886100	1.33938800
C	2.71394500	-3.00961400	3.32858400
H	3.74559600	-3.06939300	2.96712400
H	2.43434400	-3.96821500	3.77514000
H	2.64895400	-2.22713300	4.09124100
C	-1.66633300	-2.35855600	0.85278800
H	-2.24886500	-3.15782200	0.38979700
H	-1.39271500	-2.53552000	1.89469200
C	-4.24829400	-0.69364600	0.09470200
H	-4.27304600	-1.59722500	0.71930400
H	-4.67130300	-0.98030400	-0.88531100
C	-2.59112100	0.96535000	-0.96179300

H	-1.68738800	0.85033600	-1.57686800
H	-3.43280100	1.06781900	-1.66566500
C	-5.16234500	0.36446700	0.70744200
H	-5.18932500	1.25863700	0.06492400
H	-4.75342200	0.69314500	1.67629000
C	-6.58003500	-0.15253400	0.90290900
H	-6.55452300	-1.04416300	1.54770500
H	-6.97581300	-0.49299400	-0.06638600
C	-7.50445800	0.89237800	1.50178800
H	-7.57086700	1.77870600	0.85749800
H	-8.52082300	0.50652900	1.63985300
H	-7.14104800	1.22755800	2.48208300
C	-2.46008400	2.27060400	-0.16938600
H	-3.27417800	2.36384300	0.56445800
H	-1.52615700	2.24956800	0.41260000
C	-2.45270400	3.49014300	-1.07911700
H	-3.42536800	3.56736700	-1.58909900
H	-1.70378200	3.34458200	-1.87406500
C	-2.15430900	4.77169000	-0.32239200
H	-2.16389200	5.64900600	-0.97890800
H	-2.89174100	4.94415200	0.47247500
H	-1.16518800	4.72546200	0.15476900
C	0.80426300	0.12645500	0.25847700
H	-0.77006100	0.42012600	1.81232800
C	1.92411200	2.63975100	1.32851800
Si	2.33814500	1.20707200	0.14045400
C	2.43522700	1.73747700	-1.68038200
H	2.39296900	0.79239600	-2.24823200
C	3.82638500	0.19600100	0.75560500

H	3.49602600	-0.21125000	1.72761300
C	2.71600000	3.92592800	1.10127600
H	2.41073000	4.69777700	1.82263600
H	3.79641900	3.77710900	1.23407800
H	2.56187200	4.34207600	0.09800100
C	3.71642500	2.46335900	-2.08472700
H	3.86622600	3.38642200	-1.50854400
H	4.60878000	1.83957500	-1.94860600
H	3.68121000	2.75152000	-3.14526500
C	1.19759300	2.55536900	-2.05332800
H	1.14621700	2.72834300	-3.13767400
H	0.26251200	2.06053900	-1.75095900
H	1.21248400	3.54355100	-1.57240600
C	5.06786100	1.05212900	1.00930700
H	5.43190600	1.52944100	0.08957700
H	4.89059300	1.84763600	1.74329700
H	5.89145100	0.43377200	1.39407600
C	4.16451900	-0.98185400	-0.15450400
H	4.46874000	-0.64199500	-1.15536800
H	5.00537200	-1.56177700	0.25373700
H	3.31543900	-1.66771600	-0.27536300
C	2.03392100	2.19341000	2.78670800
H	3.08012300	2.01455400	3.07153100
H	1.64626900	2.96671700	3.46472100
H	1.47780300	1.26838100	2.99387700
H	0.86188600	2.86059500	1.12250400

C-TS6b

M06/BSI SCF energy in THF: -1573.214989 a.u.

M06/BSII SCF energy in THF: -1573.506338 a.u.

M06/BSII free energy in THF: -1572.855275 a.u.

C	-1.76424700	-3.32154600	1.19080300
C	-0.38534000	-3.25767500	0.81137600
C	-0.31189300	-3.51067100	-0.59502600
C	-1.63957900	-3.68719000	-1.08859900
C	-2.52912200	-3.56455700	0.02945000
Ru	-1.39633900	-1.64068400	-0.30228400
C	-0.75261200	0.43587400	0.62548400
H	0.59836500	-3.52654900	-1.18759300
H	0.45187800	-3.09472300	1.48111800
H	-2.15758400	-3.16269800	2.18813200
H	-3.61161700	-3.60847700	-0.01750500
H	-1.92681300	-3.90040800	-2.11120800
C	0.37030200	-0.50935600	-0.85144600
C	1.66916400	-0.56508600	-0.51447100
C	-4.08316700	-0.15666200	-1.36165000
N	-3.10331500	-0.66184000	-1.00972200
C	-5.29313600	0.49735400	-1.79832800
H	-5.11400300	1.02027200	-2.74305800
H	-5.61546400	1.22134700	-1.04311400
H	-6.08722800	-0.24085100	-1.94468100
C	-0.43143400	-0.73424700	-1.99285600
H	-0.10071600	-1.48466800	-2.71493200
H	-1.03317300	0.07397800	-2.41611400
C	2.70036600	-0.49358900	-1.61822900
H	2.20431700	-0.22336900	-2.56074700
H	3.10404700	-1.51158200	-1.76959300

C	2.16747600	-0.74130100	0.88864800
H	2.78030200	0.12850400	1.18221200
H	1.31582900	-0.76913200	1.58136800
C	3.87855400	0.44896600	-1.38500800
H	4.45951100	0.12929400	-0.50615600
H	3.51233300	1.46143400	-1.15500900
C	4.79638900	0.52387800	-2.59599900
H	4.20388100	0.81674500	-3.47728100
H	5.19260500	-0.47906800	-2.81731500
C	5.93777100	1.50492300	-2.39651300
H	6.55174600	1.23110100	-1.52853300
H	6.59898400	1.54744700	-3.26938600
H	5.55569500	2.51961700	-2.22040900
C	3.01166600	-2.00391100	1.07848800
H	2.47185300	-2.87811600	0.67644700
H	3.93940700	-1.93208700	0.49042900
C	3.36146000	-2.25042600	2.53835400
H	2.43351000	-2.30818200	3.12940400
H	3.91187900	-1.38086200	2.92864200
C	4.17707400	-3.51553500	2.73631900
H	4.42377500	-3.68262200	3.79079900
H	3.62854500	-4.39839200	2.38180100
H	5.12111300	-3.47131900	2.17789500
C	-1.56225500	-0.29981600	1.29122500
H	-2.20950900	-0.35591000	2.15553500
Si	-0.36930500	2.30601400	0.63425500
C	1.14517300	2.73756700	-0.42317800
H	1.86666800	1.96407800	-0.11117100
C	0.08570500	2.71508600	2.44875700

H	1.18321700	2.59161200	2.46733500
C	-1.97652200	3.17710000	0.10196900
H	-1.76133500	4.25247900	0.22920600
C	-0.23123300	4.17446300	2.78997800
H	0.18306800	4.43576700	3.77383000
H	0.17383400	4.89313500	2.06750100
H	-1.31494500	4.34406700	2.84533400
C	-0.49051000	1.80373900	3.52806400
H	-0.18759900	0.75843600	3.39996700
H	-0.14560200	2.12616400	4.52083400
H	-1.58898200	1.83496700	3.54781700
C	-2.35194600	2.93685700	-1.36067900
H	-2.39929100	1.86293900	-1.59683000
H	-3.34146200	3.36473300	-1.58011800
H	-1.64246400	3.39288500	-2.05944300
C	-3.15105500	2.81905000	1.01121300
H	-2.94591800	3.00817500	2.07254700
H	-4.04338300	3.40254600	0.74252600
H	-3.41599200	1.75573500	0.91637200
C	0.99092800	2.65432300	-1.93902000
H	0.40982100	3.50483400	-2.31894900
H	1.97694000	2.70552700	-2.42733600
H	0.50415900	1.73300300	-2.28040000
C	1.74378300	4.09754400	-0.05945200
H	2.03678900	4.17132600	0.99395700
H	2.64664100	4.28582300	-0.65943900
H	1.04419400	4.91889700	-0.27375900

D-TS6a

M06/BSI SCF energy in THF: -1573.218089 a.u.

M06/BSII SCF energy in THF: -1573.50947 a.u.

M06/BSII free energy in THF: -1572.85960 a.u.

C	-1.01957300	-1.69549700	-2.28494700
C	0.29901900	-2.22392500	-2.18067100
C	1.21389700	-1.17889200	-2.50734900
C	0.46952500	0.00243700	-2.75932300
C	-0.92356800	-0.31503300	-2.63410300
Ru	0.03953200	-0.60108700	-0.67054500
C	-0.97797400	-1.47630200	1.24005000
H	2.29365400	-1.26336900	-2.51204200
H	0.56259400	-3.24150500	-1.91522100
H	-1.93573700	-2.24314600	-2.09605800
H	-1.75076900	0.36331900	-2.80689500
H	0.88060600	0.97414300	-3.01033300
C	1.82336500	-0.86751200	0.31349400
C	0.98648600	-1.65078200	1.16154100
C	0.33020300	2.34076700	0.61847700
N	0.18771200	1.27882700	0.18214400
C	0.50837500	3.66994800	1.15219400
H	-0.45506300	4.18798800	1.19975000
H	1.18762300	4.23965600	0.50998100
H	0.93229800	3.61279400	2.15939900
C	3.06311100	-0.38351800	0.29340800
C	-1.72728800	-0.72459300	0.52038700
C	4.07491600	-0.87992200	1.30216500
H	4.35873400	-0.05189400	1.97323200
H	3.61295900	-1.64759000	1.93902100

C	3.51453100	0.67961200	-0.66992600
H	2.68253300	0.94326900	-1.33636100
H	4.32297700	0.30034800	-1.31676500
C	4.00456400	1.94740400	0.02993300
H	3.25160200	2.27306900	0.76918300
H	4.91914000	1.73421100	0.60530700
C	4.27471700	3.08213300	-0.94605100
H	4.99124700	2.73974700	-1.70824700
H	3.34549400	3.31671300	-1.49078100
C	5.34438400	-1.44777800	0.67271700
H	5.07600600	-2.23990200	-0.04540700
H	5.85697200	-0.66732000	0.08829500
C	6.30905300	-2.00450200	1.70949100
H	6.55092900	-1.21413300	2.43665100
H	5.80692000	-2.79902000	2.28228400
C	7.58515500	-2.54202100	1.08680900
H	8.11975400	-1.75483900	0.53925400
H	8.27179500	-2.94392500	1.84054800
H	7.36872000	-3.34804300	0.37363900
C	4.80437600	4.33026100	-0.26265600
H	5.75257400	4.12762400	0.25184500
H	4.98281100	5.14282600	-0.97585200
H	4.09823000	4.70213800	0.49220400
H	1.01899700	-2.73915900	1.07084700
H	0.96889200	-1.30428000	2.19803300
H	-1.03821300	-2.17006800	2.06782300
Si	-3.45815900	0.02112700	0.50912200
C	-4.36301500	-0.67191500	-1.00969900
H	-3.74216000	-0.34958100	-1.86341900

C	-3.26850100	1.91673700	0.41800400
H	-2.43088700	2.14219400	1.10189500
C	-4.26128900	-0.40818200	2.17944600
H	-5.23036600	0.12044500	2.17196000
C	-4.46347700	-2.19791600	-1.04734400
H	-4.69658100	-2.54981100	-2.06215000
H	-3.54855400	-2.70923400	-0.71757900
H	-5.27492000	-2.55217800	-0.39911400
C	-5.75109000	-0.05426600	-1.18030900
H	-6.26924400	-0.48816900	-2.04736500
H	-6.38482700	-0.24478700	-0.30172300
H	-5.71198400	1.03124700	-1.33412500
C	-2.88949700	2.42283000	-0.97151600
H	-3.69120100	2.23185400	-1.69975700
H	-2.72386600	3.51045600	-0.96088500
H	-1.97220200	1.95473600	-1.35002400
C	-4.50181900	2.66009400	0.93357600
H	-5.39066300	2.45055300	0.32318800
H	-4.75058100	2.40470200	1.97062700
H	-4.34052900	3.74708600	0.89693700
C	-3.43256300	0.14531800	3.33929700
H	-3.95041800	-0.00383100	4.29714600
H	-2.46440500	-0.36976000	3.41218300
H	-3.22455500	1.21894200	3.24425100
C	-4.53532300	-1.89380500	2.39244000
H	-4.93474900	-2.07421400	3.40085400
H	-5.26560000	-2.29229000	1.67913600
H	-3.61935700	-2.49481900	2.29872000

D-TS6b

M06/BSI SCF energy in THF: -1573.213037 a.u.

M06/BSII SCF energy in THF: -1573.503864 a.u.

M06/BSII free energy in THF: -1572.854052 a.u.

C	0.66430000	-2.56303600	-2.40206700
C	-0.01000500	-1.41447900	-2.91171500
C	-1.39415400	-1.54777200	-2.59449100
C	-1.56731500	-2.74047900	-1.84457800
C	-0.28708700	-3.38321300	-1.72486200
Ru	-0.19951100	-1.45791600	-0.68458600
C	1.80578200	-0.48315200	0.28095100
H	-2.17051000	-0.83160700	-2.83575400
H	0.44633700	-0.59817400	-3.46062900
H	1.73057000	-2.75098900	-2.47481500
H	-0.09253600	-4.33046600	-1.23625100
H	-2.50375600	-3.11083100	-1.44194400
C	-1.12616200	0.34082500	-0.34100600
C	0.21873700	0.75918000	-0.12746400
C	-1.48962900	-2.13044400	2.19231700
N	-0.97136800	-1.89077300	1.18606800
C	-2.16363300	-2.42223800	3.43459300
H	-2.16742500	-1.53396900	4.07369300
H	-1.65549500	-3.23651100	3.95949300
H	-3.19750000	-2.72010000	3.23203300
C	-2.35827200	0.81845200	-0.17648300
C	1.63544100	-1.75363200	0.21690700
C	-2.56073700	2.28239300	0.14264100
H	-2.98604200	2.37940400	1.15603900

H	-1.58599200	2.79107800	0.16868300
C	-3.58626300	-0.04660500	-0.24608400
H	-3.28832800	-1.07992800	-0.46830300
H	-4.24384700	0.27439000	-1.07114200
C	-4.39384100	-0.03931700	1.05190600
H	-3.71629800	-0.21760700	1.90583200
H	-4.83592900	0.95510800	1.22056400
C	-5.49800200	-1.08527500	1.05372700
H	-6.14145500	-0.93156500	0.17413300
H	-5.04840500	-2.08373400	0.92571400
C	-3.47827300	3.01008300	-0.83641600
H	-3.10506100	2.86971000	-1.86395100
H	-4.48355200	2.56055600	-0.81334200
C	-3.58932900	4.49687000	-0.53365500
H	-3.93128900	4.62875800	0.50440200
H	-2.58797800	4.95157900	-0.58183300
C	-4.53000900	5.21825100	-1.48271100
H	-5.54653100	4.80817700	-1.42139400
H	-4.59174300	6.28968800	-1.26068400
H	-4.19739400	5.11522600	-2.52389500
C	-6.33562700	-1.05075000	2.31960100
H	-6.82806000	-0.07726700	2.44046600
H	-7.11684200	-1.81914000	2.31449400
H	-5.71587400	-1.21388100	3.21176400
H	0.71802500	1.32503400	-0.91845500
H	0.39869900	1.13497900	0.88223900
H	2.15600600	-2.68264600	0.41549000
Si	3.24448900	0.73701100	0.49569600
C	2.80587600	2.13996700	1.71606500

H	2.06516700	1.71703900	2.41669600
C	3.65516600	1.44150100	-1.22076100
H	2.90937900	2.23347800	-1.40025100
C	5.03611500	2.10009500	-1.20776000
H	5.24562400	2.59011400	-2.16887300
H	5.13136800	2.86500700	-0.42500200
H	5.83245300	1.36118400	-1.04314600
C	3.53744200	0.42844800	-2.35653600
H	4.22158400	-0.42108100	-2.22511900
H	2.52074400	0.02071100	-2.44047000
H	3.78558900	0.89446800	-3.32082800
C	4.63485300	-0.33215100	1.22808900
H	5.42955500	0.39507300	1.46810200
C	4.02738000	2.57133600	2.53498800
H	3.75272000	3.37523400	3.23206400
H	4.45944800	1.75831300	3.12940400
H	4.82471500	2.96903300	1.89081900
C	2.19584300	3.38248600	1.06335700
H	1.94463000	4.12917900	1.82932000
H	2.90777400	3.86088800	0.37700000
H	1.27924100	3.18338100	0.49464200
C	5.22590600	-1.36091900	0.26716600
H	6.00770100	-1.95047900	0.76709300
H	4.46728400	-2.07144000	-0.09032600
H	5.68429100	-0.89639700	-0.61410500
C	4.20621600	-1.00712500	2.53185000
H	3.47737700	-1.80650100	2.34291500
H	5.06730100	-1.46943900	3.03428900
H	3.74513000	-0.31327900	3.24726600

C-IM7a

M06/BSI SCF energy in THF: -1440.585154 a.u.

M06/BSII SCF energy in THF: -1440.839594 a.u.

M06/BSII free energy in THF: -1440.227446 a.u.

C	0.06352100	-1.75807200	2.26913900
C	1.48465700	-1.83538200	2.17270000
C	1.80942200	-2.91284700	1.30931900
C	0.59035100	-3.48646200	0.83306300
C	-0.48951500	-2.77623900	1.44537000
Ru	0.64936400	-1.37233400	0.12130500
C	0.02509000	-0.38317200	-1.71286700
H	2.81008900	-3.21855900	1.02596700
H	2.19043300	-1.17611300	2.66605500
H	-0.49103500	-1.03374700	2.85510100
H	-1.54507300	-2.97466600	1.29238400
H	0.50073700	-4.32877000	0.15768800
C	1.48916800	-0.36925700	-1.47083600
C	1.99453400	0.47588400	-0.43680100
C	2.01295400	-1.69687500	-1.57935200
H	3.03499900	-1.92291000	-1.28502900
H	1.58225900	-2.36596700	-2.32210100
C	3.43612100	0.32987600	-0.00700200
H	3.69419800	-0.71178400	0.22250500
H	3.57727800	0.88719900	0.92979300
C	1.41185800	1.83527900	-0.15718300
H	2.14117600	2.57519600	-0.52975800
H	0.49259300	1.98654400	-0.73984200

C	4.41748800	0.86577100	-1.05402800
H	4.16315000	1.90536300	-1.31242100
H	4.31498000	0.28509100	-1.98440100
C	5.85907000	0.80471400	-0.57184300
H	6.11077800	-0.23582600	-0.31533700
H	5.95178600	1.37890100	0.36228800
C	6.83735900	1.33462900	-1.60495300
H	6.62154300	2.38204900	-1.85243800
H	7.87232400	1.28516900	-1.24869500
H	6.77982100	0.75900100	-2.53783200
C	1.13335000	2.14785400	1.30855000
H	0.43133300	1.39915400	1.71017500
H	2.05793600	2.05596800	1.89983800
C	0.55518300	3.54323600	1.49402300
H	-0.39294300	3.61502400	0.93536100
H	1.23214800	4.28109000	1.03753000
C	0.32278400	3.88926400	2.95371200
H	-0.10043700	4.89296800	3.07269800
H	-0.37044300	3.18044400	3.42634500
H	1.26226900	3.85622900	3.52065200
C	-0.75449200	-0.18110500	-0.58056600
H	-0.35935700	-0.78215300	-2.65888400
Si	-2.57230000	0.38653900	-0.49909600
C	-3.31440700	0.48842200	1.24050000
H	-4.28514000	0.97672800	1.04257800
C	-2.53521300	2.14763900	-1.23862600
H	-1.68303600	2.64638600	-0.74286500
C	-3.48030300	-0.84477200	-1.63777100
H	-3.13454900	-0.59737700	-2.65518500

C	-4.98904100	-0.60325000	-1.57857900
H	-5.26159600	0.43021500	-1.82722300
H	-5.51495300	-1.25809600	-2.28732800
H	-5.39129800	-0.82082200	-0.57975100
C	-3.15846500	-2.31457900	-1.37783400
H	-2.08840300	-2.53587900	-1.48827500
H	-3.46128100	-2.62765900	-0.36877700
H	-3.70079500	-2.95748900	-2.08543400
C	-2.50903900	1.41222200	2.15319500
H	-1.56133200	0.94016800	2.44926300
H	-2.26325900	2.37648000	1.68608200
H	-3.05956700	1.63001100	3.07924600
C	-3.60752800	-0.83537600	1.93811600
H	-4.11538900	-0.66271900	2.89777700
H	-4.25262100	-1.49318100	1.34277700
H	-2.68549600	-1.38622600	2.16408400
C	-3.78487300	2.96936400	-0.92271900
H	-3.69063900	3.98274200	-1.33748900
H	-4.68962100	2.52987900	-1.36522400
H	-3.96139100	3.07429500	0.15481400
C	-2.27021500	2.13908400	-2.74143400
H	-3.11148300	1.69424000	-3.29151200
H	-2.14362500	3.16150900	-3.12320700
H	-1.36581600	1.57793700	-3.01385800

C-TS7a

M06/BSI SCF energy in THF: -1440.552855 a.u.

M06/BSII SCF energy in THF: -1440.807408 a.u.

M06/BSII free energy in THF: -1440.192657 a.u.

C	0.73809000	-2.73312000	1.73038500
C	2.06761000	-2.89477800	1.20637600
C	1.97275600	-3.63167800	-0.00079000
C	0.59428000	-3.85877100	-0.28055400
C	-0.16667800	-3.33401400	0.82124600
Ru	0.86264600	-1.68921300	-0.22233200
C	-0.08586500	-0.49785900	-1.79745100
H	2.80063700	-3.89527300	-0.64913100
H	2.98164300	-2.53491900	1.66464200
H	0.48487800	-2.22595500	2.65490900
H	-1.24484700	-3.37921400	0.91583900
H	0.19247700	-4.38239800	-1.14029300
C	1.34887900	-0.25957600	-1.67921400
C	1.40653800	0.75806300	-0.60905200
C	2.27801500	-1.29377300	-1.88591800
H	3.31166300	-1.18366100	-1.55837200
H	2.10021500	-2.00457900	-2.69125900
C	2.56586300	0.78872200	0.36958600
H	2.91664300	-0.22638400	0.59017800
H	2.21299900	1.19569800	1.32875000
C	0.92010600	2.15070200	-0.98135000
H	1.72842700	2.58067400	-1.59866000
H	0.04832000	2.10173200	-1.64395800
C	3.76237400	1.60879900	-0.13214900
H	3.52080800	2.68079400	-0.16303700
H	3.99932300	1.31678500	-1.16821300
C	4.98600900	1.39232500	0.74618200
H	5.24384600	0.32172900	0.74639900

H	4.73264800	1.64433600	1.78721900
C	6.17976300	2.21078700	0.28842300
H	5.95835100	3.28528700	0.31661900
H	7.05809100	2.03660500	0.91969800
H	6.45826400	1.96073500	-0.74359200
C	0.64354900	3.09576100	0.17837500
H	-0.07936100	2.63322900	0.86788100
H	1.55318800	3.25895500	0.77379400
C	0.11915700	4.44702200	-0.29085400
H	-0.68961100	4.29876100	-1.02472600
H	0.91808300	4.97727700	-0.83016600
C	-0.39235400	5.29042400	0.86281600
H	-0.73569800	6.27608400	0.52965700
H	-1.23870100	4.79632200	1.36217500
H	0.38703100	5.44773000	1.61989800
C	-0.52083900	-0.16685100	-0.50330100
H	-0.65195500	-0.85086100	-2.65810900
Si	-2.30879800	0.25221000	0.04794700
C	-2.54545800	0.14044400	1.92582700
H	-3.51948400	0.63289200	2.08936500
C	-2.65569400	2.04614600	-0.50672100
H	-1.73555700	2.58747200	-0.25166500
C	-3.42612100	-0.93113900	-0.95400800
H	-3.32934400	-0.57113800	-1.99178100
C	-4.88324500	-0.73601300	-0.52852200
H	-5.22066900	0.30291500	-0.63309300
H	-5.55186800	-1.35744800	-1.14114600
H	-5.03836400	-1.03132400	0.51879000
C	-3.06994800	-2.41377100	-0.95132500

H	-2.02884600	-2.60006600	-1.24596700
H	-3.22301200	-2.86545900	0.03850800
H	-3.71639300	-2.96329500	-1.65044800
C	-1.48193400	0.94298000	2.67390500
H	-0.47578000	0.53122900	2.49891000
H	-1.46093700	2.00031400	2.37724800
H	-1.65970000	0.91488600	3.75839700
C	-2.64662300	-1.27029400	2.49451900
H	-2.87454200	-1.24010600	3.56953500
H	-3.43182900	-1.86353400	2.00988800
H	-1.69923700	-1.81256000	2.38524700
C	-3.78902600	2.74762200	0.24105900
H	-3.87649200	3.79012900	-0.09933200
H	-4.76333700	2.27167400	0.06588100
H	-3.62783200	2.77479500	1.32608800
C	-2.85588000	2.16221300	-2.01606600
H	-3.80611000	1.70883500	-2.33054000
H	-2.88628500	3.21634600	-2.32804300
H	-2.05494200	1.67615100	-2.59118700

C-IM8a

M06/BSI SCF energy in THF: -1440.583094 a.u.

M06/BSII SCF energy in THF: -1440.837525 a.u.

M06/BSII free energy in THF: -1440.223035 a.u.

C	2.98587900	-1.57295900	1.41132000
C	3.94801300	-1.09933500	0.45121700
C	3.74103200	-1.82885300	-0.75528200
C	2.63446500	-2.71079400	-0.56248300

C	2.19464500	-2.56995800	0.79613300
Ru	1.94906000	-0.66355800	-0.30968200
C	0.22641800	-0.66262600	-1.75271200
H	4.29605400	-1.70373200	-1.67777400
H	4.70750400	-0.34561000	0.62104400
H	2.86881600	-1.20341600	2.42445600
H	1.36716900	-3.09684500	1.25737000
H	2.23441900	-3.39929700	-1.29795900
C	1.03862100	0.53394900	-1.82661700
C	0.16200100	1.22629000	-0.76222100
C	2.39138000	0.59191900	-2.14564700
H	2.98734400	1.48017000	-1.94675400
H	2.78430400	-0.08664000	-2.90146600
C	0.96813200	1.70619800	0.44430500
H	1.67312000	0.90931500	0.83492900
H	0.29144400	1.82525600	1.30233500
C	-0.78970800	2.28137500	-1.32477400
H	-0.19788500	3.02234300	-1.88366600
H	-1.44086400	1.79379800	-2.06701900
C	1.78803000	2.97509200	0.24396300
H	1.10942000	3.84113800	0.26797000
H	2.25043000	2.99273800	-0.75391200
C	2.86535100	3.14637900	1.30377100
H	3.58045400	2.31053600	1.22467400
H	2.40924100	3.06322900	2.30231600
C	3.60125100	4.46802500	1.17661600
H	2.91519400	5.31498700	1.30351800
H	4.39309900	4.56812700	1.92690800
H	4.06631800	4.56851200	0.18714100

C	-1.63814100	2.99729900	-0.27943100
H	-1.97520700	2.28625100	0.49031700
H	-1.03028900	3.73888400	0.26053200
C	-2.85379800	3.69004200	-0.87808100
H	-3.42256500	2.96501400	-1.48267700
H	-2.52306100	4.47124900	-1.57862600
C	-3.75598800	4.28179500	0.18936400
H	-4.62615300	4.78921100	-0.24173500
H	-4.13005700	3.49681700	0.86273900
H	-3.21782300	5.01202100	0.80765800
C	-0.44359800	-0.22614500	-0.59395000
H	0.15690600	-1.55103600	-2.37566000
Si	-1.98032700	-1.04455600	0.21187200
C	-1.97605200	-1.00656400	2.11139800
H	-2.94184000	-1.47594100	2.36856900
C	-3.47361000	-0.04674300	-0.45058500
H	-3.15181500	1.00274700	-0.40068800
C	-2.10659200	-2.82449400	-0.46998800
H	-2.32278200	-2.68440600	-1.54307200
C	-3.30240000	-3.54553500	0.15426000
H	-4.24225800	-2.99261800	0.04067000
H	-3.44814000	-4.53169000	-0.30916000
H	-3.14670000	-3.71826900	1.22868500
C	-0.87381900	-3.71669800	-0.37583300
H	0.01204600	-3.26154400	-0.83150600
H	-0.62713600	-3.96041800	0.66625000
H	-1.05356400	-4.67295100	-0.88814600
C	-1.97185500	0.39944900	2.70666400
H	-0.99601800	0.88566100	2.56569300

H	-2.73597100	1.06031500	2.27580600
H	-2.14889400	0.36348500	3.79095400
C	-0.86958000	-1.84453500	2.74742100
H	-0.92727400	-1.80058700	3.84460500
H	-0.92736200	-2.90234800	2.46185900
H	0.12762700	-1.47560600	2.46199200
C	-4.74881500	-0.15271100	0.38658400
H	-5.52194400	0.51986300	-0.01307200
H	-5.17288400	-1.16546600	0.37356500
H	-4.59272700	0.12357800	1.43673900
C	-3.76943600	-0.36092100	-1.91605900
H	-4.19614100	-1.36696600	-2.03077400
H	-4.50374800	0.34600500	-2.32882300
H	-2.87483600	-0.30918600	-2.55309500

C-TS8a

M06/BSI SCF energy in THF: -1440.555678 a.u.

M06/BSII SCF energy in THF: -1440.809894 a.u.

M06/BSII free energy in THF: -1440.200292 a.u.

C	-1.77408300	-1.09117900	-2.51497800
C	-1.73173000	-2.37874600	-1.96993300
C	-0.35238500	-2.76065800	-1.86378600
C	0.45723500	-1.72865500	-2.46606300
C	-0.42754200	-0.68445400	-2.82370600
Ru	-0.47228300	-1.00732900	-0.59367100
C	0.08607400	-0.11676400	1.73880400
H	0.00937800	-3.70655600	-1.47418900
H	-2.57582400	-2.95646700	-1.61286100

H	-2.66034500	-0.48449900	-2.66258900
H	-0.14889600	0.27635400	-3.24513600
H	1.53127600	-1.75366700	-2.60966200
C	-1.29744100	-0.56506300	1.31627700
C	-2.12975600	0.33198300	0.59614400
C	-1.46429100	-1.96260100	1.09671000
H	-2.43184500	-2.37025800	0.81334200
H	-0.79692100	-2.65956000	1.60199100
C	-3.54020300	-0.03830400	0.22639900
H	-3.60706600	-1.03096300	-0.23332900
H	-3.91827200	0.67733900	-0.51684800
C	-1.83360600	1.80439600	0.56714300
H	-2.70770000	2.31150800	1.00885900
H	-0.96770700	2.03976900	1.20320300
C	-4.45066000	-0.00321300	1.46046000
H	-4.35374900	0.96664600	1.97253900
H	-4.11264100	-0.76618400	2.17868000
C	-5.90991900	-0.24573200	1.10475600
H	-5.99931200	-1.20669600	0.57531400
H	-6.24465800	0.52592100	0.39545100
C	-6.80464500	-0.24487000	2.33128400
H	-6.74853400	0.71491700	2.86072800
H	-7.85432100	-0.41855300	2.06945600
H	-6.50621500	-1.02834300	3.03959600
C	-1.60231300	2.39413600	-0.82444100
H	-0.70099500	1.93793500	-1.26849200
H	-2.43893400	2.13004900	-1.49037900
C	-1.44261700	3.90645200	-0.78838300
H	-0.61118300	4.16685000	-0.11500200

H	-2.34520900	4.35386700	-0.34569900
C	-1.19385800	4.49117600	-2.16682800
H	-1.08100700	5.58032300	-2.13270100
H	-0.27867600	4.07868400	-2.61342000
H	-2.02375400	4.26471300	-2.84885000
C	0.92873200	-0.21610700	0.68655200
H	0.30209300	0.19737200	2.76776000
Si	2.76770900	0.13628800	0.49197600
C	3.59195800	-1.42989500	-0.23193200
H	3.45416500	-1.32959300	-1.32417200
C	5.09528400	-1.48135400	0.04441200
H	5.29996000	-1.63797900	1.11231300
H	5.56452900	-2.31695800	-0.49433700
H	5.62085900	-0.56604800	-0.25551900
C	2.93950100	-2.74249200	0.19982300
H	1.86962600	-2.78374900	-0.04933300
H	3.42611600	-3.60076900	-0.28655700
H	3.02305600	-2.90101200	1.28445400
C	2.85028700	1.51031900	-0.82377800
H	2.35456600	1.03836600	-1.69371500
C	4.27547600	1.87532700	-1.23268200
H	4.82527900	1.02481200	-1.65474900
H	4.27609200	2.67099800	-1.99166100
H	4.85154300	2.25377600	-0.37526300
C	2.06360100	2.77268600	-0.47255200
H	1.08139000	2.55948200	-0.02812000
H	2.61457700	3.39679400	0.24267300
H	1.89672600	3.39341700	-1.36548800
C	3.55074200	0.60169800	2.16561100

H	4.62920600	0.72996200	1.97164000
C	3.39358400	-0.52618100	3.18698600
H	2.33603100	-0.73513900	3.40387400
H	3.84747200	-1.46715400	2.85269400
H	3.86845600	-0.25753800	4.14163700
C	3.02337100	1.91476800	2.74262700
H	3.43453100	2.08926200	3.74758500
H	3.29708100	2.77977400	2.12727500
H	1.92747600	1.91319600	2.84131100

C-IM9a

M06/BSI SCF energy in THF: -1440.583735 a.u.

M06/BSII SCF energy in THF: -1440.837379 a.u.

M06/BSII free energy in THF: -1440.228858 a.u.

C	0.52001500	-2.98726300	-1.10682900
C	-0.90919700	-3.08884400	-1.13215100
C	-1.40282600	-2.19108600	-2.11739500
C	-0.30207700	-1.48264400	-2.66116600
C	0.89412800	-1.98482900	-2.04576600
Ru	-0.31056300	-1.03730500	-0.44042100
C	-0.08849900	0.90579200	0.56336000
H	-2.44696600	-2.02017600	-2.35692200
H	-1.50789400	-3.75425200	-0.52043900
H	1.18698400	-3.57142500	-0.48254300
H	1.90406100	-1.66254900	-2.27443300
H	-0.35380700	-0.70215500	-3.41261300
C	-1.28101400	0.12882500	0.96373700
C	-0.95369800	-1.04667300	1.70051600

C	-2.33342100	0.05159900	-0.01239600
C	1.04049700	0.11705400	0.37995500
H	-0.16995100	1.94017500	0.21461200
H	-0.13754200	-1.00798000	2.41669600
H	-1.72170100	-1.78329700	1.92240900
C	-2.55166500	1.18656200	-0.98432300
H	-3.25801100	0.84991700	-1.75656900
H	-1.61512500	1.42366400	-1.51005500
C	-3.49863300	-0.88327100	0.19410200
H	-3.96347100	-1.08948100	-0.78013300
H	-3.16637800	-1.85133200	0.58752900
C	-4.55793900	-0.31545600	1.14393000
H	-5.02761600	0.57396600	0.69895500
H	-4.07483800	0.02001100	2.07590800
C	-5.63417300	-1.34217400	1.46641800
H	-6.07713500	-1.70836700	0.52767800
H	-5.16546600	-2.21660300	1.94346400
C	-6.71874900	-0.77803100	2.36625300
H	-7.22545600	0.07148500	1.89045300
H	-7.48192000	-1.52803200	2.60184900
H	-6.30000500	-0.42090800	3.31602000
C	-3.07363500	2.48146100	-0.35078600
H	-4.05518400	2.31471600	0.11535700
H	-2.39944100	2.79569400	0.46177900
C	-3.18268000	3.59687700	-1.37990400
H	-2.19306700	3.76450700	-1.83305400
H	-3.84027200	3.26992500	-2.19941500
C	-3.70465700	4.89049400	-0.78167400
H	-3.77486500	5.68553300	-1.53236500

H	-4.70447000	4.75286100	-0.35021300
H	-3.04803900	5.24987300	0.02123000
Si	2.92776100	0.33783500	0.39778400
C	3.40088200	1.10030200	-1.28374000
H	3.47728400	0.22749100	-1.95672200
C	3.61607800	-1.42219000	0.54294700
H	3.05574800	-1.97800900	-0.22867100
C	3.26312100	1.40577600	1.93125700
H	2.68509600	0.90684700	2.73029600
C	2.72495600	2.82742400	1.78240400
H	1.65711600	2.85378200	1.52801100
H	2.84723400	3.38793900	2.71976800
H	3.26700800	3.38144300	1.00388600
C	4.72941200	1.42741700	2.36096500
H	4.85418600	2.02790400	3.27311200
H	5.11852600	0.42460100	2.57590000
H	5.37207400	1.87578500	1.59136500
C	2.35940000	2.05440900	-1.86420900
H	2.67560400	2.41750800	-2.85230500
H	1.37652300	1.57803000	-1.98882500
H	2.22404200	2.94113400	-1.22887600
C	4.77181300	1.77727400	-1.23111900
H	5.08333500	2.09571200	-2.23597800
H	4.74925900	2.67843600	-0.60250800
H	5.55853200	1.12051500	-0.83900800
C	5.10196900	-1.55333400	0.21426900
H	5.73125300	-0.99271200	0.91824300
H	5.41856000	-2.60443200	0.27106800
H	5.33903900	-1.19890300	-0.79698000

C	3.29556700	-2.04212300	1.90110300
H	3.83974000	-1.53403500	2.70936400
H	2.22423700	-1.99225100	2.14284800
H	3.58945100	-3.10093500	1.93180900

C-TS9a

M06/BSI SCF energy in THF: -1440.5673319 a.u.

M06/BSII SCF energy in THF: -1440.821247 a.u.

M06/BSII free energy in THF: -1440.210456 a.u.

C	0.56306700	-2.83626000	-1.35985600
C	-0.86907000	-2.93354100	-1.32639100
C	-1.38842200	-2.02236700	-2.29308600
C	-0.30205500	-1.32244300	-2.87618400
C	0.91157600	-1.83684600	-2.30653000
Ru	-0.26909500	-0.90921200	-0.68738400
C	-0.06573400	1.12915800	0.09788000
H	-2.43813600	-1.83829800	-2.49541900
H	-1.44901000	-3.61067300	-0.70983200
H	1.25336400	-3.41093800	-0.75108500
H	1.91487800	-1.50762800	-2.55410300
H	-0.37366000	-0.53978500	-3.62310500
C	-1.13219000	0.36200600	0.73864900
C	-0.33017100	-0.53754400	1.57053300
C	-2.30871400	-0.02116400	0.05175800
C	1.04139500	0.27414600	0.26159200
H	-0.11203600	2.09870700	-0.39311800
H	0.33614600	-0.05718200	2.29015700
H	-0.67275200	-1.51772700	1.90891600

C	-2.89776500	0.91752200	-0.97648200
H	-3.71660500	0.39767500	-1.49571600
H	-2.14508000	1.15689800	-1.74111500
C	-3.21810000	-1.07143100	0.62887100
H	-3.64378500	-1.66165800	-0.19641800
H	-2.65055800	-1.77006900	1.25807300
C	-4.36541900	-0.49541900	1.46205700
H	-5.02607400	0.11284000	0.82636100
H	-3.95846400	0.18428900	2.22824700
C	-5.18196800	-1.59209100	2.13034900
H	-5.56495600	-2.27823600	1.35956400
H	-4.52098800	-2.19454000	2.77185200
C	-6.33399800	-1.03827200	2.94948700
H	-7.02777000	-0.46491700	2.32130400
H	-6.90781500	-1.83537600	3.43512100
H	-5.97082800	-0.36397600	3.73586100
C	-3.42098000	2.23676300	-0.39971800
H	-4.16046100	2.04574300	0.39158800
H	-2.59292800	2.78121900	0.08139600
C	-4.04430700	3.11182300	-1.47684100
H	-3.30351100	3.28618000	-2.27237700
H	-4.87482000	2.56671500	-1.95058400
C	-4.54111300	4.43857200	-0.93164100
H	-4.98285100	5.06148900	-1.71739500
H	-5.30657100	4.28893200	-0.15926000
H	-3.72325500	5.01103000	-0.47518100
Si	2.93464200	0.38177900	0.44775300
C	3.56337900	1.22532000	-1.14176700
H	3.69330100	0.38928000	-1.85124600

C	3.54066500	-1.41062600	0.51875800
H	3.03551200	-1.88652200	-0.34021900
C	3.18955100	1.33415500	2.07207200
H	2.54457900	0.79833500	2.79159000
C	2.69341100	2.77652500	1.98531500
H	1.65638300	2.84962900	1.63060800
H	2.73379200	3.26028800	2.97125100
H	3.31876000	3.37864100	1.31159600
C	4.61865200	1.28120200	2.61145100
H	4.68979400	1.82310500	3.56507300
H	4.95758600	0.25380500	2.79442000
H	5.33323300	1.74971100	1.92208800
C	2.59430700	2.22313700	-1.77174700
H	3.02944000	2.65837400	-2.68249700
H	1.64253400	1.75586800	-2.05824600
H	2.36978900	3.06022800	-1.09585700
C	4.93070500	1.87573500	-0.92484600
H	5.33599500	2.24467200	-1.87744700
H	4.85556200	2.73967700	-0.25127700
H	5.67086900	1.18595400	-0.49980400
C	5.04571300	-1.57417000	0.31126700
H	5.62499100	-1.10517200	1.11718600
H	5.31910900	-2.63869700	0.30194500
H	5.38988000	-1.14303200	-0.63730800
C	3.08421000	-2.11653000	1.79394900
H	3.55329300	-1.67259600	2.68376300
H	1.99525800	-2.06653500	1.93436600
H	3.36431300	-3.17946400	1.77923500

C-IM10a

M06/BSI SCF energy in THF: -1440.592704 a.u.

M06/BSII SCF energy in THF: -1440.847042 a.u.

M06/BSII free energy in THF: -1440.235989 a.u.

C	0.64986900	-2.71528200	-1.70099600
C	-0.76804000	-2.96527900	-1.62289000
C	-1.42830900	-1.97372900	-2.38907200
C	-0.44255300	-1.07681400	-2.90894200
C	0.84701800	-1.56661500	-2.51260500
Ru	-0.30945100	-0.99685000	-0.75202600
C	-0.02865600	1.13192300	-0.14400600
H	-2.50188400	-1.86297500	-2.49791600
H	-1.24550200	-3.76884500	-1.07413600
H	1.42526600	-3.30254700	-1.22285400
H	1.79884800	-1.10146500	-2.74612800
H	-0.63371800	-0.20715900	-3.52746600
C	-1.08865300	0.44712900	0.58136200
C	-0.03886000	-0.28303400	1.41261500
C	-2.31608700	-0.02160300	0.10639100
C	0.99234100	0.37978700	0.48748500
H	-0.02914900	1.93180900	-0.87878800
H	0.02269700	-0.11275000	2.49339600
H	-0.09953600	-1.41896300	1.32484800
C	-3.04839300	0.77593900	-0.94812700
H	-3.90769600	0.18856900	-1.30535000
H	-2.39861500	0.94276500	-1.81842600
C	-3.10116900	-1.03865600	0.89026300
H	-3.50656700	-1.78460600	0.18800800

H	-2.43638200	-1.58074900	1.57757700
C	-4.26420000	-0.45387300	1.69371800
H	-4.98895500	0.02545100	1.01850600
H	-3.88918900	0.33674800	2.36315000
C	-4.97676800	-1.52316700	2.50887500
H	-5.32295800	-2.31983600	1.83262900
H	-4.25691000	-1.99647300	3.19371200
C	-6.15077800	-0.96728900	3.29497700
H	-6.89624900	-0.51635300	2.62723300
H	-6.65626400	-1.74691700	3.87572300
H	-5.82481300	-0.18892900	3.99693300
C	-3.53266100	2.14045200	-0.44864400
H	-4.13848400	2.02358200	0.46198600
H	-2.66115400	2.74788100	-0.15805100
C	-4.34113500	2.88243900	-1.50158700
H	-3.73895700	2.98288900	-2.41783400
H	-5.21710600	2.27642700	-1.77866500
C	-4.78937500	4.25238500	-1.02402100
H	-5.37452800	4.77859000	-1.78639400
H	-5.41179700	4.17331700	-0.12340900
H	-3.92812500	4.88485100	-0.77224200
Si	2.88989900	0.40417200	0.55346300
C	3.57248000	0.74952700	-1.19156300
H	3.59914900	-0.24711600	-1.66967900
C	3.39398600	-1.34361400	1.11205000
H	2.77114500	-2.00689900	0.48356100
C	3.29439300	1.73128300	1.85801000
H	2.65744200	1.45716300	2.71850600
C	2.88437500	3.12945500	1.39806700

H	1.83051700	3.18510000	1.09438500
H	3.02871300	3.86604900	2.20118400
H	3.49308900	3.46261700	0.54596200
C	4.74577400	1.72868300	2.33603600
H	4.89317100	2.47325200	3.13144100
H	5.05584300	0.75679100	2.73931900
H	5.43915000	1.98508000	1.52472700
C	2.69071900	1.65204100	-2.05292400
H	3.14724800	1.80810700	-3.04121100
H	1.69383100	1.22331800	-2.21953500
H	2.56008700	2.64618000	-1.60236000
C	5.00261500	1.29171900	-1.16642000
H	5.41107100	1.35597800	-2.18492200
H	5.03255600	2.30558000	-0.74479600
H	5.68840600	0.66926700	-0.57831600
C	4.85603400	-1.69249100	0.83537700
H	5.54020200	-1.07060600	1.42908600
H	5.06320200	-2.73802200	1.10482700
H	5.12790300	-1.56994100	-0.22058700
C	3.05511100	-1.61594800	2.57613600
H	3.66982800	-0.99922300	3.24552600
H	2.00493000	-1.41113500	2.82197400
H	3.25306100	-2.66505500	2.83814200

C-IM11a

M06/BSI SCF energy in THF: -1573.258455 a.u.

M06/BSII SCF energy in THF: -1573.5479572 a.u.

M06/BSII free energy in THF: -1572.893964 a.u.

C	0.64393200	-2.27964500	-2.10391600
C	-0.08770600	-1.14824300	-2.53689000
C	-1.47428200	-1.42998400	-2.32676700
C	-1.61036400	-2.76322100	-1.83367800
C	-0.31242700	-3.27657000	-1.66272300
Ru	-0.38383200	-1.45350600	-0.34068400
C	-0.19833100	0.67179200	1.27575300
H	-2.29055200	-0.76971100	-2.59597700
H	0.31620500	-0.22875900	-2.94602800
H	1.72028200	-2.40485700	-2.13923600
H	-0.06062200	-4.25327400	-1.26421300
H	-2.53989300	-3.26536700	-1.59257700
C	-1.45248000	-0.10939000	1.03647400
C	-2.27826100	0.27489200	-0.04975800
C	1.59868000	-3.10454300	1.58186300
N	0.89058800	-2.49204000	0.90338700
C	2.50825500	-3.85525100	2.41262700
H	3.40920500	-4.10079200	1.84092400
H	2.03459900	-4.78201200	2.75000500
H	2.79097200	-3.25997500	3.28632200
C	-1.56713800	-1.43652500	1.53259500
H	-2.49725600	-1.99090000	1.42161300
H	-0.96698700	-1.71268000	2.39674500
C	-3.64009100	-0.34103200	-0.23321900
H	-3.58168500	-1.43621700	-0.26382000
H	-4.05312200	-0.01786400	-1.19907500
C	-2.02508100	1.55605900	-0.77919500
H	-0.95031600	1.76085000	-0.80022300
H	-2.37383300	1.46353600	-1.81808200

C	-4.61979500	0.05103800	0.88151400
H	-4.69668700	1.14536100	0.95504100
H	-4.23086000	-0.29656900	1.85031600
C	-6.00146100	-0.54096300	0.64496600
H	-5.91436500	-1.63459500	0.55616300
H	-6.38750500	-0.18674000	-0.32268500
C	-6.97769100	-0.18672300	1.75265400
H	-7.10688000	0.90011500	1.83467300
H	-7.96673800	-0.62509000	1.57851700
H	-6.62131700	-0.54921100	2.72555800
C	-2.70985500	2.76371000	-0.12353700
H	-3.79850400	2.71671700	-0.27536000
H	-2.54468000	2.73571400	0.96568000
C	-2.16526600	4.07029700	-0.68214200
H	-2.26604000	4.06642600	-1.77829800
H	-1.08378900	4.11808500	-0.47584800
C	-2.86114100	5.28556200	-0.09701400
H	-2.45200000	6.22055900	-0.49582000
H	-3.93595900	5.27257400	-0.31901900
H	-2.75189900	5.31501400	0.99481800
C	0.73971100	0.16502400	0.46542300
H	-0.16729800	1.56278800	1.91071500
C	2.50118100	2.48130000	1.21345400
Si	2.48310000	0.86213800	0.19468900
C	2.68991300	1.18811300	-1.67431300
H	2.42735500	0.22473900	-2.14697800
C	3.80053400	-0.37949500	0.81005900
H	3.39173700	-0.77605600	1.75670500
C	3.58143800	3.48919300	0.82399300

H	3.51689400	4.38842700	1.45463500
H	4.59246000	3.08018600	0.95610100
H	3.49232000	3.82083900	-0.21770800
C	4.11049900	1.54071400	-2.11590100
H	4.46033500	2.47872400	-1.66516600
H	4.83828500	0.76222500	-1.85874000
H	4.15299100	1.67724200	-3.20646000
C	1.71031700	2.24283900	-2.18578500
H	1.80040700	2.37290200	-3.27439900
H	0.66378800	1.98683600	-1.97479600
H	1.89959200	3.22561600	-1.73040700
C	5.15365400	0.25906900	1.12800500
H	5.61278200	0.70833800	0.23729000
H	5.08472600	1.04220700	1.89162100
H	5.85974600	-0.49741500	1.50099200
C	4.00374000	-1.54553600	-0.15609400
H	4.44322300	-1.20384500	-1.10364700
H	4.69940500	-2.28917800	0.26147100
H	3.06876100	-2.06165700	-0.39981900
C	2.54522200	2.22303600	2.72159700
H	3.53343700	1.86242100	3.03771800
H	2.35440500	3.14898300	3.28330400
H	1.80904300	1.48036900	3.05484200
H	1.52268900	2.94044900	0.98307400

C-TS10a

M06/BSI SCF energy in THF: -1573.236708 a.u.

M06/BSII SCF energy in THF: -1573.525566 a.u.

M06/BSII free energy in THF: -1572.872307 a.u.

C	-0.85152700	-2.08678400	2.17265000
C	0.06224400	-1.09771300	2.60474200
C	1.38295400	-1.60391400	2.37181000
C	1.27878200	-2.94658500	1.89650700
C	-0.08764600	-3.23616100	1.73945400
Ru	0.35388300	-1.41514100	0.42526700
C	0.03241400	0.02530100	-1.57331400
H	2.30510800	-1.08093900	2.60024100
H	-0.17706200	-0.11754400	3.00224300
H	-1.93347500	-2.01695000	2.19802000
H	-0.50322400	-4.15341000	1.33962600
H	2.10768300	-3.59350800	1.63328000
C	1.36676700	-0.55295200	-1.19558400
C	2.08076100	0.07388600	-0.12225600
C	-2.10422400	-3.40310700	-1.71435900
N	-1.36325700	-2.87970300	-0.99089400
C	-3.04650700	-4.03930500	-2.60623900
H	-3.60496300	-4.81244600	-2.07031300
H	-2.51824900	-4.50140700	-3.44522900
H	-3.75028200	-3.29642300	-2.99441800
C	1.57792700	-1.95556400	-1.33052700
H	2.52163200	-2.41725400	-1.05175900
H	0.98957800	-2.50580200	-2.06181100
C	3.46861500	-0.43109300	0.22585000
H	3.46178100	-1.48570700	0.52669100
H	3.82298300	0.12723200	1.10458300
C	1.82693300	1.47967900	0.35711800
H	0.81245200	1.80357900	0.10853500

H	1.88797700	1.46959600	1.45819600
C	4.48365300	-0.26159200	-0.91090500
H	4.38560700	0.73204600	-1.37155500
H	4.26603300	-0.98591500	-1.71021000
C	5.91132800	-0.44904600	-0.42107300
H	6.00309700	-1.43036300	0.06988600
H	6.12146800	0.30042400	0.35794000
C	6.92991800	-0.33012800	-1.54046500
H	6.86151000	0.64852400	-2.03288600
H	7.95521900	-0.44468500	-1.17133200
H	6.76704100	-1.09588700	-2.30991100
C	2.79942000	2.53992300	-0.16006300
H	3.83474700	2.30548500	0.13164500
H	2.77900200	2.55593900	-1.26182000
C	2.42836100	3.91539500	0.37769000
H	2.44057500	3.88458000	1.47846000
H	1.38890700	4.14594100	0.09532500
C	3.35742700	5.00639300	-0.12252500
H	3.08027900	5.99036600	0.27188000
H	4.39550600	4.80940400	0.17496300
H	3.33755600	5.07077600	-1.21807600
C	-0.72569200	0.12125100	-0.46096600
H	-0.22827900	0.25444900	-2.61231500
C	-2.11354600	2.72516700	-1.11214800
Si	-2.32823300	1.09598600	-0.13252800
C	-2.44986800	1.38795100	1.74533400
H	-2.29650200	0.38630000	2.18367700
C	-3.78638800	0.05588300	-0.79270800
H	-3.42827800	-0.32661400	-1.76603500

C	-3.03618300	3.86415600	-0.67644300
H	-2.84048300	4.76529000	-1.27577200
H	-4.09638600	3.61360300	-0.81937300
H	-2.90052200	4.14167900	0.37534300
C	-3.80718600	1.89730100	2.22897400
H	-4.04791000	2.88307300	1.81045600
H	-4.63088600	1.22082900	1.97140100
H	-3.80854200	2.00867300	3.32301800
C	-1.33361000	2.29630800	2.26217500
H	-1.36898100	2.37656100	3.35879000
H	-0.33351700	1.93502800	1.98967200
H	-1.42664700	3.31763400	1.86688900
C	-5.06596800	0.85384000	-1.04496900
H	-5.46336400	1.29181100	-0.11938900
H	-4.92270300	1.67274400	-1.75979100
H	-5.85268100	0.20156300	-1.45154400
C	-4.08264400	-1.14231000	0.10751500
H	-4.49235100	-0.81880400	1.07444200
H	-4.83310800	-1.80575800	-0.34843700
H	-3.18786000	-1.74359700	0.30663000
C	-2.23375700	2.51737800	-2.62213500
H	-3.25411000	2.22847600	-2.90742900
H	-2.00300200	3.44651800	-3.16244300
H	-1.55621200	1.74632900	-3.00824900
H	-1.07549400	3.03753300	-0.90066900

C-TS11a

M06/BSI SCF energy in THF: -1573.198854 a.u.

M06/BSII SCF energy in THF: -1573.487395 a.u.

M06/BSII free energy in THF: -1572.833638 a.u.

C	-0.49229800	-1.29988100	2.34726800
C	0.93193800	-1.19989600	2.24039900
C	1.43040600	-2.49895200	1.91973000
C	0.32095800	-3.37666000	1.76788800
C	-0.87488200	-2.62030400	2.04111500
Ru	0.17938200	-1.82152700	0.23428500
C	-0.01284500	-0.34433400	-1.48103000
H	2.47108500	-2.77322200	1.78612900
H	1.51313700	-0.30565300	2.43451500
H	-1.16651400	-0.48425400	2.58470200
H	-1.88985900	-2.99962600	1.99681800
H	0.36141900	-4.43018200	1.51706100
C	1.37255700	-0.71387100	-1.20155100
C	2.06113400	0.38911800	-0.55032900
C	-1.72643600	-3.60748700	-1.66446600
N	-1.01442800	-2.91714700	-1.06580100
C	-2.64546200	-4.43175500	-2.41293600
H	-3.38705800	-4.86765800	-1.73635500
H	-2.10880800	-5.23936600	-2.91949600
H	-3.16256300	-3.82272200	-3.16160900
C	1.77483100	-2.07733700	-1.26412700
H	2.75476800	-2.37292700	-0.88965800
H	1.38862000	-2.68357000	-2.08284000
C	3.24319200	0.08579000	0.30885800
H	3.15456400	-0.90851500	0.75704000
H	3.30080600	0.80758400	1.13602700
C	2.04461100	1.76312300	-1.13972600

H	2.90531400	1.80866300	-1.83567600
H	1.15204100	1.91714400	-1.75274700
C	4.56415600	0.13983000	-0.48269900
H	4.80822400	1.17997200	-0.74175700
H	4.44694500	-0.39914500	-1.43614900
C	5.70950500	-0.47139800	0.30954000
H	5.47625400	-1.52610300	0.52423700
H	5.78241300	0.03012000	1.28642000
C	7.03297600	-0.37210100	-0.42767200
H	7.30000500	0.67439200	-0.62275700
H	7.85160000	-0.82304800	0.14401100
H	6.98440400	-0.88433400	-1.39731600
C	2.19472600	2.90286800	-0.13612700
H	1.46665600	2.76148700	0.68059900
H	3.19049600	2.87618500	0.33091800
C	1.98887300	4.26337500	-0.78379900
H	0.98516600	4.30068500	-1.23744400
H	2.70100900	4.37974200	-1.61490200
C	2.15196000	5.40293700	0.20571000
H	2.00377300	6.37979600	-0.26800800
H	1.42708300	5.31880400	1.02636300
H	3.15489900	5.39975500	0.65194200
C	-0.46362100	0.33621800	-0.38449500
H	-0.56147000	-0.64325400	-2.38082500
Si	-2.12260800	1.14550700	0.05260300
C	-2.69174200	2.17239000	-1.45963100
H	-2.98992400	1.39546300	-2.18343200
C	-1.75667500	2.18775500	1.61900700
H	-0.90859600	1.65552700	2.08606200

C	-3.39296700	-0.28411600	0.27516300
H	-2.84143300	-1.05890600	0.83181500
C	-4.68159000	0.00533400	1.04562100
H	-5.33376800	-0.88092200	1.03245000
H	-4.50858600	0.26081300	2.09541100
H	-5.25952900	0.82450800	0.59737100
C	-3.75951300	-0.88019200	-1.08809800
H	-2.88975400	-1.05701500	-1.73461700
H	-4.27688600	-1.84347400	-0.96193700
H	-4.44874300	-0.22167600	-1.63434100
C	-3.93557800	3.00378800	-1.13971400
H	-4.75468300	2.40607900	-0.72017700
H	-3.71251300	3.80366600	-0.42032400
H	-4.32306000	3.49065500	-2.04647700
C	-1.64164200	3.03636000	-2.15167700
H	-2.05272300	3.47634800	-3.07222100
H	-1.31148300	3.87256700	-1.52174700
H	-0.75103500	2.46304000	-2.44078100
C	-2.86271800	2.30093900	2.66443300
H	-3.79370000	2.70810100	2.24569500
H	-3.10377700	1.33615200	3.12663200
H	-2.55380500	2.97750300	3.47461800
C	-1.25056700	3.58449900	1.25349200
H	-0.47691600	3.56733200	0.47541200
H	-2.06664200	4.22421900	0.89092200
H	-0.82188200	4.08315700	2.13518600

A-IM11b

M06/BSI SCF energy in THF: -1573.217842 a.u.

M06/BSII SCF energy in THF: -1573.507732 a.u.

M06/BSII free energy in THF: -1572.861043 a.u.

C	-1.44101600	-3.02376000	-0.33387200
C	-0.20648400	-3.32121200	0.30005200
C	0.81092400	-3.38646400	-0.69981100
C	0.21978300	-3.08743500	-1.95734600
C	-1.18117700	-2.83922600	-1.71848900
Ru	0.02616000	-1.28780500	-0.72684300
C	0.26119800	0.46575500	0.83307400
H	1.85954000	-3.59433100	-0.51915300
H	-0.05611200	-3.47313800	1.36394000
H	-2.39797200	-2.89773600	0.15968600
H	-1.91162400	-2.58701100	-2.47969900
H	0.71474200	-3.07165000	-2.92135400
C	1.94243600	-0.66286600	-0.82256100
C	1.79207400	0.28233200	0.31930600
C	-0.34289700	0.79750300	-3.14468800
N	-0.18730100	0.08678200	-2.24577200
C	-0.55989600	1.69302800	-4.25472900
H	-0.02942200	2.63398600	-4.07959100
H	-1.62978700	1.89984200	-4.36062500
H	-0.18848400	1.24167400	-5.17939000
C	2.94063900	-0.96921900	-1.64182300
H	3.93756500	-0.53992300	-1.53441500
H	2.81426200	-1.68444500	-2.45433200
C	2.13653700	1.74232400	-0.06883200
H	1.75421800	2.38095000	0.74525200
H	1.54756000	1.99987800	-0.96282100

C	2.52150500	-0.20931100	1.59486800
H	2.54042900	0.64528500	2.29279300
H	1.89547900	-0.97665700	2.07683800
C	3.59434800	2.10570200	-0.30807000
H	4.04863300	1.44342100	-1.05866500
H	4.17479800	1.98057700	0.61856100
C	3.72875800	3.54721400	-0.78417900
H	3.25070400	4.21958400	-0.05551200
H	3.16778300	3.66891200	-1.72359400
C	5.17695100	3.95449000	-0.98939100
H	5.66891100	3.30724100	-1.72705000
H	5.26427100	4.98731300	-1.34514300
H	5.74518200	3.87617800	-0.05353600
C	3.92451800	-0.77903000	1.44411000
H	3.87795400	-1.74281500	0.91424000
H	4.55736200	-0.12549000	0.82701900
C	4.59094500	-0.97842300	2.79888000
H	3.94456900	-1.60162700	3.43632700
H	4.66967600	-0.00534500	3.30774900
C	5.96601800	-1.61063800	2.68074300
H	6.44385200	-1.73508800	3.65909100
H	5.90750700	-2.60153900	2.21185400
H	6.63177600	-0.99414000	2.06262000
C	-0.94360400	0.15043200	0.36425800
H	0.34799700	0.92734500	1.82593600
Si	-2.68546200	0.74321300	0.78318200
C	-2.58368900	2.27765800	1.90356800
H	-3.62775900	2.63391600	1.95310200
C	-3.66225900	-0.67823300	1.57971400

H	-3.86973100	-1.38154300	0.75548400
C	-3.42289900	1.25975100	-0.89696100
H	-2.59584400	1.78178800	-1.40962900
C	-5.00670600	-0.19451700	2.12418300
H	-5.57477800	-1.02751200	2.56202000
H	-4.87472400	0.55377100	2.91873200
H	-5.63929400	0.25670900	1.34865900
C	-2.88899900	-1.43129700	2.66115000
H	-1.89902000	-1.76517000	2.32188700
H	-2.73126100	-0.80810700	3.55106000
H	-3.44362200	-2.32168400	2.99033200
C	-1.74271700	3.38600400	1.27333600
H	-1.78736800	4.30487800	1.87488100
H	-0.68532900	3.09477600	1.20738400
H	-2.07372000	3.64503500	0.25863900
C	-2.12980500	1.98902700	3.33175400
H	-2.07198200	2.91701600	3.91864900
H	-2.81939200	1.31463700	3.85368000
H	-1.13250100	1.52744200	3.36620900
C	-4.58863100	2.23959400	-0.77811000
H	-4.94983200	2.53847500	-1.77267900
H	-5.44296900	1.79226000	-0.25157700
H	-4.31664800	3.15684200	-0.24105700
C	-3.81767800	0.05437500	-1.74455000
H	-4.09362500	0.35702900	-2.76539700
H	-3.00473000	-0.67986200	-1.82506900
H	-4.68975100	-0.46231600	-1.31888600

A-TS10b

M06/BSI SCF energy in THF: -1573.196091 a.u.

M06/BSII SCF energy in THF: -1573.486464 a.u.

M06/BSII free energy in THF: -1572.835531 a.u.

C	-1.46362400	-2.23429100	-1.92691700
C	-0.25311600	-2.94332200	-1.64351600
C	0.75439400	-2.46675200	-2.53640500
C	0.20207900	-1.40385600	-3.30170100
C	-1.18165000	-1.27218500	-2.92526300
Ru	0.12027900	-0.82467900	-1.19146800
C	0.35064500	0.05130400	0.89662100
H	1.77986600	-2.81381500	-2.56477700
H	-0.13163600	-3.73274900	-0.91047800
H	-2.40872500	-2.36715700	-1.41202900
H	-1.87306500	-0.54446800	-3.33544200
H	0.71126100	-0.80958700	-4.05153900
C	2.05907100	-0.35425200	-1.11194000
C	1.90194500	0.10740000	0.29361900
C	-1.27663900	2.71544100	-2.39657000
N	-0.46168700	1.93382200	-2.12320000
C	-2.30133200	3.68276100	-2.72236800
H	-3.02405600	3.74467100	-1.90094700
H	-2.82517300	3.38440400	-3.63541000
H	-1.85403600	4.66935100	-2.87531600
C	3.07071600	-0.34785200	-1.97325500
H	4.04831900	0.05594200	-1.70786100
H	2.98039300	-0.75024400	-2.98095500
C	2.19002600	1.62203400	0.44429000
H	1.78185800	1.93681900	1.41929700

H	1.61216800	2.15594400	-0.32521500
C	2.65479500	-0.78735100	1.30780400
H	2.68500000	-0.22287800	2.25569400
H	2.04197900	-1.67959000	1.50630200
C	3.64365800	2.06784800	0.38685100
H	4.11692900	1.74690400	-0.55173700
H	4.21672900	1.60739700	1.20630400
C	3.75337600	3.58336300	0.49786000
H	3.24686900	3.91825000	1.41584500
H	3.20629500	4.04648000	-0.33713100
C	5.19515100	4.05960900	0.50059000
H	5.71610700	3.75179100	-0.41545200
H	5.26457700	5.15098300	0.56886300
H	5.74940000	3.63872400	1.34954900
C	4.05677100	-1.25801100	0.94865000
H	4.00375900	-1.98011400	0.12045700
H	4.68067400	-0.42735800	0.58922900
C	4.73550100	-1.91209000	2.14510500
H	4.07588900	-2.69355100	2.55294300
H	4.84949100	-1.16524100	2.94589100
C	6.08732100	-2.50736400	1.79544900
H	6.57162000	-2.96032800	2.66791000
H	5.99095500	-3.28701200	1.02856000
H	6.76784600	-1.74222400	1.39956900
C	-0.87323000	-0.15249700	0.40408900
H	0.51309700	0.23432100	1.96584600
Si	-2.58680000	0.06042100	1.17674600
C	-2.40533200	0.91197900	2.86820800
H	-3.44784600	1.11459400	3.17106400

C	-3.40282700	-1.65125400	1.29287000
H	-3.72780000	-1.89006300	0.26590000
C	-3.54138700	1.23218700	0.01506900
H	-2.79296000	1.98734200	-0.28371500
C	-4.65322800	-1.61381600	2.17165300
H	-5.15721800	-2.59036900	2.18056200
H	-4.40814900	-1.36982200	3.21469800
H	-5.38581500	-0.87329100	1.82420200
C	-2.44232000	-2.74828400	1.74859300
H	-1.56943500	-2.83744800	1.08733000
H	-2.06208900	-2.56283900	2.76263500
H	-2.94391400	-3.72634100	1.76876600
C	-1.68742300	2.25708900	2.75541100
H	-1.65604600	2.76680000	3.72873000
H	-0.64703400	2.13647900	2.42199100
H	-2.17303400	2.94073800	2.04698400
C	-1.77150300	0.04555900	3.95321000
H	-1.70281100	0.59774700	4.90143300
H	-2.34996800	-0.86603500	4.14846400
H	-0.74975400	-0.26485400	3.69180500
C	-4.70098200	1.96044500	0.69295200
H	-5.18612800	2.65362200	-0.01000400
H	-5.47700300	1.25990300	1.03245600
H	-4.38651000	2.54789600	1.56376900
C	-4.03357900	0.52309700	-1.24276400
H	-4.48999900	1.23121600	-1.95009600
H	-3.22555100	0.00292000	-1.77199200
H	-4.80425100	-0.22227400	-0.99957300

A-TS11b

M06/BSI SCF energy in THF: -1573.204349 a.u.

M06/BSII SCF energy in THF: -1573.493609 a.u.

M06/BSII free energy in THF: -1572.83971 a.u.

C	0.94504300	-3.34658300	-0.68729700
C	-0.33799000	-2.87049200	-1.10835400
C	-1.17172900	-2.85968500	0.04317800
C	-0.40959900	-3.29889000	1.17264800
C	0.90792600	-3.62113100	0.70724600
Ru	0.48580600	-1.48786200	0.39532900
C	-0.51674600	1.00952900	-1.18848400
H	-2.20598000	-2.54186500	0.08432600
H	-0.61687400	-2.59812900	-2.11990400
H	1.82105900	-3.43490800	-1.32204400
H	1.73356800	-3.97831900	1.31186700
H	-0.77496700	-3.38516500	2.18980300
C	-1.11750200	0.07255500	0.68469800
C	-1.82290000	0.74624900	-0.50959200
C	1.90977000	-0.45781100	3.11687300
N	1.41128500	-0.78369100	2.12323100
C	2.55096500	-0.01218600	4.32951900
H	2.50276200	-0.79119300	5.09623000
H	2.05201800	0.88937000	4.69944900
H	3.60104800	0.22150500	4.12313200
C	-1.44965100	0.24951200	1.96849700
H	-2.38391100	0.75661000	2.21928900
H	-0.85195600	-0.09276400	2.80784900
C	-2.47182100	2.10322600	-0.15693900

H	-2.45366900	2.70800800	-1.07863800
H	-1.79800600	2.61382500	0.54965200
C	-2.71523900	-0.14867800	-1.39076100
H	-3.06441400	0.46524100	-2.23654900
H	-2.07847800	-0.92662700	-1.83660600
C	-3.89327700	2.15508300	0.39542300
H	-4.05723600	1.39389700	1.17357500
H	-4.61453200	1.93056000	-0.40446400
C	-4.21154600	3.52911400	0.96937000
H	-4.00362500	4.29671300	0.20777900
H	-3.52319100	3.73672600	1.80345800
C	-5.64959300	3.64463400	1.44189400
H	-5.87157000	2.90073200	2.21831000
H	-5.86421300	4.63414700	1.86110100
H	-6.35242400	3.47473000	0.61593700
C	-3.90652600	-0.83159300	-0.72321600
H	-3.62173000	-1.19646100	0.27812500
H	-4.72121800	-0.11861300	-0.54731900
C	-4.44802400	-1.98673300	-1.55666900
H	-3.63686300	-2.70187700	-1.76922800
H	-4.77104600	-1.60262300	-2.53625100
C	-5.59627900	-2.70510200	-0.87137900
H	-5.98587900	-3.52866400	-1.48003000
H	-5.27574000	-3.12637100	0.09127600
H	-6.42898800	-2.01969900	-0.66759900
C	0.43187800	0.37760900	-0.46775900
H	-0.37173400	1.68305100	-2.03578400
Si	2.25655400	1.09060700	-0.70297900
C	2.18772400	2.88885100	-0.06623200

H	3.17344900	3.30001100	-0.34639200
C	2.57816400	0.89566800	-2.58559400
H	3.65908100	0.67015900	-2.59135700
C	3.73695700	0.27886600	0.18150700
H	3.54205700	0.31740000	1.26221600
C	2.37717500	2.15970100	-3.41663700
H	2.73785900	2.00375500	-4.44331700
H	1.31555500	2.43354700	-3.49094900
H	2.91854700	3.02260700	-3.00886300
C	1.87631700	-0.29014000	-3.24327600
H	1.99451600	-1.22227800	-2.67575900
H	0.79810800	-0.11057900	-3.35349900
H	2.28211300	-0.46303000	-4.25003600
C	2.07239600	2.90553400	1.45935300
H	2.07798100	3.93757200	1.83701200
H	1.12245200	2.45148000	1.78223700
H	2.89048000	2.37343500	1.96183300
C	1.11846100	3.79766700	-0.65943300
H	1.22233400	4.81564400	-0.25813100
H	1.16677100	3.86954000	-1.75131400
H	0.10990300	3.45501500	-0.38981000
C	5.00000400	1.10740100	-0.07704200
H	5.87173900	0.62227800	0.38392100
H	5.22609500	1.20709800	-1.14835600
H	4.93512400	2.11895000	0.34181400
C	3.96601700	-1.17608000	-0.20520000
H	4.84081300	-1.59087300	0.31593900
H	3.09551400	-1.79645300	0.05626400
H	4.14885200	-1.29040100	-1.28361700

D-IM11a

M06/BSI SCF energy in THF: -1573.2235794 a.u.

M06/BSII SCF energy in THF: -1573.513358 a.u.

M06/BSII free energy in THF: -1572.862397 a.u.

C	-1.16589300	-0.79669300	-2.78014500
C	0.03873300	-1.55578900	-2.80895900
C	1.13568200	-0.65058100	-2.87839100
C	0.62075500	0.67095400	-2.82227300
C	-0.82053700	0.58130200	-2.77032600
Ru	0.03005700	-0.25876000	-0.93415400
C	-0.77669000	-1.50270700	0.98687300
H	2.18360000	-0.92568900	-2.89659700
H	0.10953200	-2.63748300	-2.78454600
H	-2.16762500	-1.20629100	-2.73406800
H	-1.50748600	1.41987200	-2.76620100
H	1.19504800	1.59037700	-2.84964800
C	1.69263000	-0.79664300	0.03986300
C	0.80924100	-1.73186400	0.81707100
C	0.37000700	2.39074300	0.84952900
N	0.21939800	1.42551700	0.23021500
C	0.54284000	3.59964300	1.61852600
H	-0.43200900	3.97949000	1.94134100
H	1.03717600	4.36226200	1.00893900
H	1.15503300	3.39501900	2.50230400
C	2.98057000	-0.48914600	0.17844300
C	-1.56618900	-0.67710900	0.31804400
C	3.85795600	-1.22118400	1.17053100

H	4.10187400	-0.54471400	2.00749600
H	3.30680900	-2.06295000	1.61147300
C	3.60781600	0.65088600	-0.57843200
H	2.87531900	1.06011900	-1.28628200
H	4.46025400	0.30095000	-1.18236200
C	4.08089100	1.77782300	0.34125900
H	3.29981100	1.98573800	1.09276700
H	4.96921800	1.46163100	0.90954600
C	4.39511800	3.05714800	-0.41892400
H	5.12153400	2.83973000	-1.21681900
H	3.48015700	3.40163700	-0.92834700
C	5.16355800	-1.75025900	0.58355600
H	4.94799200	-2.36859700	-0.30303200
H	5.78717800	-0.91515700	0.22843700
C	5.95832900	-2.56678600	1.59236600
H	6.12970800	-1.95842900	2.49377700
H	5.35419000	-3.42689800	1.91906800
C	7.28561300	-3.04816800	1.03421500
H	7.92412900	-2.20233500	0.74778200
H	7.84155200	-3.65066600	1.76143500
H	7.13675200	-3.66403200	0.13771300
C	4.93002800	4.15443900	0.48402800
H	5.87123800	3.85031700	0.95967400
H	5.12195400	5.08256400	-0.06609000
H	4.21969800	4.38611100	1.28941500
H	0.92348100	-2.77233400	0.48882200
H	1.12159200	-1.65425300	1.86607000
H	-1.12655100	-2.19140800	1.75917300
Si	-3.35686400	-0.15825200	0.59567400

C	-4.34268900	-0.58863100	-0.97359000
H	-3.86592400	0.01049800	-1.77017400
C	-3.33155800	1.72156900	0.92034600
H	-2.48343600	1.86641000	1.61308400
C	-4.03116400	-1.00005100	2.16778000
H	-5.03839000	-0.56106300	2.27993800
C	-4.24765800	-2.06239400	-1.37296400
H	-4.57981900	-2.21211900	-2.41045100
H	-3.23138800	-2.47215100	-1.28437300
H	-4.89721900	-2.68437800	-0.74442800
C	-5.80792600	-0.16444600	-0.88135200
H	-6.35945000	-0.45314500	-1.78788900
H	-6.31399500	-0.64855200	-0.03314400
H	-5.92713800	0.91936900	-0.76043900
C	-3.05122800	2.53594000	-0.33895000
H	-3.87231900	2.45009200	-1.06518300
H	-2.94495100	3.60551200	-0.10429500
H	-2.12876000	2.21445600	-0.83780800
C	-4.59328900	2.23490600	1.61496000
H	-5.48954500	2.07616600	1.00018200
H	-4.77324500	1.75317400	2.58382600
H	-4.52266500	3.31676300	1.79887100
C	-3.22250600	-0.61734000	3.40822000
H	-3.71445500	-0.97912500	4.32218200
H	-2.21892600	-1.06337800	3.38648700
H	-3.09108600	0.46701800	3.51610900
C	-4.19854600	-2.51539700	2.08502700
H	-4.54599500	-2.91973200	3.04691200
H	-4.93274400	-2.81188400	1.32717100

H	-3.25526700	-3.02717400	1.84603900

D-TS10a

M06/BSI SCF energy in THF: -1573.201359 a.u.

M06/BSII SCF energy in THF: -1573.491362 a.u.

M06/BSII free energy in THF: -1572.840227 a.u.

C	1.15412800	-0.67013100	2.76700500
C	-0.13335900	-1.30533900	2.83817300
C	-1.13056300	-0.28980400	2.82724600
C	-0.48650600	0.95168800	2.62028300
C	0.93952300	0.72380900	2.60843000
Ru	0.07361000	-0.31937700	0.89101200
C	0.86245100	-1.43117300	-1.04995900
H	-2.19942900	-0.46091100	2.86452800
H	-0.31246100	-2.37023000	2.93415200
H	2.10882800	-1.18117600	2.80042900
H	1.69862200	1.49170400	2.51703700
H	-0.97053000	1.91530300	2.50160900
C	-1.58705900	-0.88381200	-0.03761100
C	-0.70368600	-1.74902400	-0.88539500
C	-0.97291900	3.19103400	-1.07504800
N	-0.42050200	2.26193000	-0.65043600
C	-1.66436700	4.34250300	-1.61213900
H	-0.95709300	4.99391700	-2.13413700
H	-2.13546600	4.91087700	-0.80454000
H	-2.43348000	4.01872300	-2.32063700
C	-2.89157900	-0.61340300	-0.15997900
C	1.65400800	-0.64465500	-0.32046000

C	-3.73774300	-1.37584200	-1.15420000
H	-4.10714700	-0.68677000	-1.93068000
H	-3.13034400	-2.12788000	-1.67401900
C	-3.58809300	0.46802400	0.62003900
H	-2.85896300	0.99463400	1.24754300
H	-4.33657300	0.03100500	1.30156300
C	-4.27989200	1.49563600	-0.27629200
H	-3.57293400	1.83672500	-1.05185400
H	-5.11975600	1.03248700	-0.81635400
C	-4.79067500	2.69282700	0.51036700
H	-5.48333400	2.34286800	1.29106800
H	-3.94861900	3.16167500	1.04495300
C	-4.93249000	-2.07872100	-0.51029700
H	-4.57892200	-2.71671200	0.31599600
H	-5.61148000	-1.34060900	-0.05670900
C	-5.71068200	-2.92282200	-1.50904500
H	-6.03714200	-2.28418600	-2.34413900
H	-5.03827100	-3.67503000	-1.94863700
C	-6.91221900	-3.60627500	-0.88077800
H	-7.61613400	-2.87102000	-0.46949200
H	-7.46008800	-4.21727300	-1.60695500
H	-6.60857200	-4.26503400	-0.05659200
C	-5.48070400	3.71750000	-0.37227400
H	-6.34509500	3.27872400	-0.88700600
H	-5.83959200	4.57876800	0.20221100
H	-4.79994400	4.09718000	-1.14668300
H	-0.76678800	-2.81347700	-0.62838200
H	-1.03588700	-1.61324200	-1.92167000
H	1.22706800	-2.02649400	-1.89151600

Si	3.46164200	-0.15160000	-0.56447800
C	4.36926300	-0.47654100	1.07423100
H	3.82601400	0.14013400	1.81220600
C	3.43432600	1.69475500	-1.02993000
H	2.63829500	1.76837900	-1.79229400
C	4.18921500	-1.11039900	-2.04221900
H	5.22478800	-0.73150600	-2.10951500
C	4.29977400	-1.93298000	1.53727800
H	4.56313700	-2.02080900	2.60128500
H	3.30904000	-2.38919600	1.39930700
H	5.01617300	-2.55477900	0.98605500
C	5.82333700	-0.00769200	1.04294400
H	6.33178400	-0.23538600	1.99101200
H	6.39005300	-0.51435600	0.24765500
H	5.91479800	1.07264900	0.87621400
C	3.04171900	2.59677900	0.13608500
H	3.78950100	2.56336000	0.94185900
H	2.96599400	3.64567900	-0.18586900
H	2.06956000	2.31682200	0.56066600
C	4.74051300	2.17688100	-1.66165600
H	5.57322900	2.13039200	-0.94721500
H	5.03441400	1.59138700	-2.54197900
H	4.65474900	3.22481500	-1.98273500
C	3.48345400	-0.75727500	-3.35295700
H	3.99103400	-1.23035300	-4.20547000
H	2.44453400	-1.11455400	-3.36217900
H	3.45443400	0.32183000	-3.54765900
C	4.25866300	-2.62623000	-1.87053400
H	4.63138300	-3.10217900	-2.78908300

H	4.92936300	-2.92679300	-1.05781200
H	3.27191700	-3.06453300	-1.66214300

D-TS11a

M06/BSI SCF energy in THF: -1573.208381 a.u.

M06/BSII SCF energy in THF: -1573.498359 a.u.

M06/BSII free energy in THF: -1572.845564 a.u.

C	-0.32015900	-3.03622800	-2.07932600
C	0.65094300	-3.25759800	-1.05309500
C	1.81372700	-2.51881900	-1.41602300
C	1.57012500	-1.85255500	-2.65859300
C	0.24248600	-2.18252900	-3.07644300
Ru	0.15541800	-1.12201100	-1.15419100
C	-0.09821200	-1.54936000	1.80045100
H	2.73045000	-2.44958600	-0.84117600
H	0.52516300	-3.88058000	-0.17537300
H	-1.33592500	-3.41980000	-2.07680700
H	-0.25476300	-1.82982000	-3.97257800
H	2.26716100	-1.20505300	-3.17869200
C	1.43587600	-0.49799300	0.59210600
C	1.37525800	-1.54682300	1.68516600
C	-0.41389200	1.61630800	-2.75887800
N	-0.18777300	0.74164600	-2.03196900
C	-0.74705400	2.71684900	-3.63071100
H	-1.35024400	2.35653400	-4.47043200
H	0.16258200	3.18143800	-4.02444400
H	-1.32301700	3.46695600	-3.07571500
C	2.26554100	0.56227300	0.57981800

C	-0.58464200	-0.81174300	0.77146300
C	3.40376300	0.65924600	1.57762300
H	3.60086100	1.72562000	1.77260200
H	3.14215000	0.21888300	2.54853800
C	2.20265000	1.73123500	-0.36573900
H	1.94329500	1.41145700	-1.38096400
H	3.20131300	2.19097100	-0.43013000
C	1.20211200	2.78288200	0.10687900
H	0.20089100	2.32350300	0.13412700
H	1.42909200	3.06580600	1.14846600
C	1.16270300	4.03055600	-0.75972800
H	2.11697100	4.57075100	-0.67011700
H	1.08657000	3.73849800	-1.81977200
C	4.68559400	0.00323300	1.06844000
H	4.50681100	-1.07806600	0.94189900
H	4.92695400	0.39000500	0.06453100
C	5.87198400	0.22075600	1.99511400
H	6.05988200	1.30095100	2.09066500
H	5.60979800	-0.12984800	3.00519700
C	7.12813800	-0.48329500	1.51348600
H	7.42147700	-0.13355800	0.51487000
H	7.97638700	-0.31077800	2.18553700
H	6.97348600	-1.56818600	1.44792500
C	0.00555500	4.94287100	-0.39037500
H	0.06562200	5.24934900	0.66250000
H	-0.01687600	5.85423300	-0.99889700
H	-0.96135900	4.43335900	-0.52055800
H	1.78425700	-2.53225100	1.40828300
H	1.87997100	-1.25086800	2.61459100

H	-0.67281000	-1.99254400	2.61392500
Si	-2.36606600	-0.04374100	0.98254100
C	-3.57105100	-1.52418000	0.87828700
H	-3.63705000	-1.72879100	-0.20434600
C	-2.81853300	1.22821000	-0.36498200
H	-1.87096000	1.73325400	-0.60493700
C	-2.40680400	0.84586700	2.66718400
H	-3.38826400	1.35108600	2.65250000
C	-3.15095000	-2.82626900	1.55330100
H	-3.91950200	-3.59741000	1.40011600
H	-2.20858700	-3.22117200	1.15186000
H	-3.03002000	-2.71209100	2.63950400
C	-4.95578700	-1.09427000	1.36515600
H	-5.68775700	-1.90182800	1.22227400
H	-4.94741500	-0.85474000	2.43736600
H	-5.33749000	-0.21276000	0.83490500
C	-3.34615100	0.56590800	-1.63795900
H	-4.33528800	0.11747800	-1.47126600
H	-3.47063700	1.30264100	-2.44608000
H	-2.68608400	-0.22938500	-2.01476800
C	-3.79222600	2.31175100	0.10051200
H	-4.76545200	1.90114300	0.40236800
H	-3.40338200	2.89332800	0.94430100
H	-3.98829500	3.02064300	-0.71761000
C	-1.31975000	1.91578200	2.76328100
H	-1.43851800	2.51164000	3.67921300
H	-0.32218100	1.45336900	2.80832000
H	-1.31843200	2.61484800	1.91494800
C	-2.37080600	-0.04321400	3.90405000

H	-2.50036600	0.55969600	4.81418400
H	-3.15795400	-0.80686700	3.90262000
H	-1.40419200	-0.55522000	4.00634700

VIII. References

(1) Dahiya, A.; Schoenebeck, F. Orthogonal and Modular Arylation of Alkynylgermanes. *ACS Catal.* **2022,** *12*, 8048–8054.

(2) Miaosheng Li, G. A. O. D. De Novo Formal Synthesis of (-)-Apicularen A via an Iterative Asymmetric Hydration Sequence. *Org. Lett.* **2006,** *8*, 6087–6090.

(3) Bucher, J.; Wurm, T.; Nalivela, K. S.; Rudolph, M.; Rominger, F.; Hashmi, A. S. Cyclization of Gold Acetylides: Synthesis of Vinyl Sulfonates via Gold Vinylidene Complexes. *Angew. Chem., Int. Ed.* **2014,** *53*, 3854–3858.

(4) Fuse, S.; Ishikawa, H.; Kitamura, H.; Masui, H.; Takahashi, T. One-step Syntheses of Diaza-dioxa-fenestranes via the Sequential (3 + 2) Cycloadditions of Linear Precursors and their Structural Analyses. *Nat. Commun.* **2024,** *15*, 6087.

(5) Thiel, N. O.; Kaewmee, B.; Tran Ngoc, T.; Teichert, J. F. A Simple Nickel Catalyst Enabling an *E*-Selective Alkyne Semihydrogenation. *Chem. Eur. J.* **2020,** *26*, 1597–1603.

(6) Zuo, H.; Irran, E.; Klare, H. F. T.; Oestreich, M. Electrophilic Activation of S-Si Reagents by Silylium Ions for Their Regio- and Diastereoselective Addition Across C-C Multiple Bonds. *Angew. Chem., Int. Ed.* **2024,** *63*, e202401599.

(7) Yan, Q.; Shen, X.; Zi, G.; Hou, G. Rh-Catalyzed Asymmetric Hydrogenation of alpha,beta- and beta,beta-Disubstituted Unsaturated Boronate Esters. *Chem. Eur. J.* **2020,** *26*, 5961–5964.

(8) Wang, Z.; Wang, Y.; Zhang, L. Soft Propargylic Deprotonation: Designed Ligand Enables Au-catalyzed Isomerization of Alkynes to 1,3-dienes. *J. Am. Chem. Soc.* **2014,** *136*, 8887–8890.

(9) Unoh, Y.; Hirano, K.; Satoh, T.; Miura, M. An Approach to Benzophosphole Oxides through Silver- or Manganese-mediated

Dehydrogenative Annulation Involving C-C and C-P Bond Formation. *Angew. Chem., Int. Ed.* **2013,** *52*, 12975–12979.

(10) Han, X. L.; Liu, X. G.; Lin, E.; Chen, Y.; Chen, Z.; Wang, H.; Li, Q. Cp*Co(III)-Catalyzed Oxidative [5+2] Annulation: Regioselective Synthesis of 2-aminobenzoxepines via C-H/O-H Functionalization of 2-vinylphenols with Ynamides. *Chem. Commun.* **2018,** *54*, 11562–11565.

(11) Wang, A.; Lv, P.; Liu, Y. 4,5-Dihydro-1,2,4-oxadiazole as a Single Nitrogen Transfer Reagent: Synthesis of Functionalized Isoxazoles Assisted by $Sc(OTf)_3$ or Au(I)/$Sc(OTf)_3$ Synergistic Catalysis. *Org. Lett.* **2023,** *25*, 4377–4382.

(12) Zhao, X.; Zhu, S.; Qing, F. L.; Chu, L. Reductive Hydrobenzylation of Terminal Alkynes via Photoredox and Nickel Dual Catalysis. *Chem. Commun.* **2021,** *57*, 9414–9417.

(13) Zhang, W.; Liu, T.; Ang, H. T.; Luo, P.; Lei, Z.; Luo, X.; Koh, M. J.; Wu, J. Modular and Practical 1,2-Aryl(Alkenyl) Heteroatom Functionalization of Alkenes through Iron/Photoredox Dual Catalysis. *Angew. Chem., Int. Ed.* **2023,** *62*, e202310978.

(14) Takashi Kippo, T. F., Ilhyong Ryu. Regioselective Radical Bromoallylation of Allenes Leading to 2-Bromo-Substituted 1,5-Dienes. *Org. Lett.* **2011,** *13*, 3864–3867.

(15) Nopper, C.; Veith, A.; Himmelsbach, F.; Sohner, L.; Breit, B. O-p-Methoxyphenyl alpha-Hydroxycarboxylic Acids as Versatile Hydroxyalkylation Agents for Photocatalysis. *Chem. Eur. J.* **2025,** *31*, e202403495.

(16) Wang, C.; Teo, W. J.; Ge, S. Access to Stereodefined (Z)-allylsilanes and (Z)-allylic Alcohols via Cobalt-catalyzed Regioselective Hydrosilylation of Allenes. *Nat. Commun.* **2017,** *8*, 2258.

(17) Geng, H. Q.; Peng, J. B.; Wu, X. F. Palladium-Catalyzed Oxidative Carbonylative Coupling of Arylallenes, Arylboronic Acids, and

Nitroarenes. *Org. Lett.* **2019,** *21*, 8215–8218.

(18) Jun Takaya, N. I. Hydrocarboxylation of Allenes with CO_2 Catalyzed by Silyl Pincer Type Palladium Complex. *J. Am. Chem. Soc.* **2008,** *130*, 15254–15255.

(19) Wu, Q.; Zhang, Z.; Chong, Q.; Meng, F. Photoredox/Cobalt-Catalyzed Chemo-, Regio-, Diastereo- and Enantioselective Reductive Coupling of 1,1-Disubstituted Allenes and Cyclobutenes. *Angew. Chem., Int. Ed.* **2025,** *64*, e202416524.

(20) Tan, Y. X.; Li, S.; Chen, L.; Huang, J.; Zhang, C.; Song, L.; Zhang, X.; Wu, Y. D.; Sun, J. Ruthenium-Catalyzed alpha-Regioselective Hydroboration of Allenes. *Angew. Chem., Int. Ed.* **2025,** *64*, e202420370.

(21) Liu, J.; Nie, M.; Zhou, Q.; Gao, S.; Jiang, W.; Chung, L. W.; Tang, W.; Ding, K. Enantioselective Palladium-catalyzed Diboration of 1,1-disubstituted Allenes. *Chem. Sci.* **2017,** *8*, 5161–5165.

(22) Han, J.; Zhou, W.; Zhang, P.-C.; Wang, H.; Zhang, R.; Wu, H.-H.; Zhang, J. Design and Synthesis of WJ-Phos, and Application in Cu-Catalyzed Enantioselective Boroacylation of 1,1-Disubstituted Allenes. *ACS Catal.* **2019,** *9*, 6890–6895.

(23) Dolomanov, O. V.; Bourhis, L. J.; Gildea, R. J.; Howard, J. A. K.; Puschmann, H. OLEX2: A Complete Structure Solution, Refinement and Analysis Program. *J. Appl. Cryst.* **2009**, *42*, 339–341.

(24) Sheldrick, G. M. Crystal Structure Solution with ShelXT. *Acta Cryst.* **2015**, *A71*, 3–8.

(25) Sheldrick, G. M. Crystal Structure Refinement with SHELXL. *Acta Cryst.* **2015**, *C71*, 3–8.

(26) Marenich, A. V.; Cramer, C. J.; Truhlar, D. G. Universal Solvation Model Based on Solute Electron Density and on a Continuum Model of the Solvent Defined by the Bulk Dielectric Constant and Atomic Surface Tensions. *J. Phys. Chem. B.* **2009**, *113*, 6378–6396.

(27) (a) Zhao, Y.; Truhlar, D. G. Benchmark Energetic Data in a Model System for Grubbs II Metathesis Catalysis and Their Use for the Development, Assessment, and Validation of Electronic Structure Methods. *J. Chem. Theory Comput.* **2009**, *5*, 324–333. (b) Zhao, Y.; Truhlar, D. G. The M06 Suite of Density Functionals for Main Group Thermochemistry, Thermochemical kinetics, Noncovalent interactions, Excited States, and Transition Elements: Two New Functionals and Systematic Testing of Four M06-class Functionals and 12 Other Functionals. *Theor. Chem. Acc.* **2008**, *120*, 215–241. (c) Zhao, Y.; Truhlar, D. G. Density Functionals with Broad Applicability in Chemistry. *Acc. Chem. Res.* **2008**, *41*, 157–167.

(28) (a) Dolg, M.; Wedig, U.; Stoll, H.; Preuss, H. Energy-adjusted Ab Initio Pseudopotentials for the First Row Transition elements. *J. Chem. Phys.* **1987**, *86*, 866–872. (b) Roy, L. E.; Hay, P. J.; Martin, R. L. Revised Basis Sets for the LANL Effective Core Potentials. *J. Chem. Theory. Comput.* **2008**, *4*, 1029–1031.

(29) Fukui, K. The Path of Chemical Reactions —The IRC Approach. *Acc. Chem. Res.***1981**, *14*, 363–368.

(30) Frisch, M. J.; Trucks, G. W.; Schlegel, H. B.; Scuseria, G. E.; Robb, M. A.; Cheeseman, J. R.; Scalmani, G.; Barone, V.; Mennucci, B.; Petersson, G. A.; Nakatsuji, H.; Caricato, M.; Li, X.; Hratchian, H. P.; Izmaylov, A. F.; Bloino, J.; Zheng, G.; Sonnenberg, J. L.; Hada, M.; Ehara, M.; Toyota, K.; Fukuda, R.; Hasegawa, J.; Ishida, M.; Nakajima, T.; Honda, Y.; Kitao, O.; Nakai, H.; Vreven, T.; Montgomery, J. A., Jr.; Peralta, J. E.; Ogliaro, F.; Bearpark, M.; Heyd, J. J.; Brothers, E.; Kudin, K. N.; Staroverov, V. N.; Keith, T.; Kobayashi, R.; Normand, J.; Raghavachari, K.; Rendell, A.; Burant, J. C.; Iyengar, S. S.; Tomasi, J.; Cossi, M.; Rega, N.; Millam, J. M.; Klene, M.; Knox, J. E.; Cross, J. B.; Bakken, V.; Adamo, C.; Jaramillo, J.; Gomperts, R.; Stratmann, R. E.; Yazyev, O.; Austin, A. J.; Cammi, R.; Pomelli, C.; Ochterski, J. W.;Martin, R. L.; Morokuma, K.; Zakrzewski, V. G.; Voth, G.

A.; Salvador, P.; Dannenberg, J. J.; Dapprich, S.; Daniels, A. D.; Farkas, O.; Foresman, J. B.; Ortiz, J. V.; Cioslowski, J.; Fox, D. J. Gaussian 09, Rev. A.01; Gaussian, Inc.: Wallingford, CT, 2010.

(31) CYLview20; Legault, C. Y. Université de Sherbrooke, 2020 (http://www.cylview.org).

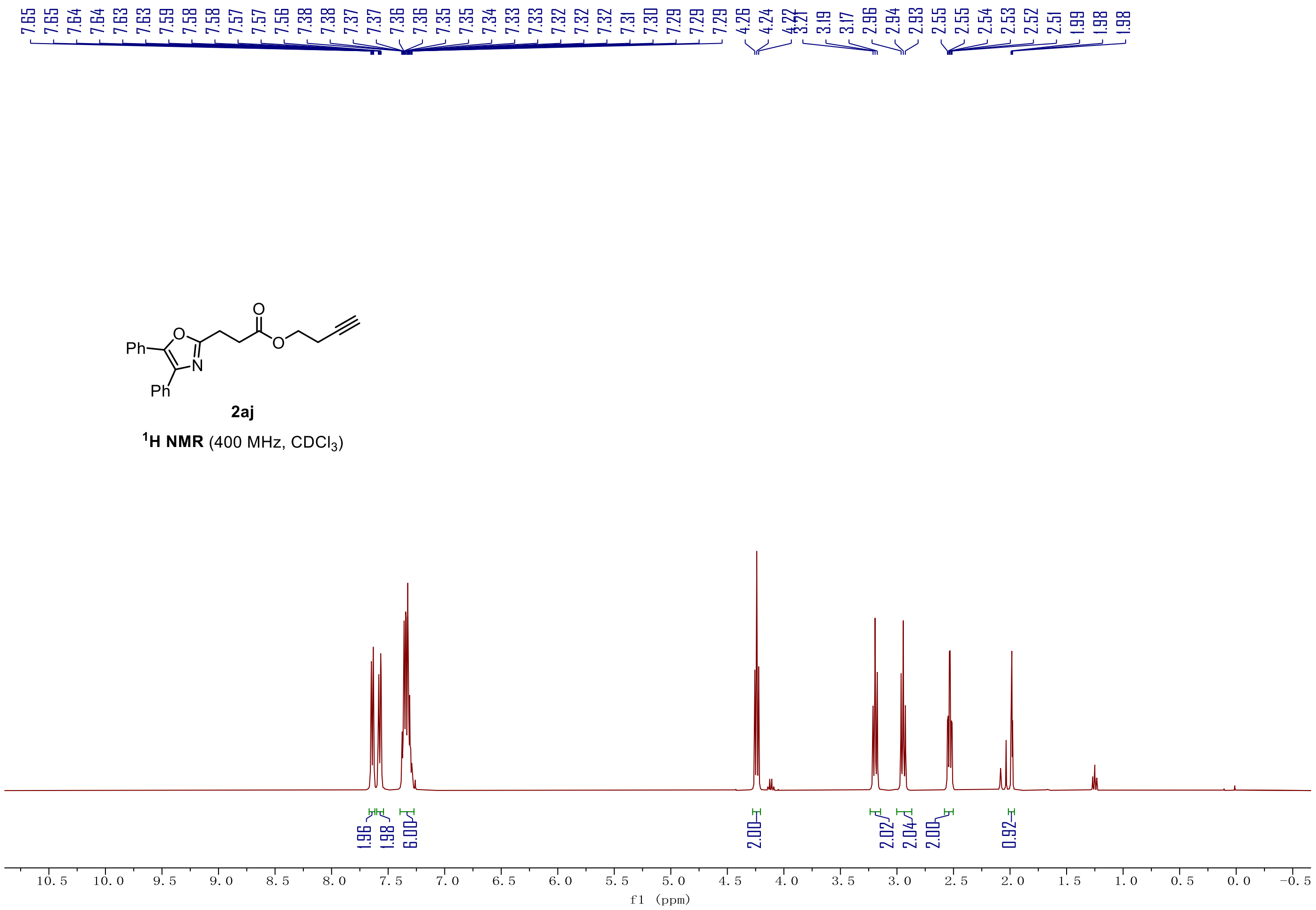

2aj

^{1}H NMR (400 MHz, $CDCl_3$)

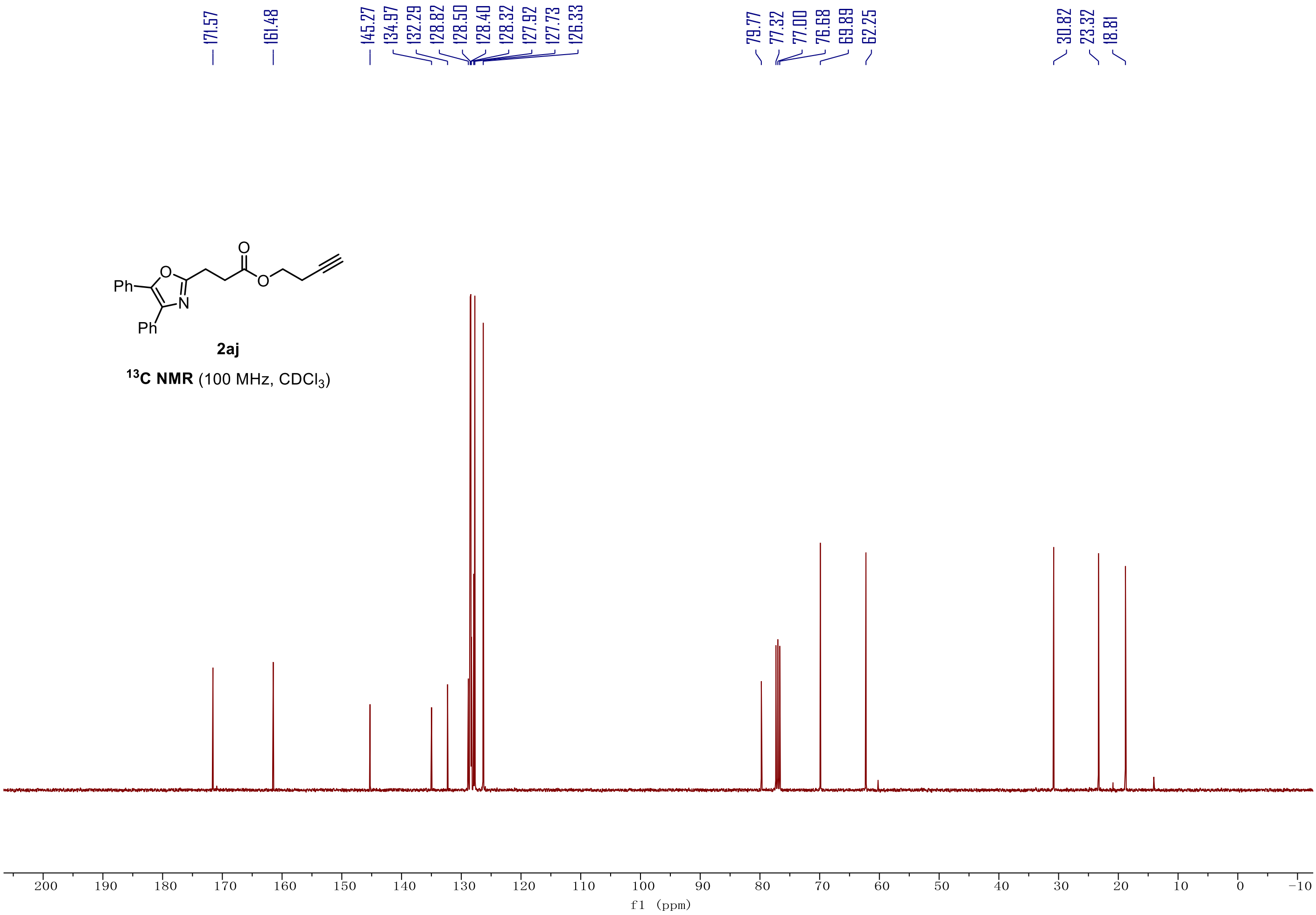

2aj

^{13}C NMR (100 MHz, $CDCl_3$)

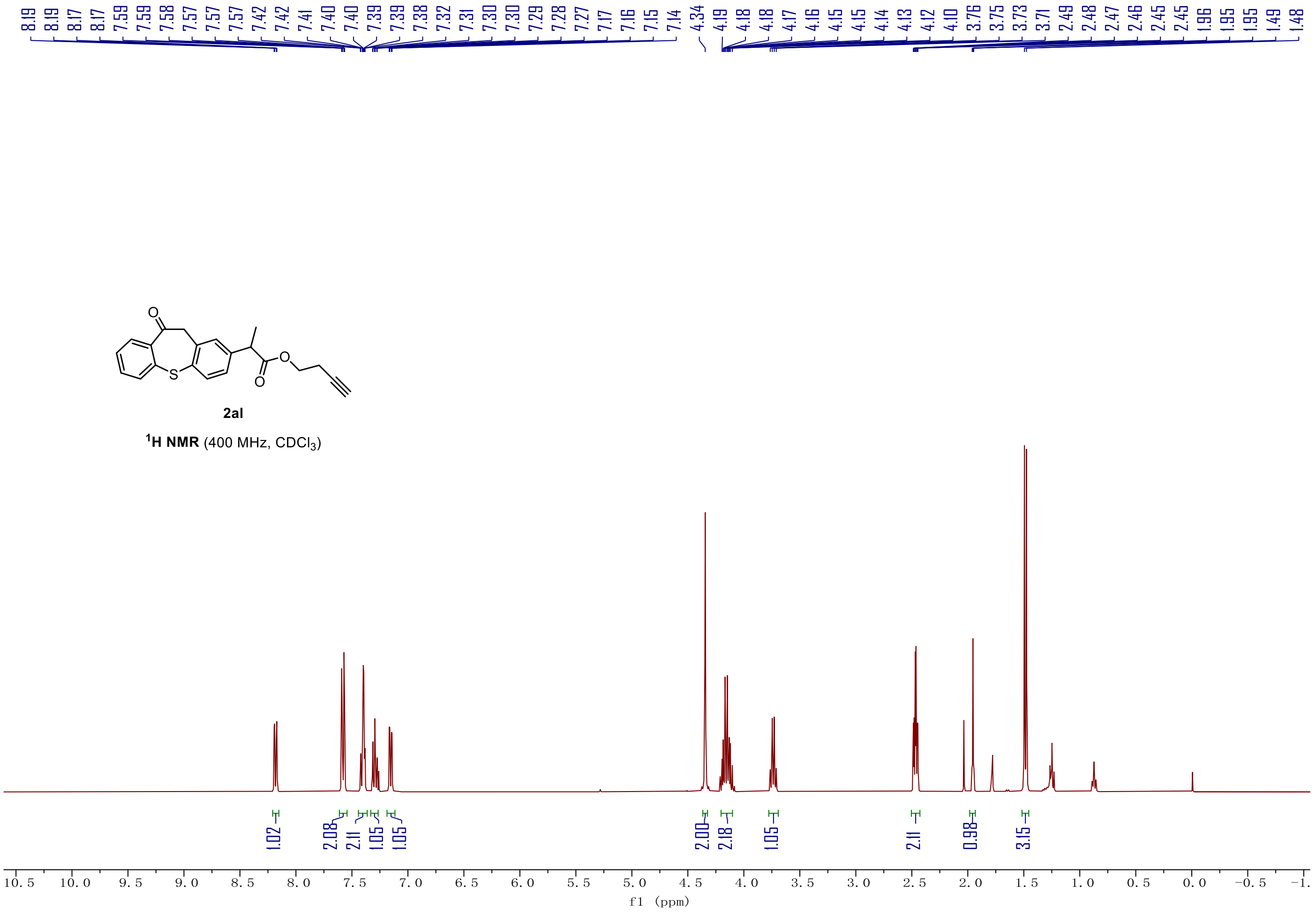
8.19 8.19 8.17 8.17 7.59 7.59 7.58 7.57 7.57 7.57 7.42 7.42 7.41 7.40 7.40 7.39 7.39 7.38 7.32 7.31 7.30 7.30 7.29 7.28 7.27 7.17 7.16 7.15 7.14 4.34 4.19 4.18 4.18 4.17 4.16 4.15 4.15 4.14 4.13 4.12 4.10 3.76 3.75 3.73 3.71 2.49 2.48 2.47 2.46 2.45 2.45 1.96 1.95 1.95 1.49 1.48
2al
^{1}H NMR (400 MHz, $CDCl_3$)
1.02 2.08 2.11 1.05 1.05 2.00 2.18 1.05 2.11 0.98 3.15
f1 (ppm)

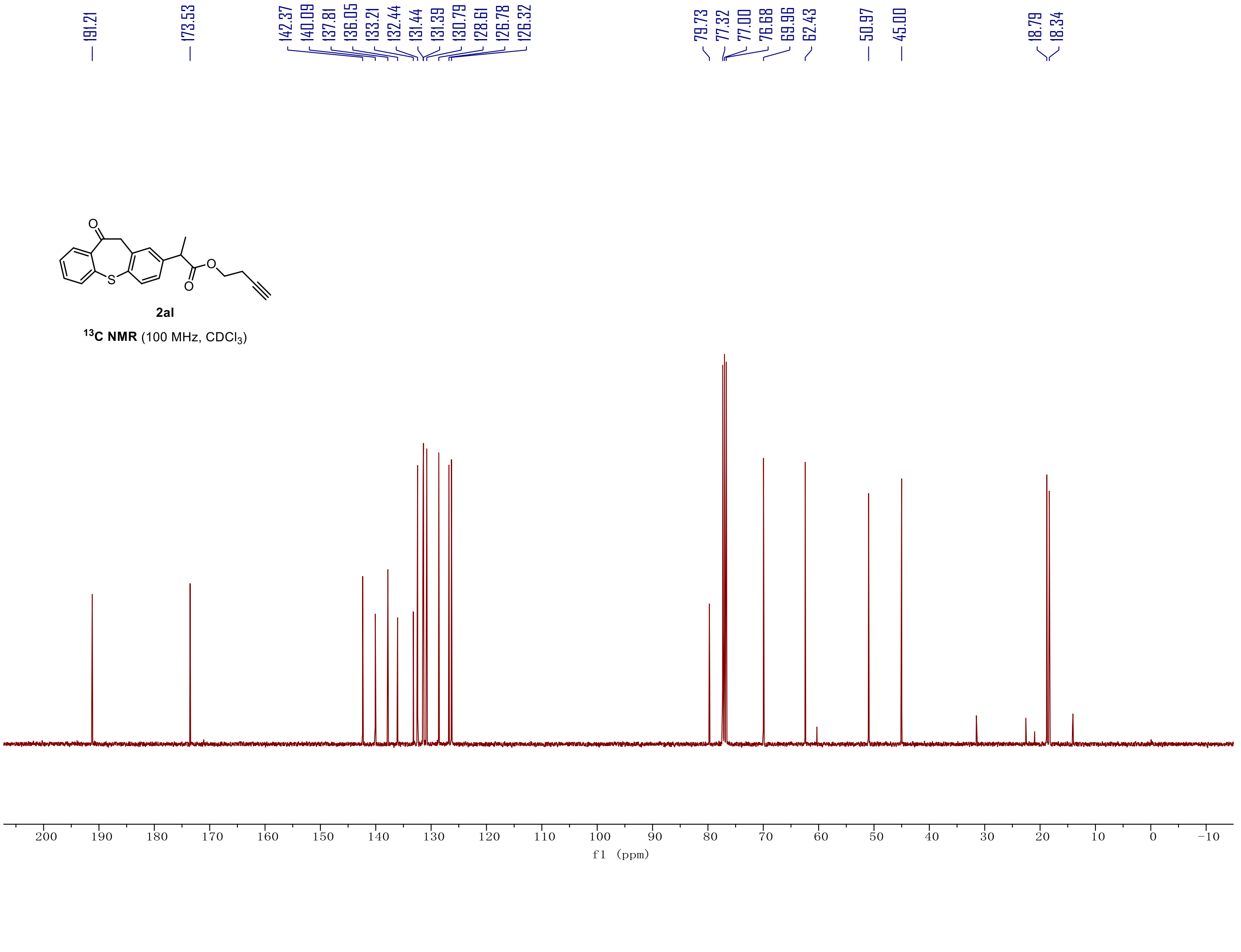
191.21
173.53
142.37 140.09 137.81 136.05 133.21 132.44 131.44 131.39 130.79 128.61 126.78 126.32
79.73 77.32 77.00 76.68 69.96 62.43
50.97 45.00
18.79 18.34
2al
^{13}C NMR (100 MHz, $CDCl_3$)
f1 (ppm)

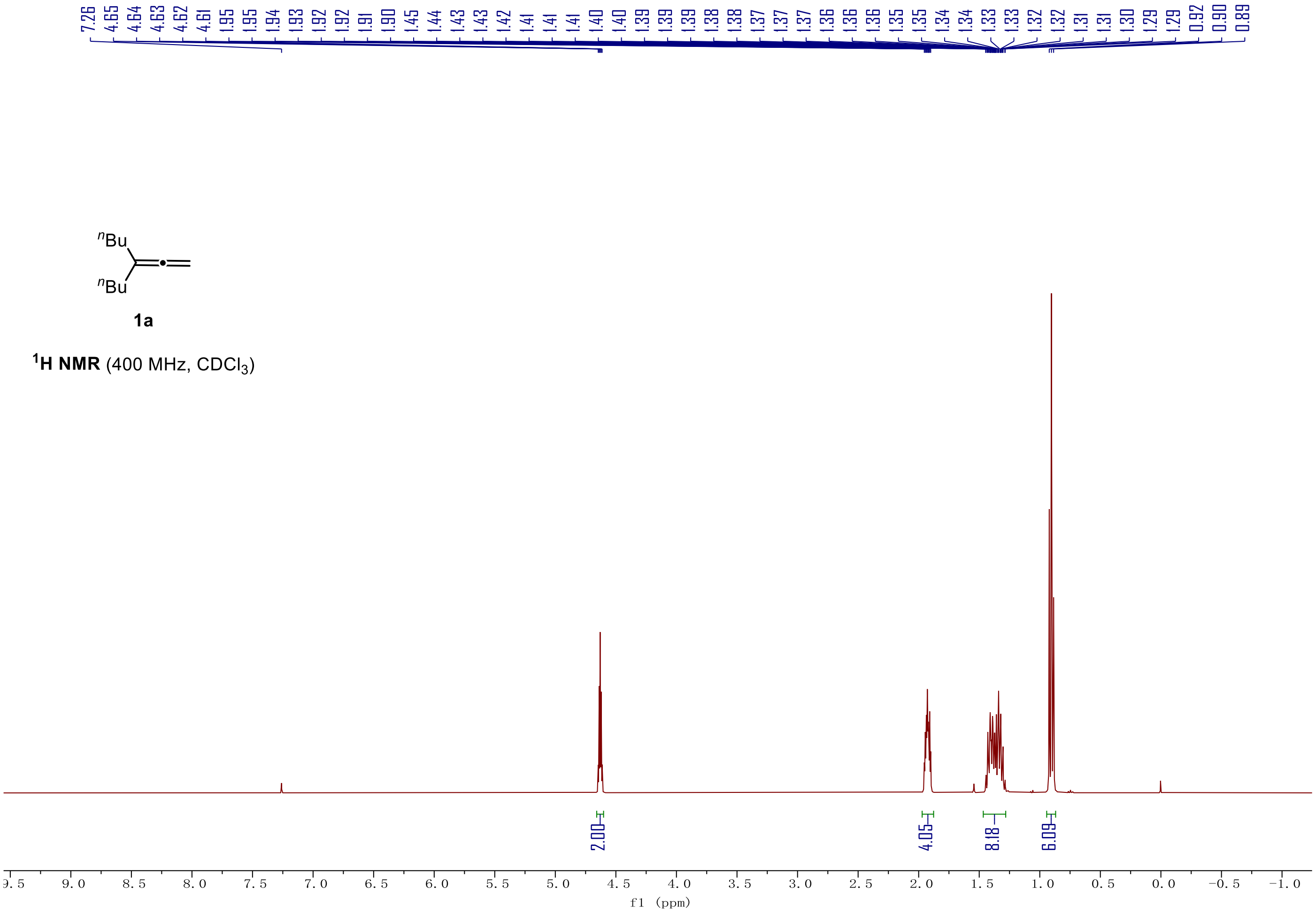
7.26 4.65 4.64 4.63 4.62 4.61 1.95 1.95 1.94 1.93 1.92 1.92 1.91 1.90 1.45 1.44 1.43 1.43 1.42 1.41 1.41 1.41 1.40 1.40 1.39 1.39 1.39 1.38 1.38 1.37 1.37 1.37 1.36 1.36 1.36 1.35 1.35 1.34 1.34 1.33 1.33 1.32 1.32 1.31 1.31 1.30 1.29 1.29 0.92 0.90 0.89
nBu
nBu
1a
1H NMR (400 MHz, CDCl3)
2.00
4.05
8.18
6.09
f1 (ppm)

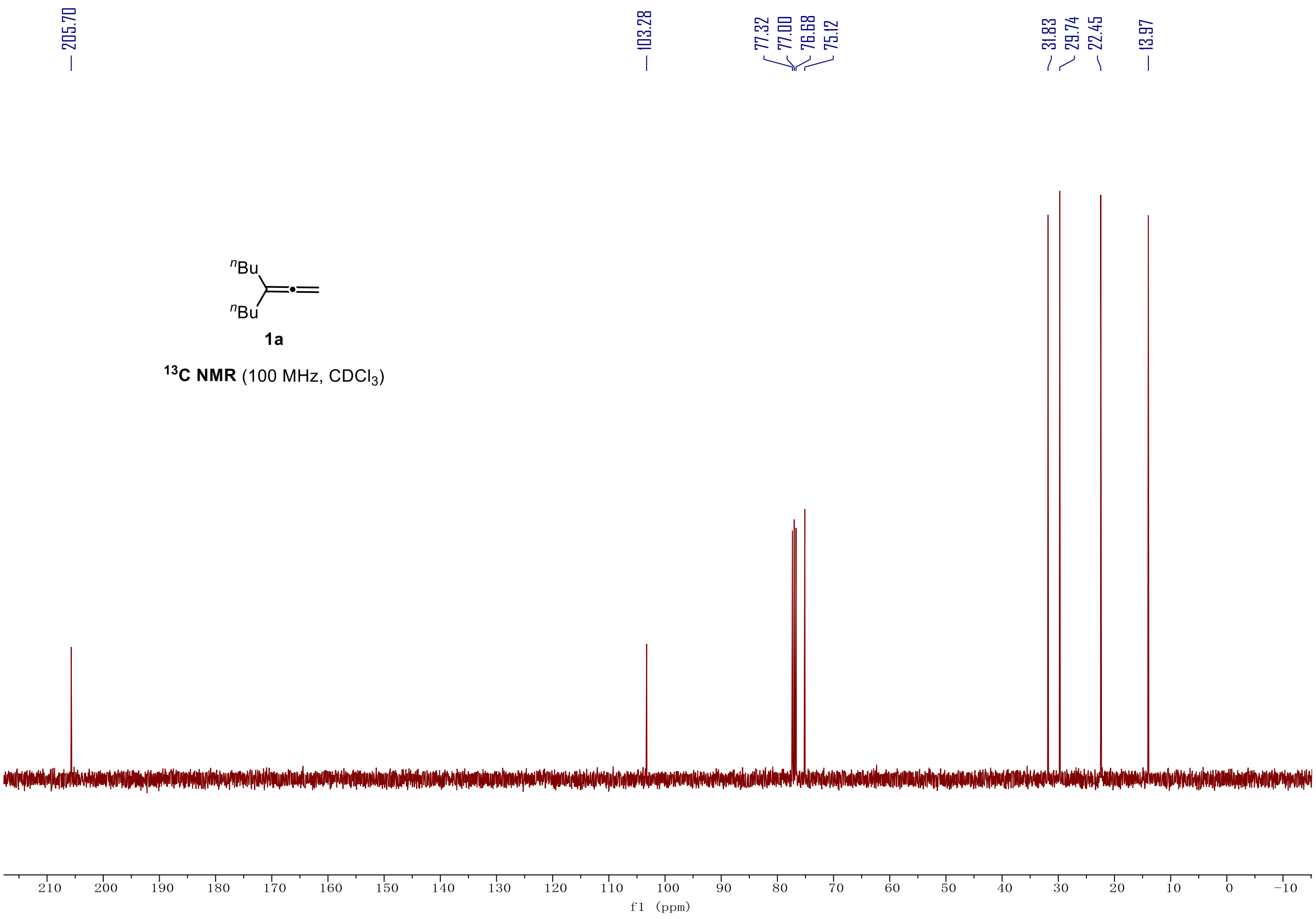
205.70
103.28
77.32
77.00
76.68
75.12
31.83
29.74
22.45
13.97
nBu
nBu
1a
13C NMR (100 MHz, CDCl3)
f1 (ppm)

1b

^{1}H NMR (400 MHz, $CDCl_3$)

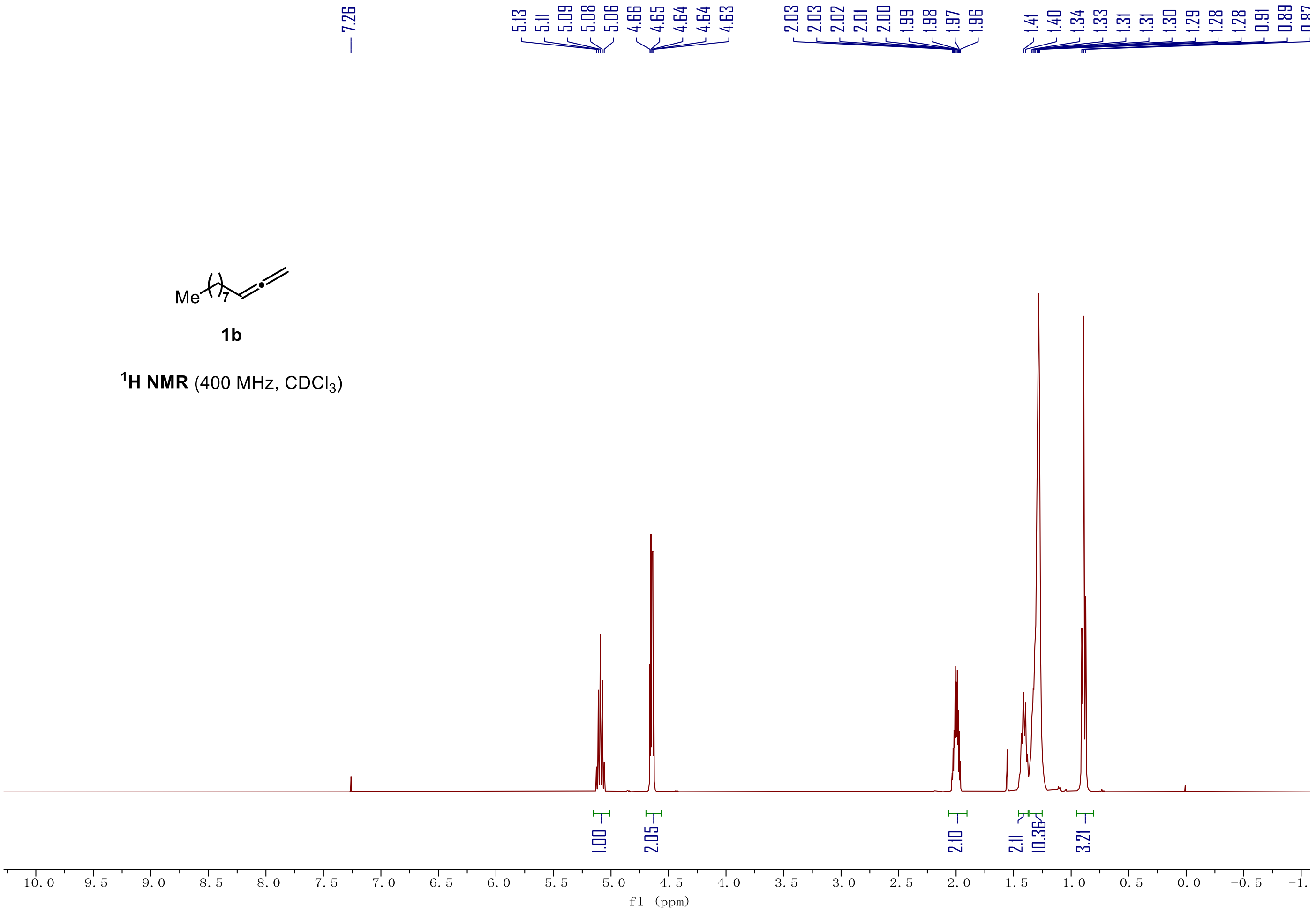

1b

^{13}C NMR (100 MHz, $CDCl_3$)

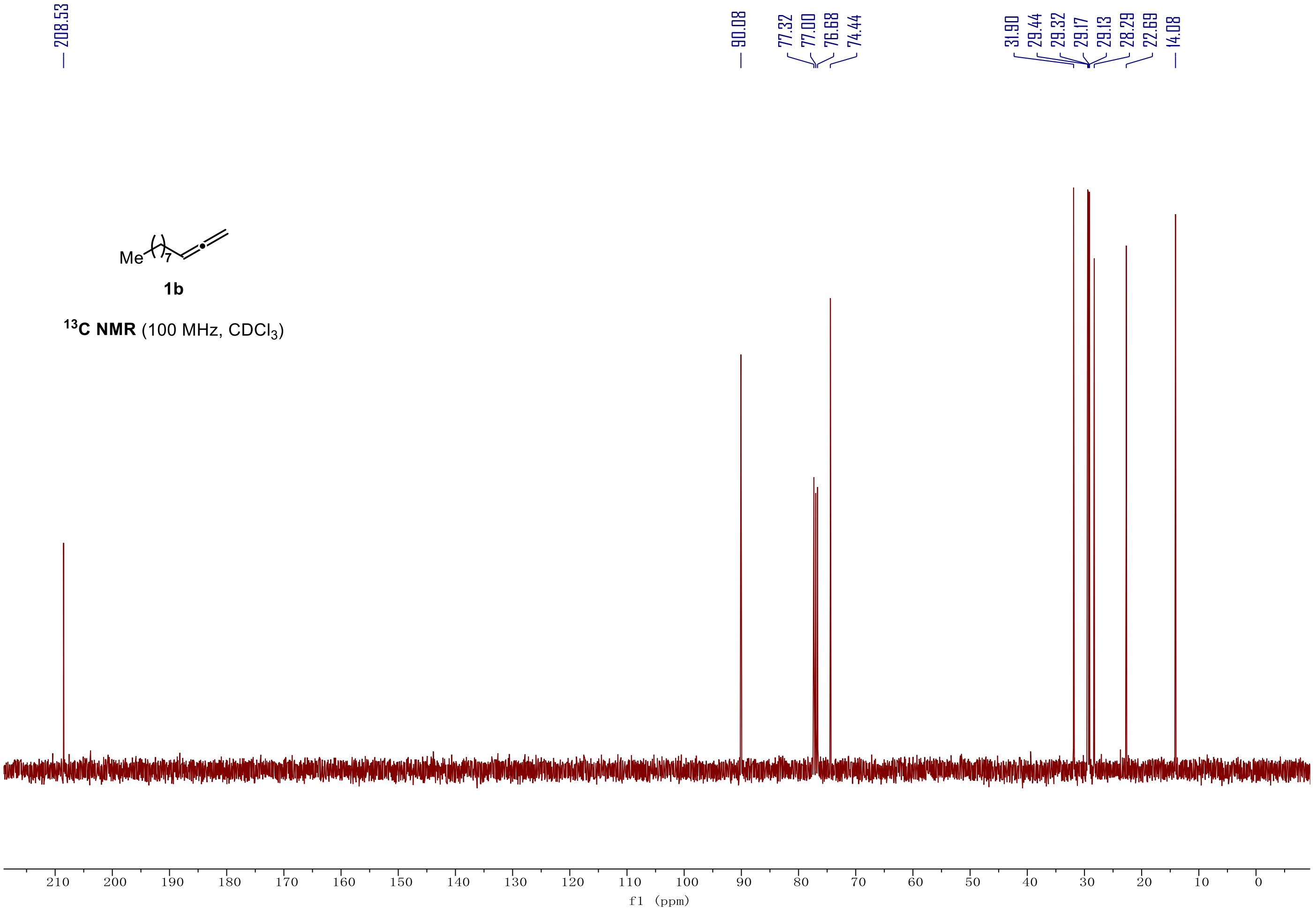

1c

^{1}H NMR (400 MHz, $CDCl_3$)

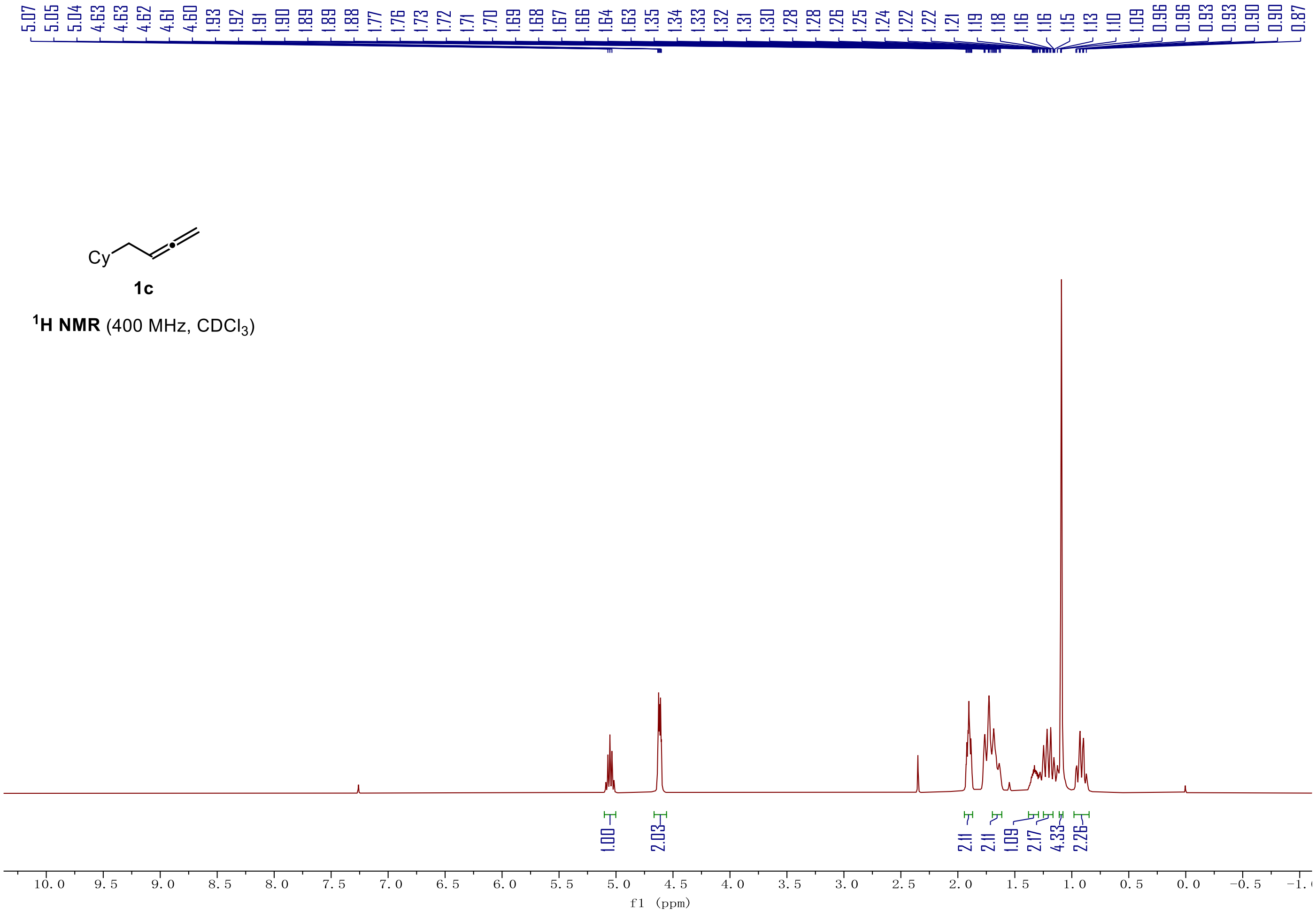

1c

^{13}C NMR (100 MHz, $CDCl_3$)

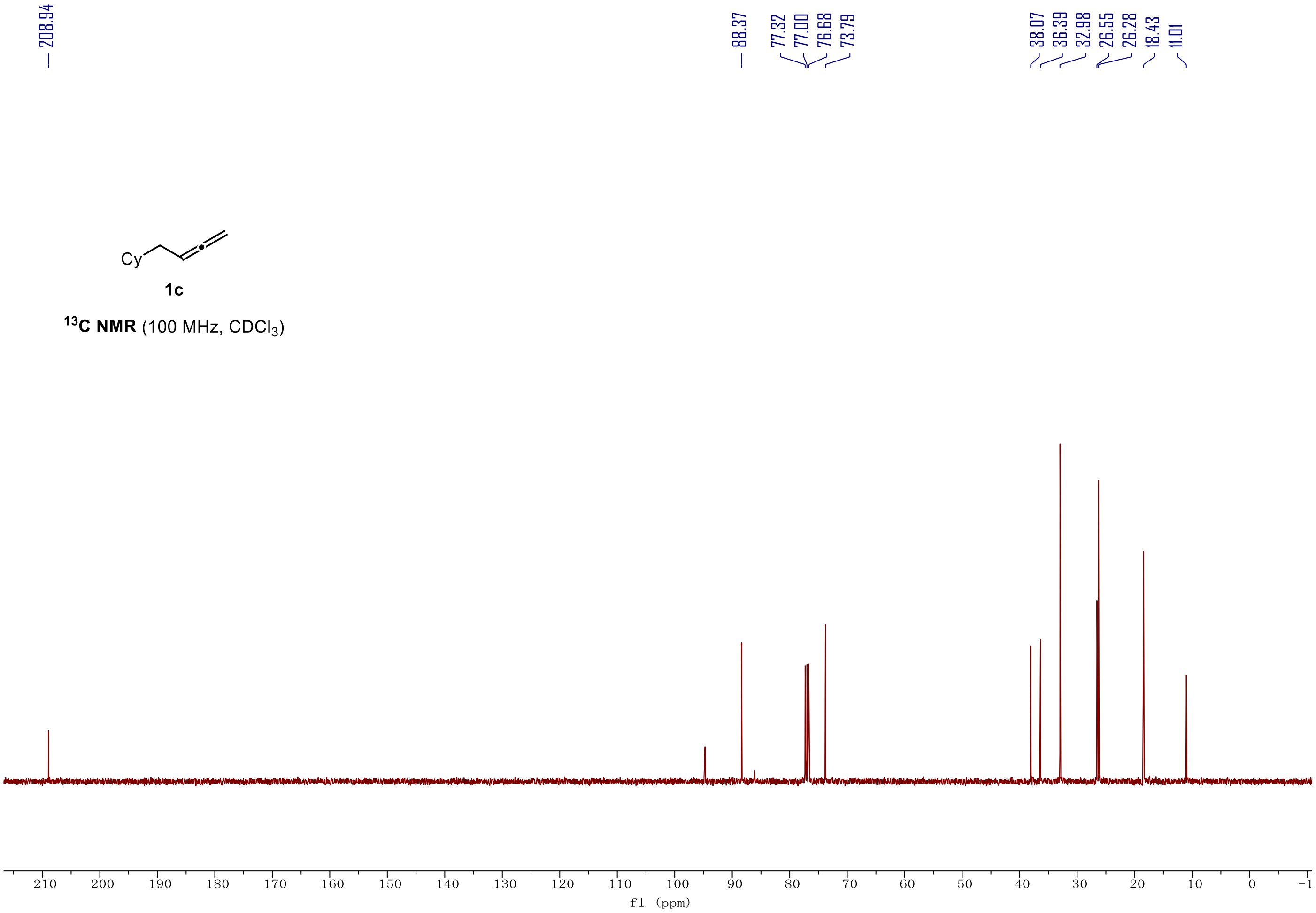

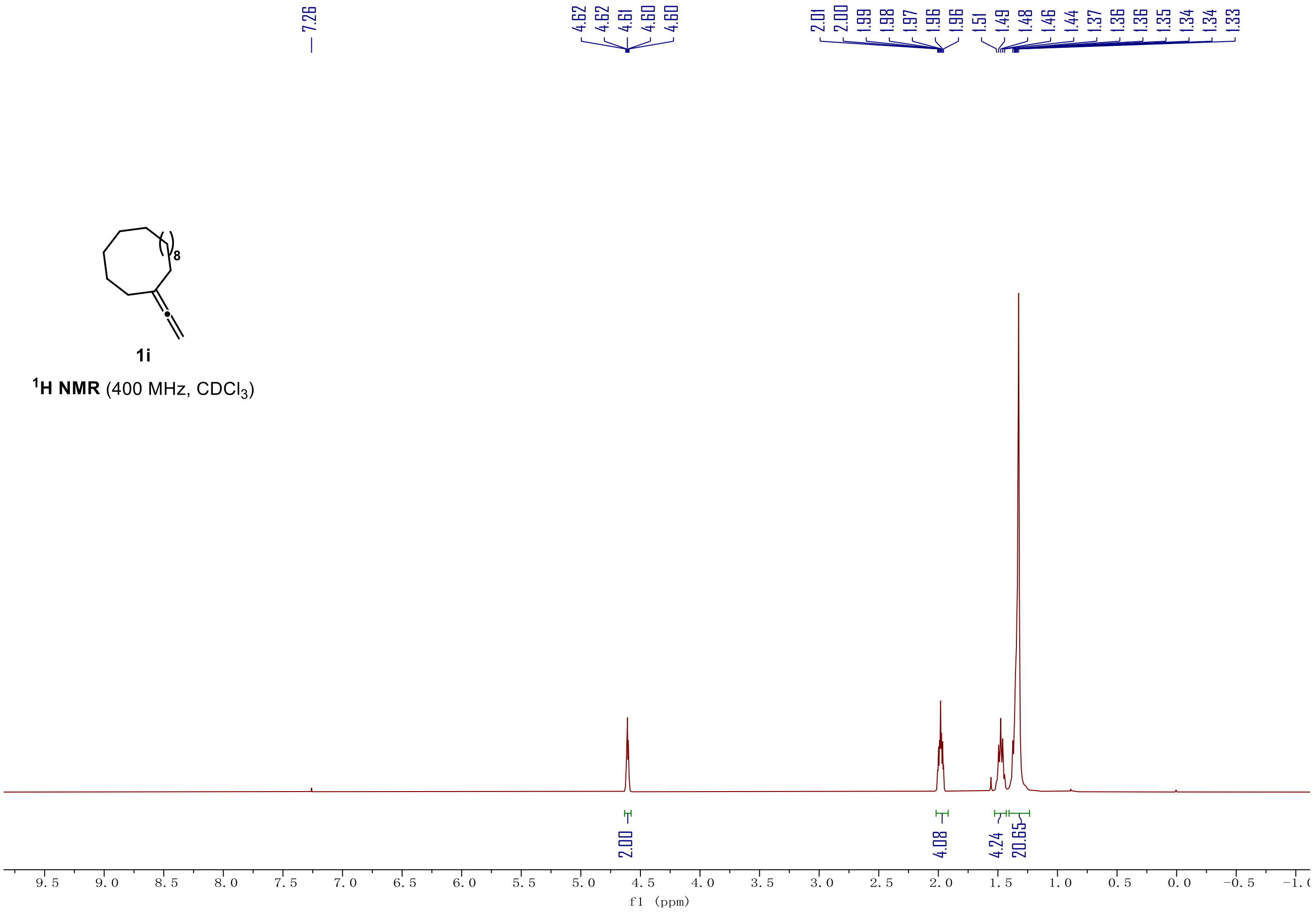
7.26
4.62
4.62
4.61
4.60
4.60
2.01
2.00
1.99
1.98
1.97
1.96
1.96
1.51
1.49
1.48
1.46
1.44
1.37
1.36
1.36
1.35
1.34
1.34
1.33
8
1i
1H NMR (400 MHz, CDCl3)
2.00
4.08
4.24
20.65
f1 (ppm)

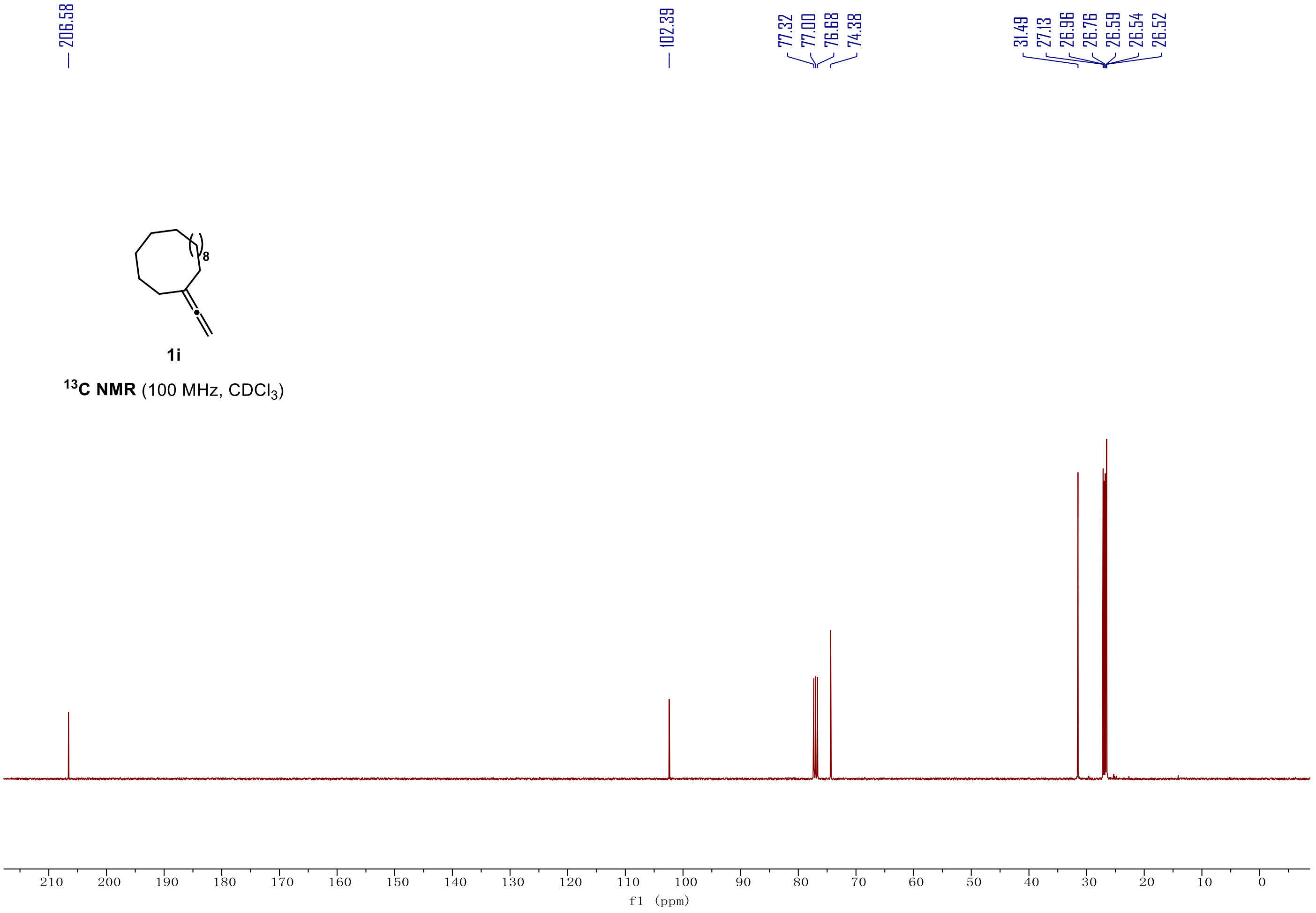
206.58
102.39
77.32
77.00
76.68
74.38
31.49
27.13
26.96
26.76
26.59
26.54
26.52
8
1i
13C NMR (100 MHz, CDCl3)
f1 (ppm)

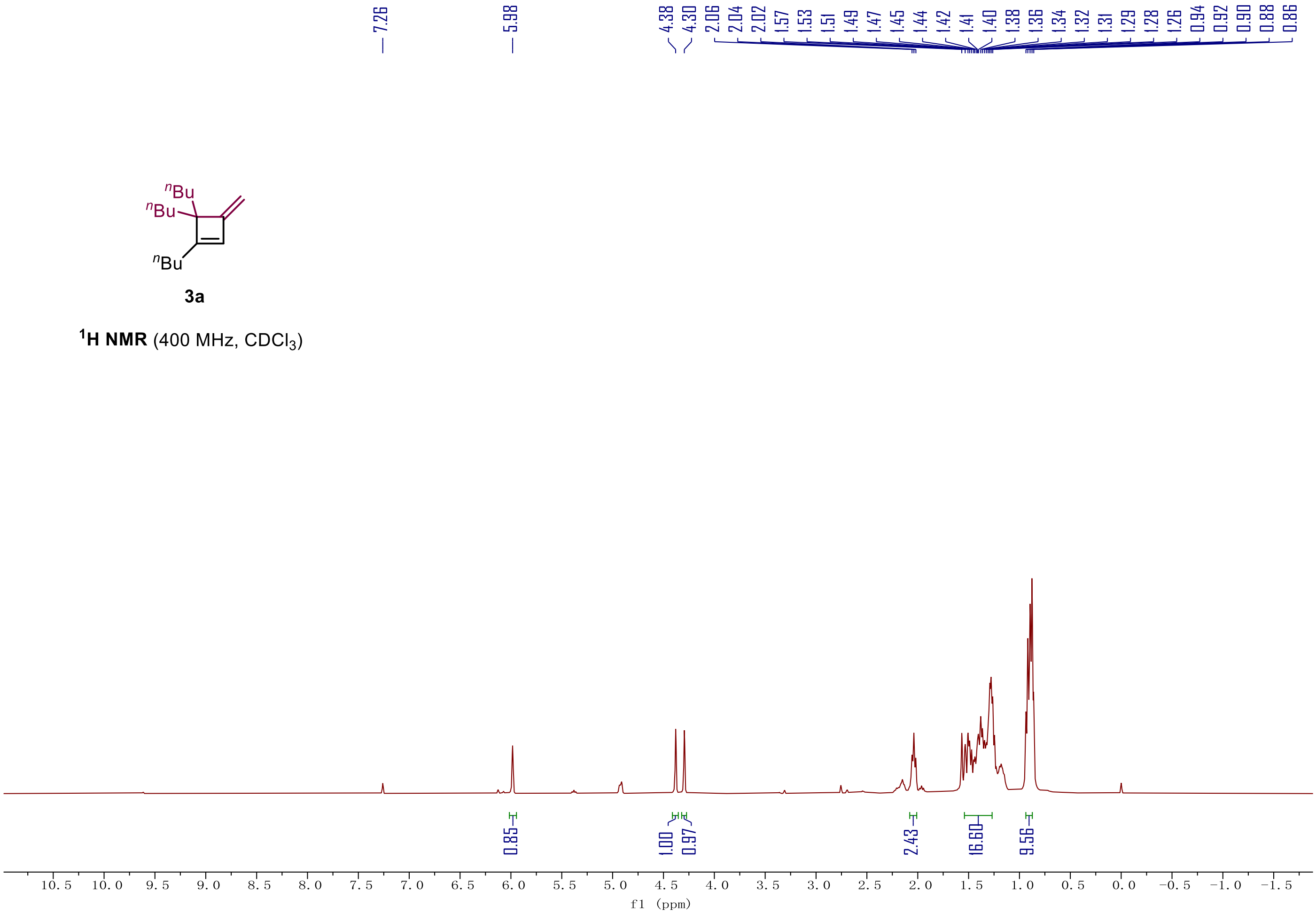

Impurities are not isomers

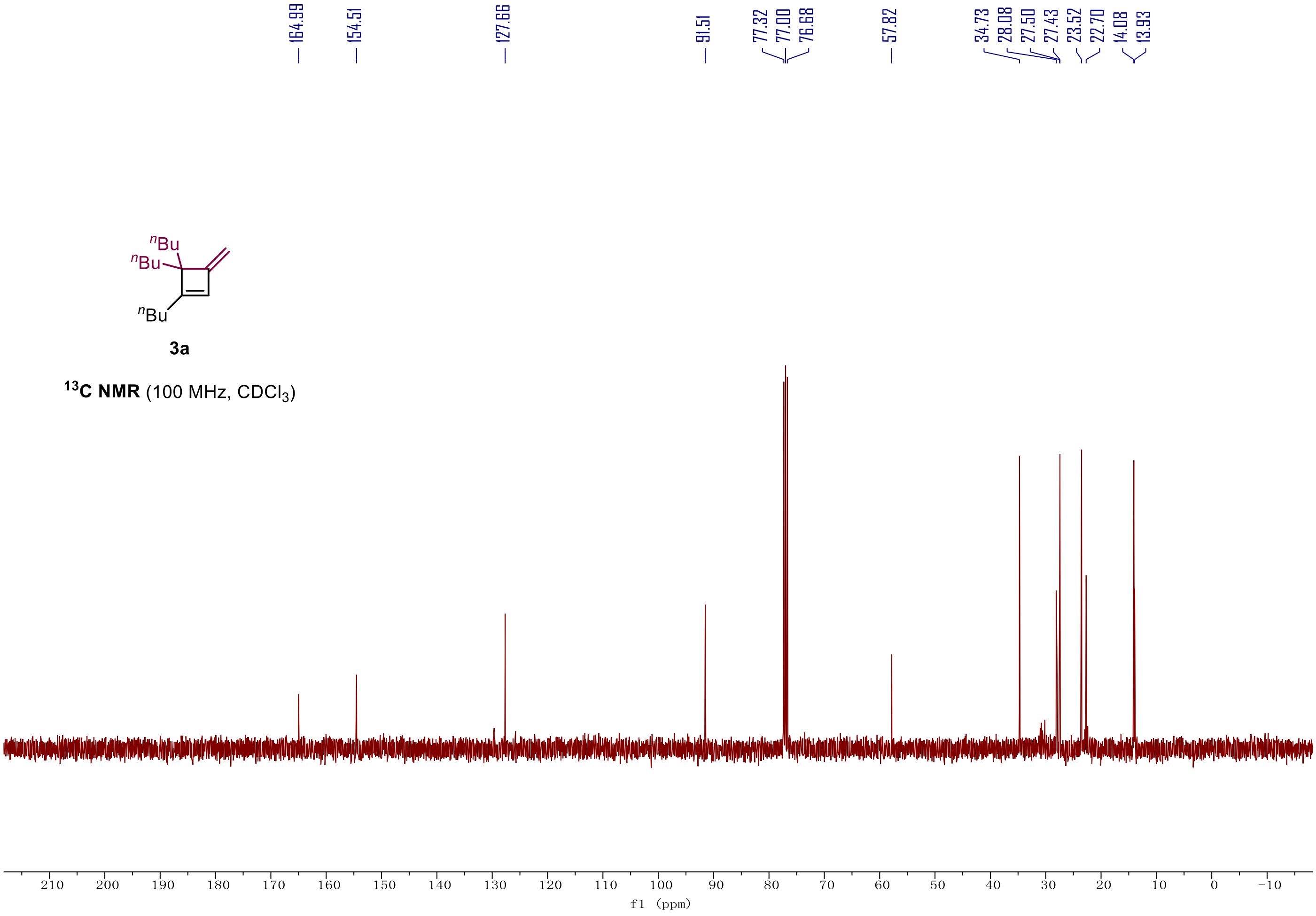

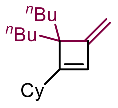

3b

^{1}H NMR (400 MHz, $CDCl_3$)

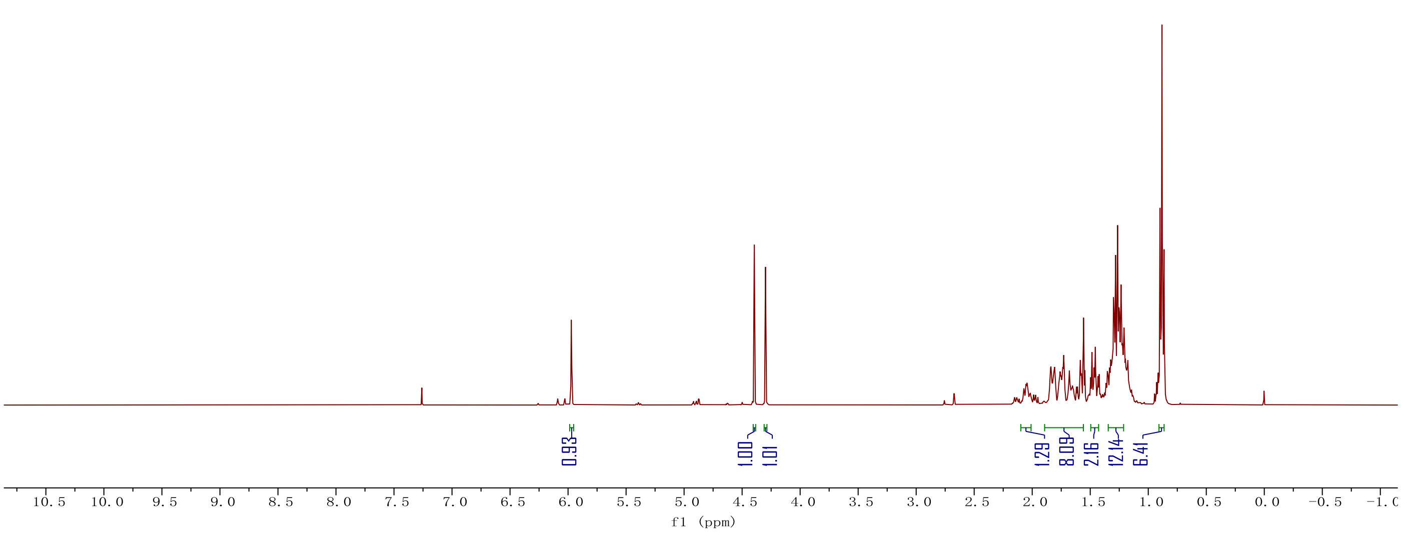

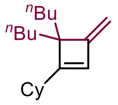

3b

^{13}C NMR (100 MHz, $CDCl_3$)

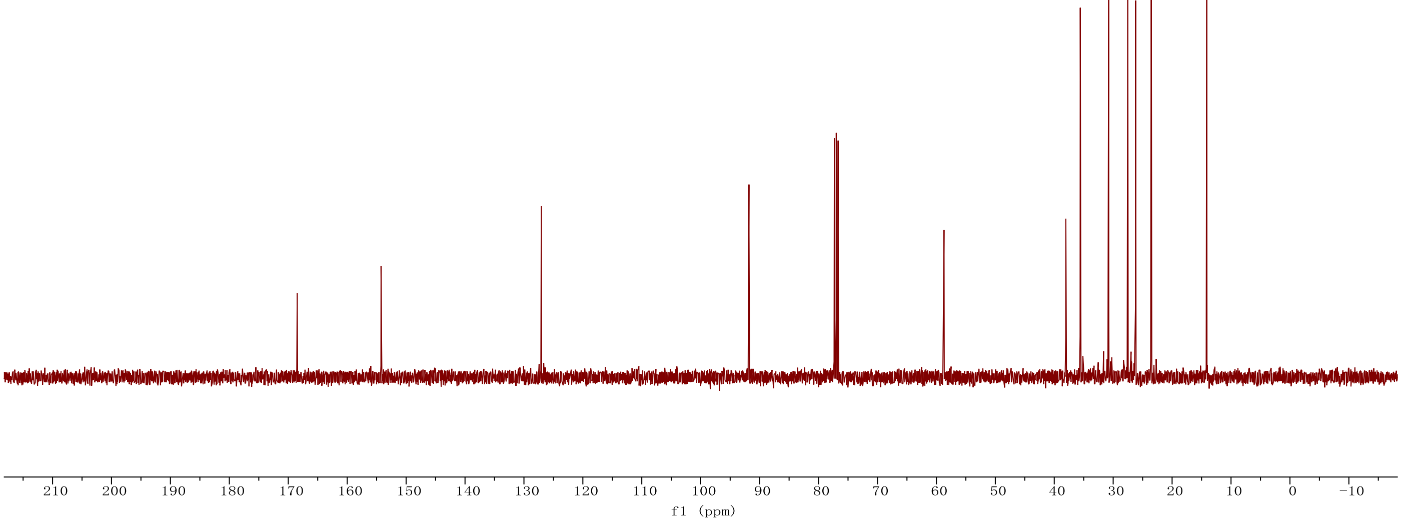

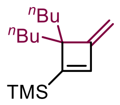

3c

^{1}H NMR (400 MHz, $CDCl_3$)

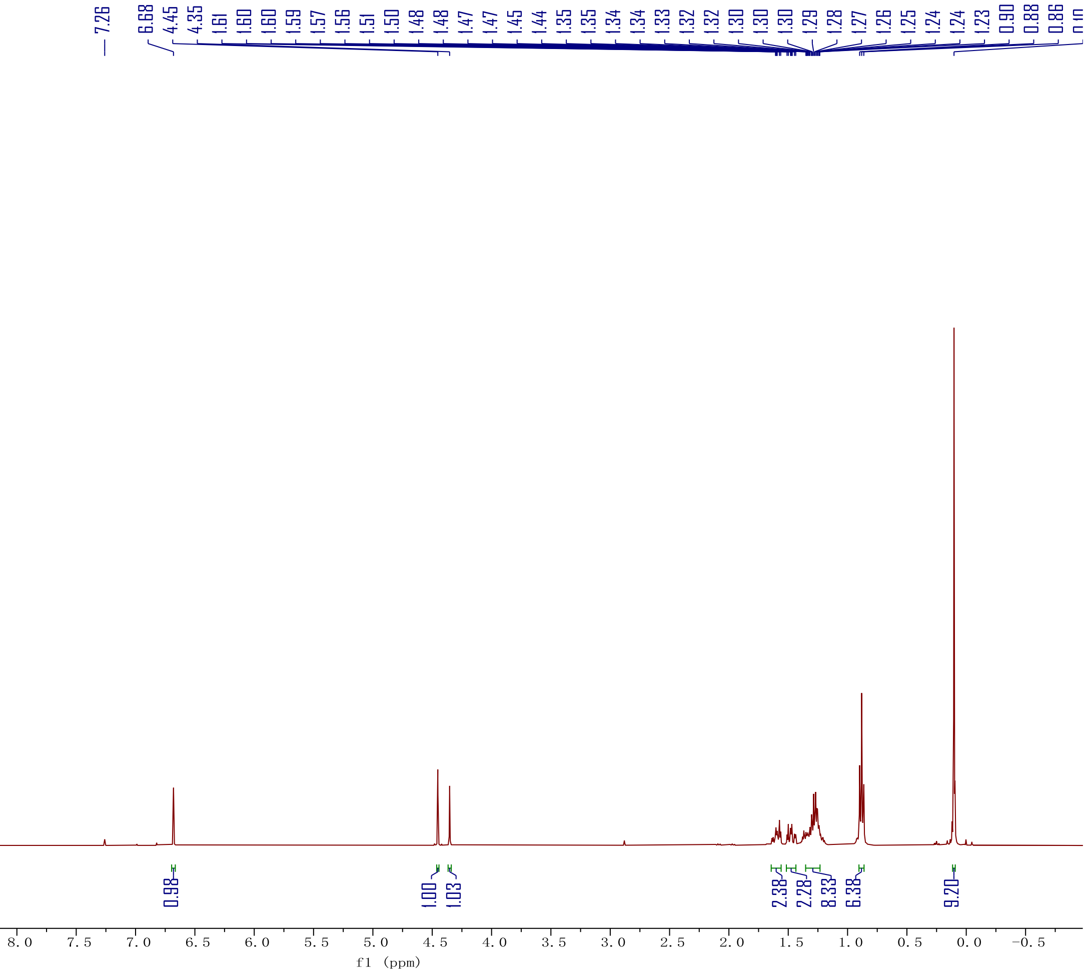

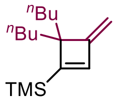

3c

^{13}C NMR (100 MHz, $CDCl_3$)

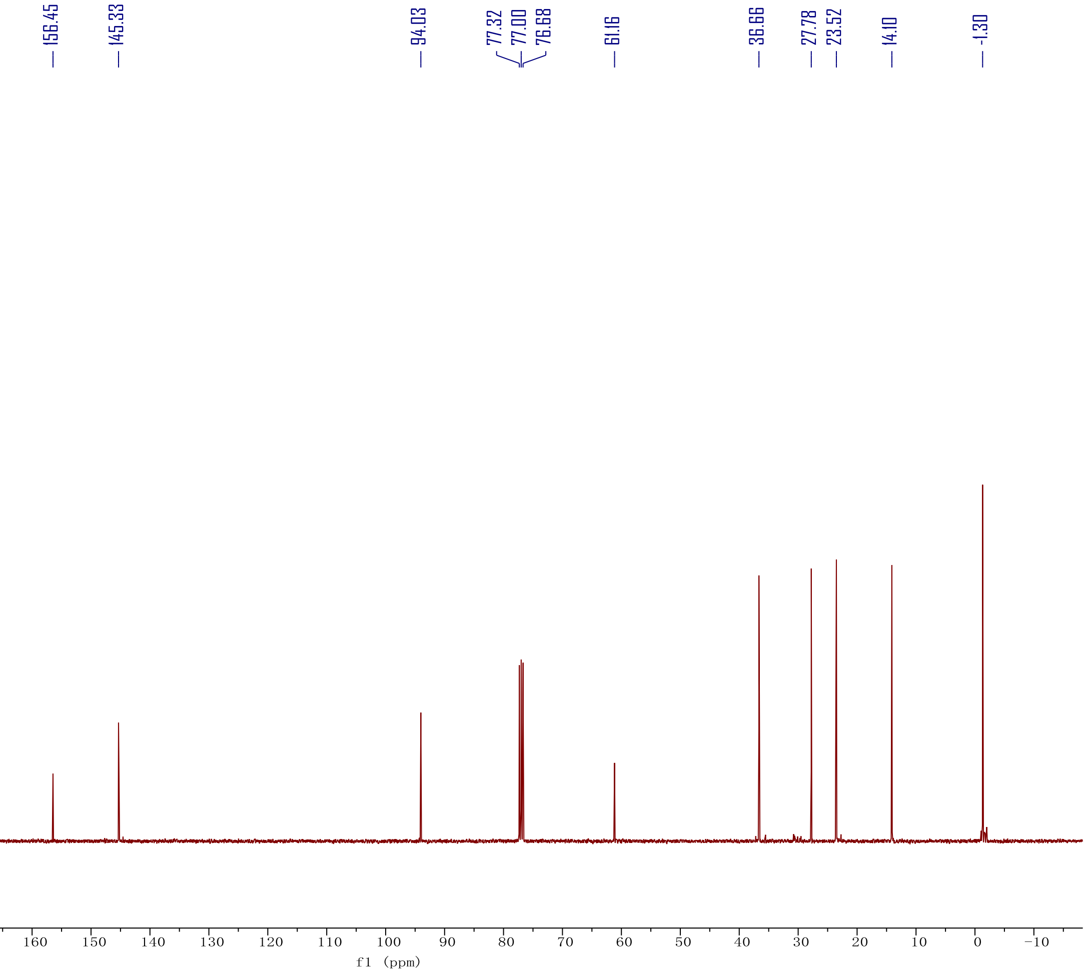

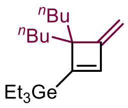

3d

^{1}H NMR (400 MHz, $CDCl_3$)

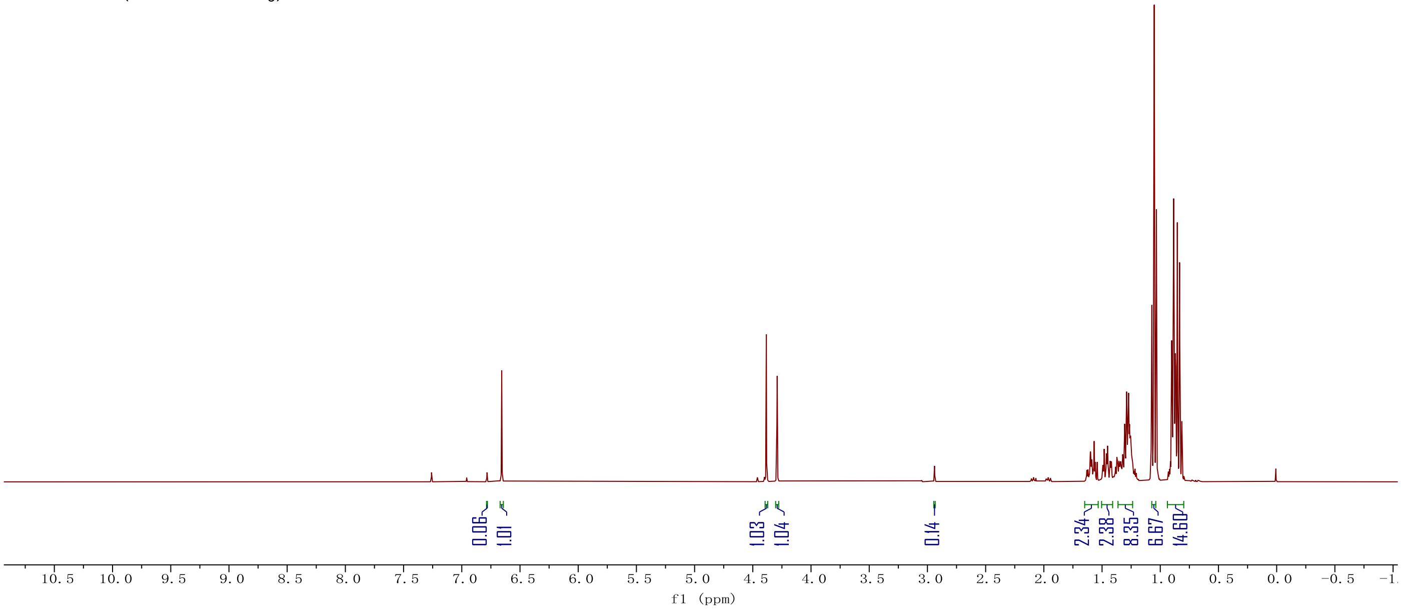

3d

^{13}C NMR (100 MHz, $CDCl_3$)

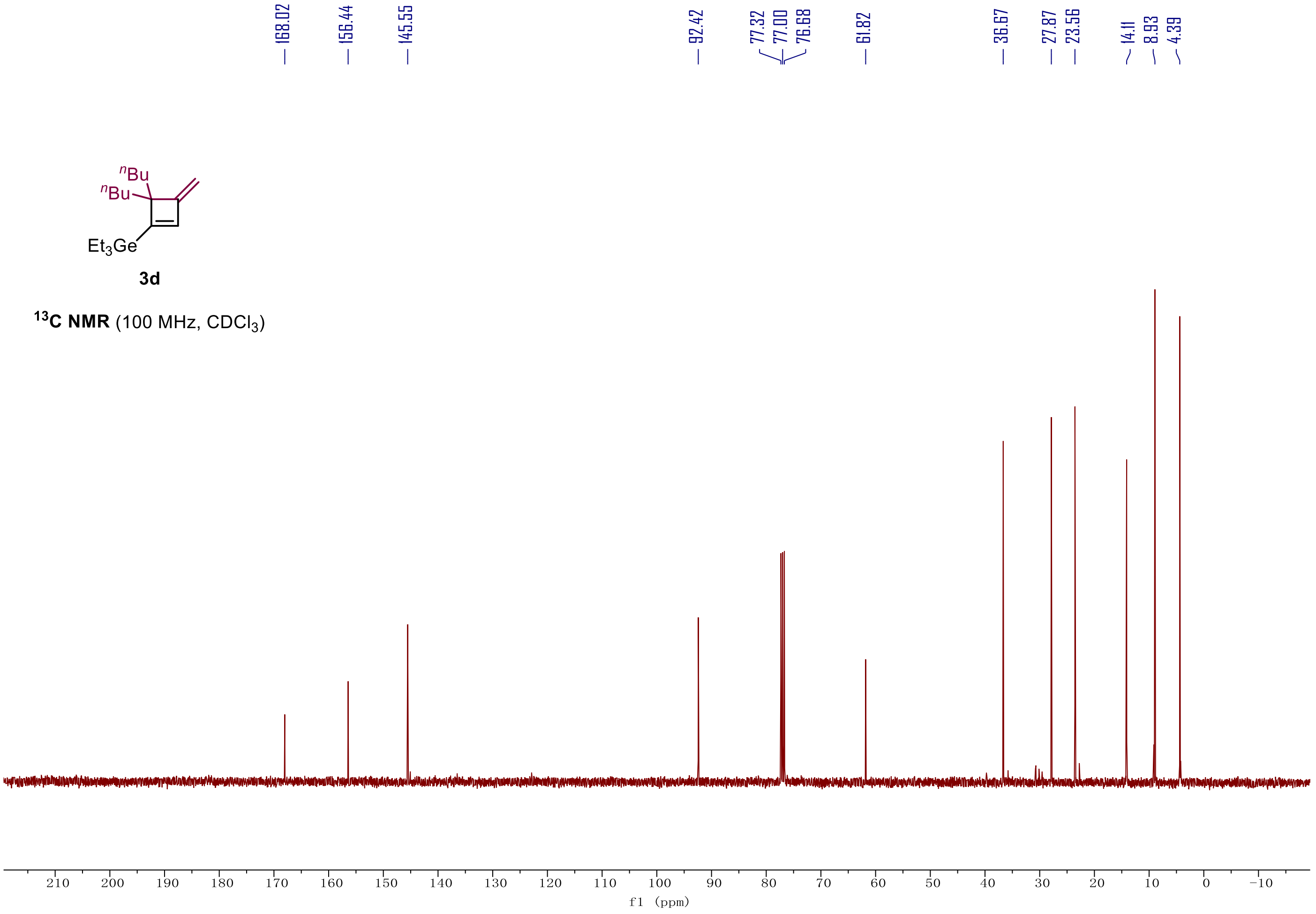

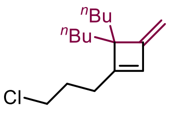

3e

^{1}H NMR (400 MHz, $CDCl_3$)

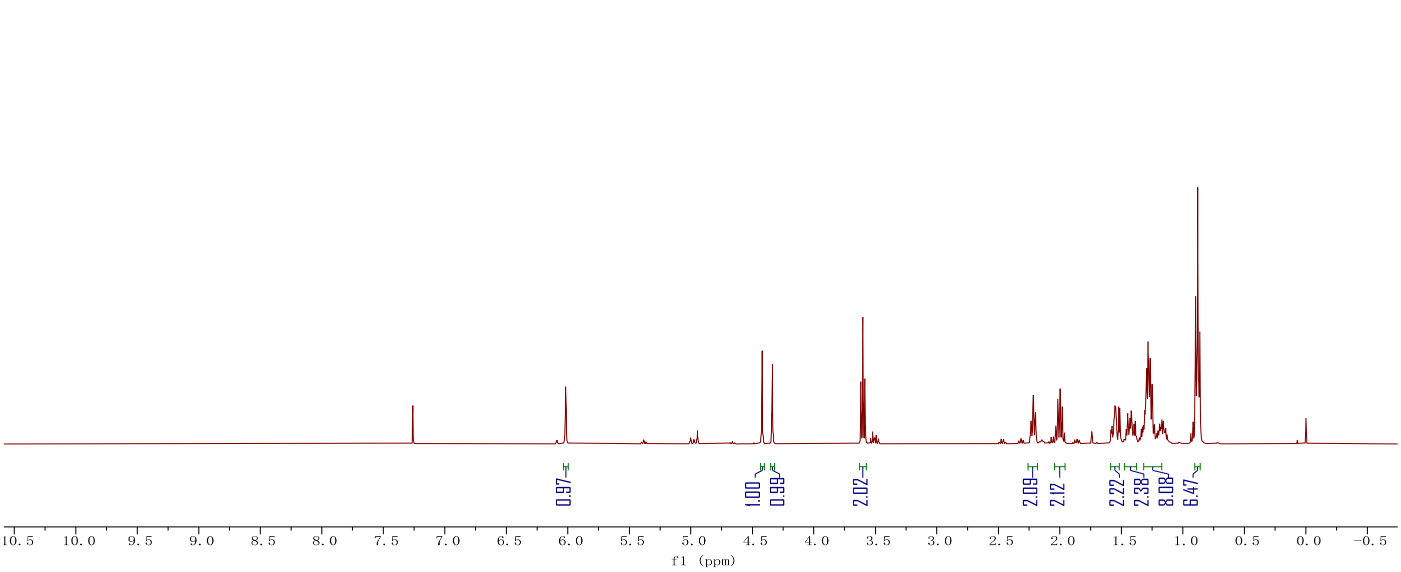

Impurities are not isomers

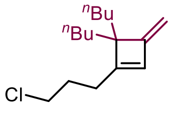

3e

^{13}C NMR (100 MHz, $CDCl_3$)

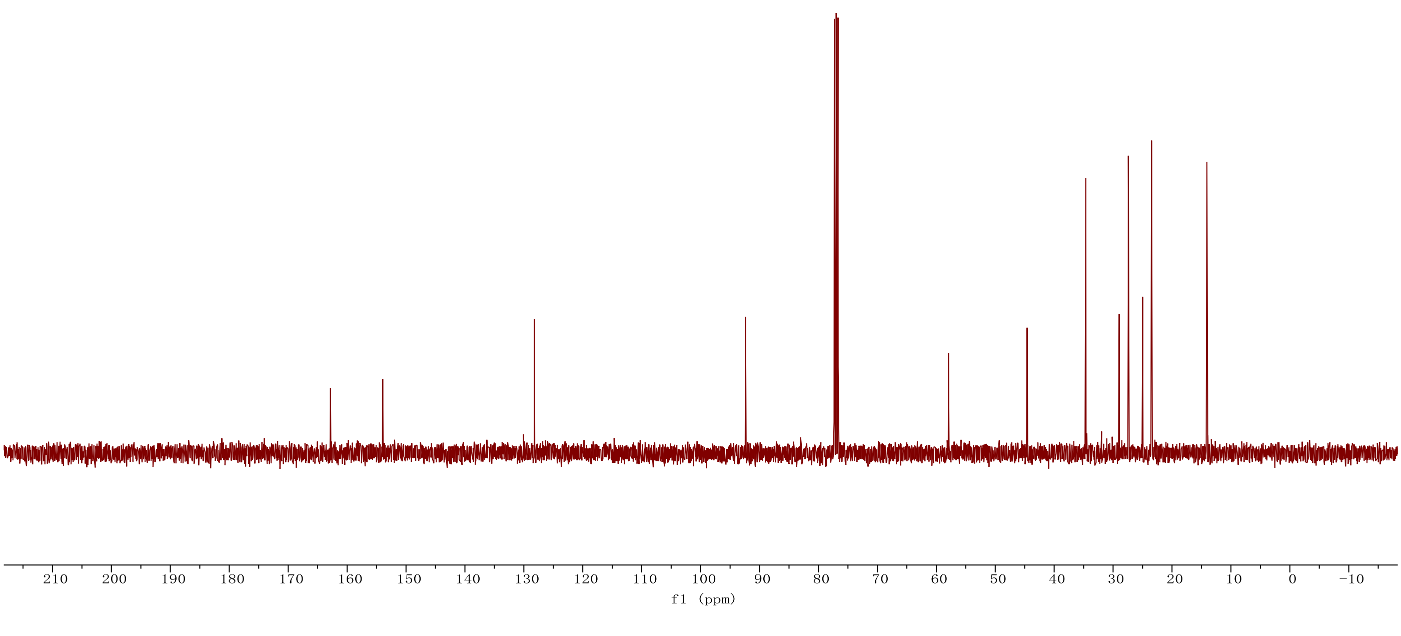

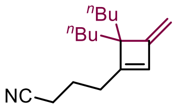

3f

^{1}H NMR (400 MHz, $CDCl_3$)

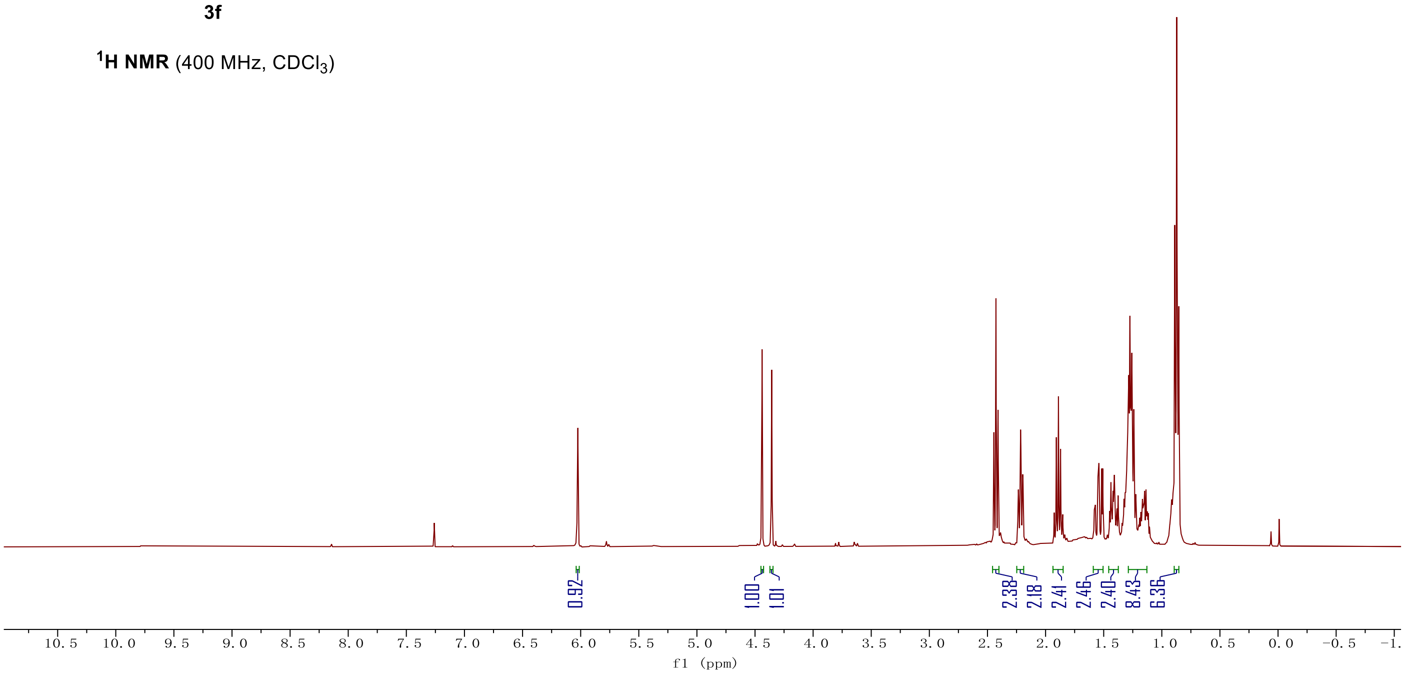

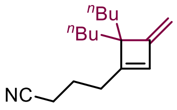

3f

^{13}C NMR (100 MHz, $CDCl_3$)

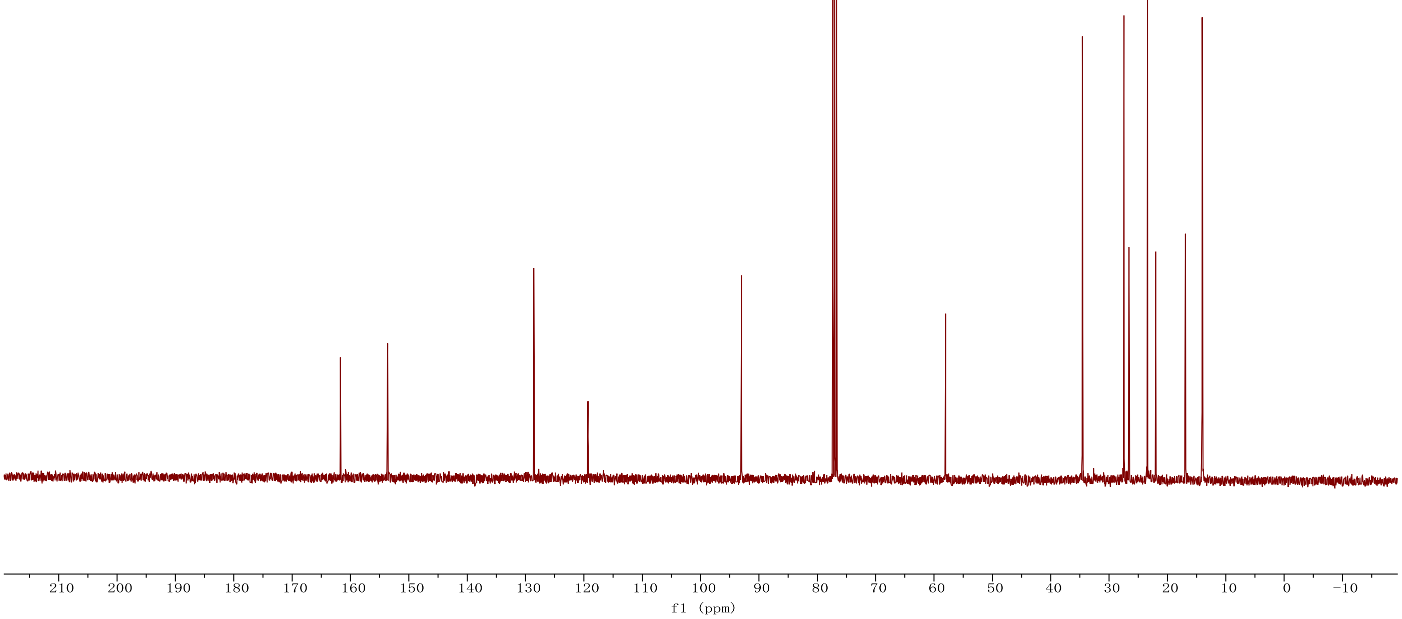

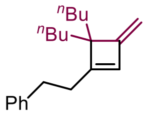

3g

^{1}H NMR (400 MHz, $CDCl_3$)

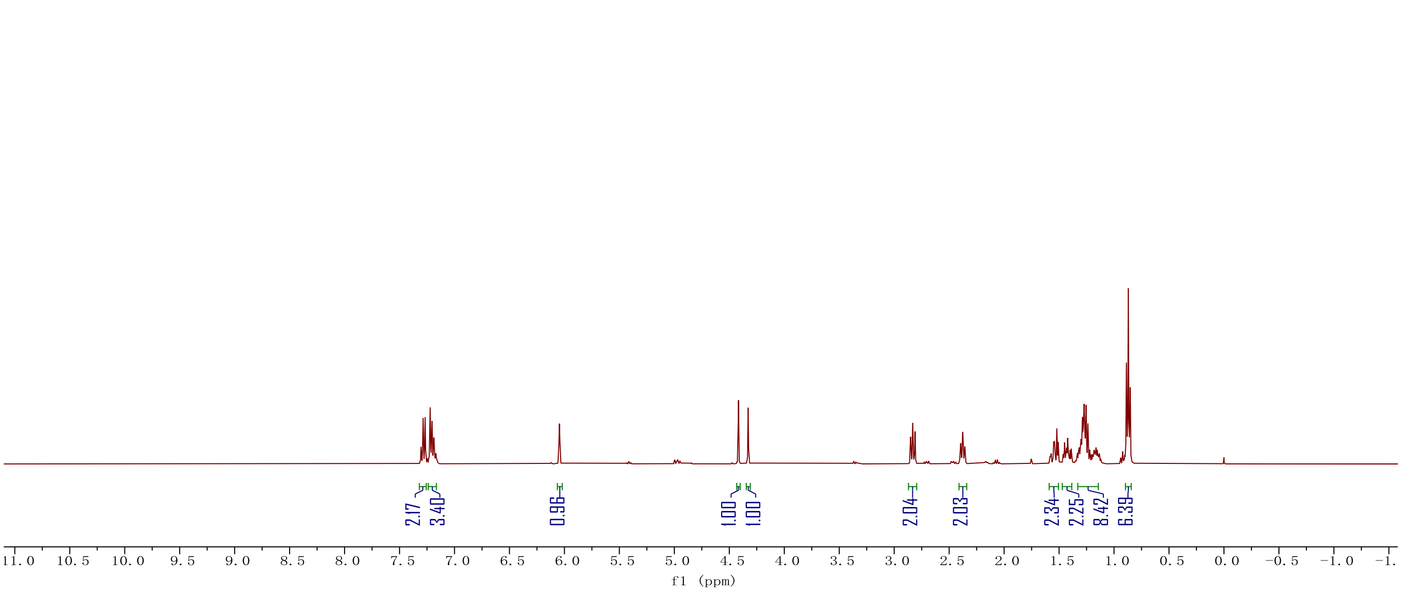

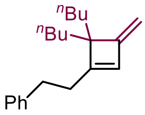

3g

^{13}C NMR (100 MHz, $CDCl_3$)

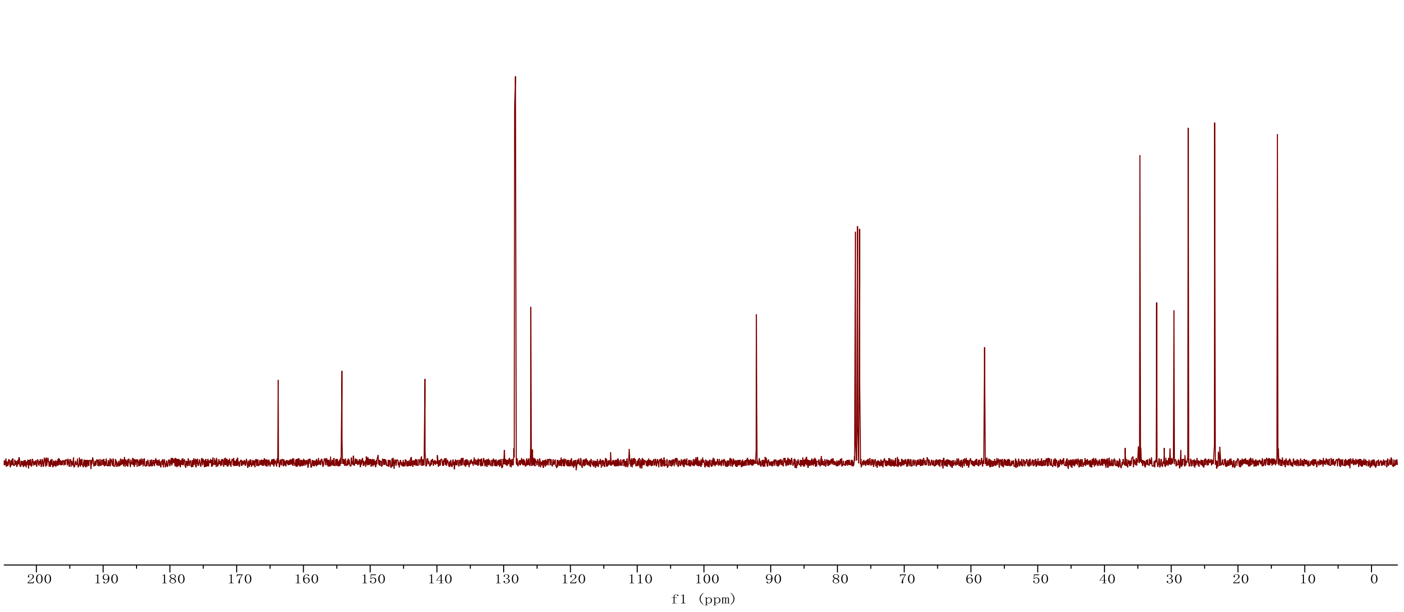

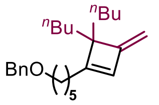

3h

^{1}H NMR (400 MHz, $CDCl_3$)

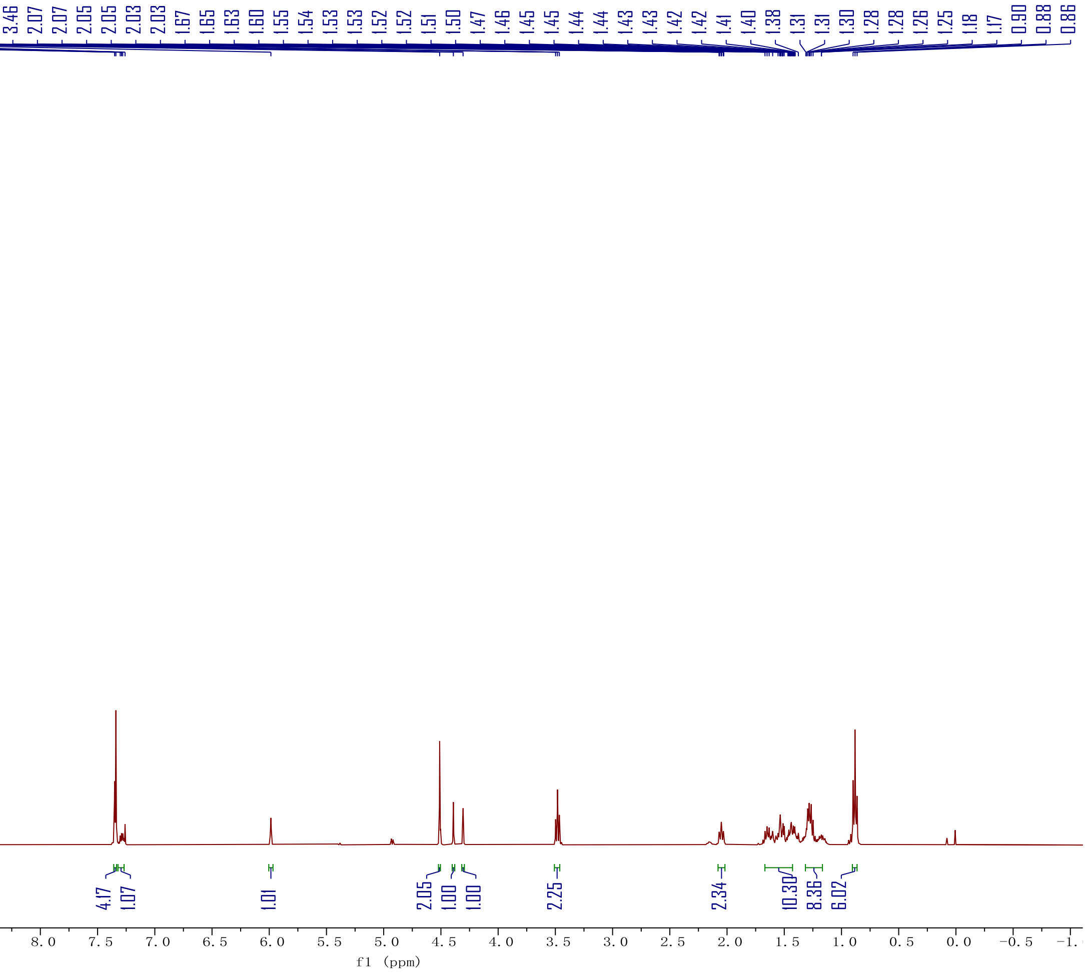

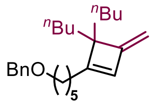

3h

^{13}C NMR (100 MHz, $CDCl_3$)

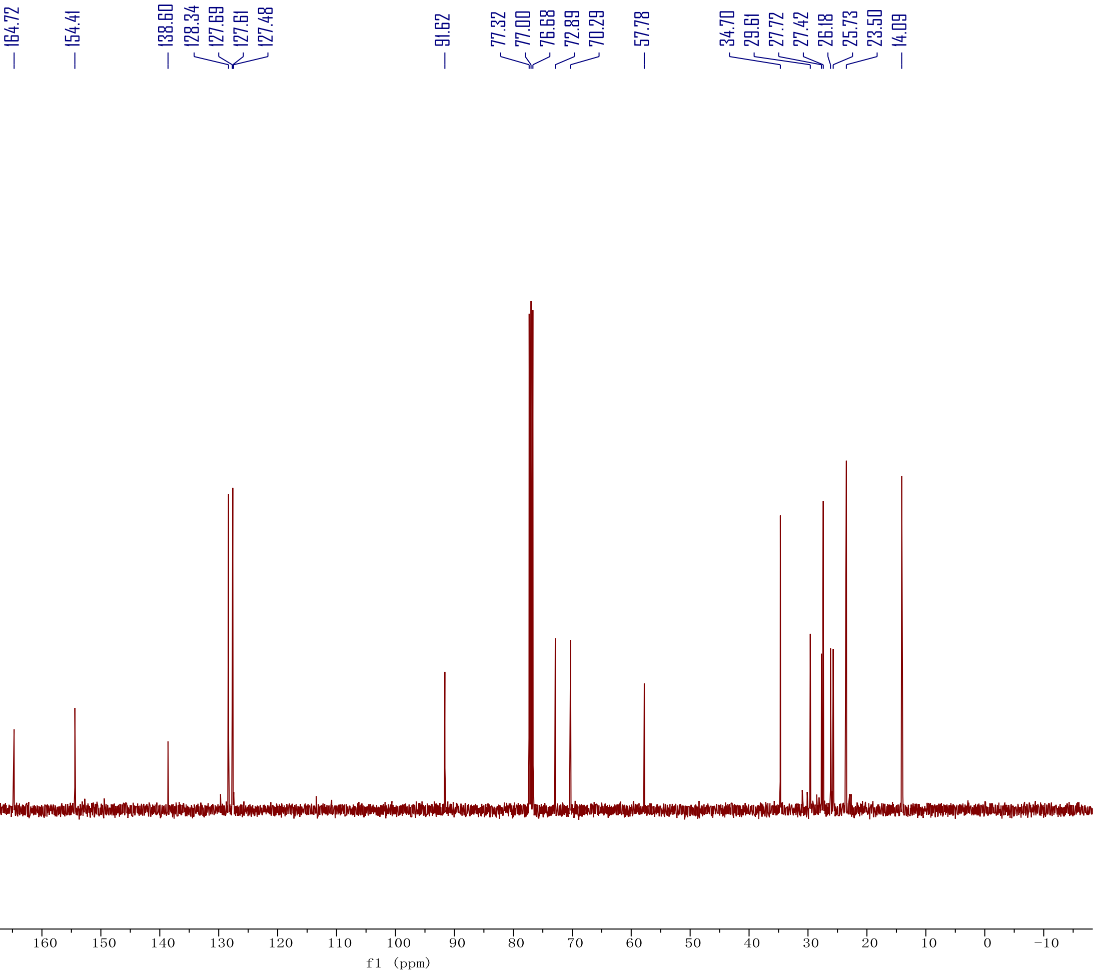

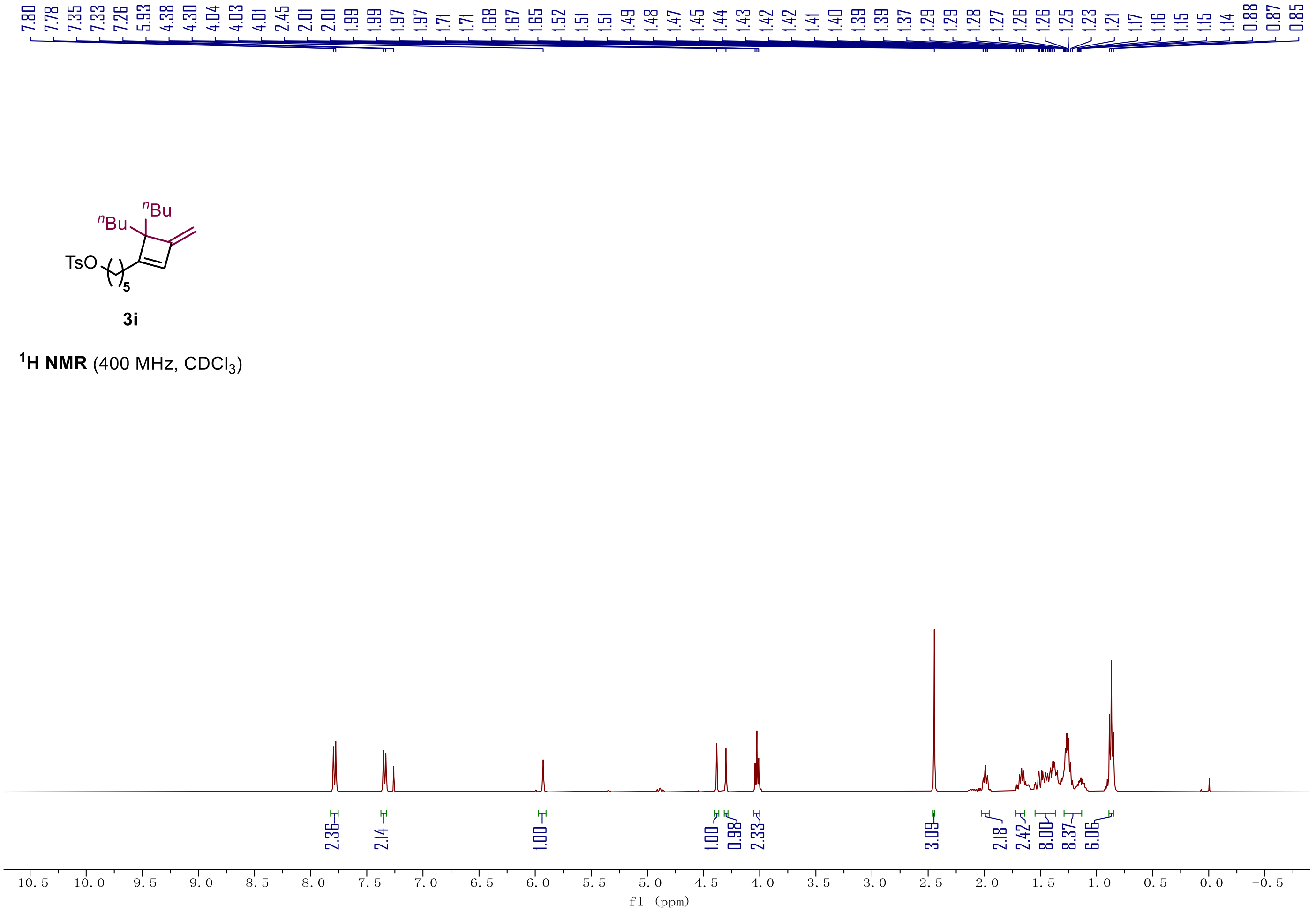

3i

^{1}H NMR (400 MHz, $CDCl_3$)

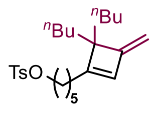

3i

^{13}C NMR (100 MHz, $CDCl_3$)

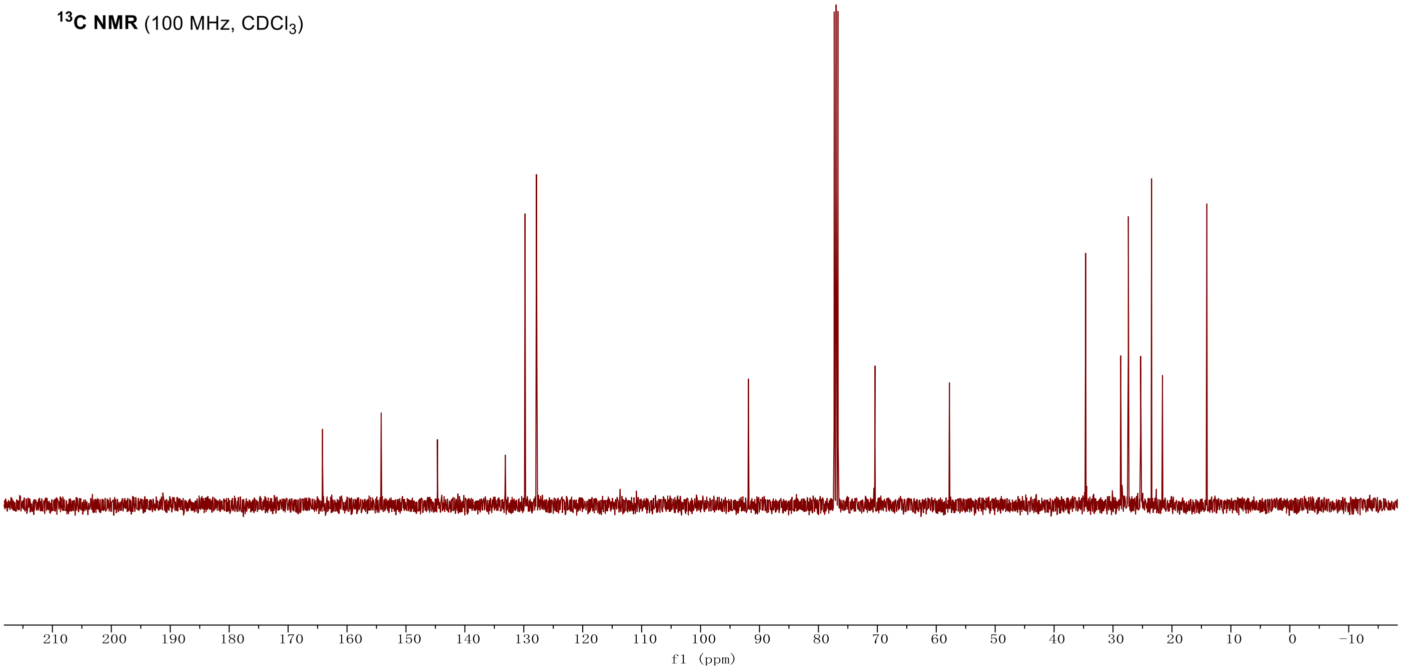

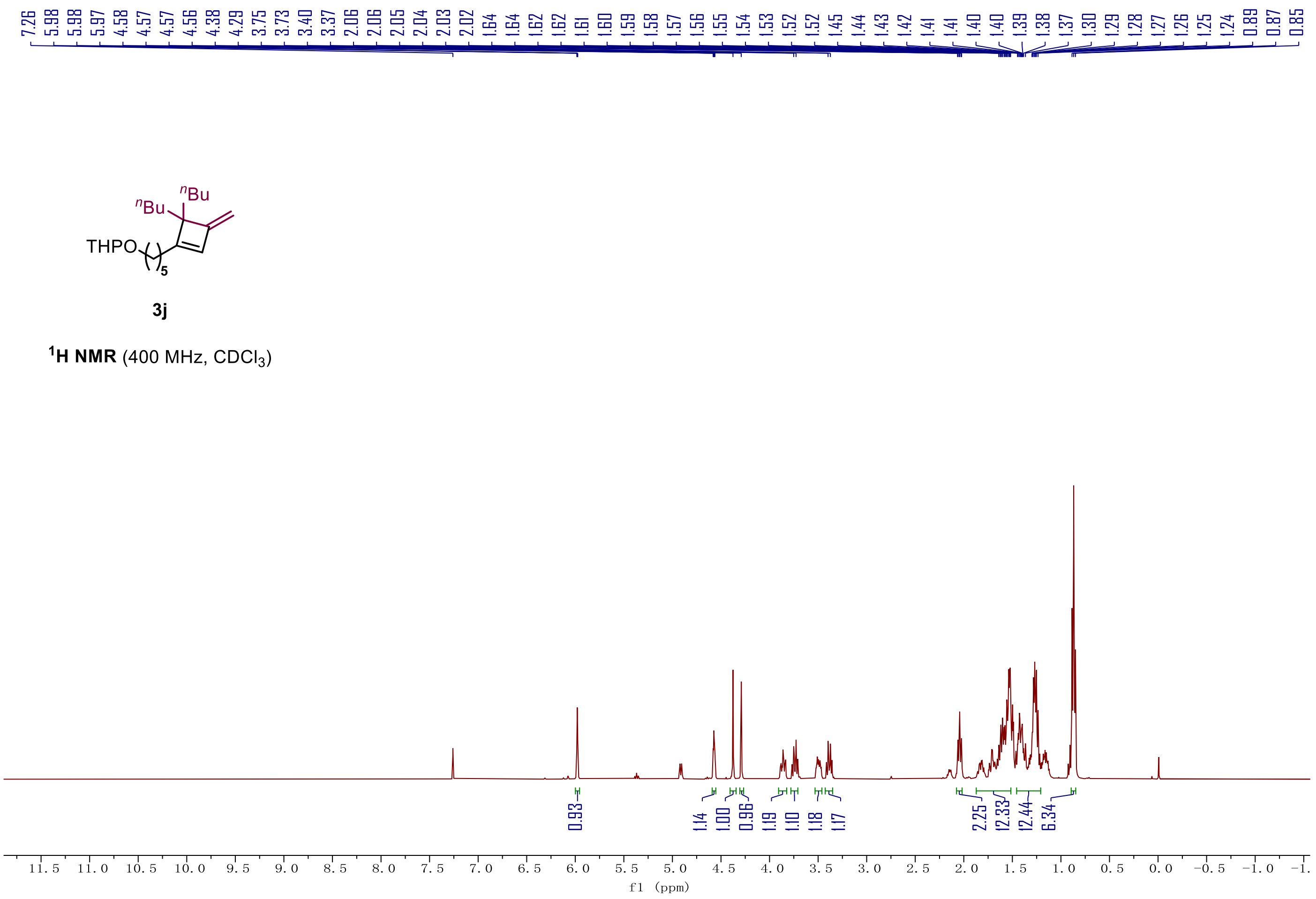

3j

^{1}H NMR (400 MHz, $CDCl_3$)

Impurities are not isomers

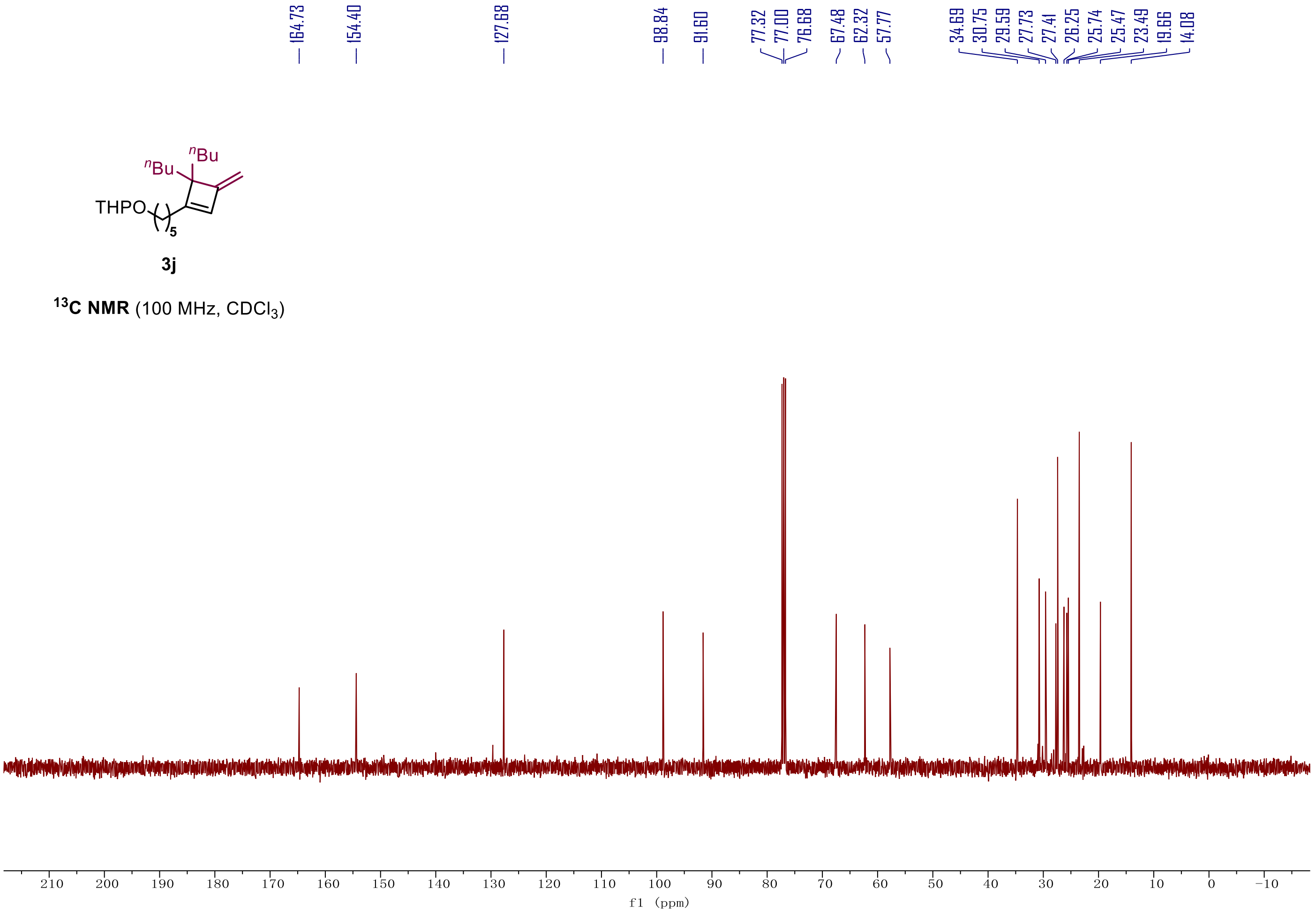

3j

^{13}C NMR (100 MHz, $CDCl_3$)

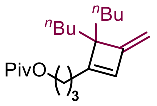

3k

^{1}H NMR (400 MHz, $CDCl_3$)

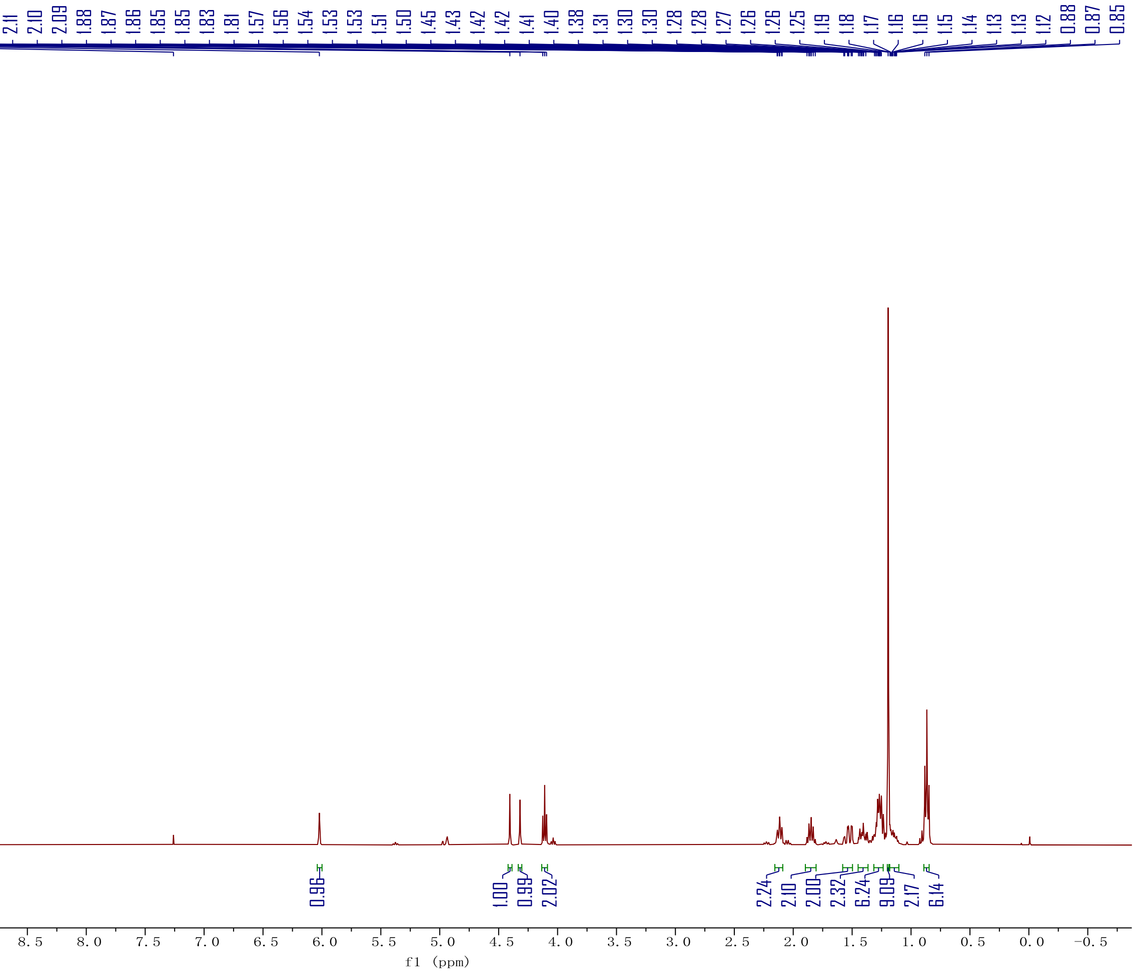

Impurities are not isomers

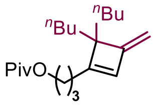

3k

^{13}C NMR (100 MHz, $CDCl_3$)

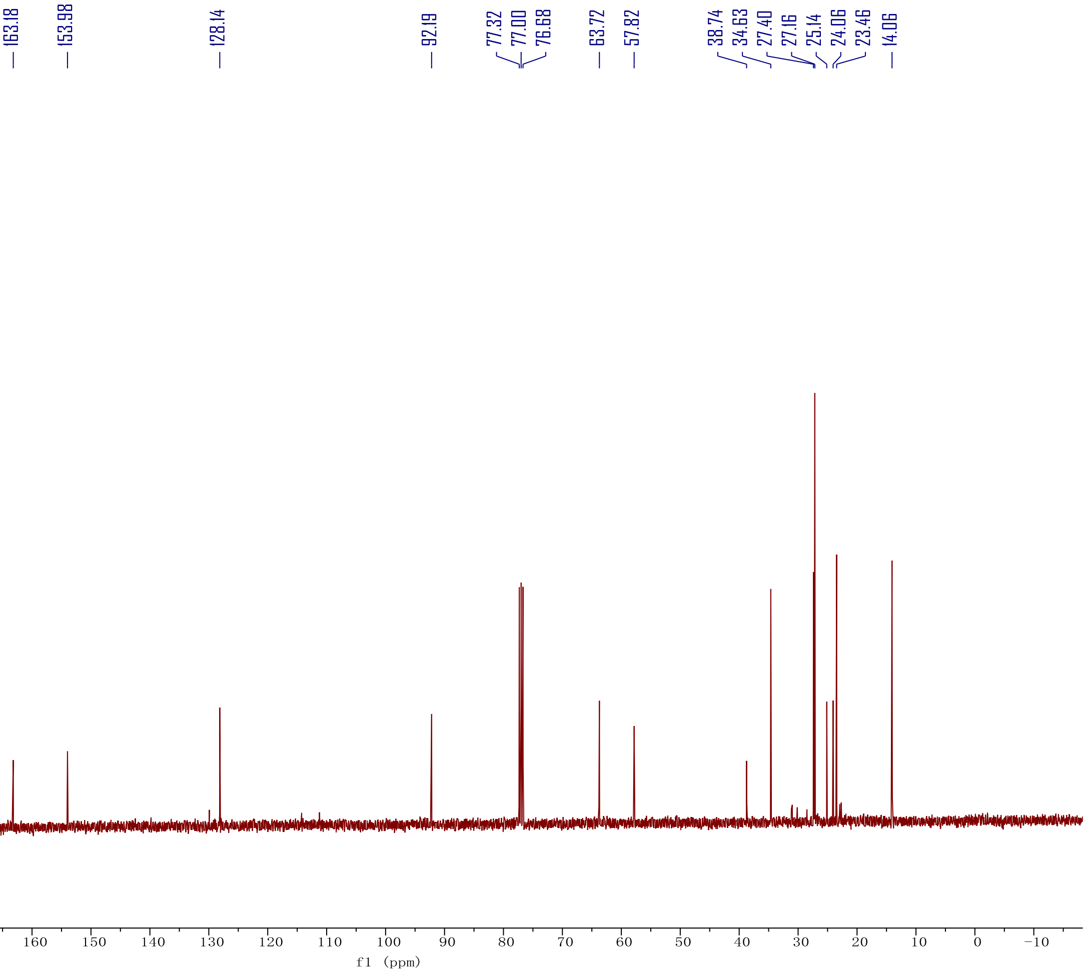

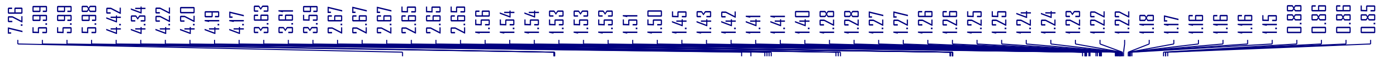

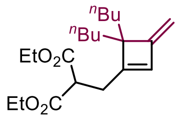

3l

^{1}H NMR (400 MHz, $CDCl_3$)

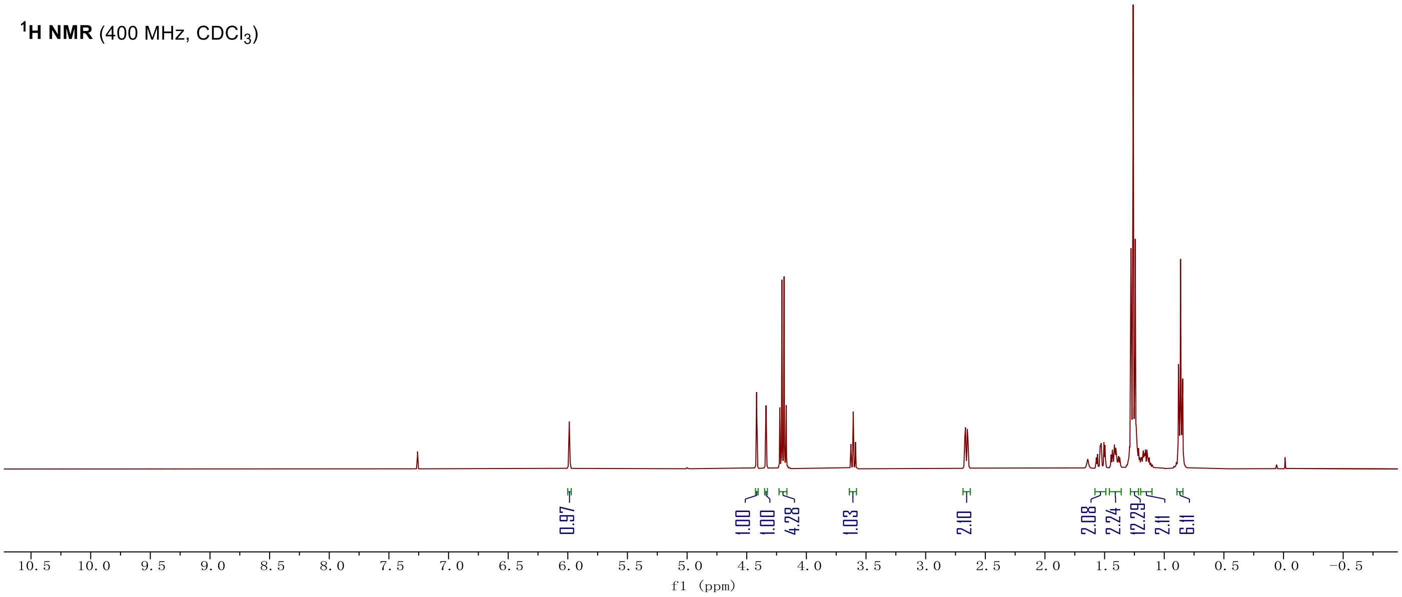

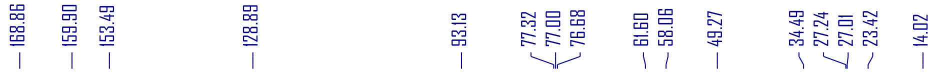

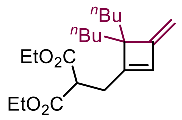

3l

^{13}C NMR (100 MHz, $CDCl_3$)

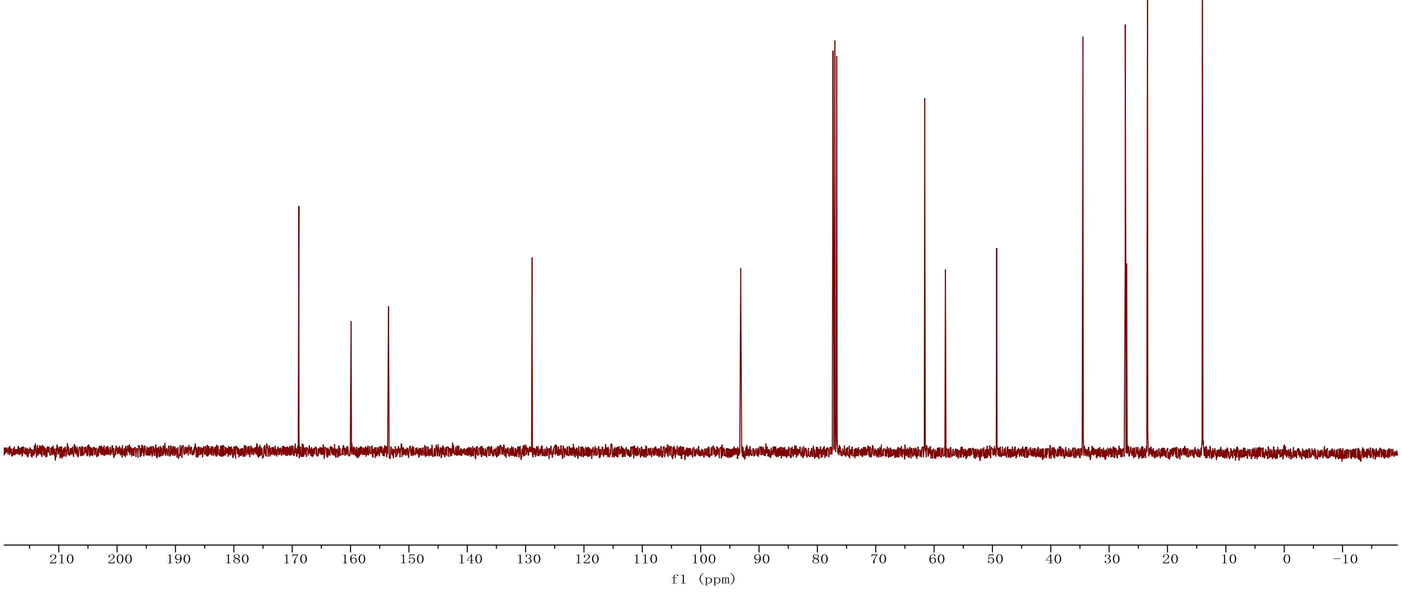

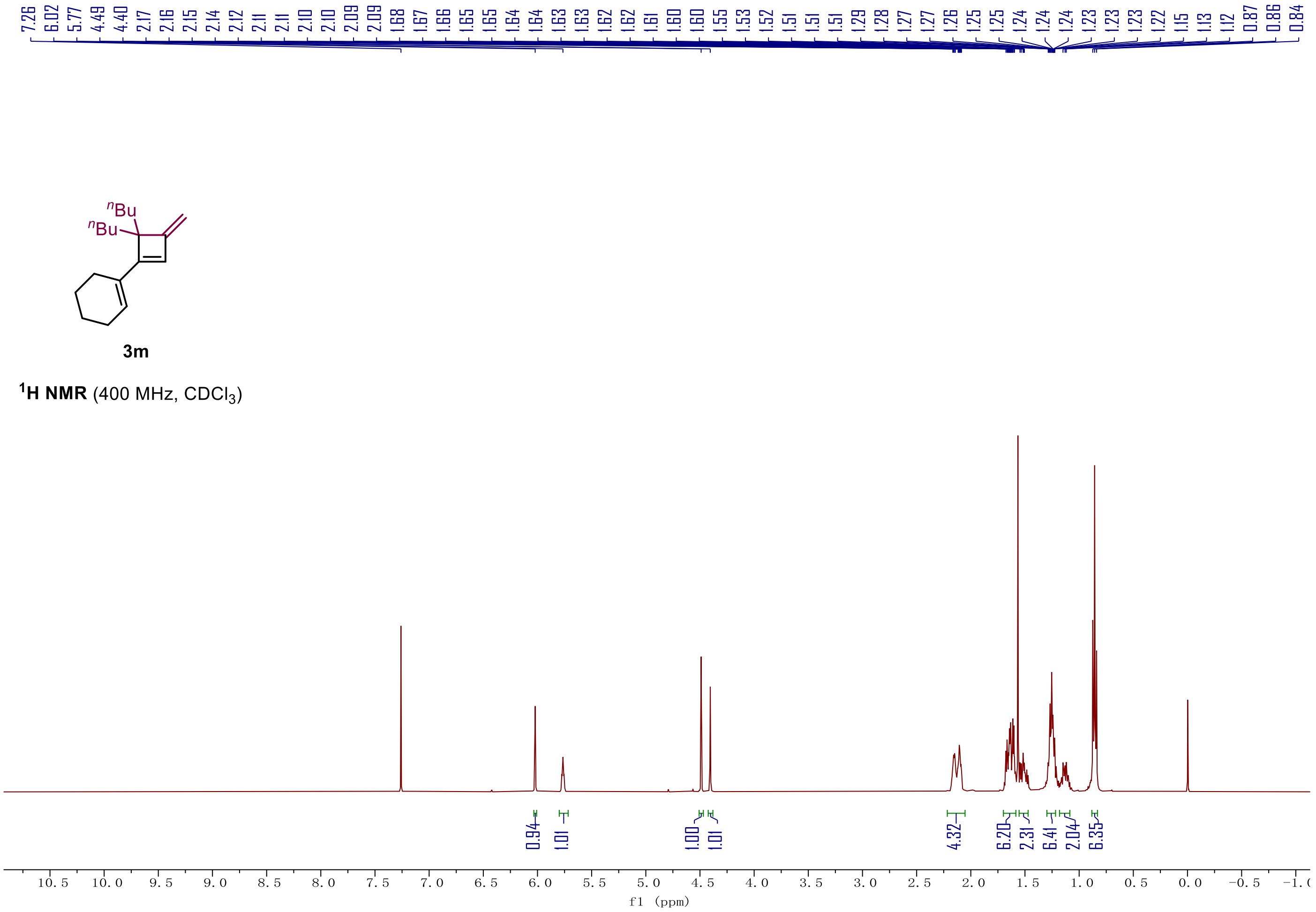

3m

1H NMR (400 MHz, $CDCl_3$)

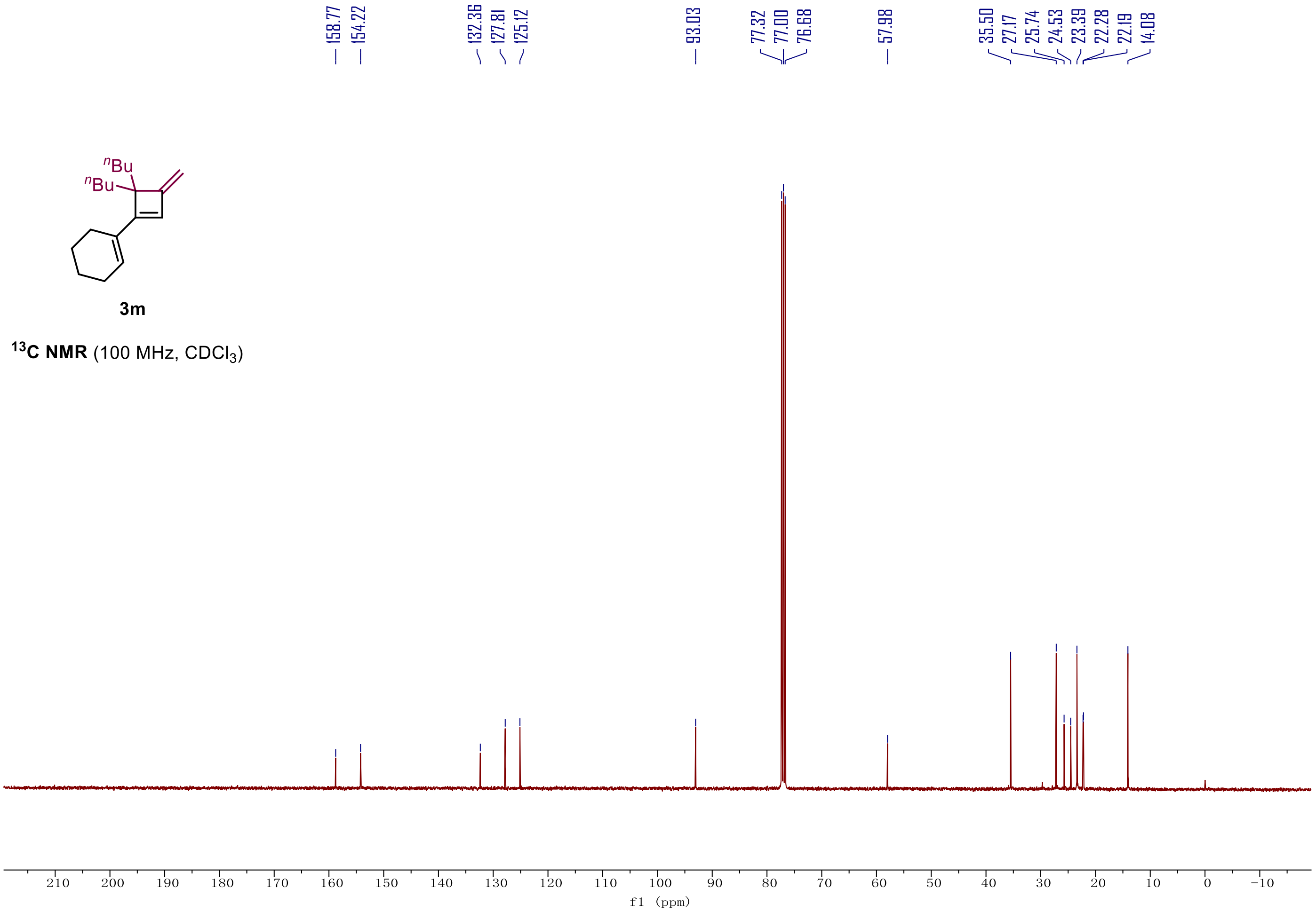

3m

13C NMR (100 MHz, $CDCl_3$)

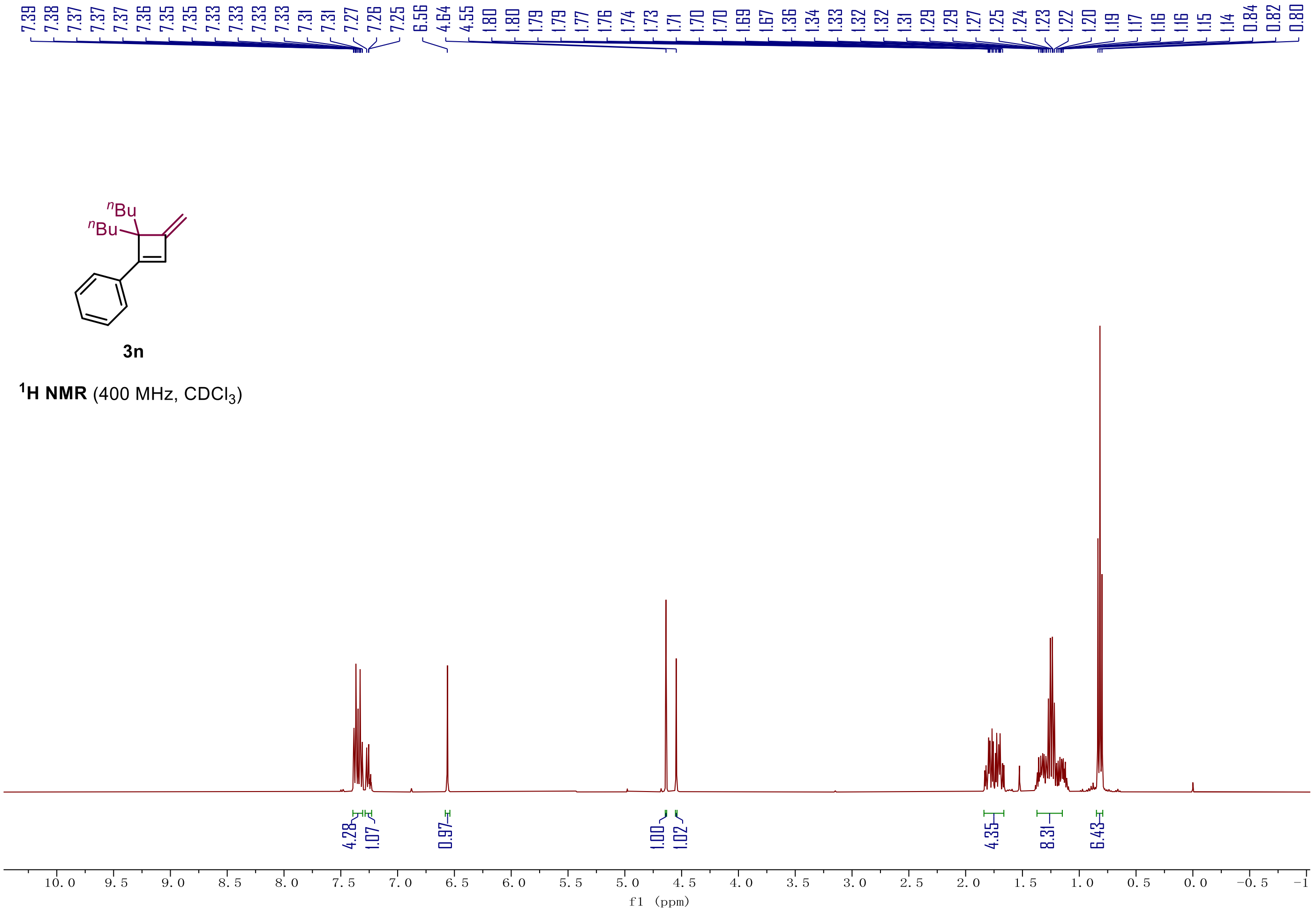

^{1}H NMR (400 MHz, CDCl$_3$)

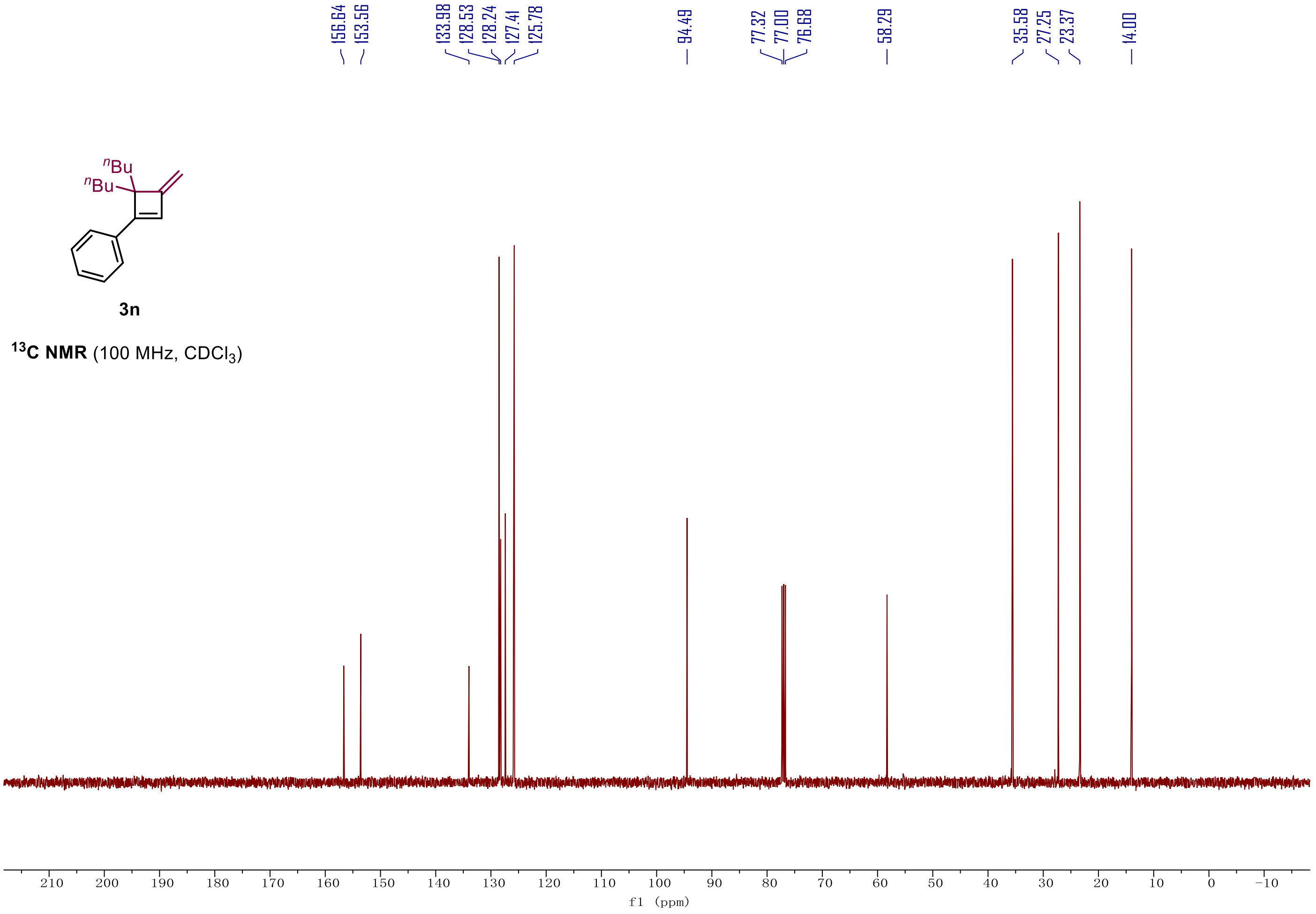

^{13}C NMR (100 MHz, CDCl$_3$)

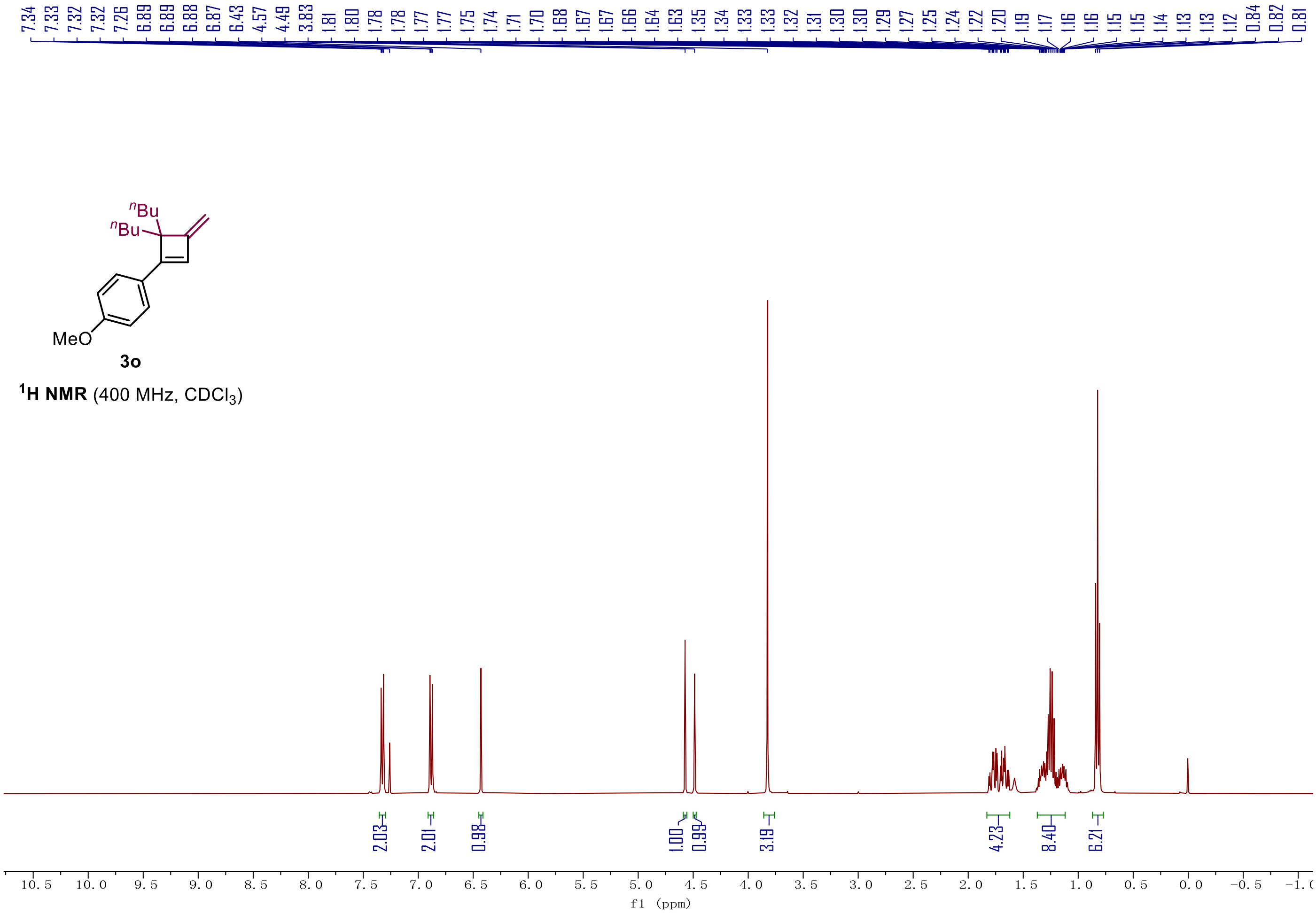

3o

^{1}H NMR (400 MHz, $CDCl_3$)

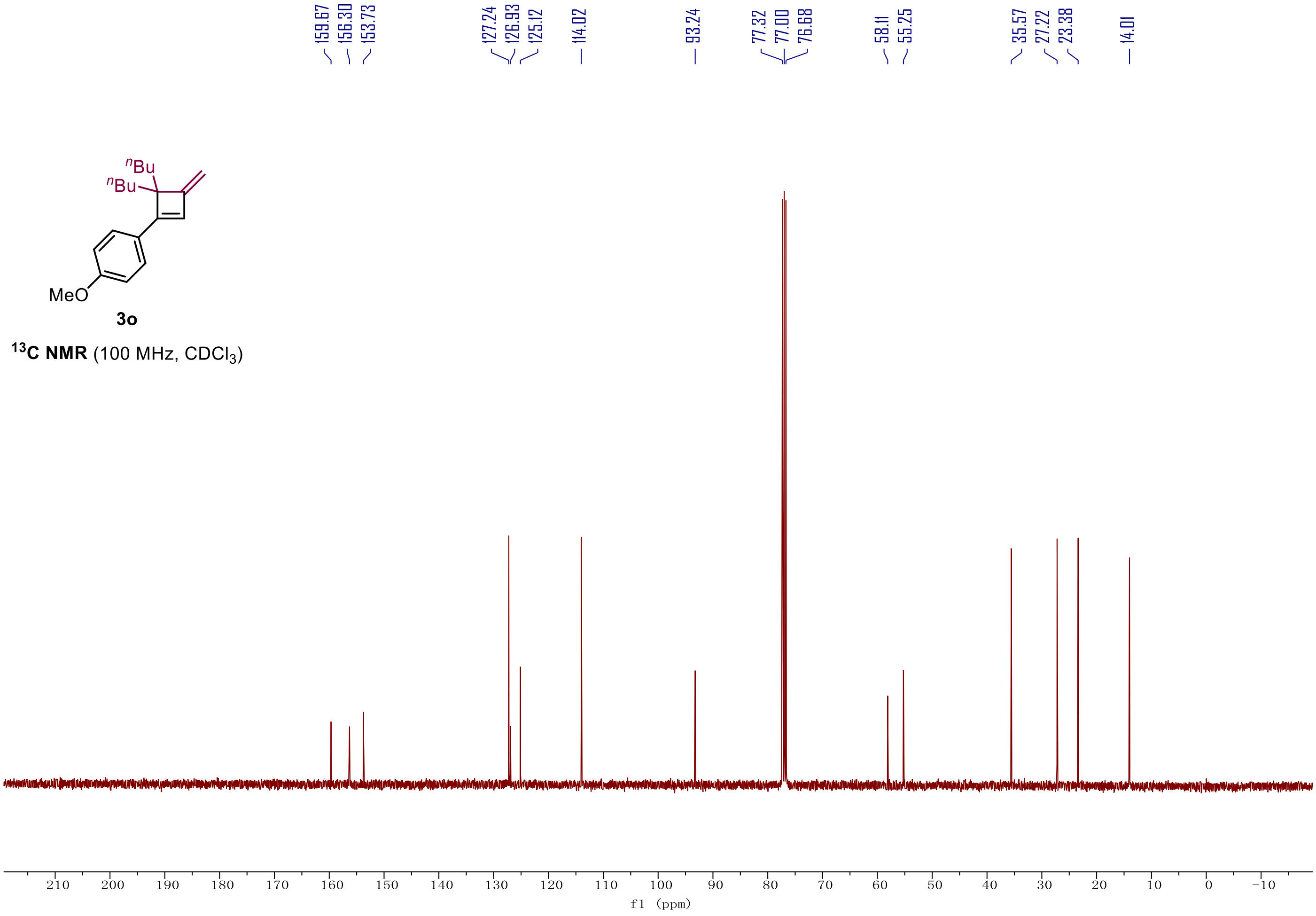

3o

^{13}C NMR (100 MHz, $CDCl_3$)

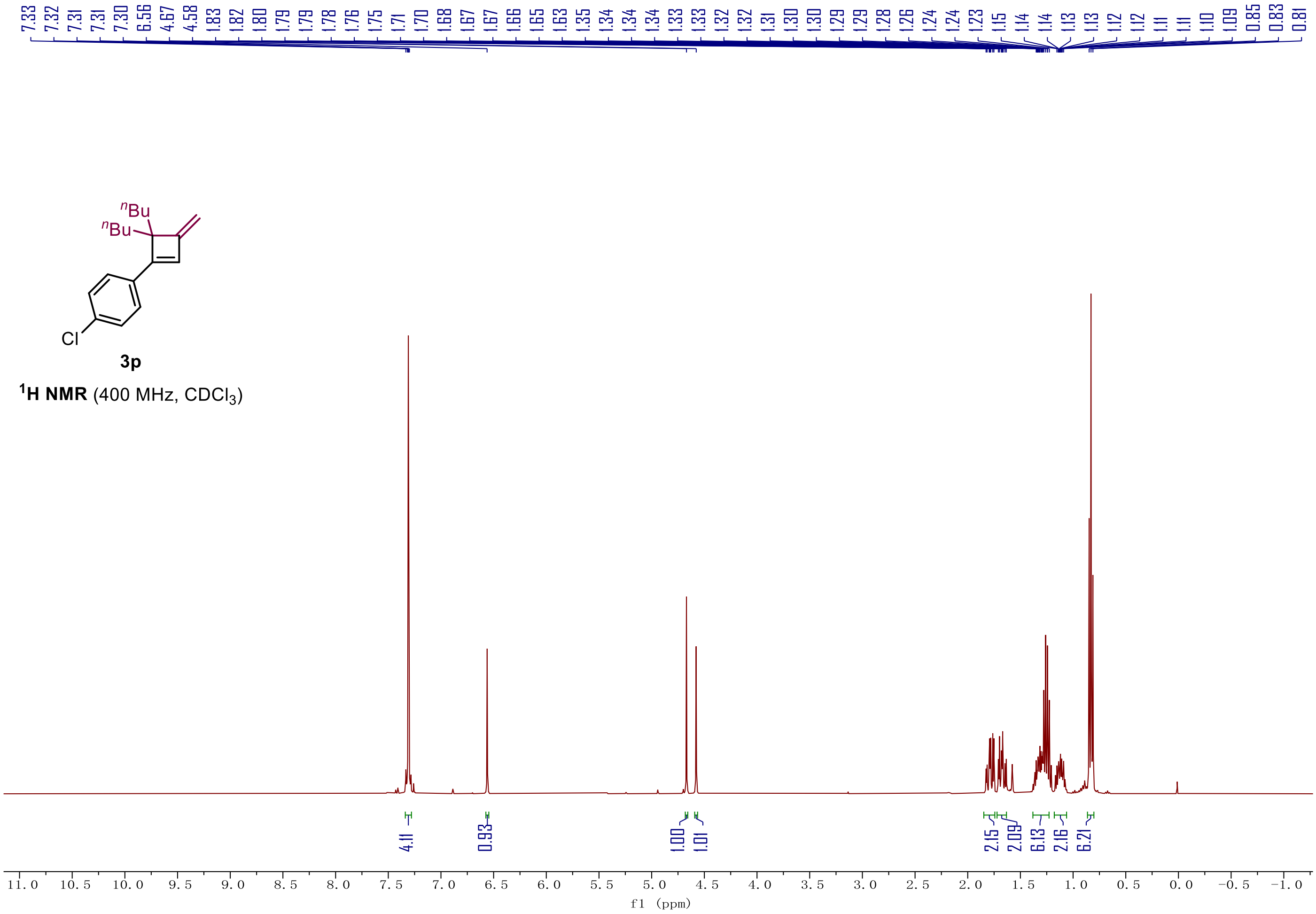

3p

^{1}H NMR (400 MHz, $CDCl_3$)

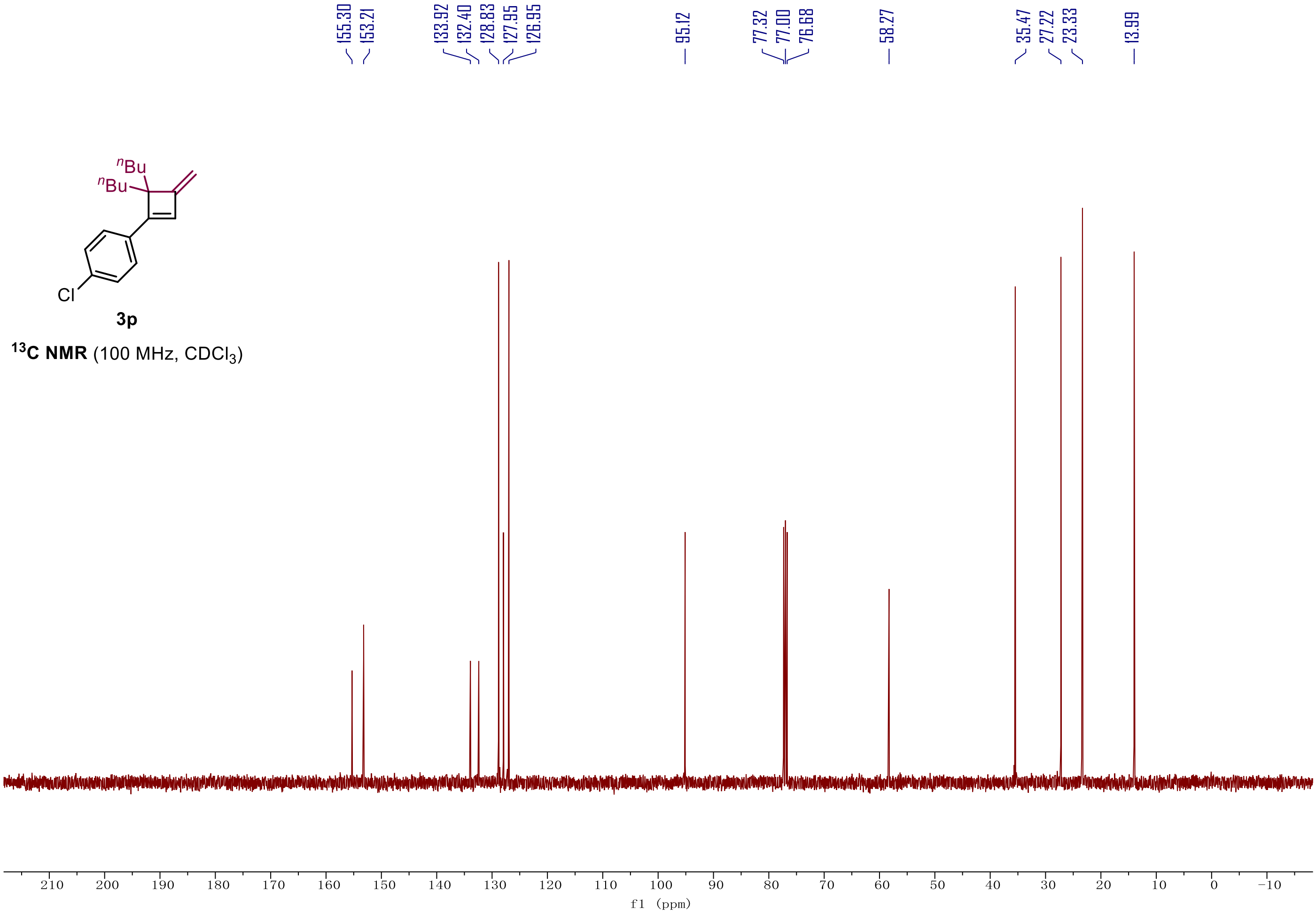

3p

^{13}C NMR (100 MHz, $CDCl_3$)

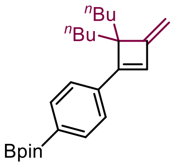

3q

^{1}H NMR (400 MHz, $CDCl_3$)

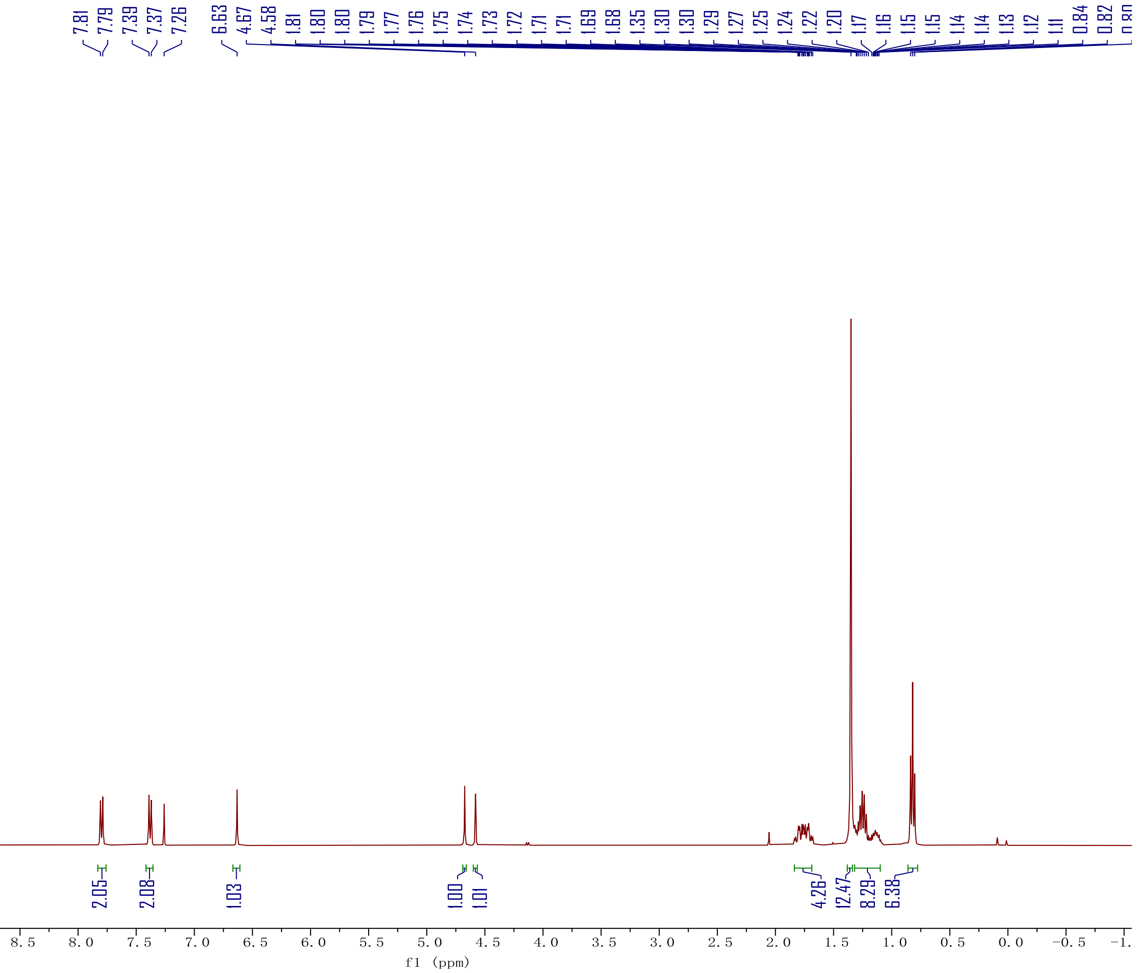

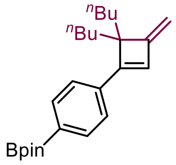

3q

^{13}C NMR (100 MHz, $CDCl_3$)

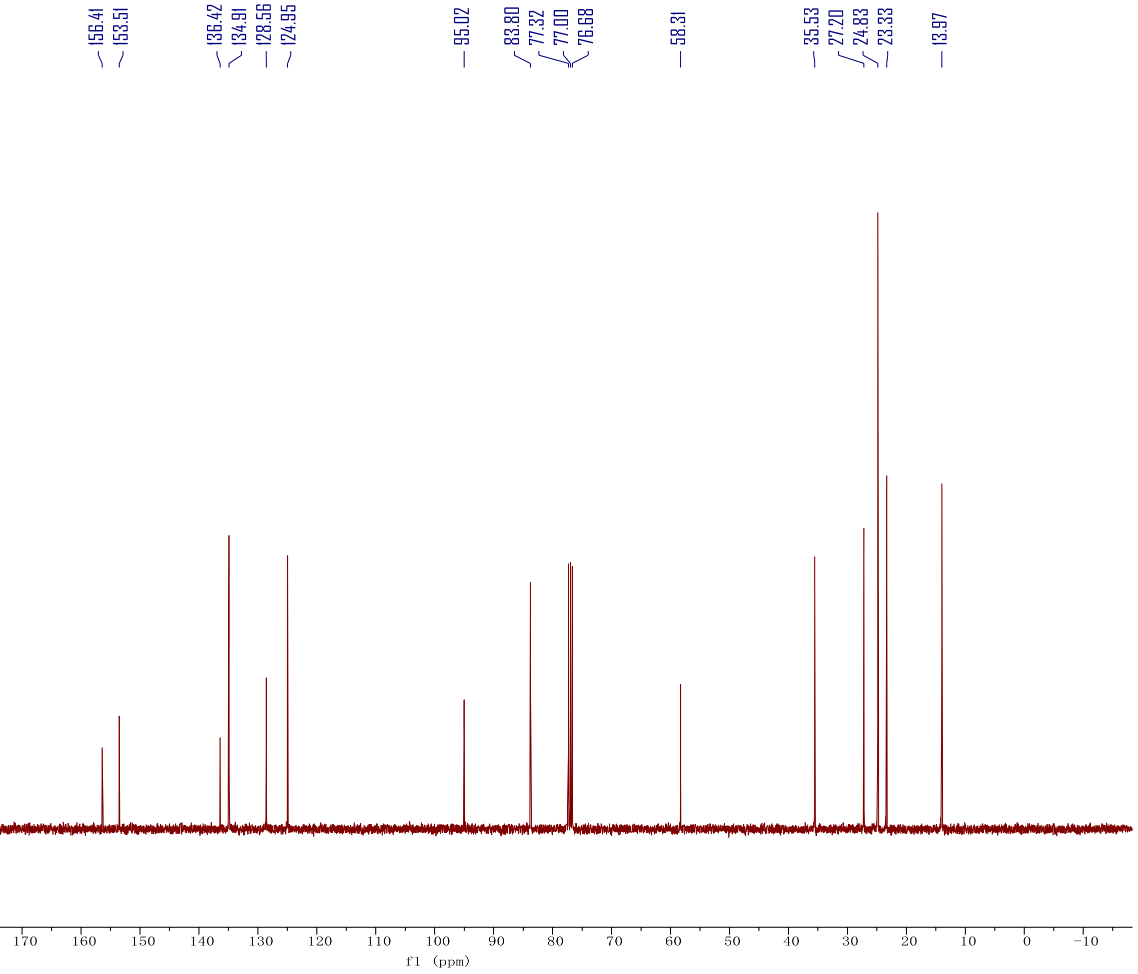

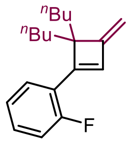

3r

^{1}H NMR (400 MHz, $CDCl_3$)

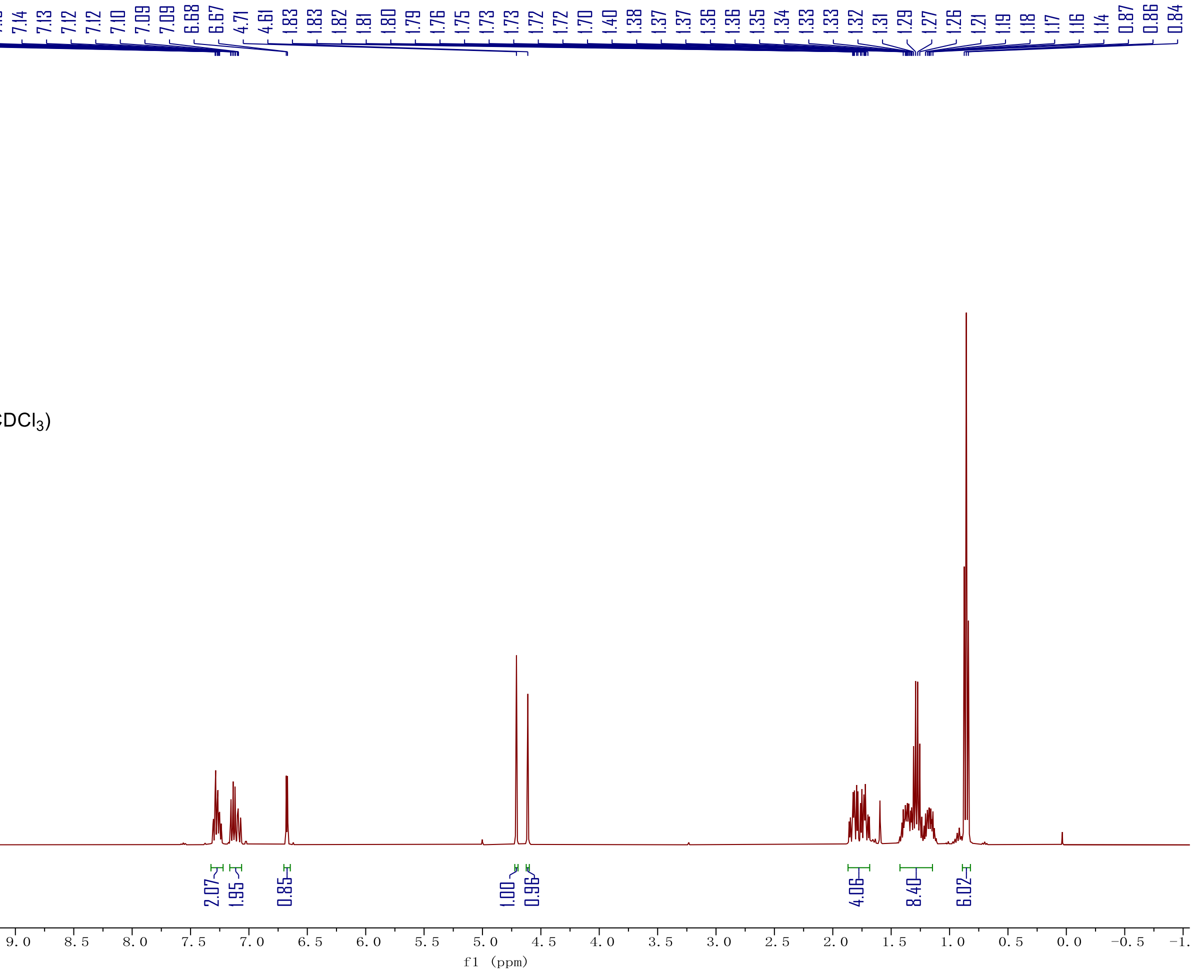

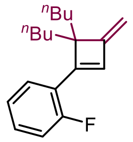

3r

^{13}C NMR (100 MHz, $CDCl_3$)

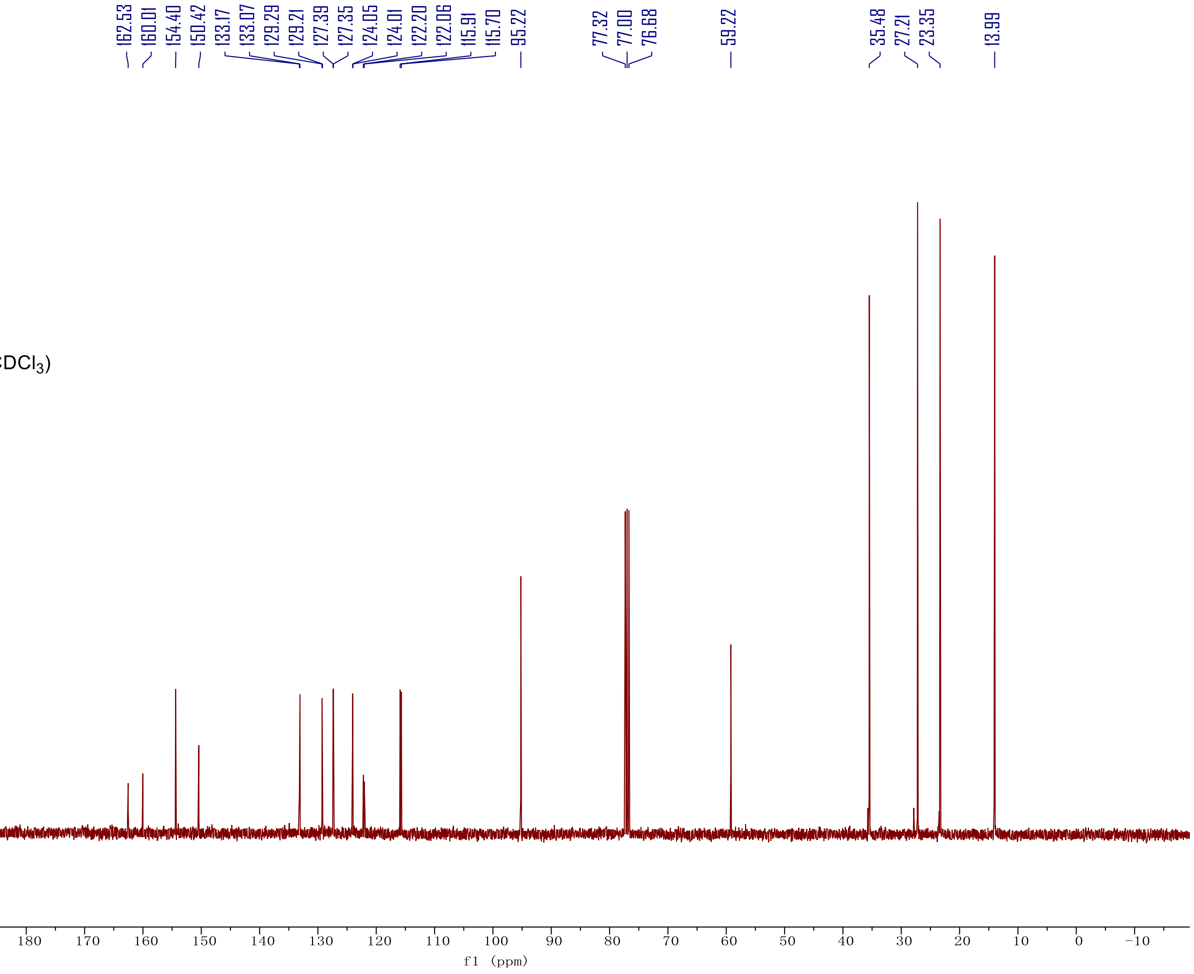

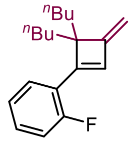

3r

^{19}F NMR (376 MHz, $CDCl_3$)

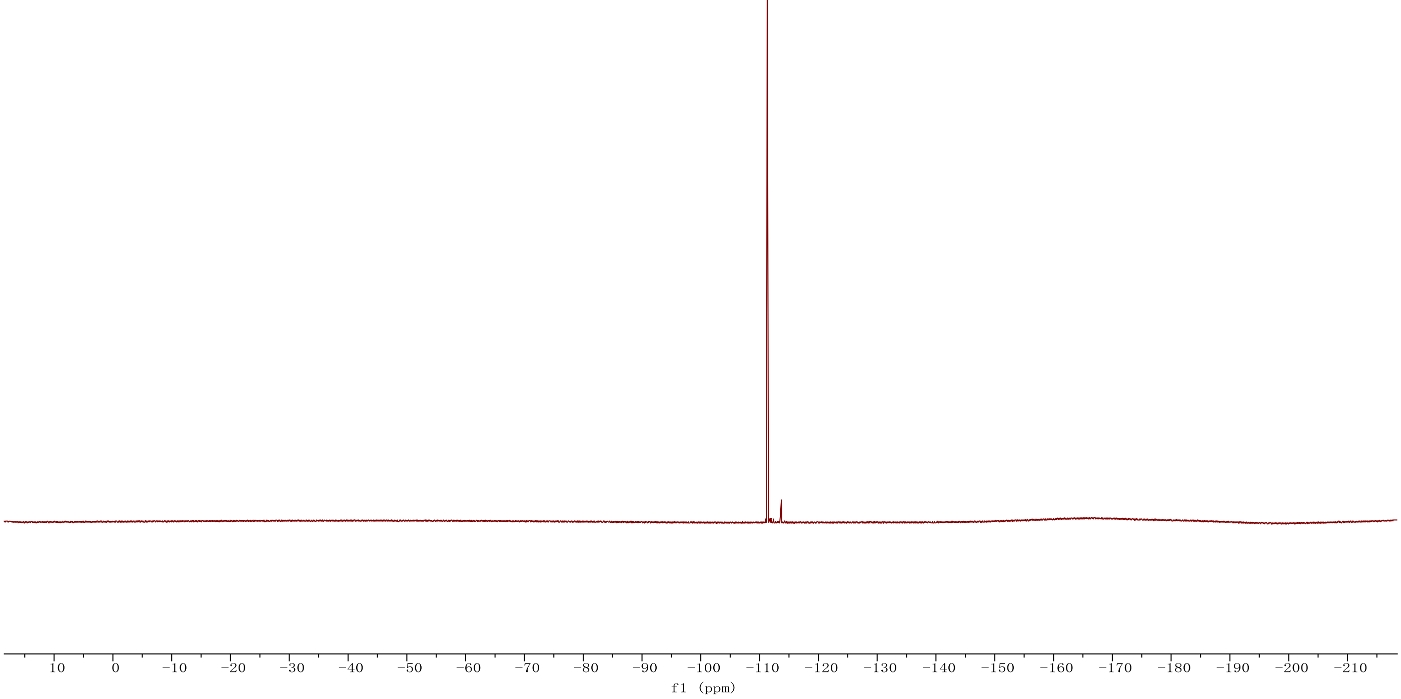

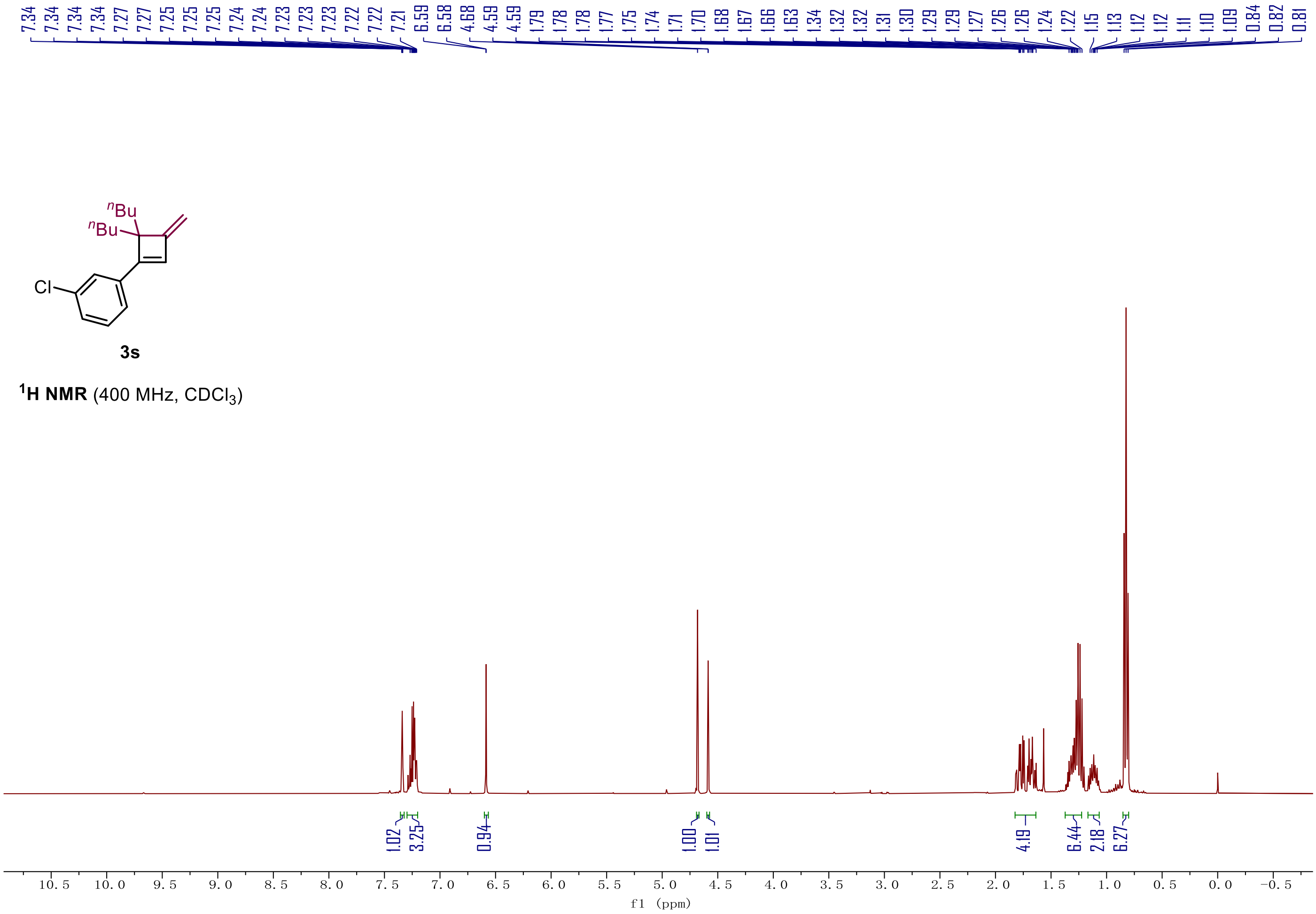
7.34 7.34 7.34 7.34 7.27 7.27 7.25 7.25 7.25 7.24 7.24 7.23 7.23 7.23 7.22 7.22 7.21 6.59 6.58 4.68 4.59 4.59 1.79 1.78 1.78 1.77 1.75 1.74 1.71 1.70 1.68 1.67 1.66 1.63 1.34 1.32 1.32 1.31 1.30 1.29 1.29 1.27 1.26 1.26 1.24 1.22 1.15 1.13 1.12 1.12 1.11 1.10 1.09 0.84 0.82 0.81
^{n}Bu
^{n}Bu
Cl
3s
^{1}H NMR (400 MHz, CDCl$_3$)
1.02 3.25 0.94 1.00 1.01 4.19 6.44 2.18 6.27
10.5 10.0 9.5 9.0 8.5 8.0 7.5 7.0 6.5 6.0 5.5 5.0 4.5 4.0 3.5 3.0 2.5 2.0 1.5 1.0 0.5 0.0 -0.5
f1 (ppm)

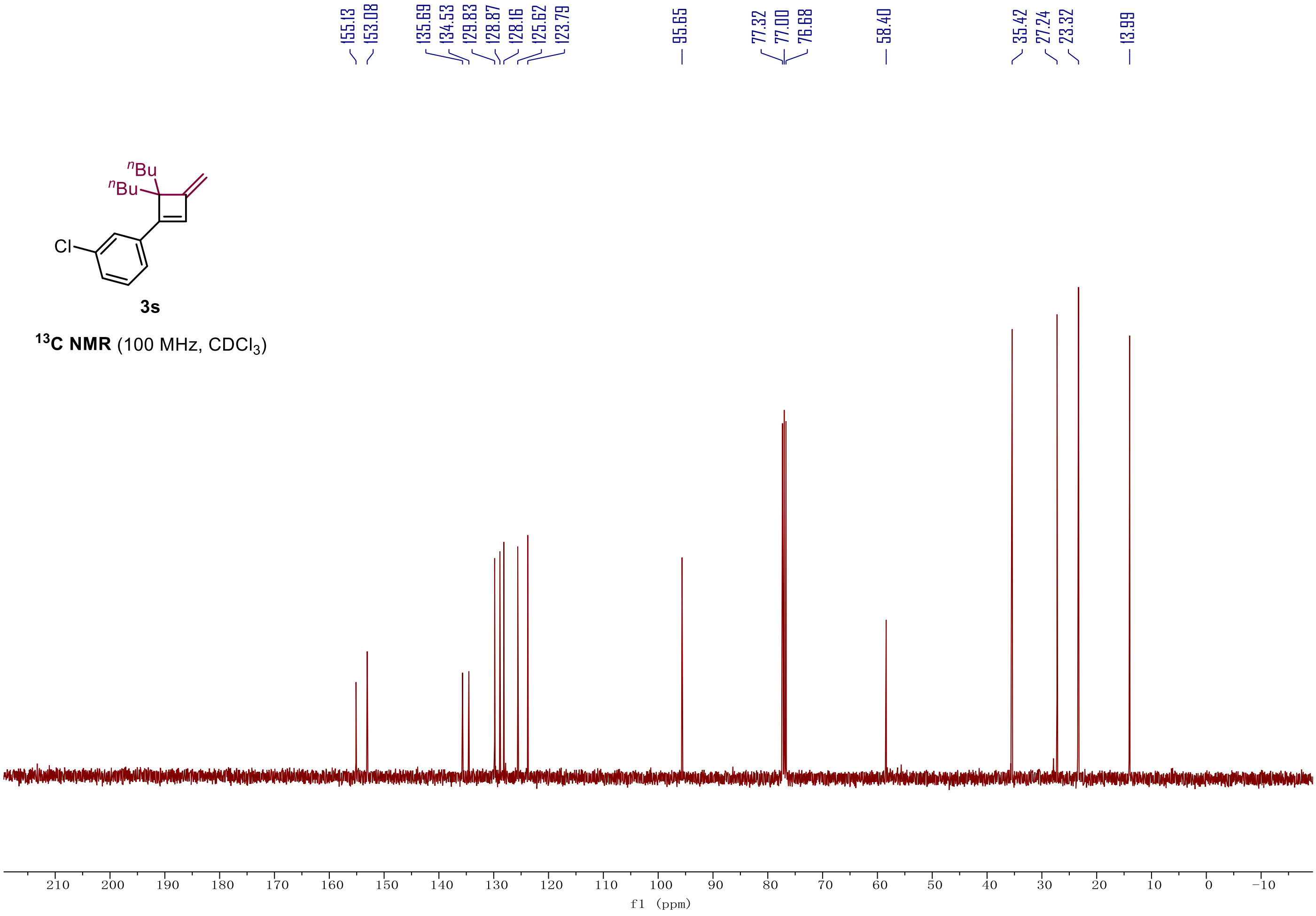
155.13 153.08 135.69 134.53 129.83 128.87 128.16 125.62 123.79 95.65 77.32 77.00 76.68 58.40 35.42 27.24 23.32 13.99
^{n}Bu
^{n}Bu
Cl
3s
^{13}C NMR (100 MHz, CDCl$_3$)
210 200 190 180 170 160 150 140 130 120 110 100 90 80 70 60 50 40 30 20 10 0 -10
f1 (ppm)

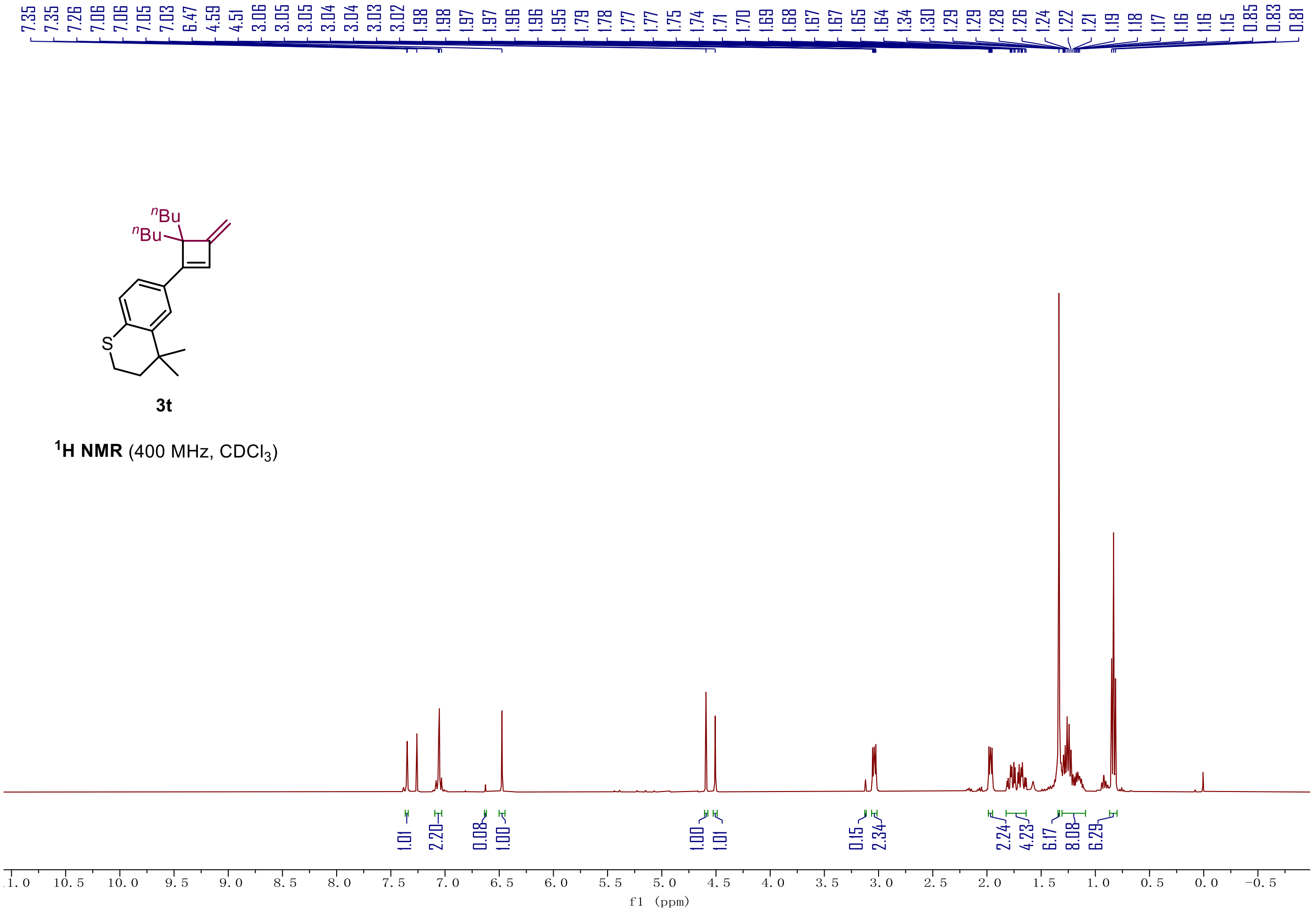
7.35 7.35 7.26 7.06 7.06 7.05 7.03 6.47 4.59 4.51 3.06 3.05 3.05 3.04 3.04 3.03 3.02 1.98 1.98 1.97 1.97 1.96 1.96 1.95 1.79 1.78 1.77 1.77 1.75 1.74 1.71 1.70 1.69 1.68 1.67 1.67 1.65 1.64 1.34 1.30 1.29 1.29 1.28 1.26 1.24 1.22 1.21 1.19 1.18 1.17 1.16 1.16 1.15 0.85 0.83 0.81
^{n}Bu
^{n}Bu
S
3t
^{1}H NMR (400 MHz, $CDCl_3$)
1.01 2.20 0.08 1.00 1.00 1.01 0.15 2.34 2.24 4.23 6.17 8.08 6.29
f1 (ppm)

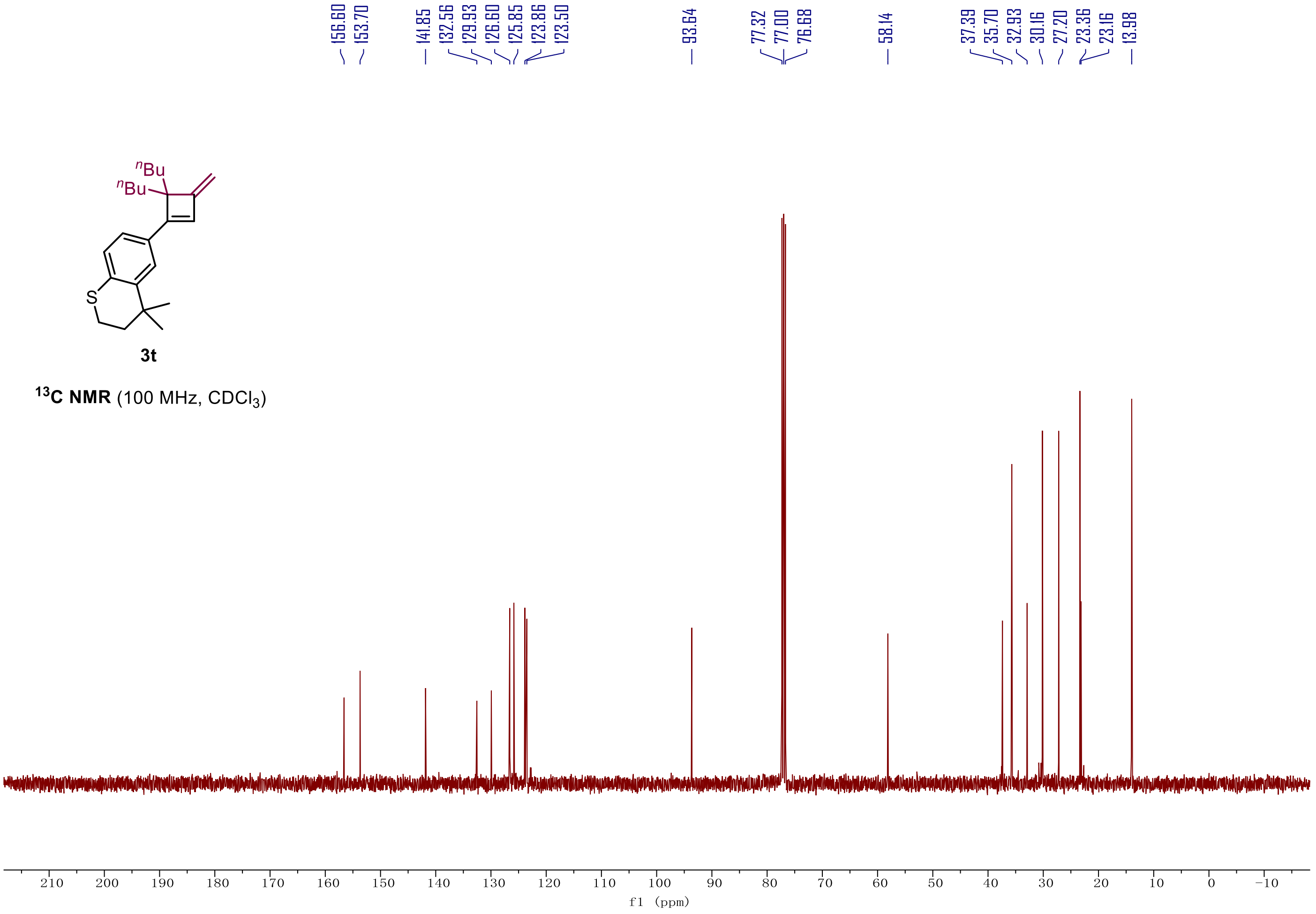
156.60 153.70 141.85 132.56 129.93 126.60 125.85 123.86 123.50 93.64 77.32 77.00 76.68 58.14 37.39 35.70 32.93 30.16 27.20 23.36 23.16 13.98
^{n}Bu
^{n}Bu
S
3t
^{13}C NMR (100 MHz, $CDCl_3$)
f1 (ppm)

3u

^{1}H NMR (400 MHz, $CDCl_3$)

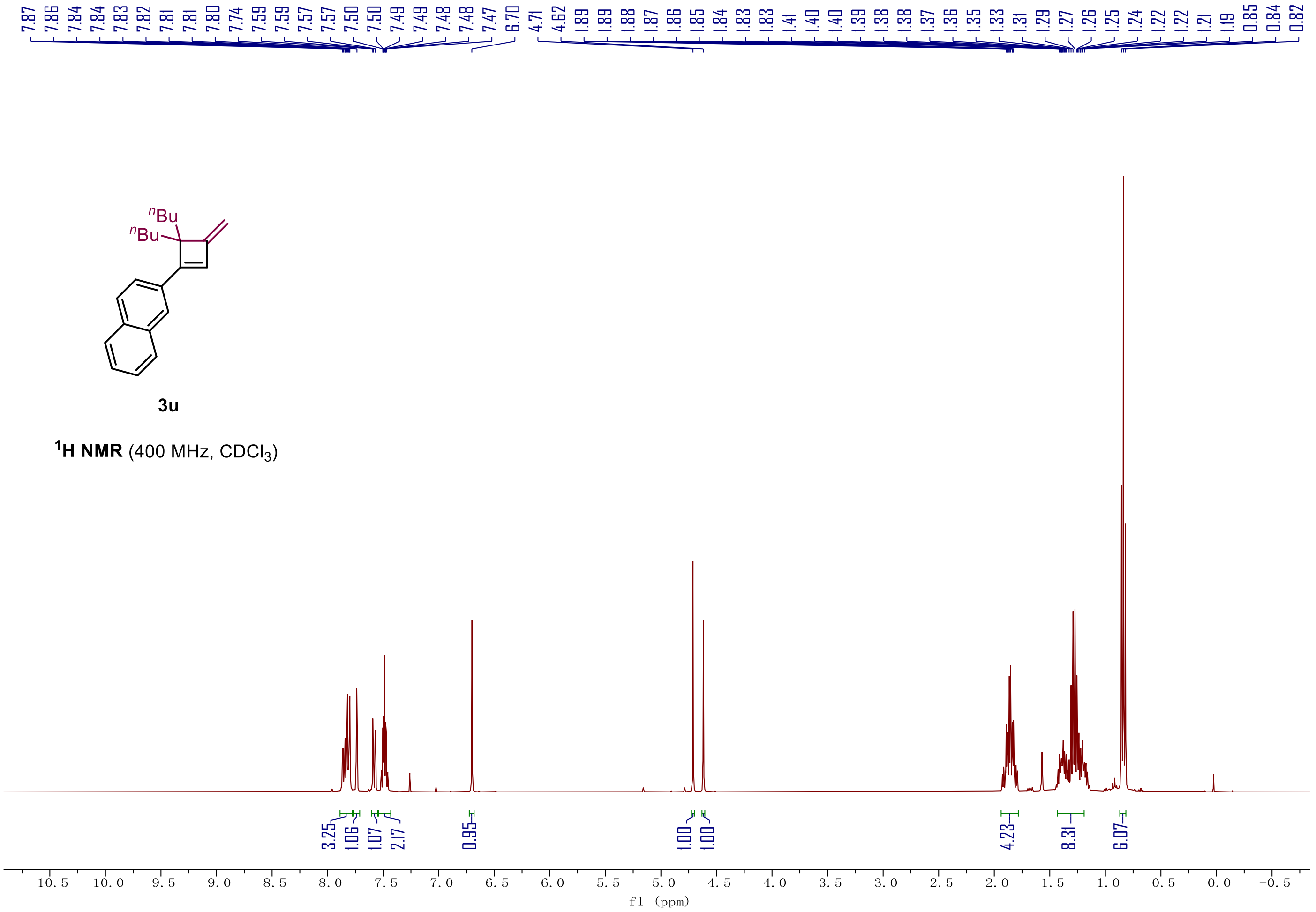

3u

^{13}C NMR (100 MHz, $CDCl_3$)

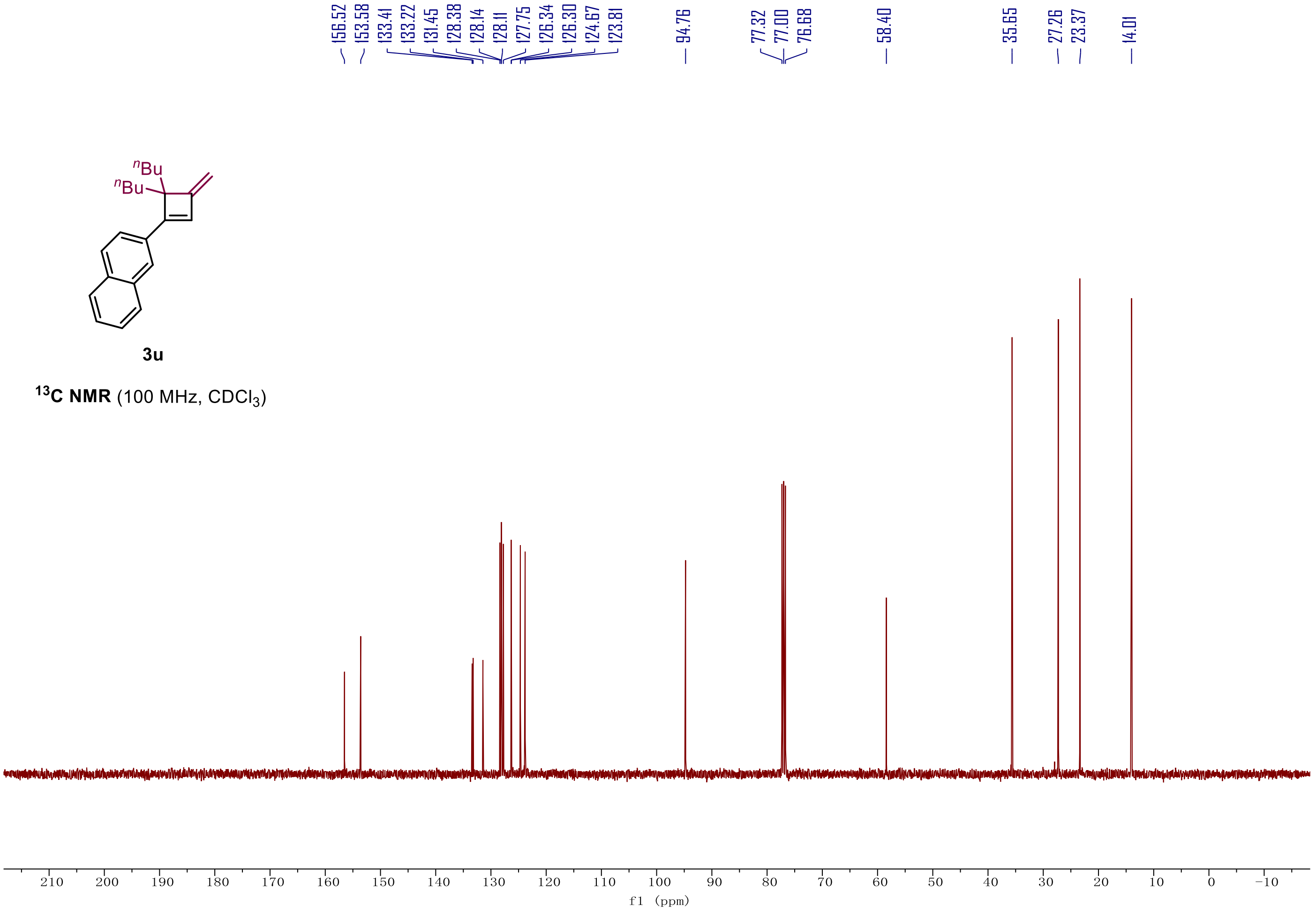

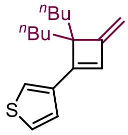

3v

^{1}H NMR (400 MHz, $CDCl_3$)

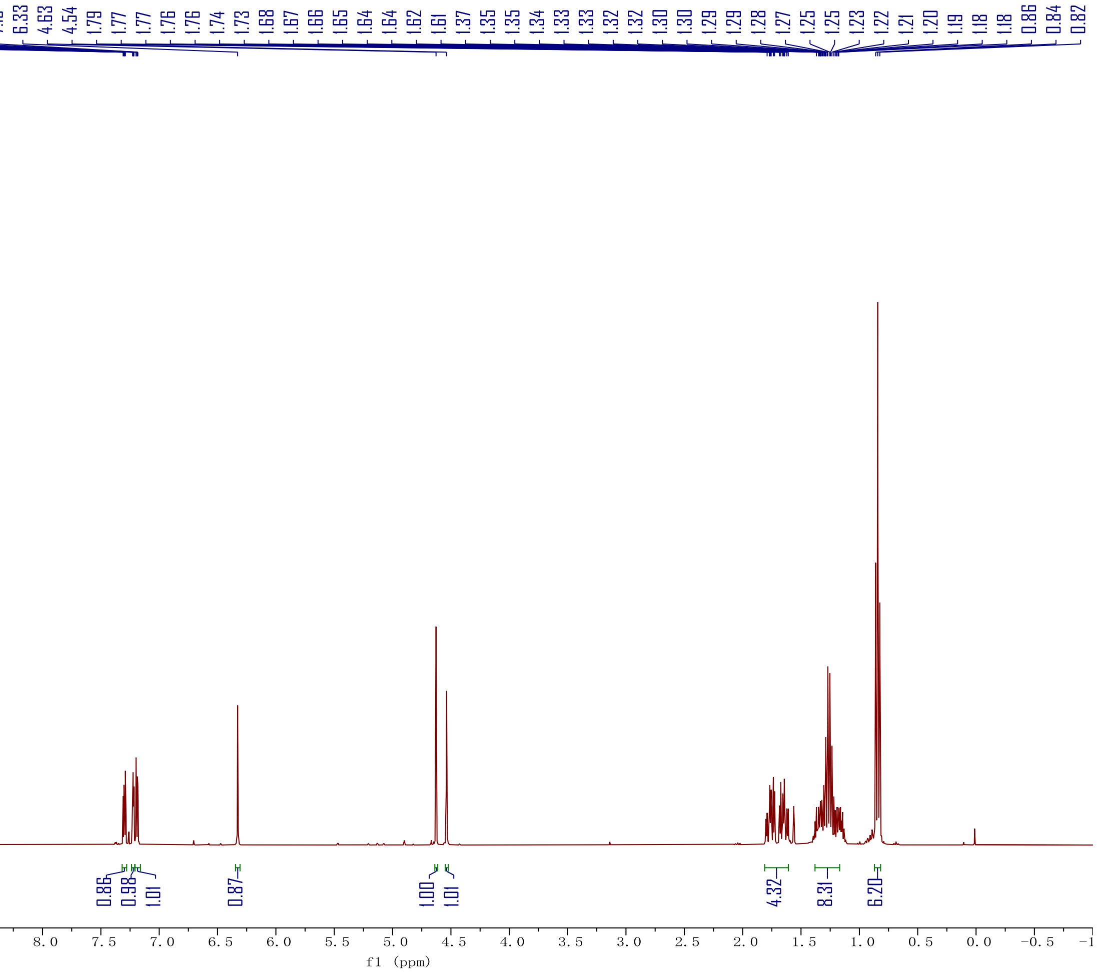

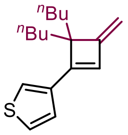

3v

^{13}C NMR (100 MHz, $CDCl_3$)

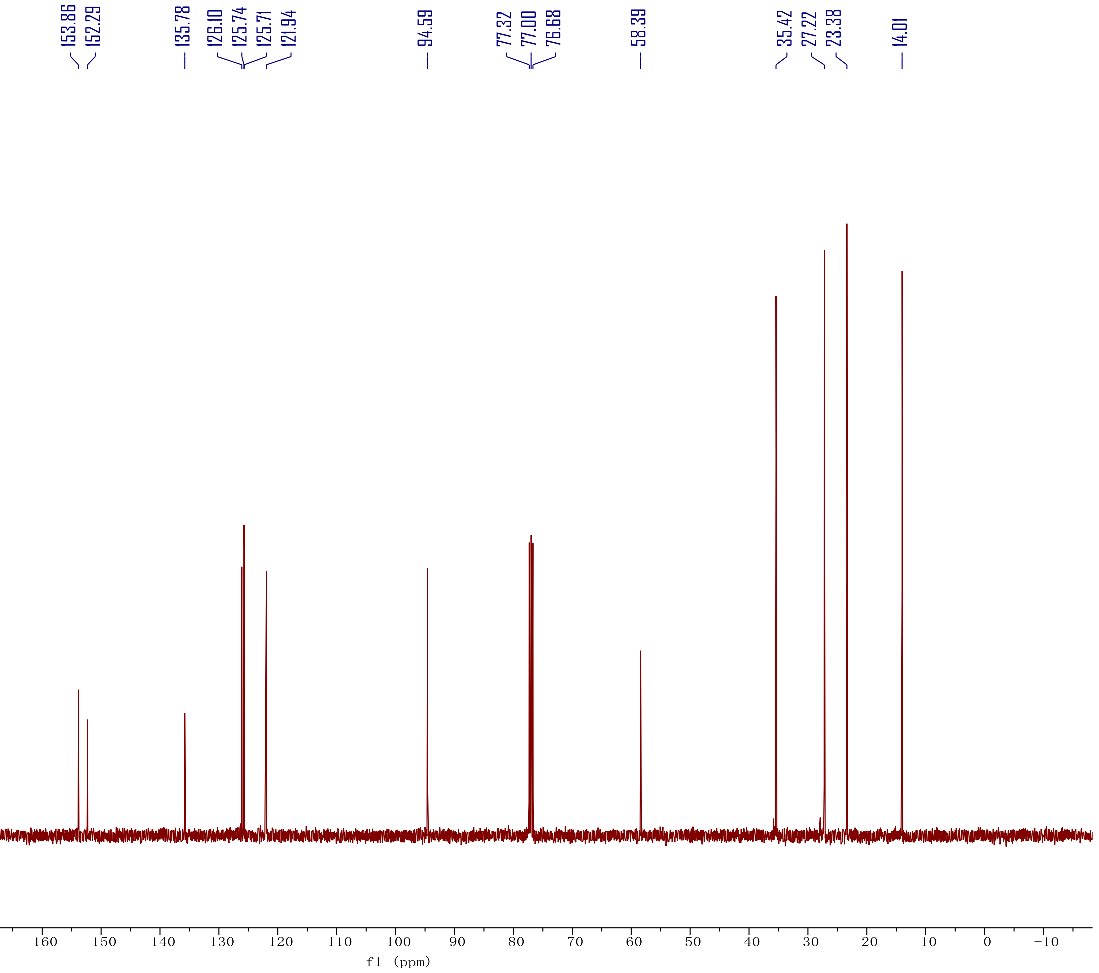

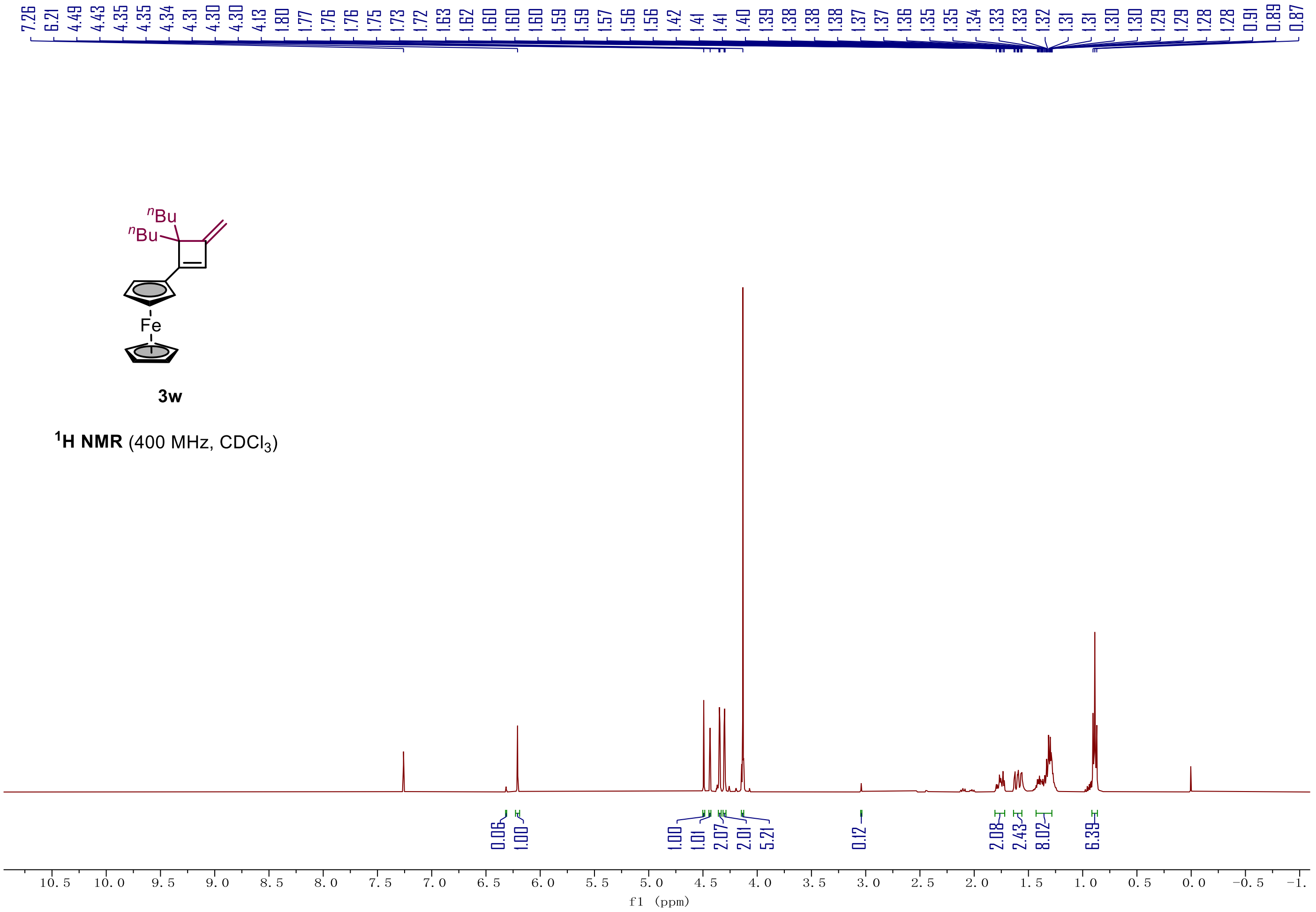
7.26 6.21 4.49 4.43 4.35 4.35 4.34 4.31 4.30 4.30 4.13 1.80 1.77 1.76 1.76 1.75 1.73 1.72 1.63 1.62 1.60 1.60 1.60 1.59 1.59 1.57 1.56 1.56 1.42 1.41 1.41 1.40 1.39 1.38 1.38 1.38 1.38 1.37 1.37 1.36 1.35 1.35 1.34 1.33 1.33 1.33 1.32 1.31 1.31 1.30 1.30 1.29 1.29 1.28 1.28 0.91 0.89 0.87
nBu
nBu
Fe
3w
1H NMR (400 MHz, CDCl3)
0.06 1.00 1.00 1.01 2.07 2.01 5.21 0.12 2.08 2.43 8.02 6.39
f1 (ppm)

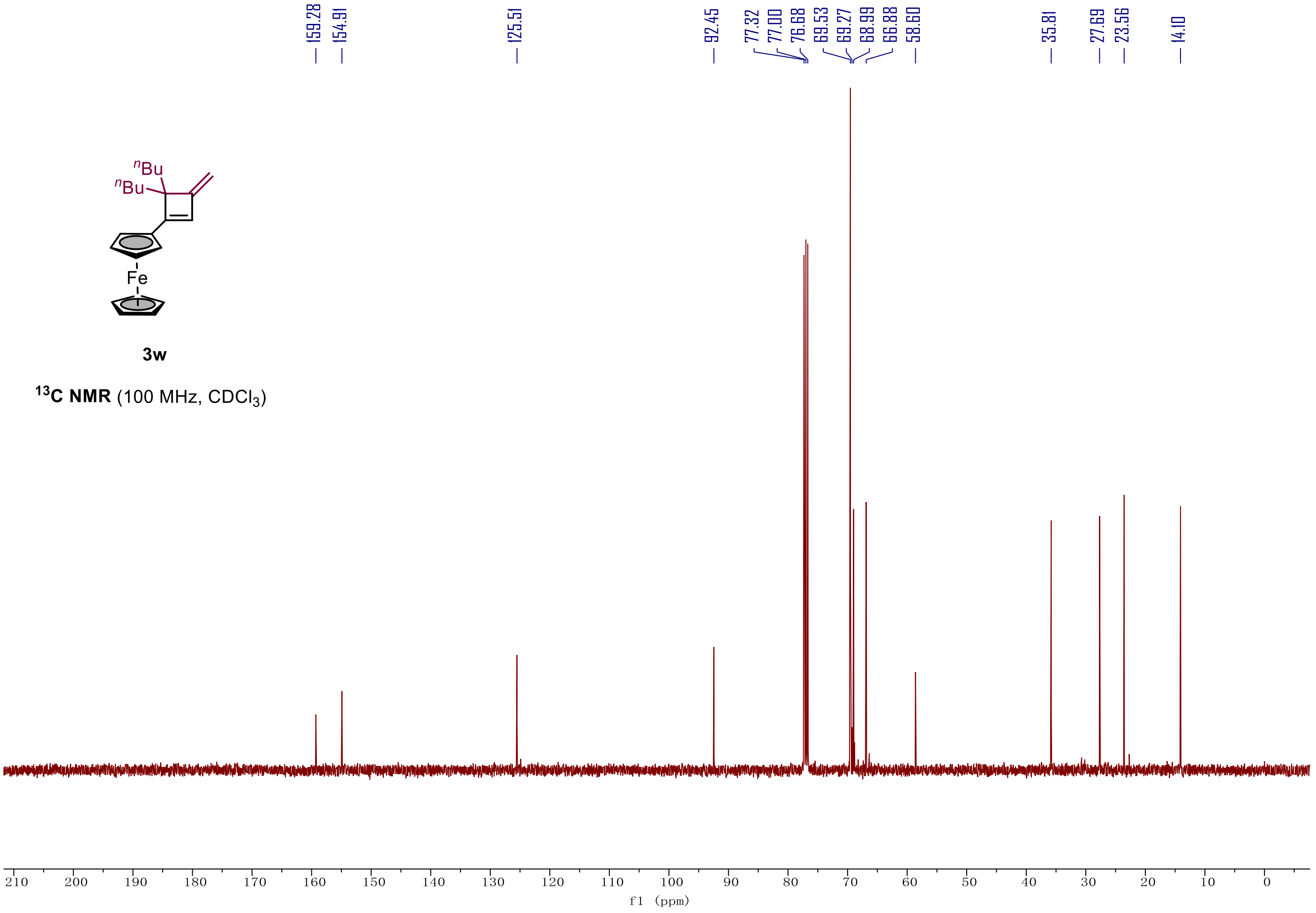
159.28 154.91 125.51 92.45 77.32 77.00 76.68 69.53 69.27 68.99 66.88 58.60 35.81 27.69 23.56 14.10
nBu
nBu
Fe
3w
13C NMR (100 MHz, CDCl3)
f1 (ppm)

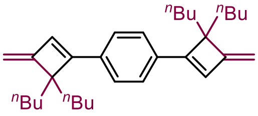

3x

^{1}H NMR (400 MHz, $CDCl_3$)

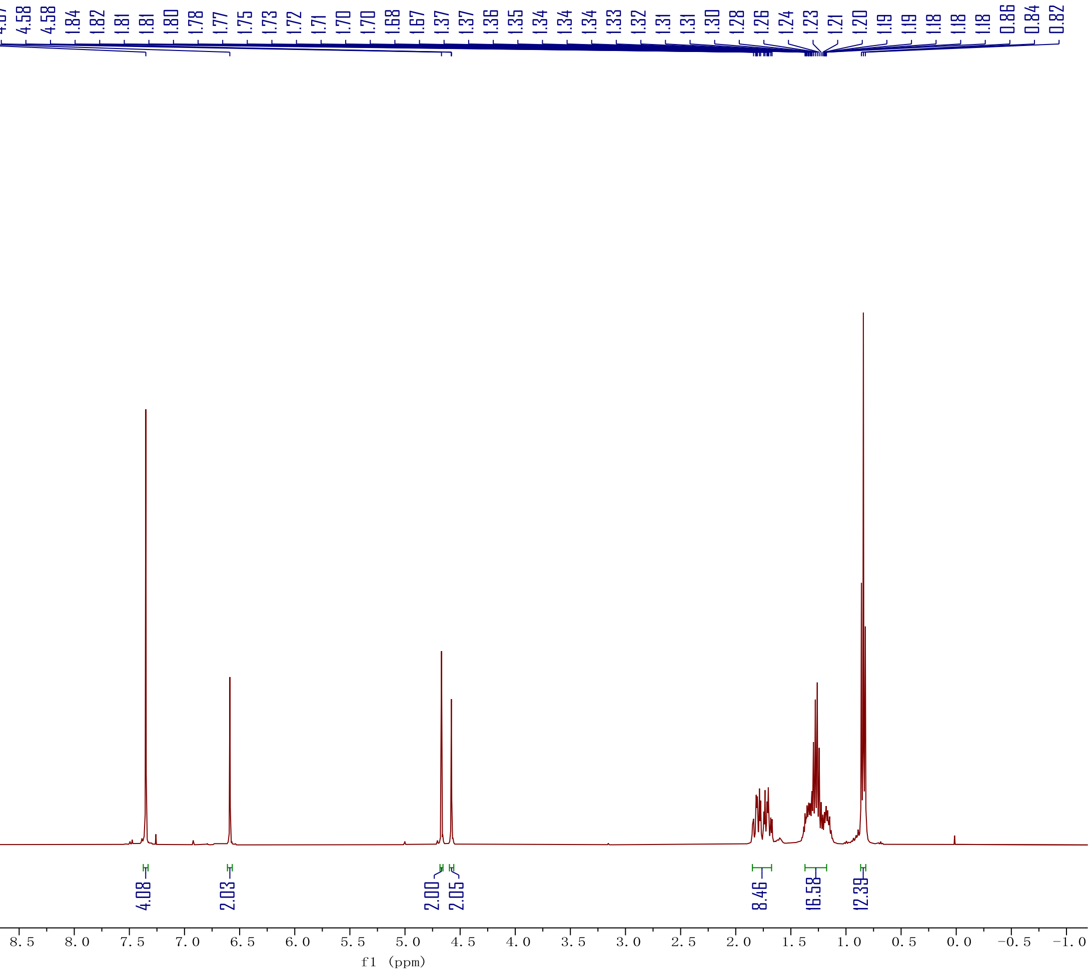

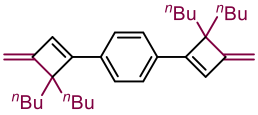

3x

^{13}C NMR (100 MHz, $CDCl_3$)

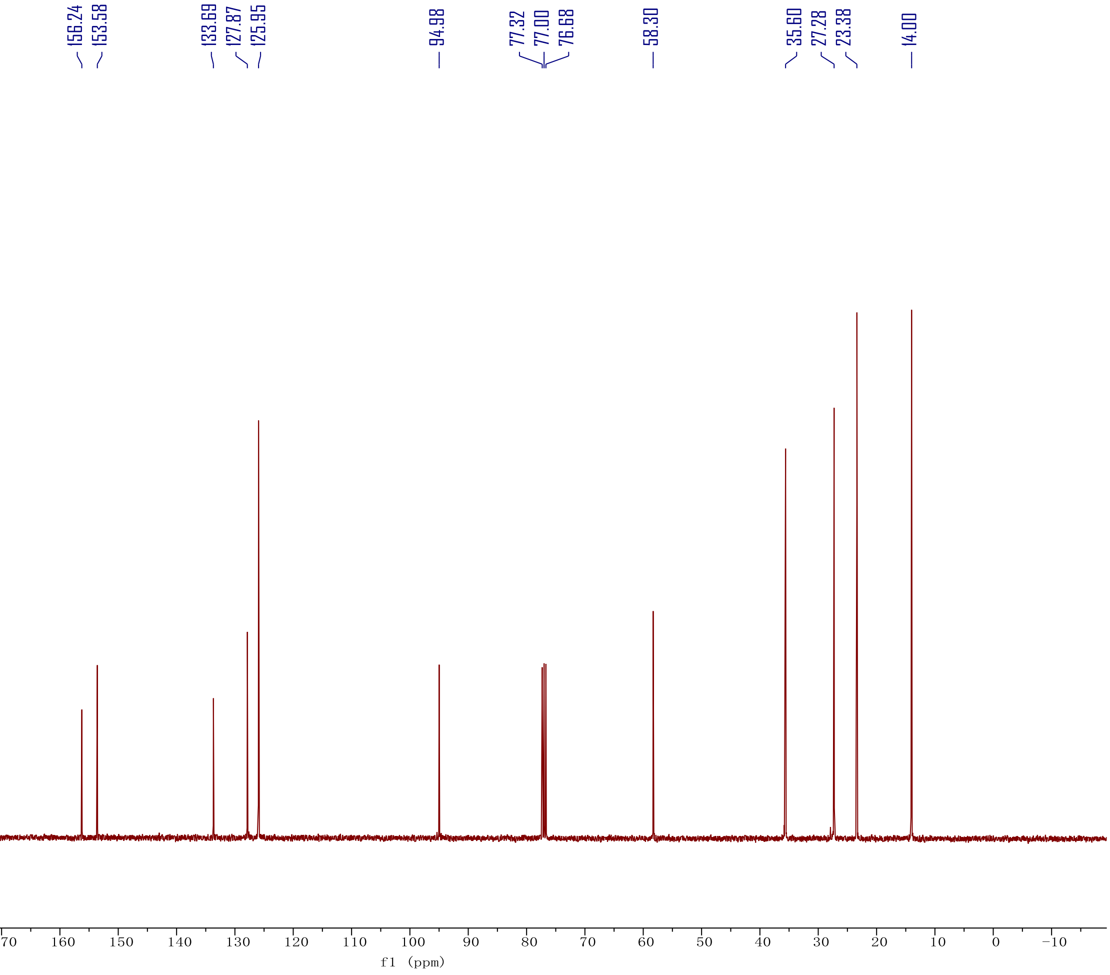

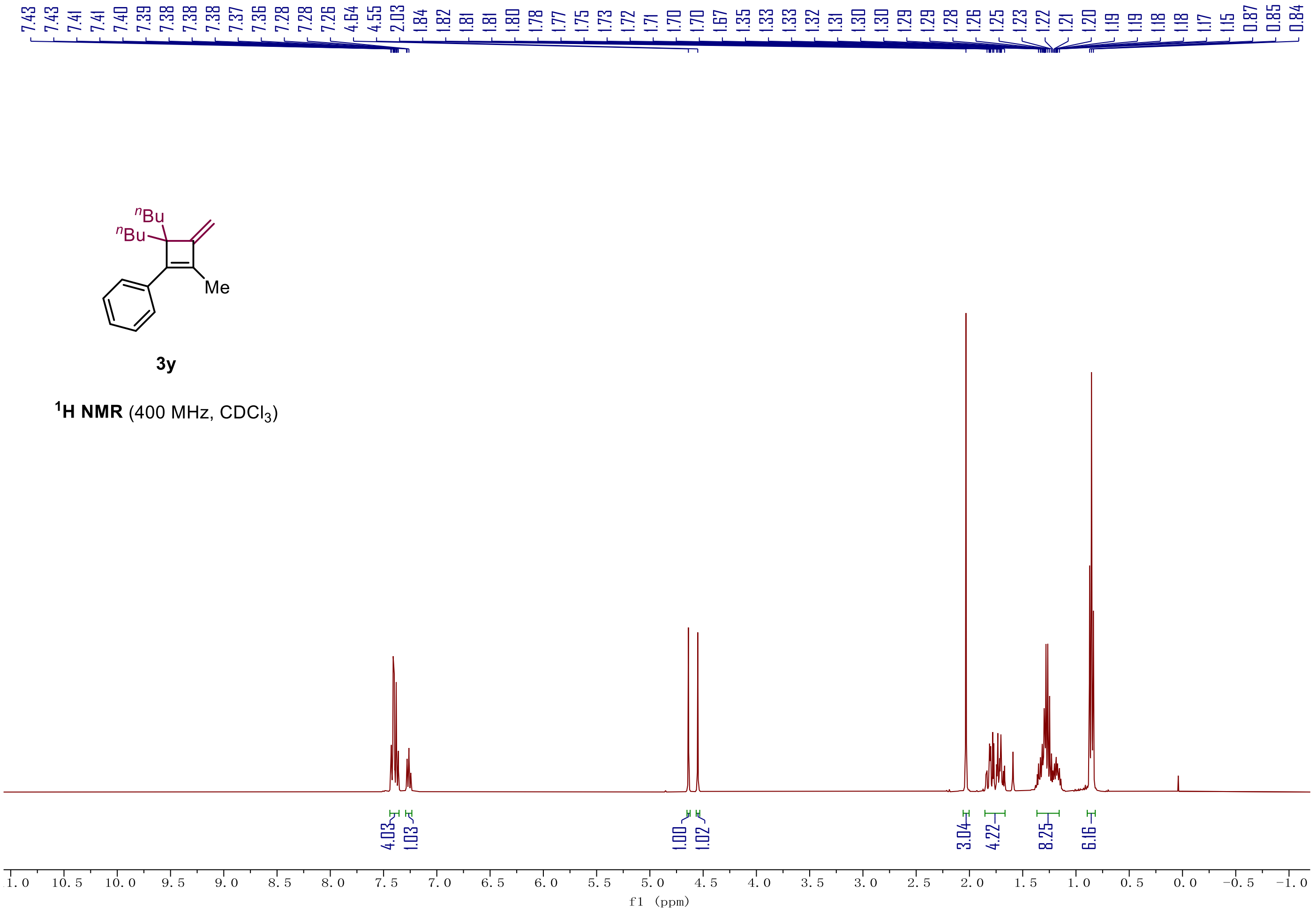
7.43 7.43 7.41 7.41 7.40 7.39 7.38 7.38 7.38 7.37 7.36 7.28 7.28 7.26 4.64 4.55 2.03 1.84 1.82 1.81 1.81 1.80 1.78 1.77 1.75 1.73 1.72 1.71 1.70 1.70 1.67 1.35 1.33 1.33 1.32 1.31 1.30 1.30 1.29 1.29 1.28 1.26 1.25 1.23 1.22 1.21 1.20 1.19 1.19 1.18 1.18 1.17 1.15 0.87 0.85 0.84
^{n}Bu
^{n}Bu
Me
3y
^{1}H NMR (400 MHz, $CDCl_3$)
4.03 1.03 1.00 1.02 3.04 4.22 8.25 6.16
f1 (ppm)

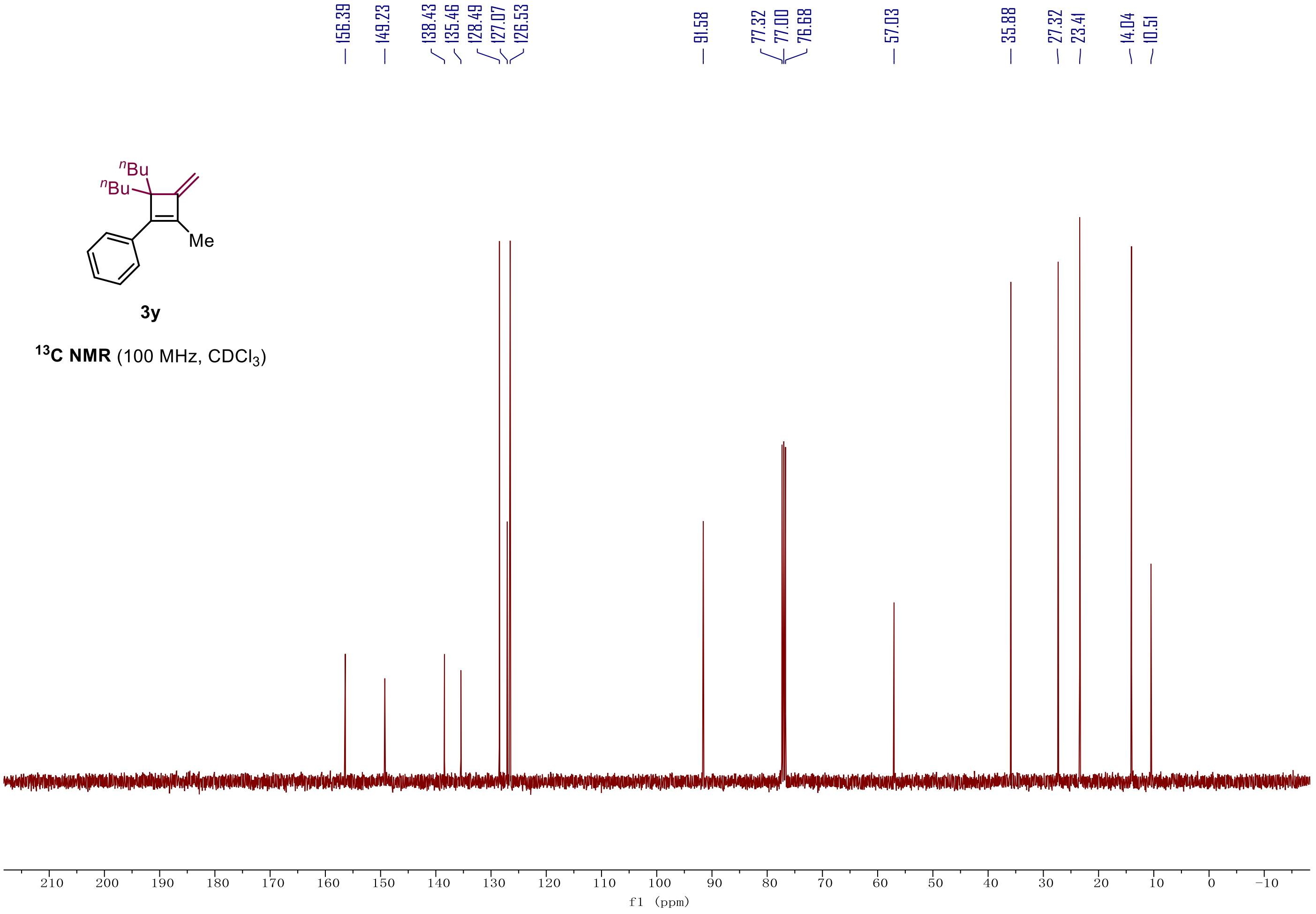
156.39 149.23 138.43 135.46 128.49 127.07 126.53 91.58 77.32 77.00 76.68 57.03 35.88 27.32 23.41 14.04 10.51
^{n}Bu
^{n}Bu
Me
3y
^{13}C NMR (100 MHz, $CDCl_3$)
f1 (ppm)

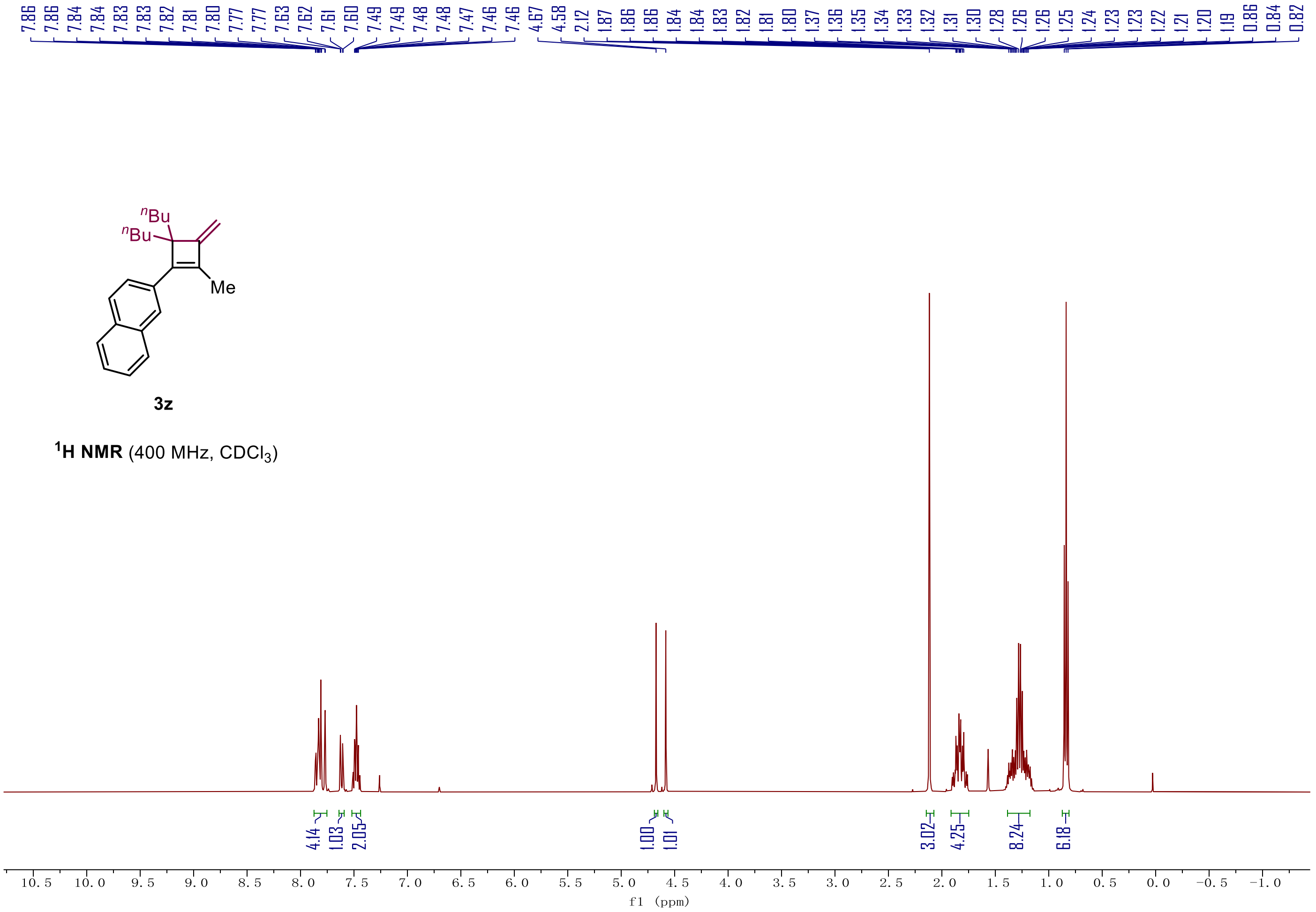
7.86 7.86 7.84 7.84 7.83 7.83 7.82 7.81 7.80 7.77 7.77 7.63 7.62 7.61 7.60 7.49 7.49 7.48 7.48 7.47 7.46 7.46 4.67 4.58 2.12 1.87 1.86 1.86 1.84 1.84 1.83 1.82 1.81 1.80 1.37 1.36 1.35 1.34 1.33 1.32 1.31 1.30 1.28 1.26 1.26 1.25 1.24 1.23 1.23 1.22 1.21 1.20 1.19 0.86 0.84 0.82
ⁿBu
ⁿBu
Me
3z
1H NMR (400 MHz, CDCl3)
4.14 1.03 2.05 1.00 1.01 3.02 4.25 8.24 6.18
10.5 10.0 9.5 9.0 8.5 8.0 7.5 7.0 6.5 6.0 5.5 5.0 4.5 4.0 3.5 3.0 2.5 2.0 1.5 1.0 0.5 0.0 -0.5 -1.0
f1 (ppm)

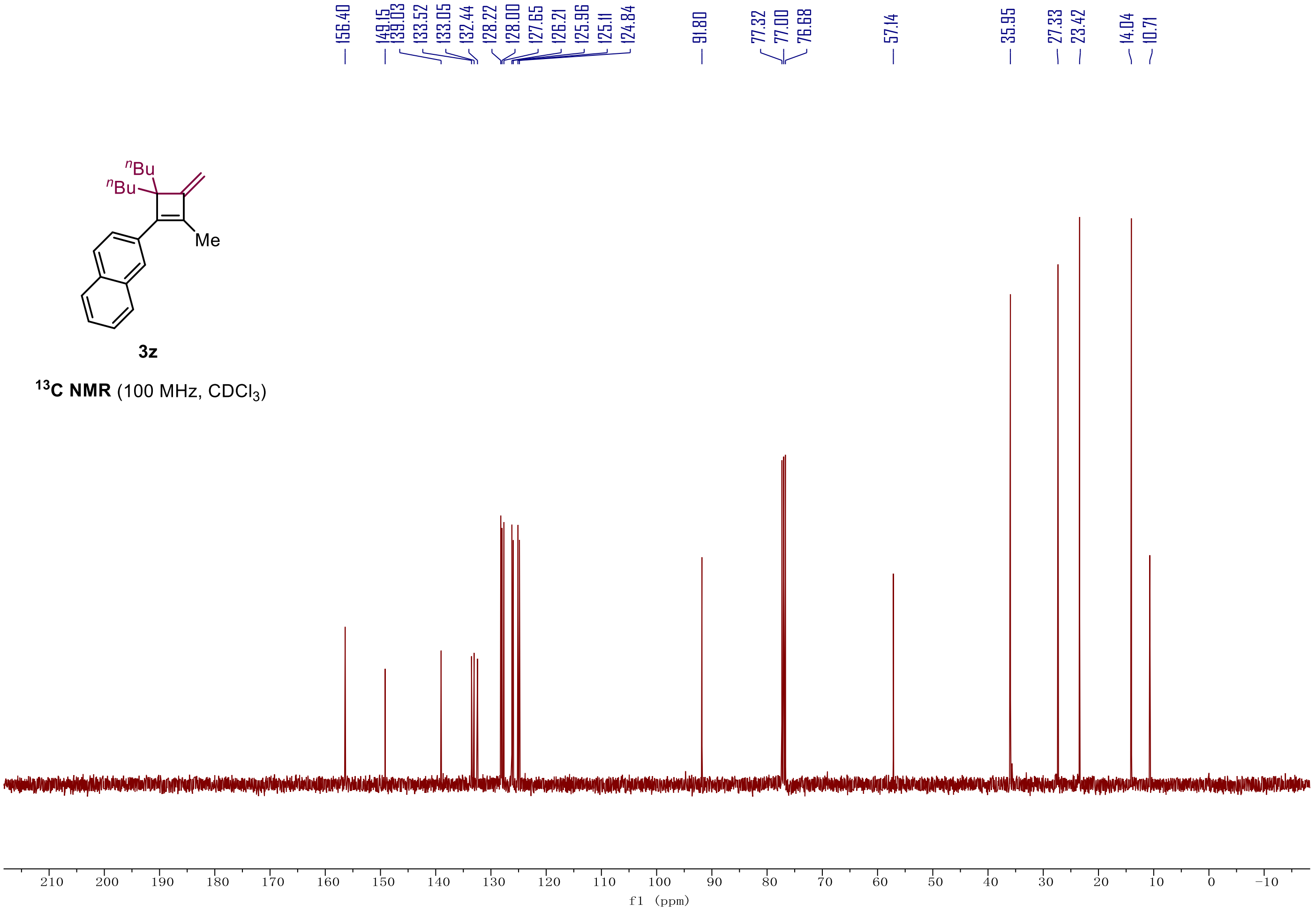
156.40 149.15 139.03 133.52 133.05 132.44 128.22 128.00 127.65 126.21 125.96 125.11 124.84 91.80 77.32 77.00 76.68 57.14 35.95 27.33 23.42 14.04 10.71
ⁿBu
ⁿBu
Me
3z
13C NMR (100 MHz, CDCl3)
210 200 190 180 170 160 150 140 130 120 110 100 90 80 70 60 50 40 30 20 10 0 -10
f1 (ppm)

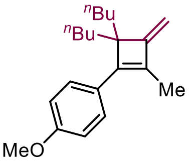

3aa

^{1}H NMR (400 MHz, $CDCl_3$)

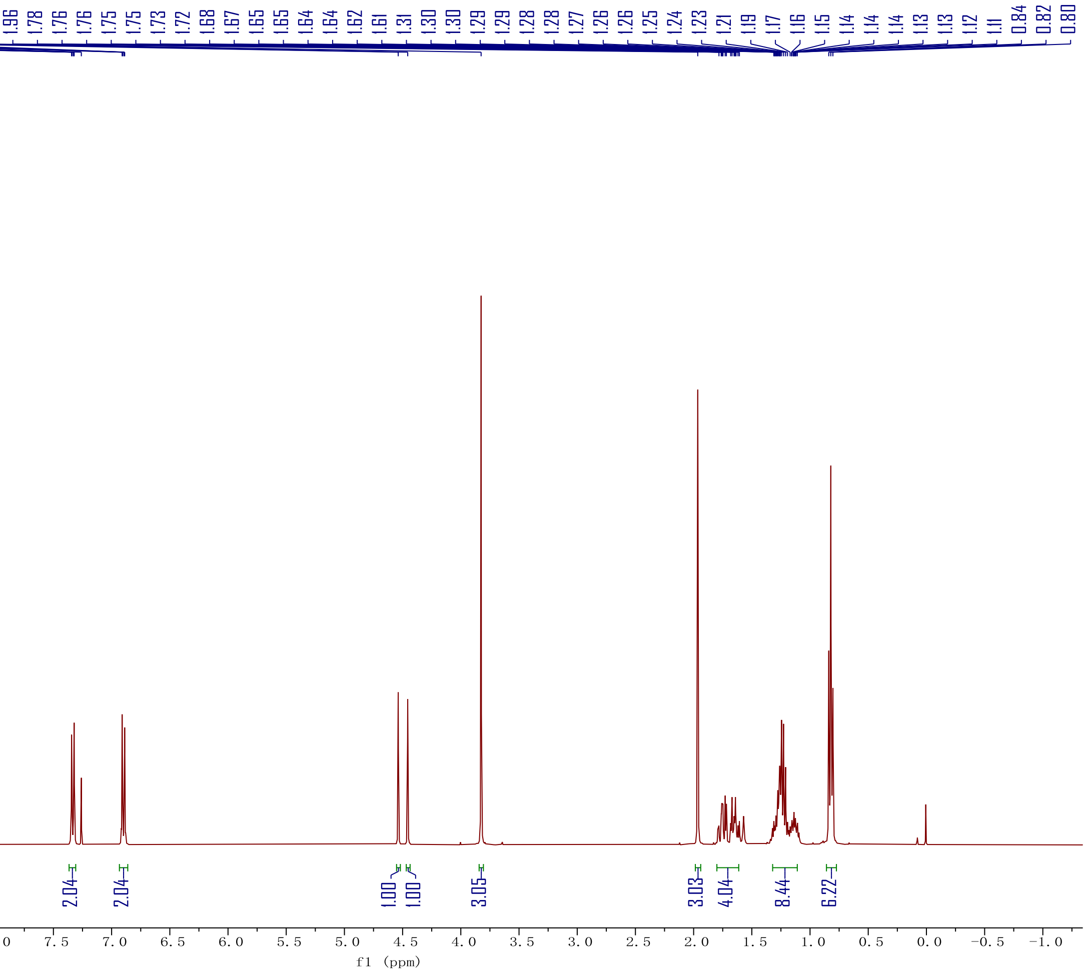

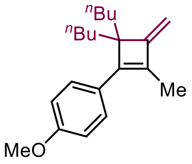

3aa

^{13}C NMR (100 MHz, $CDCl_3$)

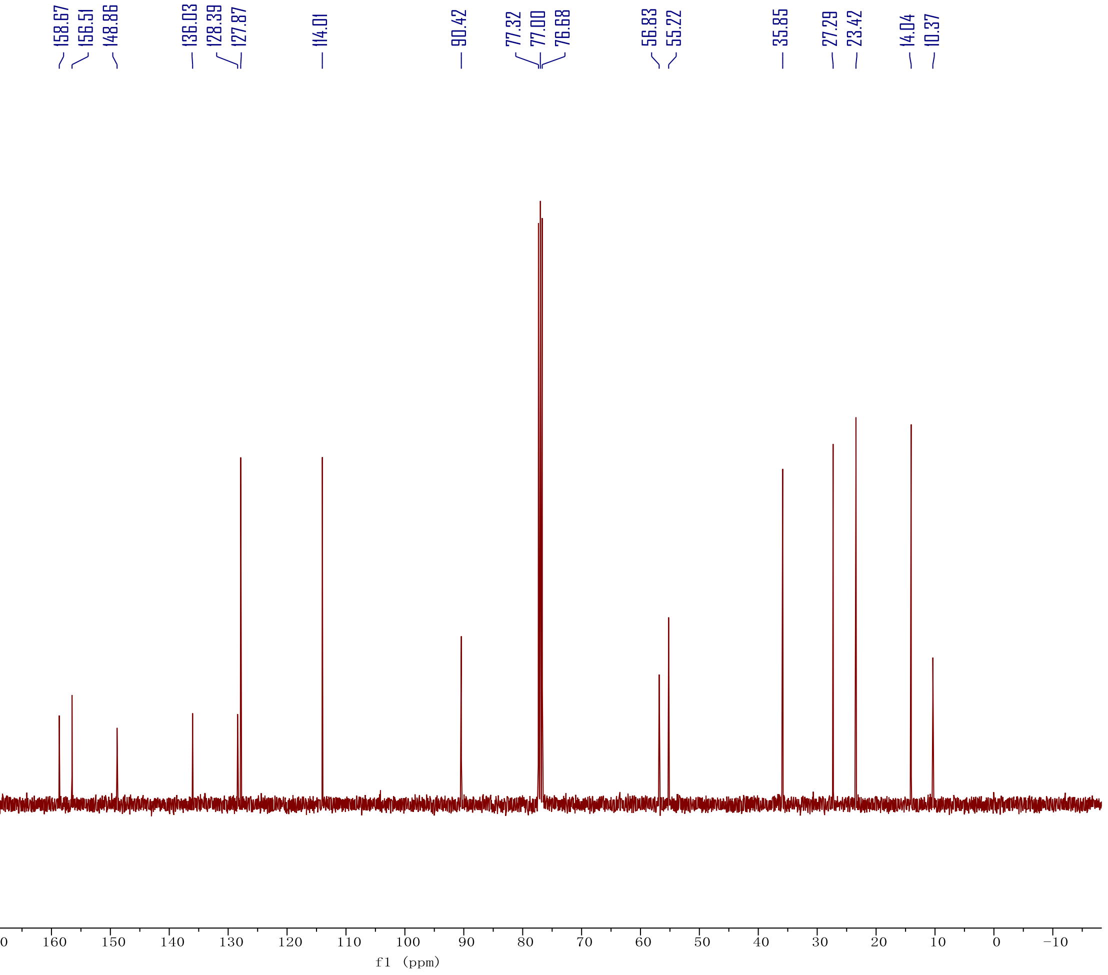

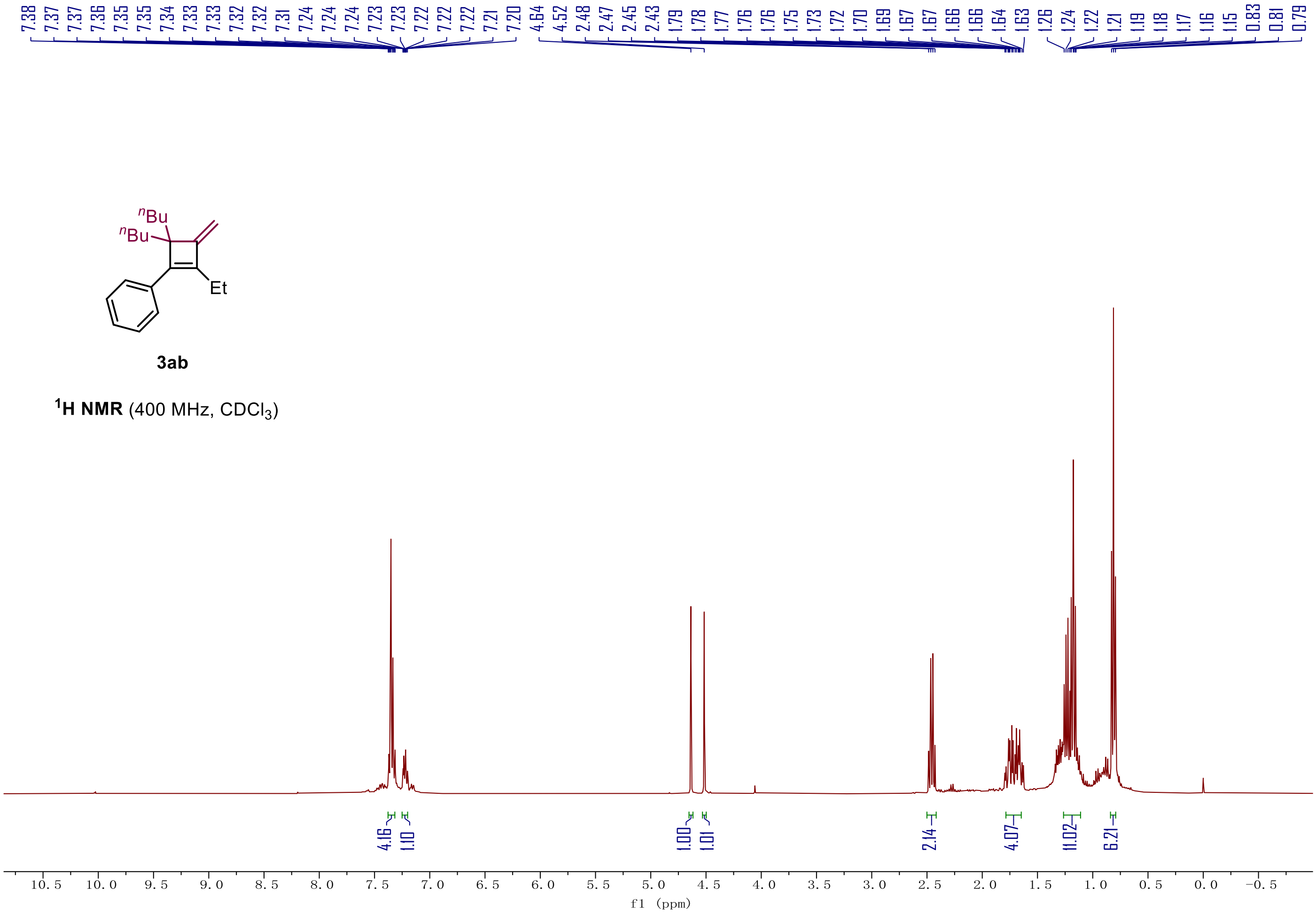

3ab

^{1}H NMR (400 MHz, $CDCl_3$)

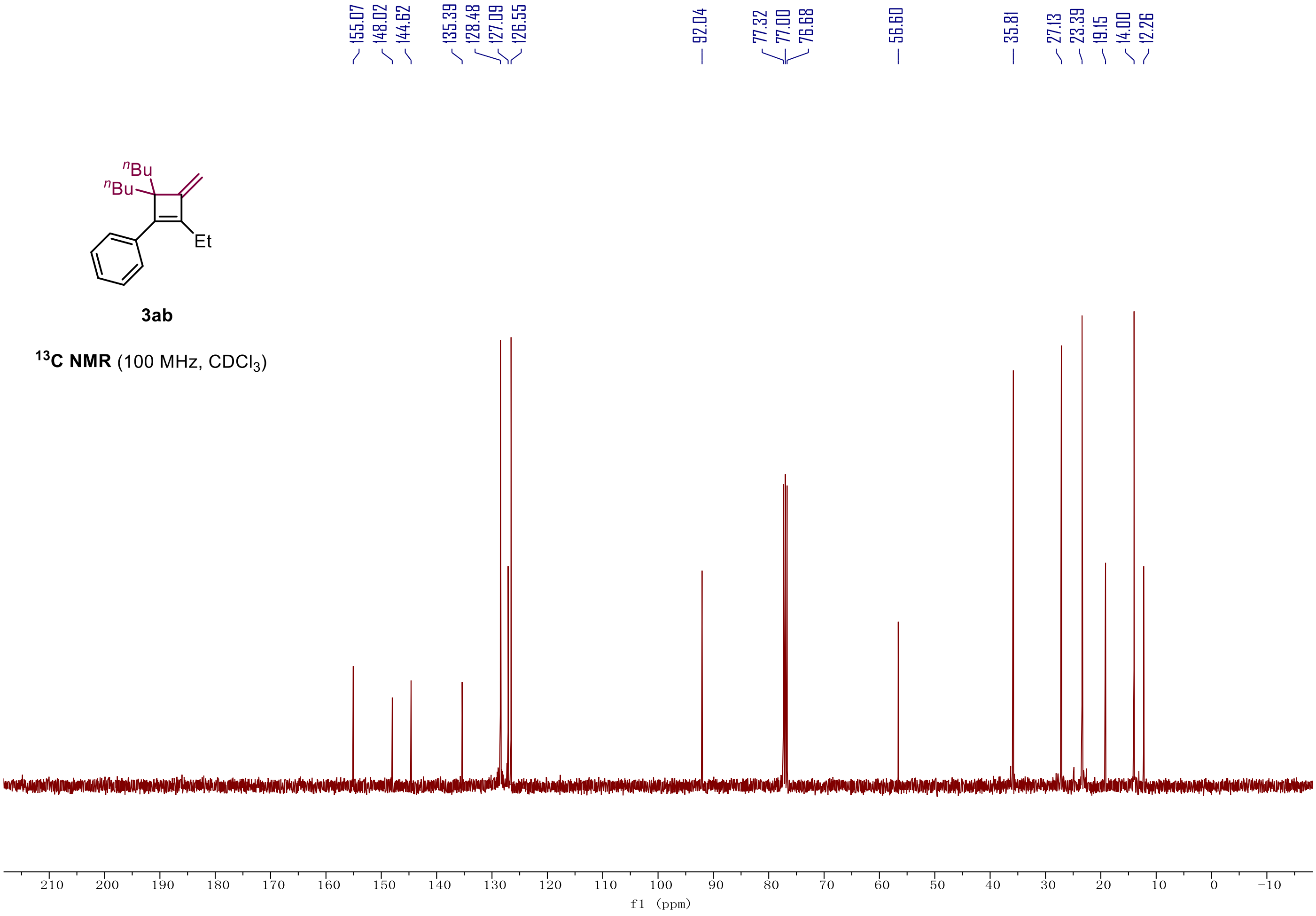

3ab

^{13}C NMR (100 MHz, $CDCl_3$)

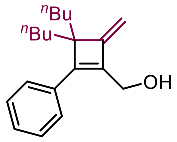

3ac

^{1}H NMR (400 MHz, $CDCl_3$)

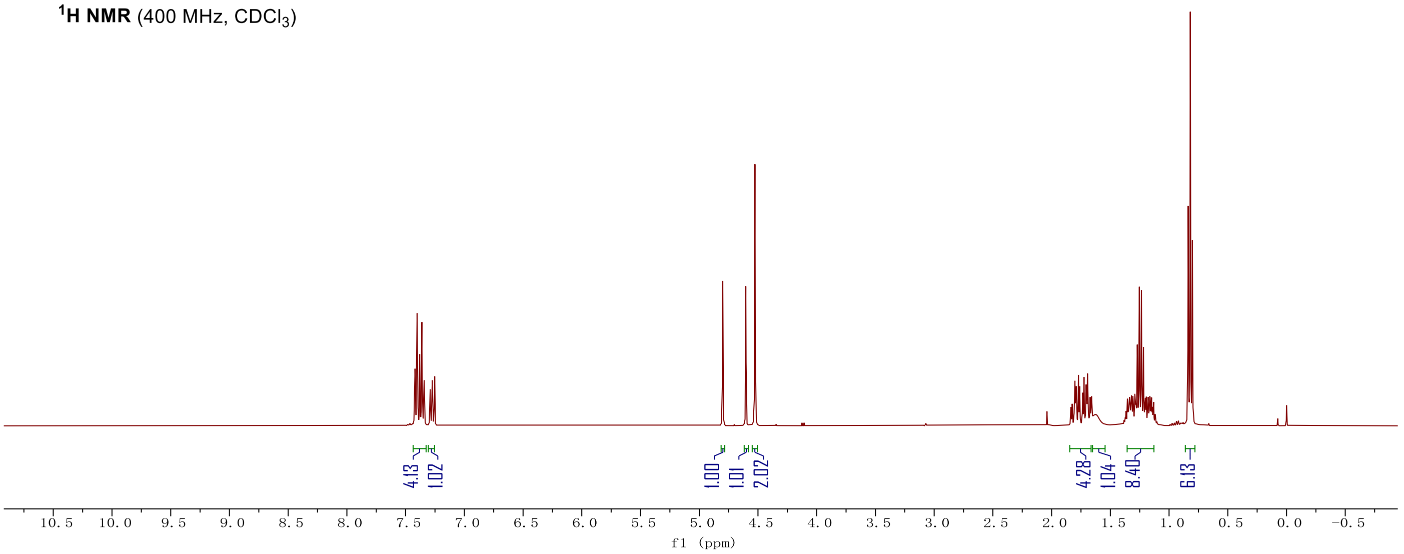

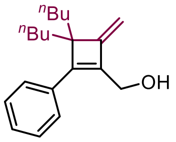

3ac

^{13}C NMR (100 MHz, $CDCl_3$)

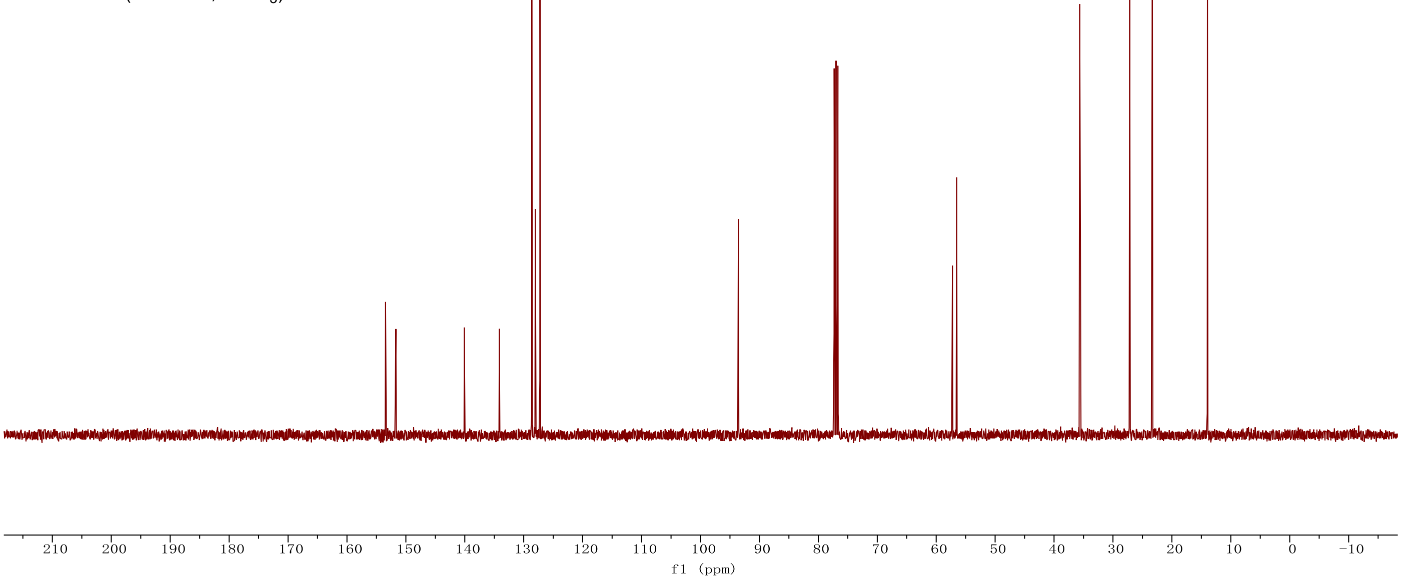

3ad

^{1}H NMR (400 MHz, $CDCl_3$)

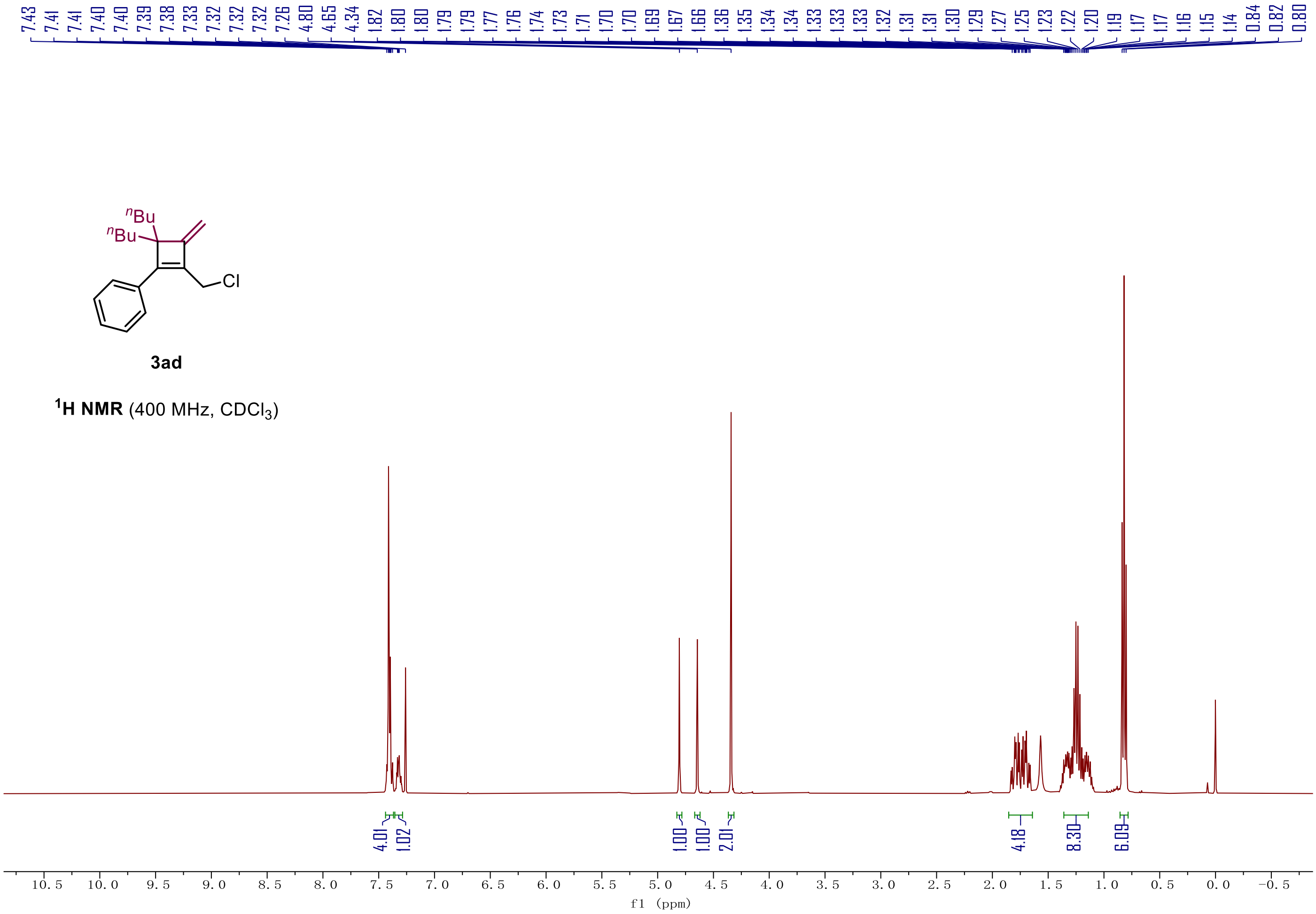

3ad

^{13}C NMR (100 MHz, $CDCl_3$)

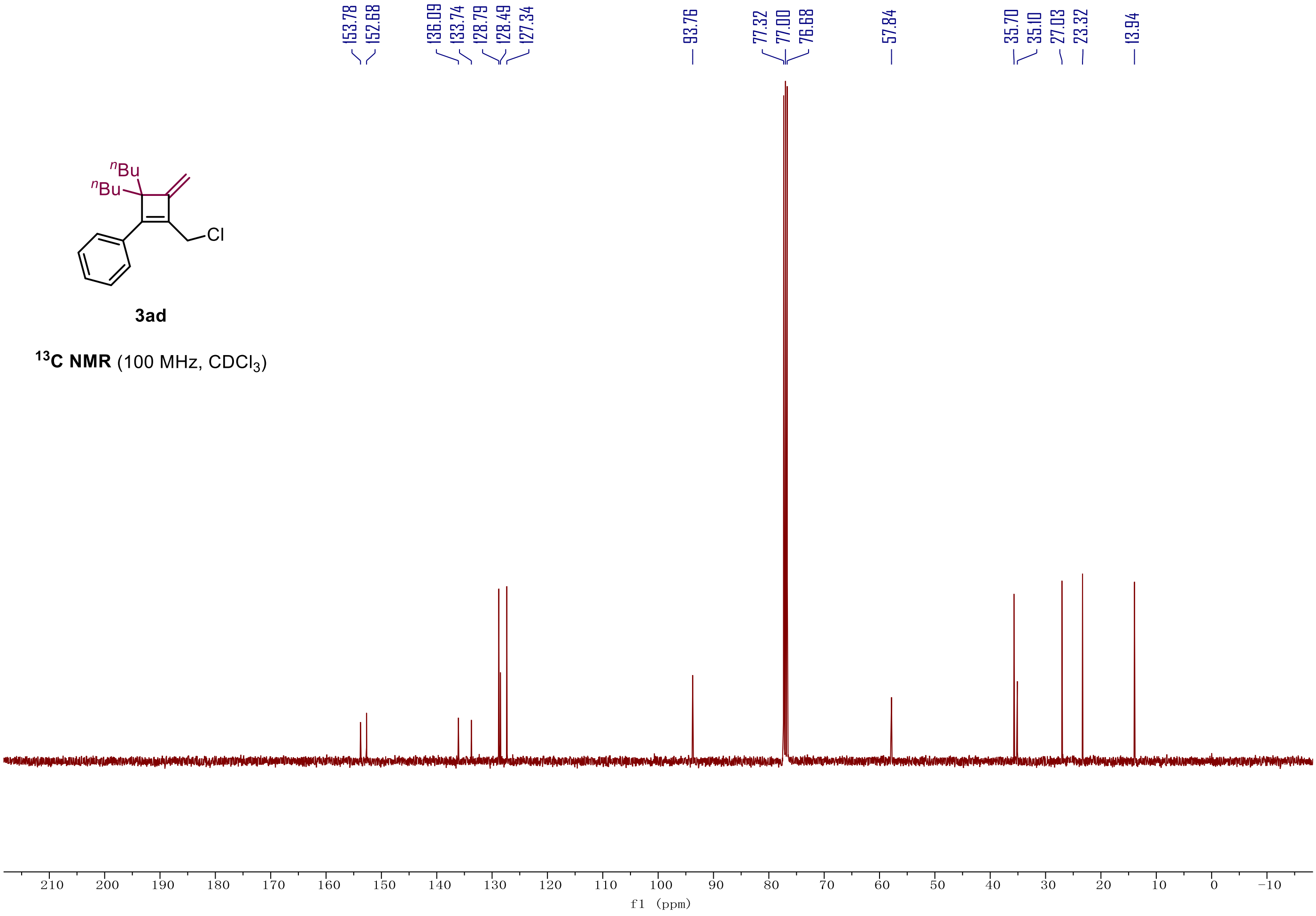

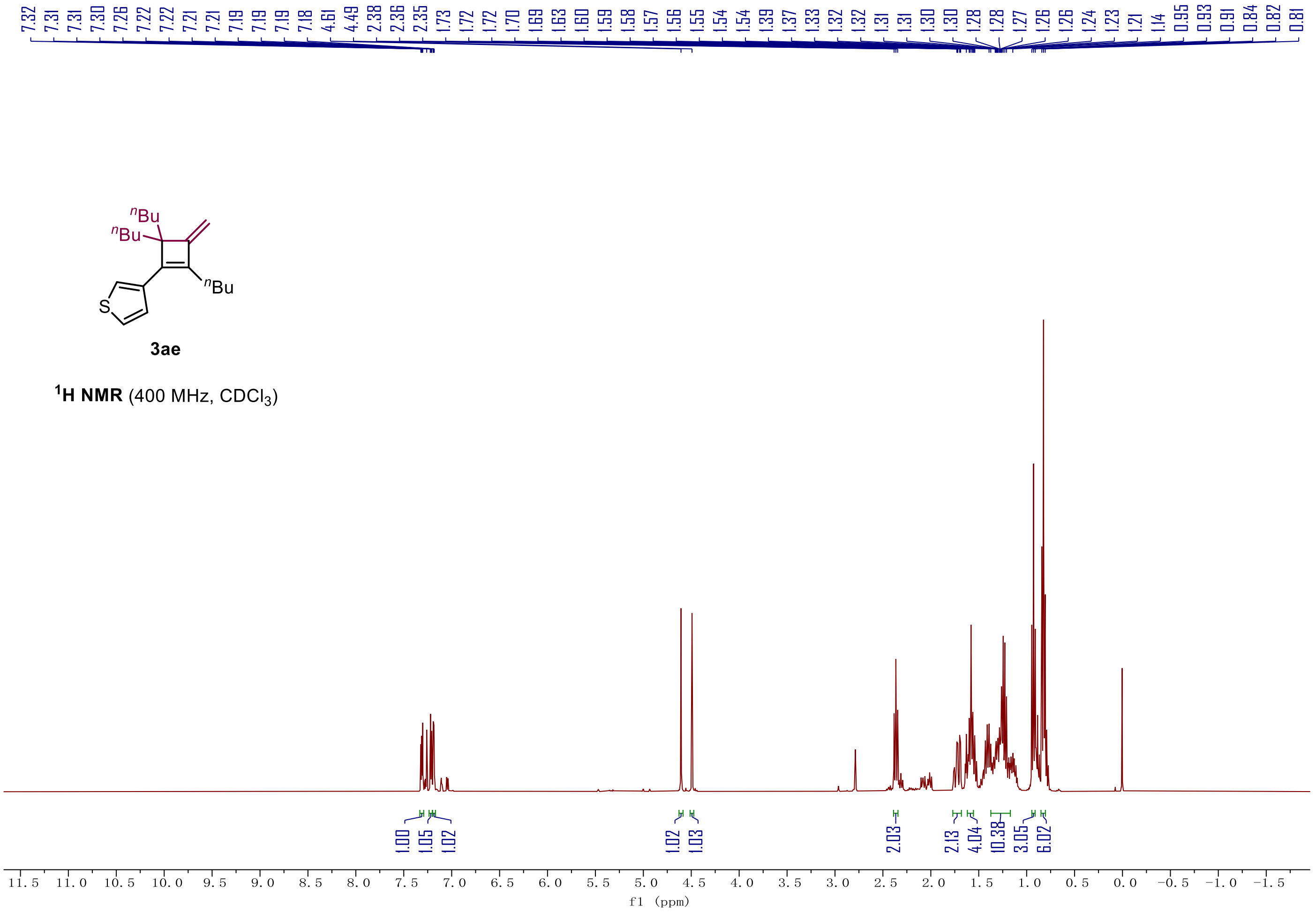
7.32 7.31 7.31 7.30 7.26 7.22 7.22 7.21 7.21 7.19 7.19 7.19 7.18 4.61 4.49 2.38 2.36 2.35 1.73 1.72 1.72 1.70 1.69 1.63 1.60 1.59 1.58 1.57 1.56 1.55 1.54 1.54 1.39 1.37 1.33 1.32 1.32 1.31 1.31 1.30 1.30 1.28 1.28 1.27 1.26 1.26 1.24 1.23 1.21 1.14 0.95 0.93 0.91 0.84 0.82 0.81
^{n}Bu
^{n}Bu
S
^{n}Bu
3ae
^{1}H NMR (400 MHz, CDCl$_3$)
1.00 1.05 1.02 1.02 1.03 2.03 2.13 4.04 10.38 3.05 6.02
f1 (ppm)

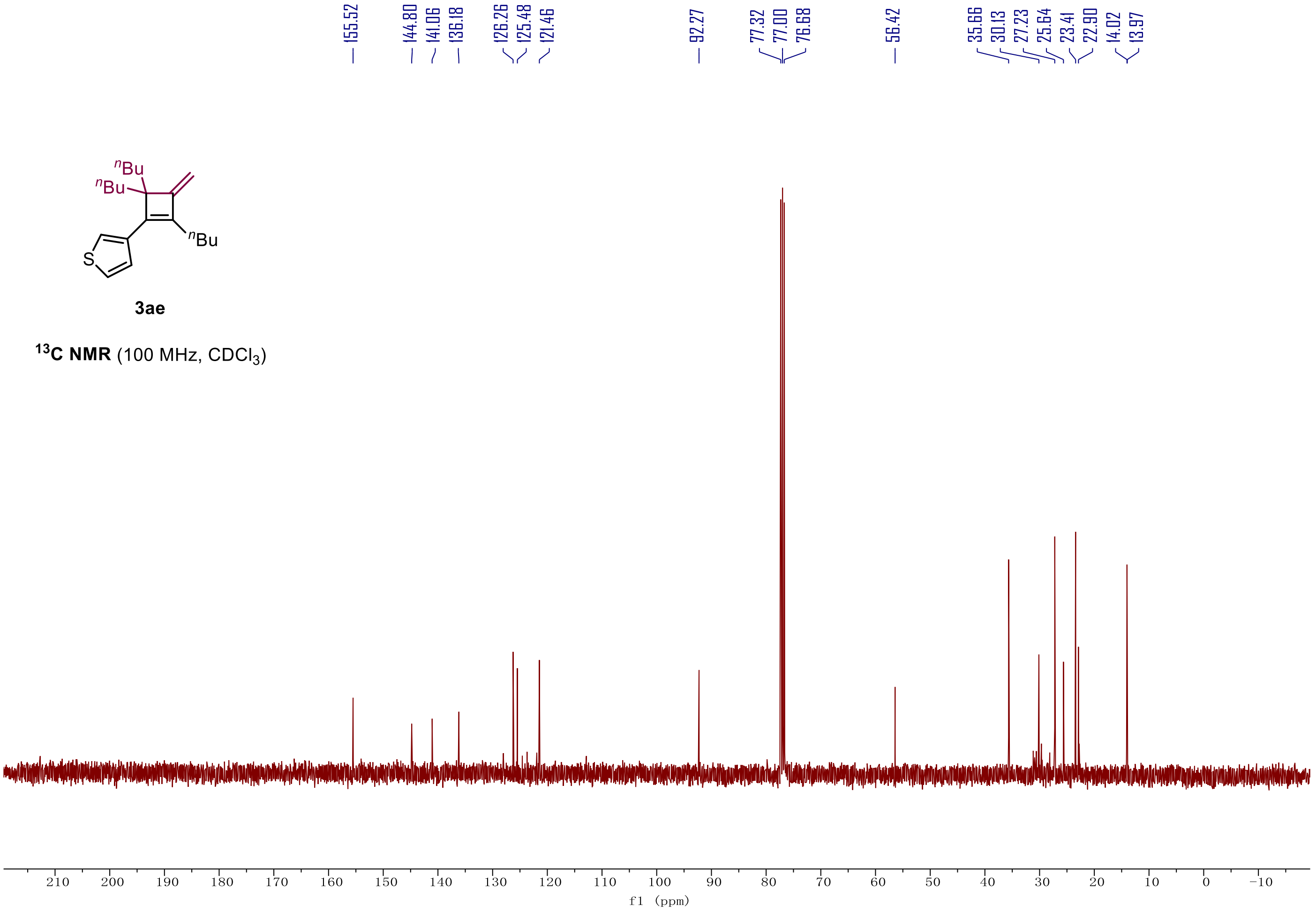
155.52 144.80 141.06 136.18 126.26 125.48 121.46 92.27 77.32 77.00 76.68 56.42 35.66 30.13 27.23 25.64 23.41 22.90 14.02 13.97
^{n}Bu
^{n}Bu
S
^{n}Bu
3ae
^{13}C NMR (100 MHz, CDCl$_3$)
f1 (ppm)

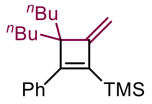

3af

^{1}H NMR (400 MHz, $CDCl_3$)

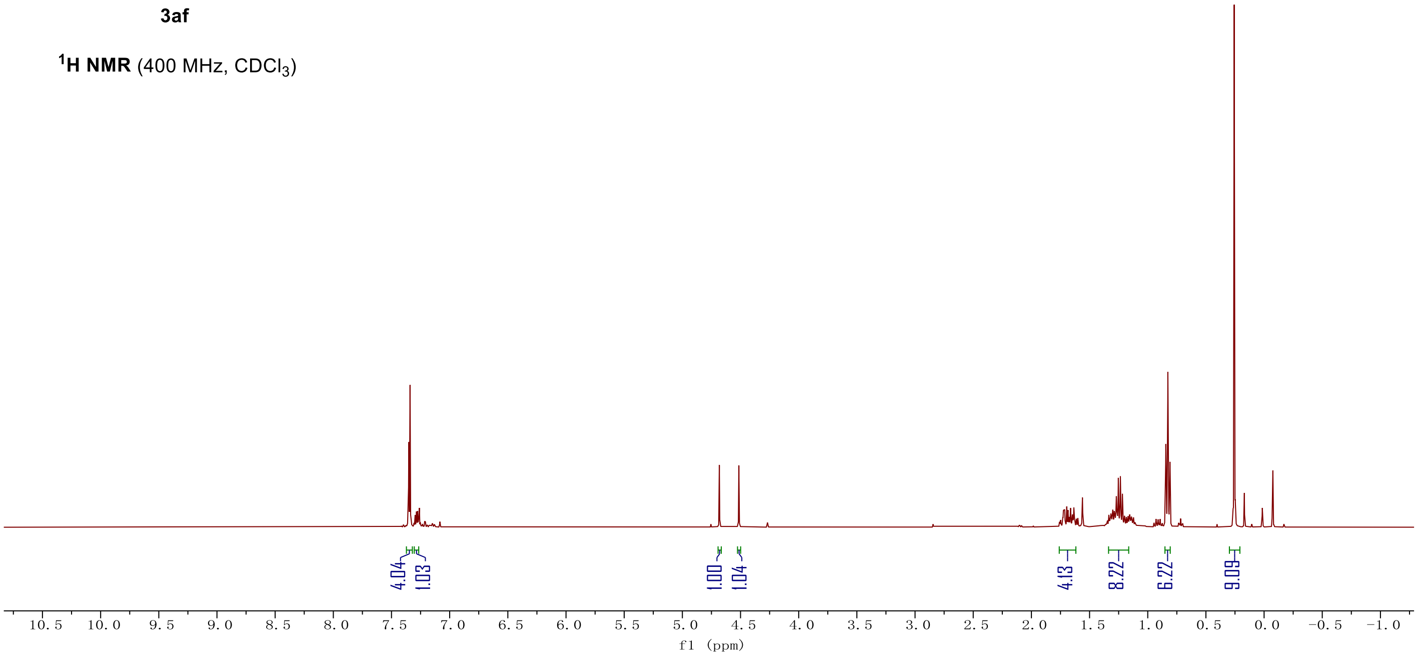

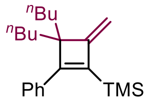

3af

^{13}C NMR (100 MHz, $CDCl_3$)

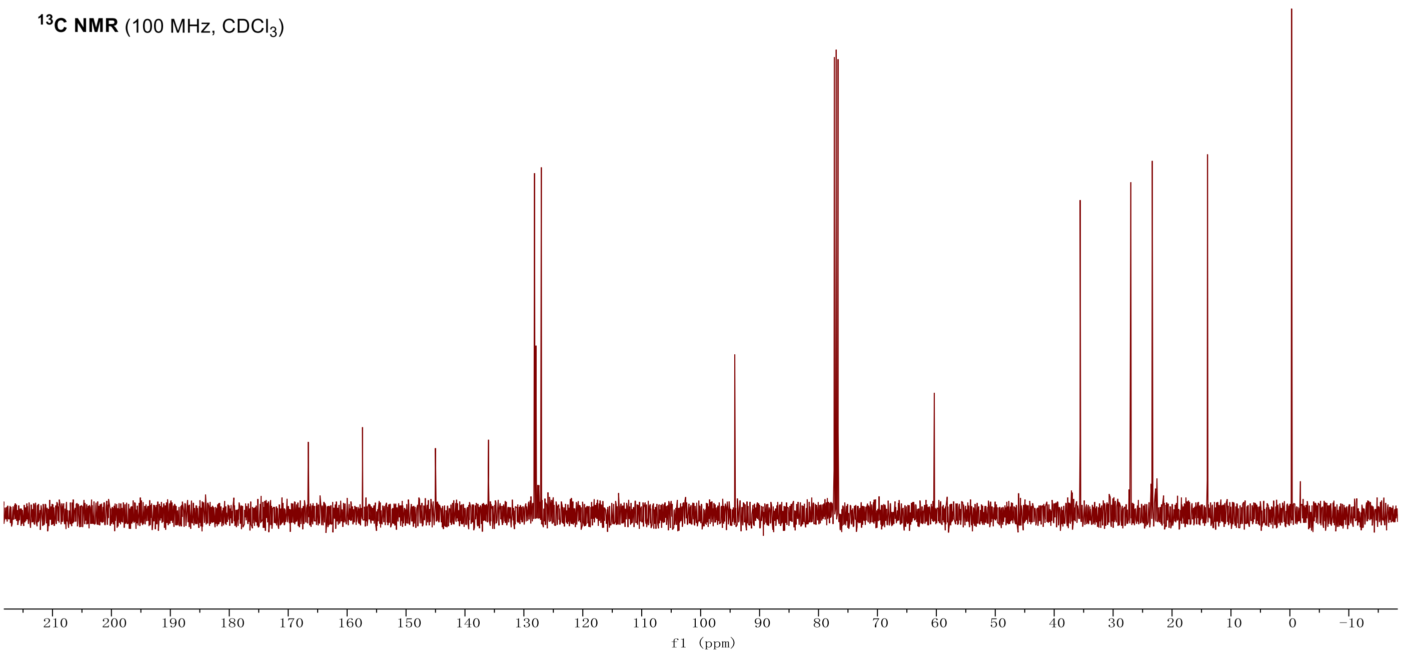

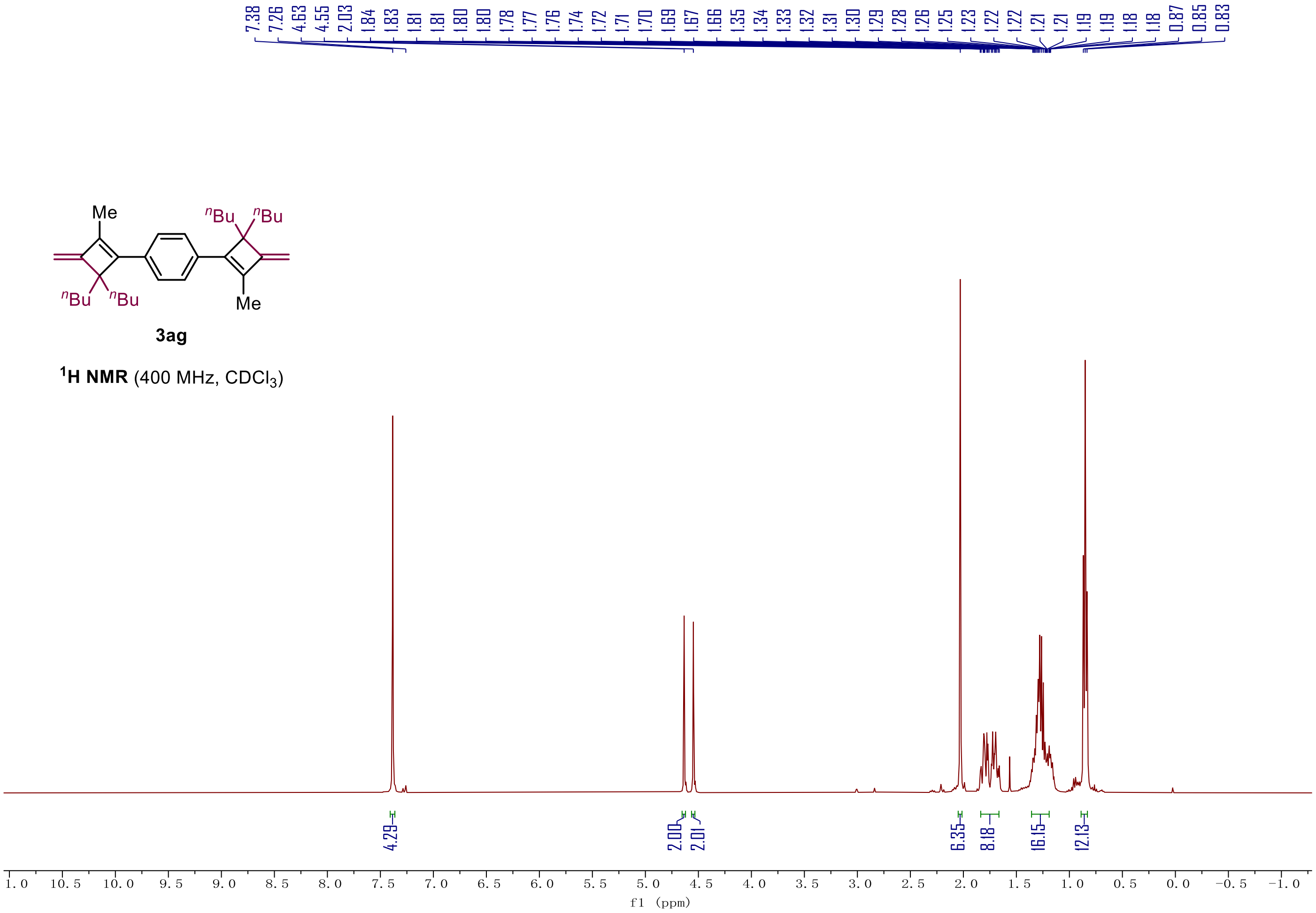
7.38 7.26 4.63 4.55 2.03 1.84 1.83 1.81 1.81 1.80 1.80 1.78 1.77 1.76 1.74 1.72 1.71 1.70 1.69 1.67 1.66 1.35 1.34 1.33 1.32 1.31 1.30 1.29 1.28 1.26 1.25 1.23 1.22 1.22 1.21 1.21 1.19 1.19 1.18 1.18 0.87 0.85 0.83
Me
ⁿBu ⁿBu
ⁿBu ⁿBu
Me
3ag
^{1}H NMR (400 MHz, $CDCl_3$)
4.29
2.00
2.01
6.35
8.18
16.15
12.13
f1 (ppm)

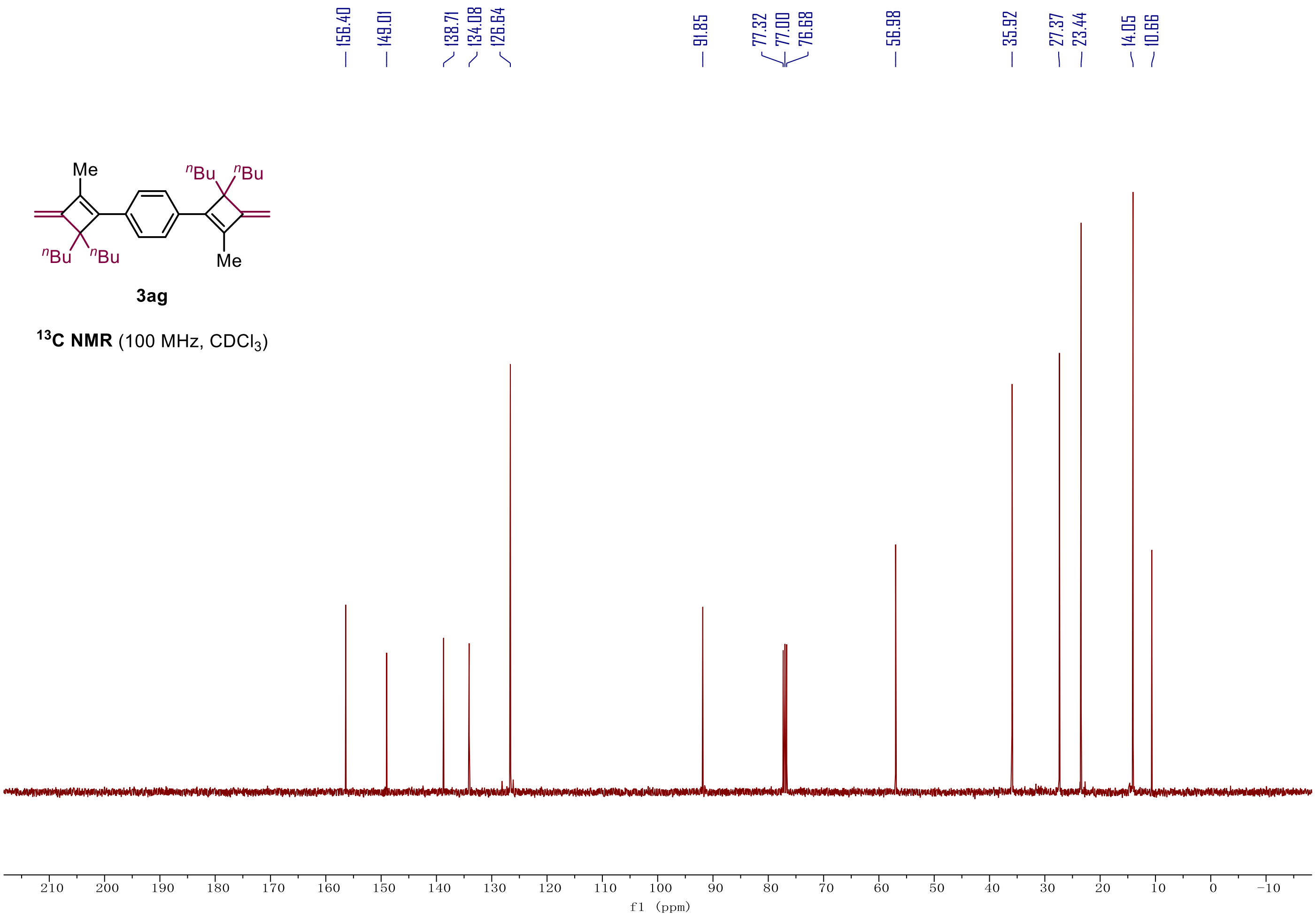
156.40
149.01
138.71
134.08
126.64
91.85
77.32
77.00
76.68
56.98
35.92
27.37
23.44
14.05
10.66
Me
ⁿBu ⁿBu
ⁿBu ⁿBu
Me
3ag
^{13}C NMR (100 MHz, $CDCl_3$)
f1 (ppm)

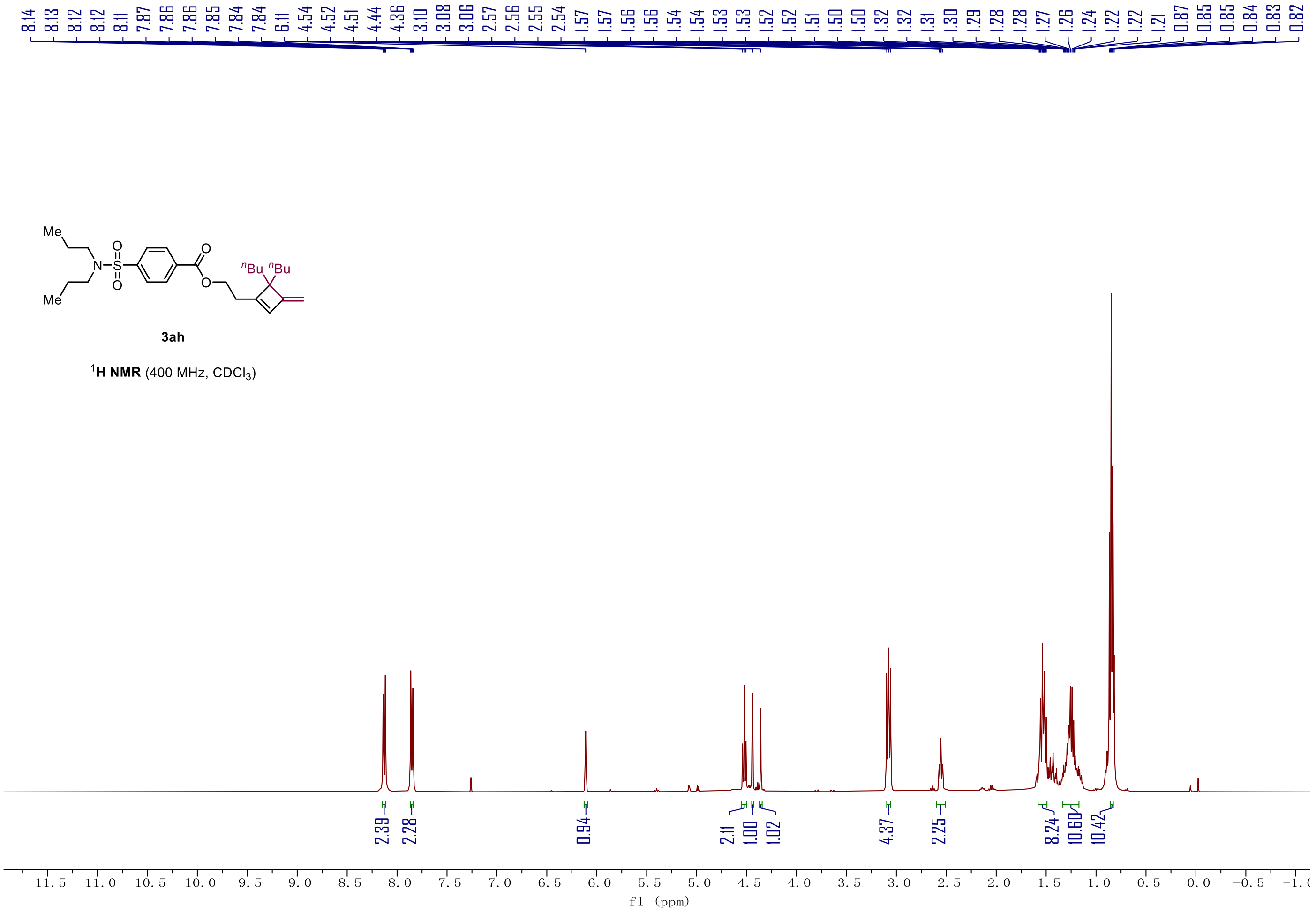

3ah

^{1}H NMR (400 MHz, $CDCl_3$)

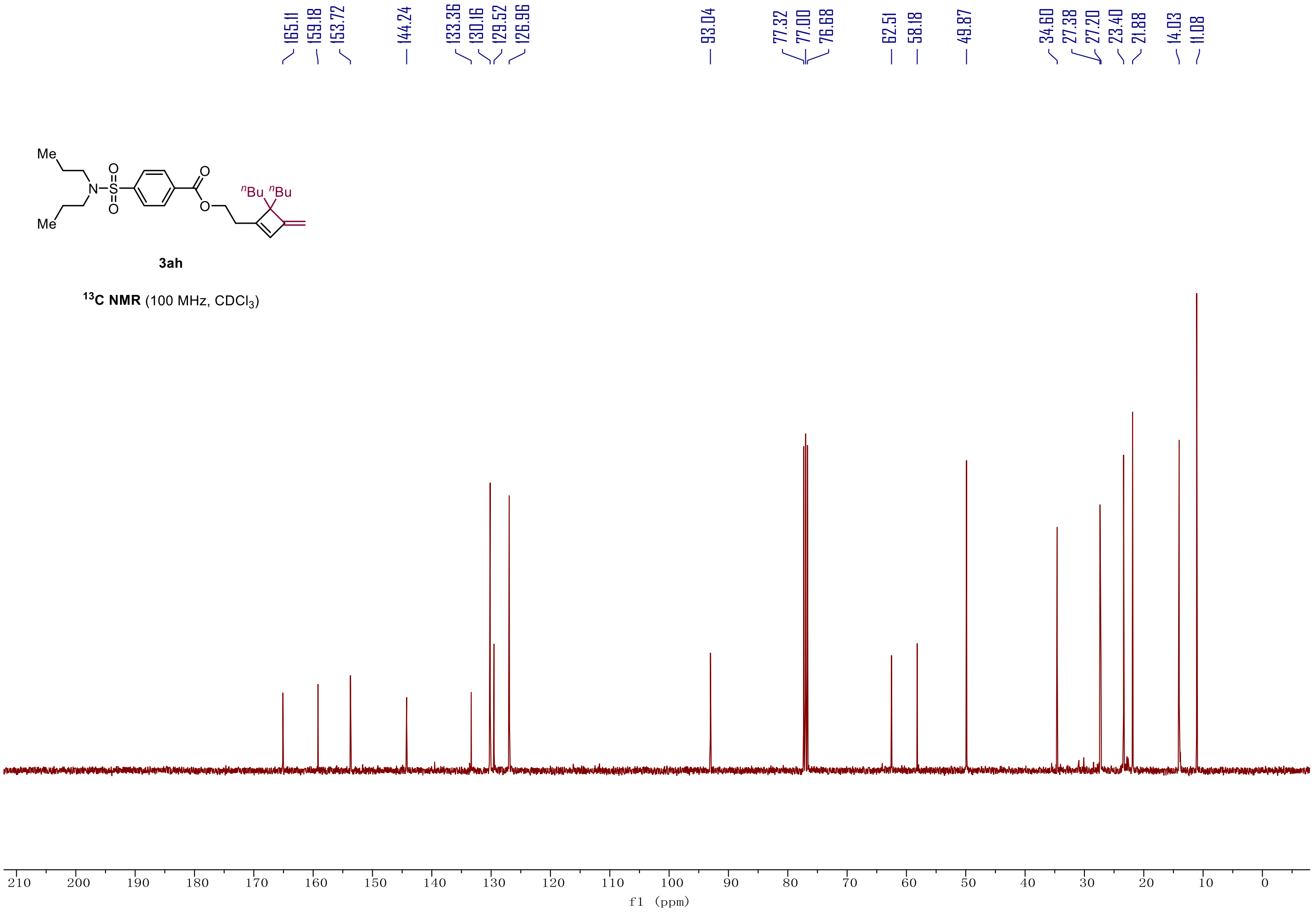

3ah

^{13}C NMR (100 MHz, $CDCl_3$)

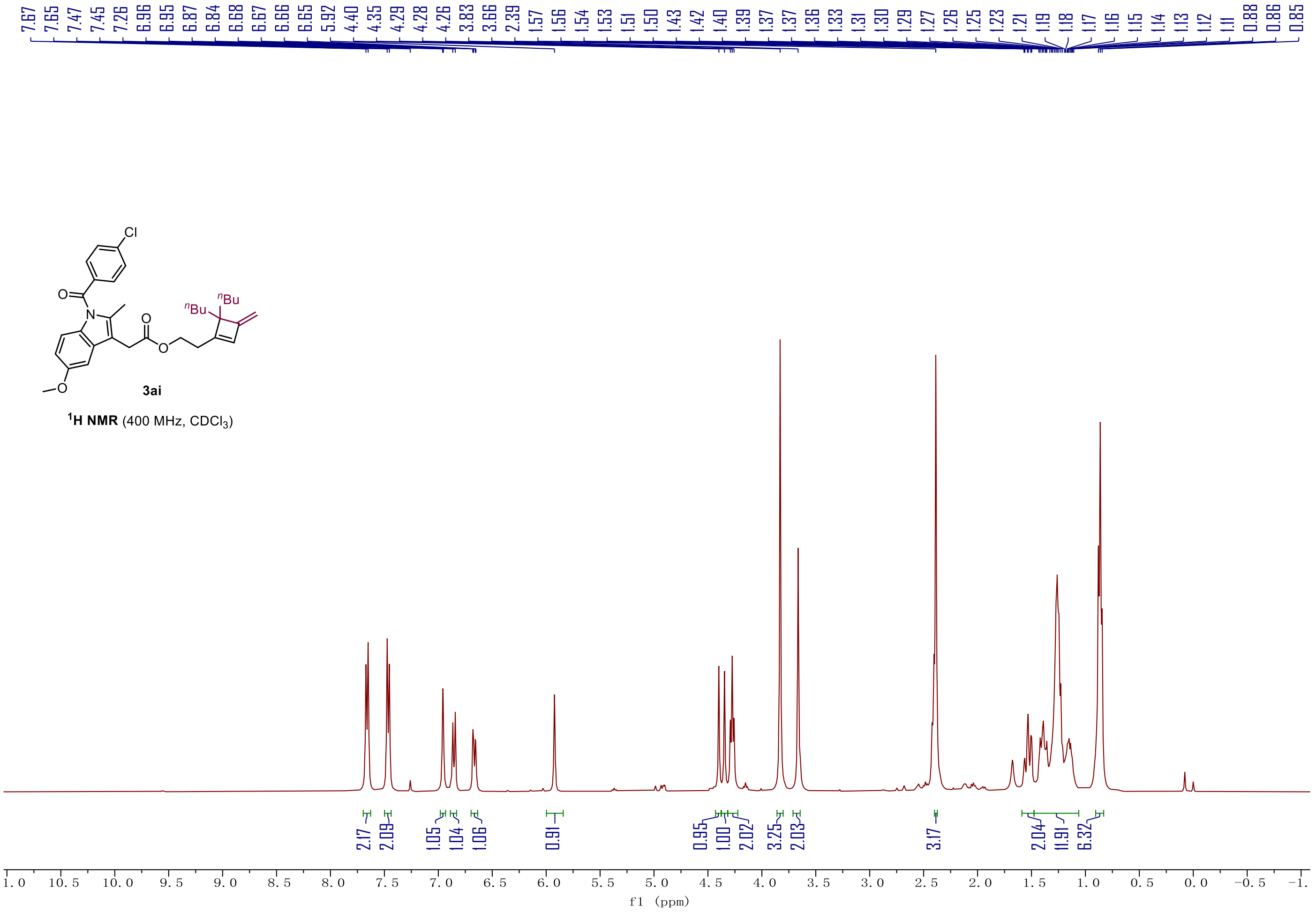

3ai

^{1}H NMR (400 MHz, $CDCl_3$)

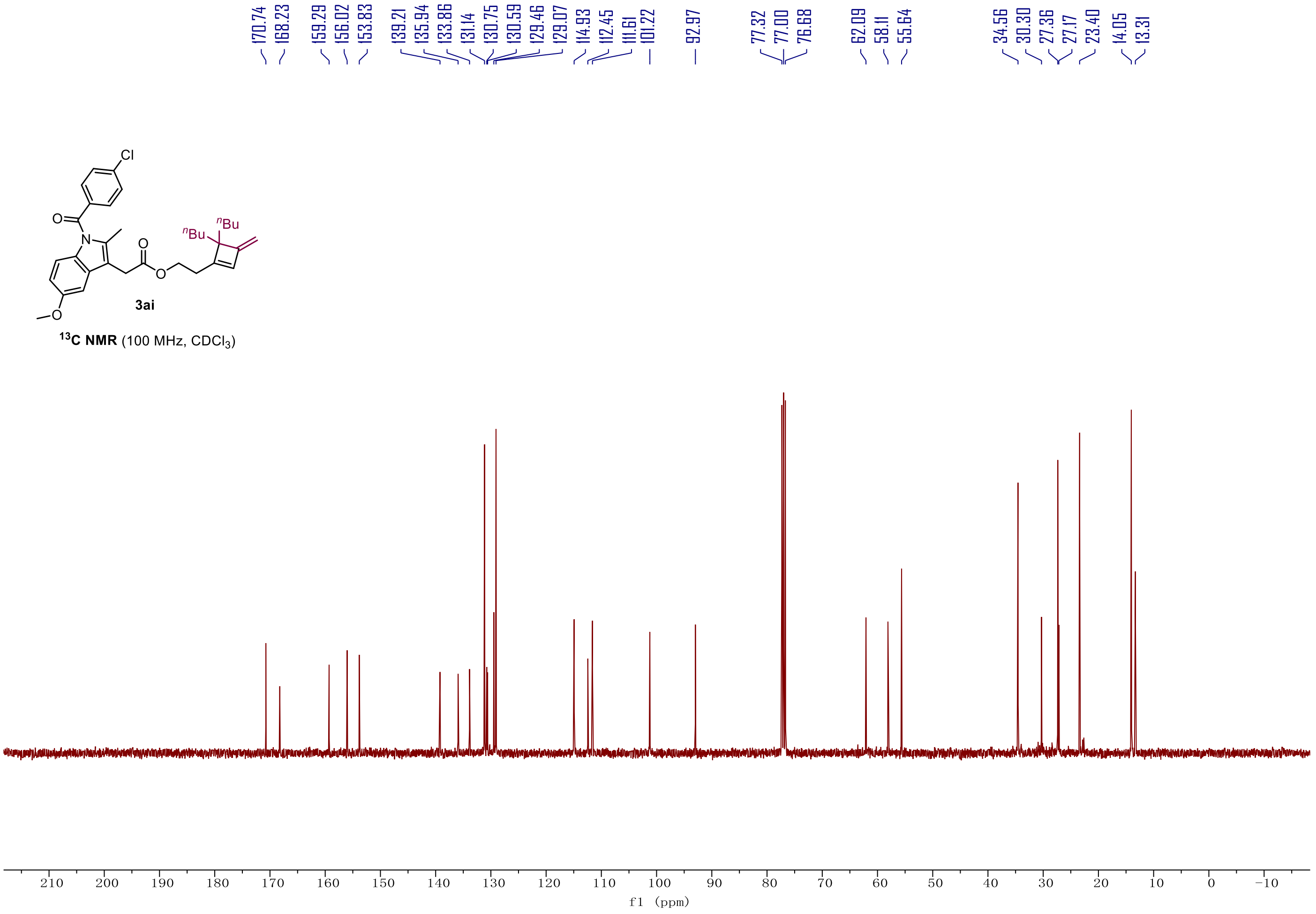

3ai

^{13}C NMR (100 MHz, $CDCl_3$)

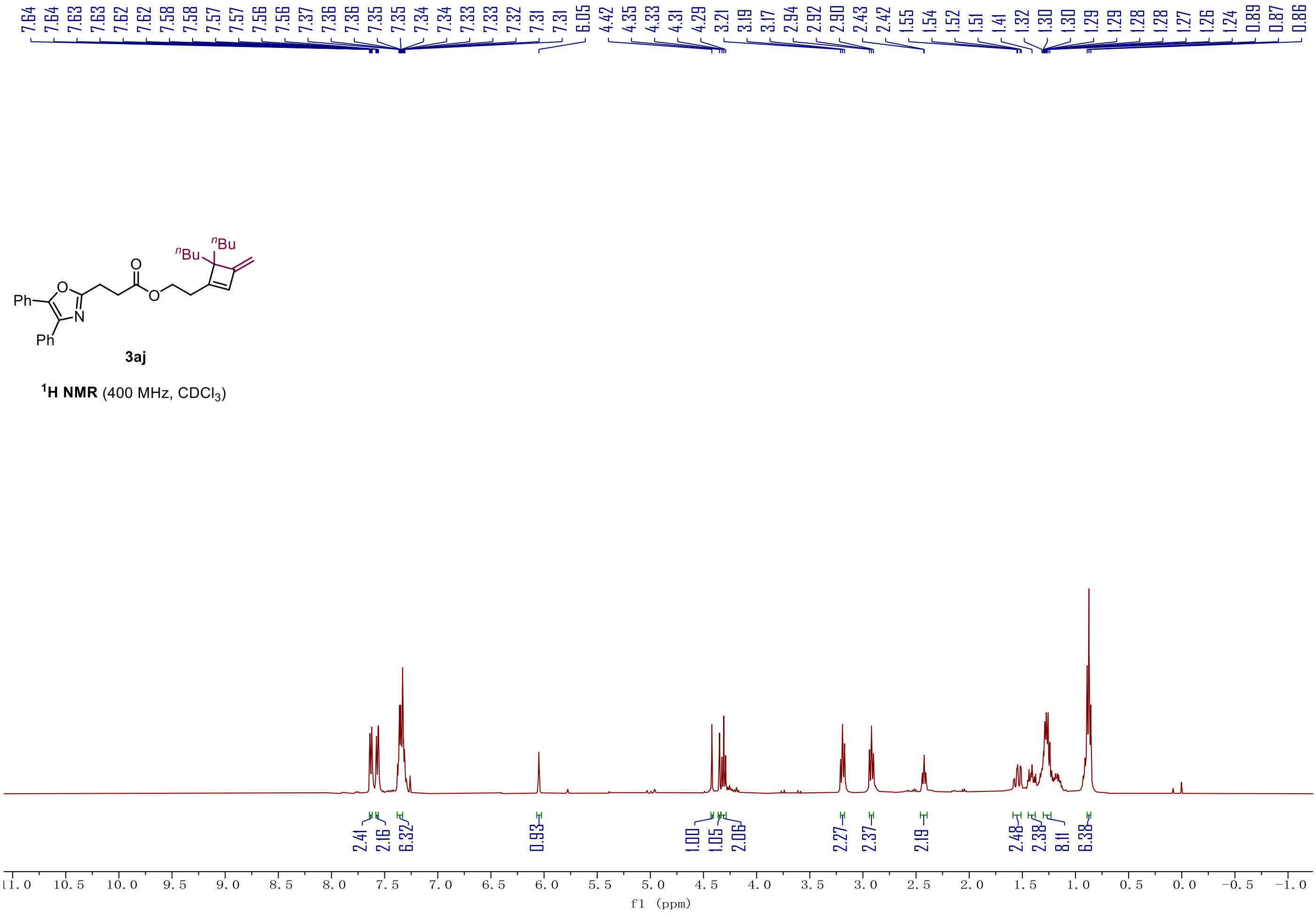
7.64 7.64 7.63 7.63 7.62 7.62 7.58 7.58 7.57 7.57 7.56 7.56 7.37 7.36 7.36 7.35 7.35 7.34 7.34 7.33 7.33 7.32 7.31 7.31 6.05 4.42 4.35 4.33 4.31 4.29 3.21 3.19 3.17 2.94 2.92 2.90 2.43 2.42 1.55 1.54 1.52 1.51 1.41 1.32 1.30 1.30 1.29 1.29 1.28 1.28 1.27 1.26 1.24 0.89 0.87 0.86
nBu
nBu
O
Ph
O
N
O
Ph
3aj
1H NMR (400 MHz, CDCl3)
2.41 2.16 6.32 0.93 1.00 1.05 2.06 2.27 2.37 2.19 2.48 2.38 8.11 6.38
11.0 10.5 10.0 9.5 9.0 8.5 8.0 7.5 7.0 6.5 6.0 5.5 5.0 4.5 4.0 3.5 3.0 2.5 2.0 1.5 1.0 0.5 0.0 -0.5 -1.0
f1 (ppm)

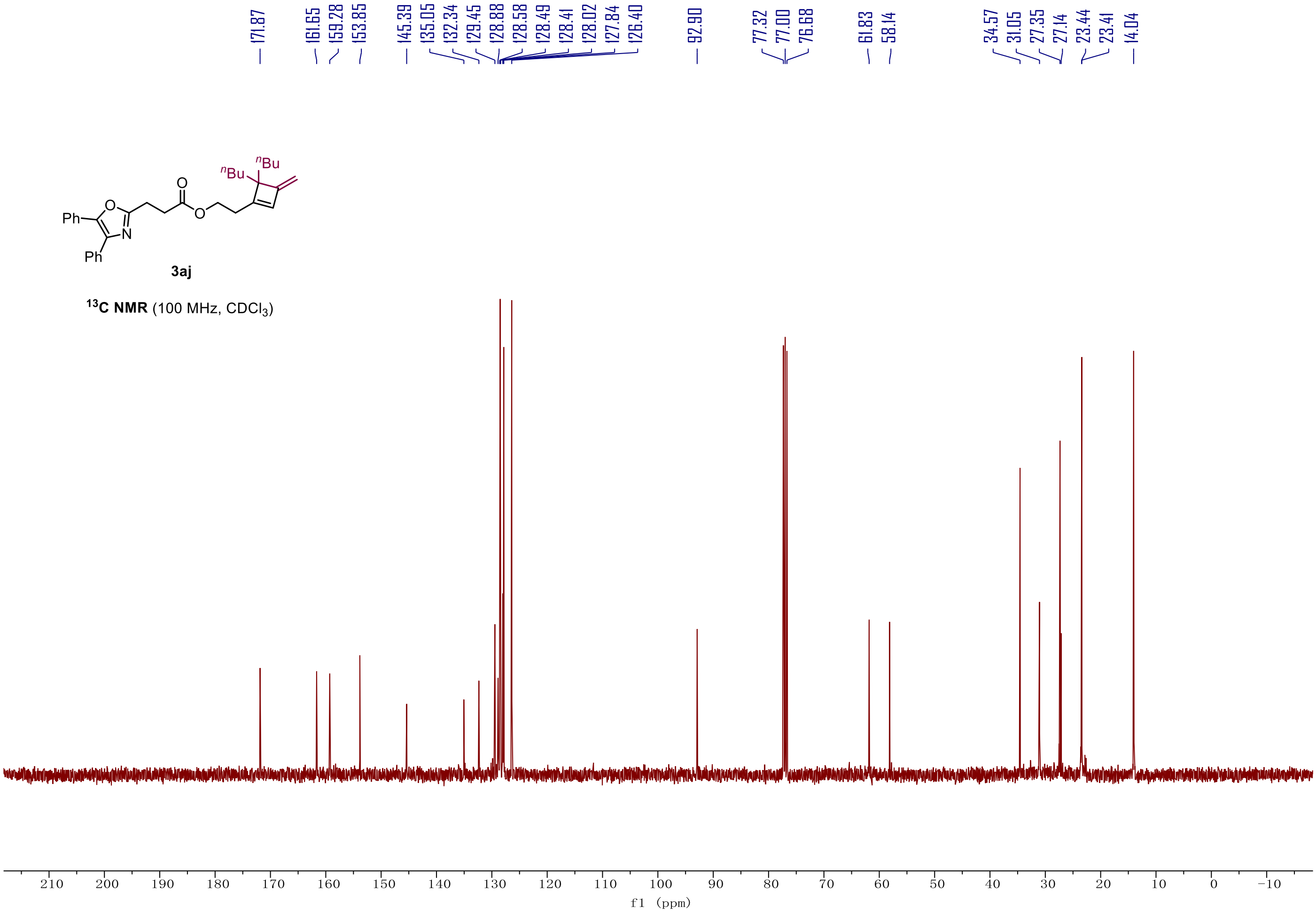
171.87 161.65 159.28 153.85 145.39 135.05 132.34 129.45 128.88 128.58 128.49 128.41 128.02 127.84 126.40 92.90 77.32 77.00 76.68 61.83 58.14 34.57 31.05 27.35 27.14 23.44 23.41 14.04
nBu
nBu
O
Ph
O
N
O
Ph
3aj
13C NMR (100 MHz, CDCl3)
210 200 190 180 170 160 150 140 130 120 110 100 90 80 70 60 50 40 30 20 10 0 -10
f1 (ppm)

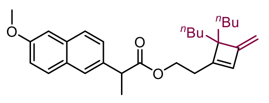

3ak

^{1}H NMR (400 MHz, $CDCl_3$)

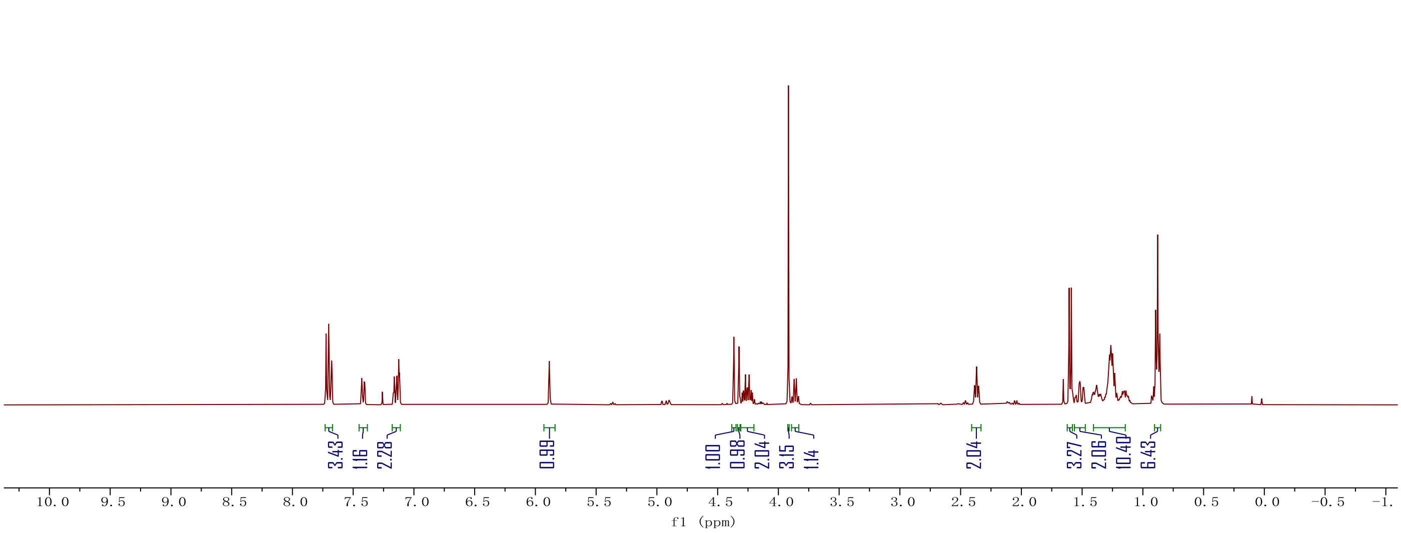

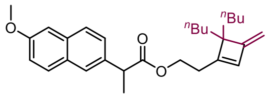

3ak

^{13}C NMR (100 MHz, $CDCl_3$)

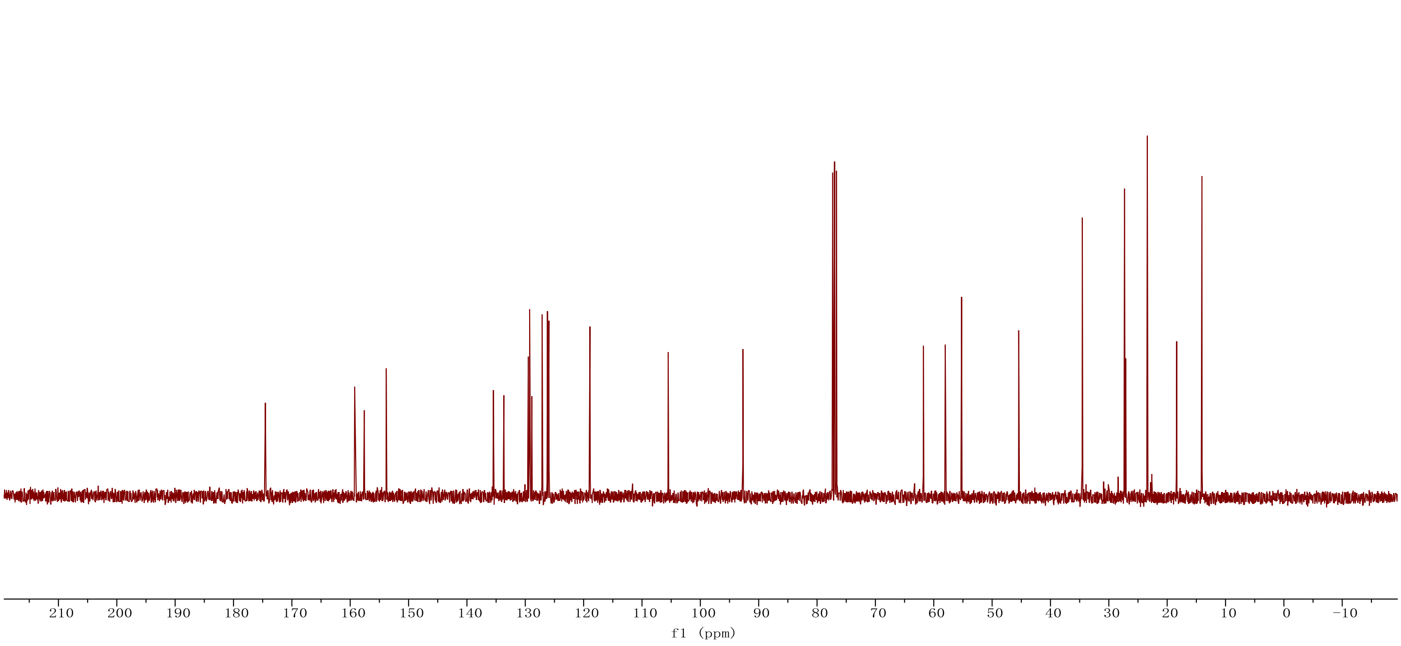

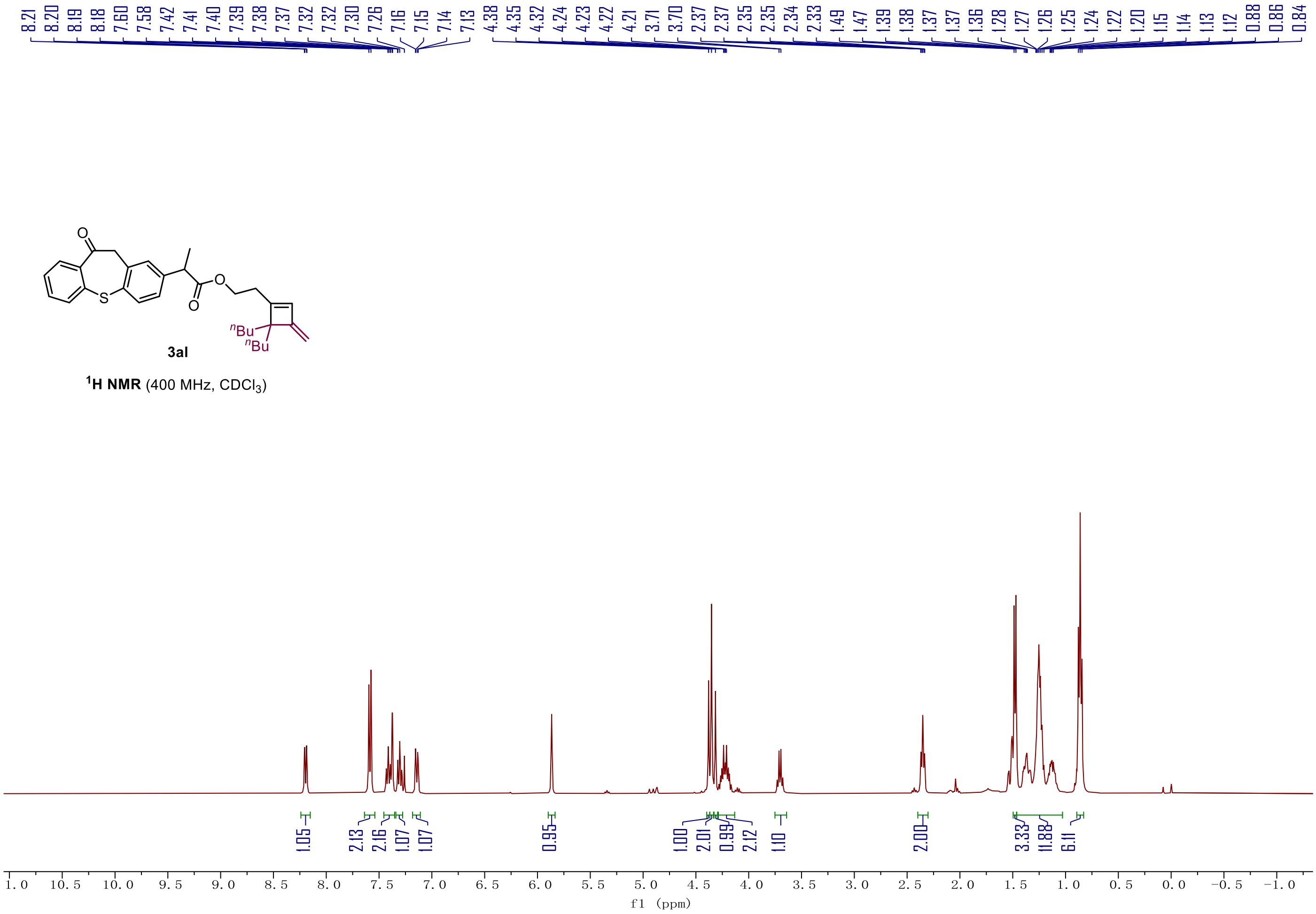

3al

^{1}H NMR (400 MHz, $CDCl_3$)

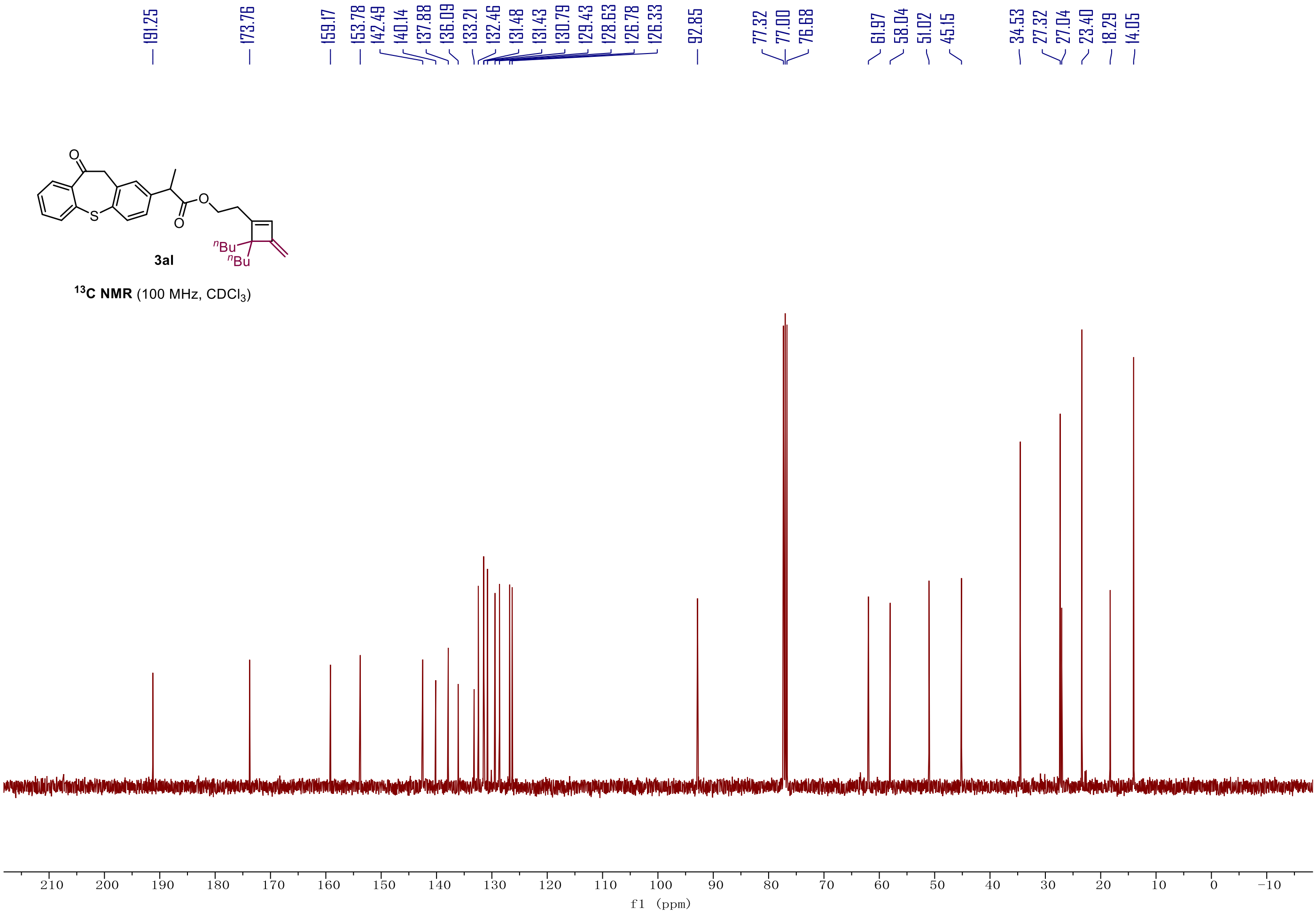

3al

^{13}C NMR (100 MHz, $CDCl_3$)

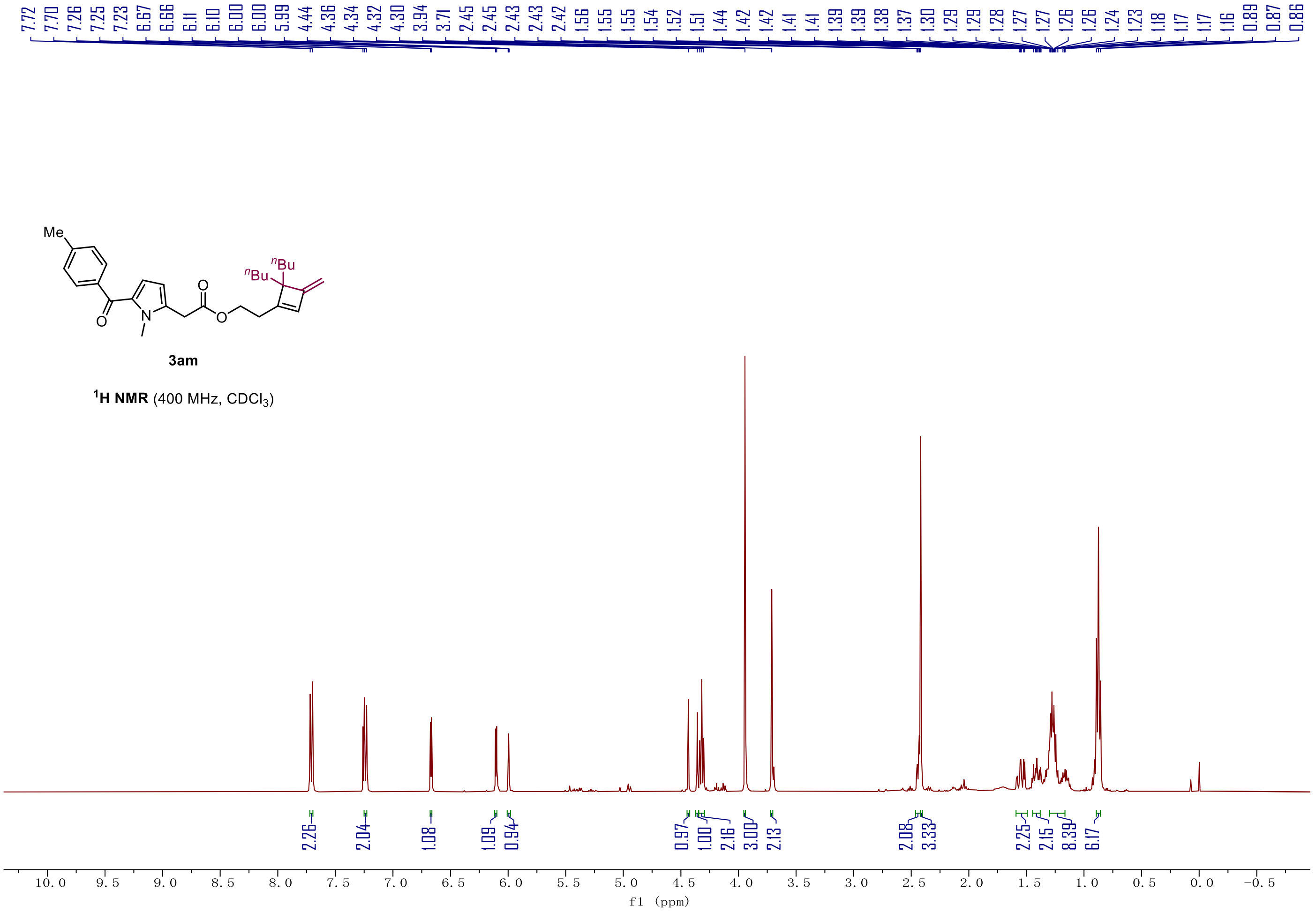
7.72 7.70 7.26 7.25 7.23 6.67 6.66 6.11 6.10 6.00 6.00 5.99 4.44 4.36 4.34 4.32 4.30 3.94 3.71 2.45 2.45 2.43 2.43 2.42 1.56 1.55 1.55 1.54 1.52 1.51 1.44 1.42 1.42 1.41 1.41 1.39 1.39 1.38 1.37 1.30 1.29 1.29 1.28 1.27 1.27 1.26 1.26 1.24 1.23 1.18 1.17 1.17 1.16 0.89 0.87 0.86
Me
O
N
O
O
^{n}Bu
^{n}Bu
3am
^{1}H NMR (400 MHz, $CDCl_3$)
2.26 2.04 1.08 1.09 0.94 0.97 1.00 2.16 3.00 2.13 2.08 3.33 2.25 2.15 8.39 6.17
f1 (ppm)

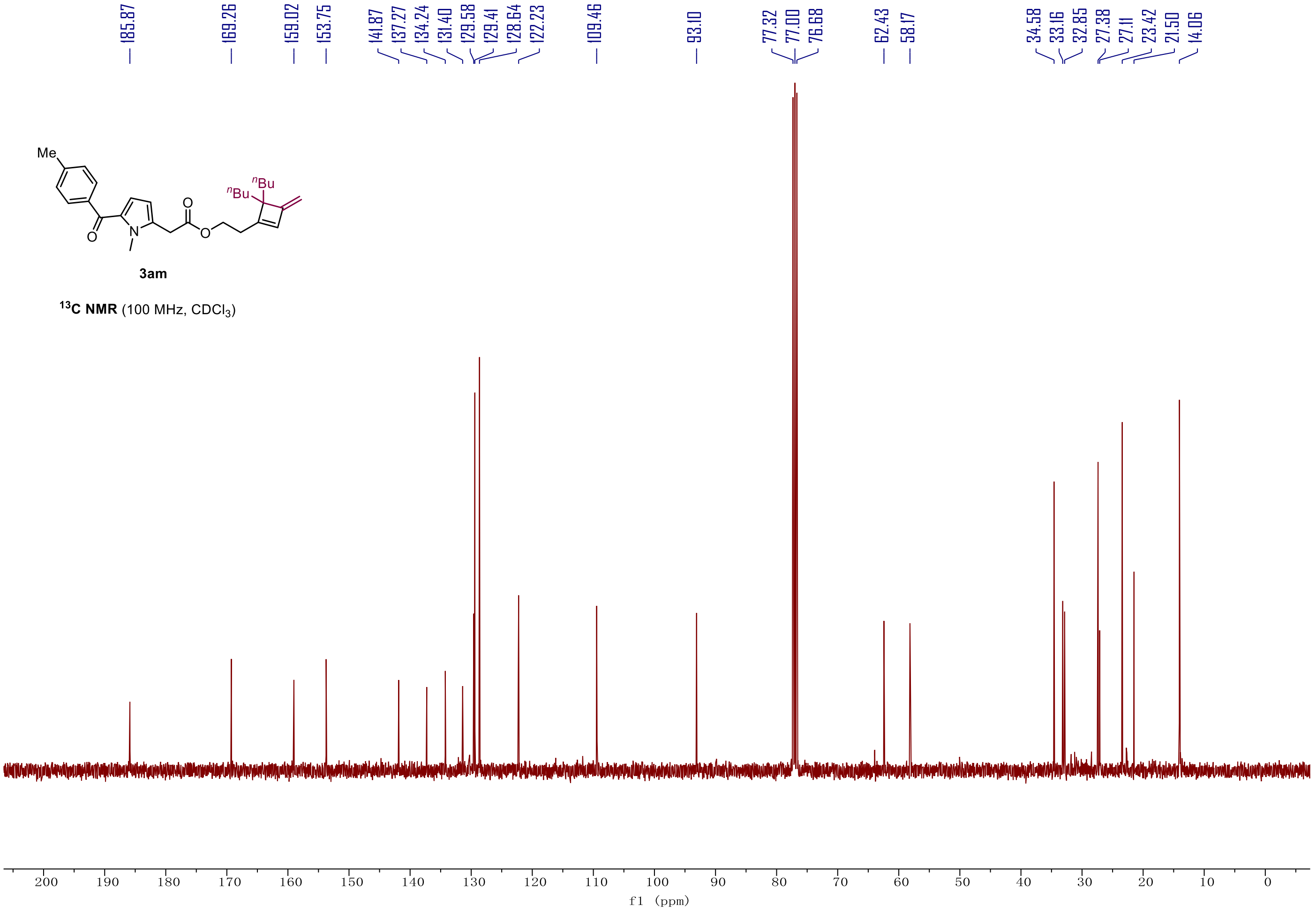
185.87 169.26 159.02 153.75 141.87 137.27 134.24 131.40 129.58 129.41 128.64 122.23 109.46 93.10 77.32 77.00 76.68 62.43 58.17 34.58 33.16 32.85 27.38 27.11 23.42 21.50 14.06
Me
O
N
O
O
^{n}Bu
^{n}Bu
3am
^{13}C NMR (100 MHz, $CDCl_3$)
f1 (ppm)

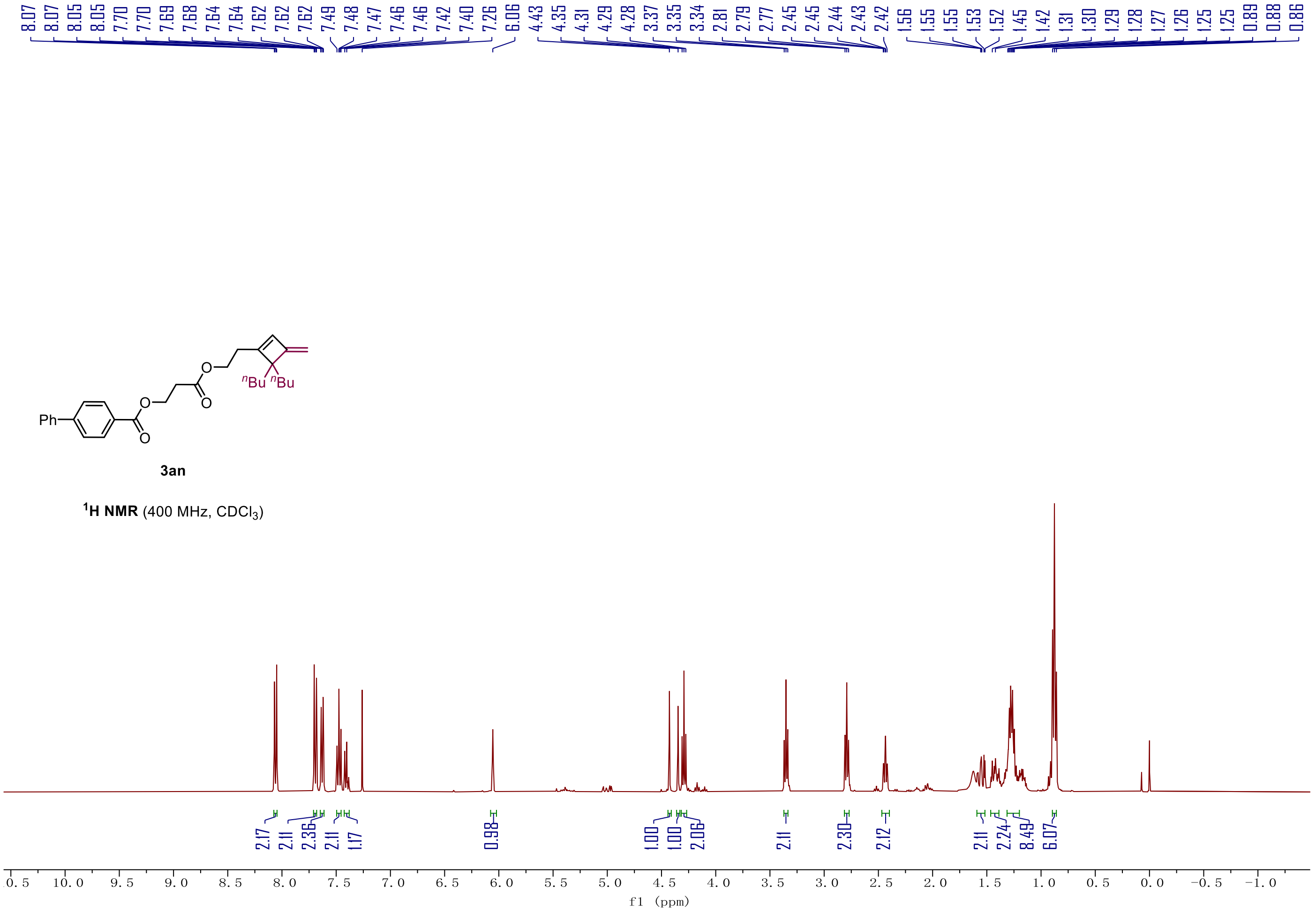

3an

^{1}H NMR (400 MHz, $CDCl_3$)

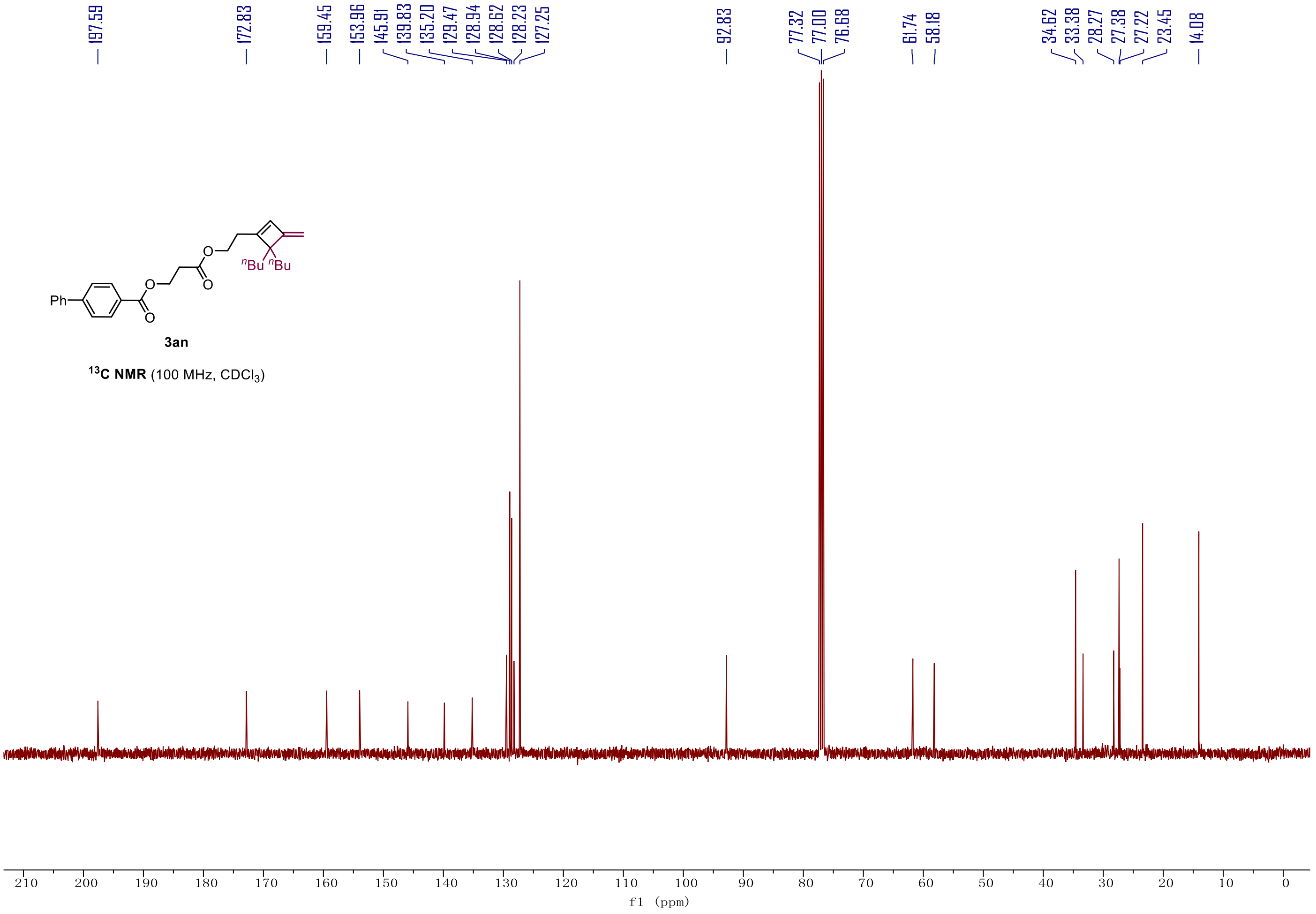

3an

^{13}C NMR (100 MHz, $CDCl_3$)

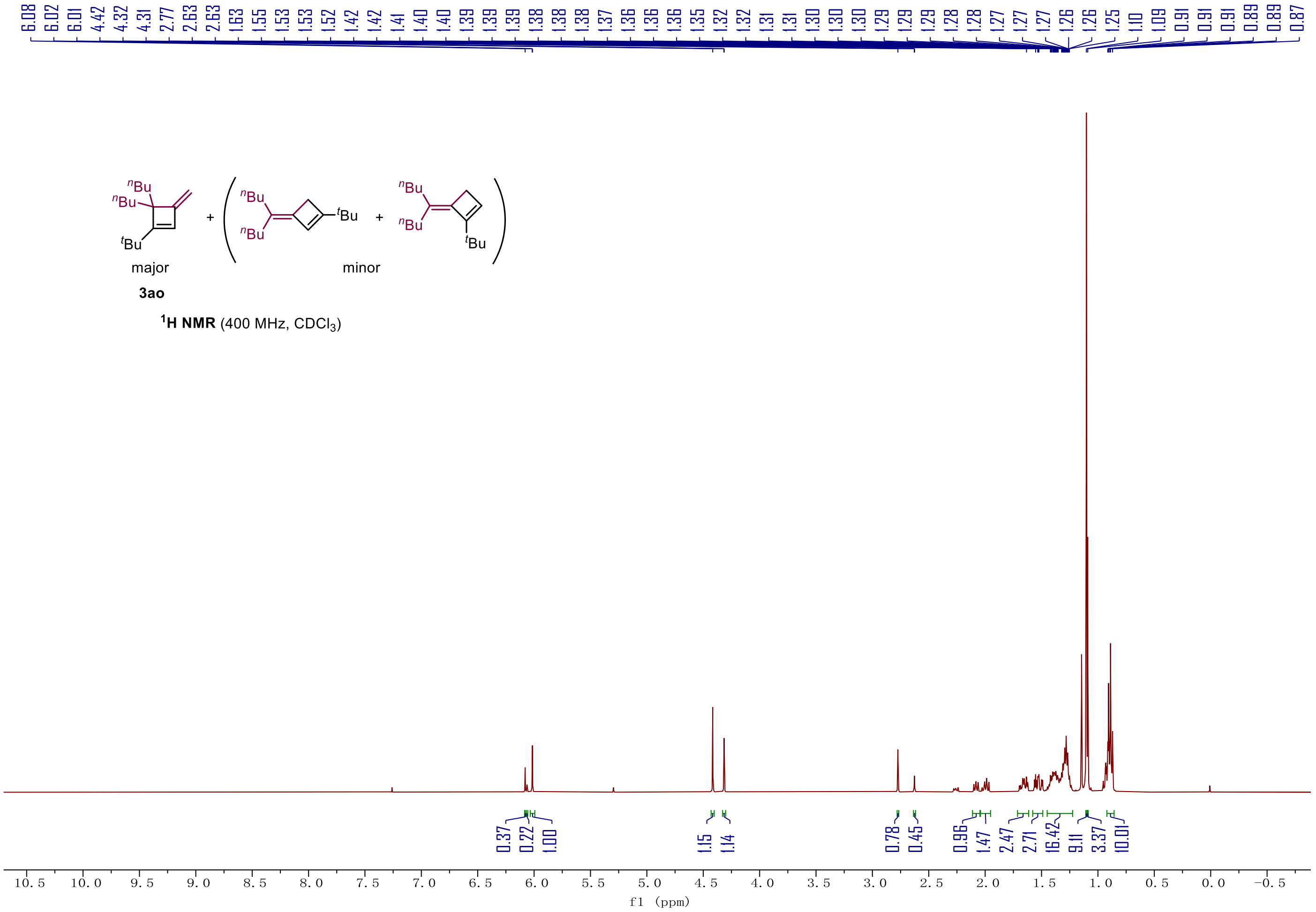
6.08 6.02 6.01 4.42 4.32 4.31 2.77 2.63 2.63 1.63 1.55 1.53 1.53 1.52 1.42 1.42 1.41 1.40 1.40 1.39 1.39 1.39 1.38 1.38 1.38 1.37 1.36 1.36 1.36 1.35 1.32 1.32 1.31 1.31 1.30 1.30 1.30 1.29 1.29 1.29 1.28 1.28 1.27 1.27 1.27 1.26 1.26 1.25 1.10 1.09 0.91 0.91 0.91 0.89 0.89 0.87
major
minor
3ao
1H NMR (400 MHz, CDCl3)
0.37 0.22 1.00 1.15 1.14 0.78 0.45 0.96 1.47 2.47 2.71 16.42 9.11 3.37 10.01
f1 (ppm)

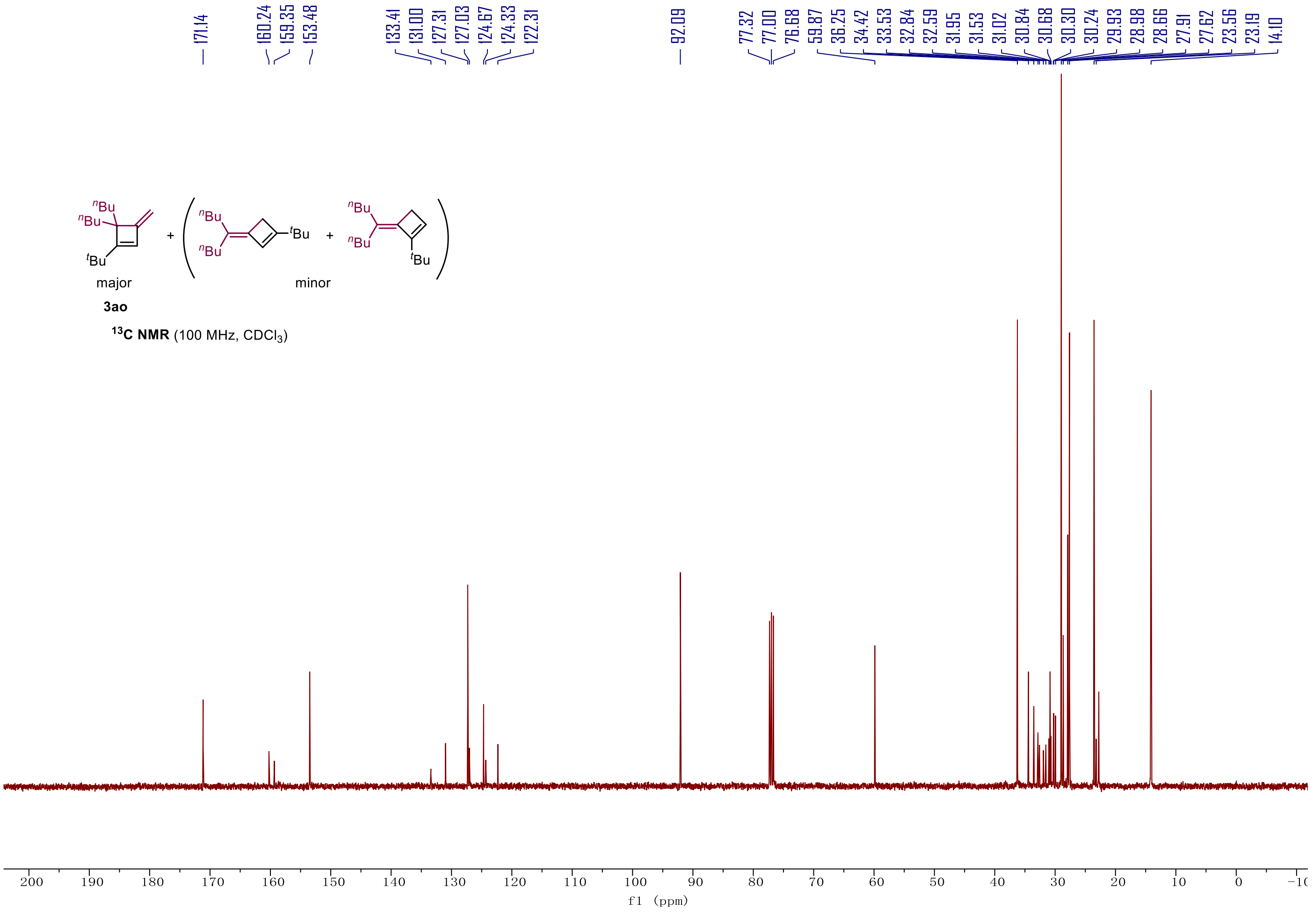
171.14 160.24 159.35 153.48 133.41 131.00 127.31 127.03 124.67 124.33 122.31 92.09 77.32 77.00 76.68 59.87 36.25 34.42 33.53 32.84 32.59 31.95 31.53 31.02 30.84 30.68 30.30 30.24 29.93 28.98 28.66 27.91 27.62 23.56 23.19 14.10
major
minor
3ao
13C NMR (100 MHz, CDCl3)
f1 (ppm)

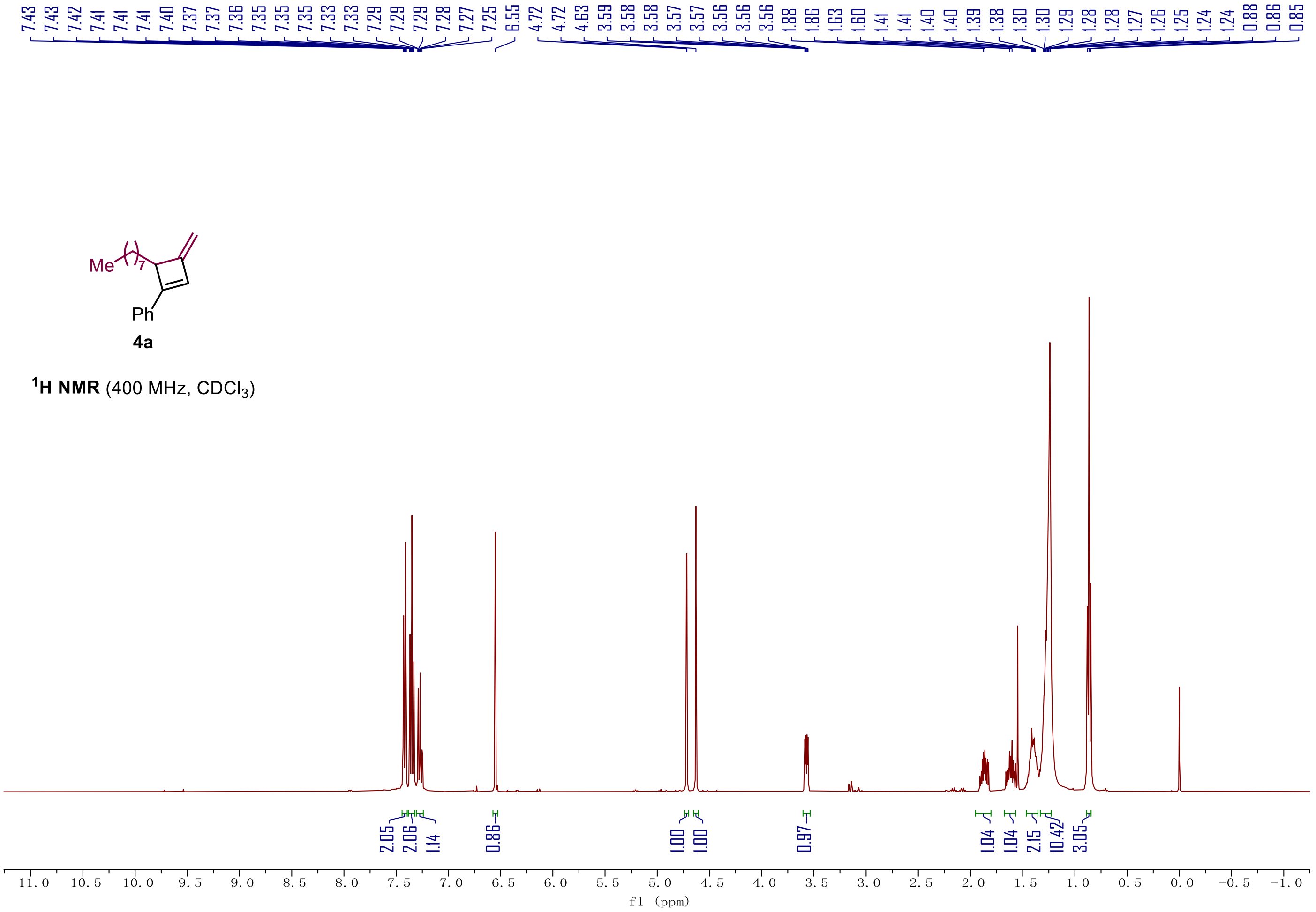
7.43 7.43 7.42 7.41 7.41 7.41 7.40 7.37 7.37 7.36 7.35 7.35 7.35 7.33 7.33 7.29 7.29 7.29 7.28 7.27 7.25 6.55 4.72 4.72 4.63 3.59 3.58 3.58 3.57 3.57 3.56 3.56 3.56 1.88 1.86 1.63 1.60 1.41 1.41 1.40 1.40 1.39 1.38 1.30 1.30 1.29 1.28 1.28 1.27 1.26 1.25 1.24 1.24 0.88 0.86 0.85
Me
7
Ph
4a
1H NMR (400 MHz, CDCl3)
2.05 2.06 1.14 0.86 1.00 1.00 0.97 1.04 1.04 2.15 10.42 3.05
f1 (ppm)

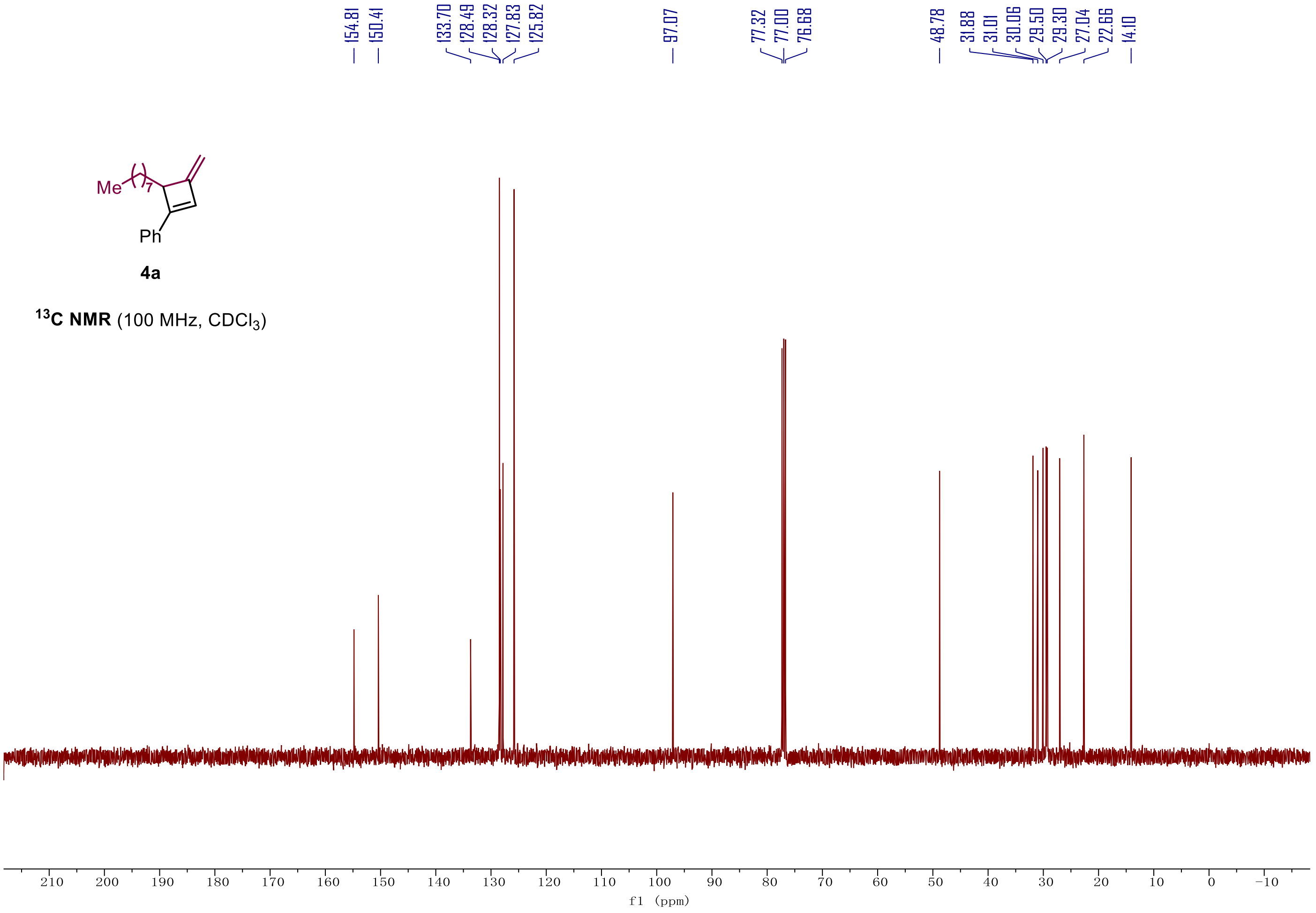
154.81 150.41 133.70 128.49 128.32 127.83 125.82 97.07 77.32 77.00 76.68 48.78 31.88 31.01 30.06 29.50 29.30 27.04 22.66 14.10
Me
7
Ph
4a
13C NMR (100 MHz, CDCl3)
f1 (ppm)

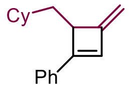

4b

[1]**H NMR** (400 MHz, $CDCl_3$)

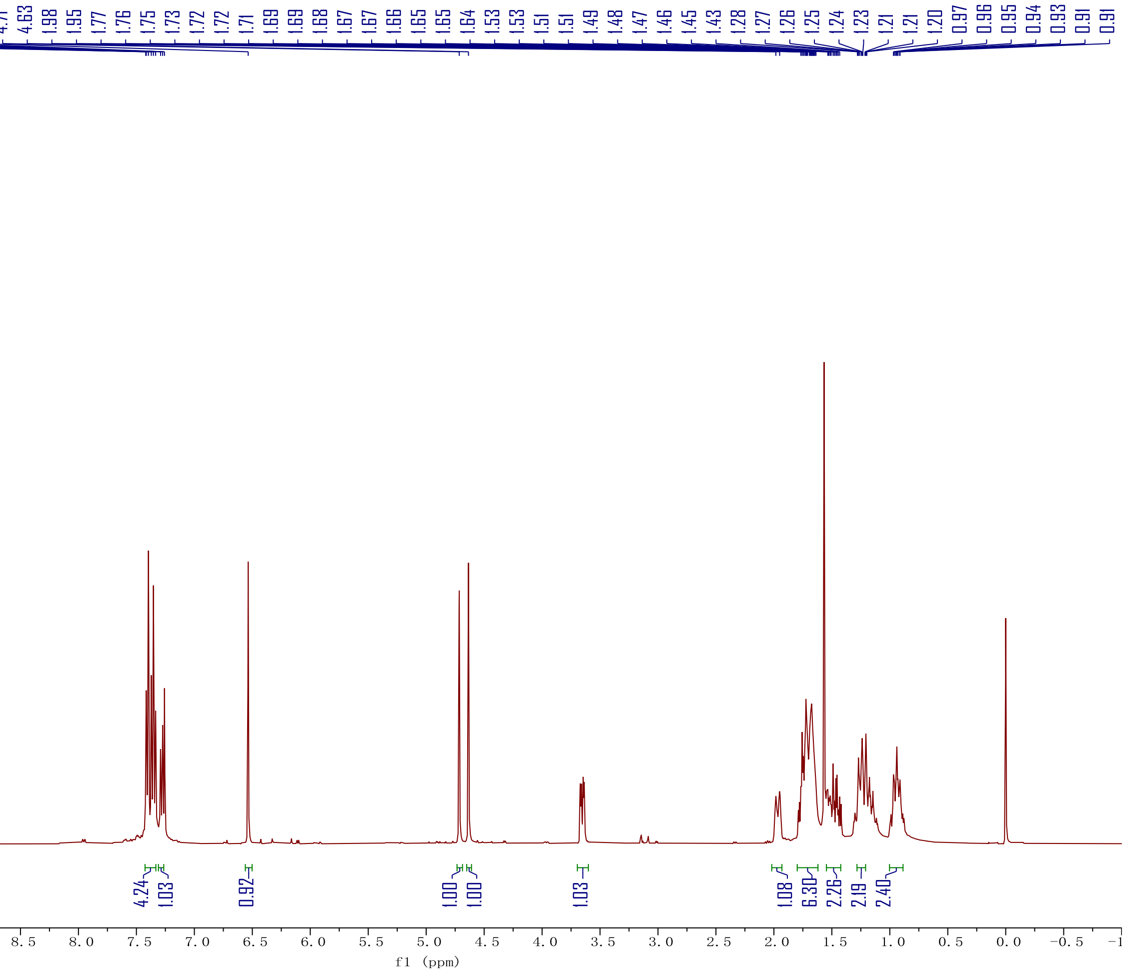

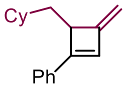

4b

[13]**C NMR** (100 MHz, $CDCl_3$)

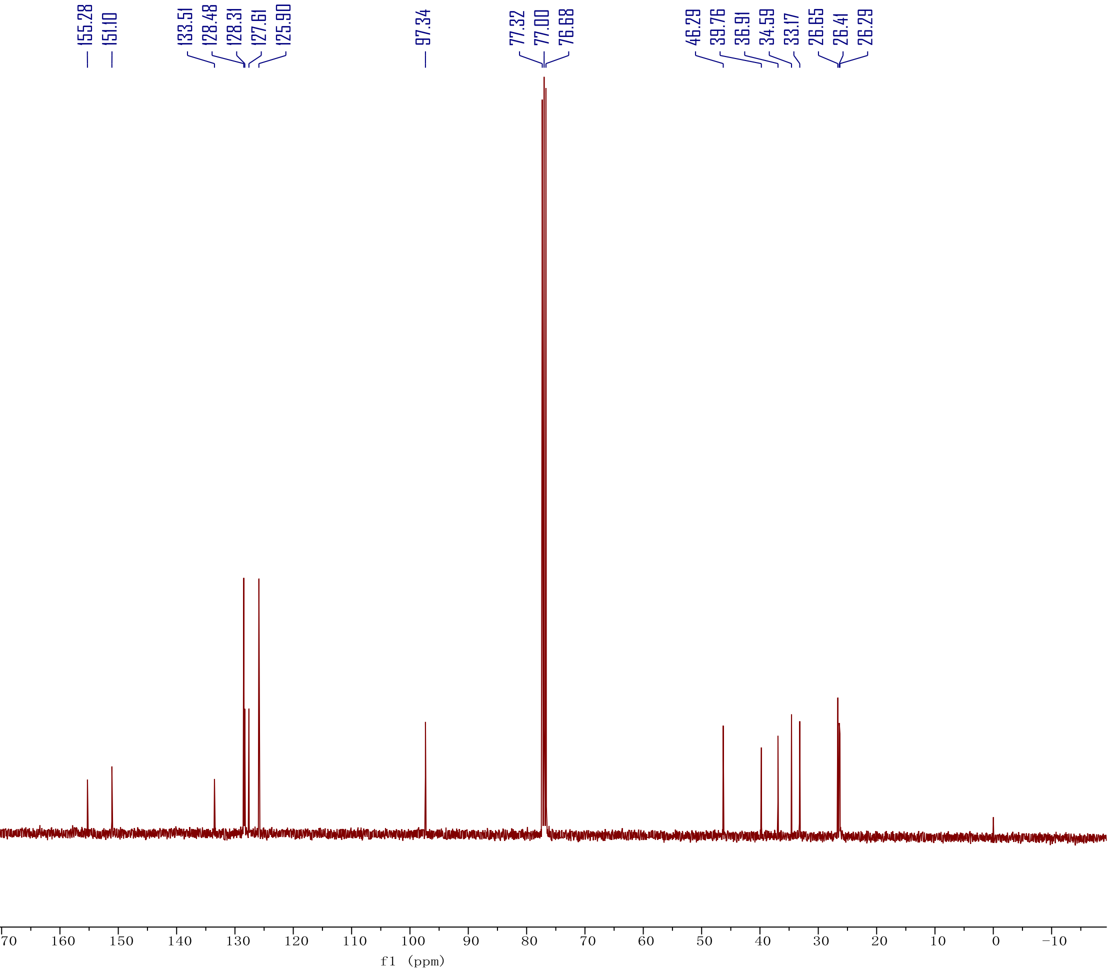

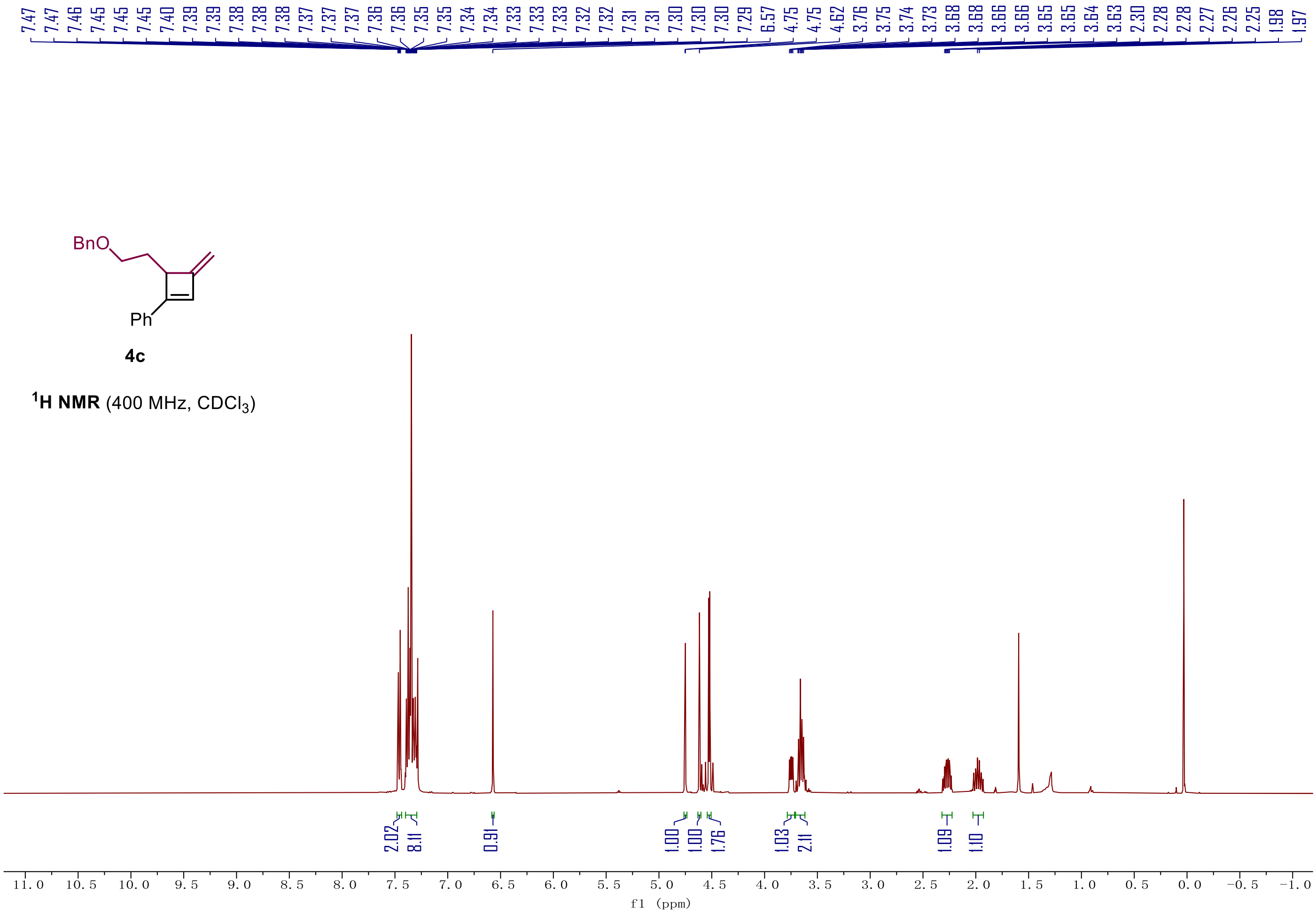

4c

^{1}H NMR (400 MHz, $CDCl_3$)

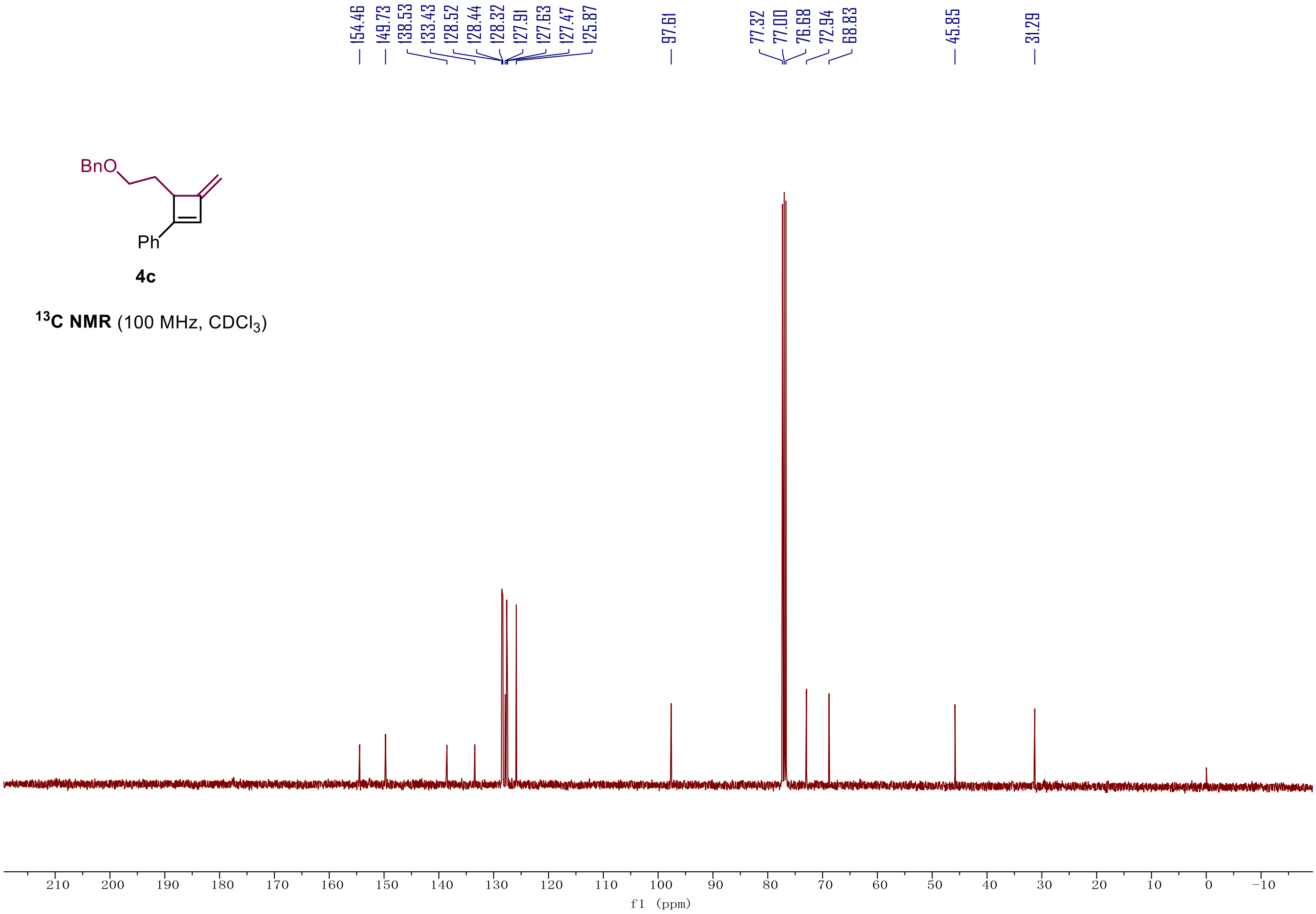

4c

^{13}C NMR (100 MHz, $CDCl_3$)

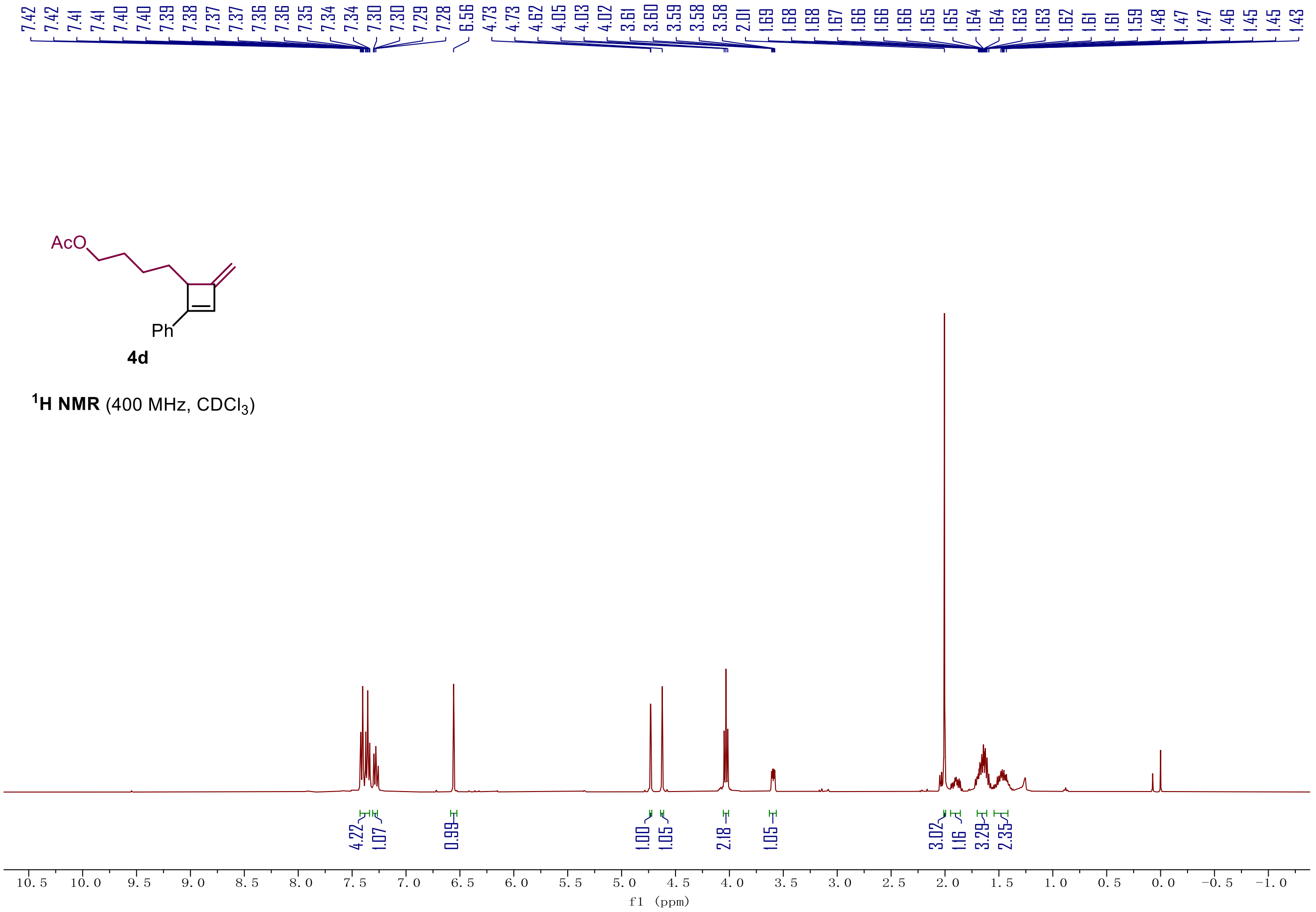
7.42 7.42 7.41 7.41 7.40 7.40 7.39 7.38 7.37 7.37 7.36 7.36 7.35 7.34 7.34 7.30 7.30 7.29 7.28 6.56 4.73 4.73 4.62 4.05 4.03 4.02 3.61 3.60 3.59 3.58 3.58 2.01 1.69 1.68 1.68 1.67 1.66 1.66 1.66 1.65 1.65 1.64 1.64 1.63 1.63 1.62 1.61 1.61 1.59 1.48 1.47 1.47 1.46 1.45 1.45 1.43
AcO
Ph
4d
1H NMR (400 MHz, CDCl3)
4.22 1.07 0.99 1.00 1.05 2.18 1.05 3.02 1.16 3.29 2.35
f1 (ppm)

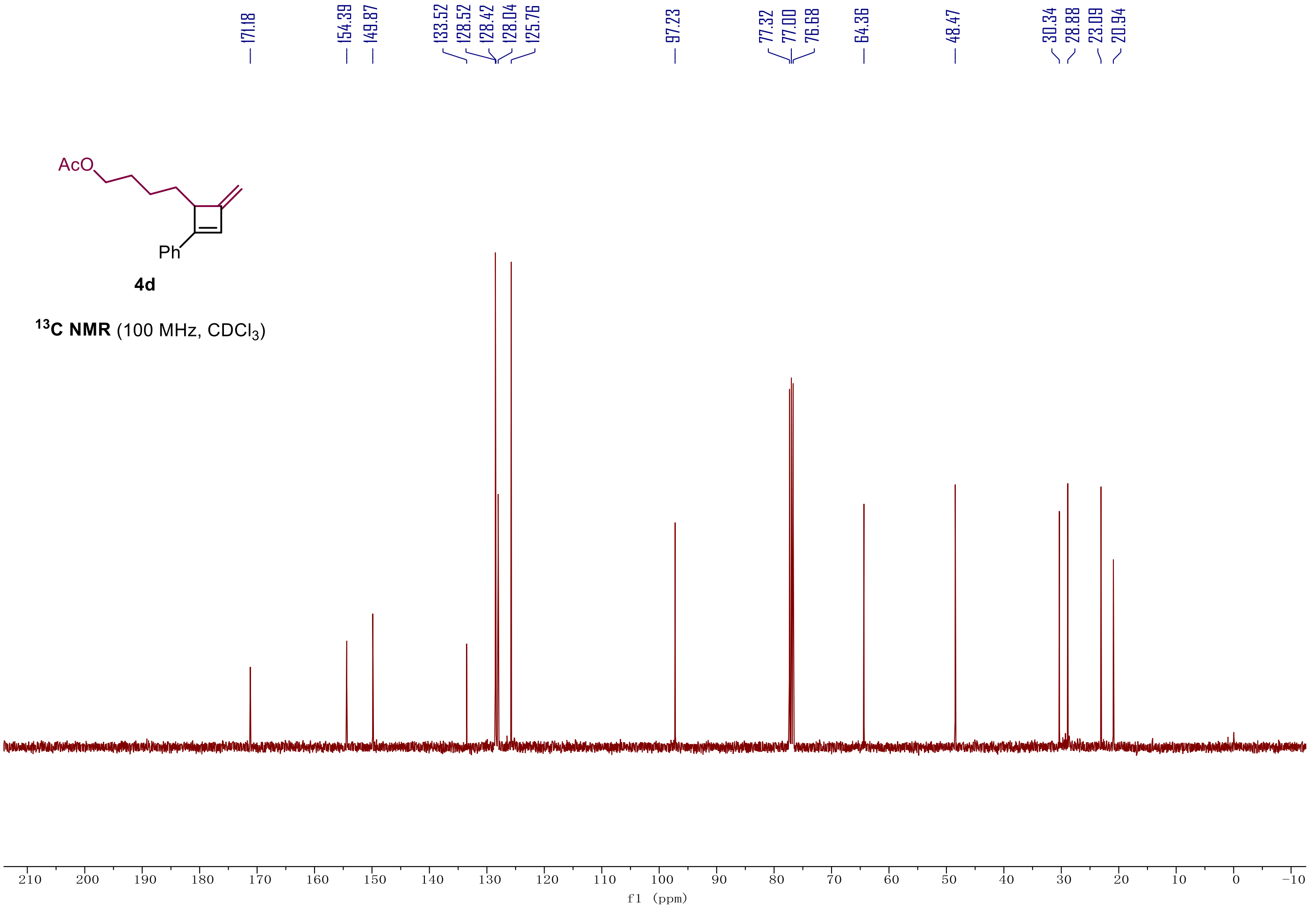
171.18 154.39 149.87 133.52 128.52 128.42 128.04 125.76 97.23 77.32 77.00 76.68 64.36 48.47 30.34 28.88 23.09 20.94
AcO
Ph
4d
13C NMR (100 MHz, CDCl3)
f1 (ppm)

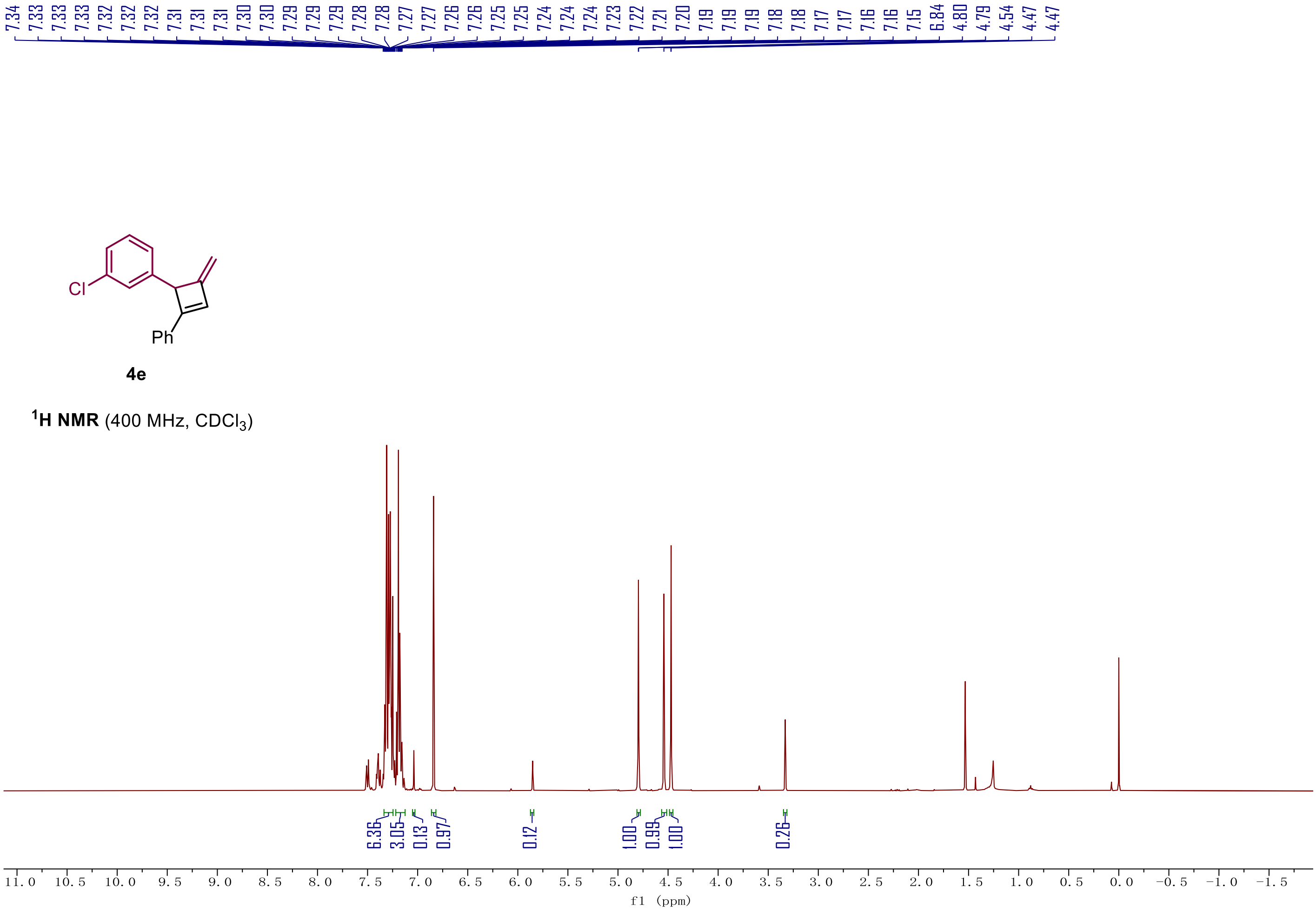

4e

^{1}H NMR (400 MHz, $CDCl_3$)

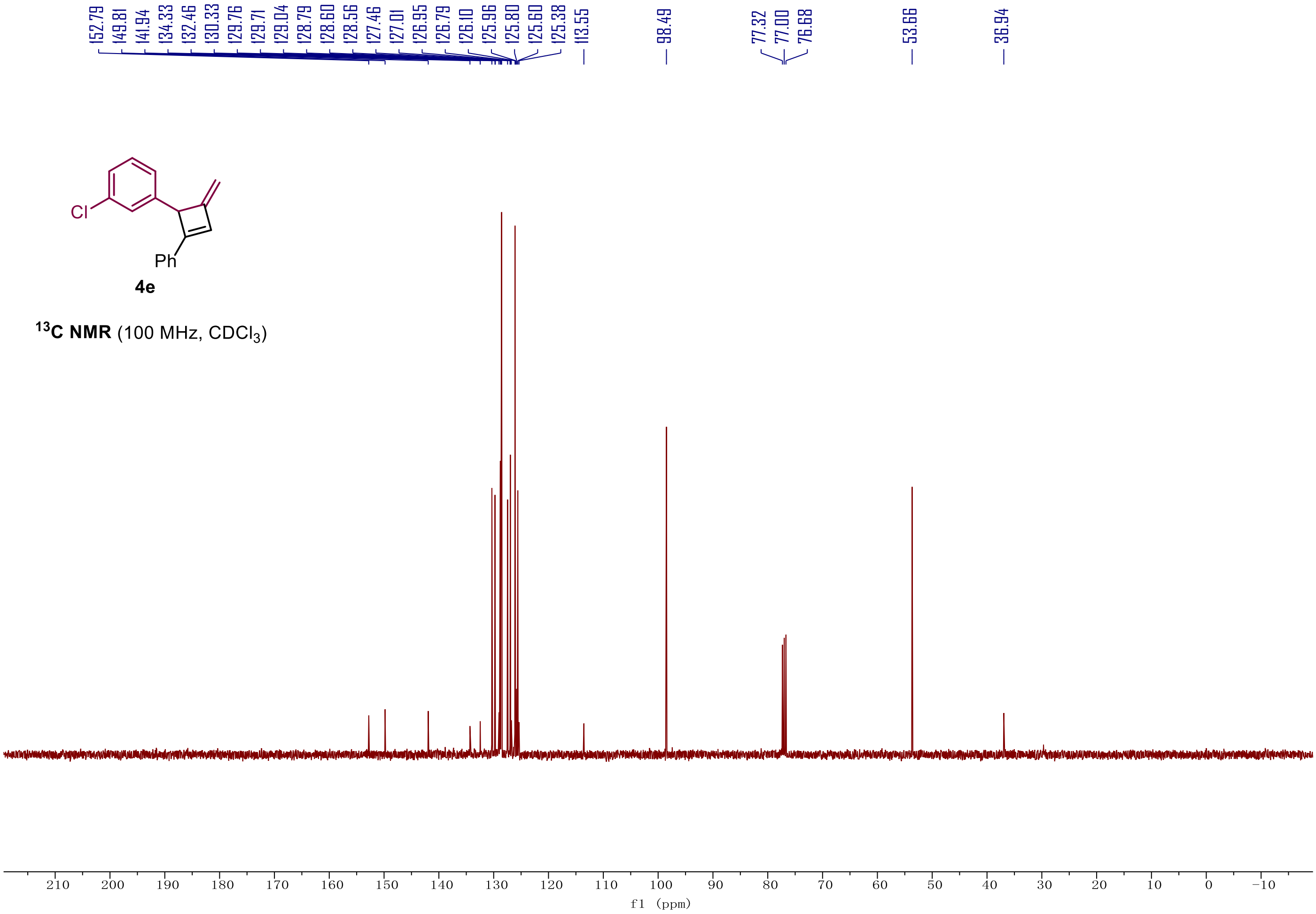

4e

^{13}C NMR (100 MHz, $CDCl_3$)

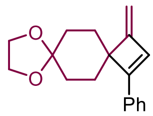

4f

^{1}H NMR (400 MHz, $CDCl_3$)

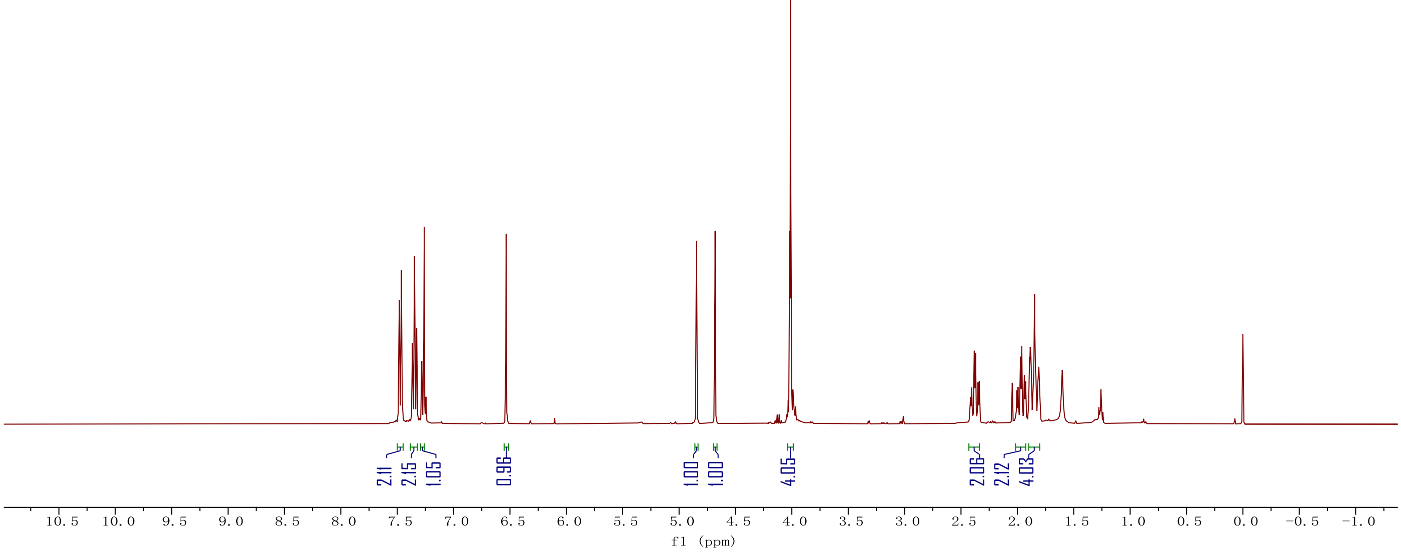

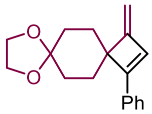

4f

^{13}C NMR (100 MHz, $CDCl_3$)

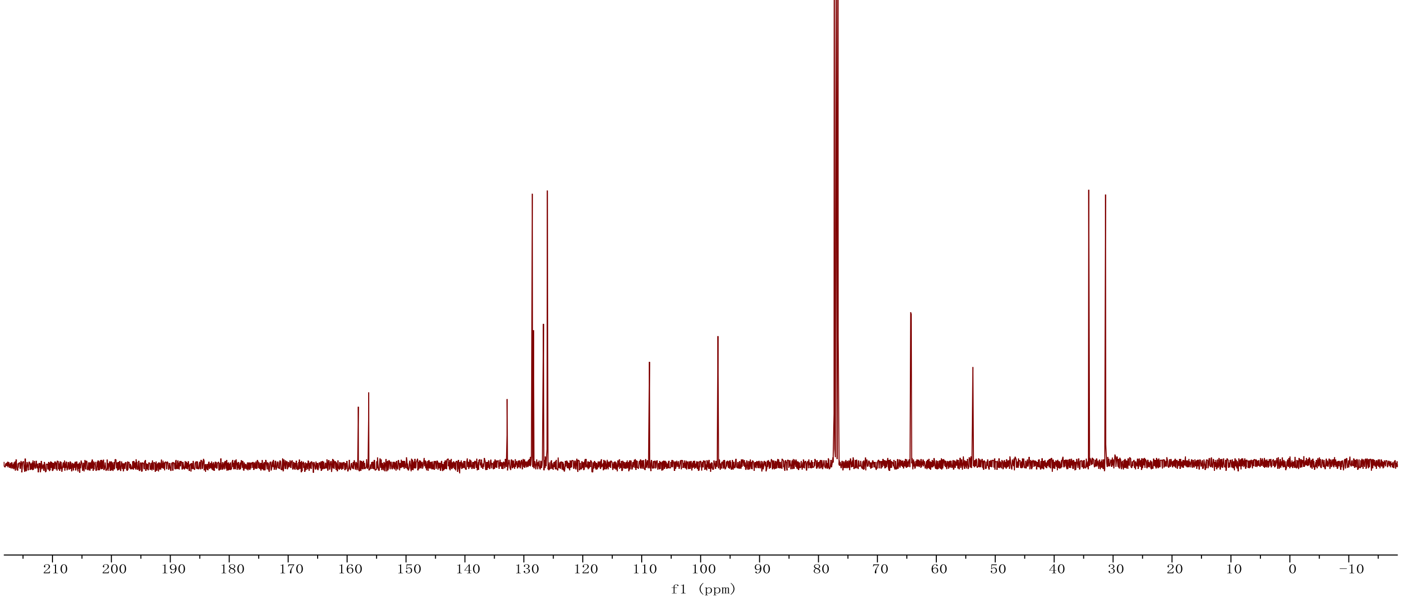

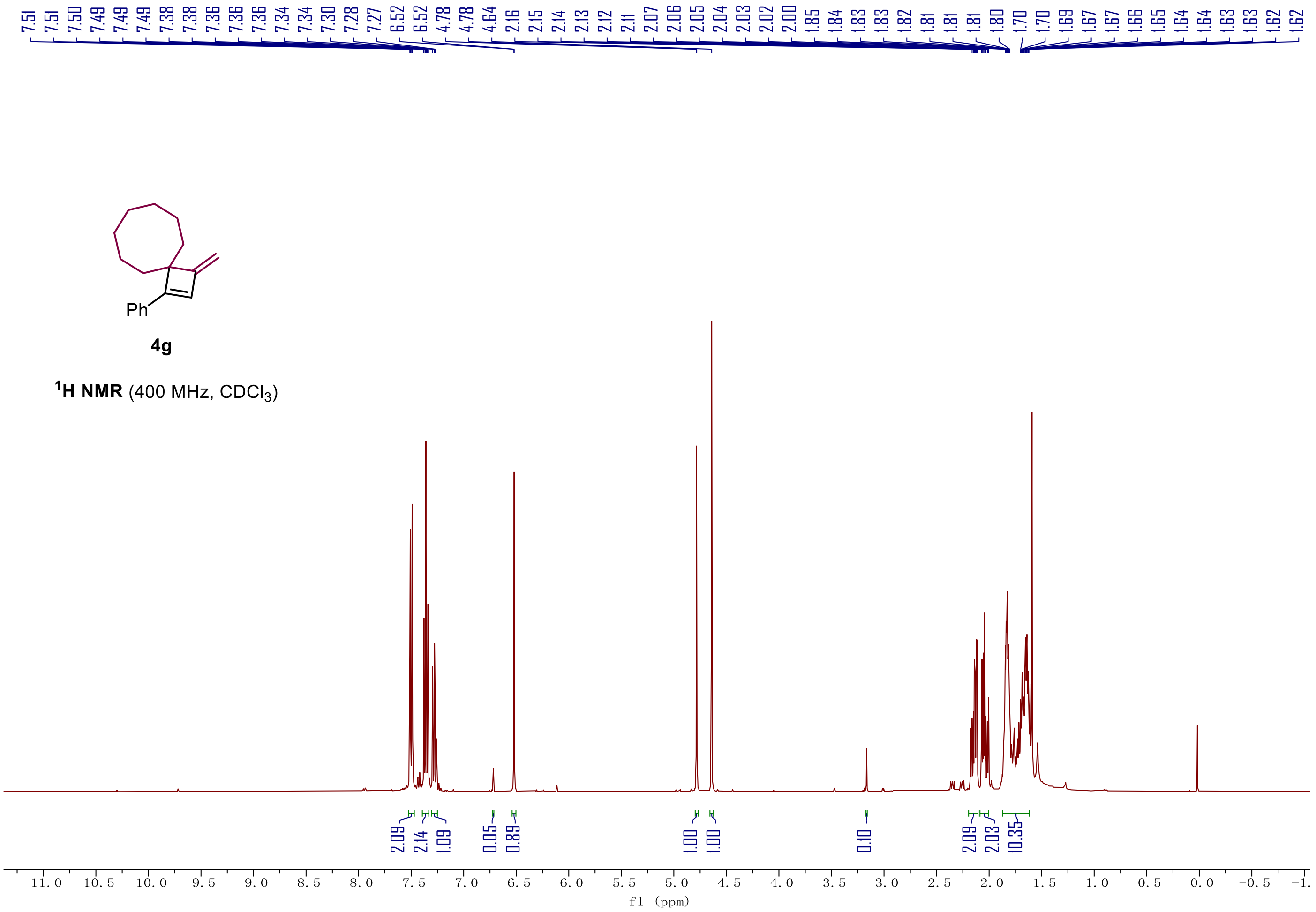
7.51 7.51 7.50 7.49 7.49 7.49 7.38 7.38 7.36 7.36 7.36 7.34 7.34 7.30 7.28 7.27 6.52 6.52 4.78 4.78 4.64 2.16 2.15 2.14 2.13 2.12 2.11 2.07 2.06 2.05 2.04 2.03 2.02 2.00 1.85 1.84 1.83 1.83 1.82 1.81 1.81 1.81 1.80 1.70 1.70 1.69 1.67 1.67 1.66 1.65 1.64 1.64 1.63 1.63 1.62 1.62
Ph
4g
1H NMR (400 MHz, CDCl3)
2.09 2.14 1.09 0.05 0.89 1.00 1.00 0.10 2.09 2.03 10.35
f1 (ppm)

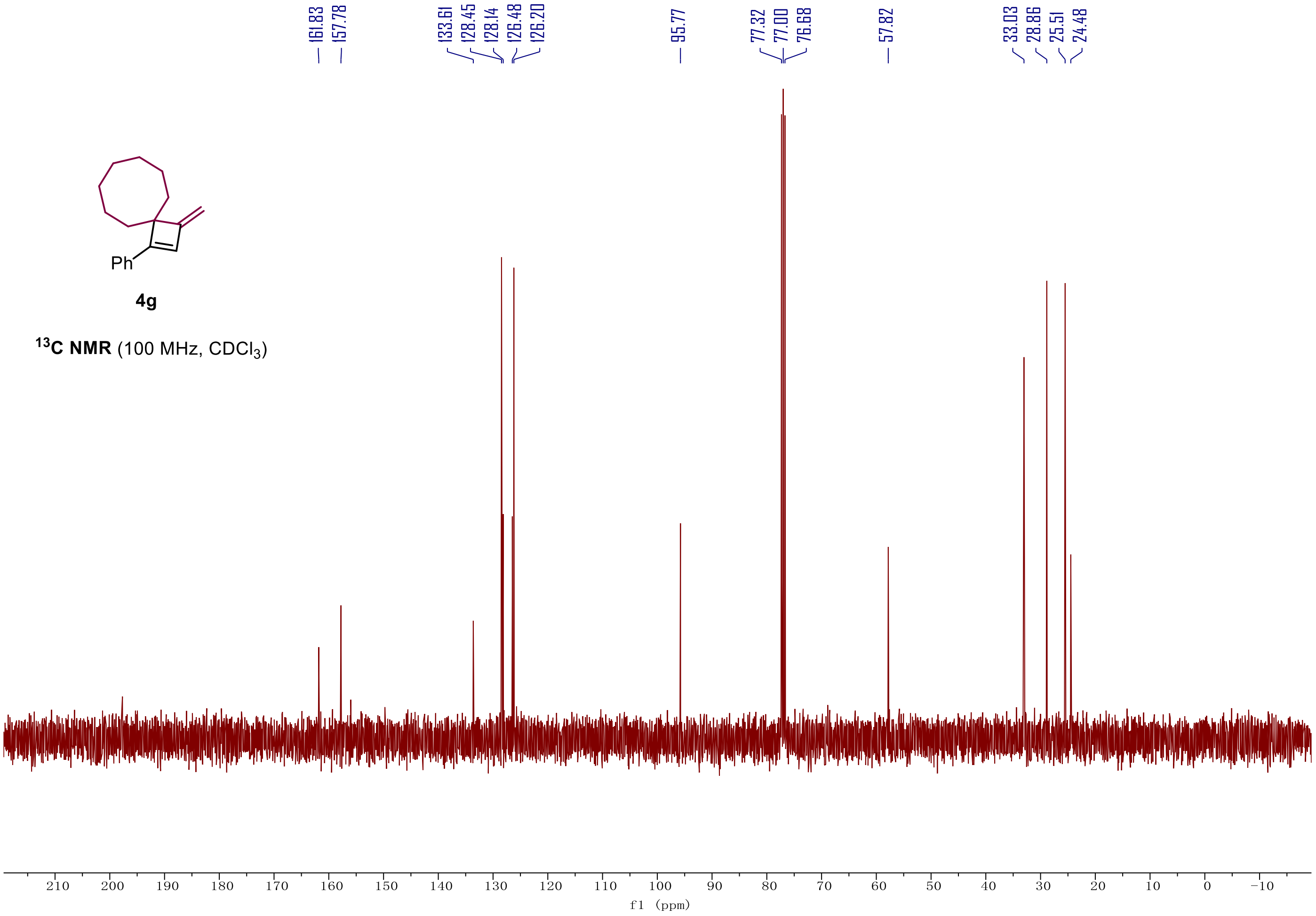
161.83 157.78 133.61 128.45 128.14 126.48 126.20 95.77 77.32 77.00 76.68 57.82 33.03 28.86 25.51 24.48
Ph
4g
13C NMR (100 MHz, CDCl3)
f1 (ppm)

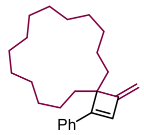

4h

^{1}H NMR (400 MHz, $CDCl_3$)

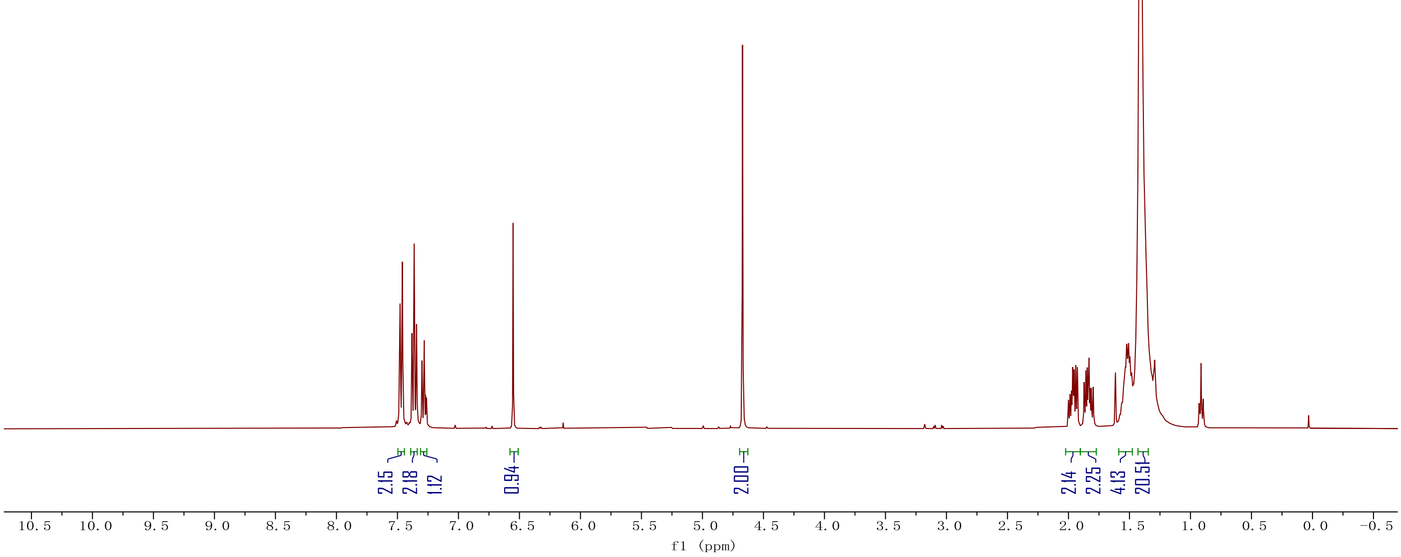

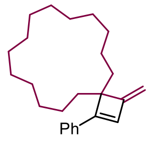

4h

^{13}C NMR (100 MHz, $CDCl_3$)

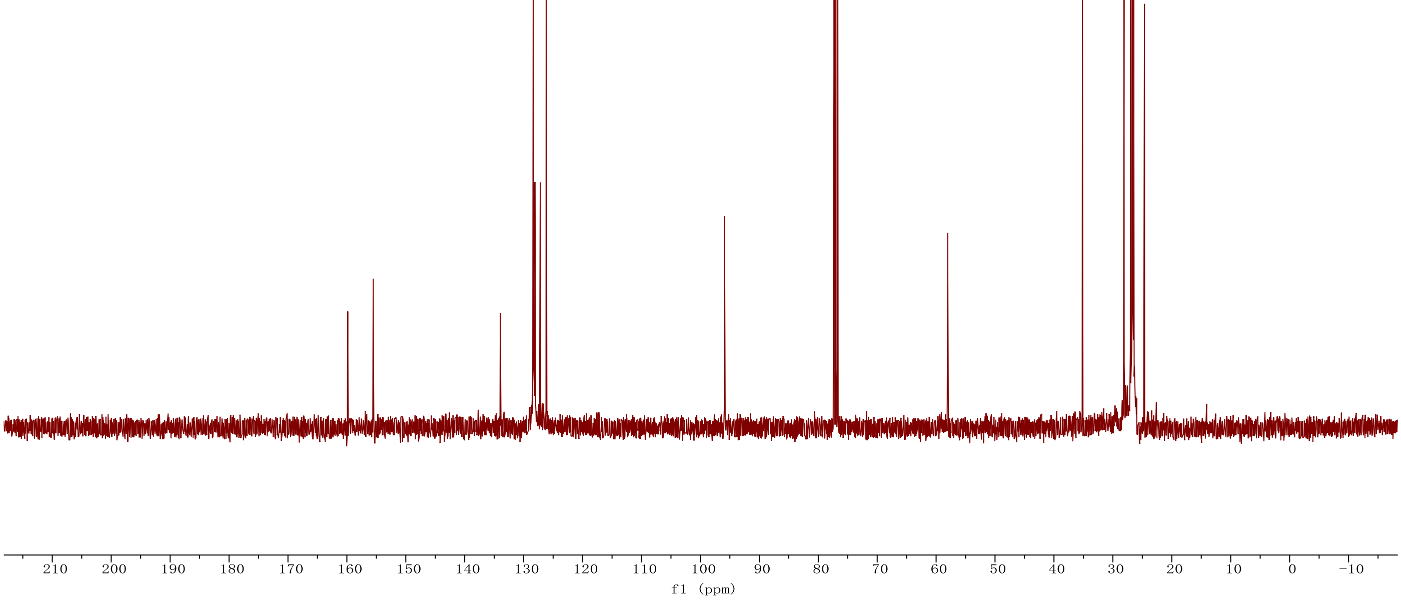

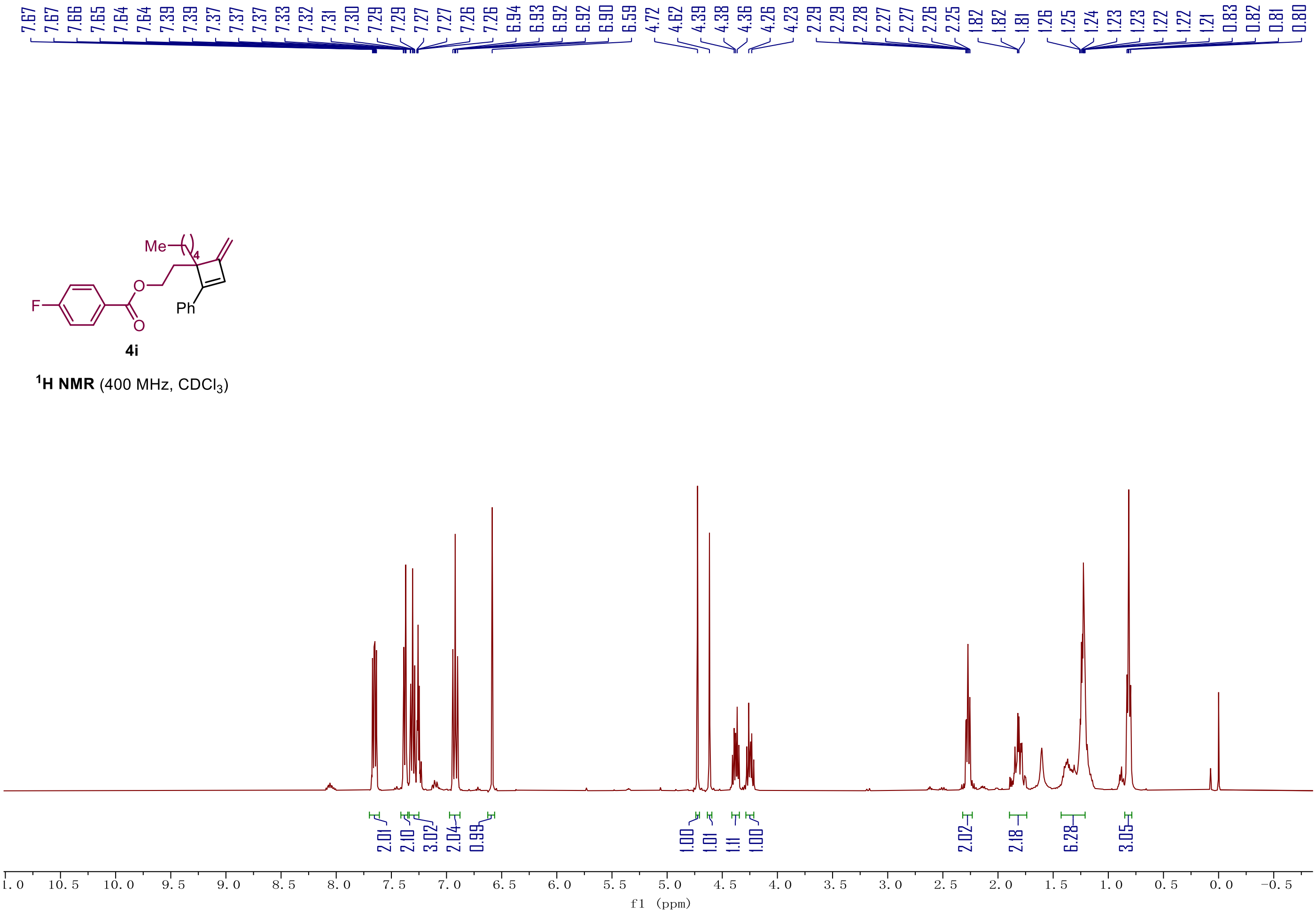

4i

^{1}H NMR (400 MHz, $CDCl_3$)

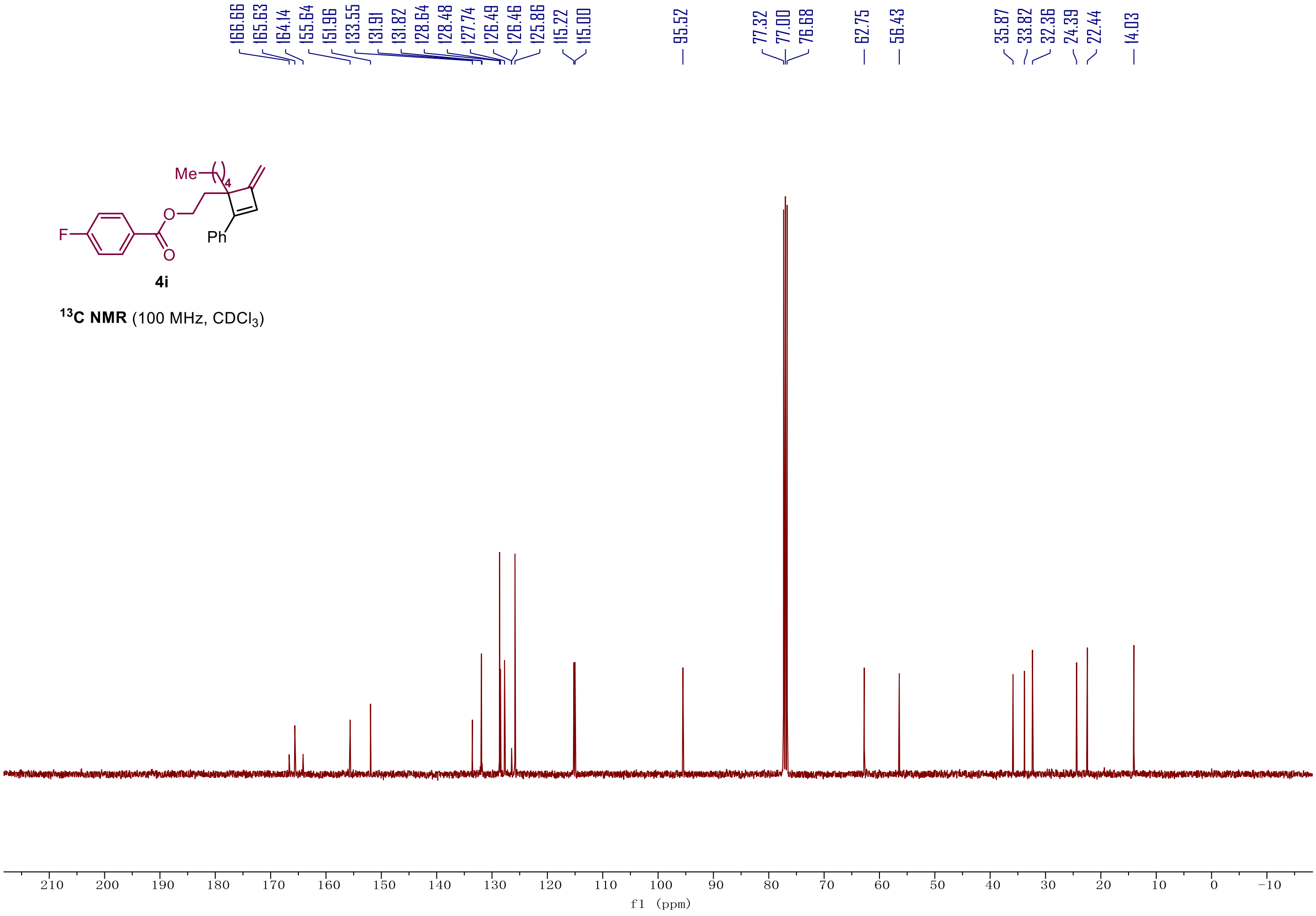

4i

^{13}C NMR (100 MHz, $CDCl_3$)

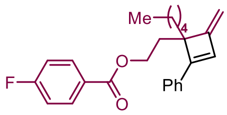

4i

^{19}F NMR (376 MHz, $CDCl_3$)

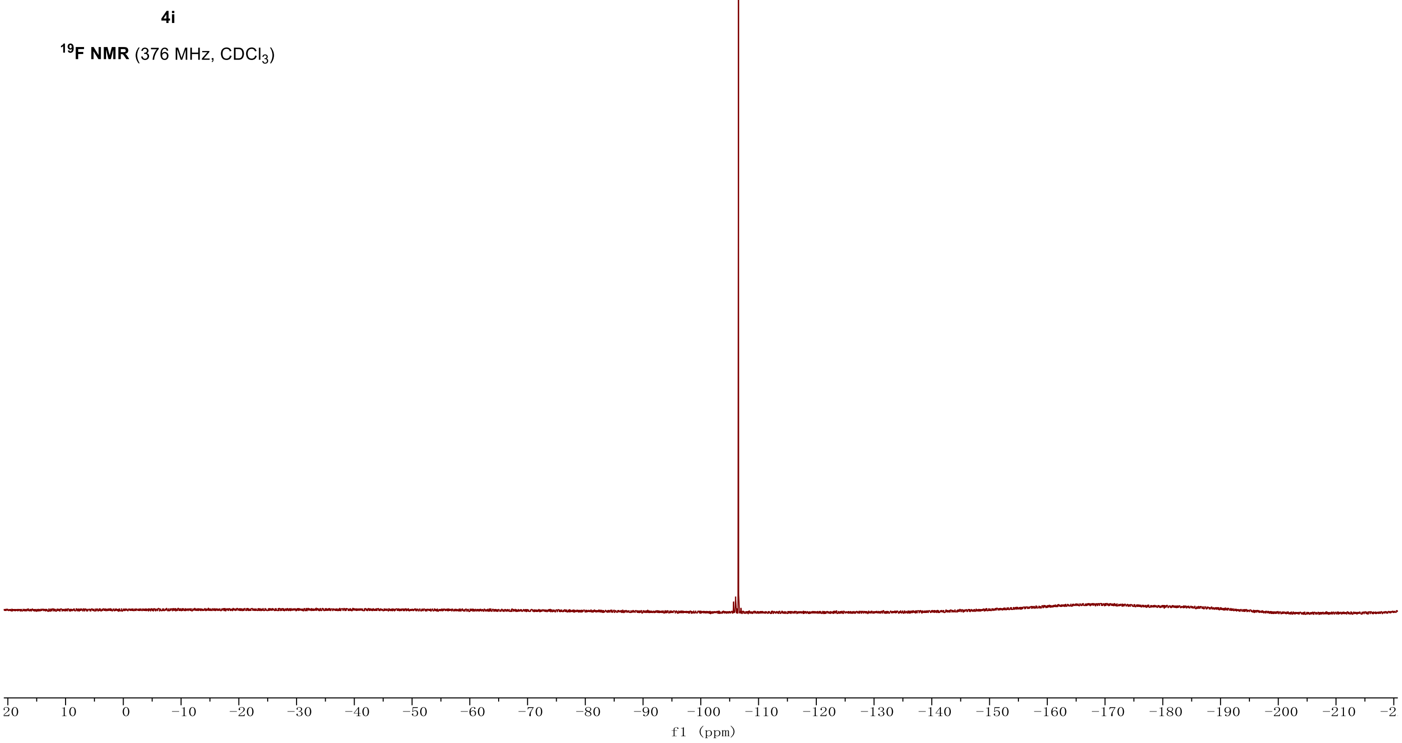

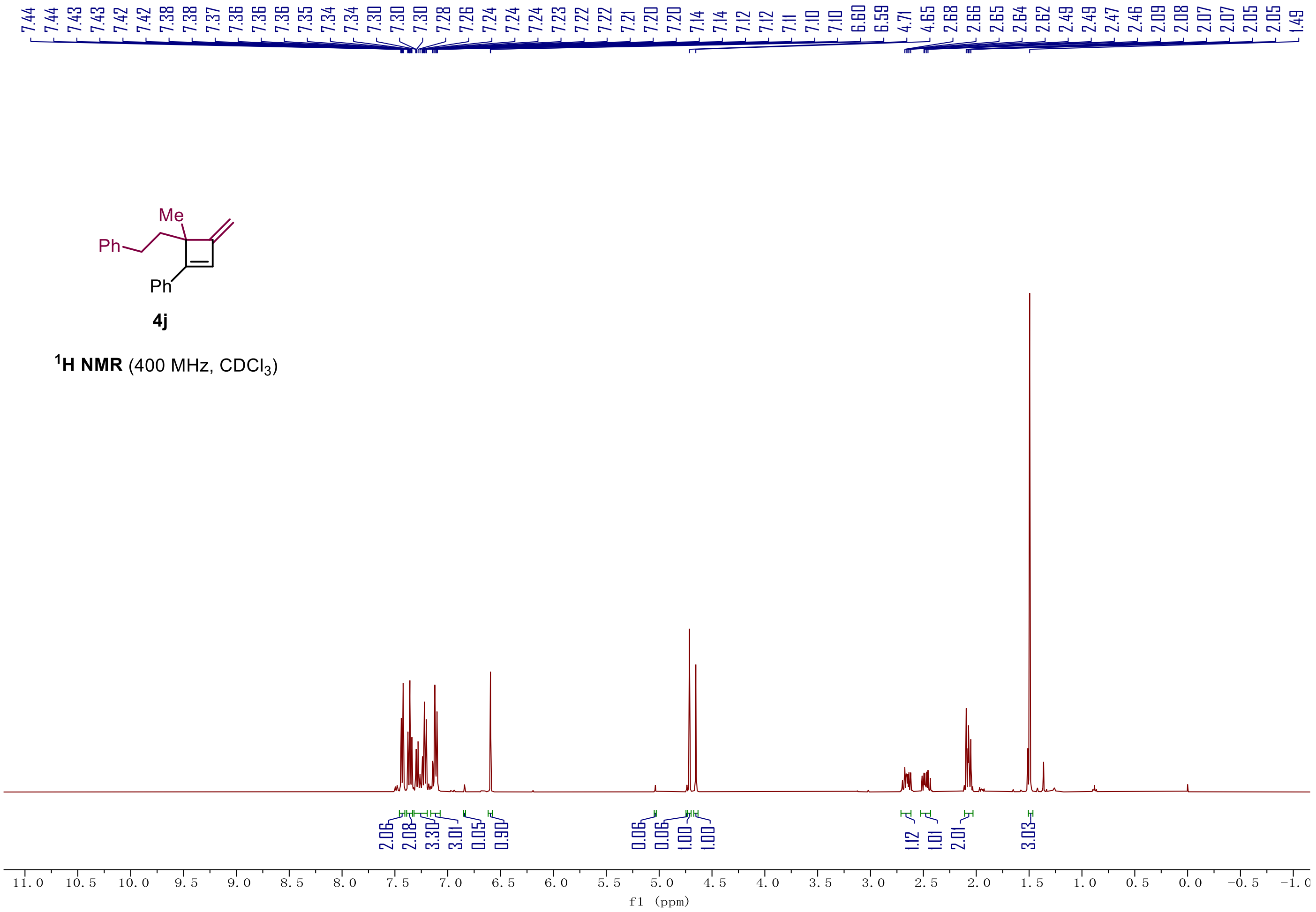
7.44 7.44 7.43 7.43 7.42 7.42 7.38 7.38 7.37 7.36 7.36 7.36 7.35 7.34 7.34 7.30 7.30 7.30 7.28 7.26 7.24 7.24 7.24 7.23 7.22 7.22 7.21 7.20 7.20 7.14 7.14 7.12 7.12 7.11 7.10 7.10 6.60 6.59 4.71 4.65 2.68 2.66 2.65 2.64 2.62 2.49 2.49 2.47 2.46 2.09 2.08 2.07 2.07 2.05 2.05 1.49
Me
Ph
Ph
4j
1H NMR (400 MHz, CDCl3)
2.06 2.08 3.30 3.01 0.05 0.90 0.06 0.06 1.00 1.00 1.12 1.01 2.01 3.03
11.0 10.5 10.0 9.5 9.0 8.5 8.0 7.5 7.0 6.5 6.0 5.5 5.0 4.5 4.0 3.5 3.0 2.5 2.0 1.5 1.0 0.5 0.0 -0.5 -1.0
f1 (ppm)

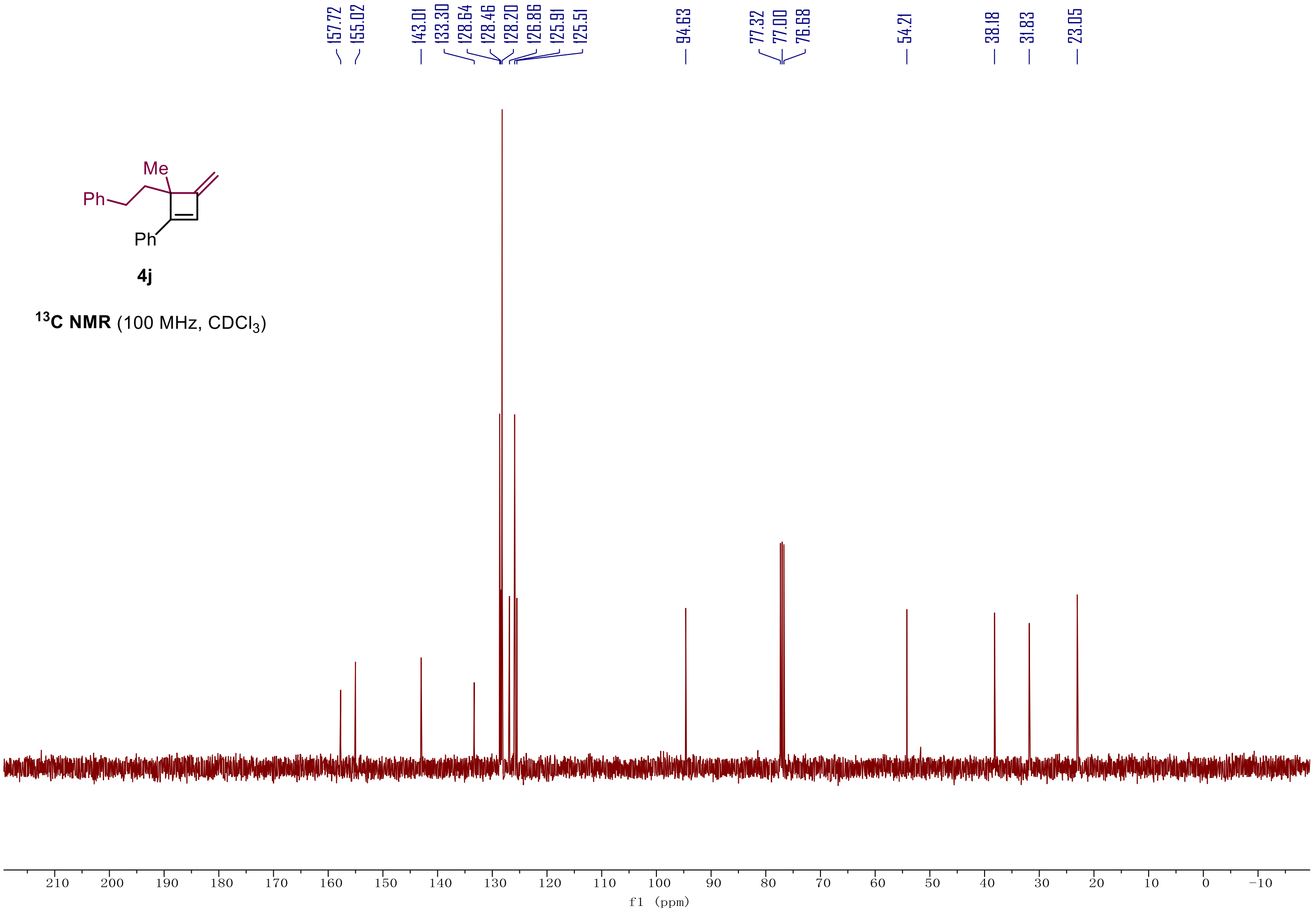
157.72 155.02 143.01 133.30 128.64 128.46 128.20 126.86 125.91 125.51 94.63 77.32 77.00 76.68 54.21 38.18 31.83 23.05
Me
Ph
Ph
4j
13C NMR (100 MHz, CDCl3)
210 200 190 180 170 160 150 140 130 120 110 100 90 80 70 60 50 40 30 20 10 0 -10
f1 (ppm)

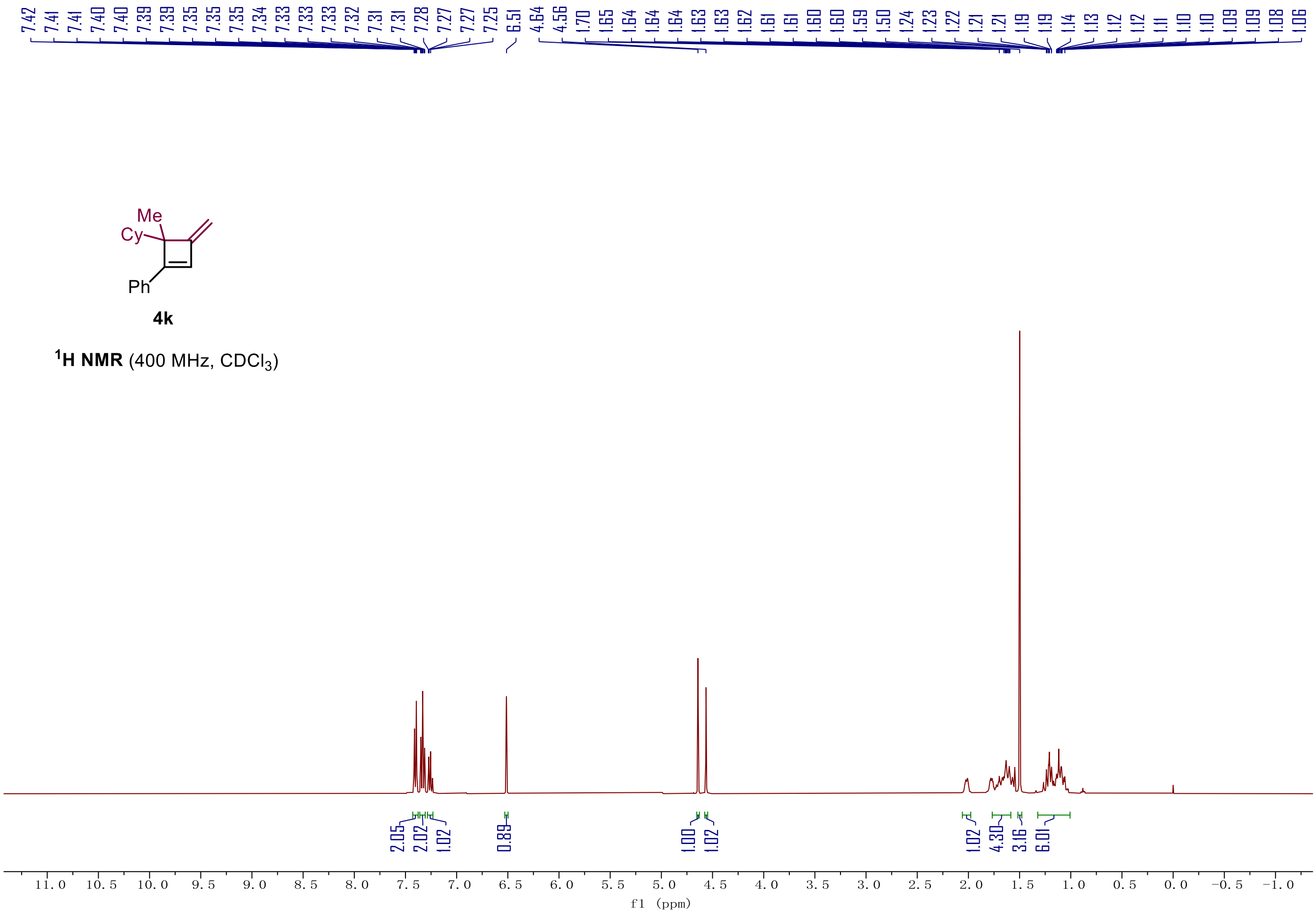

4k

^{1}H NMR (400 MHz, CDCl$_3$)

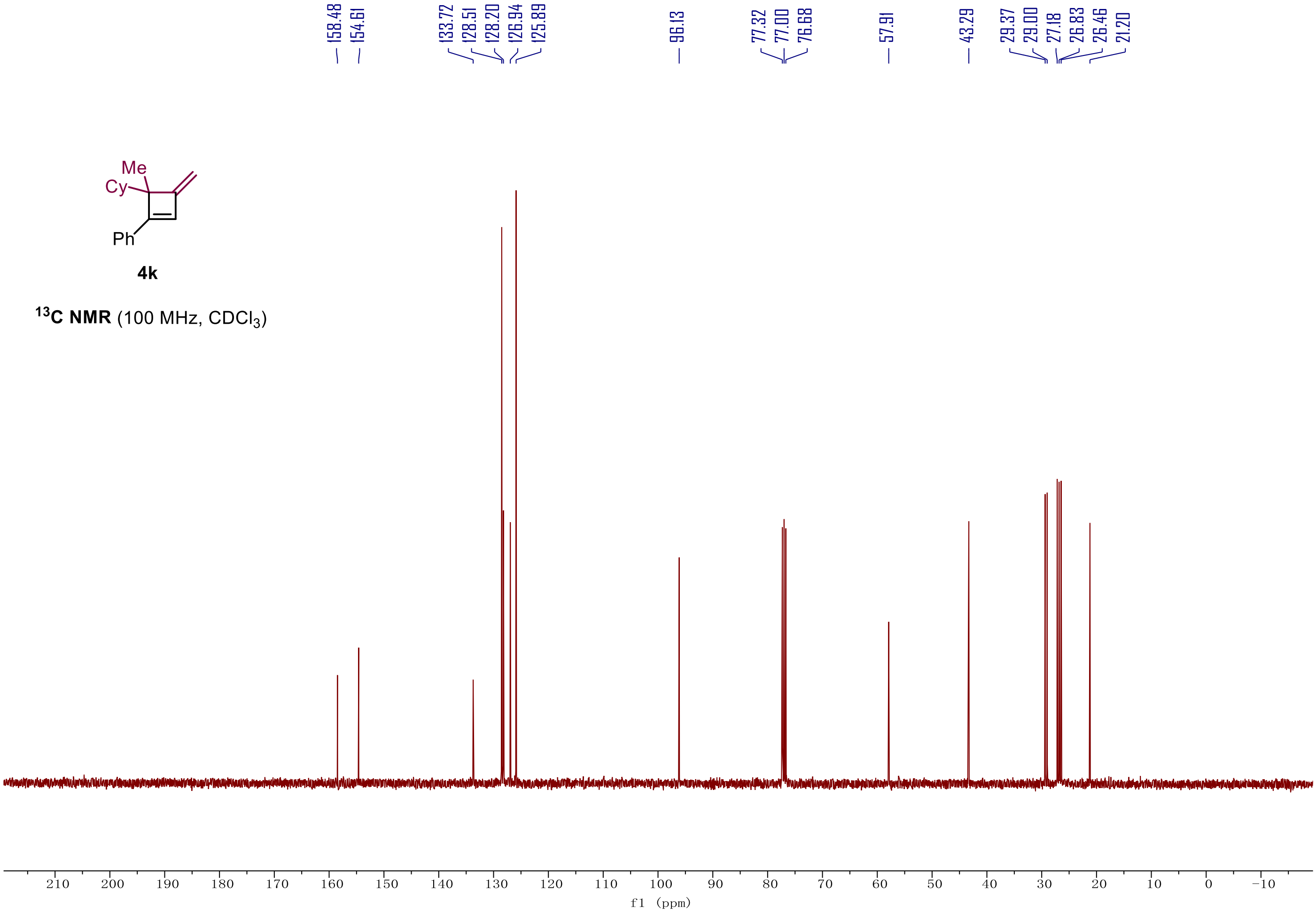

4k

^{13}C NMR (100 MHz, CDCl$_3$)

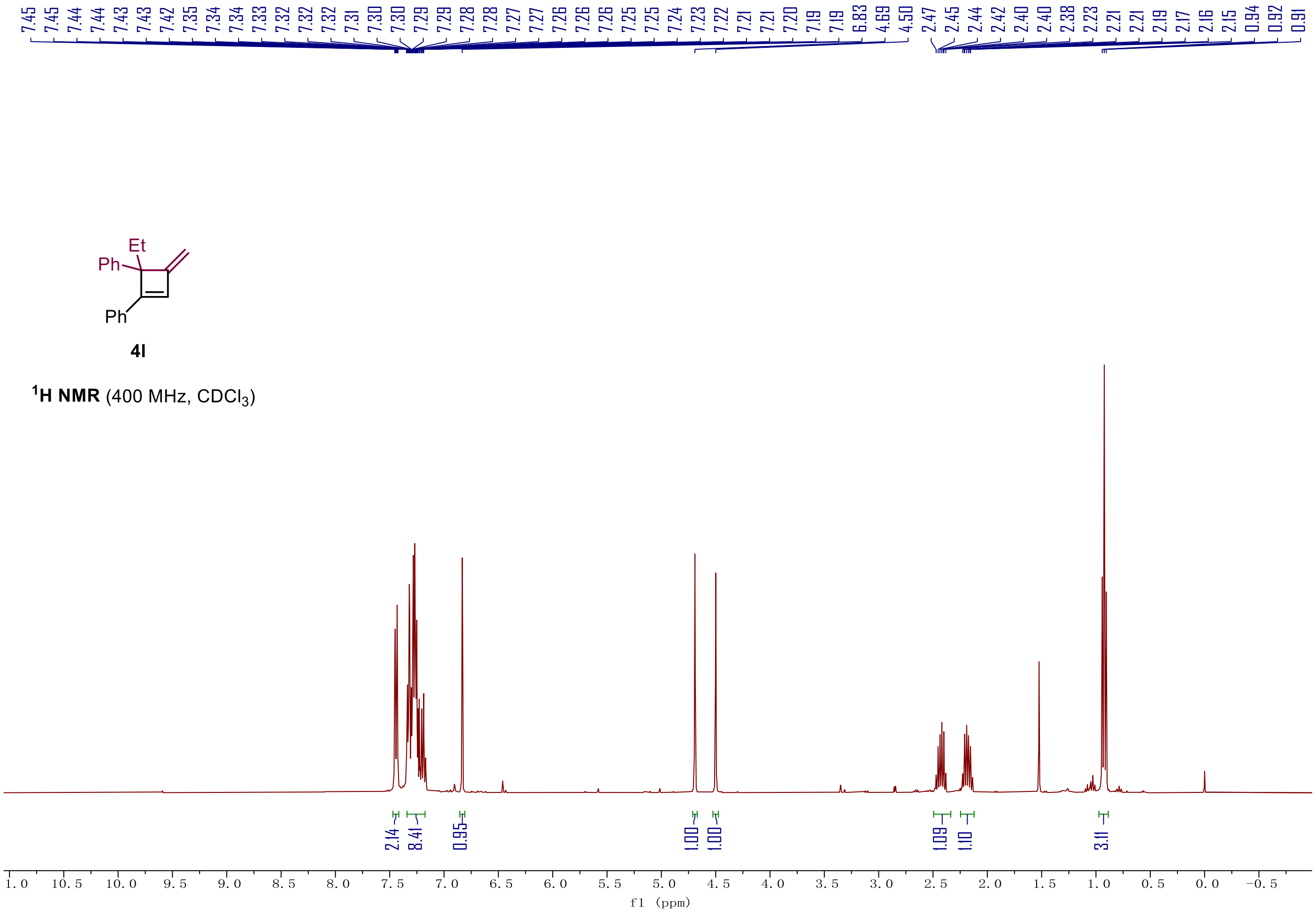
7.45 7.45 7.44 7.44 7.43 7.43 7.42 7.35 7.34 7.34 7.33 7.32 7.32 7.32 7.31 7.30 7.30 7.29 7.29 7.28 7.28 7.27 7.27 7.26 7.26 7.26 7.25 7.25 7.24 7.23 7.22 7.21 7.21 7.20 7.19 7.19 6.83 4.69 4.50 2.47 2.45 2.44 2.42 2.40 2.40 2.38 2.23 2.21 2.21 2.19 2.17 2.16 2.15 0.94 0.92 0.91
Et
Ph
Ph
4l
^{1}H NMR (400 MHz, CDCl$_3$)
2.14 8.41 0.95 1.00 1.00 1.09 1.10 3.11
f1 (ppm)

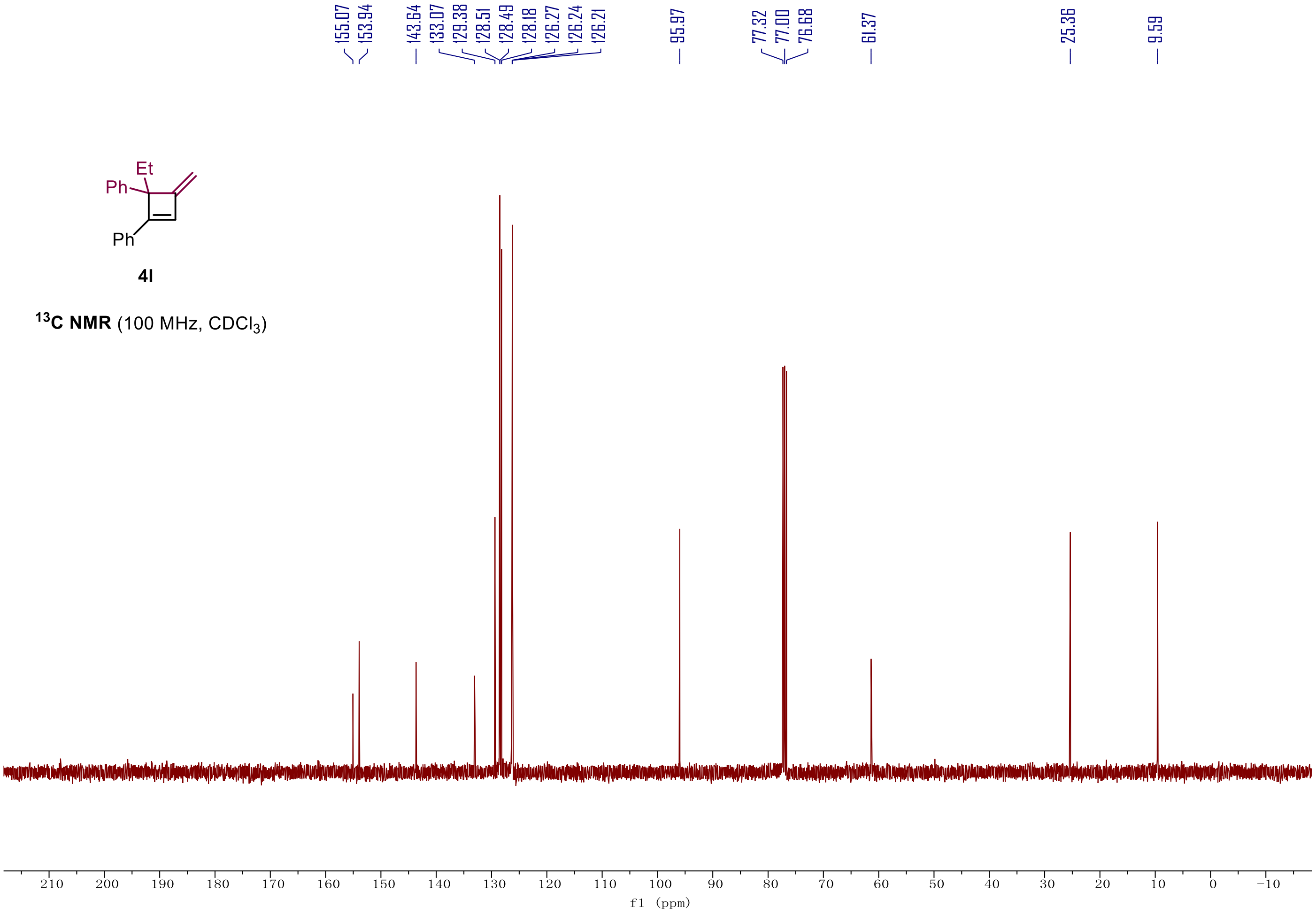
155.07 153.94 143.64 133.07 129.38 128.51 128.49 128.18 126.27 126.24 126.21 95.97 77.32 77.00 76.68 61.37 25.36 9.59
Et
Ph
Ph
4l
^{13}C NMR (100 MHz, CDCl$_3$)
f1 (ppm)

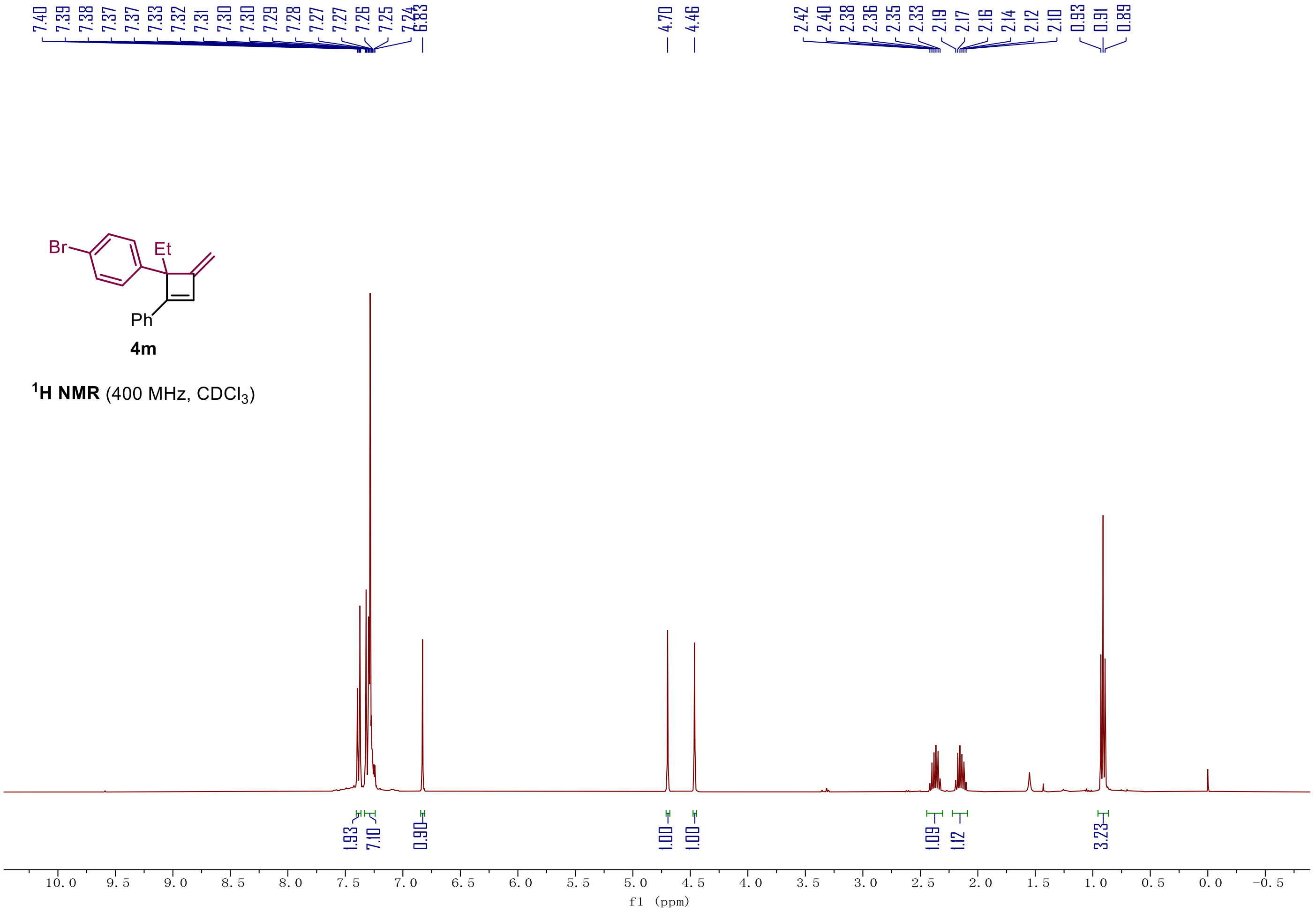
7.40 7.39 7.38 7.37 7.37 7.33 7.32 7.31 7.30 7.30 7.29 7.28 7.27 7.27 7.26 7.25 7.24 6.83
4.70 4.46
2.42 2.40 2.38 2.36 2.35 2.33 2.19 2.17 2.16 2.14 2.12 2.10 0.93 0.91 0.89
Br
Et
Ph
4m
1H NMR (400 MHz, CDCl3)
1.93 7.10 0.90 1.00 1.00 1.09 1.12 3.23
10.0 9.5 9.0 8.5 8.0 7.5 7.0 6.5 6.0 5.5 5.0 4.5 4.0 3.5 3.0 2.5 2.0 1.5 1.0 0.5 0.0 -0.5
f1 (ppm)

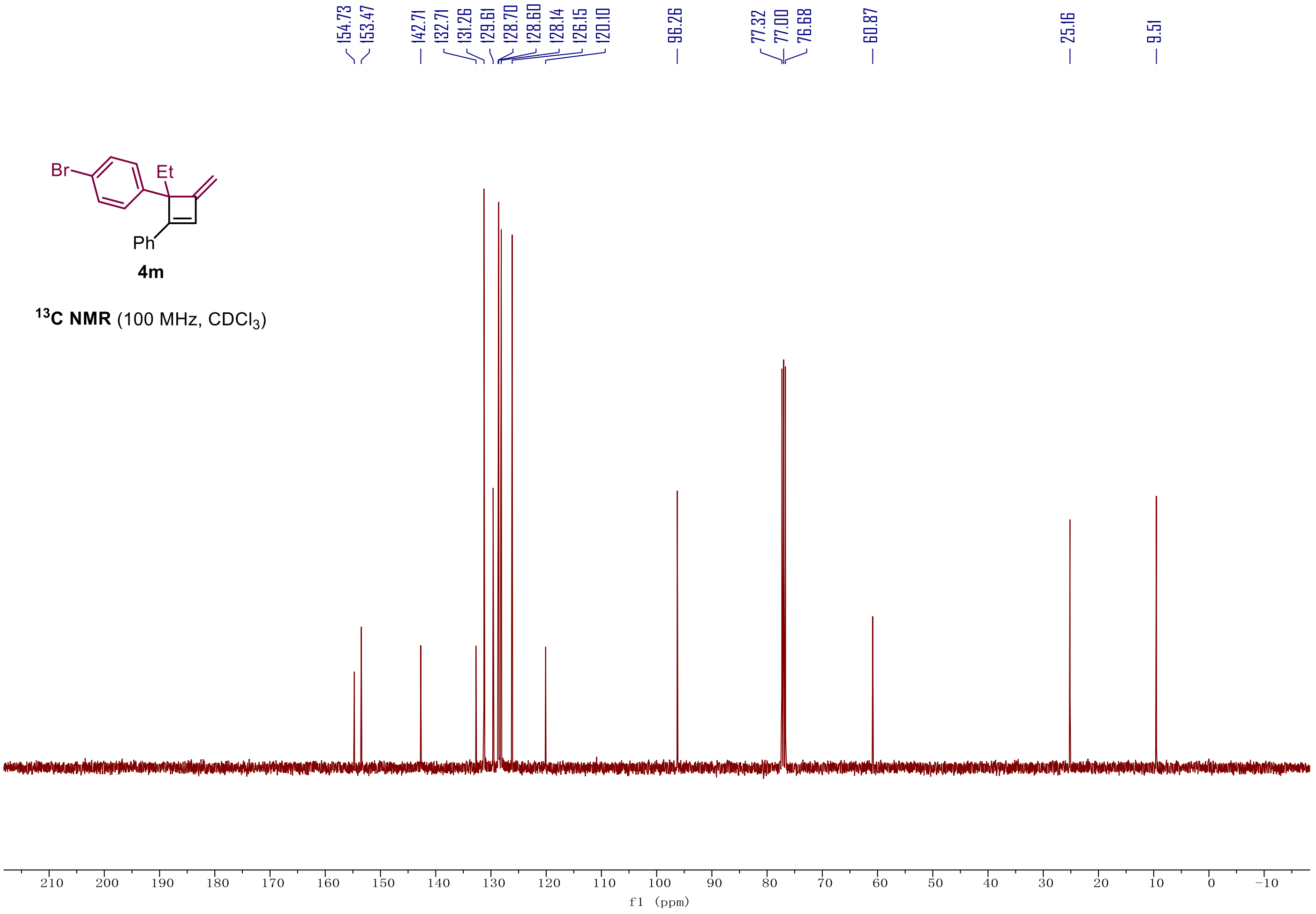
154.73 153.47 142.71 132.71 131.26 129.61 128.70 128.60 128.14 126.15 120.10 96.26 77.32 77.00 76.68 60.87 25.16 9.51
Br
Et
Ph
4m
13C NMR (100 MHz, CDCl3)
210 200 190 180 170 160 150 140 130 120 110 100 90 80 70 60 50 40 30 20 10 0 -10
f1 (ppm)

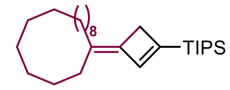

5a

^{1}H NMR (400 MHz, $CDCl_3$)

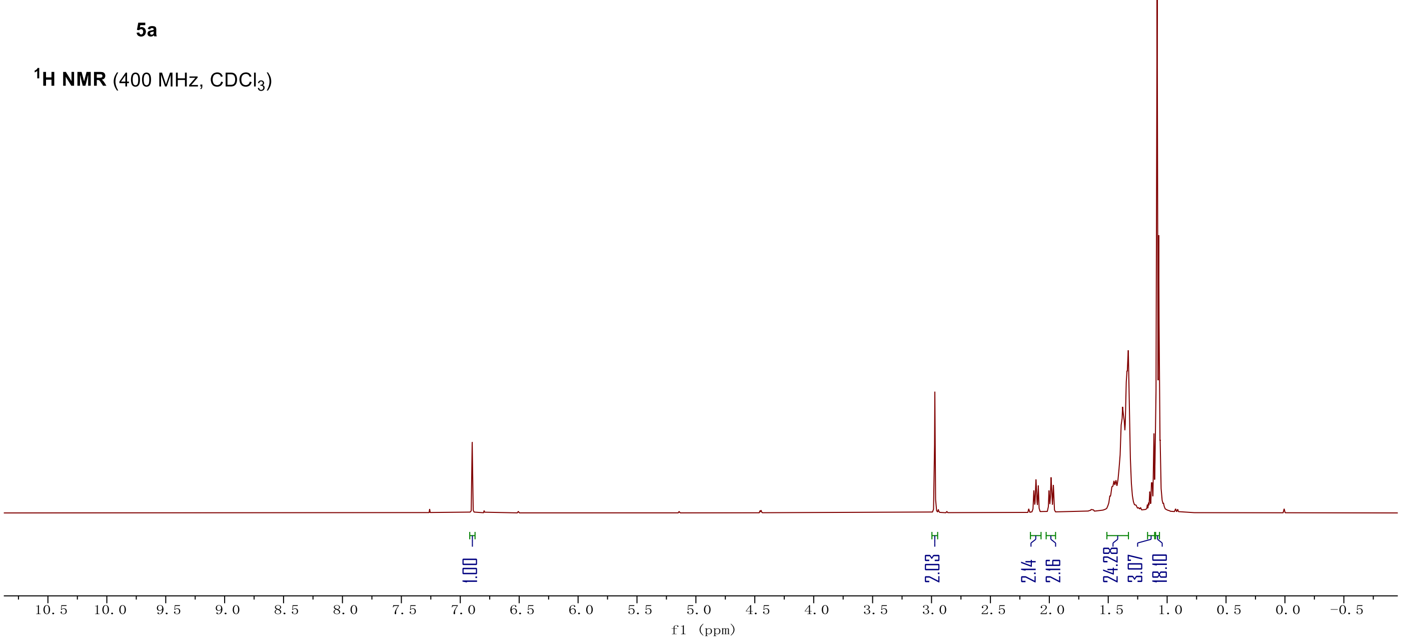

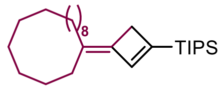

5a

^{13}C NMR (100 MHz, $CDCl_3$)

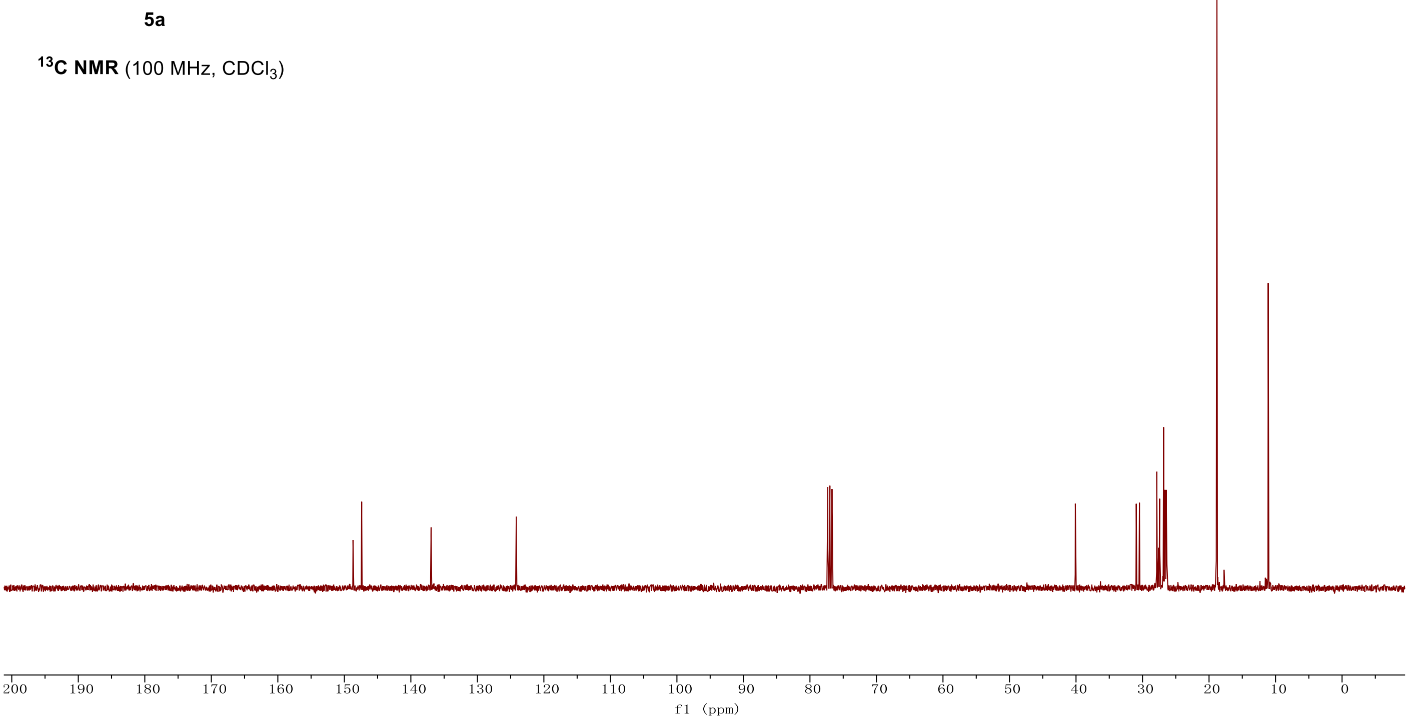

5b

^{1}H NMR (400 MHz, $CDCl_3$)

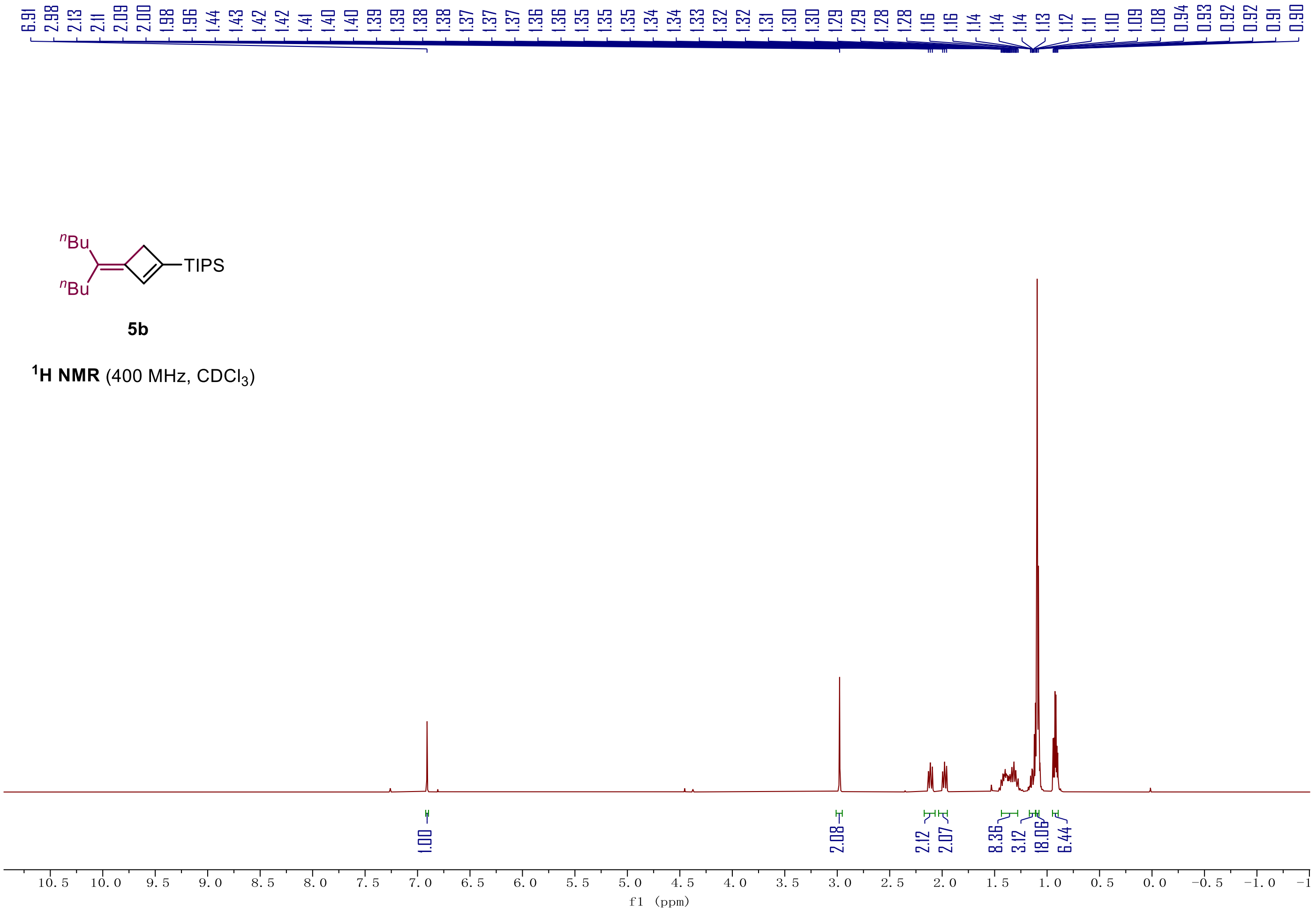

5b

^{13}C NMR (100 MHz, $CDCl_3$)

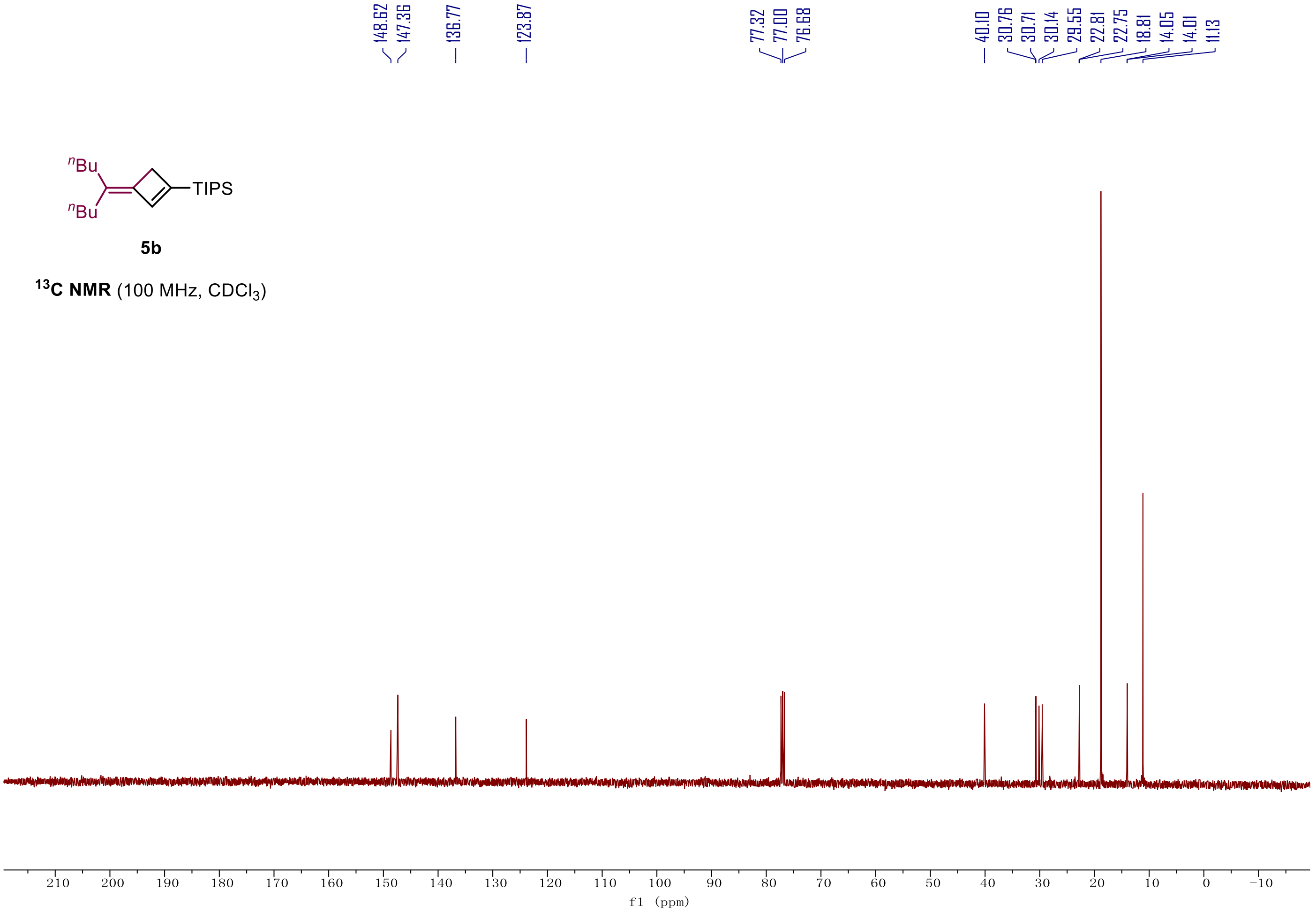

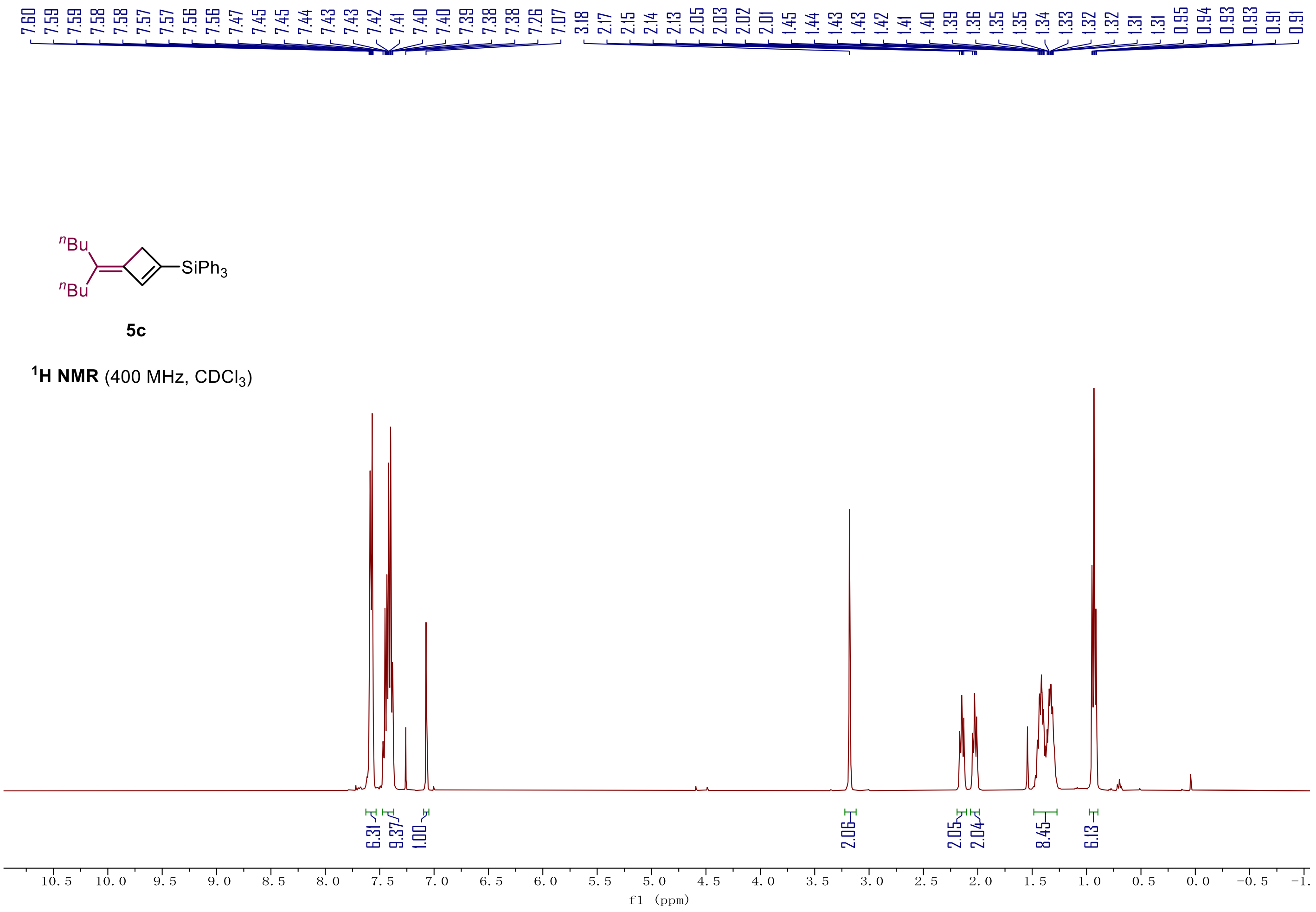
7.60 7.59 7.59 7.58 7.58 7.57 7.57 7.56 7.56 7.47 7.45 7.45 7.44 7.43 7.43 7.42 7.41 7.40 7.40 7.39 7.38 7.38 7.26 7.07 3.18 2.17 2.15 2.14 2.13 2.05 2.03 2.02 2.01 1.45 1.44 1.43 1.43 1.42 1.41 1.40 1.39 1.36 1.35 1.35 1.34 1.33 1.32 1.32 1.31 1.31 0.95 0.94 0.93 0.93 0.91 0.91
nBu
nBu
SiPh3
5c
1H NMR (400 MHz, CDCl3)
6.31
9.37
1.00
2.06
2.05
2.04
8.45
6.13
f1 (ppm)

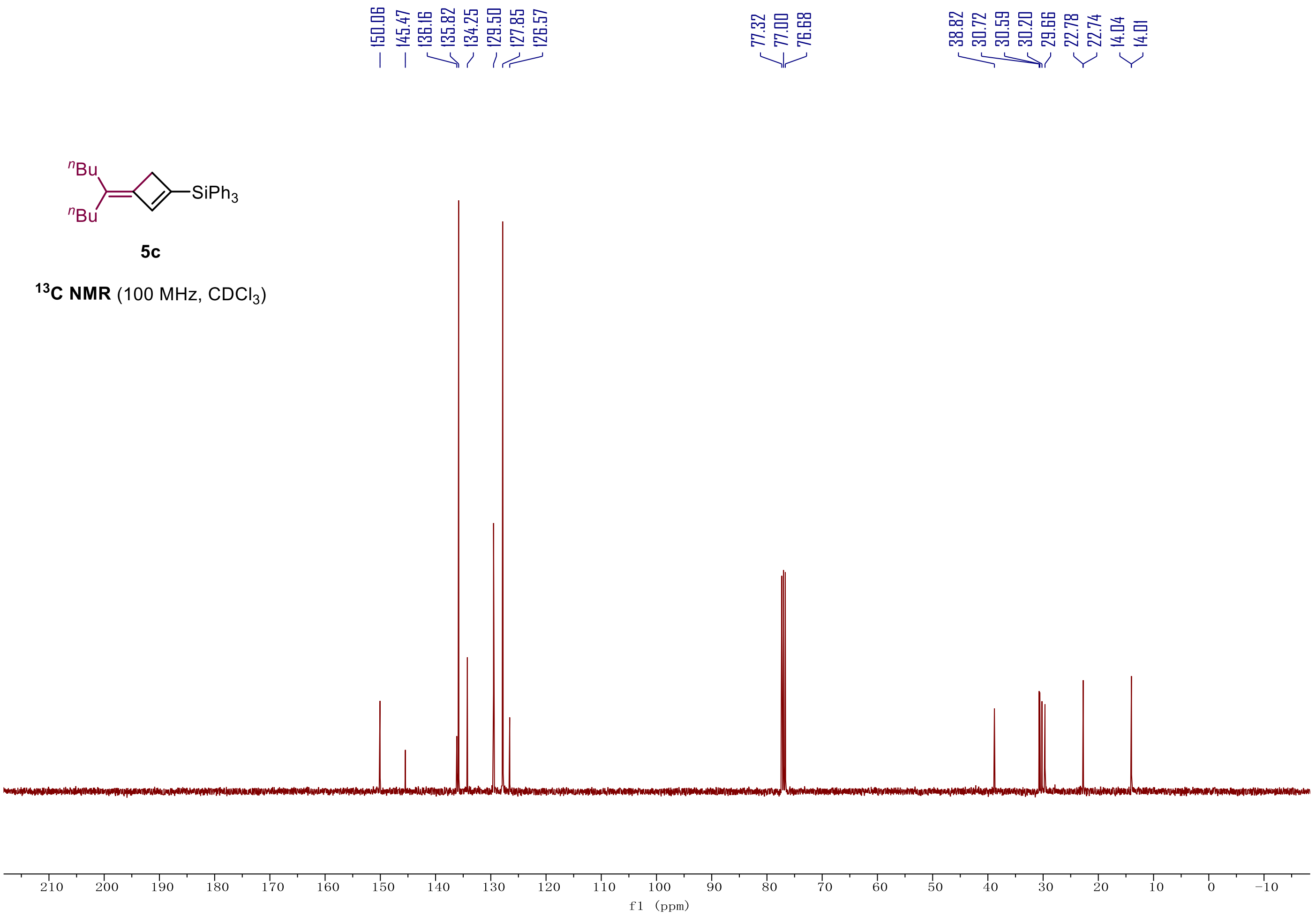
150.06 145.47 136.16 135.82 134.25 129.50 127.85 126.57
77.32 77.00 76.68
38.82 30.72 30.59 30.20 29.66 22.78 22.74 14.04 14.01
nBu
nBu
SiPh3
5c
13C NMR (100 MHz, CDCl3)
f1 (ppm)

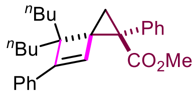

6

^{1}H NMR (400 MHz, $CDCl_3$)

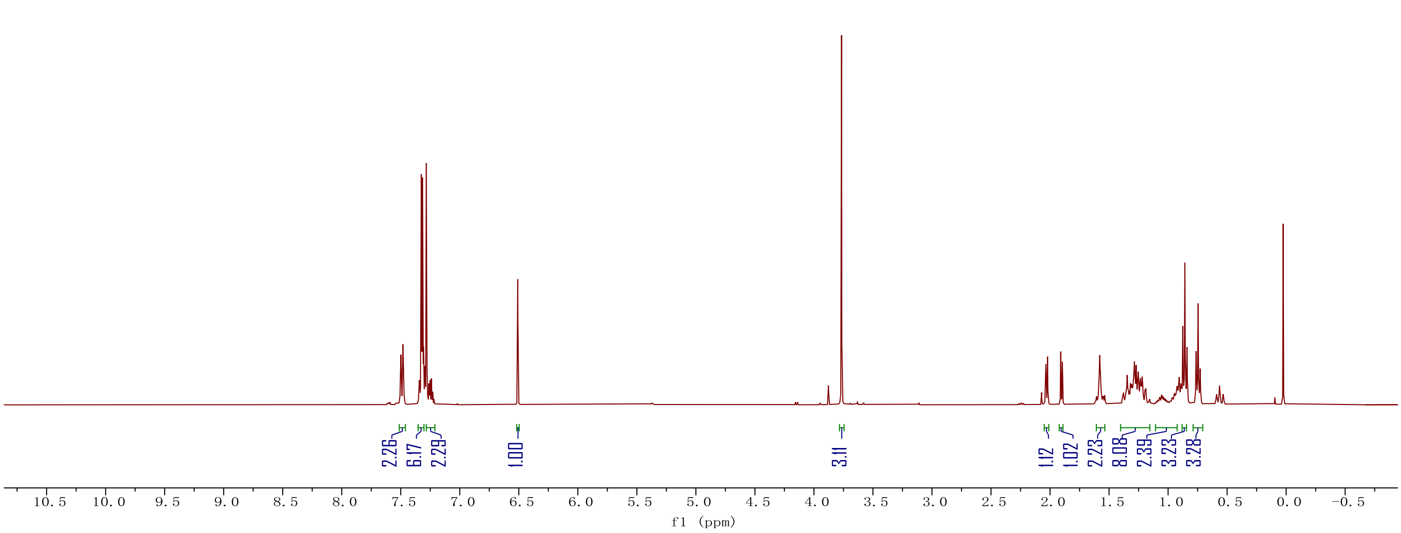

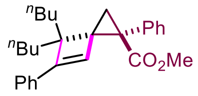

6

^{13}C NMR (100 MHz, $CDCl_3$)

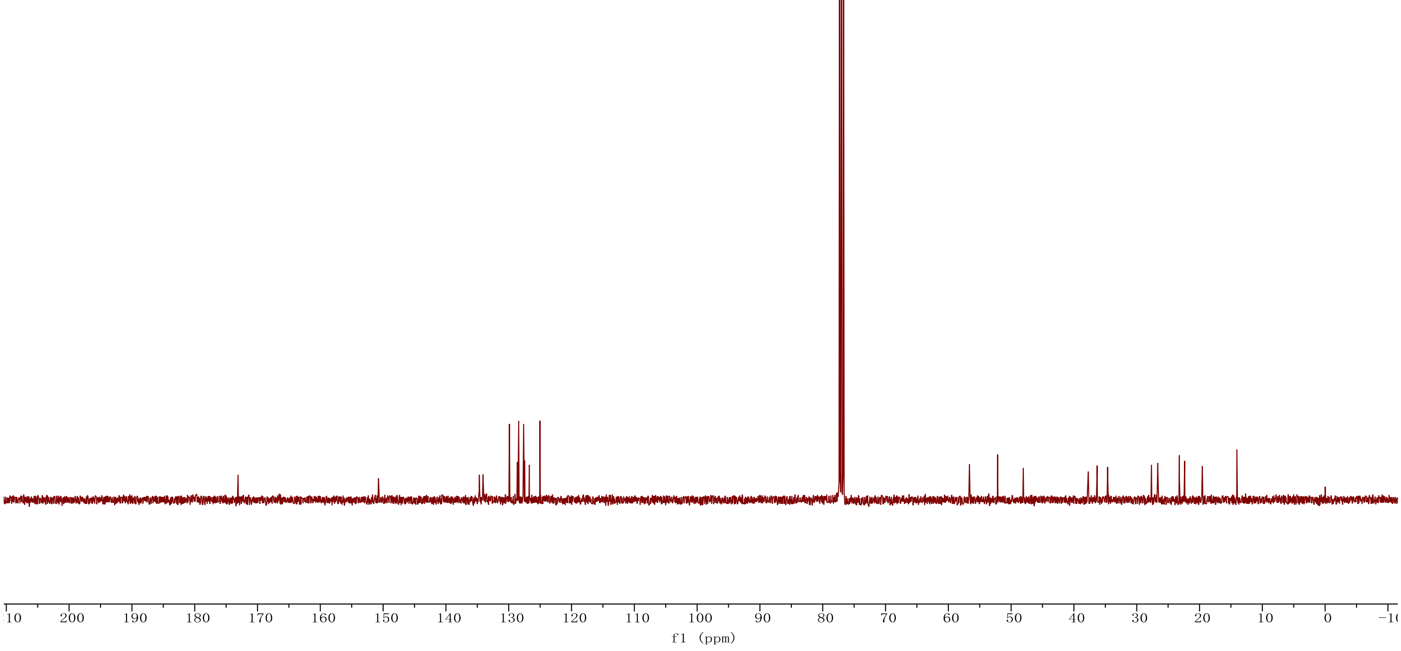

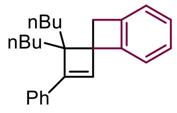

7

[1]**H NMR** (400 MHz, $CDCl_3$)

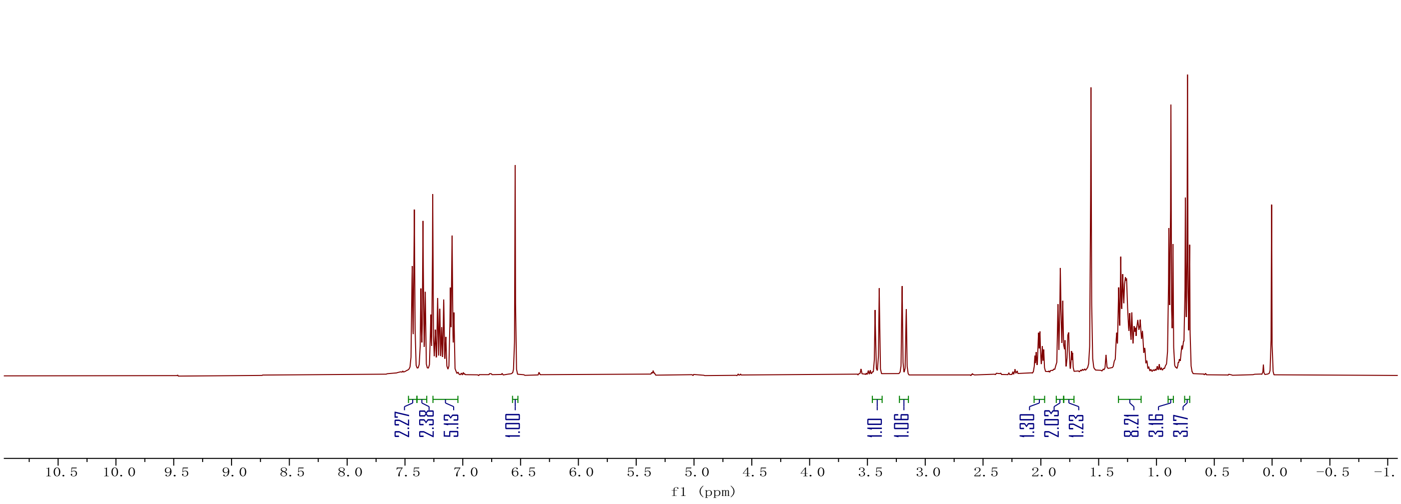

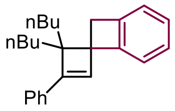

7

[13]**C NMR** (100 MHz, $CDCl_3$)

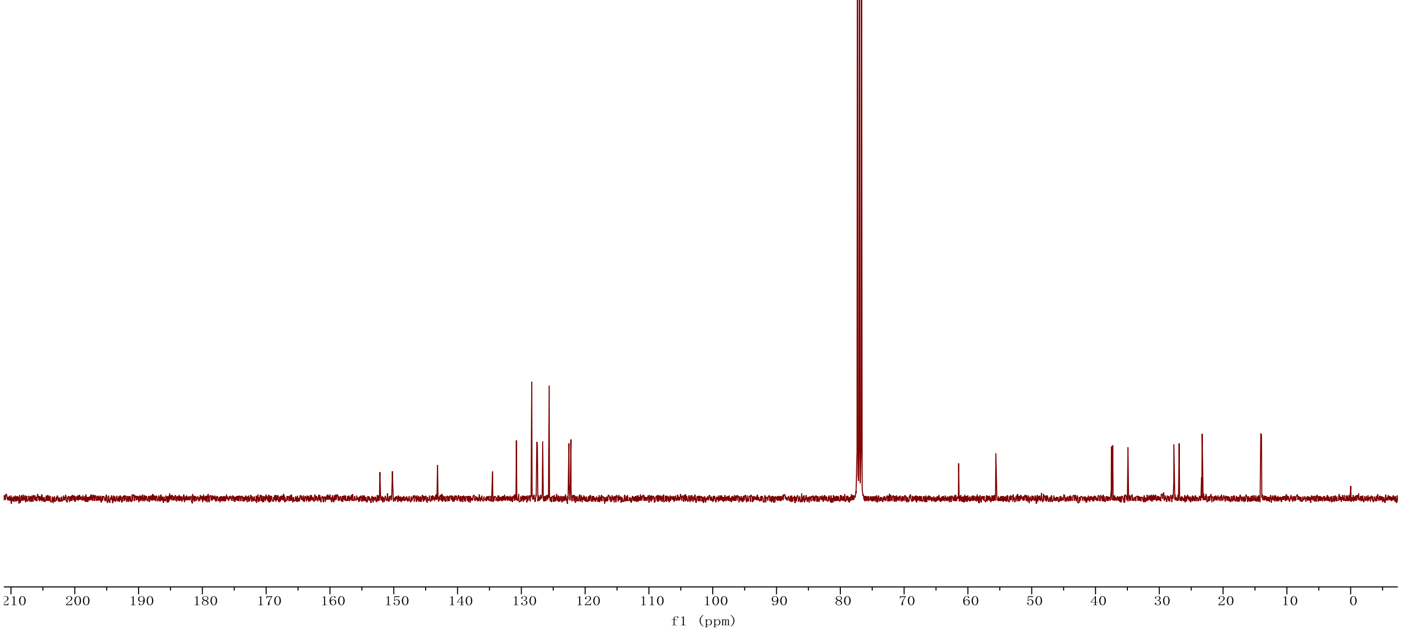

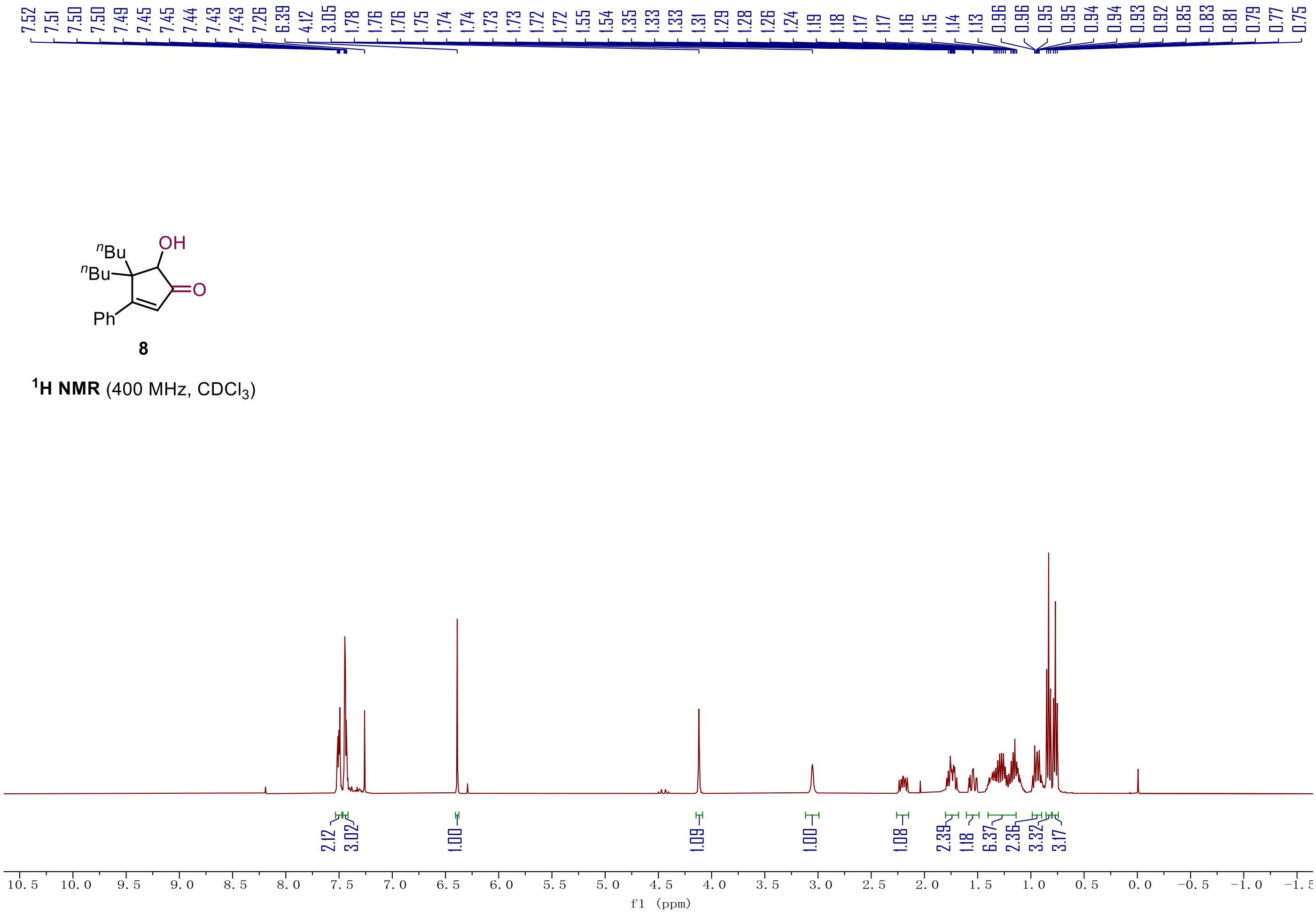
7.52 7.51 7.50 7.50 7.49 7.45 7.45 7.44 7.43 7.43 7.26 6.39 4.12 3.05 1.78 1.76 1.76 1.75 1.74 1.74 1.73 1.73 1.72 1.72 1.55 1.54 1.35 1.33 1.33 1.31 1.29 1.28 1.26 1.24 1.19 1.18 1.17 1.17 1.16 1.15 1.14 1.13 0.96 0.96 0.95 0.95 0.94 0.94 0.93 0.92 0.85 0.83 0.81 0.79 0.77 0.75
^{n}Bu
^{n}Bu
OH
O
Ph
8
^{1}H NMR (400 MHz, $CDCl_3$)
2.12 3.02 1.00 1.09 1.00 1.08 2.39 1.18 6.37 2.36 3.32 3.17
10.5 10.0 9.5 9.0 8.5 8.0 7.5 7.0 6.5 6.0 5.5 5.0 4.5 4.0 3.5 3.0 2.5 2.0 1.5 1.0 0.5 0.0 -0.5 -1.0 -1.5
f1 (ppm)

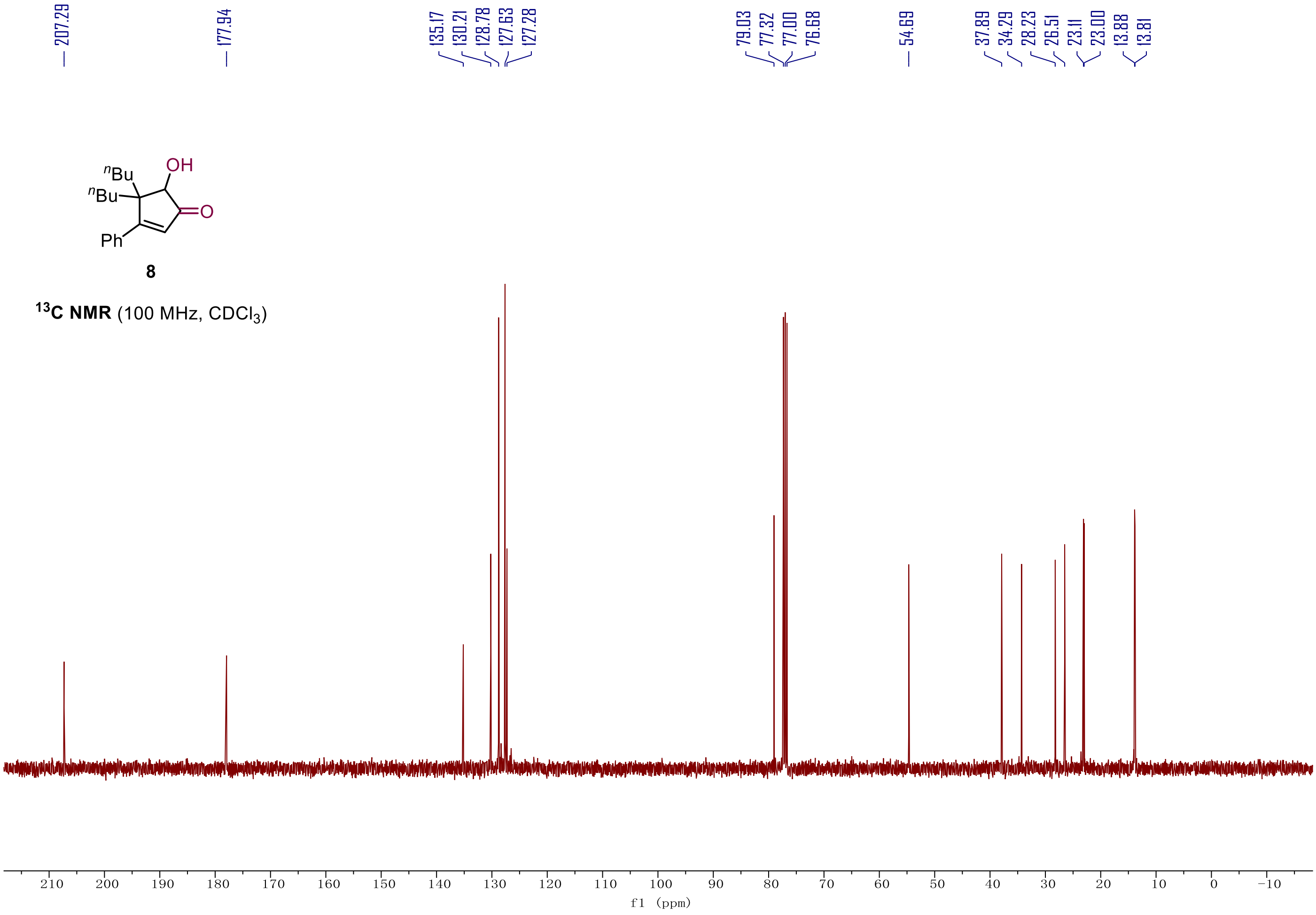
207.29
177.94
135.17 130.21 128.78 127.63 127.28
79.03 77.32 77.00 76.68
54.69
37.89 34.29 28.23 26.51 23.11 23.00 13.88 13.81
^{n}Bu
^{n}Bu
OH
O
Ph
8
^{13}C NMR (100 MHz, $CDCl_3$)
210 200 190 180 170 160 150 140 130 120 110 100 90 80 70 60 50 40 30 20 10 0 -10
f1 (ppm)

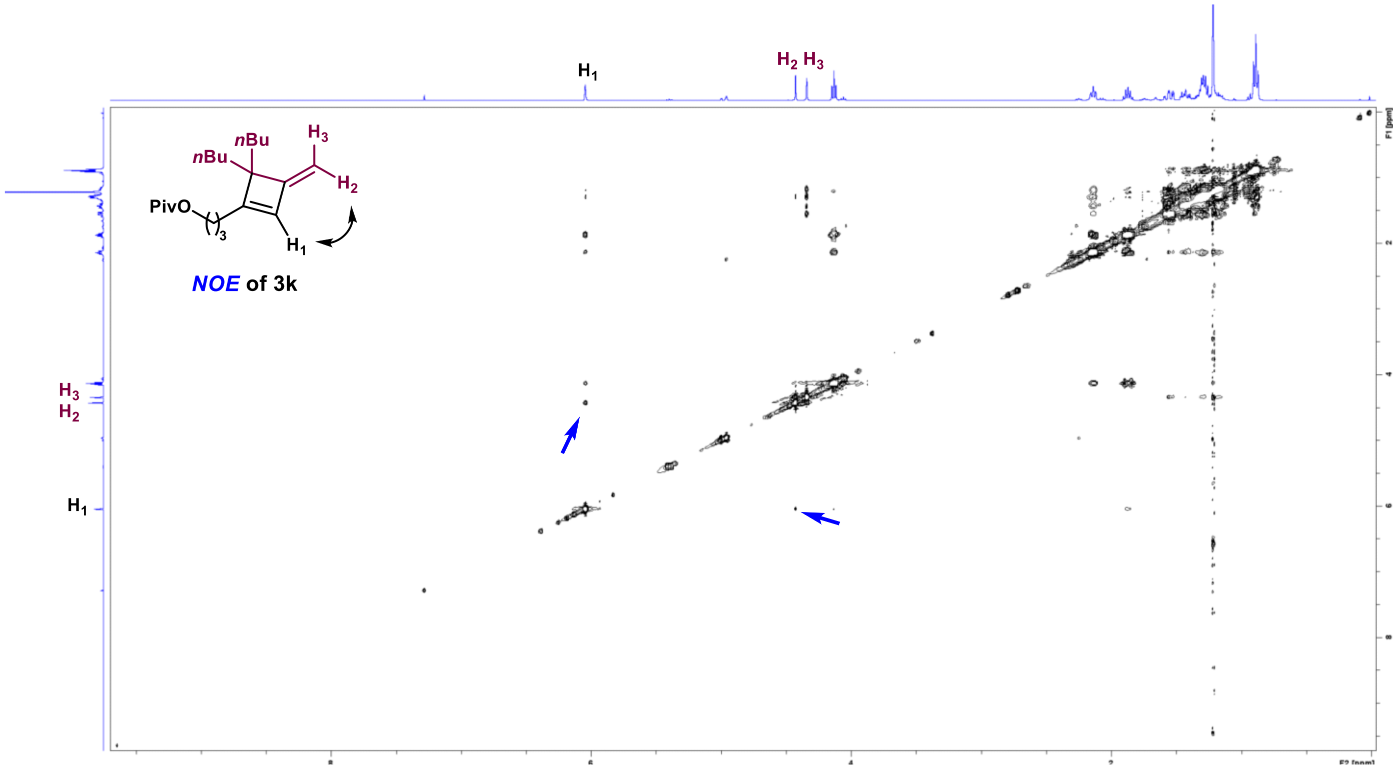
H1
H2 H3
nBu
nBu
H3
H2
PivO
3
H1
NOE of 3k
H3
H2
H1
F1 [ppm]
F2 [ppm]

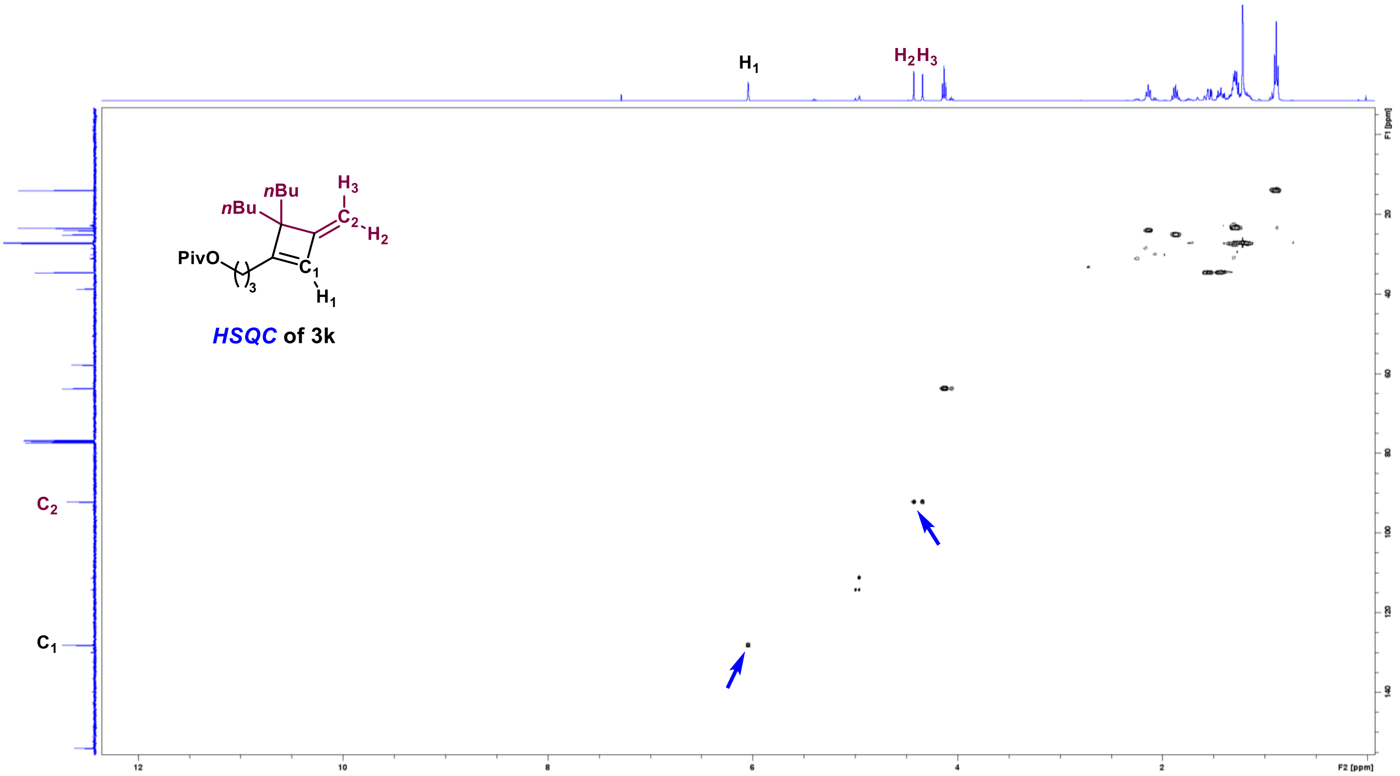
H1
H2H3
nBu
nBu
H3
C2
H2
PivO
3
C1
H1
HSQC of 3k
C2
C1
F1 [ppm]
12
10
8
6
4
2
F2 [ppm]

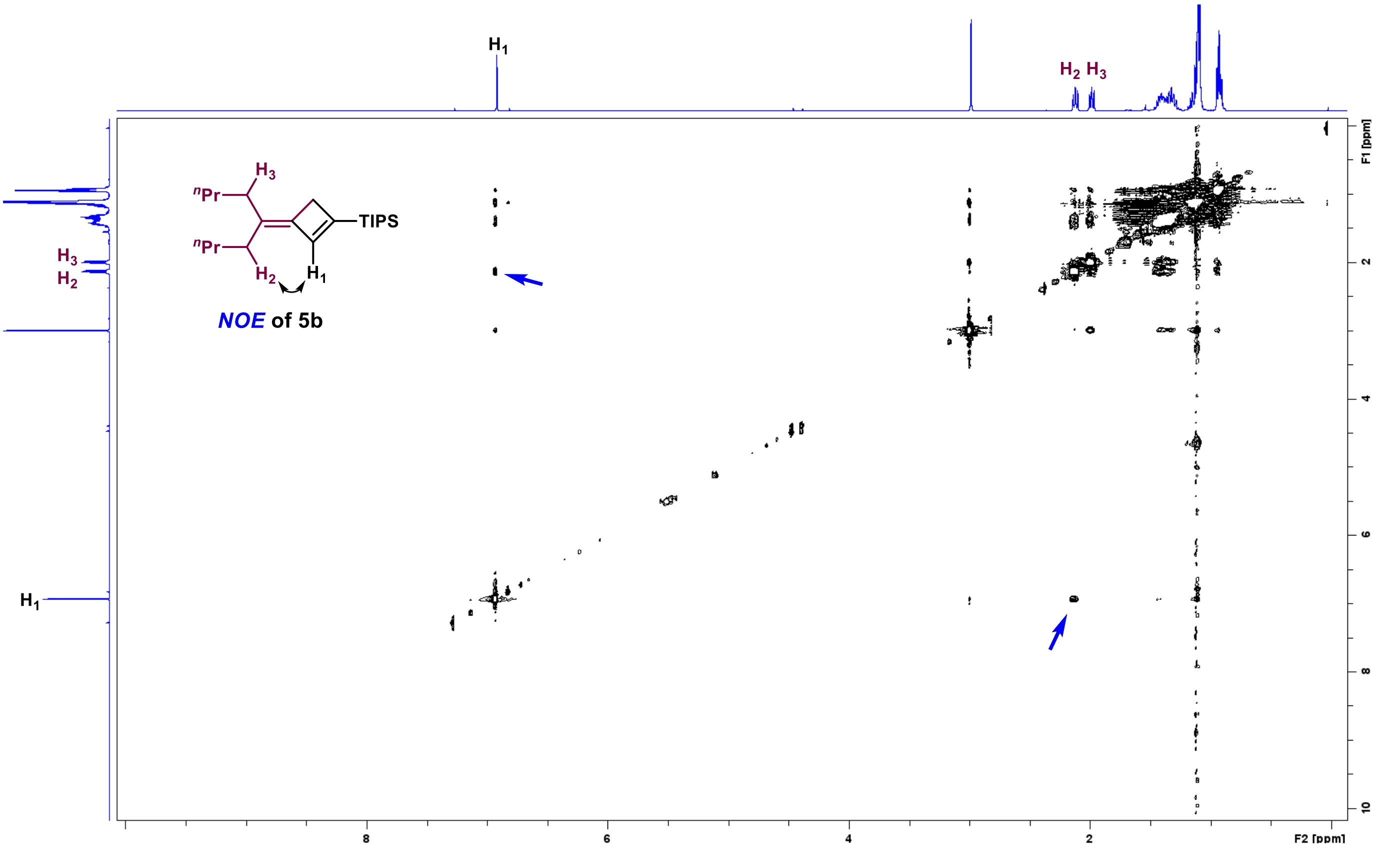
H1
H2 H3
H3
H2
H1
ⁿPr
ⁿPr
TIPS
NOE of 5b
F1 [ppm]
F2 [ppm]

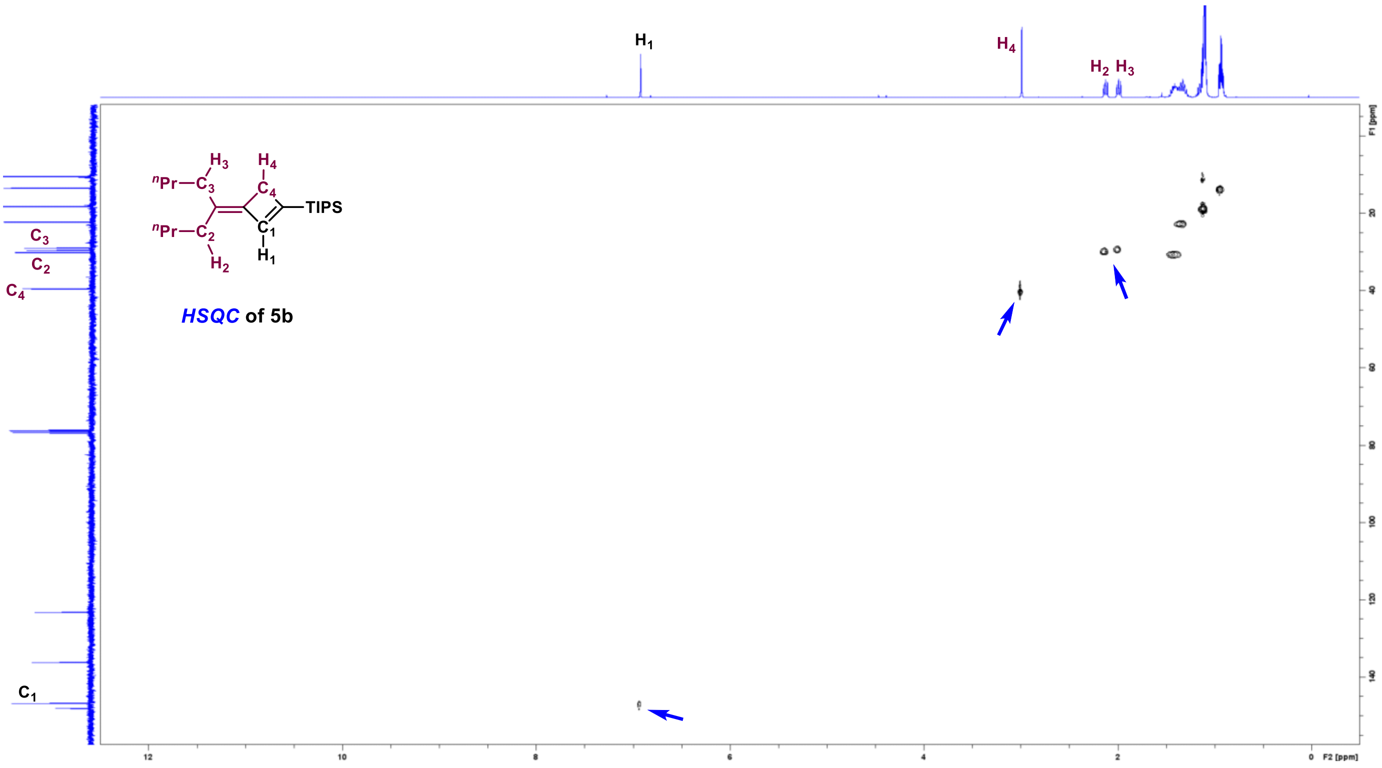
H1
H4
H2 H3
C3
C2
C4
C1
ⁿPr
ⁿPr
TIPS
HSQC of 5b
F1 [ppm]
F2 [ppm]

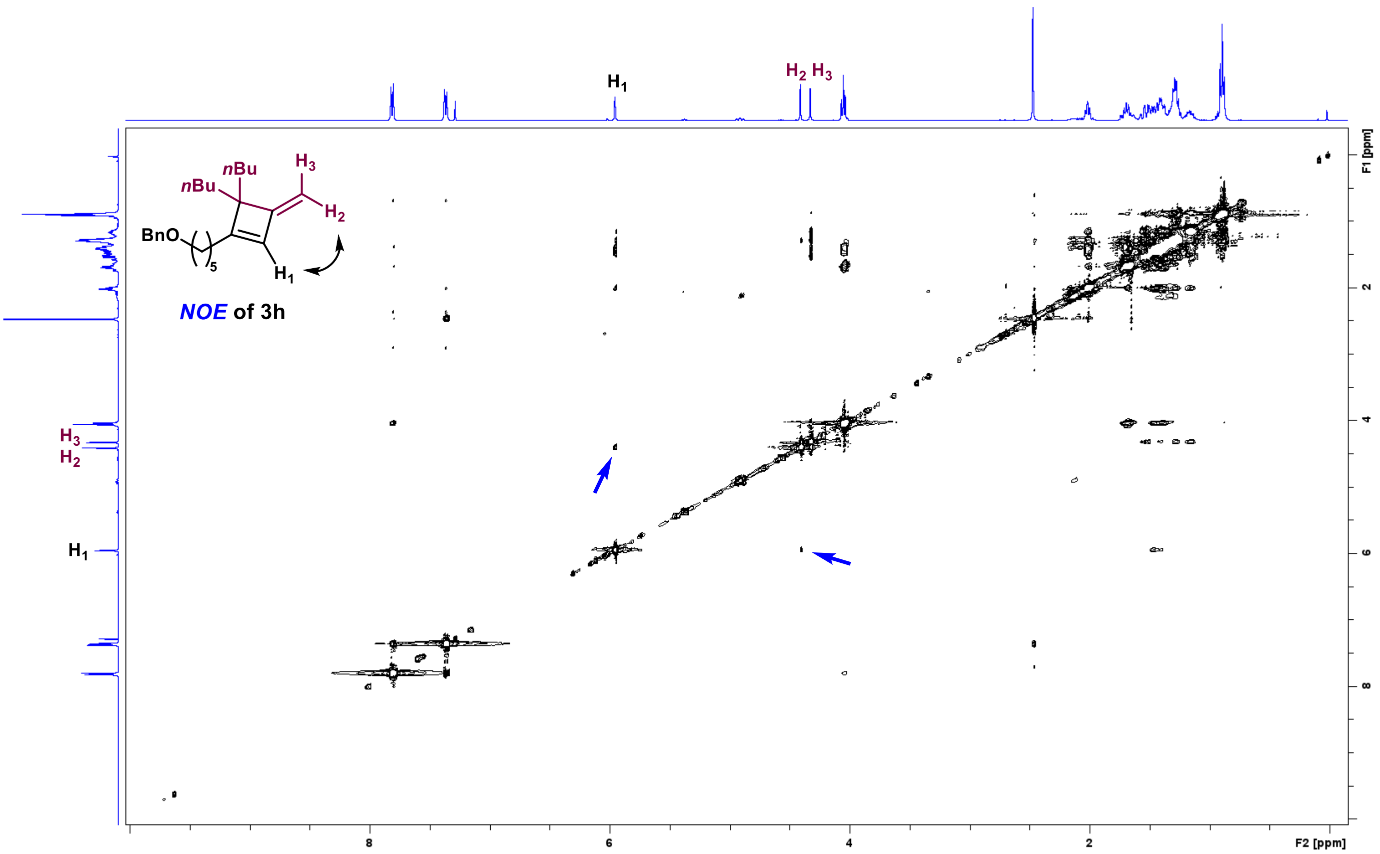
H1
H2 H3
nBu
nBu
H3
H2
BnO
5
H1
NOE of 3h
H3
H2
H1
F1 [ppm]
F2 [ppm]

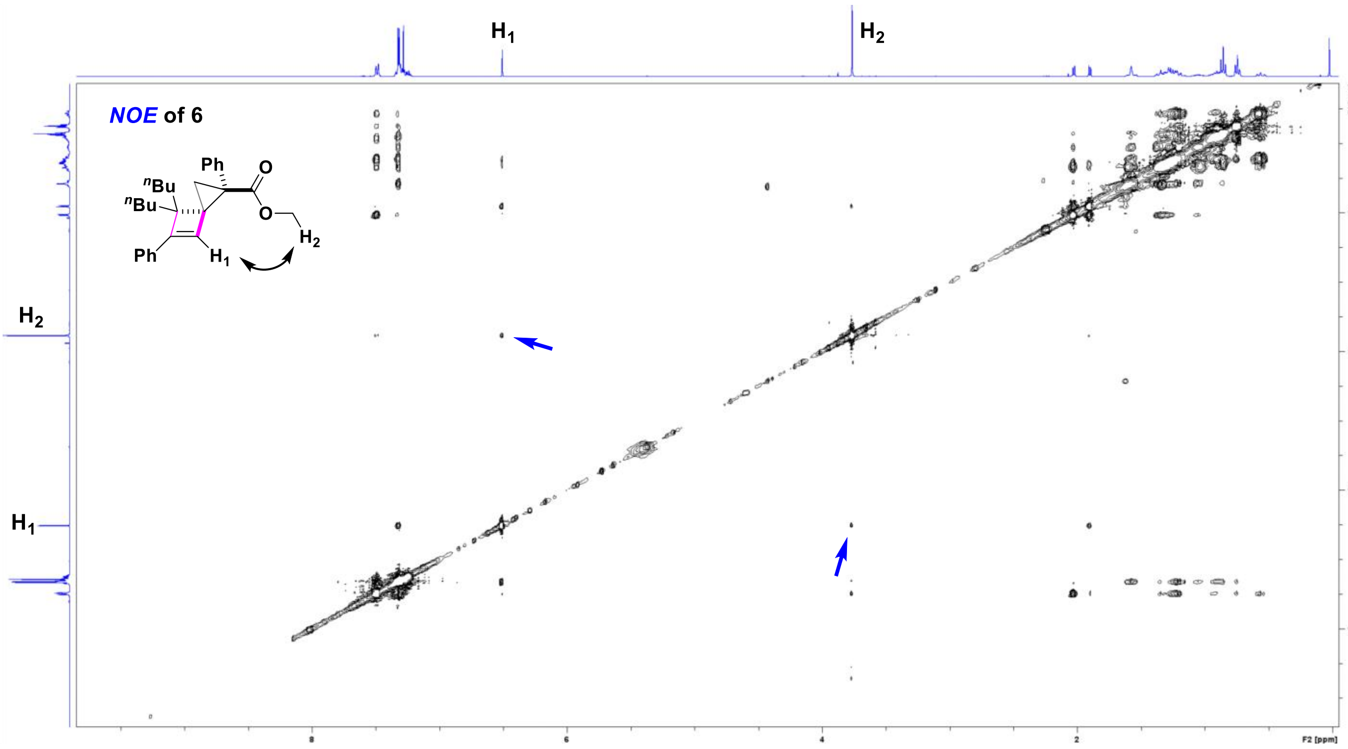
H1
H2
NOE of 6
Ph
O
nBu
nBu
O
H2
Ph
H1
H2
H1
F2 [ppm]
